# An expeditious route to sterically encumbered nonproteinogenic α-amino acid precursors using allylboronic acids†

**DOI:** 10.1039/d1sc06259j

**Published:** 2022-01-28

**Authors:** Samrat Sahu, Ganesh Karan, Lisa Roy, Modhu Sudan Maji

**Affiliations:** Department of Chemistry, Indian Institute of Technology Kharagpur Kharagpur 721302 India msm@chem.iitkgp.ac.in; Institute of Chemical Technology Mumbai, IOC Odisha Campus Bhubaneswar Bhubaneswar 751013 India

## Abstract

A diastereoselective allylation of *N-tert*-butane sulfinyl α-iminoesters using allylboronic acids is developed to obtain optically active non-proteinogenic α-amino acid precursors in good yields and diastereoselectivities. Gram-scale synthesis, broad tolerance of functional groups, excellent stereodivergence, post-synthetic modifications, and easy removal of the chiral auxiliary are some of the key highlights. The protocol is applicable to various amino acids and short peptides, resulting in the incorporation of these precursors at the N-terminal position.

## Abstract 2

A diastereoselective allylation of *N-tert*-butane sulfinyl α-iminoesters using allylboronic acids is developed to obtain optically active non-proteinogenic α-amino acid precursors in good yield and diastereoselectivities.

## Graphical abstract

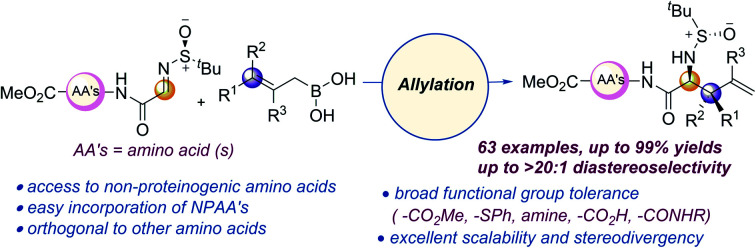

## Introduction

**Fig. 1:**
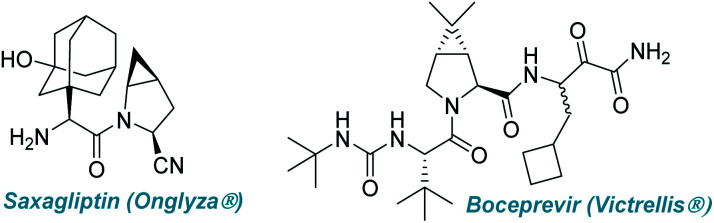
Peptide-based drug molecules.

With 60 peptide-based drugs in the market, over 150 in clinical trials, and another 400 in preclinical development, the peptide-based drug market is worth an estimated US$ 25.4 billion as of 2018 (Fig. 1).^1^ Over the past few years, optically pure non-proteinogenic α-amino acids (NPAA) have emerged as alternatives to fill the void that was left by their natural counterparts and can be used in many significant areas of biological importance, such as intermediates in biosynthesis, post-translational formation of proteins, and they have a significant role in drug discovery.^2^ The incorporation of unnatural amino acids into the peptide chain not only improves the metabolic stability but also increases the resistance to *in vivo* proteolysis under physiological conditions.^3^ Furthermore, α-amino acids with an unsaturated side chain have attracted considerable attention from medicinal chemists due to their easy integration into the protein structure.^2*a*,4^ They are also being used as chiral auxiliaries and ligands and, thus, are an important backbone in organic synthesis.^5^ However, their commercial unavailability, as well as the lack of a well-defined method to get all possible stereoisomers, remains a big challenge to date. Thus, the synthesis of these important building blocks with diverse side-chain structures is of great interest and, consequently, many methods have emerged for the synthesis of unnatural α-amino acids with allyl groups at the side chain.^6–8^

**Scheme 1:**
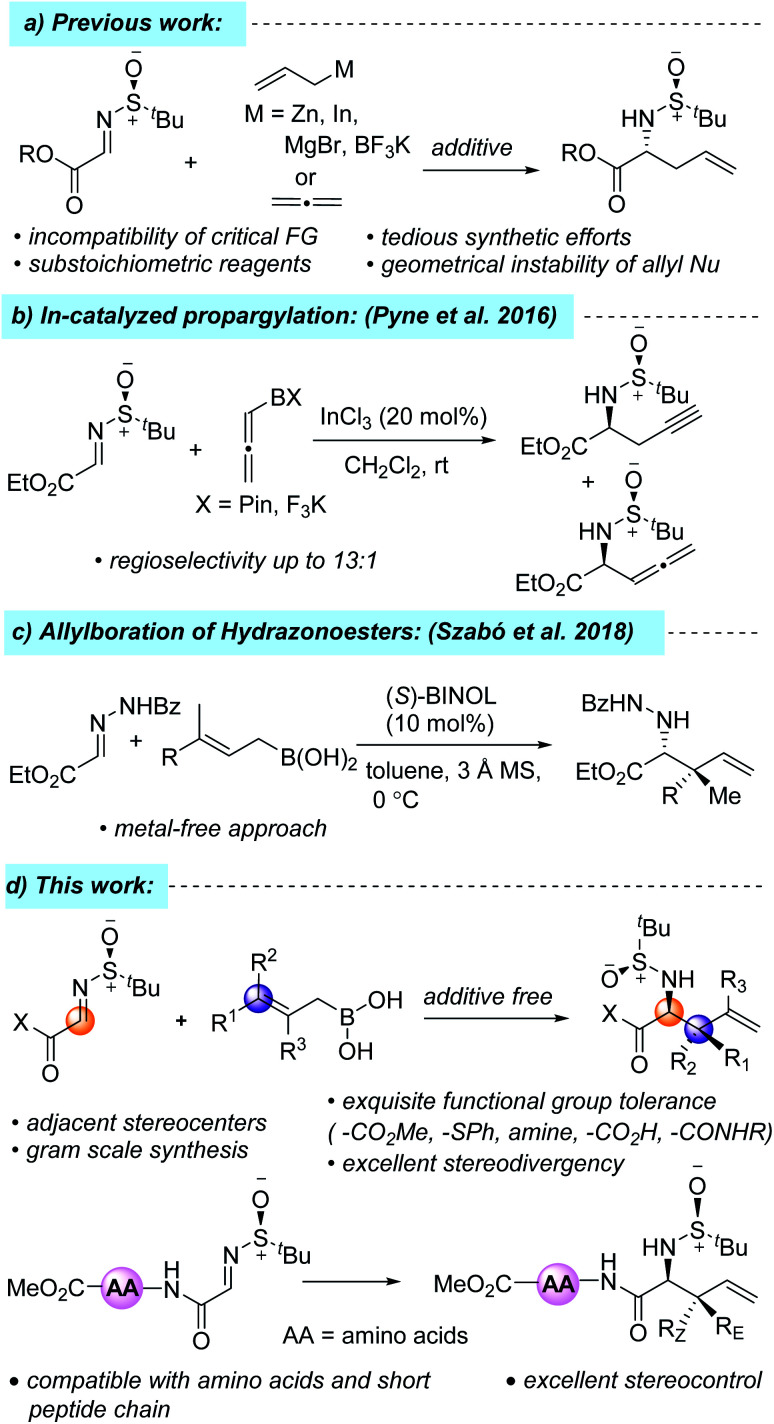
Literature reports and our work.

One of the commonly practiced methods of accessing these synthons is the diastereoselective allylation of α-iminoesters with different organozinc/indium/magnesium/tin/boronate reagents, where the diastereoselectivity is controlled by the chiral auxiliary group attached to the imine nitrogen atom (Scheme 1a).^7^ However, the incompatibility of several critical functional groups, the geometrical instability of allyl nucleophiles, tedious synthetic efforts, and the substoichiometric usage of organometallic reagents inspired chemists to look for other alternative reagents. For example, in 2016, Pyne *et al.* reported a highly regioselective and diastereoselective propargylation of *N*-sulfinyl-α-iminoester using pinacol allenylboronate and potassium allenyltrifluoroborate, which resulted in an inseparable mixture of both propargylated and allenylated products (Scheme 1b).^7*k*^ Recently, allylboronic acids have emerged as a powerful alternative to their allylboronate counterpart due to the more Lewis acidic boron atom, which results in a more organized transition state, leading to greater stereocontrol and, consequently, the diastereo- and enantioselective allylation of different acyclic and cyclic imines and hydrazonoesters has been developed.^9^ In 2018, Szabó and co-workers developed a metal-free, enantioselective allylboration of hydrazonoesters using allylboronic acids to get sterically hindered α-amino acid derivatives (Scheme 1c).^9*f*^ To the best of our knowledge, there has been no report on the diastereoselective allylation of *N-tert*-butane sulfinyliminoesters using allylboronic acids. In conjunction with our recent interest in the total synthesis of hapalindole alkaloids, herein, we report a mild, diastereoselective allylation protocol of *N-tert*-butane sulfinyliminoester using allylboronic acids and their incorporation into the N-terminal position of short peptide chains (Scheme 1d).^10^

## Results and discussion

At the outset of the optimization studies, imine 1a and geranyl boronic acid 2a were chosen as model substrates. Out of the solvents tested (Table 1, entries 1–4), CHCl_3_ was the solvent of choice, providing the allylated product 3aa in 71% yield with 18 : 1 diastereoselectivity at room temperature. Screening of other polar non-protic solvents, as well non-polar solvents, showed no further improvement in the outcome of the reaction (entries 5–8). A decrease, as well as an increase, in the reaction temperature diminished the yield of the reaction, although the diastereoselectivities remained almost unaltered (entries 9–11). Several additives, such as MeOH, HFIP and ^*t*^BuOH, were tested under the conditions, however the yields were lower, probably due to the formation of less reactive boronate esters (entries 12–14). To demonstrate the superior reactivity of allylboronic acid, a reaction was carried out using the corresponding pinacol boronate ester as an allylating agent. However, under the standard reaction conditions, no allylated product was observed even after a prolonged reaction time (entry 15).

**Table 1:** Optimization of the reaction conditions^a^

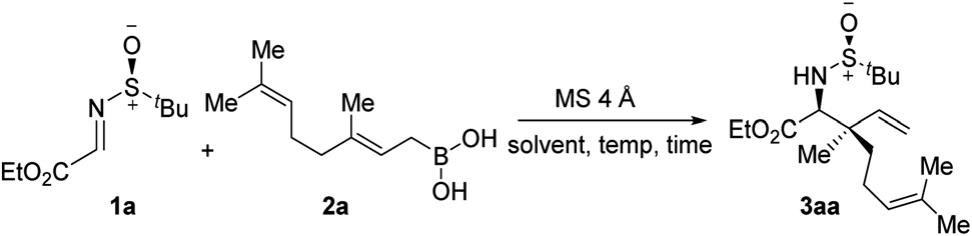					
Entry	Solvent	Temp. (°C)	Time (h)	Yield^b^ (%)	dr^c^
1	CHCl_3_	rt	5	71	18 : 1
2	CH_2_Cl_2_	rt	4	65	17 : 1
3	Toluene	rt	8	53	13 : 1
4	THF	rt	7	45	11 : 1
5	Benzene	rt	4	46	16 : 1
6	Cyclohexane	rt	8	32	13 : 1
7	DMF	rt	12	16	11 : 1
8	DMSO	rt	12	18	11 : 1
9	CHCl_3_	0	7	69	16 : 1
10	CHCl_3_	−30	10	63	17 : 1
11	CHCl_3_	60	1.5	36	17 : 1
12^d^	CHCl_3_	rt	12	34	8.7 : 1
13^e^	CHCl_3_	rt	12	42	9 : 1
14^f^	CHCl_3_	rt	12	47	5 : 1
15^g^	CHCl_3_	rt	24	—	—

a Reaction conditions: imine 1a (0.3 mmol), boronic acid 2a (0.39 mmol), MS 4 Å (150 mg), solvent (4.0 mL), rt, Ar.

b Isolated yields.

c Diastereomeric ratios (dr) were determined by ^1^H NMR analysis.

d MeOH as an additive.

e HFIP as an additive.

f ^*t*^BuOH as an additive.

g (*E*)-2-(3,7-dimethylocta-2,6-dien-1-yl)-4,4,5,5-tetramethyl-1,3,2-dioxaborolane (0.39 mmol) was used as the allylating agent instead of 2a. HFIP: 1,1,1,3,3,3-hexafluoro-2-propanol.

**Scheme 2:**
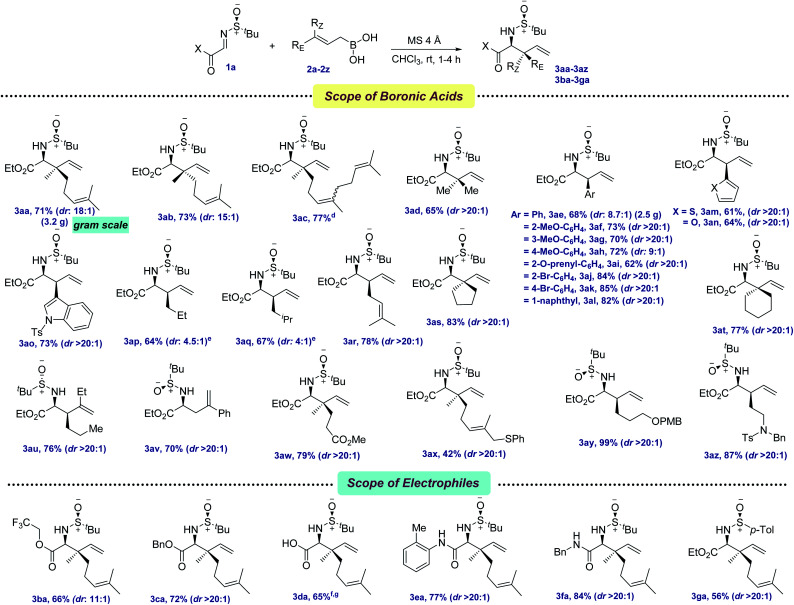
Scope of allylation with α-iminoesters^*a*,*b*,*c*^. ^*a*^ Reaction conditions: imine 1 (0.3 mmol), allylboronic acid 2 (0.39 mmol, 0.1 M in CHCl_3_), MS 4 Å (100–150 mg) at rt under Ar. ^*b*^ Diastereomeric ratios (dr) were determined by ^1^H NMR analysis. ^*c*^ Isolated yields. ^*d*^ Obtained as a mixture of *E*/*Z* isomers as present in commercially available farnesol. ^*e*^ Reactions were conducted at 60 °C. ^*f*^ Over two steps from glyoxalic acid. ^*g*^ Reaction was conducted at −30 °C.

With the optimized conditions in hand, we first evaluated the scope of allyl boronic acids (Scheme 2). Geranyl, neryl, farnesyl, and prenyl boronic acids were compatible under the reaction conditions, providing the allylated products in moderate yields and excellent diastereoselectivities (3aa–3ad, 65–77%). The mildness of the protocol enabled the synthesis of a wide range of electronically different phenylalanine analogs by the thoughtful choice of several functionalized cinnamyl boronic acids containing both electron-donating as well as withdrawing functional groups, such as –OMe, –Br, –O-prenyl in the benzene ring (3ae–3al, 62–85%). Since heteroaryl groups have long been utilized as a precursor of several key functionalities in various late-stage processes, thiophene, furan, and indole containing heteroaryl boronic acids were successfully coupled, providing the allylated products 3am–3ao in 61–73% yields. In the quest for synthesizing α-amino esters with a tertiary center at the β-position, several linear aliphatic allylboronic acids were next tested and, pleasingly, the allylation protocol provided the corresponding products 3ap–3ar in 64–78% yields. However, the diastereoselectivity was moderate for 3ap–3aq, probably due to the elevated temperature requirement for allylation.

NPAAs with cyclic side chains have long attracted keen interest due to their conformational restrictions, resulting from the constrained dihedral angles, which allow their selective identification by the target proteins.^11^ This not only stabilizes the secondary structure of the protein, but also improves their potency. The screening of several γ,γ′-cyclic boronic acids with various chain lengths revealed that with the increase in chain length, the yield of the allylation decreased, although the diastereoselectivity remained unchanged (3as–3at, 77–83%). Boronic acids with aryl and alkyl substitutions at the β,γ-positions were next tested for allylation and, to our joy, allylated products were isolated in moderate to good yields (3au–3av, 70–76%). One of the key limitations in the existing protocols of allylation was found to be the incompatibility of key functional groups. To solve this, allylboronic acids bearing –CO_2_Me, –SPh, –OPMB and tertiary amine functionalities were successfully incorporated as amino acid side chains (3aw–3az, 42–99%).

The tolerance of several allylboronic acids next prompted us to check the scope of the allylation protocol with respect to different electrophiles. Trifluoroethyl and benzyl esters were promptly allylated using 2a to furnish the corresponding 3ba–3ca in 66–72% yields. Direct access to the amino acid 3da was achieved by employing glyoxalic acid-derived sulfinyl imine in 65% yield over two steps. Delightfully, aryl and alkyl-based glyoxamides were also found to be compatible under the allylation conditions, providing 3ea–3fa in 77–84% yields. To check the scope of other chiral sulfinyl imines, we screened *N*-(*p*-tolylsulfinyl) iminoester 1g. Gratifyingly, the allylated product 3ga was obtained in 56% yield. The scalability of the developed protocol was demonstrated by synthesizing 3aa and 3ae in 3.5 g and 2.5 g, respectively.

**Scheme 3:**
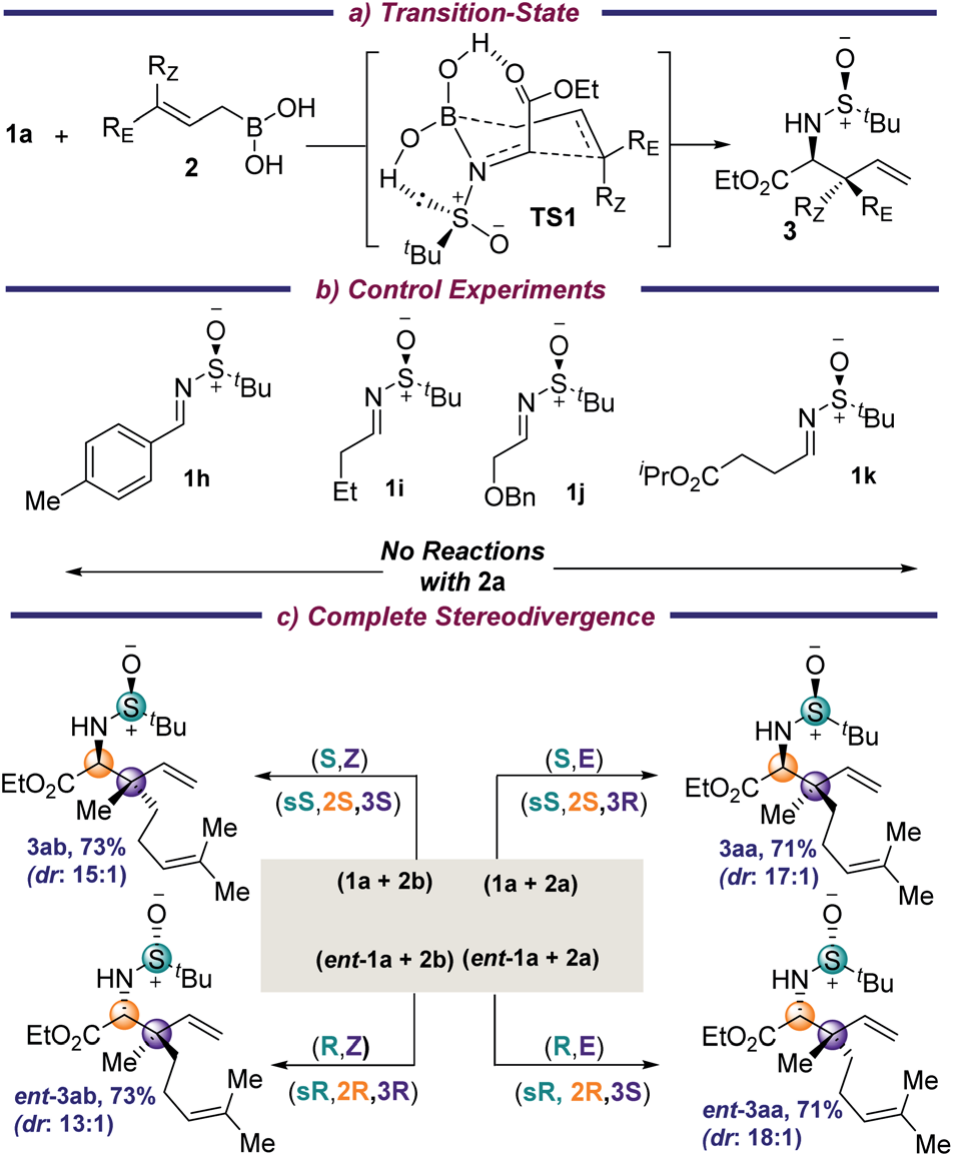
The transition state, control experiments, and stereodivergence.

The absolute stereochemistries of the allylation products 3ae, 3ak and 3av were determined by comparing with their literature data,^7*d*,8*a*^ and consequently all the other products 3 were assigned analogously. The reaction is thought to proceed through a six-membered transition state (TS1), in which the –CO_2_Et group and the lone pair on sulfur further stabilize the TS through hydrogen-bonding interactions with the boronic acid (Fig. S1, ESI†). Interestingly, the imine nitrogen coordinates to the boron atom prior to B–C(allyl) bond cleavage (Scheme 3a) due to the significant Lewis acidity of the allyl boronic center. Hence, this is a concerted mechanism of C(imine)–C(allyl) coupling together with B–C(allyl) bond cleavage. The computed Gibbs free-energy activation barrier at the B3LYP-D3BJ(CPCM)/6-311+G(d,p) level (17.4 kcal mol^−1^) is feasible under the experimental conditions and further validates the computational modelling studies.^12^ This was further supported by the fact that changing the iminoesters to the aryl or alkyl aldehyde derived imines 1h and 1i did not result in any corresponding allylated products under the standard conditions (Scheme 3b). This is probably due to the diminished electrophilicity of the imines and the absence of additional interaction of carbonyl with the boronic acid moiety. Additionally, removing the carbonyl moiety, as well as placing the ester group away from the α-position of the imine (1j and 1k, respectively), did not result in any product, which indicates that the hydrogen-bonding to the boronic acid in a chelating fashion in the TS might be crucial for transformation. A complete stereodivergency to get all possible stereoisomers is often found to be of great importance in medicinal chemistry to examine the on- and off-target interactions in drug design for an improved therapeutic profile. To demonstrate this, the geometrically isomeric allylboronic acids 2a and 2b were reacted with the enantiomeric imines 1a and *ent*-1a and, to our joy, all four sets of stereoisomers were isolated in good yields and diastereoselectivities (Scheme 3c).

The tolerance of amides in the electrophile (3ea and 3fa) prompted us to examine the scope of amino acids and short peptides at the backbone. To do so, we needed to generate an aldehyde at the N-terminal position of peptides, which was achieved by the periodate-mediated oxidative deamination of a free serine residue and later converting it to imine. Recently, various functionalities, such as silyl acetals, 2,4-thiazolidines, α-hydroxy ketones, allyl nucleophiles *etc.*, have been tagged to this aldehyde at the N-terminal position of the protein and peptides with the aldehyde generated from a serine residue.^13^ However, some of the key limitations were found to be the acidic conditions, slow kinetics, usage of harmful catalysts, high concentration, stereochemically non-selective conjugation, and poor stability of the conjugates due to the reversibility, which often create challenges for their further application in biological systems.

**Scheme 4:**
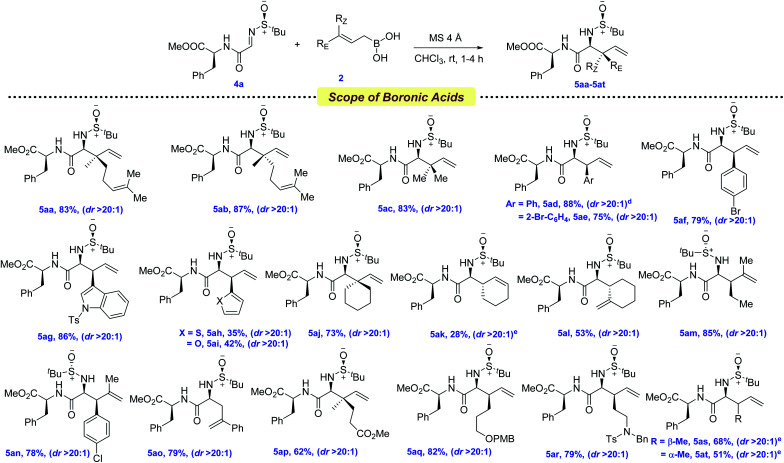
Scope of allylboronic acids for the synthesis of dipeptides^*a*,*b*,*c*^. ^*a*^ Reaction conditions: imine 4a (0.1 mmol, 1.0 equiv.), allylboronic acid 2 (0.25 mmol, 2.5 equiv.), MS 4 Å (100–150 mg), CHCl_3_ at rt under Ar. ^*b*^ Diastereomeric ratios (dr) were determined by ^1^H NMR analysis. ^*c*^ Isolated yields. ^*d*^ Reaction was conducted at −50 °C. ^*e*^ Reaction was conducted at 60 °C.

To evaluate whether our newly developed protocol could successfully be applied to peptide systems, we screened several allylboronic acids with the peptide derived imine 4a (Scheme 4). In general, it showed a broad range of substrate diversity, in line with the previous scope. Geranyl, neryl, and prenyl boronic acids were successfully coupled with admirable yields and diastereoselectivities, providing the dipeptides 5aa–5ac in 83–87% yields. A similar reactivity was observed for various substituted cinnamyl boronic acids, providing 5ad–5af in 75–88% yields, although this required a low reaction temperature in some cases. Next, heteroaryl, cyclohexyl, and cyclohexenyl boronic acids were subjected to allylation and, due to the increased steric bulk, the latter required a higher reaction temperature (5ag–5ak, 28–86% yields). Gratifyingly, products 5al–5ao were isolated in 53–85% yields when β,γ-disubstituted boronic acids were employed.

One of the important parameters in any chemical transformation is the introduction of several functionalities to change the entire portfolio, as well as providing a synthetic handle for further late-stage functionalization. In this current viewpoint, allylboronic acids bearing CO_2_Me, –OPMB and –N(Bn)Ts were thoughtfully coupled at the N-terminal position of the dipeptide, providing even more diversification (5ap–5ar, 62–82% yields). To access dehydroisoleucine and its isomer, a pair of geometrically isomeric crotyl boronic acids were next tested for this protocol and, to our delight, both of them provided the allylated products 5as–5at in 51–68% yields.

**Scheme 5:**
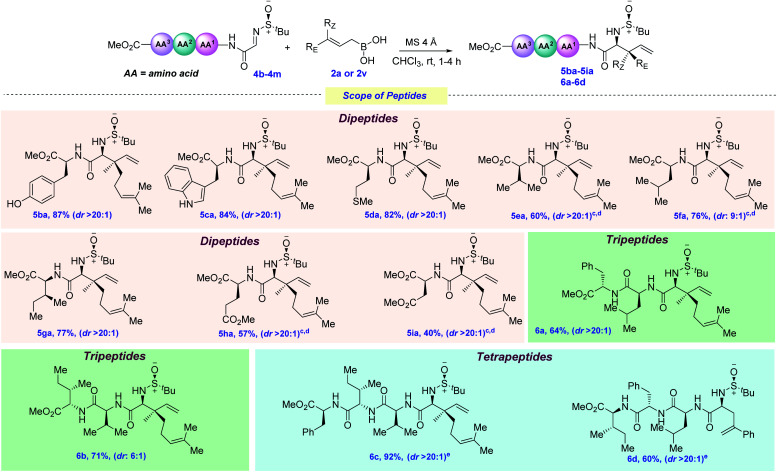
Scope of peptides^*a*,*b*^. ^*a*^ Reaction conditions: imine 4 (0.1 mmol, 1.0 equiv.), allylboronic acid 2a or 2v (0.25 mmol, 2.5 equiv.), MS 4 Å (100–150 mg), CHCl_3_ at rt under Ar. ^*b*^ Diastereomeric ratios (dr) were determined by ^1^H NMR analysis. ^*c*^ Yield is calculated over four steps starting from the (*N*)-Boc protected peptide without isolating the imine. ^*d*^ The reaction was carried out on a 0.3 mmol scale. ^*e*^ The reaction was carried out on a 0.057 mmol scale.

To check the effect of the amino acid side chain on allylation, next, we screened several varieties of peptide-derived imines (Scheme 5). The phenolic hydroxyl group (Tyr), –NH free indole (Trp) and –SMe (Met) were elegantly allylated using 2a, thus giving 5ba–5da in 82–87% yields. This is particularly remarkable, since each of these amino acids possesses several nucleophilic sites, as well electrophilic ones (especially for Trp), that could have shown interference with the incoming allyl nucleophile. Next, amino acids bearing non-polar aliphatic side chains, such as valine, leucine, and isoleucine, were tested under this allylation protocol and, to our joy, they were also efficaciously allylated in good yields and moderate to excellent diastereoselectivities (5ea–5ga, 60–77% yields). Next, glutamate and aspartate-derived dipeptide imines bearing polar chains were elegantly allylated at the N-terminus position (5ha–5ia, 40–57%). This broad range of functional group tolerance, regioselectivity and chemoselectivity inspired us to evaluate the effect of the increase in chain length on peptide functionalization. Adding one more amino acid (such as Phe for 4j and Ile for 4k) at the C-termini of the existing dipeptides did not have any detrimental effect on the outcome of the reaction, delivering 6b in 64–71% yields. By further increasing the chain length, tetrapeptides 6c–6d could also be synthesized (60–92% yields). However, for 4m, allylation using 2a was found to not be diastereoselective, presumably due to the steric congestion exerted by the long amino acid chain and geranylboronic acid. Changing to the less sterically hindered 2v provided 6d as a single diastereomer.

**Scheme 6:**
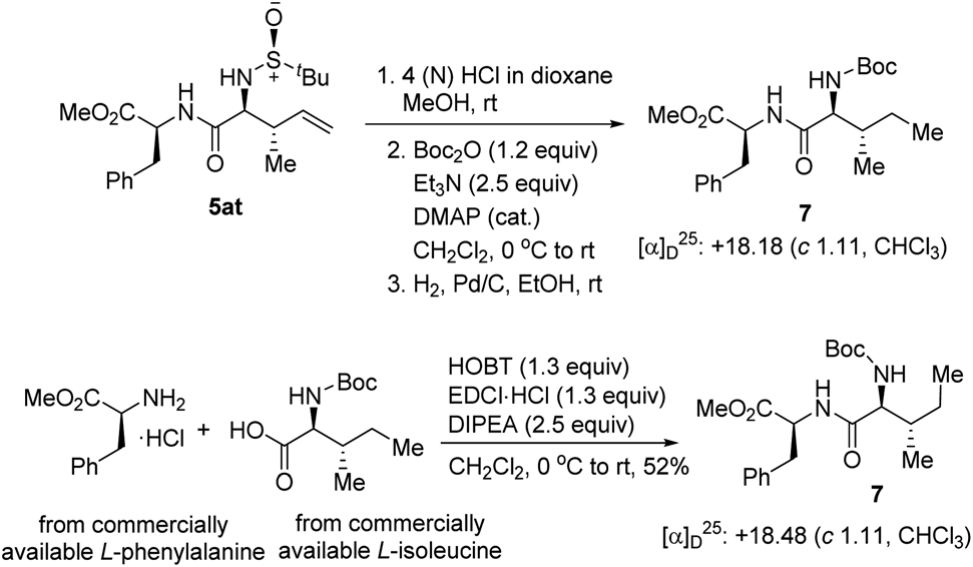
Removal of the chiral auxiliary and determination of the absolute configuration.

To re-confirm the absolute stereochemistry, we deprotected the *N-tert*-butyl sulfinyl moiety, re-protected with Boc_2_O, and hydrogenated the double bond of 5at to get the dipeptide 7, and compared its analytical data with the same obtained from the dipeptide 7 synthesized *via* a second route using commercially available l-isoleucine and l-phenylalanine (Scheme 6). This confirmed the newly generated stereocenters as (2*S*,3*S*) in 5at and, consequently, the stereochemistries of all the other compounds were assigned analogously.

**Scheme 7:**
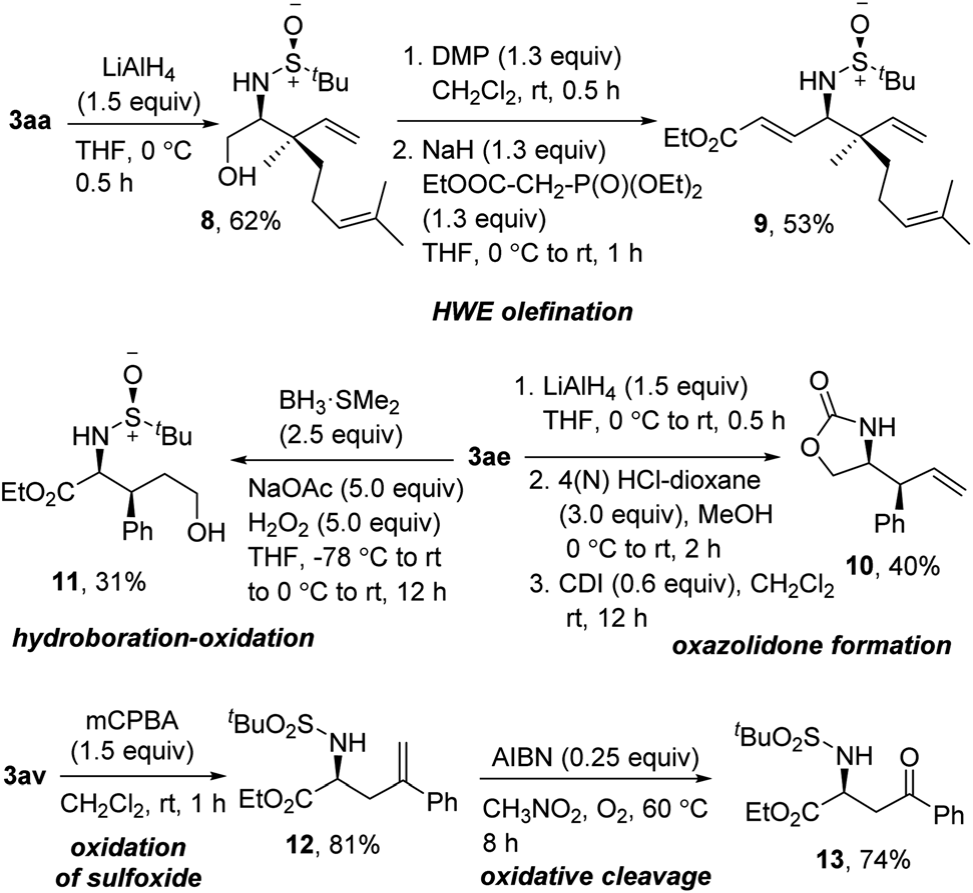
Post-synthetic modification of homoallylic amines.

To demonstrate the synthetic utility of the synthesized product, we performed some functional group modifications (Scheme 7). At first, the reduction of the ester in 3aa resulted in the protected 1,2-amino alcohol 8 in 62% yield. Dess–Martin periodinane oxidation and subsequent Horner–Wadsworth–Emmons olefination delivered the γ-amino-α,β unsaturated ester 9 in 53% yield over two steps. Furthermore, a three-step reduction, deprotection, and oxazolidone formation using 1,1′-carbonyldiimidazole (CDI) on 3ae provided the 2-oxazolidone 10 in 40% yield over three steps. To functionalize the alkene moiety, next, 3ae was subjected to sequential hydroboration–oxidation using BH_3_·SMe_2_ to get the 1,4-amino alcohol 11 in 31% yield. Finally, oxidation of the sulfoxide was smoothly carried out on 3av without affecting the terminal alkene, delivering 12 in 81% yield. The oxidative cleavage of 12 using AIBN under O_2_ provided the ketone 13 in 74% yield.^14^

## Conclusions

In conclusion, we have developed a mild protocol for accessing sterically encumbered non-proteinogenic α-amino acid precursors by the diastereoselective allylation of α-iminoesters using allylboronic acids. The ready reactivity, geometrical stability, and easy access from the corresponding allyl alcohols generated a wide variety of structurally diverse amino acid precursors with functional side chains for late-stage synthetic modifications. By employing this concept, we have also shown the incorporation of several structurally diverse NPAAs into the peptide chain at the N-terminal position with excellent chemo-, regio-, and stereoselectivity. A quick survey shows that the method can be applied to short protected peptide chains, keeping other functional side chains intact and with precise stereo-control. The synthetic value of the products has been demonstrated by several key transformations. We believe that this might pave the pathway for future functionalization techniques.

## Data availability

We have deposited all the experimental or computational data as ESI.†

## Author contributions

S. S. and G. K. performed all the experiments in the laboratory. All the authors contributed to the design of the experiments, discussion, and preparation of the manuscript. L. R. performed the DFT calculations.

## Conflicts of interest

There are no conflicts to declare.

## Supplementary Material

SC-013-D1SC06259J-s001
