# Supplementary material for: An expeditious route to sterically encumbered nonproteinogenic α-amino acid precursors using allylboronic acids
Source: Chem Sci. 2022 Jan 28;13(8):2355–62. doi: 10.1039/d1sc06259j (PMC8864702; doi:10.1039/d1sc06259j)
Supplement: SC-013-D1SC06259J-s001 [file SC-013-D1SC06259J-s001.pdf]

Electronic Supplementary Material (ESI) for Chemical Science.
This journal is © The Royal Society of Chemistry 2022

Supporting Information

An Expeditious Route to Sterically Encumbered Nonproteinogenic α-Amino Acid Precursors Using Allylboronic Acids

Samrat Sahu,[a] Ganesh Karan,[a] Lisa Roy,[b] and Modhu Sudan Maji*[a]

[a]Department of Chemistry, Indian Institute of Technology Kharagpur, Kharagpur-721302, India.

[b]Institute of Chemical Technology Mumbai, IOC Odisha Campus Bhubaneswar, Bhubaneswar 751013, India.

Email: msm@chem.iitkgp.ac.in

**Table of Contents**	**Page No.**
Experimental Section	**S1**
General Procedure (GP I): The Synthesis of Imines and Characterization Data (**1b-1g**)	**S2-S4**
General Procedure (GP II): The Synthesis of Peptide Based Imines and Characterization Data (**4b-4d, 4g**)	**S4-S6**
General Procedure (GP III): The Synthesis of Peptide based Imines **4** and Characterization Data (**4a**, **4j-4m**)	**S6-S9**
Synthesis of Known Allylboronic Acids **2**	**S9-S10**
General Procedure (GP IV): The Synthesis of New Allylboronic Acids	**S10-S11**
General Procedure (GP V): The Allylation of Sulfinyl Imines and Characterization Data (**3, 5-6**)	**S11-S42**
Comparison of data of **5aa** obtained from allylation and late-stage peptide coupling	**S43-S45**
Comparison of ^{1}H NMR data of **3ae** and **3ak**	**S45-S46**
Deprotection of Sulfinyl Group, determination of the absolute configuration and post-synthetic modifications	**S46-S52**
Computational Details	**S52-S59**
References	**S60**
^{1}H and ^{13}C NMR Spectra of All Compounds	**S61-S236**

Experimental Section:

General: All reactions involving air or moisture sensitive reagents were carried out in flame dried glassware under nitrogen or argon atmosphere. Chloroform was obtained from Merck India. All other solvents were obtained from Merck India and were dried according to the standard literature procedure. All the required solvents were degassed by purging nitrogen gas through solution under sonication for 2 hours. Reactions were monitored by thin layer chromatography (TLC) using Merck silica gel 60 F254 pre-coated plates (0.25 mm), and visualized under UV light or dipping into $KMnO_4$ solution. Silica gel (particle size 100-200 mesh) and neutral alumina were purchased from SRL India for performing column chromatography by using mixture of hexanes and ethylacetate eluent. Ethyl glyoxalate (50% in toluene) was purchased from Alfa Aesar Pvt. Ltd and was used as received. The ^{1}H NMR spectroscopic data were recorded with a Bruker 400, 500 or 600 MHz NMR instruments. ^{13}C NMR spectra were similarly recorded at 100, 125 or 150 MHz NMR instruments by broadband decoupled mode. The ^{1}H NMR spectroscopic data are reported relative to either $CDCl_3$ (7.26 ppm) or DMSO-d_6 (2.50 ppm). Proton and carbon NMR chemical shifts (δ) are reported in parts per million (ppm) relative to residual proton signals in $CDCl_3$ (δ = 7.26, 77.16) and DMSO-d_6 (δ = 2.50, 39.52). Coupling constants (*J*) are reported in Hertz (Hz) and refer to apparent multiplicities. The following abbreviations are used for the multiplicities: s: singlet, d: doublet, t: triplet, q: quartet, quint: quintet, dd: doublet of doublets, dt: doublet of triplets, ddd: doublet of doublet of doublets, tt: triplet of triplets, td: triplet of doublets, dtd: doublet of triplet of doublets, m: multiplet, br: broad. Specific rotations were measured on a JASCO polarimeter and have the unit deg cm^3 dm^{-1} g^{-1}. Concentration in bracket is given in gm/100 mL. Infrared (IR) spectra were recorded by Perkin Elmer ATIR spectrometer, and reported in terms of wave number (cm^{-1}). High resolution mass spectra (HRMS) were recorded in ESI (+ Ve) method.

General Procedure (GP I) for the Synthesis of Imines (1b-1g):

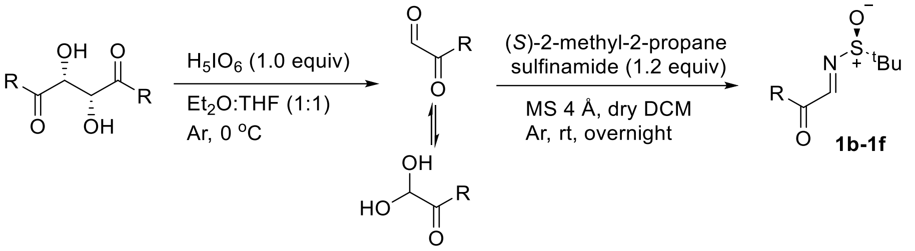

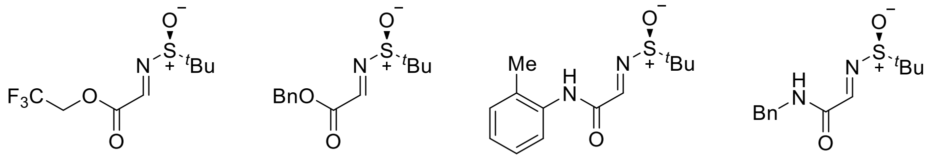

1b **1c** **1e** **1f**

To a solution of corresponding (*L*)-tartarates (1.0 mmol, 1.0 equiv) in dry Et_2O/THF (1:1, 8.0 mL) at 0 °C under argon, was added periodic acid (1.1 mmol, 1.1 equiv) in two portions. The reaction mixture was allowed to stir for 0.5 to 24 h upon which it was diluted by adding Et_2O and filtered. The crude mixture was concentrated and re-dissolved in dry DCM and used for next step without further purification.

GP IA: A 10 mL sealed tube was charged with (*S*)-2-methyl-2-propanesulfinamide (1.2 mmol) and activated 4 Å molecular sieves (0.1 g/mmol) and 5 mL dry DCM was added. After addition of freshly prepared crude glyoxalate solution in 1 mL of dry DCM and 15 μL of pyrrolidine, the resulting mixture was stirred at 60 °C for 6 hours. Upon cooling, reaction mixture was filtered through celite pad and washed with DCM. The crude product was purified by silica gel column chromatography using ethylacetate/ hexane eluent.

GP IB: To a solution of (*S*)-2-methyl-2-propanesulfinamide (1.2 mmol) and activated 4 Å molecular sieves (0.1 g/mmol) in dry DCM (5 mL), the corresponding glyoxalate/glyoxamides solution in 1 mL dry DCM was added. The resulting solution was stirred at room temperature under argon atmosphere. Upon completion, as evidenced by the TLC, reaction mixture was filtered through celite pad and washed with DCM. The crude mixture was purified on silica gel column to get the pure imine **1b-1f**.

2,2,2-Trifluoroethyl (*S*,*E*)-2((*tert*-butylsulfinyl)imino)acetate (1b):

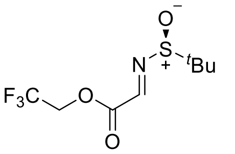

Imine **1b** was synthesized according to **GP IA** and isolated as colourless oil after column chromatography on silica using 8% ethylacetate in hexane as eluent (212 mg, 41% over two steps). **^{1}H NMR** (600 MHz, $CDCl_3$): δ = 8.05 (s, 1H), 4.72-4.61 (m, 2H), 1.27 (s, 9H) ppm. **^{13}C NMR** (151 MHz, $CDCl_3$): δ = 159.37, 153.59, 122.58 (q, *J* = 277.3 Hz), 61.56 (q, *J* = 37.4 Hz), 59.62, 22.85 ppm. **^{19}F NMR**: -73.62 ppm. **IR (film): ν_{max}** = 1732, 1665, 1324, 1245, 1145, 1095 cm^{-1}. **HRMS-ESI:** calculated for $C_8H_{13}F_3NO_3S$ $[M+H]^+$ 260.0563; found 260.0561. **$[\alpha]_D^{25}$**: +33.75, (c 0.542, $CHCl_3$).

Benzyl (*S*,*E*)-2-((*tert*-butylsulfinyl)imino)acetate (1c):

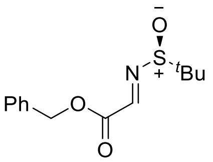

Imine **1c** was synthesized according to **GP IB** and isolated as colourless oil after column chromatography on silica using 5% ethylacetate in hexane as eluent (133 mg, 25% over two steps). **^{1}H NMR** (500 MHz, $CDCl_3$): δ = 8.03 (s, 1H), 7.40 – 7.32 (m, 5H), 5.33 (s, 2H), 1.25 (s, 9H) ppm. **^{13}C NMR** (126 MHz, $CDCl_3$): δ = 160.93, 155.35, 134.76, 128.75, 128.49, 67.93, 59.02, 22.77 ppm. **IR (film): ν_{max}** = 1734, 1639, 1452, 1265, 1131, 1083 cm^{-1}. **HRMS-ESI:** calculated for $C_{13}H_{17}NNaO_3S$ $[M+Na]^+$ 290.0821; found 290.0817. **$[\alpha]_D^{25}$**: +53.21 (c 0.487, $CHCl_3$).

(*S*,*E*)-2-((*tert*-Butylsulfinyl)imino)-*N*-(*o*-tolyl)acetamide (1e):

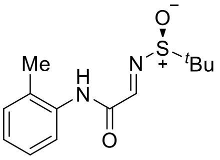

Imine **1e** was synthesized according to **GP IB** and isolated as colourless oil after column chromatography on silica using 12% ethylacetate in hexane as eluent (223 mg, 42% over two steps). **^{1}H NMR** (600 MHz, $CDCl_3$): δ = 8.62 (s, 1H), 8.10 (d, *J* = 8.1 Hz, 1H), 8.05 (s, 1H), 7.26 (t, *J* = 8.0 Hz, 1H), 7.22 (d, *J* = 7.4 Hz, 1H), 7.11 (t, *J* = 7.4 Hz, 1H), 2.31 (s, 3H), 1.30 (s, 9H) ppm. **^{13}C NMR** (151 MHz, $CDCl_3$): δ = 159.31, 158.14, 134.68, 130.69, 127.85, 127.20, 125.56, 121.48, 59.00, 22.83, 17.46 ppm. **IR (film): ν_{max}** = 3524, 1675, 1652, 1425, 1124, 1091 cm^{-1}. **HRMS-ESI:** calculated for $C_{13}H_{19}N_2O_2S$ $[M+H]^+$ 267.1162; found 267.1171. **$[\alpha]_D^{25}$**: +13.25 (c 0.421, $CHCl_3$).

(*S*,*E*)-*N*-Benzyl-2-((*tert*-butylsulfinyl)imino)acetamide (1f):

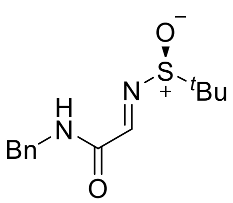

Imine **1f** was synthesized according to **GP IB** and isolated as colourless oil after column chromatography on silica using 10% ethylacetate in hexane as eluent (292 mg, 55% over two steps). **^{1}H NMR** (400 MHz, $CDCl_3$): δ = 8.00 (s, 1H), 7.39 – 7.35 (m, 2H), 7.33 – 7.30 (m, 3H), 7.10 (br s, 1H), 4.65 (dd, *J* = 14.9, 6.4 Hz, 1H), 4.51 (dd, *J* = 14.9, 5.7 Hz, 1H), 1.24 (s, 9H) ppm.

^{13}C **NMR** (151 MHz, $CDCl_3$): δ = 160.79, 158.80, 137.39, 129.02, 128.00, 127.90, 58.70, 43.63, 22.83 ppm. **IR (film): ν_{max}** = 3421, 1683, 1641, 1412, 1127, 1074 cm^{-1}. **HRMS-ESI:** calculated for $C_{13}H_{19}N_2O_2S$ $[M+H]^+$ 267.1162; found 267.1158. **$[\alpha]_D^{25}$**: +56.23 (c 0.254, $CHCl_3$).

General Procedure (GP II): The Synthesis of Peptide Based Imines:

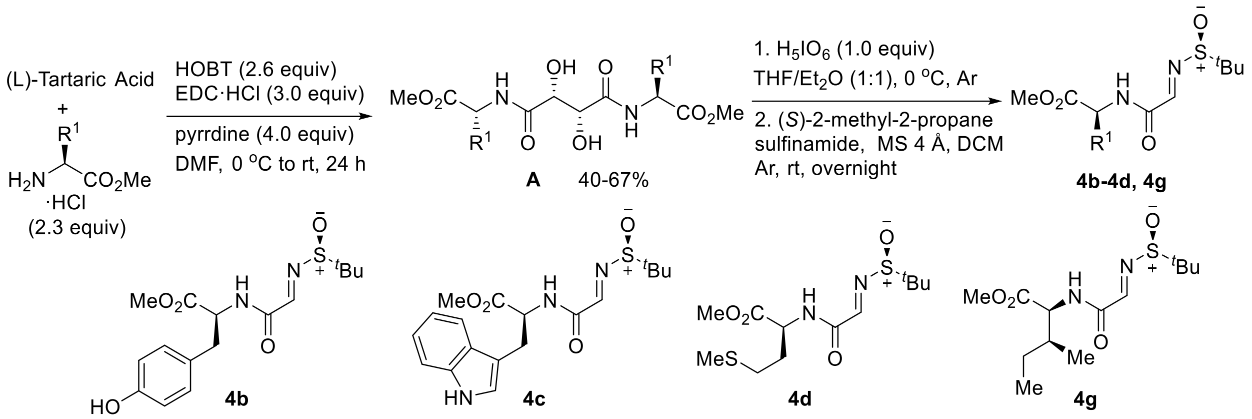

To a solution of amino acid methyl esters in dry DMF at 0 °C (ice bath) was added pyridine and it was continued to stir for 10 min. Then tartaric acid and HOBT was added sequentially at 0 °C and stirred for another 20 min. Next, EDC·HCl was added at 0 °C and the reaction mixture was allowed to come to room temperature slowly and stirred further over 24 h. After completion, the reaction was quenched with water and extracted with ethylacetate. The organic layer was washed with saturated aq. citric acid solution followed by 0.5 (N) HCl and *satd.* brine solution. Finally, the organic layer was dried over anhydrous Na_2SO_4, solvent was removed under *vacuo* and purified by silica gel column chromatography to obtain the coupled tartarates **A** in 40-67% yields.

To a solution of tartarate **A** (1.0 mmol) in THF/Et_2O (1:1, 5 mL) at 0 °C was added H_5IO_6 (1.0 mmol) under argon atmosphere. The reaction was stirred at the same temperature for 30 min. Then it was diluted with Et_2O and filtered through celite pad. The solvent was removed under reduced pressure, the crude residue was re-dissolved in 2 mL dry DCM and added to a stirred solution of (*S*)-2-methyl-2-propanesulfiamide (1.00 mmol) containing 4 Å MS in 4 mL dry DCM under argon. Upon completion of the imine formation, the reaction aliquot was filtered through celite, washed with DCM, and purified by silica gel column chromatography to get the pure imines **4b-4e**.

Methyl ((*E*)-2-(((*S*)-*tert*-butylsulfinyl)imino)acetyl)-*L*-tyrosinate (4b):

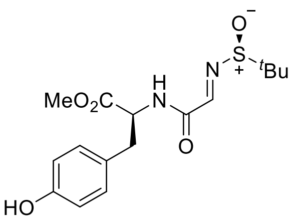

Imine **4b** was synthesized according to **GP II** and isolated as colourless oil after column chromatography on silica using 25% ethylacetate in hexane as eluent (177 mg, 25% over two steps). **^{1}H NMR** (400 MHz, $CDCl_3$): δ = 7.91 (s, 1H), 7.29 (d, *J* = 8.3 Hz, 1H), 6.93 (d, *J* = 8.2 Hz, 2H), 6.72 (d, *J* = 8.1 Hz, 2H), 6.46 (br s, 1H), 4.87 (dt, *J* = 8.0, 5.7 Hz, 1H), 3.75 (s, 3H), 3.15 (dd, *J* = 14.1, 5.5 Hz, 1H), 3.08 (dd, *J* = 14.1, 5.9 Hz, 1H), 1.22 (s, 9H) ppm. **^{13}C NMR** (101 MHz, $CDCl_3$): δ = 171.59, 160.26, 157.96, 155.58, 130.42, 126.74, 115.78, 59.12, 53.44, 52.77, 37.06, 22.78 ppm. **IR (film): ν_{max}** = 3421, 1735, 1652, 1354, 1276, 1091 cm^{-1}. **HRMS-ESI:** calculated for $C_{16}H_{23}N_2O_5S$ $[M+H]^+$ 355.1322; found 355.1335. **$[\alpha]_D^{25}$**: +65.20 (c 0.125, $CHCl_3$).

Methyl ((*E*)-2-(((*S*)-*tert*-butylsulfinyl)imino)acetyl)-*L*-tryptophanate (4c):

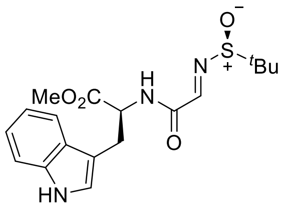

Imine **4c** was synthesized according to **GP II**. *Note: the corresponding glyoxamide is very unstable in presence of air and show significant decomposition upon exposing to air. After the completion of the first step, the round bottom flask was kept at the 0 °C without stirring for the sedimentation. After 15 min, the top layer was taken in a syringe and added directly to the solution of sulfonamide in DCM without purification for imine synthesis.* The imine **4c** was isolated as colourless oil after column chromatography on silica using 30% ethyl acetate in hexane as eluent (150 mg, 20% over two steps). **^{1}H NMR** (500 MHz, $CDCl_3$): δ = 8.16 (br s, 1H), 7.92 (s, 1H), 7.52 (d, *J* = 7.9 Hz, 1H), 7.35 (d, *J* = 8.1 Hz, 1H), 7.31 (d, *J* = 7.9 Hz, 1H), 7.18 (t, *J* = 7.3 Hz, 1H), 7.10 (t, *J* = 7.5 Hz, 1H), 7.00 (d, *J* = 2.3 Hz, 1H), 4.98 (dt, *J* = 8.1, 5.4 Hz, 1H), 3.71 (s, 3H), 3.40 (d, *J* = 5.4 Hz, 2H), 1.16 (s, 9H) ppm. **^{13}C NMR** (126 MHz, $CDCl_3$): δ = 171.77, 160.41, 158.11, 136.31, 127.64, 122.91, 122.54, 120.04, 118.67, 111.41, 109.86, 58.85, 53.18, 52.67, 27.76, 22.80 ppm. **IR (film): ν_{max}** = 1766, 1652, 1620, 1452, 1254, 1080 cm^{-1}. **HRMS-ESI:** calculated for $C_{18}H_{24}N_3O_4S$ $[M+H]^+$ 378.1842; found 378.1839. **$[\alpha]_D^{25}$**: +12.35 (c 0.253, $CHCl_3$).

Methyl ((*E*)-2-(((*S*)-*tert*-butylsulfinyl)imino)acetyl)-*L*-methioninate (4d):

Imine **4d** was synthesized according to **GP II** and isolated as colourless oil after column chromatography on silica using 28% ethyl acetate in hexane as eluent (212 mg, 33% over two steps). **^{1}H NMR** (400 MHz, $CDCl_3$): δ = 7.93 (s, 1H), 7.53 (d, *J* = 7.7 Hz, 1H), 4.81 – 4.75 (m,

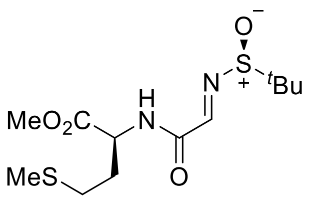

1H), 3.77 (s, 3H), 2.55 – 2.51 (m, 2H), 2.28 – 2.20 (m, 1H), 2.10 – 2.05 (m, 4H), 1.25 (s, 9H) ppm. **^{13}C NMR** (101 MHz, $CDCl_3$): δ = 171.85, 160.50, 158.01, 58.96, 52.87, 51.64, 31.49, 30.02, 22.84, 15.58 ppm. **IR (film): ν_{max}** = 3325, 1734, 1658, 1641, 1324, 1083 cm^{-1}. **HRMS-ESI:** calculated for $C_{12}H_{23}N_2O_4S_2$ $[M+H]^+$ 323.1094; found 323.1089. **$[\alpha]_D^{25}$**: +56.23 (c 0.268, $CHCl_3$).

Methyl ((*E*)-2-(((*S*)-*tert*-butylsulfinyl)imino)acetyl)-*L*-alloisoleucinate (4g):

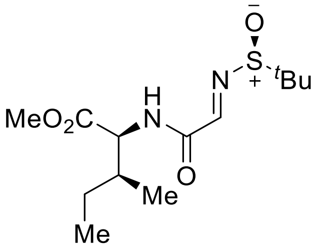

Imine **4g** was synthesized according to **GP II** and isolated as colourless oil after column chromatography on silica using 25% ethyl acetate in hexane as eluent (75 mg, 12% over two steps). **^{1}H NMR** (400 MHz, DMSO-d_6): δ = 9.20 (d, *J* = 7.9 Hz, 1H), 8.08 (s, 1H), 4.33 (t, *J* = 7.1 Hz, 1H), 3.66 (s, 3H), 1.92 – 1.85 (m, 1H), 1.47 – 1.37 (m, 1H), 1.25 – 1.22 (m, 1H), 1.17 (s, 9H), 0.88 – 0.83 (m, 6H) ppm. **^{13}C NMR** (101 MHz, DMSO-d_6): δ = 171.26, 160.42, 158.71, 57.74, 56.87, 51.93, 36.15, 24.87, 22.06, 15.42, 11.14 ppm. **IR (film): ν_{max}** = 2964, 1741, 1680, 1519, 1336, 1092 cm^{-1}. **HRMS-ESI:** calculated for $C_{13}H_{25}N_2O_4S$ $[M+H]^+$ 305.1530; found 305.1515. **$[\alpha]_D^{25}$**: +97.25 (c 0.593, $CHCl_3$).

General Procedure (GP III): The Synthesis of Peptide Based Imines (4):

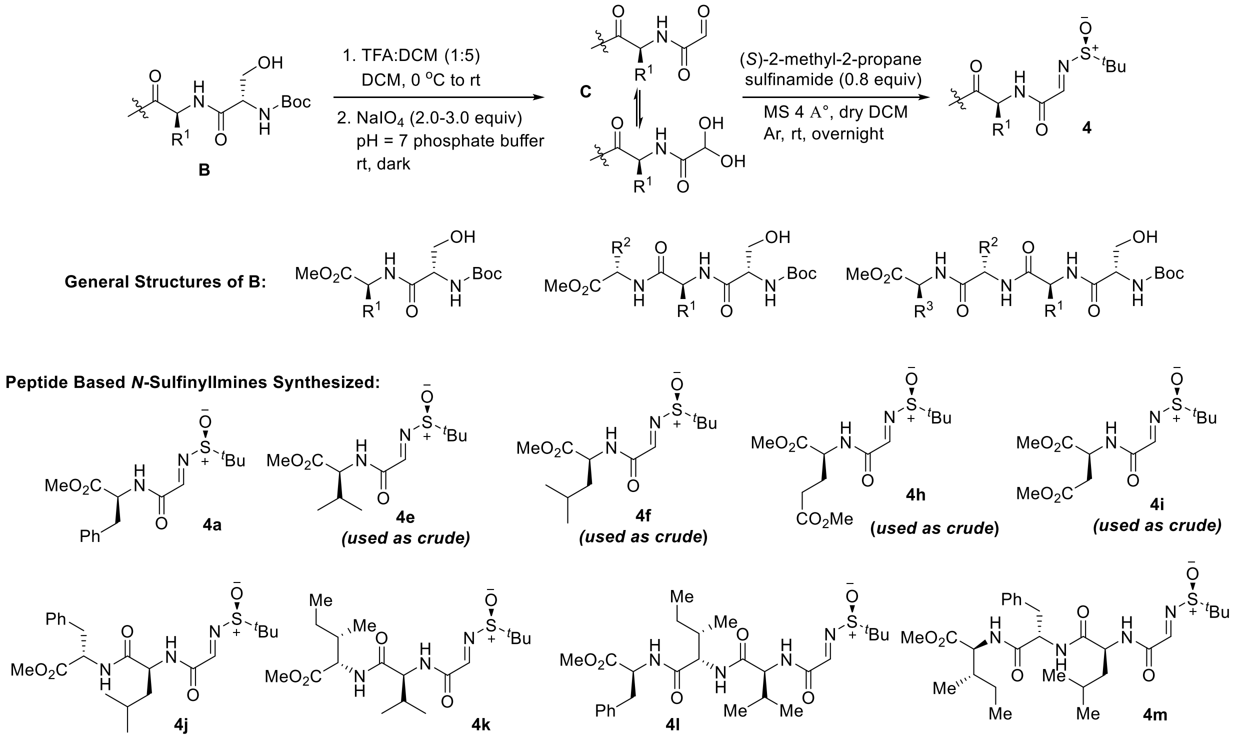

The solution of Boc-protected *N*-terminal serine residue containing oligopeptide **B** (0.3 mmol) in DCM (2.5 mL) was cooled to 0 ^{o}C and TFA (0.25 mL) was added dropwise. After 15 min, the reaction mixture was brought to room temperature and stirred for overnight. After complete consumption of the peptide, TFA and DCM was removed under vacuum. The crude mixture was used for the next step without further purification.

The above crude residue was re-dissolved in 4.0 mL of pH = 7 phosphate buffer solution. To it, $NaIO_4$ (2.0-3.0 equiv) was added at room temperature under dark and stirring was continued for 1 to 4 h. After that the reaction mixture was diluted with water and extracted with ethylacetate thrice. The combined organic layers were dried over anhydrous Na_2SO_4, solvent was removed under reduced pressure and used for next step without further purification.

The above crude mixture was dissolved in dry DCM (0.5 mL) and was added to a solution to (*S*)-2-methyl-2-propanesulfiamide (0.2 mmol, 24 mg) containing 4 Å MS (100 mg) in dry DCM (2 mL) under argon. The reaction was stirred until all the starting material was consumed as indicated by TLC. Then the reaction mixture was diluted by adding DCM, filtered through celite, filtrate was concentrated under vacuo and the crude residue was purified by silica gel column chromatography to provide pure imine **4**.

*Note: for imines **4e, 4f, 4h**, and **4i** which are unstable during the purification, was used as crude after removing the solvent, and re-dissolving the crude residue in degassed chloroform for the final allylation step.*

Methyl ((*E*)-2-(((*S*)-*tert*-butylsulfinyl)imino)acetyl)-*L*-phenylalaninate (4a):

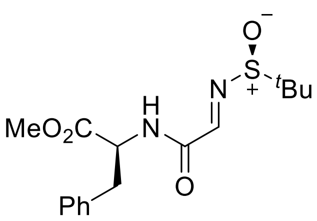

Imine **4a** was synthesized according to **GP III** and isolated as colourless oil after column chromatography on silica using 25% ethylacetate in hexane as eluent (36 mg, 36% over three steps). **^{1}H NMR** (500 MHz, $CDCl_3$): δ = 7.92 (s, 1H), 7.31 – 7.23 (m, 4H), 7.11 (d, *J* = 6.7 Hz, 2H), 4.92 (dt, *J* = 8.3, 5.8 Hz, 1H), 3.75 (s, 3H), 3.23 (dd, *J* = 13.9, 5.7 Hz, 2H), 3.18 (dd, *J* = 13.9, 5.9 Hz, 1H), 1.22 (s, 9H) ppm. **^{13}C NMR** (126 MHz, $CDCl_3$): δ = 171.34, 160.22, 157.97, 135.44, 129.30, 128.79, 127.43, 58.87, 53.21, 52.62, 37.85, 22.77 ppm. **IR (film): ν_{max}** = 3365, 1756, 1685, 1642, 1374, 1074 cm^{-1}. **HRMS-ESI:** calculated for $C_{16}H_{23}N_2O_4S$ $[M+H]^+$ 339.1379; found 339.1384. **$[\alpha]_D^{25}$**: +56.81 (c 0.452, $CHCl_3$).

Methyl ((*E*)-2-(((*S*)-*tert*-butylsulfinyl)imino)acetyl)-*L*-leucyl-*L*-phenylalaninate (4j):

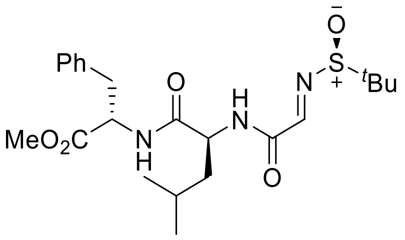

Imine **4j** was synthesized according to **GP III** and isolated as colourless oil after column chromatography on silica using 28% ethylacetate in hexane as eluent (38 mg, 28% over three steps). **^{1}H NMR** (400 MHz, $CDCl_3$): δ = 7.94 (s, 1H), 7.28 – 7.22 (m, 3H), 7.15 (d, *J* = 8.4 Hz, 1H), 7.08 (d, *J* = 7.0 Hz, 2H), 6.44 (d, *J* = 7.8 Hz, 1H), 4.83 (q, *J* = 6.1 Hz, 1H), 4.53 – 4.48 (m, 1H), 3.74 (s, 3H), 3.15 (dd, *J* = 13.9, 5.6 Hz, 1H), 3.08 (dd, *J* = 13.9, 6.3 Hz, 1H), 1.69 – 1.56 (m, 3H), 1.26 (s, 9H), 0.93 (d, *J* = 5.5 Hz, 6H) ppm. **^{13}C NMR** (101 MHz, $CDCl_3$): δ = 171.65, 170.83, 160.47, 158.19, 135.55, 129.34, 128.75, 127.41, 58.93, 53.44, 52.60, 51.66, 41.30, 37.85, 24.86, 22.91, 22.88, 22.25 ppm. **IR (film): ν_{max}** = 3411, 1747, 1655, 1611, 1254, 1085 cm^{-1}. **HRMS-ESI:** calculated for $C_{22}H_{34}N_3O_5S$ $[M+H]^+$ 452.2219; found 452.2231. **$[\alpha]_D^{25}$**: +47.26 (c 0.635, $CHCl_3$).

Methyl ((*E*)-2-(((*S*)-*tert*-butylsulfinyl)imino)acetyl)-*L*-valyl-*L*-alloisoleucinate (4k):

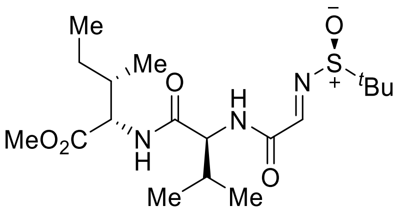

Imine **4k** was synthesized according to **GP III** and isolated as colourless oil after column chromatography on silica using 30% ethylacetate in hexane as eluent (44 mg, 34% over three steps). **^{1}H NMR** (400 MHz, $CDCl_3$): δ = 7.97 (s, 1H), 7.37 (d, *J* = 9.0 Hz, 1H), 6.23 (d, *J* = 8.0 Hz, 1H), 4.58 – 4.55 (m, 1H), 4.36 – 4.33 (m, 1H), 3.75 (s, 3H), 2.23 – 2.16 (m, 1H), 1.95 – 1.85 (m, 1H), 1.44 – 1.39 (m, 2H), 1.26 (s, 9H), 1.02 – 0.99 (m, 6H), 0.93 – 0.88 (m, 6H) ppm. **^{13}C NMR** (101 MHz, $CDCl_3$): δ = 172.05, 170.18, 160.75, 158.25, 58.93, 58.62, 56.75, 52.36, 37.82, 31.50, 25.26, 22.87, 19.25, 18.24, 15.60, 11.69 ppm. **IR (film): ν_{max}** = 3342, 1745, 1702, 1682, 1649, 1064 cm^{-1}. **HRMS-ESI:** calculated for $C_{18}H_{34}N_3O_5S$ $[M+H]^+$ 404.2219; found 404.2225. **$[\alpha]_D^{25}$**: +68.42 (c 0.631, $CHCl_3$).

Methyl ((*E*)-2-(((*S*)-*tert*-butylsulfinyl)imino)acetyl)-*L*-valyl-*L*-alloisoleucyl-*L*-phenylalaninate (4l):

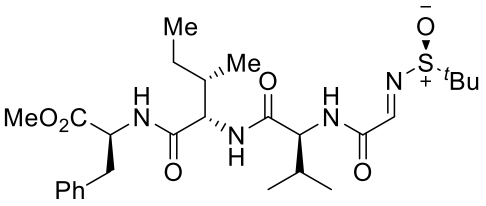

Imine **4l** was synthesized according to **GP III** except 3.0 equiv of $NaIO_4$ was used at the step II and isolated as white solid after column chromatography on silica using 40% ethyl acetate in hexane as eluent (81 mg, 49% over three steps). **^{1}H NMR** (500 MHz, $CDCl_3$): δ = 8.16 (d, *J* = 6.3 Hz, 1H), 8.11 – 8.03 (m, 1H), 7.24 – 7.19 (m, 3H), 7.11 (d, *J* = 7.3 Hz, 2H), 7.03 – 6.92 (m, 2H), 4.87 (q, *J* = 6.6

Hz, 1H), 4.47 – 4.41 (m, 2H), 3.67 (s, 3H), 3.12 – 3.03 (m, 2H), 2.14 – 2.06 (m, 1H), 1.80 – 1.75 (m, 1H), 1.44 – 1.36 (m, 1H), 1.24 (s, 9H), 1.08 – 1.00 (m, 1H), 0.94 (d, *J* = 6.6 Hz, 3H), 0.91 (d, *J* = 6.4 Hz, 3H), 0.85 (d, *J* = 6.6 Hz, 3H), 0.79 (t, *J* = 7.2 Hz, 3H) ppm. **^{13}C NMR** (126 MHz, $CDCl_3$): δ = 171.77, 170.82, 170.58, 160.62, 158.72, 136.01, 129.29, 128.64, 127.18, 58.93, 58.81, 57.55, 53.44, 52.31, 38.03, 37.77, 31.60, 24.89, 22.79, 19.31, 18.46, 15.25, 11.21 ppm. **IR (film): ν_{max}** = 3262, 2964, 1745, 1640, 1548, 1217, 1097 cm^{-1}. **HRMS-ESI:** calculated for $C_{27}H_{43}N_4O_6S$ $[M+H]^+$ 551.2903; found 551.2903. **$[\alpha]_D^{25}$**: +75.14 (c 0.366, $CHCl_3$).

Methyl ((*E*)-2-(((*S*)-*tert*-butylsulfinyl)imino)acetyl)-*L*-leucyl-*L*-phenylalanyl-*L*-isoleucinate (4m):

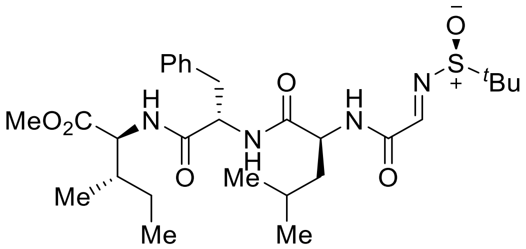

Imine **4m** was synthesized according to **GP III** except 3.0 equiv of $NaIO_4$ was used at the Step II and isolated as colourless oil after column chromatography on silica using 35% ethylacetate in hexane as eluent (158 mg, 28% over three steps). **^{1}H NMR** (500 MHz, $CDCl_3$): δ = 8.13 (s, 1H), 8.01 (br s, 1H), 7.17 – 7.06 (m, 7H), 4.90 (q, *J* = 7.2 Hz, 1H), 4.67 (q, *J* = 7.5, 7.1 Hz, 1H), 4.49 (dd, *J* = 8.4, 5.5 Hz, 1H), 3.68 (s, 3H), 3.03 (dd, *J* = 13.4, 6.6 Hz, 1H), 2.95 (dd, *J* = 13.7, 7.1 Hz, 1H), 1.85 – 1.78 (m, 1H), 1.64 – 1.61 (m, 1H), 1.40 – 1.32 (m, 2H), 1.26 (s, 9H), 1.14 – 1.07 (m, 2H), 0.89 – 0.83 (m, 12H). **^{13}C NMR** (126 MHz, $CDCl_3$): δ = 171.81, 171.23, 170.65, 160.15, 158.87, 136.28, 129.46, 129.42, 128.53, 126.99, 58.89, 56.77, 54.37, 52.09, 51.88, 41.78, 38.84, 37.81, 25.21, 24.87, 22.87, 22.83, 22.39, 15.42, 11.55 ppm. **IR (film): ν_{max}** = 3285, 2925, 1742, 1641, 1536, 1205 cm^{-1}. **HRMS-ESI:** calculated for $C_{28}H_{45}N_4O_6S$ $[M+H]^+$ 565.3060; found 565.3058. **$[\alpha]_D^{25}$**: +64.02 (0.587, $CHCl_3$).

Synthesis of Allylboronic Acids 2:

The following allylboronic acids were synthesized according to the literature reported procedures[1] and extracted in $CHCl_3$ and used immediately for the allylation reaction.

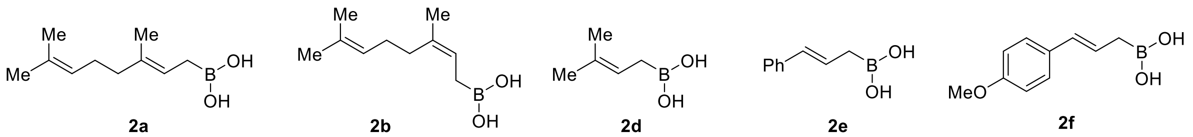

2a **2b** **2d** **2e** **2f**

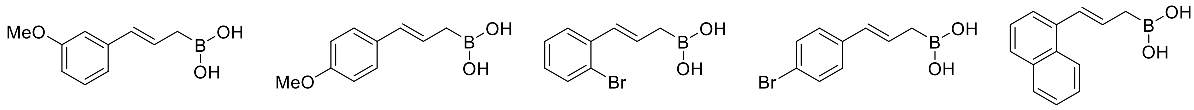

2g **2h** **2j** **2k** **2l**

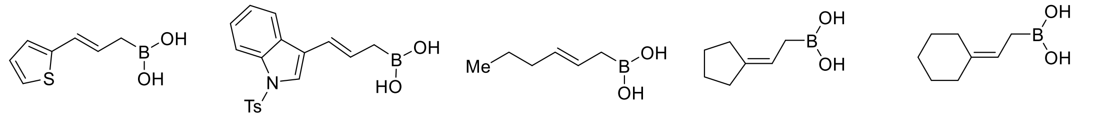

2m **2o** **2p** **2s** **2t**

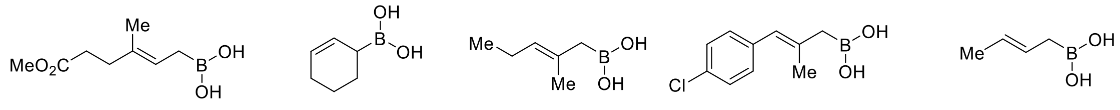

2w **2aa** **2ac** **2ad** **2ae**

General Procedure (GP IV): The Synthesis of Allylboronic Acids

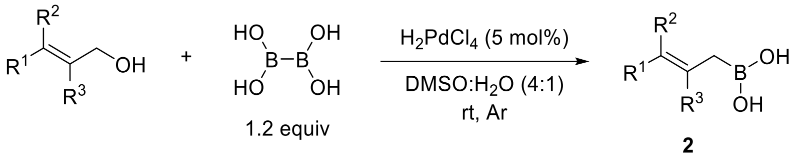

2

A 10 mL two neck round bottom flask was charged with allyl alcohol (2.0 mmol) and the flask was backfilled with nitrogen thrice by using Schlenk technique. Then DMSO (1.6 mL) and H_2O (0.4 mL) were added to it and the reaction mixture was stirred for 2 minutes. Next, freshly prepared H_2PdCl_4 (0.3 M, 0.34 mL, 0.05 mmol) and diboronic acid (216 mg, 2.4 mmol, 1.2 equiv) were added sequentially and the reaction was stirred at room temperature for another 2 to 24 hours. Upon complete consumption of allyl alcohol as evidenced by TLC (*p*-anisaldehyde stain), reaction mixture was allowed to settle down over 2 to 3 hours for the complete sedimentation of Pd. Then the reaction mixture was transferred via syringe to a 25 mL round bottom flask under nitrogen *(Note: For some boronic acids such as* ***2v*** *which are unstable under air, the reaction mixture was filtered through HPLC Teflon filter immediately after the complete conversion of allyl alcohols to remove Pd black).* Then the filtrate containing crude allyl-boronic acid was diluted with 8 mL degassed chloroform and the resulting solution was washed with degassed 16% aq. NaCl solution (performed four times by stirring the mixture, 8 mL x 4, the aqueous layer was discarded every time. To discard the aqueous layer, first the organic layer was taken away under nitrogen atmosphere using a syringe, after that the aqueous layer was discarded with the help of a syringe and finally the organic layer was pushed back to

the same round bottom flask under nitrogen atmosphere for the second washing with NaCl solution). Finally, the organic layer was taken off by a syringe and transferred into another 25 mL round bottom flask containing anhydrous Na_2SO_4 under nitrogen with occasional shaking. The resulting crude allylboronic acid solution was used immediately for allylation of sulfinyl imines.

(**Note:** [a] All the solvents were degassed for 3 hours with 99.999% argon gas under sonication.
[b] For some cases, when allyl alcohols were not completely consumed even after prolonged reaction time, then additional 1 to 2 mol% of H_2PdCl_4 catalyst was added along with 0.2-0.4 equiv of $B_2(OH)_4$. The stirring was continued further until complete consumption of allyl alcohols.)

General Procedure (GP V): The Allylation of Sulfinyl Imines

A 10 mL Schlenk tube was charged with activated 4 Å molecular sieves (100 mg) and a solution of allyl boronic acid in chloroform (0.39 mmol, 1.3 equiv) was added under argon atmosphere. After stirring at room temperature for 2 minutes, imine **1** (0.3 mmol, 1.0 equiv) was added and the resulting mixture was stirred at room temperature for 4 h. After completion of the reaction as indicated by TLC, reaction was quenched by adding 0.5 ml of methanol. The crude mixture was filtered through celite pad and washed with CH_2Cl_2. The combined organic fractions were concentrated and purified on a silica gel column to obtain analytically pure homo-allylic amines **3-6**.

Ethyl *(2S,3R)*-2-(((*S*)-*tert*-butylsulfinyl)amino)-3,7-dimethyl-3-vinyloct-6-enoate (3aa):

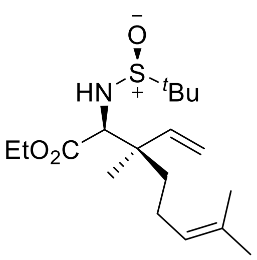

The compound **3aa** was prepared according to **GP V** by using imine **1a** (61 mg, 0.3 mmol, 1.0 equiv), geranyl boronic acid **2a** (0.39 mmol, 1.3 equiv) and 4Å molecular sieves (100 mg) at room temperature. Column chromatography on silica using 12% ethylacetate in hexane as eluent provided the product **3aa** as colourless liquid (73 mg, 71%). **^{1}H NMR** (600 MHz, $CDCl_3$): δ = 5.88 (dd, *J* = 17.6, 10.9 Hz, 1H), 5.51 (d, *J* = 10.8 Hz, 1H), 5.28 (d, *J* = 17.6 Hz, 1H), 5.05 (t, *J* = 6.6 Hz, 1H), 4.21 – 4.16 (m, 3H), 3.49 (d, *J* = 9.8 Hz, 1H), 2.04 – 1.97 (m, 2H), 1.78 – 1.73 (m, 1H), 1.66 (s, 3H), 1.64 – 1.61 (m, 1H), 1.57 (s, 3H), 1.39 (s, 3H), 1.26 (t, *J* = 7.1 Hz, 3H), 0.93 (s, 9H) ppm. **^{13}C NMR** (151 MHz, $CDCl_3$): δ = 172.67, 135.89, 132.36, 123.62, 119.34, 66.59, 65.16, 61.25, 35.39, 34.16, 26.57, 25.73, 22.67, 18.13, 17.76,

14.24. **IR (film):** ν_{max} = 3410, 2967, 1732, 1295, 1213, 1098 cm^{-1}. **HRMS-ESI:** calculated for $C_{18}H_{33}NNaO_3S$ [M+Na]$^+$ 366.2073; found 366.2054. **[α]$_D$^{25}**: +70.57 (c 0.66, $CHCl_3$).

Ethyl (*2S,3S*)-2-(((*S*)-*tert*-butylsulfinyl)amino)-3,7-dimethyl-3-vinyloct-6-enoate (3ab):

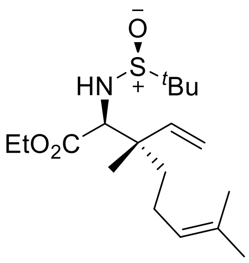

The compound **3ab** was prepared according to **GP V** by using imine **1a** (61 mg, 0.3 mmol, 1.0 equiv), neryl boronic acid **2b** (0.39 mmol, 1.3 equiv) and 4Å molecular sieves (100 mg) at room temperature. Column chromatography on silica using 15% ethylacetate in hexane as eluent provided the product **3ab** as colourless liquid (75 mg, 73%). **^{1}H NM**R (500 MHz, $CDCl_3$): δ = 5.99 (dd, *J* = 17.6, 10.8 Hz, 1H), 5.59 (d, *J* = 10.8 Hz, 1H), 5.30 (d, *J* = 17.6 Hz, 1H), 5.08 (t, *J* = 6.7 Hz, 1H), 4.32 (d, *J* = 9.8 Hz, 1H), 4.20 – 4.15 (m, 2H), 3.51 (d, *J* = 9.9 Hz, 1H), 2.09 – 2.01 (m, 1H), 1.98 – 1.91 (m, 1H), 1.83 (td, *J* = 12.7, 12.2, 4.7 Hz, 1H), 1.74 (td, *J* = 12.4, 5.4 Hz, 1H), 1.65 (s, 3H), 1.56 (s, 3H), 1.28 – 1.24 (m, 6H), 0.92 (s, 9H) ppm. **^{13}C NMR** (126 MHz, $CDCl_3$): δ = 172.68, 135.35, 132.41, 123.50, 119.90, 66.25, 65.43, 61.24, 35.97, 35.40, 26.55, 25.70, 22.57, 17.73, 16.69, 14.23 ppm. **IR (film):** ν_{max} = 2966, 1733, 1296, 1214, 1158, 1095 cm^{-1}. **HRMS-ESI:** calculated for $C_{18}H_{33}NNaO_3S$ [M+Na]$^+$ 366.2073; found 366.2093. **[α]$_D$^{25}**: +68.43 (c 0.833, $CHCl_3$).

Ethyl (*2S,3R*)-2-(((*S*)-*tert*-butylsulfinyl)amino)-3,7,11-trimethyl-3-vinyldodeca-6,10-dienoate (3ac):

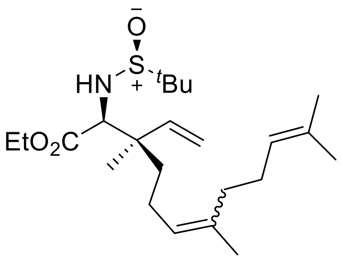

The compound **3ac** was prepared according to **GP V** by using imine **1a** (61 mg, 0.3 mmol, 1.0 equiv), farnesyl boronic acid **2c** (0.39 mmol, 1.3 equiv) and 4Å molecular sieves (100 mg) at room temperature. Column chromatography on silica using 10% ethylacetate in hexane provided the product **3ac** as a colourless oil (95 mg, 77%) (mixture of *E* and *Z* isomer originated from the commercially available farnesol). **^{1}H NMR** (600 MHz, $CDCl_3$): δ = 5.92 – 5.86 (m, 2H), 5.54 (d, *J* = 10.8 Hz, 2H), 5.31 (d, *J* = 17.6 Hz, 2H), 5.08 (t, *J* = 8.8 Hz, 2H), 4.23 – 4.19 (m, 6H), 3.51 (d, *J* = 9.8 Hz, 2H), 2.06 – 2.01 (m, 10H), 1.99 – 1.94 (m, 4H), 1.80 – 1.74 (m, 4H), 1.68 (s, 6H), 1.67 (s, 6H), 1.58 (s, 6H), 1.41 (s, 6H), 1.27 (t, *J* = 7.1 Hz, 6H), 0.94 (s, 18H) ppm. **^{13}C NMR** (151 MHz, $CDCl_3$): δ = 172.75, 136.22, 136.04, 135.80, 135.71, 131.87, 131.57, 124.30, 124.27, 124.23, 123.50, 119.58, 119.51, 66.56, 66.54, 65.18, 65.14, 61.32, 39.76, 35.44, 34.33, 34.12, 32.10, 26.72, 26.63, 26.59, 25.89, 25.83, 23.49, 22.58, 22.46, 18.26, 18.21, 17.82, 17.78, 16.15, 14.29 ppm.

IR (film): ν_{max} = 2965, 1734, 1216, 1159, 1099, 1022 cm^{-1}. **HRMS-ESI:** calculated for $C_{23}H_{42}NO_3S$ [M+H]$^+$ 412.2880; found 412.2883.

Ethyl (*S*)-2-(((*S*)-*tert*-butylsulfinyl)amino)-3,3-dimethylpent-4-enoate (3ad):

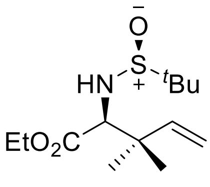

The compound **3ad** was prepared according to **GP V** by using imine **1a** (61 mg, 0.3 mmol, 1.0 equiv), prenyl boronic acid **2d** (0.39 mmol, 1.3 equiv) and 4Å molecular sieves (100 mg) at room temperature. Column chromatography on silica using 10% ethylacetate in hexane as eluent provided the product **3d** as colourless liquid (54 mg, 65%). **^{1}H NMR** (600 MHz, $CDCl_3$): δ = 6.03 (dd, *J* = 17.5, 10.7 Hz, 1H), 5.52 (d, *J* = 10.8 Hz, 1H), 5.31 (d, *J* = 17.5 Hz, 1H), 4.25 (d, *J* = 9.7 Hz, 1H), 4.21 – 4.17 (m, 2H), 3.51 (d, *J* = 9.8 Hz, 1H), 1.41 (s, 3H), 1.31 (s, 3H), 1.27 (t, *J* = 7.1 Hz, 3H), 0.94 (s, 9H) ppm. **^{13}C NMR** (151 MHz, $CDCl_3$): δ = 172.74, 136.42, 118.96, 66.30, 61.73, 61.32, 35.43, 26.54, 22.90, 20.51, 14.28 ppm. **IR (film):** ν_{max} = 2964, 1732, 1215, 1158, 1100, 1022 cm^{-1}. **HRMS-ESI:** calculated for $C_{13}H_{26}NO_3S$ [M+H]$^+$ 276.1628; found 276.1626. **[α]$_D^{25}$**: +169.0 (c 0.5, $CHCl_3$)

Ethyl (*2S,3S*)-2-(((*S*)-*tert*-butylsulfinyl)amino)-3-phenylpent-4-enoate (3ae):[2]

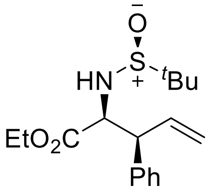

The compound **3ae** was prepared according to **GP V** by using imine **1a** (61 mg, 0.3 mmol, 1.0 equiv), cinnamyl boronic acid **2e** (0.39 mmol, 1.3 equiv) and 4Å molecular sieves (100 mg) at room temperature. Column chromatography on silica using 14% ethylacetate in hexane as eluent provided the product **3ae** as colourless liquid (66 mg, 68%). **^{1}H NMR** (600 MHz, $CDCl_3$): δ = 7.29 (t, *J* = 7.6 Hz, 2H), 7.21 (t, *J* = 7.4 Hz, 1H), 7.15 (d, *J* = 6.8 Hz, 2H), 6.08 – 6.02 (m, 1H), 5.16 – 5.13 (m, 2H), 4.20 – 4.11 (m, 3H), 3.90 (d, *J* = 9.5 Hz, 1H), 3.67 (t, *J* = 8.0 Hz, 1H), 1.25 (t, *J* = 7.2 Hz, 3H), 1.05 (s, 9H) ppm. **^{13}C NMR** (151 MHz, $CDCl_3$): δ = 172.36, 139.35, 136.99, 128.68, 128.46, 127.29, 117.67, 62.96, 61.74, 56.41, 54.24, 22.57, 14.22 ppm. **IR (film):** ν_{max} = 2981, 1732, 1455, 1200, 1178, 1091 cm^{-1}. **HRMS-ESI:** calculated for $C_{17}H_{26}NO_3S$ [M+H]$^+$ 324.1628; found 324.1272. **[α]$_D^{20}$**: + 79.8 (c 0.68, $CHCl_3$).

Ethyl (*2S,3S*)-2-(((*S*)-*tert*-butylsulfinyl)amino)-3-(2-methoxyphenyl)pent-4-enoate (3af):

The compound **3af** was prepared according to **GP V** by using imine **1a** (61 mg, 0.3 mmol, 1.0 equiv), **2f** (0.39 mmol, 1.3 equiv) and 4Å molecular sieves (100 mg) at room temperature. Column chromatography on silica using 17% ethylacetate in hexane as eluent provided the

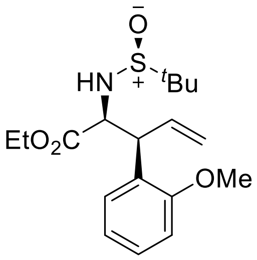

product **3af** as a colourless oil (79 mg, 73%). **^{1}H NMR** (600 MHz, $CDCl_3$): δ = 7.19 (t, *J* = 7.8 Hz, 1H), 7.12 (d, *J* = 7.5 Hz, 1H), 6.89 (t, *J* = 7.5 Hz, 1H), 6.83 (d, *J* = 8.2 Hz, 1H), 6.11 (dt, *J* = 17.2, 9.5 Hz, 1H), 5.14 (d, *J* = 17.1 Hz, 1H), 5.11 (d, *J* = 10.2 Hz, 1H), 4.26 (t, *J* = 9.3 Hz, 1H), 4.20 – 4.08 (m, 2H), 4.02 (t, *J* = 8.7 Hz, 1H), 3.90 (d, *J* = 10.0 Hz, 1H), 3.82 (s, 3H), 1.24 (t, *J* = 7.1 Hz, 3H), 0.98 (s, 9H) ppm. **^{13}C NMR** (151 MHz, $CDCl_3$): δ = 173.02, 157.26, 136.23, 129.40, 128.23, 128.05, 120.73, 117.71, 110.78, 61.71, 61.42, 56.15, 55.46, 48.59, 22.46, 14.21 ppm. **IR (film): ν_{max}** = 2967, 1732, 1493, 1366, 1245, 1096 cm^{-1}. **HRMS-ESI:** calculated for $C_{18}H_{28}NO_4S$ $[M+H]^+$ 354.1734; found 354.1742. **$[\alpha]_D^{25}$**: +73.80 (c 0.166, $CHCl_3$).

Ethyl (*2S,3S*)-2-(((*S*)-*tert*-butylsulfinyl)amino)-3-(3-methoxyphenyl)pent-4-enoate (3ag):

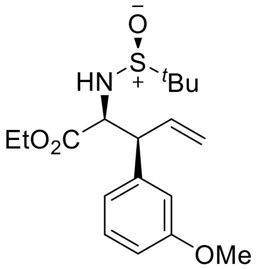

The compound **3ag** was prepared according to **GP V** by using imine **1a** (61 mg, 0.3 mmol, 1.0 equiv), **2g** (0.39 mmol, 1.3 equiv) and 4Å molecular sieves (150 mg) at room temperature. Column chromatography on silica using 17% ethylacetate in hexane as eluent provided the product **3ag** as colourless oil (74 mg, 70%). **^{1}H NMR** (600 MHz, $CDCl_3$): δ = 7.21 (t, *J* = 7.9 Hz, 1H), 6.75 (t, *J* = 6.6 Hz, 2H), 6.70 (s, 1H), 6.03 (ddd, *J* = 16.9, 10.2, 8.4 Hz, 1H), 5.16 (d, *J* = 11.1 Hz, 1H), 5.13 (d, *J* = 4.0 Hz, 1H), 4.20 – 4.13 (m, 3H), 3.92 (d, *J* = 9.5 Hz, 1H), 3.77 (s, 3H), 3.65 (t, *J* = 8.0 Hz, 1H), 1.26 (t, *J* = 7.2 Hz, 3H), 1.07 (s, 9H) ppm. **^{13}C NMR** (151 MHz, $CDCl_3$): δ = 172.33, 159.77, 140.82, 136.81, 129.69, 120.75, 117.76, 114.32, 112.38, 62.90, 61.79, 56.44, 55.31, 54.20, 22.59, 14.26 ppm. **IR (film): ν_{max}** = 2954, 1732, 1600, 1456, 1261, 1064 cm^{-1}. **HRMS-ESI:** calculated for $C_{18}H_{28}NO_4S$ $[M+H]^+$ 354.1734; found 354.1747. **$[\alpha]_D^{25}$**: +114.66 (c 0.133, $CHCl_3$).

Ethyl (*2S,3S*)-2-(((*S*)-*tert*-butylsulfinyl)amino)-3-(4-methoxyphenyl)pent-4-enoate (3ah):

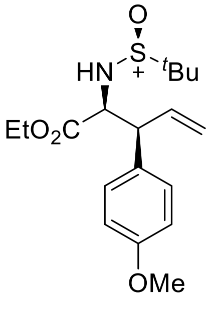

The compound **3ah** was prepared according to **GP V** by using imine **1a** (61 mg, 0.3 mmol, 1.0 equiv), **2h** (0.39 mmol, 1.3 equiv) and 4Å molecular sieves (100 mg) at room temperature. Column chromatography on silica using 17% ethylacetate in hexane as eluent provided the product **3ah** as a colourless oil (76 mg, 72%). **^{1}H NMR** (600 MHz, $CDCl_3$): δ = 7.12 (d, *J* = 9.0 Hz, 2H), 6.83 (d, *J* = 8.4 Hz, 2H), 6.09 – 6.02 (m, 1H), 5.18 (d, *J* = 10.8 Hz, 1H), 5.09 (d, *J* = 17.2 Hz, 1H), 4.13 – 4.12 (m, 2H), 4.05 (q, *J* = 7.1 Hz, 2H), 3.78 (s, 3H), 3.67 – 3.66 (m, 1H), 1.18 (s, 9H), 1.10 (t, *J* = 7.2 Hz, 3H) ppm. **^{13}C NMR** (151 MHz, $CDCl_3$):

δ = 172.43, 158.79, 136.37, 131.48, 129.53, 118.15, 113.94, 62.92, 61.73, 56.50, 55.40, 52.88, 22.82, 14.06 ppm. **IR (film):** ν_{max} = 2957, 1732, 1613, 1471, 1325, 1082 cm^{-1} cm^{-1}. **HRMS-ESI:** calculated for $C_{18}H_{28}NO_4S$ $[M+H]^+$ 354.1734; found 354.1737. $[\alpha]_D^{25}$: +55.87 (c 0.649, $CHCl_3$).

Ethyl (2*S*,3*S*)-2-(((*S*)-*tert*-butylsulfinyl)amino)-3-(2-((3-methylbut-2-en-1-yl)oxy)phenyl) pent-4-enoate (3ai):

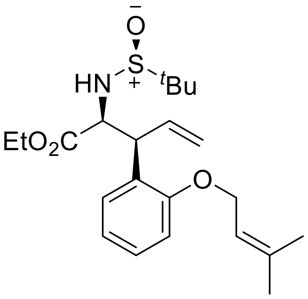

The compound **3ai** was prepared according to **GP V** by using imine **1a** (61 mg, 0.3 mmol, 1.0 equiv), **2k** (0.39 mmol, 1.3 equiv) and 4Å molecular sieves (100 mg) at room temperature. Column chromatography on silica (Et_3N neutralized) using 12% ethylacetate in hexane as eluent provided the product **3ai** as colourless oil (75 mg, 62%). **^{1}H NMR** (500 MHz, $CDCl_3$): δ = 7.16 (t, *J* = 7.8 Hz, 1H), 7.11 (d, *J* = 7.5 Hz, 1H), 6.88 (t, *J* = 7.4 Hz, 1H), 6.84 (d, *J* = 8.2 Hz, 1H), 6.12 (dt, *J* = 18.5, 9.5 Hz, 1H), 5.50 (t, *J* = 5.7 Hz, 1H), 5.16 – 5.09 (m, 2H), 4.57 – 4.49 (m, 2H), 4.28 (t, *J* = 9.4 Hz, 1H), 4.19 – 4.12 (m, 2H), 4.01 (t, *J* = 8.8 Hz, 1H), 3.92 (d, *J* = 10.1 Hz, 1H), 1.80 (s, 3H), 1.75 (s, 3H), 1.24 (t, *J* = 7.4 Hz, 3H), 0.98 (s, 9H) ppm. **^{13}C NMR** (101 MHz, $CDCl_3$): δ = 173.07, 156.60, 137.56, 136.21, 129.35, 128.39, 128.09, 120.61, 120.06, 117.73, 112.04, 65.27, 61.73, 61.37, 56.14, 48.84, 25.89, 22.45, 18.43, 14.19 ppm. **IR (film):** ν_{max} = 2979, 1733, 1491, 1453, 1260, 1079 cm^{-1}. **HRMS-ESI:** calculated for $C_{22}H_{24}NO_4S$ $[M+H]^+$ 408.2203; found 408.2212. $[\alpha]_D^{25}$: +194.2 (c 0.68, $CHCl_3$).

Ethyl (2*S*,3*S*)-3-(2-bromophenyl)-2-(((*S*)-*tert*-butylsulfinyl)amino)pent-4-enoate (3aj):

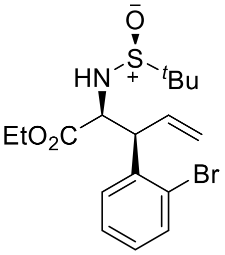

The compound **3aj** was prepared according to **GP V** by using imine **1a** (61 mg, 0.3 mmol, 1.0 equiv), **2j** (0.45 mmol, 1.5 equiv) and 4Å molecular sieves (100 mg) at room temperature. Column chromatography on silica using 15% ethylacetate in hexane as eluent provided the product **3aj** as colourless oil (101 mg, 84%). **^{1}H NMR** (400 MHz, $CDCl_3$): δ = 7.50 (d, *J* = 7.9 Hz, 1H), 7.24 – 7.12 (m, 2H), 7.05 – 7.00 (m, 1H), 5.93 (ddd, *J* = 17.1, 10.1, 8.6 Hz, 1H), 5.15 (d, *J* = 13.4 Hz, 1H), 5.12 (d, *J* = 6.4 Hz, 1H), 4.24 (t, *J* = 8.7 Hz, 1H), 4.19 – 4.11 (m, 2H), 4.07 (t, *J* = 9.7 Hz, 1H), 3.93 (d, *J* = 10.1 Hz, 1H), 1.21 (t, *J* = 6.3 Hz, 3H), 0.95 (s, 9H) ppm. **^{13}C NMR** (101 MHz, $CDCl_3$): δ = 172.26, 139.24, 135.34, 133.14, 129.02, 128.49, 127.75, 125.63, 118.80, 63.13, 61.72, 56.24, 52.30, 22.35, 14.14 ppm. **IR (film):** ν_{max} = 2980,

1733, 1471, 1305, 1181, 1080 cm^{-1}. **HRMS-ESI:** calculated for $C_{17}H_{25}BrNO_3S$ $[M+H]^+$ 404.2497; found 404.2492. **[α]$_D$^{25}**: +123.0 (c 0.5, $CHCl_3$).

Ethyl (2*S*,3*S*)-3-(4-bromophenyl)-2-(((*S*)-*tert*-butylsulfinyl)amino)pent-4-enoate (3ak):[2]

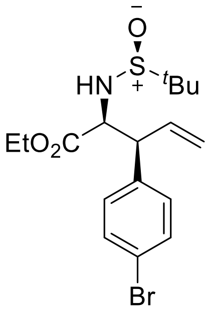

The compound **3ak** was prepared according to **GP V** by using imine **1a** (61 mg, 0.3 mmol, 1.0 equiv), **2k** (0.39 mmol, 1.3 equiv) and 4Å molecular sieves (100 mg) at room temperature. Column chromatography on silica using 15% ethylacetate in hexane as eluent provided the product **3ak** as colourless oil (102 mg, 85%). **^{1}H NMR** (500 MHz, $CDCl_3$): δ = 7.41 (d, *J* = 8.4 Hz, 2H), 7.03 (d, *J* = 8.4 Hz, 2H), 5.99 (ddd, *J* = 16.9, 10.3, 8.2 Hz, 1H), 5.16 – 5.10 (m, 2H), 4.19 – 4.11 (m, 3H), 3.92 (d, *J* = 9.2 Hz, 1H), 3.65 (t, *J* = 7.8 Hz, 1H), 1.24 (t, *J* = 7.2 Hz, 3H), 1.07 (s, 9H) ppm. **^{13}C NMR** (126 MHz, $CDCl_3$): δ = 172.03, 138.43, 136.45, 131.73, 130.21, 121.16, 118.11, 62.55, 61.86, 56.47, 53.49, 22.59, 14.20 ppm. **IR (film): ν$_{max}$** = 3278, 2968, 1727, 1489, 1178, 1077 cm^{-1}. **HRMS-ESI:** calculated for $C_{17}H_{24}BrNO_3SNa$ $[M+Na]^+$ 424.0558; found 424.0559. **[α]$_D$^{25}**: + 68.0 (c 0.99, $CHCl_3$)

Ethyl (*2S,3S*)-2-(((*S*)-*tert*-butylsulfinyl)amino)-3-(naphthalen-1-yl)pent-4-enoate (3al):

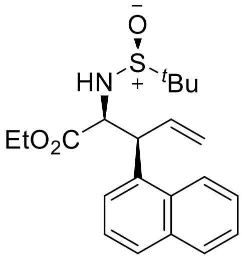

The compound **3al** was prepared according to **GP V** by using imine **1a** (61 mg, 0.3 mmol, 1.0 equiv), **2l** (0.39 mmol, 1.3 equiv) and 4Å molecular sieves (150 mg) at room temperature. Column chromatography on silica using 10% ethyl acetate in hexane as eluent provided the product **3al** as colourless oil (92 mg, 82%). **^{1}H NMR** (600 MHz, $CDCl_3$): δ = 8.11 (d, *J* = 8.6 Hz, 1H), 7.85 (d, *J* = 8.1 Hz, 1H), 7.74 (d, *J* = 7.7 Hz, 1H), 7.54 (t, *J* = 7.2 Hz, 1H), 7.48 (t, *J* = 7.2 Hz, 1H), 7.46 – 7.41 (m, 2H), 6.18 (ddd, *J* = 17.0, 10.2, 8.3 Hz, 1H), 5.23 (d, *J* = 17.0 Hz, 1H), 5.19 (d, *J* = 10.2 Hz, 1H), 4.57 (t, *J* = 8.3 Hz, 1H), 4.34 (t, *J* = 9.0 Hz, 1H), 4.16 – 4.08 (m, 2H), 3.94 (d, *J* = 9.7 Hz, 1H), 1.19 (t, *J* = 7.2 Hz, 3H), 0.86 (s, 9H) ppm. **^{13}C NMR** (151 MHz, $CDCl_3$): δ = 172.66, 136.70, 135.76, 134.14, 132.20, 129.19, 127.75, 126.35, 125.69, 125.37, 125.33, 122.85, 118.17, 62.95, 61.71, 56.27, 48.34, 26.47, 22.35, 14.10 ppm. **IR (film): ν$_{max}$** = 2954, 1738, 1452, 1362, 1176, 1085 cm^{-1}. **HRMS-ESI:** calculated for $C_{21}H_{28}NO_3S$ $[M+H]^+$ 374.1784; found 374.1793. **[α]$_D$^{25}**: +45.23 (c 0.523, $CHCl_3$).

Ethyl (*2S,3R*)-2-(((*S*)-*tert*-butylsulfinyl)amino)-3-(thiophen-2-yl)pent-4-enoate (3am):
The compound **3am** was prepared according to **GP V** by using imine **1a** (61 mg, 0.3 mmol, 1.0 equiv), **2m** (0.39 mmol, 1.3 equiv) and 4Å molecular sieves (150 mg) at room temperature.

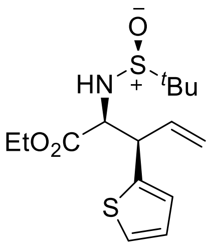

Column chromatography on silica using 25% ethylacetate in hexane as eluent provided the product **3am** as colourless oil (60 mg, 61%). **^{1}H NMR** (600 MHz, $CDCl_3$): δ = 7.19 (d, *J* = 5.1 Hz, 1H), 6.93 (t, *J* = 4.2 Hz, 1H), 6.81 (d, *J* = 3.3 Hz, 1H), 6.06 – 6.00 (m, 1H), 5.20 – 5.18 (m, 2H), 4.22 – 4.15 (m, 5H), 1.25 – 1.22 (m, 12H) ppm. **^{13}C NMR** (151 MHz, $CDCl_3$): δ = 171.44, 140.87, 137.31, 126.66, 125.78, 124.98, 117.92, 62.76, 62.04, 56.75, 49.24, 22.82, 14.24 ppm. **IR (film): ν_{max}** = 2981, 1734, 1366, 1283, 1183, 1082 cm^{-1}. **HRMS-ESI:** calculated for $C_{15}H_{24}NO_3S_2$ $[M+H]^+$ 330.1192; found 330.1177. **$[\alpha]_D^{25}$**: +205.8 (c 0.52, $CHCl_3$).

Ethyl (*2S,3R*)-2-(((*S*)-*tert*-butylsulfinyl)amino)-3-(furan-2-yl)pent-4-enoate (3an):

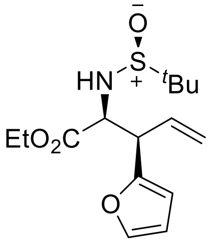

The compound **3an** was prepared according to **GP V** by using imine **1a** (61 mg, 0.3 mmol, 1.0 equiv), **2n** (0.39 mmol, 1.3 equiv) and 4Å molecular sieves (100 mg) at room temperature. Column chromatography on silica using 25% ethylacetate in hexane as eluent provided the product **3an** as colourless oil (60 mg, 64%). **^{1}H NMR** (600 MHz, $CDCl_3$): δ = 7.32 (s, 1H), 6.28 (d, *J* = 5.0 Hz, 1H), 6.07 (d, *J* = 3.2 Hz, 2H), 5.95 (ddd, *J* = 17.1, 10.2, 8.5 Hz, 1H), 5.21 (d, *J* = 5.9 Hz, 1H), 5.19 (d, *J* = 12.8 Hz, 1H), 4.20 – 4.15 (m, 3H), 4.07 (d, *J* = 9.1 Hz, 1H), 3.87 (t, *J* = 7.8 Hz, 1H), 1.23 (t, *J* = 7.2 Hz, 3H), 1.16 (s, 9H) ppm. **^{13}C NMR** (151 MHz, $CDCl_3$): δ = 171.75, 152.51, 141.95, 134.20, 118.83, 110.35, 107.45, 61.86, 61.42, 56.51, 47.92, 22.71, 14.16 ppm. **IR (film): ν_{max}** = 2982, 1734, 1367, 1175, 1094, 1011 cm^{-1}. **HRMS-ESI:** calculated for $C_{15}H_{24}NO_4S$ $[M+H]^+$ 314.1421; found 314.1408. **$[\alpha]_D^{25}$**: +35.02 (c 0.741, $CHCl_3$).

Ethyl (*2S,3S*)-2-(((*S*)-*tert*-butylsulfinyl)amino)-3-(1-tosyl-1*H*-indol-3-yl)pent-4-enoate (3ao):

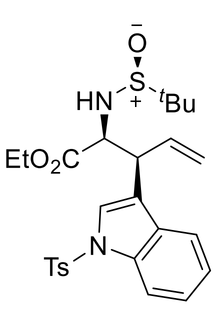

The compound **3ao** was prepared according to **GP V** by using imine **1a** (61 mg, 0.3 mmol, 1.0 equiv), **2o** (0.39 mmol, 1.3 equiv) and 4Å molecular sieves (100 mg) at room temperature. Column chromatography on silica using 18% ethylacetate in hexane as eluent provided the product **3ao** as gummy solid (113 mg, 73%). **^{1}H NMR** (600 MHz, $CDCl_3$): δ = 7.95 (d, *J* = 8.3 Hz, 1H), 7.72 (d, *J* = 8.4 Hz, 2H), 7.48 (d, *J* = 7.9 Hz, 1H), 7.42 (s, 1H), 7.30 (t, *J* = 7.4 Hz, 1H), 7.23 – 7.21 (m, 3H), 6.03 (ddd, *J* = 17.0, 10.2, 7.8 Hz, 1H), 5.19 (d, *J* = 10.2 Hz, 1H), 5.16 (d, *J* = 17.0 Hz, 1H), 4.27 (dd, *J* = 8.4, 6.3 Hz, 1H), 4.11 (dd, *J* = 7.8, 5.0 Hz, 1H), 4.08 – 3.96 (m, 3H), 2.33 (s, 3H), 1.09 – 1.06 (m, 12H) ppm. **^{13}C NMR** (151 MHz,

$CDCl_3$): δ = 171.83, 145.10, 135.61, 135.38, 135.06, 130.23, 130.03, 126.88, 125.06, 124.04, 123.25, 120.17, 119.70, 118.35, 113.85, 61.93, 61.51, 56.47, 44.91, 22.60, 21.67, 13.99 ppm. **IR (film):** ν_{max} = 2980, 1732, 1447, 1366, 1173, 1071 cm^{-1}. **HRMS-ESI:** calculated for $C_{26}H_{32}N_2NaO_5S_2$ $[M+Na]^+$ 539.1645; found 539.1634. **[α]D^20**: +175.4 (c 0.48, $CHCl_3$).

Ethyl (*2S,3R*)-2-(((*S*)-*tert*-butylsulfinyl)amino)-3-vinylhexanoate (3ap):

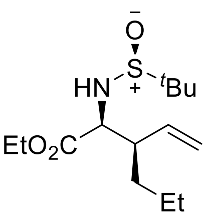

The compound **3ap** was prepared according to **GP V** by using imine **1a** (61 mg, 0.3 mmol, 1.0 equiv), **2p** (0.90 mmol, 3.0 equiv) and 4Å molecular sieves (100 mg) at 60 °C. Column chromatography on silica using 10% ethylacetate in hexane as eluent provided the product **3ap** as colourless oil (55 mg, 64%). **^{1}H NMR** (600 MHz, $CDCl_3$): δ = 5.60 – 5.54 (m, 1H), 5.10 (d, *J* = 10.2 Hz, 1H), 5.07 (d, *J* = 17.3 Hz, 1H), 4.25 – 4.15 (m, 2H), 4.08 (d, *J* = 8.3 Hz, 1H), 3.81 (t, *J* = 7.2 Hz, 1H), 2.40 – 2.32 (m, 1H), 1.48 – 1.43 (m, 1H), 1.40 – 1.31 (m, 2H), 1.31 – 1.26 (m, 4H), 1.25 (s, 9H), 0.87 (t, *J* = 6.9 Hz, 3H) ppm. **^{13}C NMR** (151 MHz, $CDCl_3$): δ = 172.64, 137.70, 117.88, 61.83, 61.60, 56.44, 48.41, 32.32, 22.90, 20.15, 14.31, 14.03 ppm. **IR (film):** ν_{max} = 2959, 1733, 1466, 1366, 1184, 1075, 917 cm^{-1}. **HRMS-ESI:** calculated for $C_{14}H_{27}NNaO_3S$ $[M+H]^+$ 312.1604; found 312.1589. **[α]D^25**: +63.91, (c 0.133, $CHCl_3$).

Ethyl (*2S,3R*)-2-(((*S*)-*tert*-butylsulfinyl)amino)-5-methyl-3-vinylhexanoate (3aq):

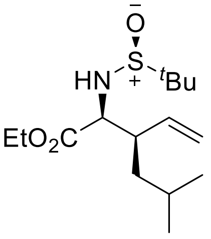

The compound **3aq** was prepared according to **GP V** by using imine **1a** (61 mg, 0.3 mmol, 1.0 equiv), **2q** (0.90 mmol, 3.0 equiv) and 4Å molecular sieves (100 mg) at 60 °C. Column chromatography on silica using 14% ethylacetate in hexane as eluent provided the major diastereomer **3aq** as a colourless oil (61 mg, 67%, *dr*: 4:1). **^{1}H NMR** (600 MHz, $CDCl_3$): δ = 5.56 (dt, *J* = 17.1, 9.8 Hz, 1H), 5.10 (d, *J* = 6.8 Hz, 1H), 5.08 (d, *J* = 15.3 Hz, 1H), 4.25 – 4.16 (m, 2H), 4.10 (d, *J* = 8.5 Hz, 1H), 3.80 (dd, *J* = 8.5, 5.6 Hz, 1H), 2.48 – 2.43 (m, 1H), 1.69 – 1.63 (m, 1H), 1.61 – 1.52 (m, 1H), 1.28 (t, *J* = 7.1 Hz, 3H), 1.25 (s, 9H), 1.21 – 1.17 (m, 1H), 0.89 (d, *J* = 6.6 Hz, 3H), 0.82 (d, *J* = 6.5 Hz, 3H) ppm. **^{13}C NMR** (151 MHz, $CDCl_3$): δ = 172.61, 137.92, 117.74, 62.09, 61.64, 56.47, 46.58, 39.23, 29.84, 25.17, 23.88, 22.92, 21.43, 14.34 ppm. **IR (film):** ν_{max} = 2926, 1734, 1467, 1366, 1184, 1083 cm^{-1}. **HRMS-ESI:** calculated for $C_{15}H_{19}NNaO_3S$ $[M+Na]^+$ 326.1760, found 326.1748. **[α]D^25**: +50.00 (c 0.100, $CHCl_3$).

Ethyl (*2S,3R*)-2-(((*S*)-*tert*-butylsulfinyl)amino)-6-methyl-3-vinylhept-5-enoate (3ar):

The compound **3ar** was prepared according to **GP V** by using imine **1a** (41 mg, 0.2 mmol, 1.0 equiv), **2r** (0.26 mmol, 1.3 equiv) and 4Å molecular sieves (100 mg) at room temperature.

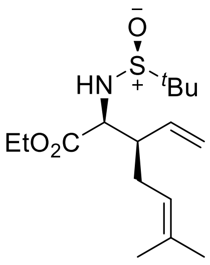

Column chromatography on silica using 12% ethylacetate in hexane as eluent provided the product **3ar** as colourless oil (49 mg, 78%). **^{1}H NMR** (400 MHz, $CDCl_3$): δ = 5.61 (dt, *J* = 16.8, 9.5 Hz, 1H), 5.09 – 5.03 (m, 3H), 4.18 (q, *J* = 7.1 Hz, 2H), 4.12 (d, *J* = 8.2 Hz, 1H), 3.85 (t, *J* = 10.2 Hz, 1H), 2.45 – 2.36 (m, 1H), 2.27 – 2.21 (m, 1H), 2.06 – 1.99 (m, 1H), 1.67 (s, 3H), 1.57 (s, 3H), 1.29 – 1.19 (m, 12H) ppm. **^{13}C NMR** (101 MHz, $CDCl_3$): δ = 172.57, 137.48, 133.67, 121.16, 117.58, 61.63, 61.07, 56.38, 48.80, 28.93, 25.89, 22.86, 18.06, 14.28 ppm. **IR (film): ν_{max}** = 2979, 1733, 1366, 1271, 1191, 1085 cm^{-1}. **HRMS-ESI:** calculated for $C_{16}H_{30}NO_3S$ $[M+H]^+$ 316.1941; found 316.1952. **$[\alpha]_D^{25}$**: +60.29 (c 0.933, $CHCl_3$).

Ethyl (*S*)-2-(((*S*)-*tert*-butylsulfinyl)amino)-2-(1-vinylcyclopentyl)acetate (3as):

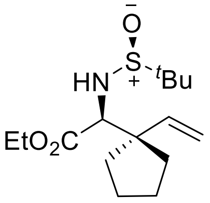

The compound **3as** was prepared according to **GP V** by using imine **1a** (61 mg, 0.3 mmol, 1.0 equiv), **2s** (0.39 mmol, 1.3 equiv) and 4Å molecular sieves (100 mg) at room temperature. Column chromatography on silica using 8% ethylacetate in hexane provided the product **3as** as colourless oil (75 mg, 83%). **^{1}H NMR** (500 MHz, $CDCl_3$): δ = 6.08 (dd, *J* = 17.5, 10.7 Hz, 1H), 5.54 (d, *J* = 10.7 Hz, 1H), 5.33 (d, *J* = 17.5 Hz, 1H), 4.30 (d, *J* = 9.8 Hz, 1H), 4.20 – 4.16 (m, 2H), 3.50 (d, *J* = 9.9 Hz, 1H), 2.30 – 2.34 (m, 1H), 1.93 – 1.65 (m, 7H), 1.26 (t, *J* = 7.0 Hz, 3H), 0.93 (s, 9H) ppm. **^{13}C NMR** (126 MHz, $CDCl_3$): δ = 172.66, 134.95, 119.15, 72.93, 66.40, 61.23, 39.69, 35.40, 34.40, 31.26, 26.54, 24.68, 24.10, 14.26 ppm. **IR (film): ν_{max}** = 2959, 1732, 1367, 1296, 1214, 1091 cm^{-1}. **HRMS-ESI:** calculated for $C_{15}H_{28}NO_3S$ $[M+H]^+$ 302.1784; found 302.1789. **$[\alpha]_D^{25}$**: +76.89 (c 1.06, $CHCl_3$).

Ethyl (*S*)-2-(((*S*)-*tert*-butylsulfinyl)amino)-2-(1-vinylcyclohexyl)acetate (3at):

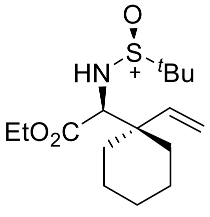

The compound **3at** was prepared according to **GP V** by using imine **1a** (61 mg, 0.3 mmol, 1.0 equiv), **2t** (0.39 mmol, 1.3 equiv) and 4Å molecular sieves (100 mg) at room temperature. Column chromatography on silica using 7% ethylacetate in hexane provided the product **3at** as colourless oil (73 mg, 77%). **^{1}H NMR** (600 MHz, $CDCl_3$): δ = 5.83 (dd, *J* = 17.7, 10.9 Hz, 1H), 5.66 (d, *J* = 10.9 Hz, 1H), 5.35 (d, *J* = 17.8 Hz, 1H), 4.31 (d, *J* = 9.7 Hz, 1H), 4.21 – 4.15 (m, 2H), 3.52 (d, *J* = 9.7 Hz, 1H), 1.95 – 1.87 (m, 2H), 1.84 – 1.80 (m, 1H), 1.77 – 1.71 (m, 1H), 1.66 – 1.61 (m, 2H), 1.58 – 1.54 (m, 1H), 1.52 – 1.38 (m, 2H), 1.38 – 1.31 (m, 1H), 1.26 (t, *J* = 7.1 Hz, 3H), 0.93 (s, 9H) ppm. **^{13}C NMR** (151 MHz, $CDCl_3$): δ = 172.66, 134.85, 120.97, 66.26, 65.54, 61.22, 35.43, 32.45, 29.58, 26.56, 25.72, 22.16, 22.00, 14.26 ppm. **IR (film): ν_{max}**

= 2954, 1738, 1456, 1385, 1269, 1088 cm^{-1}. **HRMS-ESI:** calculated for $C_{16}H_{30}NO_3S$ [M+H]$^+$ 316.1941; found 316.1943. **[α]$_D^{25}$**: +26.53 (c 0.238, $CHCl_3$).

Ethyl (2*S*,3*R*)-2-(((*S*)-*tert*-butylsulfinyl)amino)-4-methylene-3-propylhexanoate (3au):

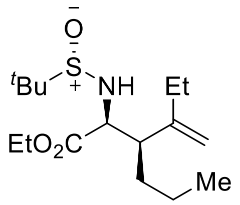

The compound **3au** was prepared according to **GP V** by using imine **1a** (61 mg, 0.3 mmol, 1.0 equiv), **2u** (0.39 mmol, 1.3 equiv) and 4Å molecular sieves (100 mg) at room temperature. Column chromatography on silica using 10% ethylacetate in hexane provided the product **3au** as colourless oil (72 mg, 76%). **^{1}H NMR** (600 MHz, $CDCl_3$): δ = 4.87 (s, 1H), 4.78 (s, 1H), 4.15 (q, *J* = 7.1 Hz, 2H), 4.01 (d, *J* = 9.3 Hz, 1H), 3.76 (t, *J* = 8.7 Hz, 1H), 2.34 (ddd, *J* = 11.2, 8.1, 3.6 Hz, 1H), 2.02 – 1.98 (m, 2H), 1.57 – 1.51 (m, 1H), 1.44 – 1.37 (m, 1H), 1.32 – 1.27 (m, 1H), 1.24 (s, 9H), 1.15 – 1.09 (m, 1H), 1.03 (t, *J* = 7.3 Hz, 3H), 0.85 (t, *J* = 7.3 Hz, 3H) ppm. **^{13}C NMR** (151 MHz, $CDCl_3$): δ = 173.43, 148.59, 111.88, 61.71, 61.46, 56.44, 50.85, 30.99, 25.69, 22.90, 20.17, 14.21, 11.91 ppm. **IR (film): $ν_{max}$** = 2928, 1735, 1342, 1287, 1174, 1075 cm^{-1}. **HRMS-ESI:** calculated for $C_{16}H_{31}NNaO_3S$ [M+Na]$^+$ 340.1917; found 340.1905. **[α]$_D^{25}$**: +49.17 (c 0.9, $CHCl_3$).

Ethyl (*S*)-2-(((*S*)-*tert*-butylsulfinyl)amino)-4-phenylpent-4-enoate (3av):

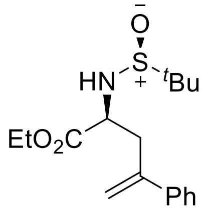

The compound **3av** was prepared according to **GP V** by using imine **1a** (61 mg, 0.3 mmol, 1.0 equiv), **2v** (0.45 mmol, 1.5 equiv) and 4Å molecular sieves (100 mg) at room temperature. Column chromatography on silica using 18% ethylacetate in hexane provided the product **3av** as colourless oil (68 mg, 70%). **^{1}H NMR** (600 MHz, $CDCl_3$): δ = 7.36 – 7.32 (m, 4H), 7.29 – 7.26 (m, 1H), 5.34 (s, 1H), 5.12 (s, 1H), 4.16 – 4.05 (m, 2H), 4.03 – 3.97 (m, 2H), 3.03 (dd, *J* = 14.2, 5.0 Hz, 1H), 2.85 (dd, *J* = 14.2, 7.2 Hz, 1H), 1.24 (t, *J* = 7.1 Hz, 3H), 1.16 (s, 9H) ppm. **^{13}C NMR** (151 MHz, $CDCl_3$) δ 173.02, 143.83, 140.28, 128.61, 127.95, 126.54, 116.68, 61.80, 57.01, 56.26, 40.54, 22.72, 14.21 ppm. **IR (film): $ν_{max}$** = 2925, 1733, 1342, 1225, 1142, 1094 cm^{-1}. **HRMS-ESI:** calculated for $C_{17}H_{25}NNaO_3S$ [M+Na]$^+$ 346.1447; found 346.1437. **[α]$_D^{25}$**: +50.43 (c 0.233, $CHCl_3$).

1-Ethyl 6-methyl (2*S*,3*R*)-2-(((*S*)-*tert*-butylsulfinyl)amino)-3-methyl-3-vinylhexanedioate (3aw):

The compound **3aw** was prepared according to **GP V** by using imine **1a** (61 mg, 0.3 mmol, 1.0 equiv), **2w** (0.39 mmol, 1.3 equiv) and 4Å molecular sieves (100 mg) at room temperature.

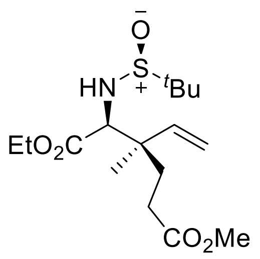

Column chromatography on silica using 12% ethylacetate in hexane as eluent provided the product **3aw** as colourless oil (82 mg, 79%). **^{1}H NMR** (600 MHz, $CDCl_3$): δ = 5.86 (dd, *J* = 17.6, 10.9 Hz, 1H), 5.57 (d, *J* = 10.8 Hz, 1H), 5.33 (d, *J* = 17.6 Hz, 1H), 4.24 (d, *J* = 9.7 Hz, 1H), 4.22 – 4.16 (m, 2H), 3.67 (s, 3H), 3.51 (d, *J* = 9.8 Hz, 1H), 2.40 – 2.35 (m, 2H), 2.19 – 2.14 (m, 1H), 2.01 – 1.96 (m, 1H), 1.38 (s, 3H), 1.27 (t, *J* = 7.1 Hz, 3H), 0.95 (s, 9H) ppm. **^{13}C NMR** (151 MHz, $CDCl_3$): δ = 173.41, 172.63, 134.88, 120.25, 66.62, 64.50, 61.38, 51.97, 35.45, 29.07, 29.00, 26.59, 18.04, 14.29 ppm. **IR (film): ν_{max}** = 2955, 1740, 1728, 1355, 1248, 1072 cm^{-1}. **HRMS-ESI:** calculated for $C_{16}H_{29}NNaO_5S$ $[M+Na]^+$ 370.1659; found 370.1662. **$[\alpha]_D^{25}$**: +65.79 (c 0.266, $CHCl_3$).

Ethyl (2*S*,3*R*,*E*)-2-(((*S*)-*tert*-butylsulfinyl)amino)-3,7-dimethyl-8-(phenylthio)-3-vinyloct-6-enoate (3ax):

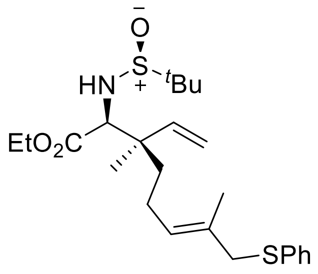

The compound **3ax** was prepared according to **GP V** by using imine **1a** (61 mg, 0.3 mmol, 1.0 equiv), **2x** (0.39 mmol, 1.3 equiv) and 4Å molecular sieves (100 mg) at room temperature. Column chromatography on silica using 7% ethylacetate in hexane as eluent provided the product **3ax** as colourless oil (57 mg, 42%). **^{1}H NMR** (500 MHz, $CDCl_3$): δ = 7.37 – 7.30 (m, 2H), 7.27 – 7.22 (m, 2H), 7.18 (t, *J* = 7.2 Hz, 1H), 5.85 (dd, *J* = 17.7, 11.0 Hz, 1H), 5.52 (d, *J* = 10.8 Hz, 1H), 5.25 (d, *J* = 17.6 Hz, 1H), 5.15 (t, *J* = 6.3 Hz, 1H), 4.21 – 4.19 (m, 3H), 3.51 – 3.47 (m, 3H), 2.04 – 1.94 (m, 2H), 1.72 (s, 3H), 1.58 – 1.50 (m, 2H), 1.36 (s, 3H), 1.28 (t, *J* = 7.1 Hz, 3H), 0.94 (s, 9H) ppm. **^{13}C NMR** (126 MHz, $CDCl_3$): δ = 172.69, 136.41, 135.83, 131.41, 130.92, 128.80, 127.91, 126.55, 119.44, 66.61, 65.07, 61.34, 44.38, 35.44, 33.73, 26.63, 22.76, 18.03, 15.38, 14.29 ppm. **IR (film): ν_{max}** = 2933, 1730, 1354, 1222, 1174, 1075 cm^{-1}. **HRMS-ESI:** calculated for $C_{24}H_{38}NO_3S_2$ $[M+H]^+$ 452.2288; found 452.2307. **$[\alpha]_D^{25}$**: +48.75 (c 0.2, $CHCl_3$).

Ethyl (2*S*,3*R*)-2-(((*S*)-*tert*-butylsulfinyl)amino)-6-((4-methoxybenzyl)oxy)-3-vinylhexanoate (3ay):

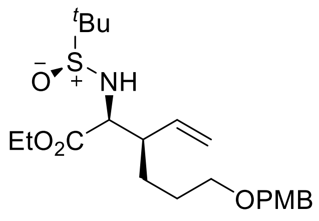

The compound **3ay** was prepared according to **GP V** by using imine **1a** (61 mg, 0.3 mmol, 1.0 equiv), **2y** (0.39 mmol, 1.3 equiv) and 4Å molecular sieves (100 mg) at room temperature. Column chromatography on silica using 25% ethylacetate in hexane as eluent provided the product **3ay** as colourless oil (126 mg, 99%).

^{1}H NMR (500 MHz, $CDCl_3$): δ = 7.23 (d, *J* = 8.5 Hz, 2H), 6.86 (d, *J* = 8.5 Hz, 2H), 5.56 (dt, *J* = 16.9, 9.7 Hz, 1H), 5.12 – 5.05 (m, 2H), 4.40 (s, 2H), 4.19 (q, *J* = 7.1 Hz, 2H), 4.06 (d, *J* = 8.8 Hz, 1H), 3.82 – 3.79 (m, 4H), 3.41 (t, *J* = 6.0 Hz, 2H), 2.38 – 2.32 (m, 1H), 1.68 – 1.59 (m, 2H), 1.54 – 1.43 (m, 1H), 1.40 – 1.32 (m, 1H), 1.26 (t, *J* = 7.1 Hz, 3H), 1.23 (s, 9H) ppm. **^{13}C NMR** (126 MHz, $CDCl_3$): δ = 172.51, 159.30, 137.37, 130.79, 129.29, 118.27, 113.90, 72.65, 69.81, 61.83, 61.61, 56.42, 55.41, 48.62, 27.37, 26.89, 22.87, 14.29 ppm. **IR (film): ν_{max}** = 2926, 1733, 1613, 1513, 1247, 1080 cm^{-1}. **HRMS-ESI:** calculated for $C_{22}H_{36}NO_5S$ $[M+H]^+$ 426.2309; found 426.2311. **$[\alpha]_D^{25}$**: +61.75 (c 0.166, $CHCl_3$).

Ethyl (*2S,3R*)-3-(2-((*N*-benzyl-4-methylphenyl)sulfonamido)ethyl)-2-(((*S*)-*tert*-butylsulfinyl)amino)pent-4-enoate (3az):

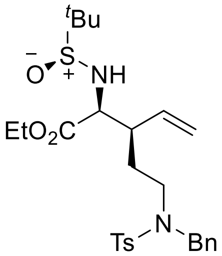

The compound **3az** was prepared according to **GP V** by using imine **1a** (61 mg, 0.3 mmol, 1.0 equiv), **2z** (0.39 mmol, 1.3 equiv) and 4Å molecular sieves (100 mg) at room temperature. Column chromatography on silica using 35% ethylacetate in hexane as eluent provided the product **3az** as colourless oil (140 mg, 87%). **^{1}H NMR** (400 MHz, $CDCl_3$): δ = 7.70 (d, *J* = 8.1 Hz, 2H), 7.33 – 7.25 (m, 7H), 5.34 (dt, *J* = 16.8, 9.6 Hz, 1H), 5.06 (d, *J* = 10.2 Hz, 1H), 4.95 (d, *J* = 17.0 Hz, 1H), 4.35 (d, *J* = 14.7 Hz, 1H), 4.17 (d, *J* = 14.8 Hz, 1H), 4.14 – 4.08 (m, 2H), 3.94 (d, *J* = 8.7 Hz, 1H), 3.61 (dd, *J* = 8.6, 5.9 Hz, 1H), 3.08 – 2.93 (m, 2H), 2.44 (s, 3H), 2.21 – 2.14 (m, 1H), 1.69 – 1.61 (m, 1H), 1.30 – 1.18 (m, 13H) ppm. **^{13}C NMR** (101 MHz, $CDCl_3$): δ = 171.90, 143.39, 136.55, 136.36, 136.11, 129.78, 128.62, 128.37, 127.92, 127.20, 118.91, 61.60, 61.33, 56.30, 52.62, 46.49, 45.97, 28.96, 22.70, 21.54, 14.14 ppm. **IR (film): ν_{max}** = 2980, 1732, 1456, 1338, 1151, 1077 cm^{-1}. **HRMS-ESI:** calculated for $C_{27}H_{39}N_2O_5S_2$ $[M+H]^+$ 535.2295; found 535.2303. **$[\alpha]_D^{25}$**: +37.03 (c 1.33, $CHCl_3$).

2,2,2-Trifluoroethyl (*2S,3R*)-2-(((*S*)-*tert*-butylsulfinyl)amino)-3,7-dimethyl-3-vinyloct-6-enoate (3ba):

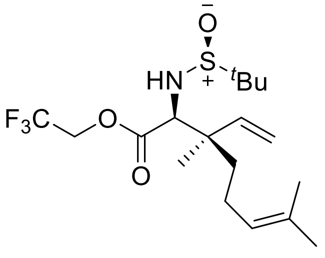

The compound **3ba** was prepared according to **GP V** by using imine **1b** (78 mg, 0.3 mmol, 1.0 equiv), geranyl boronic acid **2a** (0.39 mmol, 1.3 equiv) and 4Å molecular sieves (100 mg) at room temperature. Column chromatography on silica (Et_3N neutralized) using 6% ethylacetate in hexane as eluent provided the product **3ba** as colourless oil (78 mg, 66%). **^{1}H NMR** (600 MHz, $CDCl_3$): δ = 5.88 (dd, *J* = 17.6, 10.9 Hz, 1H), 5.56 (d, *J* = 10.9 Hz, 1H), 5.32 (d, *J* = 17.6 Hz, 1H), 5.06 (t, *J* = 7.0 Hz, 2H), 4.71 – 4.65

(m, 1H), 4.39 – 4.32 (m, 1H), 4.17 (d, *J* = 10.5 Hz, 1H), 3.62 (d, *J* = 10.5 Hz, 1H), 2.06 – 2.00 (m, 2H), 1.77 – 1.72 (m, 2H), 1.68 (s, 3H), 1.59 (s, 3H), 1.42 (s, 3H), 0.97 (s, 9H) ppm. **^{13}C NMR** (151 MHz, $CDCl_3$): δ = 171.38, 135.53, 132.61, 123.64 (q, *J* = 265 Hz), 123.47, 119.82, 66.48, 65.28, 60.85 (q, *J* = 36 Hz), 35.52, 34.02, 26.40, 25.82, 22.66, 18.38, 17.84 ppm. **IR (film): ν_{max}** = 2968, 1756, 1277, 1162, 1090 cm^{-1}. **HRMS-ESI:** calculated for $C_{18}H_{31}F_3NO_3S$ $[M+H]^+$ 398.1971, found 398.1984. **$[\alpha]_D^{25}$**: +85.36 (c 1.45, $CHCl_3$).

Benzyl (2*S*,3*R*)-2-(((*S*)-*tert*-butylsulfinyl)amino)-3,7-dimethyl-3-vinyloct-6-enoate (3ca):

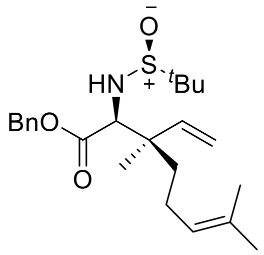

The compound **3ca** was prepared according to **GP V** by using imine **1c** (80 mg, 0.3 mmol, 1.0 equiv), geranyl boronic acid **2a** (0.39 mmol, 1.3 equiv) and 4Å molecular sieves (100 mg) at room temperature. Column chromatography on silica using 7% ethylacetate in hexane as eluent provided the product **3ca** as colourless oil (87 mg, 72%). **^{1}H NMR** (600 MHz, $CDCl_3$): δ = 7.37 – 7.31 (m, 5H), 5.90 (dd, *J* = 17.6, 10.9 Hz, 1H), 5.52 (d, *J* = 10.9 Hz, 1H), 5.30 (d, *J* = 17.6 Hz, 1H), 5.20 (d, *J* = 12.3 Hz, 1H), 5.11 (d, *J* = 12.2 Hz, 1H), 5.06 (t, *J* = 7.0 Hz, 1H), 4.25 (d, *J* = 9.9 Hz, 1H), 3.58 (d, *J* = 10.0 Hz, 1H), 2.06 – 1.98 (m, 2H), 1.79 – 1.74 (m, 1H), 1.68 – 1.64 (m, 4H), 1.58 (s, 3H), 1.41 (s, 3H), 0.92 (s, 9H) ppm. **^{13}C NMR** (151 MHz, $CDCl_3$): δ = 172.62, 135.80, 135.43, 132.41, 128.72, 128.63, 128.47, 123.59, 119.49, 67.26, 66.57, 65.21, 35.57, 34.11, 26.57, 25.76, 22.67, 18.23, 17.79 ppm. **IR (film): ν_{max}** = 2965, 1733, 1291, 1210, 1165, 1083 $cm^{-1.}$ **HRMS-ESI:** calculated for $C_{23}H_{35}NO_3SNa$ $[M+Na]^+$ 428.2235; found 428.2233. **$[\alpha]_D^{25}$**: +165.8 (c 0.48, $CHCl_3$).

(2*S*,3*R*)-2-(((*S*)-*tert*-Butylsulfinyl)amino)-3,7-dimethyl-3-vinyloct-6-enoic acid (3da):

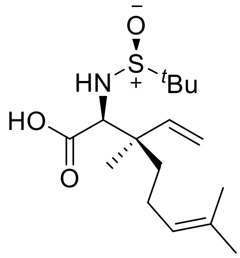

The compound **3da** was prepared according to **GP V** by using imine **1d** (added as a crude), geranyl boronic acid **2a** (0.39 mmol, 1.3 equiv) and 4Å molecular sieves (100 mg) at -30°C. Column chromatography on silica using 0.5% MeOH in DCM provided the product **3da** as colourless oil (62 mg, 65% over two steps from glyoxalic acid). **^{1}H NMR** (500 MHz, $CDCl_3$): δ = 5.86 (dd, *J* = 17.5, 10.8 Hz, 1H), 5.26 (d, *J* = 10.8 Hz, 1H), 5.10 (d, *J* = 17.6 Hz, 1H), 5.05 (t, *J* = 7.5 Hz, 1H), 4.04 (d, *J* = 9.2 Hz, 1H), 3.77 (d, *J* = 9.2 Hz, 1H), 2.04 – 1.96 (m, 1H), 1.91 – 1.85 (m, 1H), 1.66 (s, 3H), 1.60 – 1.53 (m, 4H), 1.44 – 1.37 (m, 1H), 1.25 (s, 3H), 1.19 (s, 9H) ppm. **^{13}C NMR** (126 MHz, $CDCl_3$): δ = 174.49, 141.21, 131.83, 124.24, 116.85, 65.46, 57.16, 43.89, 38.00, 29.83, 25.80, 22.92, 22.61, 19.29, 17.77 ppm. **IR (film): ν_{max}** =

3402, 1721, 1461,1375, 1275, 1091 cm^{-1}. **HRMS-ESI:** calculated for $C_{16}H_{30}NO_3S$ $[M+H]^+$ 316.1941; found 314.1952. **[α]$_D$^{25}**: +96.20 (c 0.586, $CHCl_3$).

(2*S*,3*R*)-2-(((*S*)-*tert*-butylsulfinyl)amino)-3,7-dimethyl-*N*-(*o*-tolyl)-3-vinyloct-6-enamide (3ea):

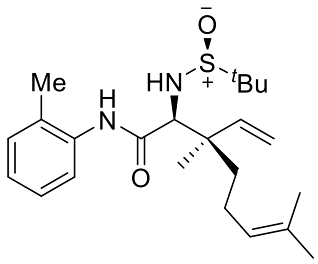

The compound **3ea** was prepared according to **GP V** by using imine **1e** (80 mg, 0.3 mmol, 1.0 equiv), geranyl boronic acid **2a** (0.39 mmol, 1.3 equiv) and 4Å molecular sieves (100 mg) at room temperature. Column chromatography on silica using 30% ethylacetate in hexane as eluent provided the product **3ea** as colourless oil (94 mg, 77%). **^{1}H NMR** (500 MHz, $CDCl_3$): δ = 8.10 (br s, 1H), 7.54 (d, *J* = 7.2 Hz, 1H), 7.10 (d, *J* = 8.1 Hz, 1H), 7.04 – 6.99 (m, 2H), 5.97 (dd, *J* = 17.5, 10.8 Hz, 1H), 5.22 (d, *J* = 10.9 Hz, 1H), 5.10 – 5.04 (m, 2H), 4.61 (d, *J* = 8.3 Hz, 1H), 3.85 (d, *J* = 8.3 Hz, 1H), 2.21 (s, 3H), 1.92 (q, *J* = 8.0 Hz, 2H), 1.67 (s, 3H), 1.63 (s, 3H), 1.61 – 1.58 (m, 1H), 1.53 – 1.46 (m, 1H), 1.31 (s, 9H), 1.11 (s, 3H) ppm. **^{13}C NMR** (126 MHz, $CDCl_3$): δ = 169.69, 142.45, 135.40, 131.70, 130.53, 130.43, 126.51, 125.50, 124.45, 123.99, 115.75, 66.37, 56.80, 45.00, 38.43, 25.79, 23.14, 22.84, 18.26, 17.83 ppm. **IR (film): ν_{max}** = 3325, 1657, 1324, 1211, 1185, 1094 cm^{-1}. **HRMS-ESI:** calculated for $C_{23}H_{37}N_2O_2S$ $[M+H]^+$ 405.2570; found 405.2587. **[α]$_D$^{25}**: +54.12 (c 0.259, $CHCl_3$).

(2*S*,3*R*)-*N*-Benzyl-2-(((*S*)-*tert*-butylsulfinyl)amino)-3,7-dimethyl-3-vinyloct-6-enamide (3fa):

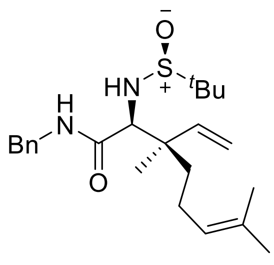

The compound **3fa** was prepared according to **GP V** by using imine **1f** (80 mg, 0.3 mmol, 1.0 equiv), geranyl boronic acid **2a** (0.45 mmol, 1.5 equiv) and 4Å molecular sieves (100 mg) at room temperature. Column chromatography on silica using 30% ethylacetate in hexane as eluent provided the product **3fa** as colourless oil (102 mg, 84%). **^{1}H NMR** (600 MHz, $CDCl_3$): δ = 7.30 – 7.21 (m, 5H), 6.95 (s, 1H), 5.81 (dd, *J* = 17.5, 10.8 Hz, 1H), 5.07 (d, *J* = 10.8 Hz, 1H), 4.97 – 4.92 (m, 2H), 4.55 – 4.50 (m, 2H), 4.19 (dd, *J* = 14.6, 4.2 Hz, 1H), 3.54 (d, *J* = 8.4 Hz, 1H), 1.84 – 1.79 (m, 2H), 1.63 (s, 3H), 1.51 (s, 3H), 1.45 – 1.40 (m, 1H), 1.36 – 1.30 (m, 1H), 1.23 (s, 9H), 0.99 (s, 3H) ppm. **^{13}C NMR** (151 MHz, $CDCl_3$) δ 170.92, 142.29, 138.09, 131.55, 128.61, 128.30, 127.41, 124.45, 115.38, 65.91, 56.70, 44.83, 43.90, 38.24, 25.79, 23.06, 22.69, 18.17, 17.76 ppm. **IR (film): ν_{max}** = 3452, 1666, 1452, 1375,

1215, 1094 cm^{-1}. **HRMS-ESI:** calculated for $C_{23}H_{37}N_2O_2S$ $[M+H]^+$ 405.2570; found 405.2587. **[α]$_D$^{25}**: +97.21 (c 0.496, $CHCl_3$).

Ethyl (2S,3R)-3,7-dimethyl-2-(((S)-p-tolylsulfinyl)amino)-3-vinyloct-6-enoate (3ga):

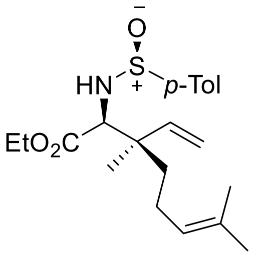

The compound **3ga** was prepared according to **GP V** by using imine **1g** (72 mg, 0.3 mmol, 1.0 equiv), geranyl boronic acid **2a** (0.45 mmol, 1.5 equiv) and 4Å molecular sieves (100 mg) at room temperature. Column chromatography on silica using 8% ethylacetate in hexane as eluent provided the product **3ga** as colourless oil (63 mg, 56%). **^{1}H NMR** (400 MHz, $CDCl_3$): δ = 7.57 (d, *J* = 8.1 Hz, 2H), 7.29 (d, *J* = 8.9 Hz, 2H), 5.82 (dd, *J* = 17.4, 10.8 Hz, 1H), 5.18 (d, *J* = 10.8 Hz, 1H), 5.06 – 5.01 (m, 2H), 4.64 (d, *J* = 9.5 Hz, 1H), 4.08 – 3.96 (m, 2H), 3.74 (d, *J* = 9.7 Hz, 1H), 2.41 (s, 3H), 2.00 – 1.88 (m, 2H), 1.67 (s, 3H), 1.58 (s, 3H), 1.54 – 1.46 (m, 1H), 1.36 – 1.28 (m, 1H), 1.17 (t, *J* = 7.1 Hz, 3H), 1.11 (s, 3H) ppm. **^{13}C NMR** (101 MHz, $CDCl_3$): δ = 171.95, 141.83, 141.57, 141.38, 131.78, 129.60, 125.61, 124.18, 115.76, 62.61, 61.05, 43.84, 38.18, 25.76, 22.67, 21.44, 18.47, 17.74, 14.09 ppm. **IR (film): ν$_{max}$** = 2975, 1732, 1446, 1185, 1070, 810 cm^{-1}. **HRMS-ESI:** calculated for $C_{21}H_{32}NO_3S$ $[M+H]^+$ 378.2097; found 378.2082. **[α]$_D$^{25}**: +32.50 (c 0.1, $CHCl_3$).

Ethyl (2*R*,3*S*)-2-(((*R*)-*tert*-butylsulfinyl)amino)-3,7-dimethyl-3-vinyloct-6-enoate (*ent*-3aa):

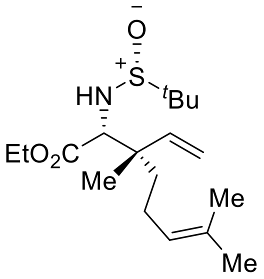

The compound ***ent*-3aa** was prepared according to **GP V** by using imine ***ent*-1a** (61 mg, 0.3 mmol, 1.0 equiv), geranyl boronic acid **2a** (0.45 mmol, 1.5 equiv) and 4Å molecular sieves (100 mg) at room temperature. Column chromatography on silica using 7% ethylacetate in hexane as eluent provided the product ***ent*-3aa** as colourless oil (73 mg, 71%). **^{1}H NMR** (500 MHz, $CDCl_3$) δ 5.87 (dd, *J* = 17.6, 10.9 Hz, 1H), 5.51 (d, *J* = 10.9 Hz, 1H), 5.28 (d, *J* = 17.6 Hz, 1H), 5.04 (t, *J* = 7.1 Hz, 1H), 4.21 – 4.15 (m, 3H), 3.49 (d, *J* = 9.8 Hz, 1H), 2.03 – 1.97 (m, 2H), 1.77 – 1.71 (m, 1H), 1.66 (s, 3H), 1.64 – 1.61 (m, 1H), 1.56 (s, 3H), 1.38 (s, 3H), 1.25 (t, *J* = 7.1 Hz, 3H), 0.93 (s, 9H) ppm. **^{13}C NMR** (126 MHz, $CDCl_3$): δ = 172.66, 135.87, 132.35, 123.61, 119.34, 66.57, 65.14, 61.24, 35.39, 34.14, 26.56, 25.73, 22.66, 18.12, 17.76, 14.23 ppm. **^{13}C NMR** (126 MHz, $CDCl_3$): δ = 172.66, 135.87, 132.35, 123.61, 119.34, 66.57, 65.14, 61.24, 35.39, 34.14, 26.56, 25.73, 22.66, 18.12, 17.76, 14.23 ppm. **IR (film): ν$_{max}$** = 2966, 1733, 1369, 1294, 1213, 1081 cm^{-1}. **HRMS-ESI:** calculated for $C_{18}H_{34}NO_3S$ $[M+H]^+$ 344.2259; found 344.2265. **[α]$_D$^{25}**: -66.44 (c 0.66, $CHCl_3$).

Ethyl (*2R,3R*)-2-((*R*)-*tert*-butylsulfinyl)amino)-3,7-dimethyl-3-vinyloct-6-enoate (*ent*-3ab):

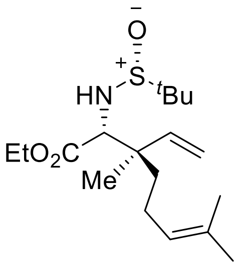

The compound ***ent*-3ab** was prepared according to **GP V** by using imine ***ent*-1a** (61 mg, 0.3 mmol, 1.0 equiv), neryl boronic acid **2b** (0.45 mmol, 1.5 equiv) and 4Å molecular sieves (100 mg) at room temperature. Column chromatography on silica using 7% ethylacetate in hexane as eluent provided the product ***ent*-3ab** as colourless oil (75 mg, 73%). **^{1}H NMR** (500 MHz, $CDCl_3$): δ = 6.00 (dd, *J* = 17.6, 10.8 Hz, 1H), 5.59 (d, *J* = 10.8 Hz, 1H), 5.31 (d, *J* = 17.6 Hz, 1H), 5.09 (t, *J* = 7.1 Hz, 1H), 4.32 (d, *J* = 9.8 Hz, 1H), 4.21 – 4.15 (m, 2H), 3.51 (d, *J* = 9.9 Hz, 1H), 2.09 – 2.01 (m, 1H), 1.99 – 1.91 (m, 1H), 1.83 (td, *J* = 12.6, 11.6, 4.9 Hz, 1H), 1.75 (ddd, *J* = 13.5, 11.5, 5.6 Hz, 1H), 1.65 (s, 3H), 1.57 (s, 3H), 1.28 (s, 3H), 1.26 (t, *J* = 7.2 Hz, 3H), 0.93 (s, 9H) ppm. **^{13}C NMR** (126 MHz, $CDCl_3$): δ = 172.67, 135.44, 132.41, 123.54, 119.83, 66.28, 65.44, 61.23, 35.99, 35.41, 26.57, 25.71, 22.59, 17.74, 16.72, 14.25 ppm. **IR (film): ν_{max}** = 2966, 1733, 1368, 1295, 1213, 1090 cm^{-1}. **HRMS-ESI:** calculated for $C_{18}H_{33}NO_3SNa$ $[M+Na]^+$ 366.2079; found 366.2079. **$[\alpha]_D^{25}$**: -149.62 (c 0.533, $CHCl_3$).

Methyl ((*2S,3R*)-2-(((*S*)-*tert*-butylsulfinyl)amino)-3,7-dimethyl-3-vinyloct-6-enoyl)-*L*-phenylalaninate (5aa):

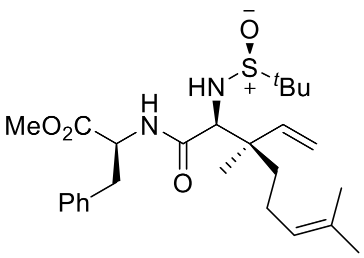

The compound **5aa** was synthesized according to **GP V** by using imine **4a** (34 mg, 0.1 mmol), geranyl boronic acid **2a** (0.25 mmol, 2.5 equiv), and 4Å molecular sieves (100 mg) at room temperature. Column chromatography on silica using 22% ethylacetate in hexane as eluent provided the product **5aa** as colourless oil (39 mg, 83%). **^{1}H NMR** (500 MHz, $CDCl_3$): δ = 7.29 (t, *J* = 7.3 Hz, 2H), 7.23 (t, *J* = 7.3 Hz, 1H), 7.15 (d, *J* = 7.1 Hz, 2H), 6.34 (d, *J* = 7.3 Hz, 1H), 5.75 (dd, *J* = 17.5, 10.8 Hz, 1H), 5.06 (d, *J* = 10.9 Hz, 1H), 5.01 – 4.97 (m, 2H), 4.81 (q, *J* = 6.3 Hz, 1H), 4.50 (d, *J* = 8.2 Hz, 1H), 3.68 (s, 3H), 3.48 (d, *J* = 8.3 Hz, 1H), 3.15 (dd, *J* = 13.8, 4.9 Hz, 1H), 3.07 (dd, *J* = 13.8, 6.4 Hz, 1H), 1.85 – 1.81 (m, 2H), 1.64 (s, 3H), 1.54 (s, 3H), 1.49 – 1.43 (m, 2H), 1.27 (s, 9H), 0.99 (s, 3H) ppm. **^{13}C NMR** (126 MHz, $CDCl_3$): δ = 171.62, 170.58, 142.39, 135.80, 131.59, 129.66, 128.76, 127.21, 124.42, 115.88, 65.95, 56.75, 53.56, 52.23, 44.75, 38.04, 37.72, 25.75, 23.08, 22.69, 18.06, 17.77 ppm. **IR (film): ν_{max}** = 3247, 2958, 737, 1652, 1527, 1082 cm^{-1}. **HRMS-ESI:** calculated for $C_{26}H_{41}N_2O_4S$ $[M+H]^+$ 477.2782; found 477.2891. **$[\alpha]_D^{25}$**: + 123.0 (c 0.52, $CHCl_3$).

Methyl ((2*S*,3*S*)-2-(((*S*)-*tert*-butylsulfinyl)amino)-3,7-dimethyl-3-vinyloct-6-enoyl)-*L*-phenylalaninate (5ab):

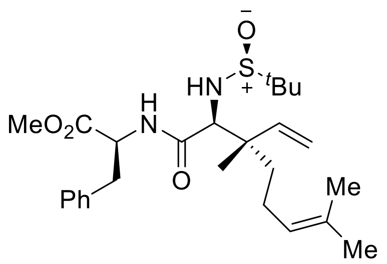

The compound **5ab** was synthesized according to **GP V** by using imine **4a** (34 mg, 0.1 mmol), neryl boronic acid **2b** (0.25 mmol, 2.5 equiv), and 4Å molecular sieves (100 mg) at room temperature. Column chromatography on silica using 20% ethylacetate in hexane as eluent provided the product **5ab** as foamy solid (47 mg, 87%). **^{1}H NMR** (500 MHz, $CDCl_3$): δ = 7.29 (t, *J* = 7.3 Hz, 2H), 7.23 (t, *J* = 7.3 Hz, 1H), 7.16 (d, *J* = 7.1 Hz, 1H), 6.45 – 6.41 (m, 1H), 5.75 (dd, *J* = 17.6, 10.9 Hz, 1H), 5.09 (d, *J* = 10.9 Hz, 1H), 5.03 – 4.98 (m, 2H), 4.83 (q, *J* = 6.3 Hz, 1H), 4.43 (d, *J* = 8.1 Hz, 1H), 3.68 (s, 3H), 3.49 (d, *J* = 8.1 Hz, 1H), 3.16 (dd, *J* = 13.8, 5.1 Hz, 1H), 3.08 (dd, *J* = 13.8, 6.4 Hz, 1H), 1.87 – 1.81 (m, 2H), 1.65 (s, 3H), 1.55 (s, 3H), 1.47 – 1.42 (m, 1H), 1.39 – 1.34 (m, 1H), 1.26 (s, 9H), 1.05 (s, 3H) ppm. **^{13}C NMR** (126 MHz, $CDCl_3$): δ = 171.70, 170.54, 143.20, 135.82, 131.56, 129.64, 128.77, 127.21, 124.52, 115.18, 66.40, 56.78, 53.65, 52.25, 44.30, 37.71, 36.19, 25.75, 23.08, 22.71, 20.01, 17.77 ppm. **IR (film): ν_{max}** = 3261, 2964, 1748, 1671, 1548, 1050 cm^{-1}. **HRMS-ESI:** calculated for $C_{26}H_{41}N_2O_4S$ $[M+H]^+$ 477.2782; found 477.2808**. [α]$_D$^{20}**: +161.7 (c 0.4, $CHCl_3$).

Methyl ((2*S*)-2-(((*S*)-*tert*-butylsulfinyl)amino)-3,3-dimethylpent-4-enoyl)-*L*-phenylalaninate (5ac):

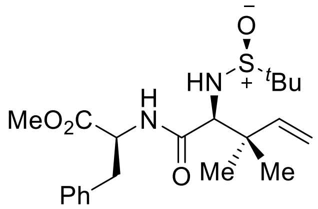

The compound **5ac** was synthesized according to **GP V** by using imine **4a** (34 mg, 0.1 mmol), prenyl boronic acid **2d** (0.25 mmol, 2.5 equiv), and 4Å molecular sieves (100 mg) at room temperature. Column chromatography on silica using 18% ethylacetate in hexane as eluent provided the product **5ac** as foamy solid (42 mg, 83%). **^{1}H NMR** (500 MHz, $CDCl_3$): δ = 7.30 (t, *J* = 7.4 Hz, 2H), 7.23 (t, *J* = 7.3 Hz, 1H), 7.17 (d, *J* = 7.6 Hz, 2H), 6.17 (d, *J* = 7.4 Hz, 1H), 6.06 (dd, *J* = 17.5, 10.8 Hz, 1H), 5.51 (d, *J* = 10.7 Hz, 1H), 5.32 (d, *J* = 17.5 Hz, 1H), 4.87 (q, *J* = 6.3 Hz, 1H), 4.51 (d, *J* = 8.6 Hz, 1H), 3.69 (s, 3H), 3.30 (d, *J* = 8.7 Hz, 1H), 3.17 (dd, *J* = 13.8, 4.8 Hz, 1H), 3.08 (dd, *J* = 13.8, 6.3 Hz, 1H), 1.44 (s, 3H), 1.34 (s, 3H), 0.93 (s, 9H) ppm. **^{13}C NMR** (126 MHz, $CDCl_3$): δ = 171.81, 170.94, 136.64, 135.74, 129.71, 128.80, 127.23, 118.86, 67.39, 61.96, 53.36, 52.30, 37.83, 35.67, 26.64, 22.77, 20.68 ppm. **IR (film): ν_{max}** = 3268, 2958, 1747, 1671, 1210, 1051 cm^{-1}. **HRMS-ESI:** calculated for $C_{21}H_{33}N_2O_4S$ $[M+H]^+$ 409.2156; found 409.2170**. [α]$_D$^{25}**: +53.24 (c 0.693, $CHCl_3$).

Methyl ((2*S*,3*S*)-2-(((*S*)-*tert*-butylsulfinyl)amino)-3-phenylpent-4-enoyl)-*L*-phenylalaninate (5ad):

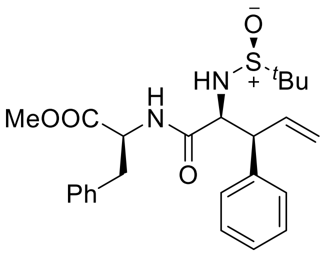

The compound **5ad** was synthesized according to **GP V** by using imine **9a** (34 mg, 0.1 mmol), cinnamyl boronic acid **2e** (0.25 mmol, 2.5 equiv), and 4Å molecular sieves (100 mg) at -50 °C. Column chromatography on silica using 28% ethylacetate in hexane as eluent provided the product **5ad** as foamy solid (40 mg, 88%). **^{1}H NMR** (500 MHz, $CDCl_3$): δ = 7.35 (d, *J* = 7.1 Hz, 1H), 7.29 – 7.26 (m, 2H), 7.23 (d, *J* = 7.0 Hz, 1H), 7.19 (d, *J* = 7.0 Hz, 2H), 7.15 – 7.14 (m, 3H), 6.92 – 6.90 (m, 2H), 6.07 (ddd, *J* = 17.7, 10.4, 7.6 Hz, 1H), 5.18 – 5.13 (m, 2H), 4.75 (q, *J* = 6.6 Hz, 1H), 4.21 – 4.19 (m, 1H), 4.13 (dd, *J* = 9.9, 4.9 Hz, 1H), 3.70 (s, 3H), 3.59 (d, *J* = 10.0 Hz, 1H), 3.05 – 2.97 (m, 2H), 1.10 (s, 9H) ppm. **^{13}C NMR** (126 MHz, $CDCl_3$): δ = 171.63, 171.01, 139.07, 137.30, 136.17, 129.38, 129.16, 128.68, 128.52, 127.36, 127.00, 117.49, 64.23, 56.93, 54.03, 52.38, 51.26, 37.68, 22.69 ppm. **IR (film): $ν_{max}$** = 3279, 1744, 1658, 1542, 1204, 1046 cm^{-1}. **HRMS-ESI:** calculated for $C_{25}H_{32}N_2O_4SNa$ $[M+Na]^+$ 479.1980; found 479.1981 **. $[α]_D^{20}$**: +48.2 (c 0.14, $CHCl_3$).

Methyl ((2*S*,3*S*)-3-(2-bromophenyl)-2-(((*S*)-*tert*-butylsulfinyl)amino)pent-4-enoyl)-*L*-phenylalaninate (5ae):

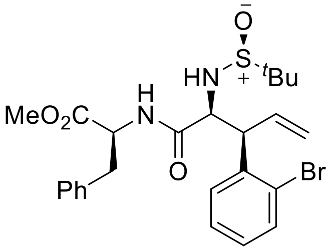

The compound **5ae** was synthesized according to **GP V** by using imine **4a** (34 mg, 0.1 mmol), **2j** (0.25 mmol, 2.5 equiv), and 4Å molecular sieves (100 mg) at room temperature. Column chromatography on silica using 25% ethylacetate in hexane as eluent provided the product **5ae** as foamy solid (40 mg, 75%). **^{1}H NMR** (500 MHz, $CDCl_3$): δ = 7.54 (d, *J* = 7.9 Hz, 1H), 7.30 – 7.27 (m, 2H), 7.25 – 7.21 (m, 3H), 7.17 – 7.15 (m, 2H), 7.08 – 7.04 (m, 1H), 6.54 (d, *J* = 7.2 Hz, 1H), 5.98 (ddd, *J* = 17.0, 10.3, 8.3 Hz, 1H), 5.14 – 5.09 (m, 2H), 4.85 – 4.81 (m, 1H), 4.25 (t, *J* = 8.4 Hz, 1H), 4.13 (d, *J* = 9.2 Hz, 1H), 4.02 (t, *J* = 8.9 Hz, 1H), 3.70 (s, 3H), 3.16 – 3.08 (m, 2H), 1.01 (s, 9H) ppm. **^{13}C NMR** (126 MHz, $CDCl_3$): δ = 171.62, 170.49, 139.67, 135.98, 135.40, 133.28, 129.88, 129.67, 128.74, 128.47, 127.62, 127.18, 125.69, 119.14, 63.40, 56.35, 53.65, 52.59, 52.37, 37.98, 22.52 ppm. **IR (film): $ν_{max}$** = 3292, 1745, 1672, 1546, 1219, 1050 cm^{-1}. **HRMS-ESI:** calculated for $C_{25}H_{32}N_2O_4BrS$ $[M+H]^+$ 535.1266; found 535.1259. **$[α]_D^{25}$**: +13.75 (c 0.4, $CHCl_3$).

Methyl ((2*S*,3*S*)-3-(4-bromophenyl)-2-(((*S*)-*tert*-butylsulfinyl)amino)pent-4-enoyl)-*L*-phenylalaninate (5af):

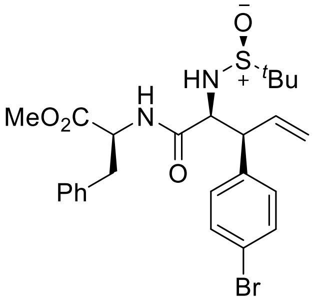

The compound **5af** was synthesized according to **GP V** by using imine **4a** (34 mg, 0.1 mmol), **2k** (0.25 mmol, 2.5 equiv), and 4Å molecular sieves (100 mg) at room temperature. Column chromatography on silica using 27% ethylacetate in hexane as eluent provided the product **5af** as yellow foamy solid (42 mg, 79%). **^{1}H NMR** (500 MHz, $CDCl_3$): δ = 7.43 (d, *J* = 7.4 Hz, 1H), 7.36 (d, *J* = 8.4 Hz, 2H), 7.20 – 7.14 (m, 3H), 7.03 (d, *J* = 8.4 Hz, 2H), 6.95 (d, *J* = 6.8 Hz, 2H), 6.00 (ddd, *J* = 17.5, 10.4, 7.4 Hz, 1H), 5.18 (d, *J* = 10.4 Hz, 1H), 5.14 (d, *J* = 17.2 Hz, 1H), 4.71 (q, *J* = 7.4 Hz, 1H), 4.19 – 4.17 (m, 1H), 4.12 (dd, *J* = 10.1, 4.5 Hz, 1H), 3.72 (s, 3H), 3.41 (d, *J* = 10.1 Hz, 1H), 3.09 (dd, *J* = 13.9, 5.6 Hz, 1H), 2.96 (dd, *J* = 13.9, 7.5 Hz, 1H), 1.14 (s, 9H) ppm. **^{13}C NMR** (126 MHz, $CDCl_3$): δ = 171.61, 170.72, 137.99, 136.87, 136.16, 131.68, 130.92, 129.31, 128.55, 127.12, 121.34, 117.85, 64.02, 57.03, 54.06, 52.46, 50.32, 37.61, 22.73 ppm. **IR (film): ν_{max}** = 3286, 2924, 1743, 1660, 1488, 1042 cm^{-1}. **HRMS-ESI:** calculated for $C_{25}H_{31}BrN_2O_4S$ $[M+Na]^+$ 557.1086; found 557.1083. **$[\alpha]_D^{25}$**: +26.25 (c 0.2, $CHCl_3$).

Methyl ((2*S*,3*S*)-2-(((*S*)-*tert*-butylsulfinyl)amino)-3-(1-tosyl-1*H*-indol-3-yl)pent-4-enoyl)-*L*-phenylalaninate (5ag):

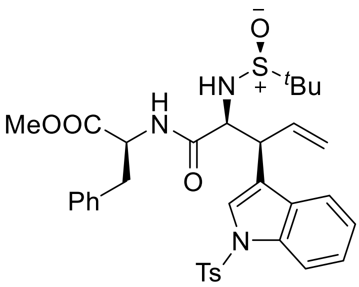

The compound **5ag** was synthesized according to **GP V** by using imine **4a** (34 mg, 0.1 mmol), **2o** (0.25 mmol, 2.5 equiv), 4Å molecular sieves (100 mg) at room temperature. Column chromatography on silica using 25% ethylacetate in hexane as eluent provided the product **5ag** as colourless oil (56 mg, 86%). **^{1}H NMR** (400 MHz, $CDCl_3$): δ = 7.98 (d, *J* = 8.3 Hz, 1H), 7.76 (d, *J* = 8.3 Hz, 2H), 7.64 (d, *J* = 7.9 Hz, 1H), 7.40 (s, 1H), 7.32 (t, *J* = 7.8 Hz, 1H), 7.25 – 7.20 (m, 3H), 7.11 (d, *J* = 7.3 Hz, 1H), 7.08 (d, *J* = 7.3 Hz, 1H), 7.02 (t, *J* = 7.3 Hz, 2H), 6.71 (d, *J* = 8.0 Hz, 2H), 6.05 (ddd, *J* = 17.3, 10.4, 7.4 Hz, 1H), 5.19 – 5.12 (m, 2H), 4.65 (q, *J* = 6.6 Hz, 1H), 4.38 – 4.35 (m, 1H), 4.19 (dd, *J* = 9.5, 4.5 Hz, 1H), 3.75 (d, *J* = 9.5 Hz, 1H), 3.69 (s, 3H), 2.85 (d, *J* = 6.3 Hz, 2H), 2.32 (s, 3H), 1.09 (s, 9H) ppm. **^{13}C NMR** (101 MHz, $CDCl_3$): δ = 171.50, 170.40, 145.18, 136.24, 135.65, 135.35, 135.12, 130.06, 129.90, 129.05, 128.43, 127.09, 126.97, 125.16, 124.74, 123.32, 120.84, 120.09, 117.80, 113.67, 62.61, 56.89, 53.74,

52.44, 43.53, 37.48, 22.62, 21.67 ppm. **IR (film):** ν_{max} = 1743, 1689, 1447, 1365, 1175, 1122 cm^{-1}. **HRMS-ESI:** calculated for $C_{34}H_{40}N_3O_6S_2$ $[M+H]^+$ 650.2353; found 650.2362. **[α]$_D$^{25}**: +64.4 (c 0.26, $CHCl_3$).

Methyl ((*2S,3R*)-2-(((*S*)-*tert*-butylsulfinyl)amino)-3-(thiophen-2-yl)pent-4-enoyl)-*L*-phenylalaninate (5ah):

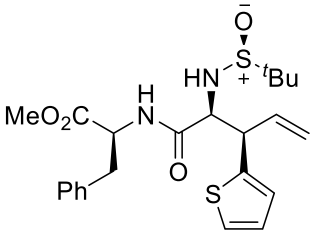

The compound **5ah** was synthesized according to **GP V** by using imine **4a** (34 mg, 0.1 mmol), **2m** (0.25 mmol, 2.5 equiv), and 4Å molecular sieves at room temperature. Column chromatography on silica using 25% ethylacetate in hexane as eluent provided the product **5ah** as foamy solid (16 mg, 35%). **^{1}H NMR** (500 MHz, $CDCl_3$): δ = 7.75 (d, *J* = 7.2 Hz, 1H), 7.18 – 7.15 (m, 3H), 7.10 (t, *J* = 7.7 Hz, 1H), 6.95 – 6.90 (m, 3H), 6.79 (d, *J* = 3.4 Hz, 1H), 6.03 (ddd, *J* = 17.3, 10.3, 7.8 Hz, 1H), 5.24 (d, *J* = 17.1 Hz, 1H), 5.20 (d, *J* = 10.3 Hz, 1H), 4.77 – 4.73 (m, 1H), 4.65 (dd, *J* = 7.6, 2.8 Hz, 1H), 4.12 (dd, *J* = 10.5, 3.2 Hz, 1H), 3.72 (s, 3H), 3.60 (d, *J* = 10.5 Hz, 1H), 3.08 (dd, *J* = 13.9, 5.6 Hz, 1H), 2.97 (dd, *J* = 13.9, 7.5 Hz, 1H), 1.19 (s, 9H) ppm. **^{13}C NMR** (126 MHz, $CDCl_3$): δ = 171.59, 170.59, 141.14, 137.40, 136.41, 129.37, 128.51, 127.12, 126.97, 126.51, 124.84, 117.74, 64.90, 57.23, 54.23, 52.42, 46.52, 37.64, 22.79 ppm. **IR (film):** ν_{max} = 3288, 2926, 1742, 1671, 1258, 1029 cm^{-1}. **HRMS-ESI:** calculated for $C_{23}H_{31}N_2O_4S_2$ $[M+H]^+$ 463.1725; found 463.1772. **[α]$_D$^{25}**: +22.73 (c 0.33, $CHCl_3$).

Methyl ((*2S,3R*)-2-(((*S*)-*tert*-butylsulfinyl)amino)-3-(furan-2-yl)pent-4-enoyl)-*L*-phenylalaninate (5ai):

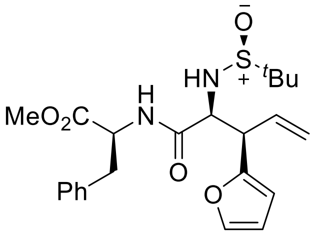

The compound **5ai** was synthesized according to **GP V** by using imine **4a** (34 mg, 0.1 mmol), **2n** (0.25 mmol, 2.5 equiv), and 4Å molecular sieves at room temperature. Column chromatography on silica using 25% ethylacetate in hexane as eluent provided the product **5ai** as colourless liquid (19 mg, 42%). **^{1}H NMR** (500 MHz, $CDCl_3$): δ = 7.53 (d, *J* = 7.2 Hz, 1H), 7.24 – 7.21 (m, 3H), 7.10 – 7.08 (m, 3H), 6.25 (dd, *J* = 3.1, 1.9 Hz, 1H), 6.03 (d, *J* = 3.2 Hz, 1H), 5.93 (ddd, *J* = 17.7, 10.3, 7.8 Hz, 1H), 5.19 (d, *J* = 10.3 Hz, 1H), 5.16 (d, *J* = 17.1 Hz, 1H), 4.75 – 4.70 (m, 1H), 4.29 (dd, *J* = 7.7, 3.9 Hz, 1H), 4.13 (dd, *J* = 9.9, 4.1 Hz, 1H), 3.91 (d, *J* = 10.0 Hz, 1H), 3.70 (s, 3H), 3.10 (dd, *J* = 13.8, 5.6 Hz, 1H), 2.99 (dd, *J* = 14.0, 7.5 Hz, 1H), 1.19 (s, 9H) ppm. **^{13}C NMR** (126 MHz, $CDCl_3$): δ = 171.61, 170.61, 152.86, 142.00, 136.48, 135.01, 129.47, 128.58, 127.02, 118.54, 110.60,

108.29, 63.61, 57.01, 54.16, 52.39, 45.91, 37.74, 22.73 ppm. **IR (film):** ν_{max} = 3296, 2927, 1746, 1658, 1214, 1051 cm^{-1}. **HRMS-ESI:** calculated for $C_{23}H_{31}N_2O_5SNa$ $[M+Na]^+$ 469.1773; found 469.1772. **[α]$_D$^{20}:** +13.9 (c 0.2, $CHCl_3$).

Methyl ((2*S*)-2-(((*S*)-*tert*-butylsulfinyl)amino)-2-(1-vinylcyclohexyl)acetyl)-*L*-phenylalaninate (5aj):

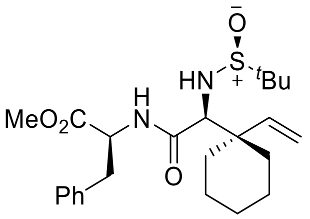

The compound **5aj** was synthesized according to **GP V** by using imine **4a** (34 mg, 0.1 mmol), **2t** (0.25 mmol, 2.5 equiv), and 4Å molecular sieves at room temperature. Column chromatography on silica using 20% ethylacetate in hexane as eluent provided the product **5aj** as colourless oil (34 mg, 73%). **^{1}H NMR** (500 MHz, $CDCl_3$): δ = 7.30 (t, *J* = 7.3 Hz, 1H), 7.26 – 7.22 (m, 2H), 7.16 (d, *J* = 7.3 Hz, 2H), 6.22 (d, *J* = 7.0 Hz, 1H), 5.58 (dd, *J* = 17.9, 11.0 Hz, 1H), 5.21 (d, *J* = 11.0 Hz, 1H), 5.04 (d, *J* = 17.8 Hz, 1H), 4.85 (q, *J* = 5.9 Hz, 1H), 4.40 (d, *J* = 8.4 Hz, 1H), 3.71 (s, 3H), 3.41 (d, *J* = 8.5 Hz, 1H), 3.17 (dd, *J* = 13.9, 5.1 Hz, 1H), 3.09 (dd, *J* = 13.8, 6.2 Hz, 1H), 1.73 – 1.68 (m, 1H), 1.54 – 1.45 (m, 4H), 1.41 – 1.31 (m, 5H), 1.28 (s, 9H) ppm. **^{13}C NMR** (126 MHz, $CDCl_3$): δ = 171.80, 170.50, 142.03, 135.85, 129.68, 128.82, 127.26, 117.12, 56.84, 53.51, 52.32, 44.64, 37.79, 33.52, 31.31, 26.25, 23.14, 22.17, 21.87 ppm. **IR (film):** ν_{max} = 3261, 2926, 1748, 1673, 1456, 1052 cm^{-1}. **HRMS-ESI:** calculated for $C_{24}H_{37}N_2O_4S$ $[M+H]^+$ 449.2469; found 449.2489 . **[α]$_D$^{25}:** +63.25 (0.152, $CHCl_3$).

Methyl ((2*S*)-2-(((*S*)-*tert*-butylsulfinyl)amino)-2-((*S*)-cyclohex-2-en-1-yl)acetyl)-*L*-phenylalaninate (5ak):

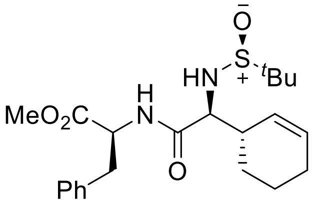

The compound **5ak** was synthesized according to **GP V** by using imine **4a** (34 mg, 0.1 mmol), **2aa** (0.25 mmol, 2.5 equiv), and 4Å molecular sieves at 60 °C. Column chromatography on silica using 25% ethylacetate in hexane as eluent provided the product **5ak** as colourless liquid (10 mg, 28%). **^{1}H NMR** (500 MHz, $CDCl_3$): δ = 7.43 (d, *J* = 7.8 Hz, 1H), 7.29 (t, *J* = 7.3 Hz, 2H), 7.23 (t, *J* = 7.2 Hz, 1H), 7.19 (d, *J* = 7.0 Hz, 2H), 5.78 – 5.75 (m, 1H), 5.02 (d, *J* = 10.2 Hz, 1H), 4.85 (td, *J* = 8.0, 5.7 Hz, 1H), 3.82 (d, *J* = 8.8 Hz, 1H), 3.73 (s, 3H), 3.67 (dd, *J* = 8.8, 3.8 Hz, 1H), 3.22 (dd, *J* = 13.9, 5.6 Hz, 1H), 3.03 (dd, *J* = 13.9, 8.0 Hz, 1H), 2.92 – 2.84 (m, 1H), 1.99 – 1.92 (m, 1H), 1.91 – 1.86 (m, 1H), 1.76 – 1.68 (m, 2H), 1.66 – 1.57 (m, 2H), 1.25 (s, 9H) ppm. **^{13}C NMR** (126 MHz, $CDCl_3$): δ = 171.83, 171.59, 136.51, 132.05, 129.54, 128.69, 127.12, 125.80, 63.17, 56.88, 53.86, 52.44, 39.04, 37.97, 25.83, 25.16, 22.91,

21.83 ppm. **IR (film):** ν_{max} = 3266, 2925, 1745, 1667, 1205, 1050 cm^{-1}. **HRMS-ESI:** calculated for $C_{22}H_{32}N_2O_4SNa$ [M+Na]$^+$ 443.1980; found 443.1982. **[α]$_D$^{25}**: -10.0 (c 0.12, $CHCl_3$).

Methyl ((2*S*)-2-(((*S*)-*tert*-butylsulfinyl)amino)-2-((*R*)-2-methylenecyclohexyl)acetyl)-*L*-phenylalaninate (5al):

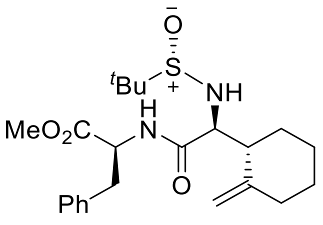

The compound **5al** was synthesized according to **GP V** by using imine **4a** (34 mg, 0.1 mmol), **2ab** (0.25 mmol, 2.5 equiv) and 4Å molecular sieves at room temperature. Column chromatography on silica using 24% ethylacetate in hexane as eluent provided the product **5al** as colourless oil (23 mg, 53%). **^{1}H NMR** (500 MHz, $CDCl_3$): δ = 7.28 (t, *J* = 7.3 Hz, 2H), 7.23 – 7.19 (m, 3H), 7.17 (d, *J* = 7.6 Hz, 1H), 4.79 (q, *J* = 7.3 Hz, 1H), 4.66 (s, 1H), 4.54 (s, 1H), 4.04 (dd, *J* = 9.9, 5.9 Hz, 1H), 3.81 (d, *J* = 9.8 Hz, 1H), 3.71 (s, 3H), 3.20 (dd, *J* = 13.8, 5.7 Hz, 1H), 3.05 (dd, *J* = 13.8, 7.2 Hz, 1H), 2.67 – 2.63 (m, 1H), 2.21 – 2.16 (m, 1H), 2.10 – 2.04 (m, 1H), 1.60 – 1.56 (m, 2H), 1.46 – 1.42 (m, 2H), 1.40 – 1.35 (m, 2H), 1.26 (s, 9H) ppm. **^{13}C NMR** (126 MHz, $CDCl_3$): δ = 172.06, 171.79, 148.22, 136.47, 129.67, 128.63, 127.07, 108.58, 60.73, 56.94, 53.88, 52.38, 45.73, 37.99, 35.09, 28.14, 27.92, 23.94, 22.96 ppm. **IR (film):** ν_{max} = 3263, 2925, 1749, 1641, 1556, 1058 cm^{-1}. **HRMS-ESI:** calculated for $C_{23}H_{35}N_2O_4S$ [M+H]$^+$ 435.2318; found 435.2332. **[α]$_D$^{25}**: +61.16 (c 0.233, $CHCl_3$).

Methyl ((2*S*,3*R*)-2-(((*S*)-*tert*-butylsulfinyl)amino)-3-ethyl-4-methylpent-4-enoyl)-*L*-phenylalaninate (5am):

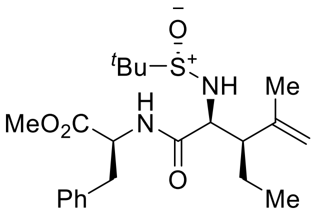

The compound **5am** was synthesized according to **GP V** by using imine **4a** (34 mg, 0.1 mmol), **2ac** (0.25 mmol, 2.5 equiv), and 4Å molecular sieves at room temperature. Column chromatography on silica using 24% ethylacetate in hexane as eluent provided the product **5am** as white solid (36 mg, 85%). **^{1}H NMR** (500 MHz, $CDCl_3$): δ = 7.28 (t, *J* = 7.3 Hz, 2H), 7.23 – 7.19 (m, 3H), 7.07 (d, *J* = 7.5 Hz, 1H), 4.86 (s, 1H), 4.78 (q, *J* = 7.1 Hz, 1H), 4.72 (s, 1H), 3.90 (d, *J* = 9.3 Hz, 1H), 3.71 – 3.68 (m, 4H), 3.17 (dd, *J* = 13.8, 5.9 Hz, 1H), 3.06 (dd, *J* = 13.8, 7.0 Hz, 1H), 2.40 (ddd, *J* = 10.2, 6.0, 3.7 Hz, 1H), 1.73 – 1.70 (m, 1H), 1.63 (s, 3H), 1.42 – 1.36 (m, 1H), 1.32 – 1.27 (m, 1H), 1.24 (s, 9H), 0.77 (t, *J* = 7.3 Hz, 3H) ppm. **^{13}C NMR** (126 MHz, $CDCl_3$): δ = 171.91, 171.75, 143.83, 136.36, 129.60, 128.64, 127.10, 114.00, 62.54, 56.89, 53.87, 52.35, 51.38, 37.95, 22.89, 21.30, 21.26, 11.85 ppm. **IR (film):** ν_{max} = 3273, 2927, 1748, 1647, 1248, 1050 cm^{-1}. **HRMS-ESI:** calculated for $C_{22}H_{35}N_2O_4S$ [M+H]$^+$ 423.2312; found 423.2314. **[α]$_D$^{25}**: +57.3 (c 0.34, $CHCl_3$).

Methyl ((2*S*,3*S*)-2-(((*S*)-*tert*-butylsulfinyl)amino)-3-(4-chlorophenyl)-4-methylpent-4-enoyl)-*L*-phenylalaninate (5an):

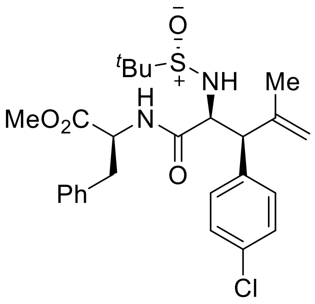

The compound **5an** was synthesized according to **GP V** by using imine **4a** (34 mg, 0.1 mmol), **2ad** (0.25 mmol, 2.5 equiv) and 4Å molecular sieves at room temperature. Column chromatography on silica using 23% ethylacetate in hexane as eluent provided the product **5an** as foamy solid (39 mg, 78%). **^{1}H NMR** (500 MHz, $CDCl_3$): δ = 7.71 (d, *J* = 7.3 Hz, 1H), 7.19 (d, *J* = 8.1 Hz, 2H), 7.17 – 7.14 (m, 3H), 7.05 (d, *J* = 8.3 Hz, 2H), 7.01 (d, *J* = 7.0 Hz, 2H), 4.97 (s, 1H), 4.93 (s, 1H), 4.72 (q, *J* = 7.6 Hz, 1H), 4.38 (dd, *J* = 10.4, 4.7 Hz, 1H), 4.16 (d, *J* = 4.6 Hz, 1H), 3.72 (s, 3H), 3.22 (d, *J* = 10.4 Hz, 1H), 3.12 (dd, *J* = 14.0, 5.5 Hz, 1H), 2.97 (dd, *J* = 13.9, 7.9 Hz, 1H), 1.60 (s, 3H), 1.08 (s, 9H) ppm. **^{13}C NMR** (126 MHz, $CDCl_3$): δ = 171.67, 171.45, 144.75, 136.40, 133.19, 131.11, 129.34, 128.59, 128.48, 127.03, 112.26, 62.34, 57.10, 54.29, 52.42, 52.33, 37.62, 22.93, 22.68 ppm. **IR (film): ν_{max}** = 3268, 2926, 1744, 1663, 1218, 1051 cm^{-1}. **HRMS-ESI:** calculated for $C_{26}H_{34}ClN_2O_4S$ $[M+H]^+$ 505.1922; found 505.1949. **$[\alpha]_D^{25}$**: -76.7 (c 0.16, $CHCl_3$).

Methyl ((2*S*)-2-(((*S*)-*tert*-butylsulfinyl)amino)-4-phenylpent-4-enoyl)-*L*-phenylalaninate (5ao):

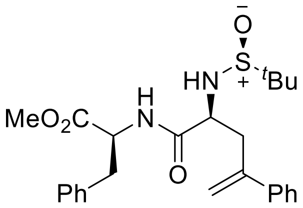

The compound **5ao** was synthesized according to **GP V** by using imine **4a** (34 mg, 0.1 mmol), **2v** (0.25 mmol, 2.5 equiv) and 4Å molecular sieves at room temperature. Column chromatography on silica using 27% ethylacetate in hexane as eluent provided the product **5ao** as gummy solid (36 mg, 79%). **^{1}H NMR** (500 MHz, $CDCl_3$): δ = 7.43 (d, *J* = 7.3 Hz, 1H), 7.29 – 7.26 (m, 3H), 7.26 – 7.20 (m, 5H), 7.14 – 7.12 (m, 2H), 5.25 (s, 1H), 5.02 (s, 1H), 4.64 (td, *J* = 7.7, 5.7 Hz, 1H), 3.83 (td, *J* = 8.6, 5.0 Hz, 1H), 3.66 (s, 3H), 3.60 (d, *J* = 9.0 Hz, 1H), 3.18 (dd, *J* = 15.0, 5.4 Hz, 1H), 3.10 (dd, *J* = 13.9, 5.6 Hz, 1H), 2.94 (dd, *J* = 13.9, 7.9 Hz, 1H), 2.66 (dd, *J* = 14.7, 8.3 Hz, 1H), 1.09 (s, 9H) ppm. **^{13}C NMR** (126 MHz, $CDCl_3$): δ = 171.97, 171.77, 144.38, 139.98, 136.52, 129.53, 128.79, 128.65, 128.09, 127.11, 126.46, 116.67, 58.93, 56.73, 54.21, 52.41, 39.59, 37.77, 22.62 ppm. **IR (film): ν_{max}** = 3273, 2927, 1743, 1668, 1213, 1045 cm^{-1}. **HRMS-ESI:** calculated for $C_{25}H_{33}N_2O_4S$ $[M+H]^+$ 456.2161; found 456.2160. 1999. **$[\alpha]_D^{25}$**: +86.75, (c 0.366, $CHCl_3$).

Methyl (*R*)-4-((*S*)-1-(((*S*)-*tert*-butylsulfinyl)amino)-2-(((*S*)-1-methoxy-1-oxo-3-phenylpropan-2-yl)amino)-2-oxoethyl)-4-methylhex-5-enoate (5ap):

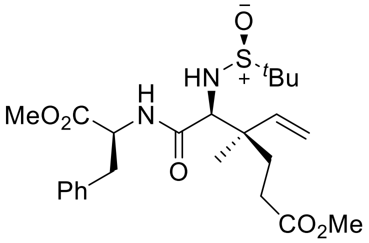

The compound **5ap** was synthesized according to **GP V** by using imine **4a** (34 mg, 0.1 mmol), **2w** (0.25 mmol, 2.5 equiv) and 4Å molecular sieves at room temperature. Column chromatography on silica using 23% ethylacetate in hexane as eluent provided the product **5ap** as colourless oil (29 mg, 62%). **^{1}H NMR** (500 MHz, $CDCl_3$): δ = 7.30 (t, *J* = 7.4 Hz, 2H), 7.23 (t, *J* = 7.0 Hz, 1H), 7.15 (d, *J* = 7.5 Hz, 2H), 6.31 (d, *J* = 7.3 Hz, 1H), 5.69 (dd, *J* = 17.5, 10.8 Hz, 1H), 5.08 (d, *J* = 10.8 Hz, 1H), 5.01 (d, *J* = 17.6 Hz, 1H), 4.82 (q, *J* = 6.0 Hz, 1H), 4.54 (d, *J* = 8.1 Hz, 1H), 3.70 (s, 3H), 3.63 (s, 3H), 3.48 (d, *J* = 8.1 Hz, 1H), 3.16 (dd, *J* = 13.8, 4.8 Hz, 1H), 3.08 (dd, *J* = 13.8, 6.2 Hz, 1H), 2.19 (t, *J* = 8.1 Hz, 2H), 1.90 – 1.83 (m, 1H), 1.76 – 1.70 (m, 1H), 1.28 (s, 9H), 0.96 (s, 3H) ppm. **^{13}C NMR** (126 MHz, $CDCl_3$): δ = 174.14, 171.61, 170.22, 141.62, 135.74, 129.67, 128.83, 127.29, 116.82, 66.04, 56.84, 53.58, 52.33, 51.71, 44.46, 37.70, 32.57, 29.23, 23.08, 17.51 ppm. **IR (film): ν_{max}** = 3273, 2925, 1732, 1632, 1201, 1052 cm^{-1}. **HRMS-ESI:** calculated for $C_{24}H_{37}N_2O_6S$ $[M+H]^+$ 481.2372; found 481.2379. **$[\alpha]_D^{25}$**: +64.15 (0.125, $CHCl_3$).

Methyl ((*2S,3R*)-2-(((*S*)-*tert*-butylsulfinyl)amino)-6-((4-methoxybenzyl)oxy)-3-vinylhexanoyl)-*L*-phenylalaninate (5aq):

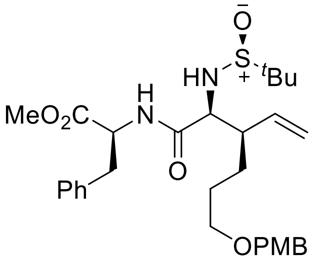

The compound **5aq** was synthesized according to **GP V** by using imine **4a** (34 mg, 0.1 mmol), **2y** (0.25 mmol, 2.5 equiv) and 4Å molecular sieves at room temperature. Column chromatography on silica using 30% ethylacetate in hexane as eluent provided the product **5aq** as colourless oil (45 mg, 82%). **^{1}H NMR** (500 MHz, $CDCl_3$): δ = 7.27 – 7.21 (m, 6H), 7.19 – 7.17 (m, 2H), 6.86 (d, *J* = 8.6 Hz, 2H), 5.50 (ddd, *J* = 16.2, 10.9, 9.0 Hz, 1H), 5.05 – 5.02 (m, 2H), 4.79 (q, *J* = 7.5 Hz, 1H), 4.39 (s, 2H), 3.83 – 3.79 (m, 4H), 3.74 (dd, *J* = 9.5, 4.4 Hz, 1H), 3.70 (s, 3H), 3.39 – 3.33 (m, 2H), 3.18 (dd, *J* = 13.9, 5.8 Hz, 1H), 3.03 (dd, *J* = 13.9, 7.5 Hz, 1H), 2.54 – 2.49 (m, 1H), 1.78 – 1.66 (m, 1H), 1.63 – 1.54 (m, 1H), 1.45 – 1.34 (m, 2H), 1.23 (s, 9H) ppm. **^{13}C NMR** (126 MHz, $CDCl_3$): δ = 171.80, 171.15, 159.33, 138.13, 136.42, 130.89, 129.55, 129.34, 128.65, 127.13, 117.87, 113.94, 72.62, 69.91, 63.58, 56.86, 55.43, 53.82, 52.39, 47.47, 37.88, 27.60, 26.28, 22.85 ppm. **IR (film): ν_{max}** = 3266, 2927, 1745, 1672, 1513, 1240, 1045 cm^{-1}. **HRMS-ESI:** calculated for $C_{30}H_{42}N_2O_6SNa$ $[M+Na]^+$ 581.2661; found 581.2664. **$[\alpha]_D^{25}$**: +25.0 (c 0.3, $CHCl_3$).

Methyl ((2*S*,3*R*)-3-(2-((*N*-benzyl-4-methylphenyl)sulfonamido)ethyl)-2-(((*S*)-*tert*-butylsulfinyl)amino)pent-4-enoyl)-*L*-phenylalaninate (5ar):

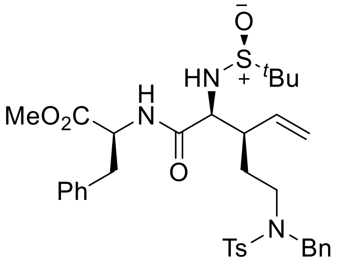

The compound **5ar** was synthesized according to **GP V** by using imine **4a** (34 mg, 0.1 mmol), **2z** (0.25 mmol, 2.5 equiv) and 4Å molecular sieves at room temperature. Column chromatography on silica using 35% ethylacetate in hexane as eluent provided the product **5ar** as colourless oil (52 mg, 79%). **^{1}H NMR** (500 MHz, $CDCl_3$): δ = 7.67 (d, *J* = 8.2 Hz, 2H), 7.29 – 7.26 (m, 4H), 7.26 – 7.21 (m, 7H), 7.17 (d, *J* = 6.9 Hz, 2H), 5.30 (dt, *J* = 17.2, 9.8 Hz, 1H), 4.97 (d, *J* = 10.2 Hz, 1H), 4.90 (d, *J* = 17.1 Hz, 1H), 4.71 (q, *J* = 7.4 Hz, 1H), 4.35 (d, *J* = 14.8 Hz, 1H), 4.11 (d, *J* = 14.9 Hz, 1H), 3.73 – 3.69 (m, 4H), 3.55 (dd, *J* = 9.7, 4.3 Hz, 1H), 3.14 (dd, *J* = 13.9, 5.9 Hz, 1H), 3.04 – 2.95 (m, 3H), 2.42 (s, 3H), 2.34 (tt, *J* = 9.2, 4.2 Hz, 1H), 1.56 – 1.50 (m, 1H), 1.20 (s, 9H) ppm. **^{13}C NMR** (126 MHz, $CDCl_3$): δ = 171.77, 170.92, 143.38, 137.19, 136.93, 136.75, 136.37, 129.84, 129.51, 128.69, 128.60, 127.95, 127.37, 127.19, 118.42, 63.02, 56.86, 53.97, 52.53, 52.40, 46.77, 44.71, 37.79, 28.71, 22.85, 21.62 ppm. **IR (film): ν_{max}** = 3263, 2925, 1744, 1673, 1137, 1052 cm^{-1}. **HRMS-ESI:** calculated for $C_{35}H_{46}N_3O_6S_2$ $[M+H]^+$ 668.2828; found 668.2850. **$[\alpha]_D^{25}$**: +21.2 (c 0.24, $CHCl_3$).

Methyl ((2*S*,3*R*)-2-(((*S*)-*tert*-butylsulfinyl)amino)-3-methylpent-4-enoyl)-*L*-phenylalaninate (5as):

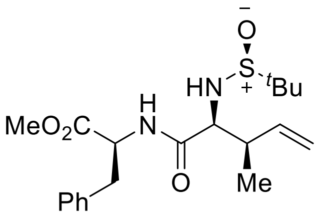

The compound **5as** was synthesized according to **GP V** by using imine **4a** (34 mg, 0.1 mmol), **2ae** (0.25 mmol, 2.5 equiv) and 4Å molecular sieves at 60 °C. Column chromatography on silica using 24% ethylacetate in hexane as eluent provided the product **5as** as colourless oil (27 mg, 68%). **^{1}H NMR** (500 MHz, $CDCl_3$): δ = 7.30 – 7.27 (m, 3H), 7.23 (d, *J* = 7.1 Hz, 1H), 7.19 (d, *J* = 7.6 Hz, 2H), 5.70 (ddd, *J* = 17.4, 10.4, 7.1 Hz, 1H), 5.05 – 5.01 (m, 2H), 4.81 (q, *J* = 7.1 Hz, 1H), 3.82 – 3.75 (m, 2H), 3.72 (s, 3H), 3.20 (dd, *J* = 13.9, 5.8 Hz, 1H), 3.05 (dd, *J* = 13.9, 7.5 Hz, 1H), 2.81 – 2.76 (m, 1H), 1.24 (s, 9H), 0.85 (d, *J* = 6.8 Hz, 3H) ppm. **^{13}C NMR** (126 MHz, $CDCl_3$): δ = 171.84, 171.22, 140.06, 136.38, 129.52, 128.65, 127.14, 115.77, 63.79, 56.93, 53.84, 52.42, 40.57, 37.83, 22.85, 14.05 ppm. **IR (film): ν_{max}** = 3261, 2961, 1745, 1672, 1209, 1048 cm^{-1}. **HRMS-ESI:** calculated for $C_{20}H_{30}N_2O_4SNa$ $[M+Na]^+$ 417.1824; found 417.1822. **$[\alpha]_D^{25}$**: +45.11 (c 0.266, $CHCl_3$).

Methyl ((2*S*,3*S*)-2-(((*S*)-*tert*-butylsulfinyl)amino)-3-methylpent-4-enoyl)-*L*-phenylalaninate (5at):

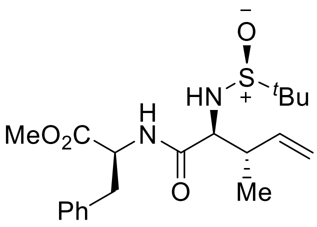

The compound **5at** was synthesized according to **GP V** by using imine **4a** (34 mg, 0.1 mmol), **2af** (0.25 mmol, 2.5 equiv) and 4Å molecular sieves at 60 °C. Column chromatography on silica using 20% ethylacetate in hexane as eluent provided the product **5at** as colourless oil (20 mg, 51%). **^{1}H NMR** (500 MHz, $CDCl_3$) δ = 7.30 – 7.23 (m, 4H), 7.19 (d, *J* = 7.3 Hz, 2H), 5.69 (ddd, *J* = 17.4, 10.5, 7.1 Hz, 1H), 5.04 – 5.01 (m, 2H), 4.82 (q, *J* = 7.0 Hz, 1H), 3.81 (d, *J* = 9.0 Hz, 1H), 3.76 (d, *J* = 8.9 Hz, 1H), 3.73 (s, 3H), 3.21 (dd, *J* = 13.9, 5.6 Hz, 1H), 3.05 (dd, *J* = 13.9, 7.6 Hz, 1H), 2.80 – 2.76 (m, 1H), 1.24 (s, 9H), 0.85 (d, *J* = 6.9 Hz, 3H) ppm. **^{13}C NMR** (126 MHz, $CDCl_3$): δ = 171.83, 171.22, 140.08, 136.41, 129.53, 128.73, 128.66, 127.14, 115.77, 63.83, 56.94, 53.84, 52.41, 40.58, 37.87, 22.87, 14.11 ppm. **IR (film): ν_{max}** = 3267, 2926, 1744, 1661, 1543, 1212, 1050 cm^{-1}. **HRMS-ESI:** calculated for $C_{20}H_{30}N_2O_4SNa$ $[M+Na]^+$ 417.1824; found 417.1825. **$[\alpha]_D^{25}$**: +33.33 (c 0.33, $CHCl_3$).

Methyl ((2*S*,3*R*)-2-(((*S*)-*tert*-butylsulfinyl)amino)-3,7-dimethyl-3-vinyloct-6-enoyl)-*L*-tyrosinate (5ba):

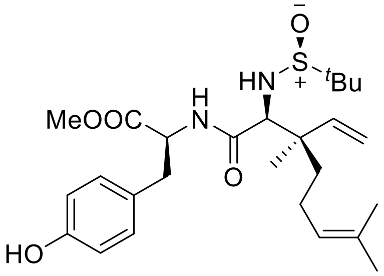

The compound **5ba** was synthesized according to **GP V** by using imine **4b** (34 mg, 0.1 mmol), geranyl boronic acid **2a** (0.25 mmol, 2.5 equiv), and 4Å molecular sieves (100 mg) at room temperature. Column chromatography on silica using 30% ethylacetate in hexane as eluent provided the product **5ba** as white solid (35 mg, 87%). **^{1}H NMR** (500 MHz, $CDCl_3$): δ = 6.93 (d, *J* = 8.3 Hz, 2H), 6.73 (d, *J* = 8.4 Hz, 2H), 6.25 (d, *J* = 7.4 Hz, 1H), 5.77 (dd, *J* = 17.5, 10.8 Hz, 1H), 5.12 (d, *J* = 10.9 Hz, 1H), 5.03 – 4.99 (m, 2H), 4.73 (dt, *J* = 7.3, 5.5 Hz, 1H), 4.64 (d, *J* = 8.0 Hz, 1H), 3.68 (s, 3H), 3.48 (d, *J* = 8.1 Hz, 1H), 3.01 (dd, *J* = 14.0, 4.9 Hz, 1H), 2.95 (dd, *J* = 14.0, 6.1 Hz, 1H), 1.84 (q, *J* = 7.7 Hz, 2H), 1.65 (s, 3H), 1.54 (s, 3H), 1.49 – 1.43 (m, 1H), 1.42 – 1.36 (m, 1H), 1.29 (s, 9H), 0.99 (s, 3H) ppm. **^{13}C NMR** (126 MHz, $CDCl_3$): δ = 171.83, 170.41, 155.73, 142.34, 131.68, 130.68, 126.93, 124.40, 116.07, 115.80, 65.97, 56.93, 53.78, 52.28, 44.82, 38.04, 36.96, 25.77, 23.13, 22.70, 17.95, 17.80 ppm. **IR (film): ν_{max}** = 3367, 2956, 1762, 1682, 1527, 1059 cm^{-1}. **HRMS-ESI:** calculated for $C_{26}H_{41}N_2O_5S$ $[M+H]^+$ 493.2731; found 491.2758. **$[\alpha]_D^{25}$**: +84.30 (c 0.433, $CHCl_3$).

Methyl ((*2S,3R*)-2-(((*S*)-*tert*-butylsulfinyl)amino)-3,7-dimethyl-3-vinyloct-6-enoyl)-*L*-tryptophanate (5ca):

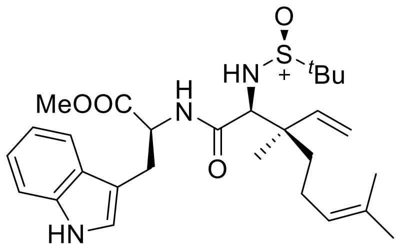

The compound **5ca** was synthesized according to **GP V** by using imine **4c** (38 mg, 0.1 mmol), geranyl boronic acid **2a** (0.25 mmol, 2.5 equiv), and 4Å molecular sieves (100 mg) at room temperature. Column chromatography on silica using 25% ethylacetate in hexane as eluent provided the product **5ca** as gummy solid (43 mg, 84%). **^{1}H NMR** (400 MHz, $CDCl_3$): δ = 8.35 (br s, 1H), 7.53 (d, *J* = 7.8 Hz, 1H), 7.34 (d, *J* = 8.0 Hz, 1H), 7.19 – 7.14 (m, 2H), 7.10 (d, *J* = 7.2 Hz, 2H), 6.28 (d, *J* = 7.4 Hz, 1H), 5.69 (dd, *J* = 17.4, 10.9 Hz, 1H), 5.00 – 4.84 (m, 4H), 4.60 (d, *J* = 8.2 Hz, 1H), 3.66 (s, 3H), 3.42 (d, *J* = 8.2 Hz, 1H), 3.33 – 3.27 (m, 2H), 1.83 – 1.77 (m, 2H), 1.64 (s, 3H), 1.53 (s, 3H), 1.44 – 1.33 (m, 2H), 1.29 (s, 9H), 0.97 (s, 3H) ppm. **^{13}C NMR** (101 MHz, $CDCl_3$): δ = 171.99, 170.41, 142.20, 136.22, 131.61, 127.70, 124.42, 124.35, 122.07, 119.68, 118.65, 115.83, 111.37, 109.27, 66.18, 56.77, 53.08, 52.42, 44.76, 37.92, 27.26, 25.78, 23.13, 22.65, 17.94, 17.79 ppm. **IR (film): ν_{max}** = 3258, 2956, 1744, 1684, 1482, 1052 cm^{-1}. **HRMS-ESI:** calculated for $C_{28}H_{42}N_3O_4S$ $[M+H]^+$ 516.2891; found 516.2896. **$[\alpha]_D^{25}$**: +143.0 (c 0.5, $CHCl_3$).

Methyl ((*2S,3R*)-2-(((*S*)-*tert*-butylsulfinyl)amino)-3,7-dimethyl-3-vinyloct-6-enoyl)-*L*-methioninate (5da):

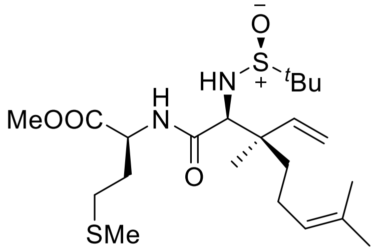

The compound **5da** was synthesized according to **GP V** by using imine **4d** (32 mg, 0.1 mmol), geranyl boronic acid **2a** (0.25 mmol, 2.5 equiv), and 4Å molecular sieves (100 mg) at room temperature. Column chromatography on silica using 25% ethylacetate in hexane as eluent provided the product **5da** as colourless oil (37 mg, 82%). **^{1}H NMR** (400 MHz, $CDCl_3$): δ = 6.90 (d, *J* = 6.5 Hz, 1H), 5.87 (dd, *J* = 17.5, 10.8 Hz, 1H), 5.18 (d, *J* = 10.8 Hz, 1H), 5.05 – 5.01 (m, 2H), 4.63 (td, *J* = 7.2, 5.2 Hz, 1H), 4.43 (d, *J* = 8.6 Hz, 1H), 3.72 (s, 3H), 3.49 (d, *J* = 8.6 Hz, 1H), 2.55 (t, *J* = 7.1 Hz, 2H), 2.17 – 2.09 (m, 4H), 2.03 – 1.96 (m, 1H), 1.90 – 1.83 (m, 3H), 1.65 (s, 3H), 1.55 (s, 3H), 1.51 – 1.45 (m, 1H), 1.25 (s, 9H), 1.04 (s, 3H). ppm. **^{13}C NMR** (101 MHz, $CDCl_3$): δ = 172.15, 171.02, 142.25, 131.58, 124.49, 115.68, 66.22, 56.66, 52.44, 51.94, 44.68, 37.80, 31.36, 29.97, 25.78, 23.04, 22.74, 18.37, 17.79, 15.58 ppm. **IR (film): ν_{max}** = 3269, 2965, 1748 1671, 1551,

1053 cm^{-1}. **HRMS-ESI:** calculated for $C_{22}H_{41}N_2O_4S_2$ $[M+H]^+$ 461.2502; found 461.2513. **$[\alpha]_D^{25}$**: +69.82 (c 0.333, $CHCl_3$).

Methyl ((*2S,3R*)-2-(((*S*)-*tert*-butylsulfinyl)amino)-3,7-dimethyl-3-vinyloct-6-enoyl)-*L*-valinate (5ea):

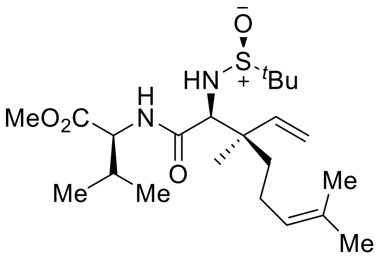

The compound **5ea** was synthesized according to **GP V** on 0.3 mmol scale by using imine **4e**, geranyl boronic acid **2a** (0.25 mmol, 2.5 equiv), and 4Å molecular sieves (100 mg) at room temperature. Column chromatography on silica using 20% ethylacetate in hexane as eluent provided the product **5ea** as colourless oil (77 mg, 60% over four steps from parent peptide). **^{1}H NMR** (400 MHz, $CDCl_3$): δ = 6.56 (d, *J* = 7.4 Hz, 1H), 5.86 (dd, *J* = 17.5, 10.8 Hz, 1H), 5.18 (d, *J* = 10.8 Hz, 1H), 5.06 – 5.01 (m, 2H), 4.47 (d, *J* = 8.3 Hz, 1H), 4.40 (dd, *J* = 8.0, 5.0 Hz, 1H), 3.70 (s, 3H), 3.55 (d, *J* = 8.3 Hz, 1H), 2.16 – 2.08 (m, 1H), 1.89 – 1.83 (m, 2H), 1.64 (s, 3H), 1.54 (s, 3H), 1.50 – 1.47 (m, 1H), 1.43 – 1.37 (m, 1H), 1.25 (s, 9H), 1.02 (s, 3H), 0.93 (d, *J* = 7.0 Hz, 3H), 0.91 (d, *J* = 7.0 Hz, 3H). **^{13}C NMR** (101 MHz, $CDCl_3$): δ = 172.06, 171.06, 142.41, 131.55, 124.47, 115.67, 66.02, 57.80, 56.63, 52.04, 44.74, 37.88, 31.26, 25.77, 23.03, 22.71, 18.91, 18.15, 18.11, 17.77 ppm. **IR (film): ν_{max}** = 3264, 2962, 1751, 1667, 1548, 1042 cm^{-1}. **HRMS-ESI:** calculated for $C_{22}H_{41}N_2O_4S$ $[M+H]^+$ 429.2787; found 429.2804. **$[\alpha]_D^{25}$**: +55.45 (c 1.33, $CHCl_3$).

Methyl ((*2S,3R*)-2-(((*S*)-*tert*-butylsulfinyl)amino)-3,7-dimethyl-3-vinyloct-6-enoyl)-*L*-leucinate (5fa):

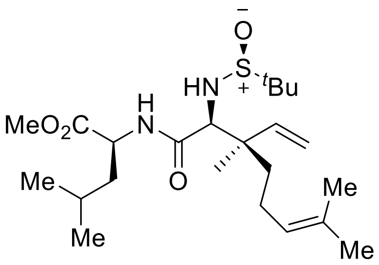

The compound **5fa** was synthesized according to **GP V** on 0.3 mmol scale from imine **4f**, geranyl boronic acid **2a** (0.25 mmol, 2.5 equiv), and 4Å molecular sieves (100 mg) at room temperature. Column chromatography on silica using 22% ethylacetate in hexane as eluent provided the product **5fa** as colourless oil (102 mg, 76% over four steps) along with its minor diastereomer (11 mg, 8%); *dr*: 9:1. **^{1}H NMR** (400 MHz, $CDCl_3$): δ = 6.67 (d, *J* = 7.2 Hz, 1H), 5.85 (dd, *J* = 17.5, 10.8 Hz, 1H), 5.17 (d, *J* = 10.9 Hz, 1H), 5.05 – 5.01 (m, 2H), 4.50 – 4.42 (m, 2H), 3.68 (s, 3H), 3.53 (d, *J* = 8.0 Hz, 1H), 1.87 – 1.82 (m, 1H), 1.66 – 1.62 (m, 4H), 1.59 – 1.55 (m, 1H), 1.53 (s, 3H), 1.49 – 1.39 (m, 3H), 1.24 (s, 9H), 1.01 (s, 3H), 0.90 (d, *J* = 4.6 Hz, 3H), 0.89 (d, *J* = 4.6 Hz, 3H) ppm. **^{13}C NMR** (101 MHz, $CDCl_3$): δ = 173.02, 170.79, 142.47, 131.47, 124.50, 115.62, 65.57, 56.63, 52.11, 51.44, 44.79, 41.20, 37.69, 25.75, 24.83, 23.01, 22.69, 22.63, 22.35, 18.17,

17.75 ppm. **IR (film):** ν_{max} = 3266, 2959, 1748, 1671, 1548, 1051 cm^{-1}. **HRMS-ESI:** calculated for $C_{23}H_{43}N_2O_4S$ [M+H]$^+$ 443.2938; found 443.2943. **[α]$_D$^{25}:** +63.13 (c 0.8, $CHCl_3$).

Methyl ((2*S*,3*R*)-2-(((*S*)-*tert*-butylsulfinyl)amino)-3,7-dimethyl-3-vinyloct-6-enoyl)-*L*-alloisoleucinate (5ga):

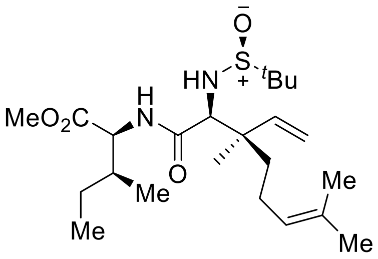

The compound **5ga** was synthesized according to **GP V** by using imine **4g** (30 mg, 0.1 mmol), geranyl boronic acid **2a** (0.25 mmol, 2.5 equiv), and 4Å molecular sieves (100 mg) at room temperature. Column chromatography on silica using 25% ethylacetate in hexane as eluent provided the product **5ga** as gummy solid (34 mg, 77%). **^{1}H NMR** (400 MHz, $CDCl_3$): δ = 6.25 (d, *J* = 8.5 Hz, 1H), 5.88 (dd, *J* = 17.6, 10.9 Hz, 1H), 5.22 (d, *J* = 10.7 Hz, 1H), 5.10 – 5.02 (m, 2H), 4.53 (dd, *J* = 7.9, 4.7 Hz, 1H), 4.48 (d, *J* = 8.4 Hz, 1H), 3.72 (s, 3H), 3.50 (d, *J* = 8.3 Hz, 1H), 1.92 – 1.84 (m, 2H), 1.66 (s, 3H), 1.56 (s, 3H), 1.52 – 1.47 (m, 1H), 1.45 – 1.36 (m, 3H), 1.26 (s, 9H), 1.23 – 1.15 (m, 1H), 1.03 (s, 3H), 0.92 – 0.89 (m, 6H) ppm. **^{13}C NMR** (126 MHz, $CDCl_3$): δ = 172.08, 170.93, 142.60, 131.67, 124.49, 115.88, 66.26, 56.90, 56.72, 52.07, 44.81, 38.13, 37.98, 25.79, 25.40, 23.10, 22.76, 18.21, 17.81, 15.64, 11.75 ppm. **IR (film):** ν_{max} = 3256, 2964, 1745, 1671, 1544, 1202, 1051 cm^{-1}. **HRMS-ESI:** calculated for $C_{23}H_{42}N_2O_4SNa$ [M+Na]$^+$ 465.2763; found 465.2759. **[α]$_D$^{25}:** +84.52 (c 0.253, $CHCl_3$).

Dimethyl ((2*S*,3*R*)-2-(((*S*)-*tert*-butylsulfinyl)amino)-3,7-dimethyl-3-vinyloct-6-enoyl)-*L*-glutamate (5ha):

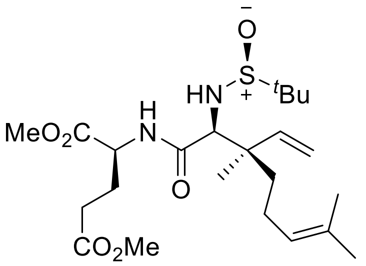

The compound **5ha** was synthesized according to **GP V** by using imine **4h**, geranyl boronic acid **2a** (0.25 mmol, 2.5 equiv), and 4Å molecular sieves (100 mg) at room temperature. Column chromatography on silica using 30% ethylacetate in hexane as eluent provided the product **5ha** as colourless oil (70 mg, 57% over four steps). **^{1}H NMR** (500 MHz, $CDCl_3$): δ = 6.79 (br s, 1H), 5.87 (dd, *J* = 17.5, 10.8 Hz, 1H), 5.19 (d, *J* = 10.8 Hz, 1H), 5.05 – 5.02 (m, 2H), 4.55 (q, *J* = 7.3 Hz, 1H), 4.43 (d, *J* = 8.7 Hz, 1H), 3.72 (s, 3H), 3.67 (s, 3H), 3.48 (d, *J* = 8.6 Hz, 1H), 2.48 – 2.44 (m, 2H), 2.22 – 2.15 (m, 1H), 2.01 – 1.96 (m, 1H), 1.90 – 1.83 (m, 2H), 1.66 (s, 3H), 1.56 (s, 3H), 1.52 – 1.43 (m, 2H), 1.25 (s, 9H), 1.04 (s, 3H) ppm. **^{13}C NMR** (126 MHz, $CDCl_3$): δ = 173.95, 172.02, 171.25, 142.26, 131.61, 124.52, 115.72, 66.40, 56.74, 52.45, 52.16, 51.94, 44.67, 37.89, 30.05, 29.83, 27.10, 25.77, 23.07, 22.76, 18.40, 17.78 ppm. **IR (film):** ν_{max} = 2924, 1739, 1668, 1336, 1205,

1045 cm^{-1}. **HRMS-ESI:** calculated for $C_{23}H_{40}N_2NaO_6S$ $[M+Na]^+$ 495.2505; found 495.2504. **[α]D^{25}**: +33.47 (c 0.366, $CHCl_3$).

Dimethyl ((*2S,3R*)-2-(((*S*)-*tert*-butylsulfinyl)amino)-3,7-dimethyl-3-vinyloct-6-enoyl)-*L*-aspartate (5ia):

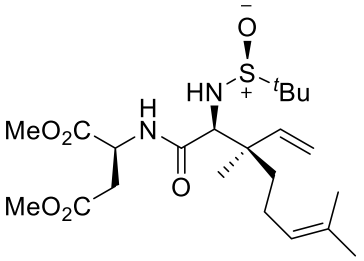

The compound **5ia** was synthesized according to **GP V** by using imine **4i**, geranyl boronic acid **2a** (0.25 mmol, 2.5 equiv), and 4Å molecular sieves (100 mg) at room temperature. Column chromatography on silica using 35% ethylacetate in hexane as eluent provided the product **5ia** as colourless oil (55 mg, 40% over four steps). **^{1}H NMR** (500 MHz, $CDCl_3$): δ = 6.91 (d, *J* = 8.3 Hz, 1H), 5.89 (dd, *J* = 17.5, 10.8 Hz, 1H), 5.24 (d, *J* = 10.9 Hz, 1H), 5.08 – 5.05 (m, 2H), 4.90 – 4.87 (m, 1H), 4.45 (d, *J* = 8.8 Hz, 1H), 3.72 (s, 3H), 3.68 (s, 3H), 3.46 (d, *J* = 8.8 Hz, 1H), 3.01 (dd, *J* = 17.4, 4.2 Hz, 1H), 2.83 (dd, *J* = 17.3, 4.6 Hz, 1H), 1.91 – 1.86 (m, 2H), 1.66 (s, 3H), 1.56 (s, 3H), 1.51 – 1.44 (m, 2H), 1.25 (s, 9H), 1.06 (s, 3H) ppm. **^{13}C NMR** (126 MHz, $CDCl_3$): δ = 172.07, 170.99, 170.96, 142.13, 131.63, 124.49, 116.00, 66.41, 56.74, 52.68, 52.17, 48.60, 44.65, 37.95, 35.78, 25.77, 23.05, 22.77, 18.51, 17.78 ppm. **IR (film): ν_{max}** = 3272, 2924, 1737, 1666, 1439, 1368, 1051 cm^{-1}. **HRMS-ESI:** calculated for $C_{22}H_{39}N_2O_6S$ $[M+H]^+$ 459.2523; found 459.2505. **[α]D^{25}**: +72.07 (c 0.333, $CHCl_3$).

Methyl ((*2S,3R*)-2-(((*S*)-*tert*-butylsulfinyl)amino)-3,7-dimethyl-3-vinyloct-6-enoyl)-*L*-leucyl-*L*-phenylalaninate (6a):

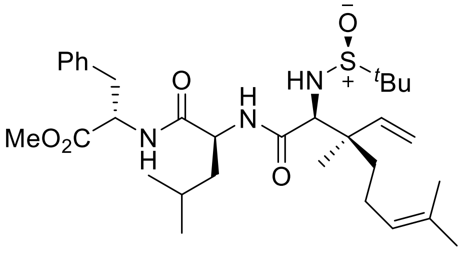

The compound **6a** was prepared according to **GP V** by using imine **4j** (45 mg, 0.1 mmol), geranyl boronic acid **2a** (0.25 mmol, 2.5 equiv), and 4 Å molecular sieves (100 mg) at room temperature. Column chromatography on silica using 28% ethylacetate in hexane as eluent provided the product **6a** as colourless oil (38 mg, 64%). **^{1}H NMR** (400 MHz, $CDCl_3$): δ = 7.30 – 7.22 (m, 3H), 7.15 – 7.13 (m, 2H), 6.78 (d, *J* = 7.8 Hz, 1H), 6.29 (d, *J* = 7.9 Hz, 1H), 5.81 (dd, *J* = 17.5, 10.8 Hz, 1H), 5.17 (d, *J* = 10.8 Hz, 1H), 5.06 – 5.01 (m, 2H), 4.79 (dt, *J* = 7.9, 6.3 Hz, 1H), 4.35 (td, *J* = 8.3, 5.7 Hz, 1H), 4.27 (d, *J* = 8.8 Hz, 1H), 3.68 (s, 3H), 3.50 (d, *J* = 8.8 Hz, 1H), 3.09 (dd, *J* = 6.3, 2.2 Hz, 2H), 1.91 – 1.82 (m, 2H), 1.63 (s, 3H), 1.62 – 1.57 (m, 2H), 1.54 (s, 3H), 1.52 – 1.45 (m, 2H), 1.42 – 1.35 (m, 1H), 1.26 (s, 9H), 1.03 (s, 3H), 0.89 (d, *J* = 6.0 Hz, 3H), 0.86 (d, *J* = 6.0 Hz, 3H) ppm. **^{13}C NMR** (101 MHz, $CDCl_3$): δ = 171.83,

171.44, 170.74, 142.41, 136.25, 131.76, 129.42, 128.69, 127.16, 124.32, 116.11, 66.38, 56.87, 53.52, 52.55, 52.36, 44.72, 41.23, 38.15, 37.90, 25.77, 24.82, 22.99, 22.87, 22.72, 22.17, 18.53, 17.79 ppm. **IR (film):** ν_{max} = 3289, 2959, 1739, 1646, 1524, 1055 cm^{-1}. **HRMS-ESI:** calculated for $C_{32}H_{52}N_3O_5S$ $[M+H]^+$ 590.3622; found 590.3647. **[α]D^{25}**: +80.7 (c 0.24, CHCl$_3$).

Methyl ((2*S*,3*R*)-2-(((*S*)-*tert*-butylsulfinyl)amino)-3,7-dimethyl-3-vinyloct-6-enoyl)-*L*-valyl-*L*-alloisoleucinate (6b):

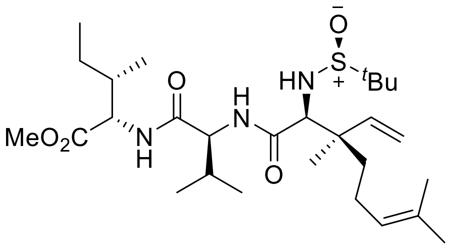

The compound **6b** was synthesized according to **GP V** by using imine **4k** (40 mg, 0.1 mmol), geranyl boronic acid **2a** (0.25 mmol, 2.5 equiv), 4 Å molecular sieves (100 mg) at room temperature. Column chromatography on silica using 30% ethylacetate in hexane as eluent provided the product **6b** as a gummy oil (38 mg, 71 %, *dr*: 6:1). **^{1}H NMR** (400 MHz, ***major diastereomer***, CDCl$_3$): δ = 6.46 (d, *J* = 8.4 Hz, 1H), 6.38 (d, *J* = 8.4 Hz, 1H), 5.84 (dd, *J* = 17.5, 10.8 Hz, 1H), 5.18 (d, *J* = 10.8 Hz, 1H), 5.05 – 5.00 (m, 2H), 4.56 (dd, *J* = 8.5, 4.9 Hz, 1H), 4.31 (d, *J* = 9.0 Hz, 1H), 4.21 (t, *J* = 5.6 Hz, 1H), 3.73 (s, 3H), 3.50 (d, *J* = 9.0 Hz, 1H), 2.12 – 2.02 (m, 1H), 1.90 – 1.82 (m, 2H), 1.80 – 1.70 (m, 2H), 1.64 (s, 3H), 1.55 (s, 3H), 1.50 – 1.44 (m, 1H), 1.40 – 1.34 (m, 2H), 1.26 (s, 9H), 1.03 (s, 3H), 0.97 – 0.95 (m, 6H), 0.90 – 0.88 (m, 6H) ppm. **^{13}C NMR** (101 MHz, CDCl$_3$): δ = 172.16, 171.00, 170.75, 142.25, 131.66, 124.36, 115.84, 66.64, 59.04, 56.79, 56.62, 52.26, 44.72, 38.05, 37.80, 31.62, 25.80, 25.24, 23.02, 22.71, 19.26, 18.34, 18.32, 17.81, 15.63, 11.68 ppm. **IR (film):** ν_{max} = 3032, 2964, 1743, 1647, 1543, 1203, 1050 cm^{-1}. **HRMS-ESI:** calculated for $C_{28}H_{52}N_3O_5S$ $[M+H]^+$ 542.3622; found 542.3629. **[α]D^{25}**: +34.84 (c 0.366, CHCl$_3$).

Methyl ((2*S*,3*R*)-2-(((*S*)-*tert*-butylsulfinyl)amino)-3,7-dimethyl-3-vinyloct-6-enoyl)-*L*-valyl-*L*-alloisoleucyl-*L*-phenylalaninate (6c):

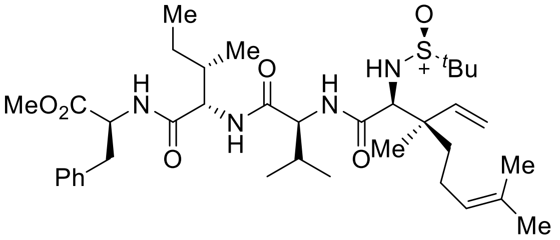

The compound **6c** was synthesized according to **GP V** using imine **4l** (31 mg, 0.057 mmol), **2a** (0.14 mmol, 2.5 equiv), 4 Å molecular sieves (100 mg) at room temperature. Column chromatography on silica using 40% ethylacetate in hexane as eluent provided the product **11k** as a white solid (36 mg, 92%). **^{1}H NMR** (500 MHz, CDCl$_3$): δ = 7.61 (br s, 1H), 7.40 (br s, 1H), 7.23 – 7.18 (m, 3H), 7.11 (d, *J* = 7.2 Hz, 2H), 6.80 (d, *J* = 8.2 Hz, 1H), 5.84 (dd, *J* = 17.4, 10.8 Hz, 1H), 5.09 (d, *J* = 10.8 Hz, 1H), 5.01

– 4.95 (m, 2H), 4.83 (q, *J* = 6.7 Hz, 1H), 4.50 (t, *J* = 8.6 Hz, 1H), 4.39 (d, *J* = 8.6 Hz, 1H), 4.26 (t, *J* = 7.9 Hz, 1H), 3.77 (d, *J* = 8.6 Hz, 1H), 3.65 (s, 3H), 3.09 (dd, *J* = 13.8, 6.0 Hz, 1H), 3.01 (dd, *J* = 13.8, 6.9 Hz, 1H), 2.05 – 1.99 (m, 1H), 1.95 – 1.91 (m, 1H), 1.85 – 1.76 (m, 3H), 1.62 (s, 3H), 1.52 – 1.46 (m, 4H), 1.36 – 1.30 (m, 1H), 1.26 (s, 9H), 1.09 – 1.05 (m, 1H), 1.02 (s, 3H), 0.90 – 0.81 (m, 12H) ppm. **^{13}C NMR** (126 MHz, $CDCl_3$): δ = 172.14, 171.21, 171.13, 171.10, 142.59, 136.35, 131.40, 129.21, 128.61, 127.04, 124.57, 115.02, 66.23, 58.89, 57.68, 56.65, 53.61, 52.29, 44.76, 38.13, 37.73, 37.65, 32.27, 29.79, 25.71, 25.12, 23.08, 22.73, 19.45, 18.67, 17.73, 15.30, 11.47 ppm. **IR (film): ν_{max}** = 3293, 2963, 1745, 1647, 1542, 1218, 1048 cm^{-1}. **HRMS-ESI:** calculated for $C_{37}H_{61}N_4O_6S$ $[M+H]^+$ 689.4306; found 689.4304. **$[\alpha]_D^{25}$**: +53.26 (c 0.168, $CHCl_3$).

Methyl ((2*S*)-2-(((*S*)-*tert*-butylsulfinyl)amino)-4-phenylpent-4-enoyl)-*L*-leucyl-*L*-phenylalanyl-*L*-isoleucinate (6d):

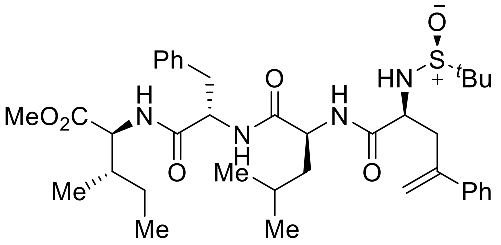

The compound **6d** was synthesized according to **GP V** by using imine **4m** (21 mg, 0.057 mmol), **2v** (0.14 mmol, 2.5 equiv), 4 Å molecular sieves (100 mg) at room temperature. Column chromatography on silica using 40% ethylacetate in hexane as eluent provided the product **6d** as a white solid (23 mg, 60%). **^{1}H NMR** (500 MHz, $CDCl_3$): δ = 7.40 – 7.35 (m, 5H), 7.33 – 7.32 (m, 1H), 7.22 – 7.20 (m, 5H), 7.14 – 7.11 (m, 1H), 6.72 (d, *J* = 8.3 Hz, 1H), 5.38 (s, 1H), 5.14 (s, 1H), 4.64 (td, *J* = 8.5, 6.0 Hz, 1H), 4.47 (dd, *J* = 8.4, 5.4 Hz, 1H), 4.28 (td, *J* = 8.9, 4.8 Hz, 1H), 3.83 (td, *J* = 8.6, 4.5 Hz, 1H), 3.72 (d, *J* = 8.7 Hz, 1H), 3.68 (s, 3H), 3.29 (dd, *J* = 14.5, 4.3 Hz, 1H), 3.19 (dd, *J* = 14.1, 5.7 Hz, 1H), 3.01 (dd, *J* = 14.1, 8.9 Hz, 1H), 2.78 (dd, *J* = 14.6, 8.7 Hz, 1H), 1.88 – 1.82 (m, 1H), 1.52 – 1.44 (m, 2H), 1.39 – 1.25 (m, 3H), 1.14 (s, 9H), 0.88 – 0.82 (m, 12H) ppm. **^{13}C NMR** (126 MHz, $CDCl_3$): δ = 172.33, 171.94, 171.91, 170.99, 144.26, 139.75, 137.54, 129.50, 128.98, 128.55, 128.45, 126.74, 126.47, 117.00, 59.87, 56.88, 56.86, 54.79, 53.23, 52.07, 40.79, 39.26, 37.78, 36.98, 25.24, 25.08, 22.99, 22.49, 22.01, 15.54, 11.62 ppm. **IR (film): ν_{max}** = 3972, 2967, 1755, 1642, 1230, 1058 cm^{-1}. **HRMS-ESI:** calculated for $C_{37}H_{55}N_4O_6S$ $[M+H]^+$ 683.3837; found 683.3836. **$[\alpha]_D^{25}$**: -20.35 (c 0.086, $CHCl_3$).

Comparison of data of 5aa obtained from allylation of imine and peptide coupling:

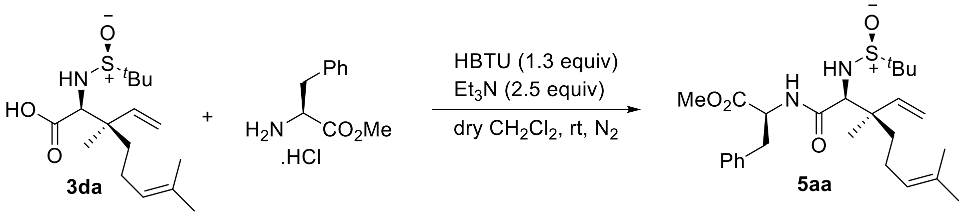

To a solution of carboxylic acid **3da** (157 mg, 0.5 mmol, 1.0 equiv) and (*L*)-phenylalanine-Me-ester·HCl salt (140 mg, 0.65 mmol, 1.3 equiv) in dry CH_2Cl_2, Et_3N (0.174 mL) and HBTU (246 mg, 0.65 mmol, 1.3 equiv) were added at room temperature under N_2 atmosphere. The reaction was stirred overnight. Next morning, the reaction mixture was diluted with CH_2Cl_2, washed with saturated brine solution (2× 5 mL). The organic layer was dried over Na_2SO_4, concentrated, and purified on silica gel column using 20% ethylacetate in hexane as eluent to give **5aa** as colourless oil (42 mg, 18% yield). **^{1}H NMR** (500 MHz, $CDCl_3$): δ = 7.28 (t, *J* = 7.6 Hz, 2H), 7.22 (t, *J* = 7.0 Hz, 1H), 7.16 (d, *J* = 7.6 Hz, 2H), 6.42 (d, *J* = 7.0 Hz, 1H), 5.76 (dd, *J* = 17.5, 10.8 Hz, 1H), 5.06 (d, *J* = 10.8 Hz, 1H), 5.00 – 4.97 (m, 2H), 4.80 (q, *J* = 6.5 Hz, 1H), 4.51 (d, *J* = 8.2 Hz, 1H), 3.67 (s, 3H), 3.50 (d, *J* = 8.2 Hz, 1H), 3.15 (dd, *J* = 13.8, 4.8 Hz, 1H), 3.07 (dd, *J* = 13.8, 6.4 Hz, 1H), 1.86 – 1.81 (m, 2H), 1.64 (s, 3H), 1.54 (s, 3H), 1.49 – 1.43 (m, 1H), 1.40 – 1.36 (m, 1H), 1.27 (s, 9H), 0.99 (s, 3H) ppm. **^{13}C NMR** (126 MHz, $CDCl_3$): δ = 171.61, 170.60, 142.38, 135.84, 131.53, 129.63, 128.72, 127.16, 124.45, 115.78, 65.91, 56.73, 53.62, 52.17, 44.74, 37.99, 37.71, 25.72, 23.06, 22.69, 18.10, 17.75 ppm. **IR (film): ν_{max}** = 3273, 2966, 1747, 1699, 1545, 1207, 1053 cm^{-1}. **HRMS-ESI:** calculated for $C_{26}H_{40}N_2NaO_4S$ $[M+Na]^+$ 499.2601; found 499.2597. **$[\alpha]_D^{25}$**: +122.7 (c 0.52, $CHCl_3$).

Comparison of ^{1}H NMR data of 5aa (500 MHz, $CDCl_3$):

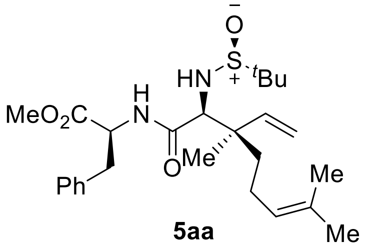 *Obtained from the allylation of* **4a**	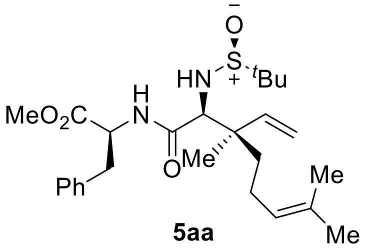 *Obtained from the peptide coupling of* **3da**
7.29 (t, *J* = 7.3 Hz, 2H)	7.28 (t, *J* = 7.6 Hz, 2H)
7.23 (t, *J* = 7.3 Hz, 1H)	7.22 (t, *J* = 7.0 Hz, 1H)
7.15 (d, *J* = 7.1 Hz, 2H)	7.16 (d, *J* = 7.6 Hz, 2H)

6.34 (d, *J* = 7.3 Hz, 1H)	6.42 (d, *J* = 7.0 Hz, 1H)
5.75 (dd, *J* = 17.5, 10.8 Hz, 1H)	5.76 (dd, *J* = 17.5, 10.8 Hz, 1H)
5.06 (d, *J* = 10.9 Hz, 1H)	5.06 (d, *J* = 10.8 Hz, 1H)
5.01 – 4.97 (m, 2H)	5.00 – 4.97 (m, 2H)
4.81 (q, *J* = 6.3 Hz, 1H)	4.80 (q, *J* = 6.5 Hz, 1H)
4.50 (d, *J* = 8.2 Hz, 1H)	4.51 (d, *J* = 8.2 Hz, 1H)
3.68 (s, 3H)	3.67 (s, 3H)
3.48 (d, *J* = 8.3 Hz, 1H)	3.50 (d, *J* = 8.2 Hz, 1H)
3.15 (dd, *J* = 13.8, 4.9 Hz, 1H)	3.15 (dd, *J* = 13.8, 4.8 Hz, 1H)
3.07 (dd, *J* = 13.8, 6.4 Hz, 1H)	3.07 (dd, *J* = 13.8, 6.4 Hz, 1H)
1.85 – 1.81 (m, 2H)	1.86 – 1.81 (m, 2H)
1.64 (s, 3H)	1.64 (s, 3H)
1.54 (s, 3H)	1.54 (s, 3H)
1.49 – 1.43 (m, 2H)	1.49 – 1.43 (m, 1H)<br>1.40 – 1.36 (m, 1H),
1.27 (s, 9H)	1.27 (s, 9H)
0.99 (s, 3H).	0.99 (s, 3H)
**Comparison of ^{13}C NMR Data of 5aa (126 MHz, $CDCl_3$):**	
**5aa** (*Obtained from the allylation of* **4a**)	**5aa** (from peptide coupling)
171.6	171.6
170.6	170.6
142.4	142.4
135.8	135.8
131.6	131.5
129.7	129.6
128.8	128.7
127.2	127.2
124.4	124.4
115.9	115.8
65.9	65.9
56.7	56.7
53.6	53.6
52.2	52.2
44.7	44.7
38.0	38.0
37.7	37.7
25.7	25.7

23.1	23.1
22.7	22.7
18.1	18.1
17.8	17.7

Comparison of NMR Data for the compound 3ae:

Literature Reported Data for **3ae** (Ref: Lin *et al. Chem. Commun.*, **2010**, *46*, 8460-8462)	Experimental Data for **3ae**	Difference
7.32-7.15 (m, 5H)	7.29 (t, *J* = 7.6 Hz, 2H), 7.21 (t, *J* = 7.4 Hz, 1H), 7.15 (d, *J* = 6.8 Hz, 2H),	0.00
6.12-6.00 (m, 1H)	6.08 – 6.02 (m, 1H)	0.02
5.18-5.13 (m, 2H)	5.16 – 5.13 (m, 2H)	0.00
4.22-4.13 (m, 3H)	4.20 – 4.11 (m, 3H)	0.02
3.92 (d, 1H, J = 9.6 Hz)	3.90 (d, *J* = 9.5 Hz, 1H)	0.02
3.69 (t, 1H, J = 8.1 Hz)	3.67 (t, *J* = 8.0 Hz, 1H)	0.02
1.25 (t, 3H, J = 7.2 Hz)	1.25 (t, *J* = 7.2 Hz, 3H)	0.00
1.05 (s, 9H)	1.05 (s, 9H)	0.00
13C Data Comparison **(After calibrating the $CDCl_3$ peak at δ = 77.003 ppm)**		
172.2	172.2	0.0
139.2	139.2	0.0
136.8	136.8	0.0
128.5	128.5	0.0
128.3	128.3	0.0
127.1	127.1	0.0
117.5	117.5	0.0
62.8	62.8	0.0
61.6	61.6	0.0
56.2	56.3	0.1
54.1	54.1	0.0
22.4	22.4	0.0
14.0	14.1	0.1

Comparison of NMR Data for the compound 3ak:

Literature Reported Data for **3ak** (Ref: Lin *et al. Chem. Commun.*, **2010**, *46*, 8460-8462)	Experimental Data for **3ak**	Difference
7.43 (d, 2H, *J* = 8.4 Hz)	7.41 (d, *J* = 8.4 Hz, 2H)	0.02
7.04 (d, 2H, *J* = 8.1 Hz)	7.03 (d, *J* = 8.4 Hz, 2H)	0.01
6.07-5.95 (m, 1H)	5.99 (ddd, *J* = 16.9, 10.3, 8.2 Hz, 1H)	0.04
5.18 (d, 1H, *J* = 4.5 Hz)	5.16 – 5.10 (m, 2H)	0.03
5.13 (d, 1H, *J* = 11.4 Hz)		
4.22-4.12 (m, 3H)	4.19 – 4.11 (m, 3H)	0.01
3.93 (d, 1H, *J* = 9.3 Hz)	3.92 (d, *J* = 9.2 Hz, 1H)	0.01
3.67 (t, 1H, *J* = 8.0 Hz)	3.65 (t, *J* = 7.8 Hz, 1H),	0.02
1.26 (t, 3H, *J* = 7.2 Hz)	1.24 (t, *J* = 7.2 Hz, 3H),	0.02
1.09 (s, 9H)	1.07 (s, 9H)	0.02

13C Data Comparison (After Calibrating the $CDCl_3$ peak at δ = 77.018 ppm)		
171.9	171.9	0.0
138.3	138.3	0.0
136.4	136.3	0.1
131.6	131.6	0.0
130.1	130.1	0.0
121.1	121.0	0.1
118.0	118.0	0.0
62.4	62.4	0.0
61.8	61.7	0.1
56.4	56.3	0.1
53.4	53.4	0.0
22.5	22.5	0.0
14.1	14.1	0.0

N-Sulfinyl amide deprotection and determination of the absolute configuration:

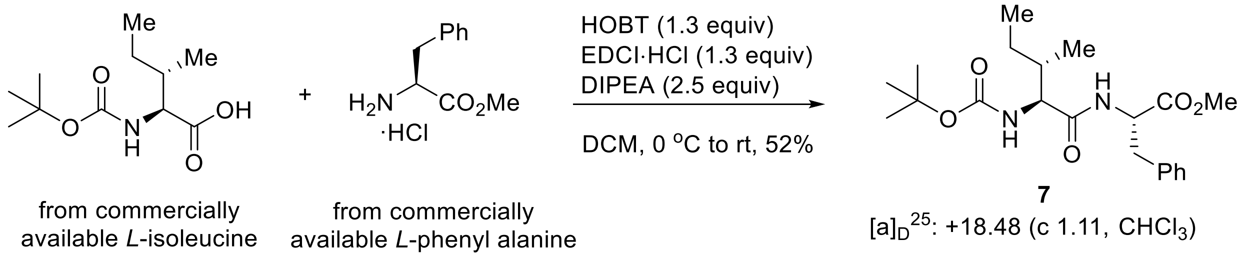

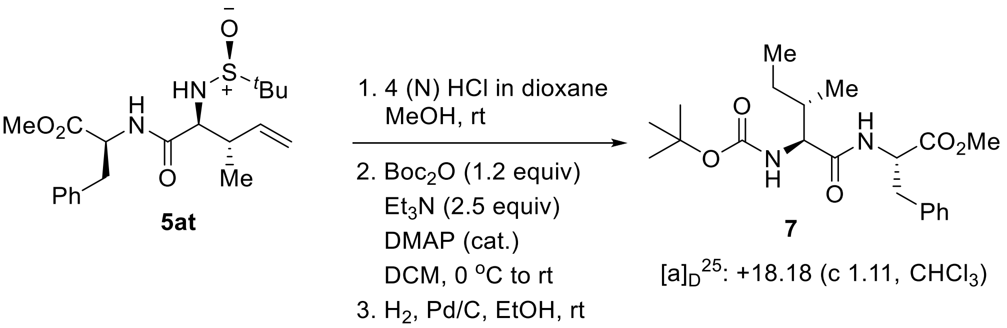

Synthesis of 7: To an ice-cooled solution of (*tert*-butoxycarbonyl)-*L*-isoleucine (578 mg, 2.5 mmol, 1.0 equiv) and methyl *L*-phenylalaninate hydrochloride (700 mg, 3.24 mmol, 1.3 equiv) in dry CH_2Cl_2 (30 mL), DIPEA (807 mg, 1.08 mL, 6.25 mmol. 2.5 equiv) and HOBT (496 mg, 3.24 mmol, 1.3 equiv) were added. After stirring for 30 min at 0 °C, EDC·HCl (620 mg, 3.24 mmol, 1.3 equiv) was added to the reaction mixture and the reaction was slowly allowed to come to room temperature and stirring was continued for further 18 h. After complete consumption of starting material, reaction mixture was diluted with CH_2Cl_2, and washed with 2×30 mL 10% aq. citric acid solution, 1 M HCl, and brine solution. The organic layer was dried over Na_2SO_4, concentrated and purified on silica gel column using 12% ethylacetate in hexane as eluent to get dipeptide **7** as white solid (510 mg, 52%). **^{1}H NMR** (500 MHz, $CDCl_3$): δ = 7.29 – 7.26 (m, 2H), 7.25 – 7.19 (m, 1H), 7.12 (d, *J* = 7.4 Hz, 2H), 6.48 (d, *J* = 7.5 Hz, 1H),

5.10 – 5.06 (m, 1H), 4.87 (q, *J* = 6.4 Hz, 1H), 3.97 – 3.92 (m, 1H), 3.69 (s, 3H), 3.13 (dd, *J* = 13.8, 5.7 Hz, 1H), 3.07 (dd, *J* = 13.8, 6.1 Hz, 1H), 1.85 – 1.79 (m, 1H), 1.46 – 1.42 (m, 10H), 1.10 – 1.05 (m, 1H), 0.89 – 0.85 (m, 6H) ppm. **^{13}C NMR** (126 MHz, $CDCl_3$): δ = 171.77, 171.42, 155.74, 135.88, 129.33, 128.65, 127.19, 79.90, 59.34, 53.22, 52.27, 38.10, 37.29, 28.39, 24.75, 15.50, 11.42 ppm. **IR (film): ν_{max}** = 2969, 1659, 1499, 1367, 1216, 1166 cm^{-1}. **HRMS-ESI:** calculated for $C_{21}H_{32}N_2NaO_5$ $[M + Na]^+$ 415.2209; found 415.2190. **$[\alpha]_D^{25}$**: +18.48 (c 1.11, $CHCl_3$).

Synthesis of 7 from 5at:

Step I: To a solution of **5at** (150 mg, 0.38 mmol) in MeOH, 4 (N) HCl in dioxane (1.14 mmol, 0.285 mL) was added at room temperature. The reaction was stirred for 30 min, until all the starting material was consumed. Then, MeOH was removed under reduced pressure by using rotary evaporator and the residue was dissolved in 1 (N) NaOH and extracted with ethylacetate (4×8 mL). The combined organic layer was dried over Na_2SO_4, evaporated and the crude product was used for next step.

Step II: The crude material obtained from step I was dissolved in anhydrous CH_2Cl_2 (6 mL), and to it was sequentially added Et_3N (96 mg, 0.95 mmol, 2.5 equiv), Boc_2O (100 mg, 0.45 mmol, 1.2 equiv), and DMAP (~2-3 mg) at 0 ^oC (ice-bath). The reaction was allowed to slowly come to room temperature and stirred overnight. Next, it was diluted with CH_2Cl_2 and washed with saturated brine solution (3×10 mL). The combined organic layer was dried, concentrated and used for next step.

Step III: The crude mixture was dissolved in EtOH (8 mL) and to it was added Pd/C (50 mg) under hydrogen atmosphere (using hydrogen balloon) at room temperature. The reaction was stirred for 24 hours, then diluted with CH_2Cl_2 (15 mL), and filtered through cellite. The crude mixture was concentrated and purified on silica gel column using 12% ethylacetate in hexane as eluent to give **7** (38 mg, 26% over three steps) as white solid.

^{1}H NMR (500 MHz, $CDCl_3$): δ = 7.29 – 7.26 (m, 2H), 7.24 – 7.21 (m, 1H), 7.12 (d, *J* = 7.5 Hz, 2H), 6.45 (d, *J* = 6.9 Hz, 1H), 5.08 (d, *J* = 8.5 Hz, 1H), 4.87 (q, *J* = 6.3 Hz, 1H), 3.97 – 3.91 (m, 1H), 3.70 (s, 3H), 3.13 (dd, *J* = 13.8, 5.6 Hz, 1H), 3.08 (dd, *J* = 13.7, 6.0 Hz, 1H), 1.85 – 1.79 (m, 1H), 1.46 – 1.42 (m, 10H), 1.12 – 1.04 (m, 1H), 0.89 – 0.85 (m, 6H) ppm. **^{13}C NMR** (126 MHz, $CDCl_3$): δ = 171.78, 171.45, 155.79, 135.87, 129.35, 128.69, 127.23, 79.97, 59.36, 53.24, 52.32, 38.12, 37.30, 28.41, 24.78, 15.52, 11.45 ppm. **IR (film): ν_{max}** = 2952,

1678, 1507, 1375, 1242, 1141 cm^{-1}. **HRMS-ESI:** calculated for $C_{21}H_{32}N_2NaO_5$ $[M+Na]^+$ 415.2203; found 415.2187. **$[\alpha]_D^{25}$**: +18.18 (c 1.11, $CHCl_3$).

Comparison of NMR Data for the compound 7:

^{1}H NMR Data for **7** (obtained by direct peptide coupling)	^{1}H NMR Data for **7** (obtained from **5at**)	Difference
7.29 – 7.26 (m, 2H)	7.29 – 7.26 (m, 2H)	0.0
7.25 – 7.19 (m, 1H)	7.24 – 7.21 (m, 1H)	0.01
7.12 (d, *J* = 7.4 Hz, 2H)	7.12 (d, *J* = 7.5 Hz, 2H),	0.0
6.48 (d, *J* = 7.5 Hz, 1H)	6.45 (d, *J* = 6.9 Hz, 1H)	0.03
5.10 – 5.06 (m, 1H)	5.08 (d, *J* = 8.5 Hz, 1H)	0.02
4.87 (q, *J* = 6.4 Hz, 1H)	4.87 (q, *J* = 6.3 Hz, 1H)	0.0
3.97 – 3.92 (m, 1H)	3.97 – 3.91 (m, 1H)	0.0
3.69 (s, 3H)	3.70 (s, 3H)	0.01
3.13 (dd, *J* = 13.8, 5.7 Hz, 1H)	3.13 (dd, *J* = 13.8, 5.6 Hz, 1H)	0.0
3.07 (dd, *J* = 13.8, 6.1 Hz, 1H),	3.08 (dd, *J* = 13.7, 6.0 Hz, 1H)	0.01
1.85 – 1.79 (m, 1H)	1.85 – 1.79 (m, 1H)	0.0
1.46 – 1.42 (m, 10H)	1.46 – 1.42 (m, 10H),	0.0
1.10 – 1.05 (m, 1H)	1.12 – 1.04 (m, 1H)	0.02
0.89 – 0.85 (m, 6H)	0.89 – 0.85 (m, 6H)	0.0
**Comparison of ^{13}C NMR Data**		
171.8	171.8	0.0
171.4	171.4	0.0
155.7	155.8	0.1
135.9	135.9	0.0
129.3	129.3	0.0
128.6	128.7	0.1
127.2	127.2	0.0
79.9	80.0	0.1
59.3	59.4	0.1
53.2	53.2	0.0
52.3	52.3	0.0
38.1	38.1	0.0
37.3	37.3	0.0
28.4	28.4	0.0
24.7	24.8	0.1
15.5	15.5	0.0
11.4	11.4	0.0

Synthetic Modifications of Homoallyl Amino Esters:

(*S*)-N-((2*S*,3*R*)-1-hydroxy-3,7-dimethyl-3-vinyloct-6-en-2-yl)-2-methylpropane-2-sulfinamide (8):

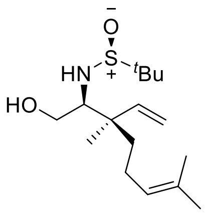

In a 25 ml two-necked round bottom flask containing $LiAlH_4$ (57 mg, 1.5 mmol, 1.5 equiv) was added dry THF under nitrogen at 0 °C. Then the solution of **3aa** (344 mg, 1.0 mmol) in THF (2 mL) was added to it dropwise. Then the reaction mixture was stirred for 0.5 h, until all the starting material was consumed. After completion of the reaction as indicated by TLC, reaction was quenched by adding aq. NH_4Cl solution. The crude mixture was filtered through celite pad and washed with EtOAc. The organic layer was dried over Na_2SO_4, concentrated, and purified on silica gel column using 20% ethylacetate in hexane as eluent to give **8** as colourless oil (187 mg, 62% yield). **^{1}H NMR** (400 MHz, $CDCl_3$): δ = 5.88 (dd, *J* = 17.6, 10.8 Hz, 1H), 5.55 (d, *J* = 10.8 Hz, 1H), 5.31 (d, *J* = 17.6 Hz, 1H), 5.07 – 5.03 (m, 1H), 3.87 – 3.86 (m, 2H), 3.41 – 3.34 (m, 1H), 3.30 (d, *J* = 10.5 Hz, 1H), 2.97 (t, *J* = 9.6 Hz, 1H), 2.08 – 1.98 (m, 2H), 1.81 – 1.77 (m, 1H), 1.75 – 1.70 (m, 1H), 1.68 (s, 3H), 1.58 (s, 3H), 1.45 (s, 3H), 0.92 (s, 9H) ppm. **^{13}C NMR** (101 MHz, $CDCl_3$) δ = 135.54, 132.63, 123.37, 119.96, 69.47, 65.07, 63.11, 34.67, 34.05, 27.17, 25.81, 22.61, 18.94, 17.85 ppm. **IR (film): ν_{max}** = 3406, 2963, 1465, 1366, 1127, 1024 cm^{-1}. **HRMS-ESI:** calculated for $C_{16}H_{32}NO_2S$ $[M+H]^+$ 302.2148; found 302.2133. **$[\alpha]_D^{25}$**: +93.35 (c 0.23, $CHCl_3$).

Ethyl (*4R*,*5R*,*E*)-4-(((*S*)-*tert*-butylsulfinyl)amino)-5,9-dimethyl-5-vinyldeca-2,8-dienoate (9):

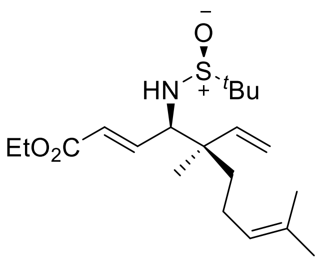

To a solution of **8** (151 mg, 0.5 mmol, 1.0 equiv) in dry CH_2Cl_2 (5 mL) was added Dess Martin periodinane (276 mg, 0.65 mmol, 1.3 equiv) at rt. The reaction was stirred at rt for 0.5 h. After completion of the reaction, it was quenched with saturated solution of $Na_2S_2O_3$ (10 mL). The organic layer diluted with CH_2Cl_2 and washed with brine. The organic layer was dried over anhydrous Na_2SO_4, concentrated and used for next step without further purification.

A 25 mL two neck round bottom flask was charged with NaH (26 mg, 0.65 mmol, 1.3 equiv) and backfilled three times with nitrogen. Then 5 mL dry THF was added and the reaction mixture was cooled to 0 °C (ice-bath). Then triethylphosphonoacetate (145 mg, 1.3 equiv, 0.13 mL) was added dropwise. The reaction mixture was stirred at 0 °C for 15 min. Then, a solution

of the crude aldehyde in 3 mL THF was added slowly at 0 °C. The reaction mixture was stirred at rt for 1 h and subsequently quenched with saturated brine solution. The organic layer was separated, dried over anhydrous Na_2SO_4, concentrated and purified on silica using 8% ethylacetate in hexane as eluent to get **9** as colourless oil (98 mg, 53% over two steps). **^{1}H NMR** (500 MHz, $CDCl_3$): δ = 6.95 (dd, *J* = 15.6, 7.5 Hz, 1H), 6.01 (d, *J* = 16.6 Hz, 1H), 5.89 (dd, *J* = 17.6, 10.8 Hz, 1H), 5.52 (d, *J* = 10.9 Hz, 1H), 5.28 (d, *J* = 17.6 Hz, 1H), 5.06 (t, *J* = 7.1 Hz, 1H), 4.21 – 4.14 (m, 2H), 3.56 (t, *J* = 9.0 Hz, 1H), 3.43 (d, *J* = 9.7 Hz, 1H), 2.04 – 1.97 (m, 2H), 1.76 – 1.70 (m, 1H), 1.68 (s, 3H), 1.65 – 1.61 (m, 1H), 1.59 (s, 3H), 1.40 (s, 3H), 1.27 (t, *J* = 7.1 Hz, 3H), 0.92 (s, 9H) ppm. **^{13}C NMR** (126 MHz, $CDCl_3$): δ = 166.15, 146.23, 136.21, 132.47, 123.94, 123.63, 119.35, 67.08, 65.78, 60.60, 35.99, 34.18, 26.77, 25.78, 22.71, 18.34, 17.83, 14.35 ppm. **IR (film): ν_{max}** = 2968, 2930, 1737, 1368, 1255, 1023 cm^{-1}. **HRMS-ESI:** calculated for $C_{20}H_{36}NO_3S$ $[M+H]^+$ 370.2410; found 370.2397. **$[\alpha]_D^{25}$**: +143.78 (c 0.23, $CHCl_3$).

(*S*)-4-((*S*)-1-phenylallyl) oxazolidin-2-one (10):

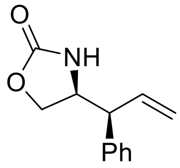

To a slurry of $LiAlH_4$ (28 mg, 0.75 mmol, 1.5 equiv) in dry THF (3 mL) at 0 °C was dropwisely added a solution of **3ae** (161 mg, 0.5 mmol, 1.0 equiv) in 3 mL THF. The reaction mixture was stirred at rt for 0.5 h and quenched with saturated aq. Na_2SO_4 solution. The reaction mixture was diluted with EtOAc, filtered through celite, dried over Na_2SO_4, concentrated and used for next step.

The amino alcohol was dissolved in MeOH (3 mL) and cooled to 0 °C (ice-bath). Then 4 N HCl-dioxane (0.37 mL, 1.5 mmol, 3.0 equiv) was added to it. After 15 min at 0 °C, the reaction was brought to rt and stirred for 3 h. After complete consumption of the starting material, MeOH was concentrated, and the residue was dissolved in ethylacetate. The organic layer was washed with 2(M) aq. Na_2CO_3 solution. The organic layer was dried using Na_2SO_4, concentrated, and redissolved in dry CH_2Cl_2. Then 1,1′-carbonyldiimidazole (48 mg, 0.3 mmol, 0.6 equiv) was added to it and the reaction was stirred overnight. The crude reaction mixture was concentrated and purified on silica using 25% ethylacetate in hexane as eluent to get **10** as whitish solid (40 mg, 40% over three steps). **^{1}H NMR** (500 MHz, $CDCl_3$): δ = 7.37 (t, *J* = 7.5 Hz, 2H), 7.31 – 7.28 (m, 1H), 7.21 – 7.19 (m, 2H), 5.94 – 5.87 (m, 1H), 5.24 – 5.18 (m, 2H), 4.86 – 4.78 (m, 1H), 4.53 – 4.49 (m, 1H), 4.27 (dd, *J* = 9.0, 5.6 Hz, 1H), 4.13 – 4.08 (m, 1H), 3.35 (t, *J* = 8.9 Hz, 1H) ppm. **^{13}C NMR** (101 MHz, $CDCl_3$): δ = 158.80, 139.33, 135.98, 129.42, 127.96, 127.86, 118.44, 68.92, 55.97, 55.56 ppm. **IR (film): ν_{max}** = 3244, 1758, 1685, 1404,

1237, 931 cm^{-1}. **HRMS-ESI:** calculated for $C_{12}H_{14}NO_2$ $[M+H]^+$ 204.1019; found 204.1003. **[α]$_D$^{25}**: -100.00 (c 0.1, $CHCl_3$).

Ethyl (2*S*,3*S*)-2-(((*S*)-*tert*-butylsulfinyl)amino)-5-hydroxy-3-phenylpentanoate (11):

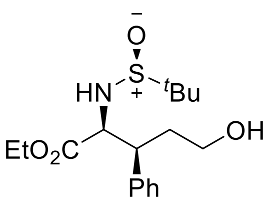

In a 25 ml two-necked round bottom flask containing homoallylic amino ester **3ae** (33 mg, 0.1 mmol, 1.0 equiv) was dissolved in 6 mL THF and cooled at –78 °C. $BH_3{\cdot}SMe_2$ (0.25 mmol, 2.5 equiv, 25 μL) was added dropwise to the reaction mixture. The reaction mixture was stirred at this temperature for 1 h, then allowed to warm to room temperature and stirred for additional 1 h. Then the reaction was cooled at 0 °C and NaOAc (42 mg, 0.5 mmol, 5.0 equiv) solution in 2 mL of water mixed with 0.1 mL of H_2O_2 (30% in H_2O) was added dropwise into the reaction mixture. The reaction was then allowed to warm to room temperature and stirred overnight. The aqueous layer was extracted with EtOAc (3 × 5 mL) and dried over anhydrous Na_2SO_4. The crude reaction mixture was purified on silica using 70% ethylacetate in hexane as eluent to give **11** as colourless oil (11 mg, 31%). **^{1}H NMR** (400 MHz, $CDCl_3$): δ = 7.30 – 7.23 (m, 3H), 7.13 (d, *J* = 7.2 Hz, 2H), 4.20 – 4.12 (m, 3H), 4.00 (d, *J* = 8.8 Hz, 1H), 3.65 – 3.58 (m, 1H), 3.50 – 3.44 (m, 1H), 3.35 – 3.30 (m, 1H), 2.05 – 1.96 (m, 3H), 1.28 – 1.25 (m, 3H), 1.16 (s, 9H) ppm. **^{13}C NMR** (101 MHz, $CDCl_3$): δ = 172.38, 139.19, 128.65, 127.49, 62.60, 61.90, 60.46, 56.47, 45.81, 35.20, 22.78, 14.25 ppm. **IR (film): ν_{max}** = 2927, 1733, 1445, 1366, 1178, 1044 cm^{-1}. **HRMS-ESI:** calculated for $C_{17}H_{28}NO_4S$ $[M+H]^+$ 342.1734; found 342.1719. **[α]$_D$^{25}**: +9.17 (c 0.3, $CHCl_3$)

Ethyl (*S*)-2-((1,1-dimethylethyl)sulfonamido)-4-phenylpent-4-enoate (12):

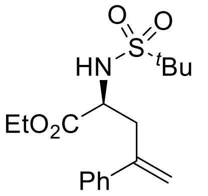

To a solution of **3av** (162 mg, 0.5 mmol, 1 equiv) in CH_2Cl_2 (5 mL) was added *m*-CPBA (130 mg, 0.75 mmol, 1.5 equiv), and the reaction mixture was stirred at room temperature for 1 h. Upon completion, 10% aqueous $Na_2S_2O_3$ (3 mL) and saturated aqueous $NaHCO_3$ (2 mL) were added to the reaction vessel. Then the reaction mixture was diluted with CH_2Cl_2, washed with saturated brine solution (2×5 mL). The organic layer was dried over Na_2SO_4, concentrated, and purified on silica gel column using 10% ethylacetate in hexane as eluent to give **12** as colourless oil (138 mg, 81% yield). **^{1}H NMR** (400 MHz, $CDCl_3$): δ = 7.40 (d, *J* = 7.5 Hz, 2H), 7.34 (t, *J* = 7.4 Hz, 2H), 7.29 (d, *J* = 7.1 Hz, 1H), 5.42 (s, 1H), 5.19 (s, 1H), 4.54 (d, *J* = 10.1 Hz, 1H), 4.16 – 4.10 (m, 1H), 4.07 – 3.93 (m, 2H), 3.01 (d, *J* = 6.5 Hz, 2H), 1.26 (s, 9H), 1.20 (t, *J* = 7.2 Hz, 3H) ppm. **^{13}C NMR** (101 MHz, $CDCl_3$): δ = 172.10, 142.97, 139.48, 128.62, 128.15, 126.45,

117.34, 61.70, 60.25, 56.33, 39.98, 24.05, 14.12 ppm. **IR (film):** ν_{max} = 2975, 1732, 1445, 1301, 1185, 1070 cm^{-1}. **HRMS-ESI:** calculated for $C_{17}H_{26}NO_4S$ $[M+H]^+$ 340.1577; found 340.1580. **$[\alpha]_D^{25}$**: -60.15 (c 0.13, $CHCl_3$).

Ethyl (*S*)-2-((1,1-dimethylethyl)sulfonamido)-4-oxo-4-phenylbutanoate (13):

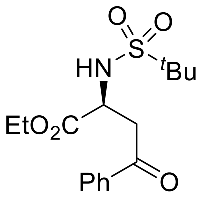

In a 10 mL oven-dried Schlenk tube containing alkene **12** (80 mg, 0.2 mmol, 1.0 equiv) in CH_3NO_2 (1 mL) were added AIBN (9 mg, 0.05 mmol, 0.25 equiv). Then the tube was evacuated and filled with oxygen (this procedure was repeated three times). The mixture was stirred at 60 °C for 8 h and then cooled to room temperature. The reaction mixture was diluted with EtOAc concentrated, and purified by silica gel column using 12% ethylacetate in hexane as eluent to give **13** as colourless oil (50 mg, 74% yield). **^{1}H NMR** (400 MHz, $CDCl_3$): δ = 7.93 (d, *J* = 8.2 Hz, 2H), 7.61 (t, *J* = 7.2 Hz, 1H), 7.48 (t, *J* = 7.6 Hz, 2H), 5.17 (d, *J* = 9.6 Hz, 1H), 4.47 – 4.43 (m, 1H), 4.28 – 4.19 (m, 2H), 3.83 (dd, *J* = 18.2, 3.7 Hz, 1H), 3.59 (dd, *J* = 18.2, 4.1 Hz, 1H), 1.43 (s, 9H), 1.25 (t, *J* = 7.1 Hz, 3H) ppm. **^{13}C NMR** (126 MHz, $CDCl_3$): δ = 197.70, 171.15, 136.14, 134.02, 128.93, 128.34, 62.21, 60.64, 53.54, 43.35, 24.29, 14.19 ppm. **IR (film):** ν_{max} = 3297, 2927, 1740, 1683, 1450, 1317, 1136 cm^{-1}. **HRMS-ESI:** calculated for $C_{16}H_{24}NO_5S$ $[M+H]^+$ 342.1370; found 342.1355. **$[\alpha]_D^{25}$**: -15.00 (c 0.1, $CHCl_3$).

Computational Details:

All density functional theory (DFT) calculations were performed within the Gaussian 09 suite of quantum chemical package.[3] Geometries of all the stationary points were optimized in the solvent phase with the B3LYP functional [4] in conjunction with Pople's 6-31G(d,p) double-ζ split valence basis set on all atoms.[5] Solvent effect was introduced by utilizing conductor-like polarizable continuum (CPCM) solvation model[6] employing the dielectric parameters of chloroform as solvent (ε=4.7113). Harmonic vibrational frequencies were computed at the same level of theory, to distinguish transition states (with one negative Hessian index) from minima (with all positive Hessian index). The non-thermal zero-point energy (ZPE) correction and the thermal corrections to enthalpy and Gibbs free energy were obtained from the frequency calculations, using standard approximations at 298.15 K and 1 atm pressure. Furthermore, the electronic energies were refined by single point calculations at B3LYP functional overlayed with Grimme's empirical dispersion D3(BJ) correction term,[7] employing the IOp(3/124=40) keyword with the larger triple-ζ 6-311+G(d,p) basis set. This approach has been recently successful in evaluating boronic acid assisted stereo-isomerism of imines.[8]

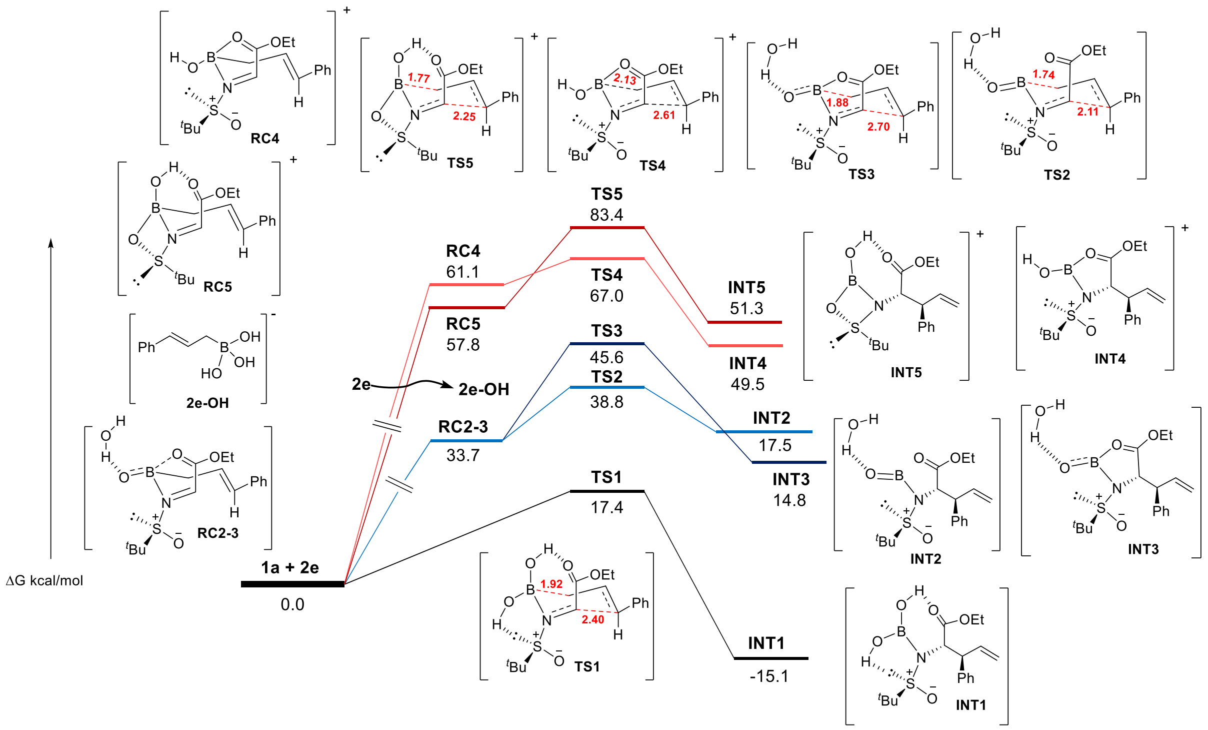

Figure S1. Relative Gibbs free energy profile at B3LYP-D3BJ(CPCM)/6-311+G(d,p) for C-C coupling reaction step. Distances shown are in units of Å.

Initially, the reaction is thought to be undergoing through a chelation assisted monocationic six-membered transition state (**TS4**) in which both the imine nitrogen and the carbonyl moiety co-ordinate to the boron atom. The intrinsic barrier for **TS4** with respect to the preceding reactant complex **RC4** is exceedingly low (~ 6 kcal/mol, Figure S1). However, formation of **RC4** itself is a predominantly high endoergic process (61.1 kcal) starting from the reactants and hence the free energy barrier for **TS4** turns out to be 67.0 kcal/mol. Therefore, the C(imine)-C(allyl) coupling step is not possible though this mechanism. Similarly, another monocationic six-membered transition state (**TS5**) featuring coordination of the imine nitrogen and sulfinyl oxygen to the boron centre and shows even higher free energetic expense at 83.4 kcal/mol ruling out the possibility of C(imine)-C(allyl) coupling through the positively charged transition states. Thereby, we hypothesized neutral transition states starting from the reactant complex **RC2-3** where one OH^- and one H^+ released from the allyl boronic acid forms a water molecule which is coordinated to the remaining boronic acid through H-bonding. Here two situations are envisaged: **TS2** where there is no coordination of the ester oxygen to the boron centre and **TS3** where an explicit B-O(ester) bond is formed. Although, free energy barrier for **TS2** (38.8 kcal/mol) and **TS3** (45.6 kcal/mol) are significantly lower as compared to the

monocationic transition states, they are too high to occur at the experimental conditions. Instead, we predict a concerted mechanism of B-C bond cleavage and C-C coupling through the neutral **TS1** (17.4 kcal/mol) which is further assisted by chelating H-bonding interactions, as reported in the main text. **TS1** is followed by **INT1** (-15.1 kcal/mol) which is a highly exoergic intermediate with respect to the reactants (**1** and **2**), presumably due to the chelating H-bonding interactions. I**NT1** can further undergo hydrolysis to generate the desired product **3ae**.

XYZ coordinates

1

C	0.745285000	-0.241127000	-0.411212000
N	-0.325385000	0.380597000	-0.717192000
C	2.035041000	0.529531000	-0.320623000
O	2.164973000	1.713595000	-0.549994000
O	3.018937000	-0.298647000	0.058522000
C	4.338195000	0.293248000	0.204377000
H	4.270522000	1.111547000	0.927165000
H	4.632927000	0.718347000	-0.759556000
C	5.281787000	-0.800579000	0.661740000
H	5.327792000	-1.610939000	-0.071222000
H	4.964194000	-1.215797000	1.622313000
H	6.287612000	-0.387293000	0.781149000
S	-1.742208000	-0.630656000	-0.862248000
O	-1.475754000	-2.004394000	-0.290379000
C	-2.788951000	0.312498000	0.408631000
C	-4.108530000	-0.472685000	0.440330000
H	-3.948584000	-1.504223000	0.764619000
H	-4.790590000	0.008272000	1.148802000
H	-4.596106000	-0.485226000	-0.540142000
C	-2.088420000	0.270198000	1.766727000
H	-1.193237000	0.896824000	1.780207000
H	-2.774220000	0.638090000	2.537326000
H	-1.806654000	-0.755245000	2.023796000
C	-2.994886000	1.734747000	-0.119511000
H	-3.440342000	1.733280000	-1.119979000
H	-3.684760000	2.261202000	0.549216000
H	-2.056983000	2.292062000	-0.155902000
H	0.779080000	-1.314006000	-0.199429000

2

B	3.492836000	0.104541000	-0.117578000
C	2.418390000	-0.598576000	0.818190000
C	1.023407000	-0.064479000	0.667893000
C	-0.041134000	-0.794768000	0.297401000
H	2.763968000	-0.427832000	1.852372000
H	2.440941000	-1.683077000	0.664629000
H	0.905683000	1.000728000	0.861939000
H	0.109575000	-1.858886000	0.110592000
O	4.520993000	-0.664137000	-0.596760000
H	5.167306000	-0.185248000	-1.131779000
C	-1.424869000	-0.328983000	0.109417000
C	-1.821888000	1.014711000	0.263062000
C	-2.413887000	-1.266347000	-0.243693000
H	-1.088075000	1.769290000	0.529901000
H	-2.131061000	-2.308826000	-0.368586000
C	-3.147852000	1.396622000	0.074233000
C	-3.742305000	-0.884865000	-0.433836000
H	-3.427609000	2.439301000	0.198255000
H	-4.483401000	-1.631491000	-0.705663000
C	-4.117420000	0.450393000	-0.275431000
H	-5.150125000	0.752702000	-0.422837000
O	3.352738000	1.444240000	-0.370654000
H	4.044120000	1.827561000	-0.925038000

2-OH

B	-3.106926000	0.071420000	0.195214000
C	-2.115648000	-0.336614000	-1.122953000
C	-0.717141000	0.083976000	-0.919444000
C	0.313724000	-0.705267000	-0.531744000
H	-2.544706000	0.161618000	-2.003016000
H	-2.180650000	-1.423012000	-1.275531000
H	-0.538093000	1.156909000	-1.011410000
H	0.132877000	-1.779121000	-0.458002000
O	-4.496035000	-0.363483000	-0.036896000
H	-4.555032000	-1.263844000	0.302941000
C	1.686956000	-0.291268000	-0.226626000
C	2.112064000	1.055709000	-0.189393000
C	2.660380000	-1.277088000	0.043097000
H	1.395358000	1.849942000	-0.376281000
H	2.363491000	-2.323470000	0.026145000
C	3.435075000	1.389796000	0.089150000
C	3.984448000	-0.942864000	0.325438000
H	3.727821000	2.436809000	0.108755000
H	4.705675000	-1.731088000	0.527349000
C	4.384980000	0.395251000	0.348630000
H	5.415374000	0.660345000	0.567991000
O	-3.061657000	1.542211000	0.287027000
H	-3.687739000	1.781657000	0.982564000
O	-2.629900000	-0.624798000	1.413257000
H	-1.668033000	-0.545066000	1.428410000

TS1

B	1.746910000	-1.807269000	0.931495000
C	0.646685000	-2.853679000	-0.248994000
C	-0.683268000	-2.383042000	-0.173401000
C	-1.191384000	-1.371556000	-0.978539000
C	0.255955000	0.211707000	0.108137000
N	1.424633000	-0.410544000	0.225653000
H	0.852623000	-3.796735000	0.255561000
H	1.127171000	-2.777626000	-1.225424000
H	-1.281661000	-2.727878000	0.667797000
H	-0.623088000	-1.116274000	-1.870411000
C	-0.675163000	0.448643000	1.241459000
O	-0.737305000	-0.169438000	2.298081000
O	-1.472372000	1.479532000	0.944820000
C	-2.457930000	1.858346000	1.946499000
H	-1.925594000	2.152169000	2.855715000
H	-3.071042000	0.983304000	2.173824000
C	-3.275799000	2.993689000	1.365456000

H	-3.795583000	2.672657000	0.458752000
H	-2.641952000	3.851626000	1.123850000
H	-4.023458000	3.313890000	2.097197000
O	3.068642000	-2.267251000	0.717062000
S	2.548742000	-0.043644000	-1.156027000
O	1.733271000	0.678399000	-2.203127000
H	3.248983000	-2.521303000	-0.193485000
C	3.606393000	1.298074000	-0.315023000
C	4.199418000	0.739058000	0.979938000
H	4.712153000	-0.212185000	0.820012000
H	4.925675000	1.462329000	1.367112000
H	3.431444000	0.585415000	1.741296000
C	4.699747000	1.564324000	-1.363835000
H	4.274025000	1.921104000	-2.305788000
H	5.375391000	2.336696000	-0.982289000
H	5.295812000	0.667946000	-1.563958000
C	2.748502000	2.540462000	-0.083184000
H	2.006954000	2.381494000	0.704090000
H	3.398267000	3.364103000	0.231314000
H	2.234453000	2.840711000	-0.999952000
C	-2.577873000	-0.881050000	-0.970943000
C	-3.504545000	-1.193268000	0.044453000
C	-3.011454000	-0.061164000	-2.030701000
H	-3.208445000	-1.819471000	0.879982000
H	-2.312243000	0.190744000	-2.824016000
C	-4.808838000	-0.704751000	-0.004792000
C	-4.316420000	0.426390000	-2.079826000
H	-5.506850000	-0.961711000	0.786953000
H	-4.625995000	1.053276000	-2.911142000
C	-5.223057000	0.107417000	-1.065573000
H	-6.241409000	0.482743000	-1.102046000
H	0.143260000	0.891536000	-0.733539000
O	1.356477000	-1.928344000	2.258943000
H	0.568743000	-1.390899000	2.454975000

INT1

B	-1.928092000	1.704059000	1.121047000
C	0.623598000	3.833511000	-0.168710000
C	0.991098000	2.639818000	0.300735000
C	0.952607000	1.378886000	-0.525218000
C	0.003413000	0.252617000	0.045494000
N	-1.390898000	0.694705000	0.188854000
H	0.673239000	4.726731000	0.447036000
H	0.262723000	3.960285000	-1.186856000
H	1.341017000	2.558982000	1.327306000
H	0.487463000	1.625743000	-1.485360000
C	0.570550000	-0.383136000	1.314979000
O	0.579812000	0.128340000	2.428807000
O	1.109296000	-1.572833000	1.062566000
C	1.783710000	-2.245427000	2.168276000
H	1.043348000	-2.448600000	2.946536000
H	2.531621000	-1.560779000	2.576009000
C	2.404275000	-3.510894000	1.613392000
H	3.132216000	-3.276708000	0.831833000
H	1.641169000	-4.173424000	1.195590000
H	2.920231000	-4.044226000	2.417170000
O	-3.107744000	2.357507000	0.852663000
S	-2.441941000	0.234014000	-1.124485000
O	-1.570910000	-0.058439000	-2.327815000
H	-3.439988000	2.220359000	-0.042513000
C	-3.143010000	-1.440634000	-0.578248000
C	-3.760969000	-1.259264000	0.809934000
H	-4.476938000	-0.430705000	0.834224000
H	-4.302234000	-2.171735000	1.082103000
H	-2.997629000	-1.079948000	1.571236000
C	-4.222933000	-1.722567000	-1.636544000
H	-3.787319000	-1.805524000	-2.636113000
H	-4.718529000	-2.670006000	-1.400899000
H	-4.987870000	-0.939063000	-1.652528000
C	-2.057230000	-2.515058000	-0.607771000
H	-1.319362000	-2.380336000	0.186860000
H	-2.520700000	-3.497740000	-0.466659000
H	-1.540691000	-2.517840000	-1.571566000
C	2.331413000	0.788915000	-0.822482000
C	3.401236000	0.867511000	0.080572000
C	2.528860000	0.111107000	-2.035370000
H	3.284417000	1.395219000	1.021910000
H	1.708581000	0.044751000	-2.746068000
C	4.632530000	0.278678000	-0.219717000
C	3.757377000	-0.476976000	-2.337519000
H	5.450486000	0.353770000	0.491238000
H	3.889704000	-0.993141000	-3.284164000
C	4.815084000	-0.396291000	-1.428094000
H	5.773893000	-0.849660000	-1.662135000
H	0.016949000	-0.522371000	-0.721091000
O	-1.348189000	1.987582000	2.313757000
H	-0.585787000	1.406470000	2.509380000

RC2-3

B	-1.578848000	1.670089000	0.024212000
C	-0.522375000	2.158878000	-1.113259000
C	0.898101000	1.764652000	-0.915949000
C	1.512734000	0.722348000	-1.519944000
C	-0.440499000	-0.629945000	0.499329000
N	-1.462841000	0.024561000	0.084204000
H	-0.637711000	3.249385000	-1.104666000
H	-0.895039000	1.788540000	-2.078430000
H	1.449475000	2.352107000	-0.183917000
H	0.941768000	0.139739000	-2.243692000
C	0.491023000	-0.048370000	1.524888000
O	0.322522000	1.023261000	2.071860000
O	1.482391000	-0.905766000	1.757268000
C	2.469369000	-0.504537000	2.754352000
H	1.948946000	-0.364306000	3.705852000
H	2.890200000	0.455620000	2.446130000
C	3.516393000	-1.596200000	2.820301000
H	4.021977000	-1.709818000	1.857682000
H	3.069159000	-2.553601000	3.101561000
H	4.264572000	-1.331834000	3.573308000
O	-2.491108000	2.284466000	0.652819000
S	-2.483222000	-0.928636000	-1.146182000
O	-1.750031000	-2.226756000	-1.335330000
H	-2.377378000	4.008772000	0.293039000
C	-3.971010000	-1.313843000	-0.019215000
C	-4.782060000	-2.298735000	-0.878568000
H	-4.229150000	-3.222643000	-1.063279000
H	-5.700052000	-2.547330000	-0.336658000
H	-5.072315000	-1.861484000	-1.839696000
C	-3.485728000	-1.974256000	1.269460000
H	-3.006723000	-1.257953000	1.940687000
H	-4.349397000	-2.398886000	1.791705000
H	-2.790860000	-2.791900000	1.055098000
C	-4.735628000	-0.009150000	0.214397000
H	-5.082956000	0.425043000	-0.729057000
H	-5.621654000	-0.239800000	0.816061000
H	-4.136622000	0.739769000	0.738807000
C	2.904386000	0.283453000	-1.336518000
C	3.843908000	0.989194000	-0.557566000
C	3.330402000	-0.899916000	-1.968641000
H	3.559666000	1.918624000	-0.073704000
H	2.623664000	-1.456276000	-2.579252000
C	5.146828000	0.518971000	-0.409113000
C	4.634803000	-1.370786000	-1.820624000
H	5.854247000	1.082097000	0.193135000
H	4.935914000	-2.288212000	-2.318286000
C	5.550133000	-0.664814000	-1.037068000
H	6.567568000	-1.026430000	-0.921511000
H	-0.277461000	-1.655576000	0.160770000
O	-2.157353000	4.942212000	0.061186000
H	-1.500190000	5.181160000	0.726745000

TS2

B	-1.738827000	1.642141000	0.137119000
C	-0.778205000	2.483049000	-1.053624000
C	0.574957000	2.075083000	-0.888688000
C	1.057283000	0.836231000	-1.330705000
C	-0.066667000	-0.246187000	0.083702000
N	-1.351403000	0.162215000	0.027837000
H	-0.998315000	3.524719000	-0.818905000
H	-1.248456000	2.158337000	-1.986680000
H	1.196982000	2.670780000	-0.224772000
H	0.496017000	0.361755000	-2.133665000
C	0.678365000	0.085408000	1.346496000
O	0.479948000	1.082819000	2.014910000
O	1.579061000	-0.860521000	1.617976000
C	2.397617000	-0.656918000	2.806706000
H	1.733882000	-0.608849000	3.674311000
H	2.905777000	0.306046000	2.710213000
C	3.372043000	-1.813654000	2.888297000
H	4.019890000	-1.839848000	2.007835000
H	2.843676000	-2.768027000	2.965024000
H	4.000545000	-1.696228000	3.776008000
O	-2.678829000	2.195256000	0.774430000
S	-2.376490000	-0.748720000	-1.160764000
O	-1.447602000	-1.711651000	-1.859773000
H	-2.713551000	3.951360000	0.555181000
C	-3.386395000	-1.776932000	0.069613000
C	-4.257555000	-2.654923000	-0.843663000
H	-3.647555000	-3.326467000	-1.453257000
H	-4.918171000	-3.263968000	-0.218274000
H	-4.888354000	-2.053161000	-1.506544000
C	-2.447503000	-2.626572000	0.922741000
H	-1.878342000	-2.016974000	1.628356000
H	-3.041998000	-3.342181000	1.500546000
H	-1.753649000	-3.192788000	0.294571000
C	-4.235797000	-0.803795000	0.891500000
H	-4.901819000	-0.213845000	0.253809000
H	-4.861143000	-1.383734000	1.579033000
H	-3.624503000	-0.111238000	1.473808000
C	2.475593000	0.409161000	-1.257590000
C	3.434352000	1.051305000	-0.453499000
C	2.880661000	-0.697284000	-2.024120000
H	3.160808000	1.911280000	0.149999000
H	2.152224000	-1.203920000	-2.652096000
C	4.752764000	0.598480000	-0.421062000
C	4.198709000	-1.150946000	-1.989819000
H	5.479696000	1.111515000	0.201763000
H	4.489235000	-2.006778000	-2.591906000
C	5.141105000	-0.504863000	-1.186687000
H	6.169083000	-0.853604000	-1.160103000
H	0.154488000	-1.204755000	-0.380257000
O	-2.549377000	4.910486000	0.398073000
H	-1.962235000	5.152904000	1.125252000

INT2

B	-1.861423000	1.607838000	0.348541000
C	0.179176000	2.943435000	-1.766034000
C	0.828812000	2.121447000	-0.938011000
C	0.861160000	0.623985000	-1.103777000
C	-0.014097000	-0.076789000	0.013574000
N	-1.418953000	0.348056000	-0.064815000
H	0.094201000	4.006088000	-1.562430000
H	-0.328643000	2.568063000	-2.652204000
H	1.311304000	2.538864000	-0.055745000
H	0.329776000	0.372531000	-2.027371000
C	0.478588000	0.248346000	1.417367000
O	0.045558000	1.192291000	2.058300000
O	1.433819000	-0.578961000	1.827158000
C	2.045878000	-0.290164000	3.120490000
H	1.273281000	-0.377730000	3.889213000
H	2.400911000	0.743505000	3.107910000
C	3.172293000	-1.283612000	3.316464000
H	3.918569000	-1.185807000	2.523245000
H	2.795287000	-2.310116000	3.318627000
H	3.660576000	-1.094266000	4.276928000
O	-2.456590000	2.643682000	0.628142000
S	-2.534836000	-0.590306000	-1.069486000
O	-1.674322000	-1.357322000	-2.041126000
H	-1.888156000	4.404824000	0.151177000
C	-3.197564000	-1.866314000	0.164039000
C	-4.257284000	-2.614047000	-0.664451000
H	-3.804979000	-3.134819000	-1.512728000
H	-4.744384000	-3.358512000	-0.026750000
H	-5.032125000	-1.937856000	-1.041005000
C	-2.088341000	-2.814477000	0.616331000
H	-1.385725000	-2.329277000	1.298883000
H	-2.535199000	-3.659116000	1.151613000
H	-1.540184000	-3.210699000	-0.243048000
C	-3.836934000	-1.097529000	1.322268000
H	-4.571400000	-0.364258000	0.971244000
H	-4.364284000	-1.805453000	1.970426000
H	-3.088339000	-0.581665000	1.928693000
C	2.249085000	-0.004603000	-1.187403000
C	3.397890000	0.605916000	-0.667834000
C	2.379958000	-1.255307000	-1.811294000
H	3.332969000	1.580438000	-0.194977000
H	1.498374000	-1.734740000	-2.230705000
C	4.643145000	-0.021732000	-0.761527000
C	3.621253000	-1.884442000	-1.904988000
H	5.523261000	0.470543000	-0.357527000
H	3.700588000	-2.850389000	-2.395502000
C	4.759493000	-1.269079000	-1.377266000
H	5.728451000	-1.753885000	-1.452424000
H	0.040798000	-1.150870000	-0.158914000
O	-1.478415000	5.274597000	-0.007486000
H	-0.823242000	5.339427000	0.698566000

TS3

B	-1.367493000	1.522164000	-0.368752000
C	-0.401555000	1.219262000	-1.949044000
C	0.946476000	0.826541000	-1.680730000
C	1.354743000	-0.459334000	-1.401184000
C	-0.235939000	-0.296055000	0.778617000
N	-1.363300000	0.013017000	0.168334000
H	-0.538605000	2.218760000	-2.361700000
H	-1.027134000	0.478698000	-2.448734000
H	1.667987000	1.632335000	-1.554317000
H	0.640108000	-1.265035000	-1.558575000
C	0.447148000	0.933303000	1.108587000
O	-0.114111000	1.979910000	0.662981000
O	1.556882000	0.920959000	1.801074000
C	2.188484000	2.211733000	2.096615000
H	1.477287000	2.804401000	2.677690000
H	2.375670000	2.721367000	1.148749000
C	3.462529000	1.918835000	2.858097000
H	4.142112000	1.307938000	2.258157000
H	3.249259000	1.396433000	3.794396000
H	3.961773000	2.862583000	3.095488000
O	-2.397202000	2.287342000	-0.484784000
S	-2.315290000	-1.325077000	-0.532565000
O	-1.543904000	-2.581782000	-0.218418000
H	-2.224583000	3.481573000	-1.662953000
C	-3.791499000	-1.263822000	0.660484000
C	-4.575128000	-2.538112000	0.308473000
H	-3.996235000	-3.438812000	0.526764000
H	-5.488804000	-2.559486000	0.911028000
H	-4.872025000	-2.556815000	-0.745599000
C	-3.295001000	-1.302694000	2.104979000
H	-2.814404000	-0.365696000	2.395326000
H	-4.152422000	-1.462975000	2.767144000
H	-2.593857000	-2.128905000	2.258017000

C	-4.586742000	0.001409000	0.323394000
H	-5.000277000	-0.051517000	-0.689098000
H	-5.428245000	0.074531000	1.021251000
H	-3.982402000	0.911126000	0.393642000
C	2.706450000	-0.898708000	-1.054676000
C	3.794151000	-0.015684000	-0.885712000
C	2.940795000	-2.277742000	-0.876260000
H	3.657669000	1.051831000	-1.025765000
H	2.114810000	-2.973215000	-1.001120000
C	5.058121000	-0.497163000	-0.554137000
C	4.205365000	-2.757417000	-0.543000000
H	5.882821000	0.199684000	-0.435365000
H	4.359774000	-3.824402000	-0.411937000
C	5.271014000	-1.868803000	-0.379215000
H	6.258323000	-2.239498000	-0.120627000
H	0.080544000	-1.302824000	1.016268000
O	-1.982455000	4.161844000	-2.347991000
H	-1.414093000	4.767723000	-1.856308000

INT3

B	1.649430000	1.592523000	0.350971000
C	-0.246421000	-0.088421000	3.374576000
C	-0.933100000	0.178194000	2.262465000
C	-0.935191000	-0.718752000	1.050209000
C	-0.067194000	-0.093516000	-0.130031000
N	1.327570000	0.168721000	0.213289000
H	-0.257575000	0.588059000	4.224136000
H	0.354010000	-0.989714000	3.464405000
H	-1.506048000	1.103920000	2.207872000
H	-0.373601000	-1.622748000	1.298321000
C	-0.517911000	1.304328000	-0.468754000
O	0.292450000	2.231857000	-0.183721000
O	-1.674750000	1.514039000	-0.988656000
C	-2.094704000	2.912356000	-1.255128000
H	-1.340548000	3.354991000	-1.908093000
H	-2.091615000	3.433855000	-0.296422000
C	-3.464203000	2.845229000	-1.889249000
H	-4.180268000	2.354974000	-1.225201000
H	-3.432639000	2.306432000	-2.839563000
H	-3.811057000	3.864447000	-2.081840000
O	2.632241000	2.296125000	0.708805000
S	2.499941000	-1.014182000	0.635945000
O	1.790304000	-2.198859000	1.264034000
H	2.514992000	4.052969000	0.667668000
C	3.109399000	-1.647234000	-1.045721000
C	4.276689000	-2.571173000	-0.657849000
H	3.930240000	-3.413604000	-0.053237000
H	4.739048000	-2.968298000	-1.567465000
H	5.048597000	-2.033409000	-0.097026000
C	2.011055000	-2.434346000	-1.760673000
H	1.257025000	-1.775554000	-2.200252000
H	2.449929000	-3.018004000	-2.577320000
H	1.522995000	-3.128370000	-1.070065000
C	3.602399000	-0.442764000	-1.849702000
H	4.327538000	0.153206000	-1.285696000
H	4.097541000	-0.796612000	-2.760589000
H	2.777158000	0.209724000	-2.146258000
C	-2.314828000	-1.138548000	0.548453000
C	-3.498492000	-0.491641000	0.928768000
C	-2.409278000	-2.237006000	-0.321722000
H	-3.467302000	0.345066000	1.618144000
H	-1.506821000	-2.771135000	-0.610030000
C	-4.736828000	-0.921257000	0.443605000
C	-3.642920000	-2.665939000	-0.810655000
H	-5.641565000	-0.407724000	0.756423000
H	-3.689795000	-3.520167000	-1.479948000
C	-4.814346000	-2.005555000	-0.431125000
H	-5.777299000	-2.339128000	-0.806494000
H	-0.189513000	-0.733627000	-1.008123000
O	2.439427000	5.026294000	0.538397000
H	2.925249000	5.174323000	-0.282025000

RC4

B	1.194225000	1.286784000	1.369496000
C	0.223156000	0.439867000	2.439014000
C	-1.117328000	0.136362000	1.895323000
C	-1.444736000	-0.975465000	1.178515000
C	0.430083000	0.357406000	-0.672294000
N	1.432528000	0.277763000	0.151329000
H	0.141931000	1.088854000	3.318739000
H	0.775153000	-0.461001000	2.726836000
H	-1.877861000	0.898924000	2.051024000
H	-0.680631000	-1.740515000	1.042880000
C	-0.266339000	1.620470000	-0.413076000
O	0.214910000	2.259236000	0.577292000
O	-1.224993000	2.025201000	-1.168729000
C	-1.865483000	3.331706000	-0.873542000
H	-1.084756000	4.090717000	-0.954142000
H	-2.223310000	3.287671000	0.156746000
C	-2.977511000	3.512984000	-1.878863000
H	-3.721879000	2.718164000	-1.787996000
H	-2.588193000	3.526227000	-2.899699000
H	-3.469047000	4.470526000	-1.685009000
O	2.329850000	1.923987000	1.832277000
S	2.366099000	-1.291111000	0.171413000
O	1.651164000	-2.160561000	-0.818260000
H	2.337756000	2.070036000	2.784295000
C	3.966941000	-0.686569000	-0.674498000
C	4.735033000	-2.003696000	-0.876225000
H	4.205925000	-2.680865000	-1.550523000
H	5.705295000	-1.763101000	-1.321598000
H	4.924151000	-2.519409000	0.070924000
C	3.624664000	-0.029566000	-2.009518000
H	3.162506000	0.951730000	-1.877976000
H	4.551704000	0.109887000	-2.574846000
H	2.964047000	-0.665581000	-2.605966000
C	4.689271000	0.241543000	0.303092000
H	4.909127000	-0.262746000	1.249452000
H	5.645525000	0.527065000	-0.147716000
H	4.123933000	1.150061000	0.517476000
C	-2.749139000	-1.312141000	0.606637000
C	-3.877765000	-0.470523000	0.696444000
C	-2.884209000	-2.541538000	-0.068459000
H	-3.811047000	0.481827000	1.212890000
H	-2.023160000	-3.199806000	-0.148074000
C	-5.091471000	-0.851325000	0.133284000
C	-4.100744000	-2.921265000	-0.631293000
H	-5.951445000	-0.193378000	0.214213000
H	-4.184027000	-3.873792000	-1.145492000
C	-5.208940000	-2.077057000	-0.532671000
H	-6.158777000	-2.369903000	-0.969482000
H	0.190332000	-0.372123000	-1.438227000

TS4

B	1.218512000	1.590997000	1.023877000
C	0.243367000	0.568118000	2.621112000
C	-1.008177000	0.259335000	2.083031000
C	-1.251073000	-0.856515000	1.277456000
C	0.319604000	0.156141000	-0.541196000
N	1.432138000	0.354430000	0.200882000
H	0.315028000	1.407564000	3.306852000
H	0.968707000	-0.224266000	2.784882000
H	-1.795157000	1.000210000	2.199307000
H	-0.463688000	-1.604475000	1.212821000
C	-0.435444000	1.353490000	-0.484855000
O	0.088580000	2.227798000	0.343861000
O	-1.517501000	1.588442000	-1.159992000
C	-2.166253000	2.908767000	-1.034088000
H	-1.431632000	3.662134000	-1.327318000
H	-2.427135000	3.053766000	0.016143000

C	-3.377196000	2.889943000	-1.938191000
H	-4.076086000	2.105058000	-1.638478000
H	-3.087526000	2.731376000	-2.979909000
H	-3.886333000	3.854947000	-1.864220000
O	2.130682000	2.436018000	1.582868000
S	2.550964000	-1.028647000	0.468473000
O	1.794784000	-2.232313000	-0.020489000
H	2.801339000	2.031881000	2.144466000
C	3.852221000	-0.615810000	-0.848319000
C	4.837438000	-1.790546000	-0.722621000
H	4.362244000	-2.740828000	-0.978206000
H	5.664832000	-1.623431000	-1.419112000
H	5.259855000	-1.865632000	0.284803000
C	3.203652000	-0.579064000	-2.230269000
H	2.561228000	0.295625000	-2.359183000
H	3.993661000	-0.527429000	-2.986377000
H	2.621162000	-1.486025000	-2.414820000
C	4.505741000	0.709262000	-0.448064000
H	4.944435000	0.660023000	0.554054000
H	5.318499000	0.919757000	-1.150458000
H	3.806143000	1.547832000	-0.487588000
C	-2.517386000	-1.232001000	0.675309000
C	-3.676707000	-0.426118000	0.737568000
C	-2.592113000	-2.467486000	-0.005913000
H	-3.658412000	0.523485000	1.261587000
H	-1.706965000	-3.095032000	-0.062018000
C	-4.861436000	-0.848133000	0.144158000
C	-3.778623000	-2.884631000	-0.599119000
H	-5.746745000	-0.223135000	0.206083000
H	-3.819087000	-3.837896000	-1.116410000
C	-4.916881000	-2.075608000	-0.526465000
H	-5.844474000	-2.399782000	-0.987989000
H	0.136372000	-0.717806000	-1.147236000

INT4

B	1.297586000	2.075890000	0.458955000
C	-0.074628000	-0.047684000	3.349607000
C	-0.848578000	0.128622000	2.276786000
C	-0.706163000	-0.672118000	1.005941000
C	0.006411000	0.187108000	-0.138625000
N	1.347629000	0.677000000	0.222058000
H	-0.209456000	0.550355000	4.245938000
H	0.712683000	-0.796380000	3.363721000
H	-1.619428000	0.897986000	2.306254000
H	0.020752000	-1.468593000	1.183799000
C	-0.697575000	1.497114000	-0.356392000
O	-0.028826000	2.532178000	0.035148000
O	-1.864263000	1.581040000	-0.840398000
C	-2.546131000	2.914731000	-0.970891000
H	-1.860540000	3.558826000	-1.522224000
H	-2.672793000	3.286801000	0.046471000
C	-3.848231000	2.662089000	-1.687982000
H	-4.478060000	1.967294000	-1.127923000
H	-3.676590000	2.266715000	-2.691708000
H	-4.378079000	3.614674000	-1.778470000
O	2.137203000	3.002573000	0.917025000
S	2.766245000	-0.278465000	0.611840000
O	2.320637000	-1.531351000	1.318422000
H	2.995647000	2.681343000	1.223635000
C	3.389586000	-0.823897000	-1.092897000
C	4.748422000	-1.461545000	-0.746488000
H	4.626818000	-2.341395000	-0.109403000
H	5.233512000	-1.777435000	-1.675033000
H	5.415808000	-0.753082000	-0.244856000
C	2.447167000	-1.857242000	-1.709487000
H	1.553838000	-1.396970000	-2.140008000
H	2.966261000	-2.377547000	-2.520883000
H	2.149317000	-2.602523000	-0.966598000
C	3.570421000	0.428263000	-1.952266000
H	4.195576000	1.177887000	-1.455018000
H	4.075208000	0.146500000	-2.882045000
H	2.614501000	0.887086000	-2.217440000
C	-1.989910000	-1.302173000	0.472395000
C	-3.269155000	-0.858781000	0.835201000
C	-1.885026000	-2.386080000	-0.414461000
H	-3.391321000	-0.040822000	1.536809000
H	-0.902740000	-2.762535000	-0.690108000
C	-4.410710000	-1.475906000	0.316553000
C	-3.022729000	-3.000471000	-0.935975000
H	-5.392851000	-1.121999000	0.615791000
H	-2.917659000	-3.840136000	-1.616460000
C	-4.292519000	-2.544350000	-0.572828000
H	-5.180708000	-3.023822000	-0.972969000
H	-0.002867000	-0.396843000	-1.060616000

RC5

B	2.033069000	1.664312000	0.738548000
C	0.361569000	-1.563868000	2.923950000
C	-0.317536000	-0.728360000	2.136440000
C	-0.557216000	-0.970616000	0.664249000
C	-0.099814000	0.251511000	-0.216761000
N	1.307431000	0.610303000	0.033327000
H	0.494027000	-1.361852000	3.982457000
H	0.792964000	-2.488058000	2.545767000
H	-0.739987000	0.178683000	2.563604000
H	0.084963000	-1.801632000	0.351335000
C	-0.983595000	1.479060000	0.036643000
O	-0.830849000	2.245742000	0.978909000
O	-1.948035000	1.574752000	-0.859145000
C	-2.936464000	2.641191000	-0.669720000
H	-2.415429000	3.597454000	-0.760896000
H	-3.331978000	2.555748000	0.344884000
C	-4.004177000	2.455061000	-1.726149000
H	-4.496020000	1.484901000	-1.616254000
H	-3.580803000	2.525598000	-2.731693000
H	-4.758583000	3.239215000	-1.615123000
S	2.625729000	0.488247000	-1.046081000
O	3.274713000	1.594647000	0.006052000
C	3.632708000	-1.052482000	-0.678704000
C	4.970470000	-0.735489000	-1.374498000
H	5.474026000	0.121423000	-0.919989000
H	5.617116000	-1.609726000	-1.251395000
H	4.852299000	-0.560092000	-2.448012000
C	3.787154000	-1.259069000	0.824571000
H	2.824913000	-1.363614000	1.330900000
H	4.348232000	-2.186574000	0.974231000
H	4.351453000	-0.447381000	1.288168000
C	2.885579000	-2.197197000	-1.379133000
H	2.696080000	-1.990190000	-2.437003000
H	3.520229000	-3.086938000	-1.327926000
H	1.940993000	-2.437273000	-0.885063000
C	-1.996873000	-1.356007000	0.313768000
C	-3.089086000	-0.971489000	1.102736000
C	-2.236699000	-2.104889000	-0.847699000
H	-2.932468000	-0.411352000	2.019006000
H	-1.399939000	-2.427386000	-1.463495000
C	-4.390827000	-1.317025000	0.730681000
C	-3.535620000	-2.450756000	-1.220794000
H	-5.225164000	-1.014479000	1.356567000
H	-3.700455000	-3.034914000	-2.121326000
C	-4.619031000	-2.053775000	-0.432851000
H	-5.630825000	-2.325206000	-0.718468000
H	-0.211522000	-0.026158000	-1.266723000
O	1.763708000	2.524302000	1.703556000
H	0.797299000	2.553826000	1.862378000

TS5

B	1.821520000	-1.349947000	-1.210306000
C	1.203662000	-0.151619000	-2.353760000
C	-0.184271000	0.036645000	-2.067305000

C	-0.660663000	0.921641000	-1.094369000
C	0.102733000	-0.379386000	0.571507000
N	1.319703000	-0.762665000	0.167158000
H	1.399822000	-0.657386000	-3.300735000
H	1.816189000	0.738247000	-2.231619000
H	-0.887841000	-0.652133000	-2.528837000
H	0.025631000	1.700097000	-0.768813000
C	-0.976748000	-1.429622000	0.510561000
O	-0.953667000	-2.393291000	-0.237266000
O	-1.936111000	-1.147744000	1.380244000
C	-3.060290000	-2.083848000	1.453330000
H	-2.664566000	-3.054849000	1.761730000
H	-3.481487000	-2.182925000	0.450448000
C	-4.053184000	-1.518620000	2.445941000
H	-4.426006000	-0.545847000	2.114509000
H	-3.601309000	-1.407150000	3.435297000
H	-4.902597000	-2.202394000	2.531529000
S	2.941312000	-0.542510000	0.664003000
O	3.303405000	-1.047835000	-0.823554000
C	3.552590000	1.240507000	0.811965000
C	5.064185000	0.996659000	1.001622000
H	5.521306000	0.556134000	0.111623000
H	5.531878000	1.971174000	1.173622000
H	5.281269000	0.366998000	1.869392000
C	3.273289000	2.090073000	-0.420881000
H	2.210616000	2.302361000	-0.550535000
H	3.782385000	3.049963000	-0.285731000
H	3.672184000	1.627720000	-1.325957000
C	2.902792000	1.781484000	2.092997000
H	3.056364000	1.119096000	2.950611000
H	3.378868000	2.737940000	2.329892000
H	1.832898000	1.967736000	1.970692000
C	-2.083293000	1.224979000	-0.844720000
C	-3.138055000	0.436161000	-1.341735000
C	-2.398571000	2.359790000	-0.075681000
H	-2.934337000	-0.445009000	-1.941689000
H	-1.595584000	2.981477000	0.311781000
C	-4.462714000	0.776747000	-1.074689000
C	-3.723644000	2.698859000	0.190610000
H	-5.264349000	0.160830000	-1.470534000
H	-3.945929000	3.580867000	0.783255000
C	-4.761173000	1.907053000	-0.307326000
H	-5.794653000	2.170272000	-0.104347000
H	0.003826000	0.367542000	1.352622000
O	1.623348000	-2.661633000	-1.565551000
H	0.703231000	-2.923196000	-1.400205000

INT5

B	2.033069000	1.664407000	0.738448000
C	0.361231000	-1.564046000	2.924043000
C	-0.317584000	-0.728302000	2.136523000
C	-0.557181000	-0.970502000	0.664303000
C	-0.099757000	0.251529000	-0.216787000
N	1.307482000	0.610365000	0.033230000
H	0.493595000	-1.362120000	3.982578000
H	0.792452000	-2.488307000	2.545837000
H	-0.739910000	0.178791000	2.563698000
H	0.085015000	-1.801516000	0.351427000
C	-0.983550000	1.479097000	0.036539000
O	-0.830933000	2.245681000	0.978909000
O	-1.947820000	1.574903000	-0.859399000
C	-2.936320000	2.641296000	-0.670038000
H	-2.415451000	3.597570000	-0.762105000
H	-3.331241000	2.556436000	0.344840000
C	-4.004589000	2.454395000	-1.725770000
H	-4.496268000	1.484252000	-1.614988000
H	-3.581765000	2.524334000	-2.731587000
H	-4.759008000	3.238548000	-1.614835000
S	2.625864000	0.488259000	-1.046088000
O	3.274793000	1.594649000	0.006085000
C	3.632722000	-1.052523000	-0.678615000
C	4.970573000	-0.735644000	-1.374278000
H	5.474160000	0.121216000	-0.919705000
H	5.617136000	-1.609936000	-1.251119000
H	4.852536000	-0.560222000	-2.447801000
C	3.786988000	-1.259102000	0.824676000
H	2.824681000	-1.363415000	1.330931000
H	4.347837000	-2.186729000	0.974433000
H	4.351408000	-0.447513000	1.288290000
C	2.885551000	-2.197154000	-1.379130000
H	2.696319000	-1.990165000	-2.437051000
H	3.520034000	-3.087005000	-1.327744000
H	1.940823000	-2.437056000	-0.885248000
C	-1.996824000	-1.355926000	0.313795000
C	-3.089078000	-0.971232000	1.102627000
C	-2.236604000	-2.104978000	-0.847570000
H	-2.932506000	-0.410967000	2.018825000
H	-1.399822000	-2.427612000	-1.463263000
C	-4.390806000	-1.316776000	0.730541000
C	-3.535517000	-2.450854000	-1.220696000
H	-5.225172000	-1.014103000	1.356327000
H	-3.700315000	-3.035150000	-2.121146000
C	-4.618963000	-2.053705000	-0.432891000
H	-5.630748000	-2.325143000	-0.718532000
H	-0.211472000	-0.026230000	-1.266723000
O	1.763608000	2.524515000	1.703324000
H	0.797214000	2.553896000	1.862241000

Reference:

[1] (a) Raducan, M.; Alam, R.; Szabó, K. J. *Angew. Chem. Int. Ed.* **2012**, *51*, 13050–13053; (b) Tan, Q.; Wang, X.; Xiong, Y.; Zhao, Z.; Li, L.; Tang, P.; Zhang, M. *Angew. Chem. Int. Ed.* **2017**, *56*, 4829–4833; (c) Karan, G.; Sahu, S.; Maji, M. S. *Chem. Commun.* **2021**, *57*, 5274–5277.

[2] Liu, M.; Shen, A.; Sun, X. W.; Deng, F.; Xu, M. H.; Lin, G. Q. *Chem. Commun.* **2010**, *46*, 8460–8462.

[3] Gaussian 09 (Revision A.02), M. J. Frisch, G. W. Trucks, H. B. Schlegel, G. E. Scuseria, M. A. Robb, J. R. Cheeseman, G. Scalmani, V. Barone, B. Mennucci, G. A. Petersson, et. al, Gaussian, Inc., Wallingford CT, 2009.

[4] A. D. Becke, *J. Chem. Phys.*, **1993**, *98*, 5648−5652.

[5] W. J. Hehre, R. Ditchfield, J. A. Pople, *J. Chem. Phys.,* **1972**, *56*, 2257−2261.

[6] (a) M. Cossi, N. Rega, G. Scalmani, V. Barone, *J. Comput. Chem.* **2003**, *24*, 669–681; (b) V. Borane, M. Cossi, *J. Phys. Chem. A.* **1998**, *102*, 1995–2001.

[7] S. Grimme, J. Antony, S. Ehrlich, H. A. Krieg, *J. Chem. Phys.* **2010**, *132*, 154104(1−19).

[8] R. Alam, A. Das, G. Huang, L. Eriksson, F. Himo, K. J. Szabó, Chem. Sci., **2014**, *5*, 2732-2738.

^{1}H and ^{13}C NMR Spectra of All Compounds:

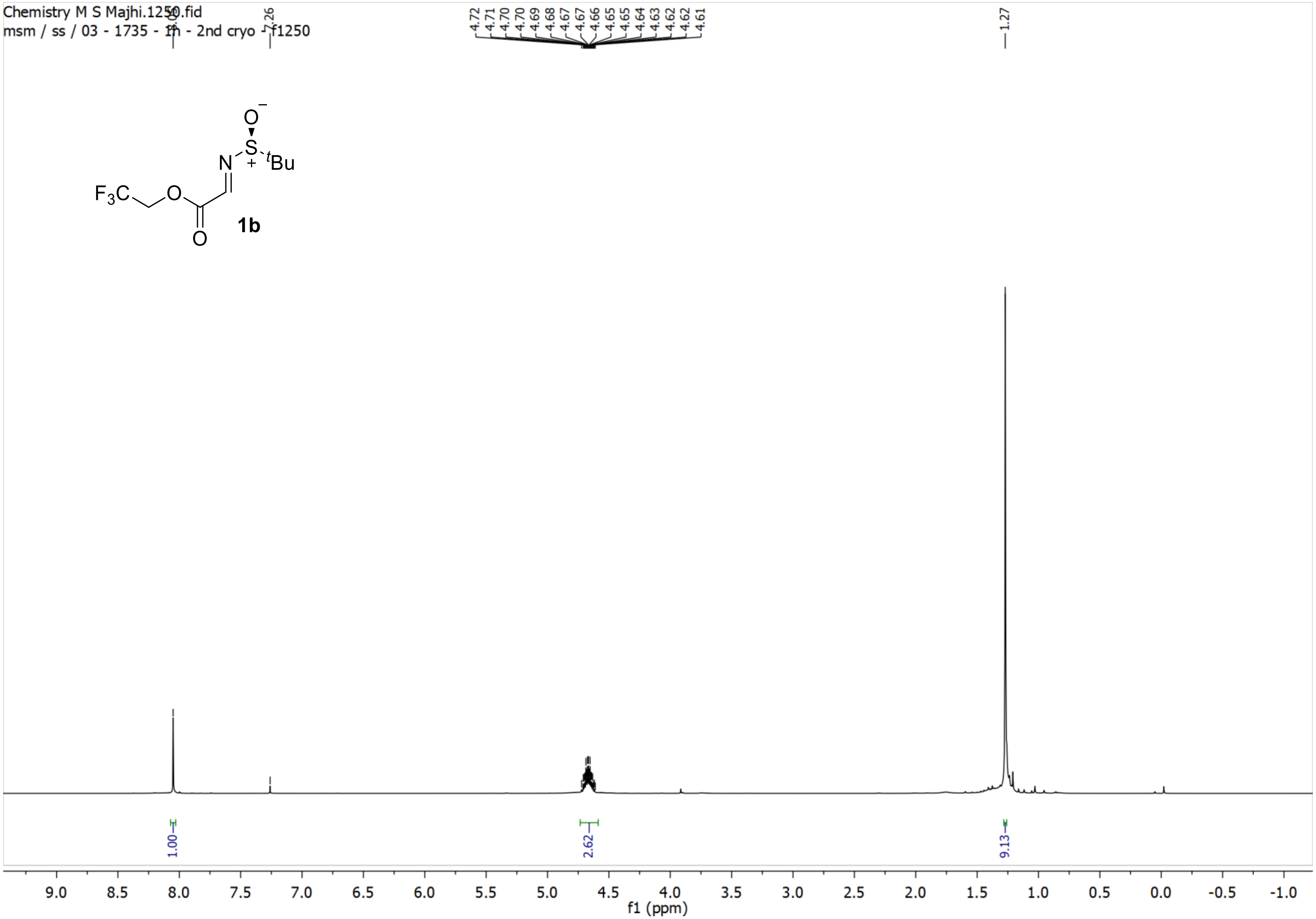

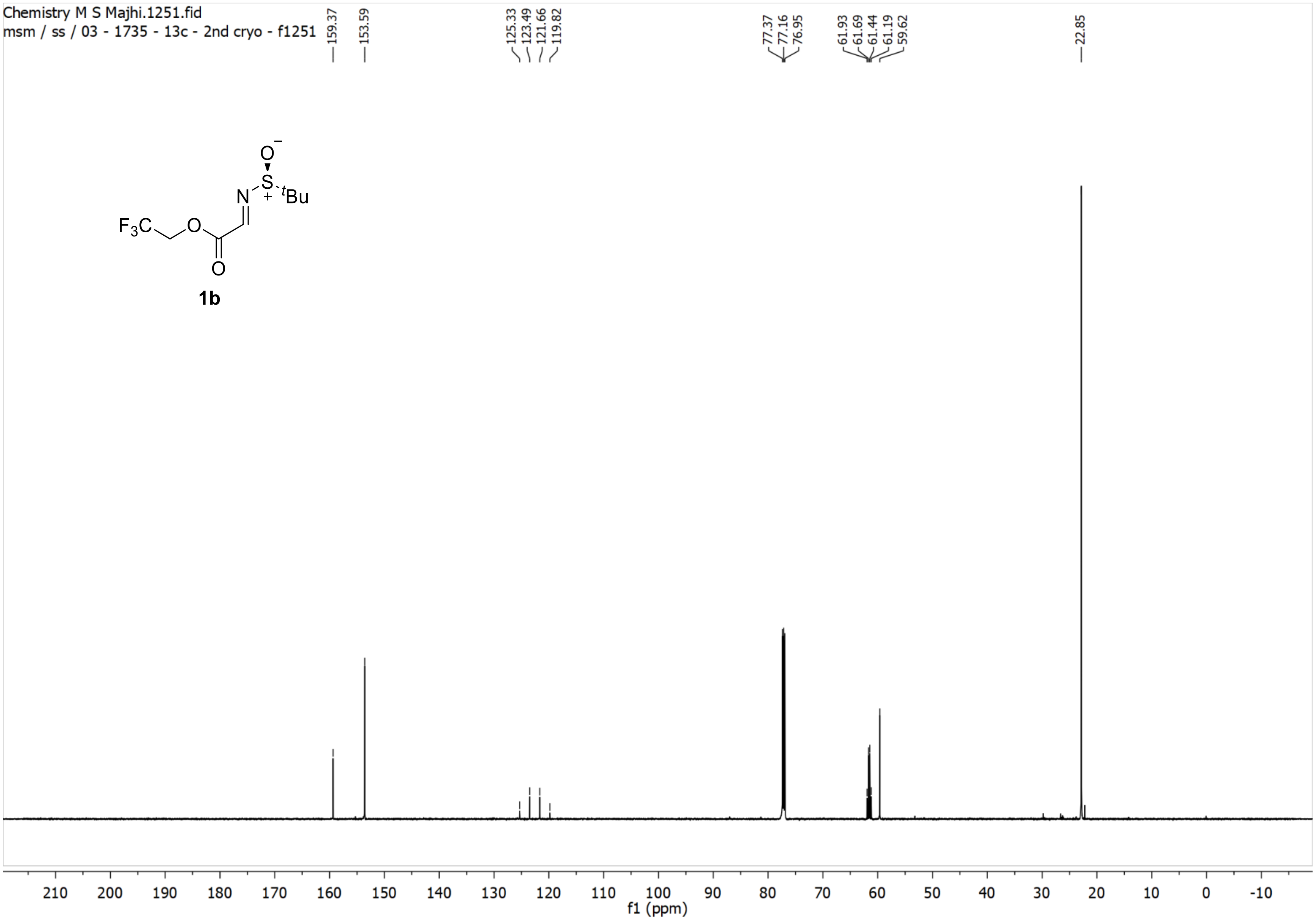
Chemistry M S Majhi.1251.fid
msm / ss / 03 - 1735 - 13c - 2nd cryo - f1251
159.37
153.59
125.33
123.49
121.66
119.82
77.37
77.16
76.95
61.93
61.69
61.44
61.19
59.62
22.85
1b
210 200 190 180 170 160 150 140 130 120 110 100 90 80 70 60 50 40 30 20 10 0 -10
f1 (ppm)

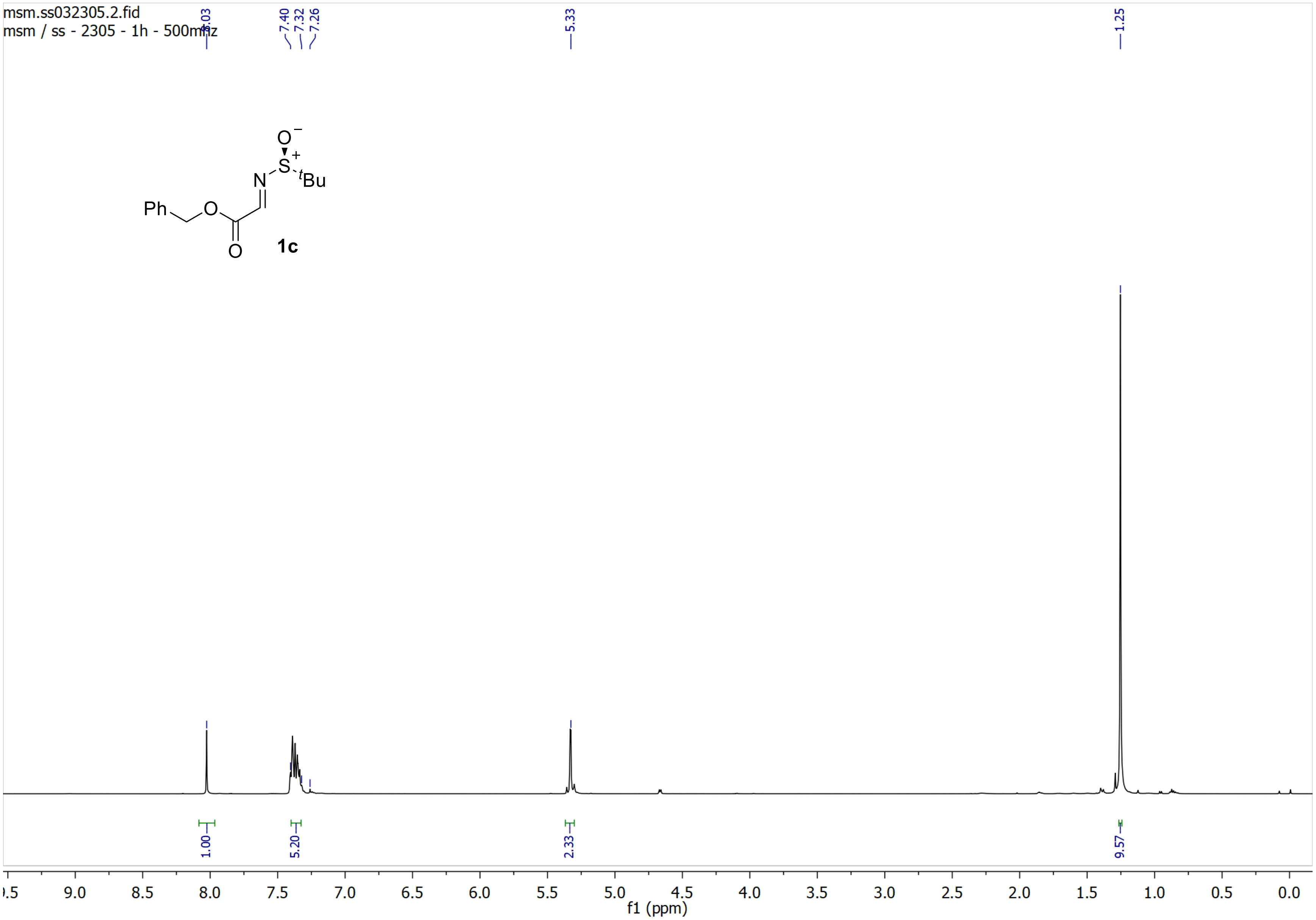
msm.ss032305.2.fid
msm / ss - 2305 - 1h - 500mhz
8.03
7.40
7.32
7.26
5.33
1.25
1c
1.00
5.20
2.33
9.57
f1 (ppm)

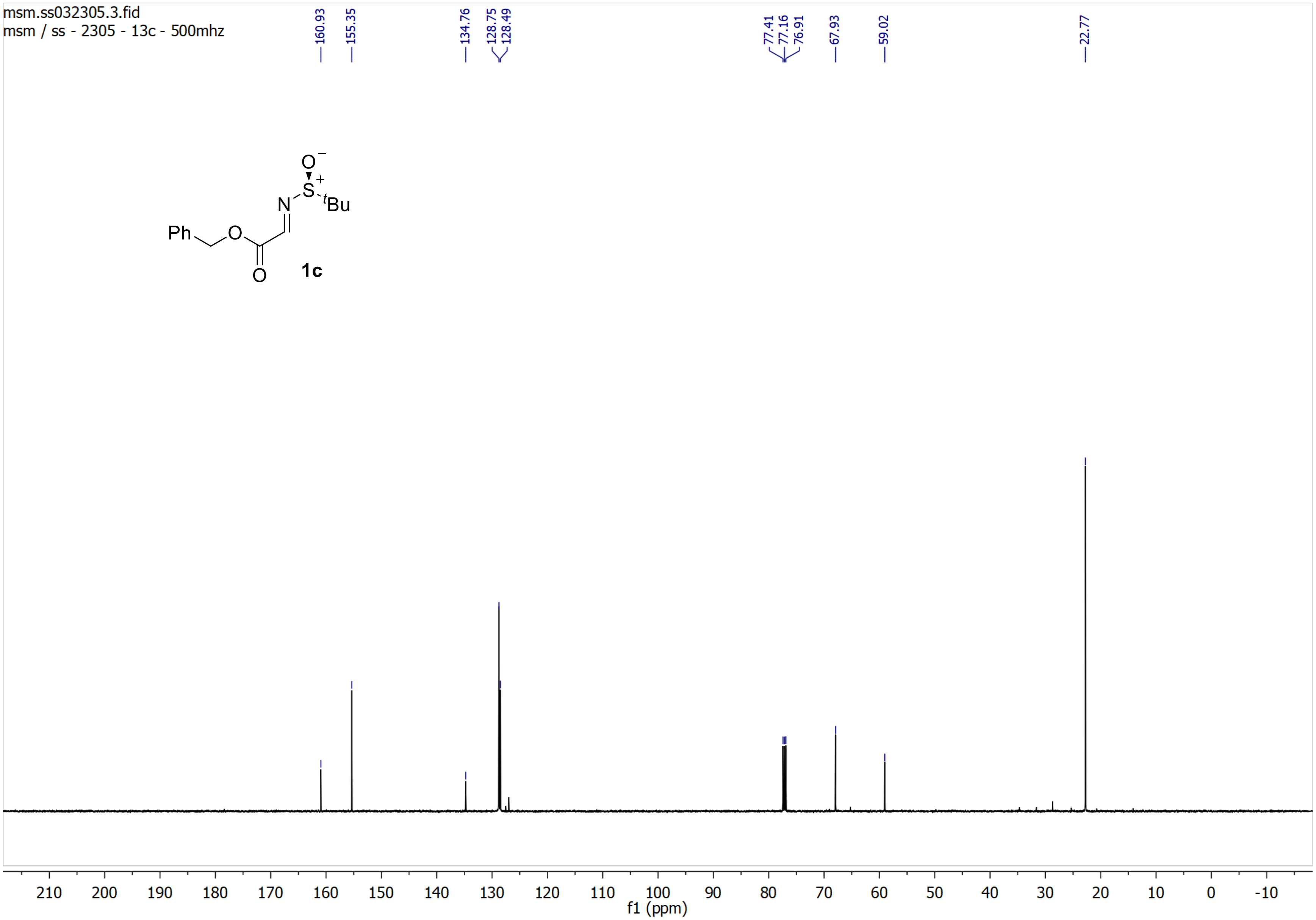
msm.ss032305.3.fid
msm / ss - 2305 - 13c - 500mhz
160.93
155.35
134.76
128.75
128.49
77.41
77.16
76.91
67.93
59.02
22.77
Ph
O
O
N
S
+
tBu
1c
210 200 190 180 170 160 150 140 130 120 110 100 90 80 70 60 50 40 30 20 10 0 -10
f1 (ppm)

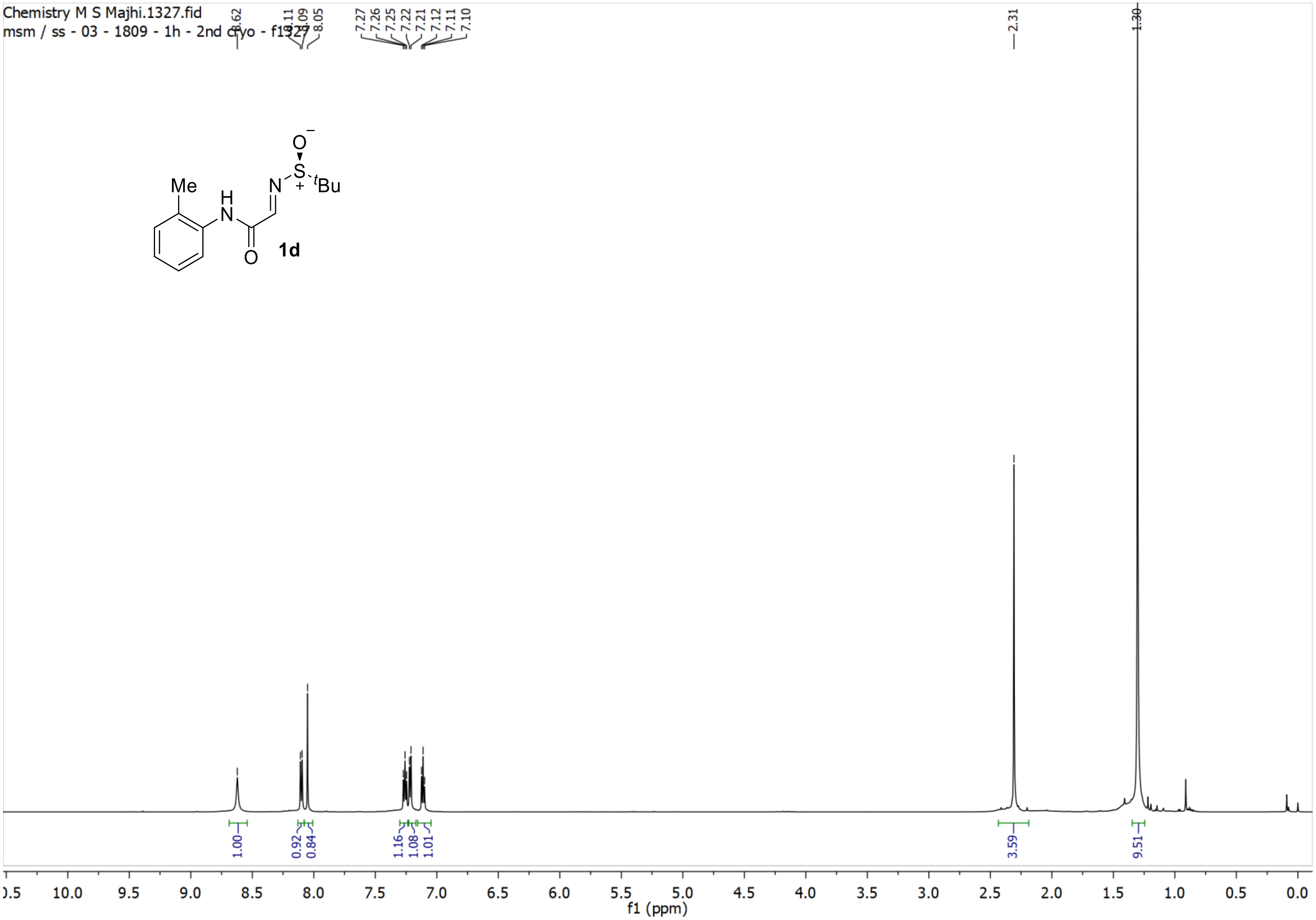
Chemistry M S Majhi.1327.fid
msm / ss - 03 - 1809 - 1h - 2nd cryo - f1327
8.62
8.11
8.09
8.05
7.27
7.26
7.25
7.22
7.21
7.12
7.11
7.10
2.31
1.30
Me
H
N
O
N
S
tBu
1d
1.00
0.92
0.84
1.16
1.08
1.01
3.59
9.51
f1 (ppm)

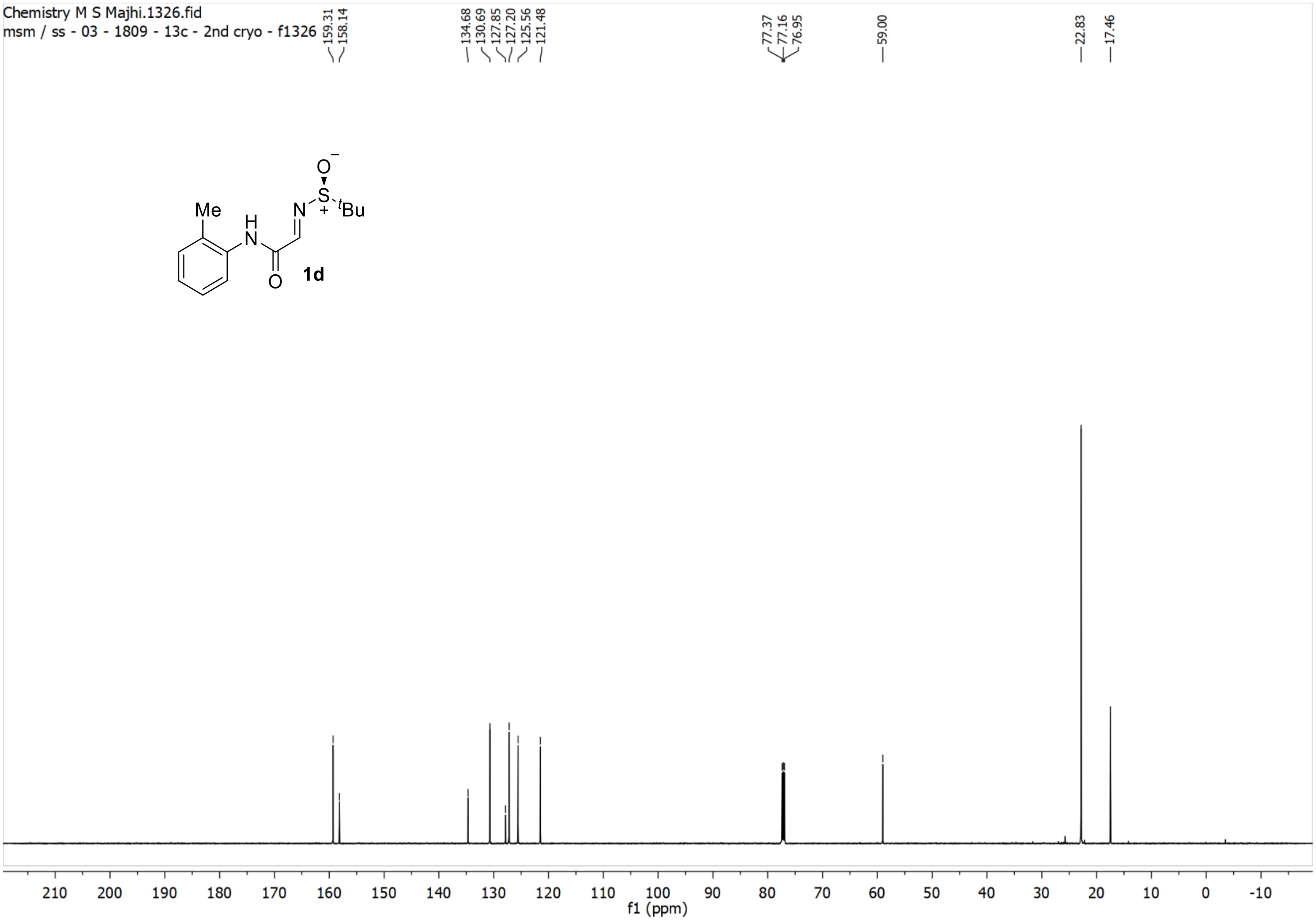
Chemistry M S Majhi.1326.fid
msm / ss - 03 - 1809 - 13c - 2nd cryo - f1326
159.31
158.14
134.68
130.69
127.85
127.20
125.56
121.48
77.37
77.16
76.95
59.00
22.83
17.46
O
S
tBu
N
Me
H
N
O
1d
210 200 190 180 170 160 150 140 130 120 110 100 90 80 70 60 50 40 30 20 10 0 -10
f1 (ppm)

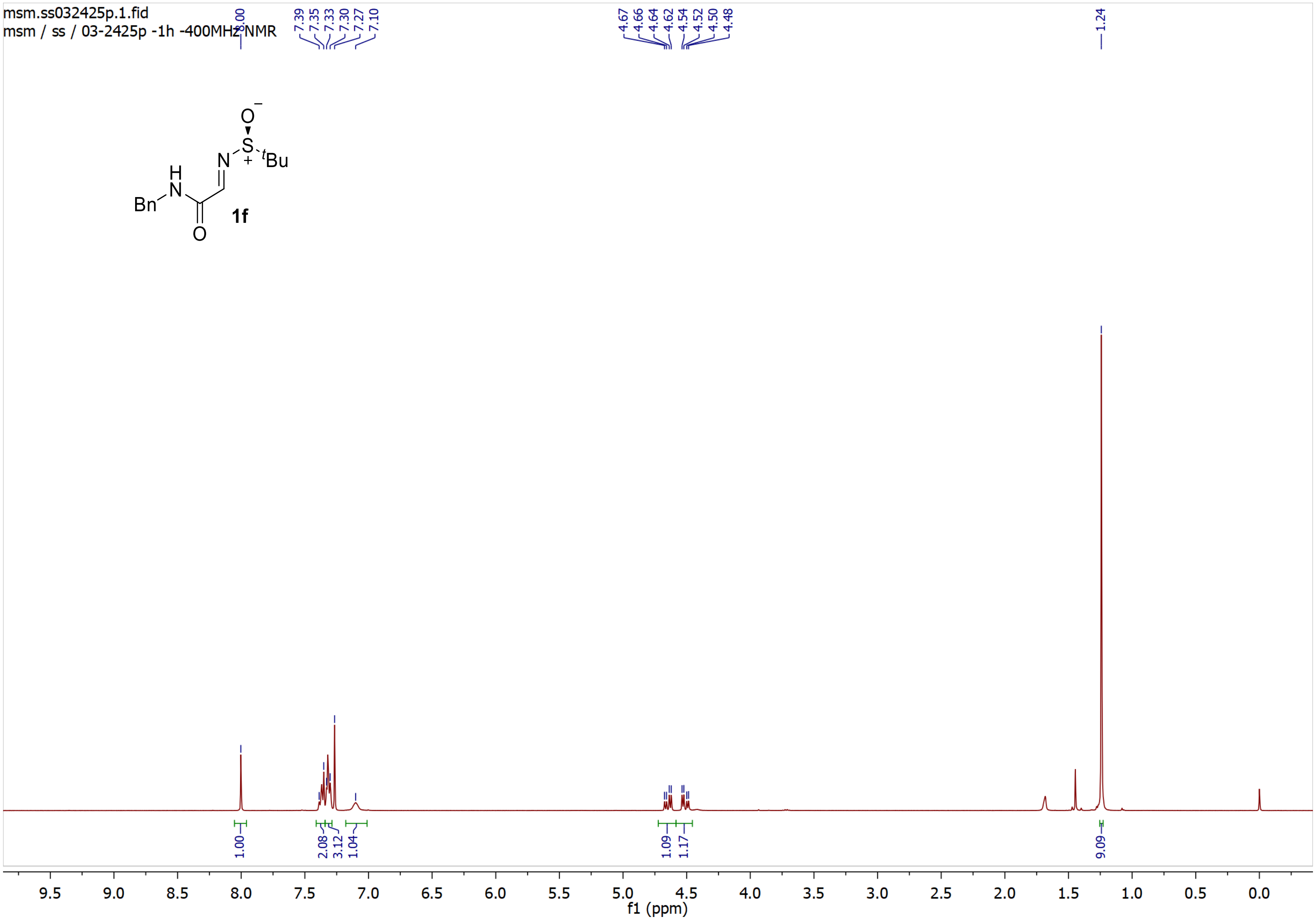
msm.ss032425p.1.fid
msm / ss / 03-2425p -1h -400MHz NMR
8.00
7.39
7.35
7.33
7.30
7.27
7.10
4.67
4.66
4.64
4.62
4.54
4.52
4.50
4.48
1.24
1f
1.00
2.08
3.12
1.04
1.09
1.17
9.09
f1 (ppm)

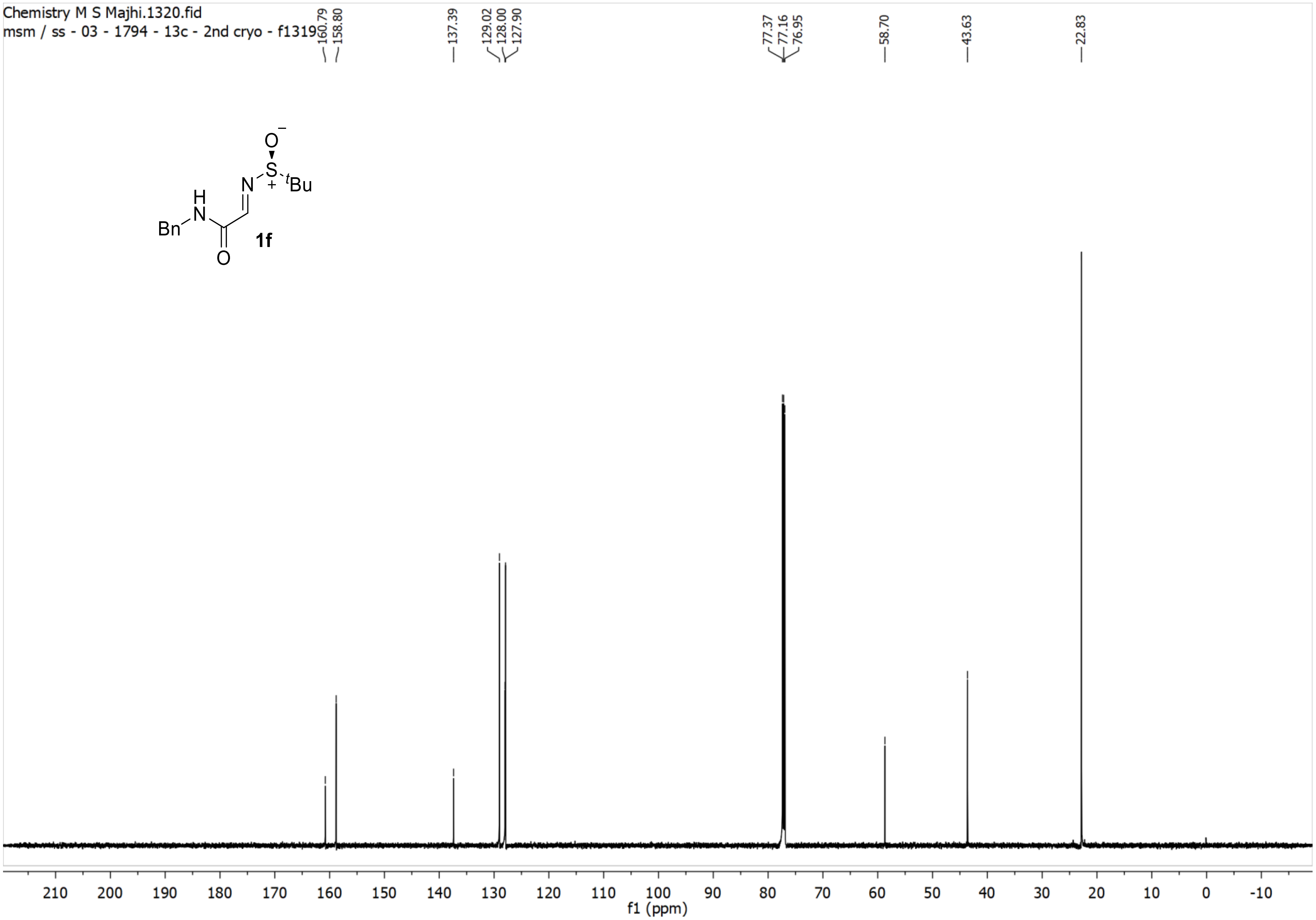

Chemistry M S Majhi.1320.fid
msm / ss - 03 - 1794 - 13c - 2nd cryo - f1319
160.79
158.80
137.39
129.02
128.00
127.90
77.37
77.16
76.95
58.70
43.63
22.83
O
S
N
tBu
H
N
Bn
O
1f
210 200 190 180 170 160 150 140 130 120 110 100 90 80 70 60 50 40 30 20 10 0 -10
f1 (ppm)

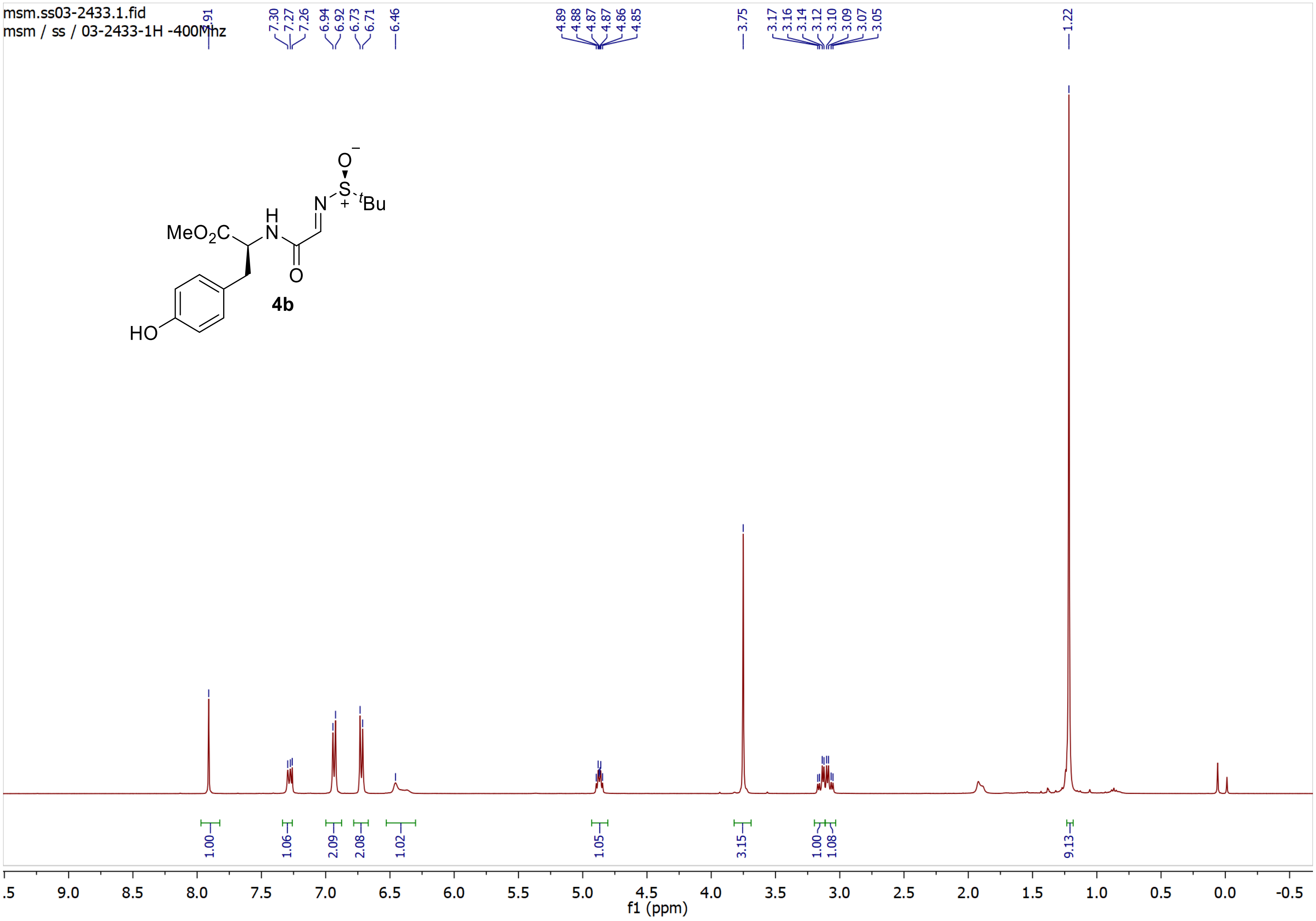
msm.ss03-2433.1.fid
msm / ss / 03-2433-1H -400Mhz
7.91
7.30
7.27
7.26
6.94
6.92
6.73
6.71
6.46
4.89
4.88
4.87
4.87
4.86
4.85
3.75
3.17
3.16
3.14
3.12
3.10
3.09
3.07
3.05
1.22
MeO2C
H
N
O
N
S
+
O
−
tBu
HO
4b
1.00
1.06
2.09
2.08
1.02
1.05
3.15
1.00
1.08
9.13
f1 (ppm)

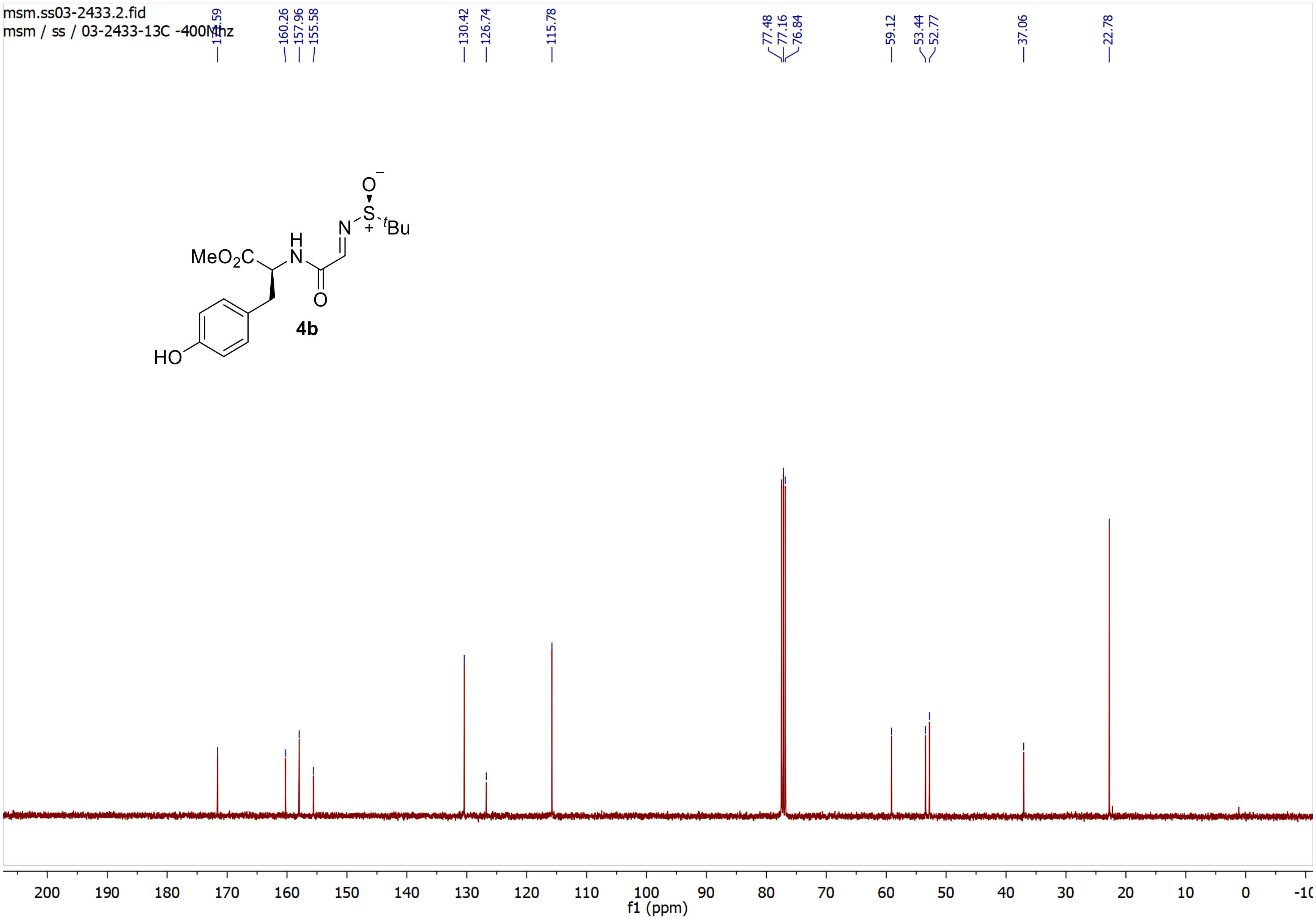
msm.ss03-2433.2.fid
msm / ss / 03-2433-13C -400Mhz
160.26
157.96
155.58
130.42
126.74
115.78
77.48
77.16
76.84
59.12
53.44
52.77
37.06
22.78
MeO2C
H
N
O
N
S
tBu
O
HO
4b
200
190
180
170
160
150
140
130
120
110
100
90
80
70
60
50
40
30
20
10
0
f1 (ppm)

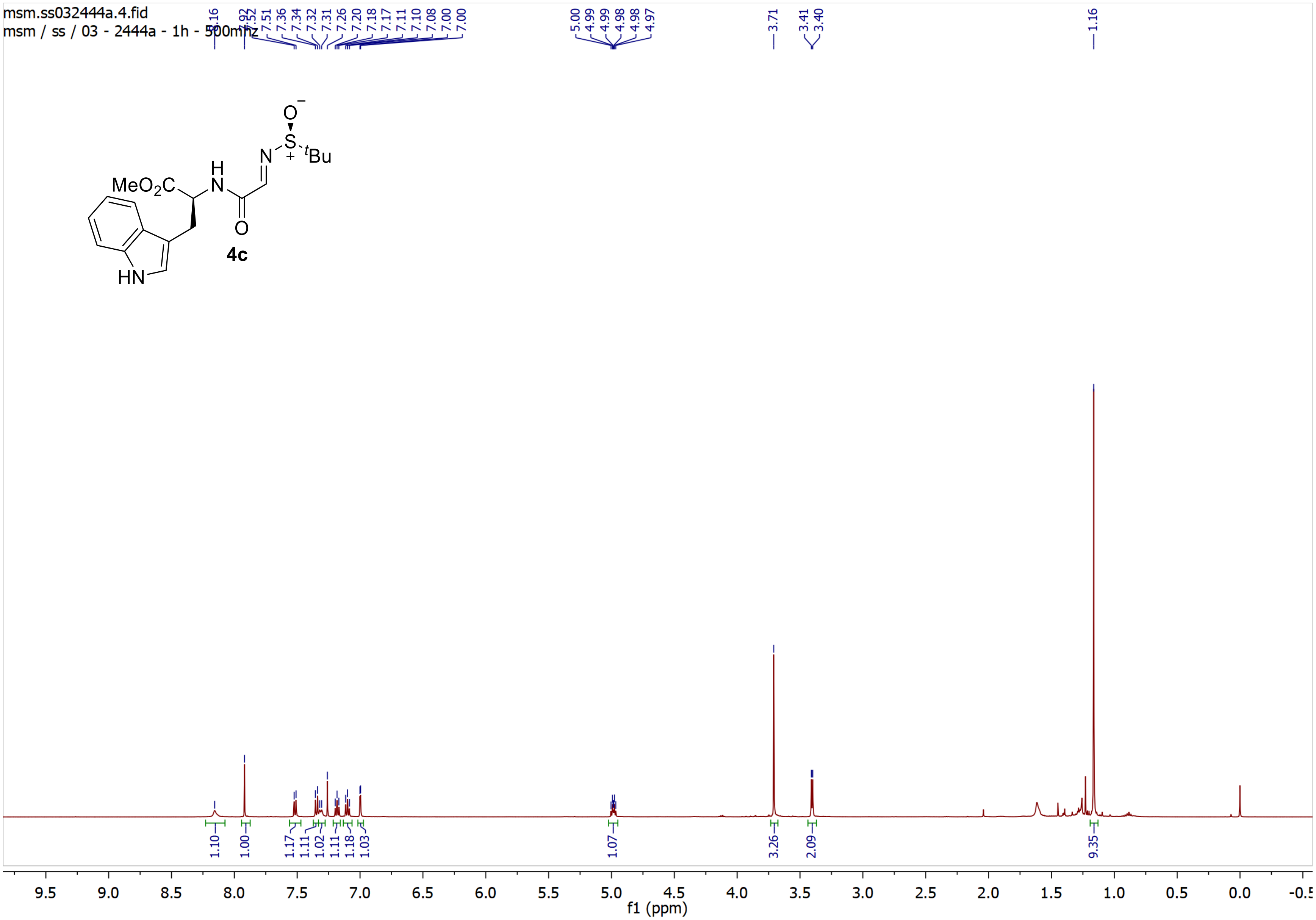
msm.ss032444a.4.fid
msm / ss / 03 - 2444a - 1h - 500mhz
8.16
7.92
7.52
7.51
7.36
7.34
7.32
7.31
7.26
7.20
7.18
7.17
7.11
7.10
7.08
7.00
7.00
5.00
4.99
4.99
4.98
4.98
4.97
3.71
3.41
3.40
1.16
MeO2C
H
N
N
O
S
+
tBu
O
HN
4c
1.10
1.00
1.17
1.11
1.02
1.11
1.18
1.03
1.07
3.26
2.09
9.35
9.5
9.0
8.5
8.0
7.5
7.0
6.5
6.0
5.5
5.0
4.5
4.0
3.5
3.0
2.5
2.0
1.5
1.0
0.5
0.0
-0.5
f1 (ppm)

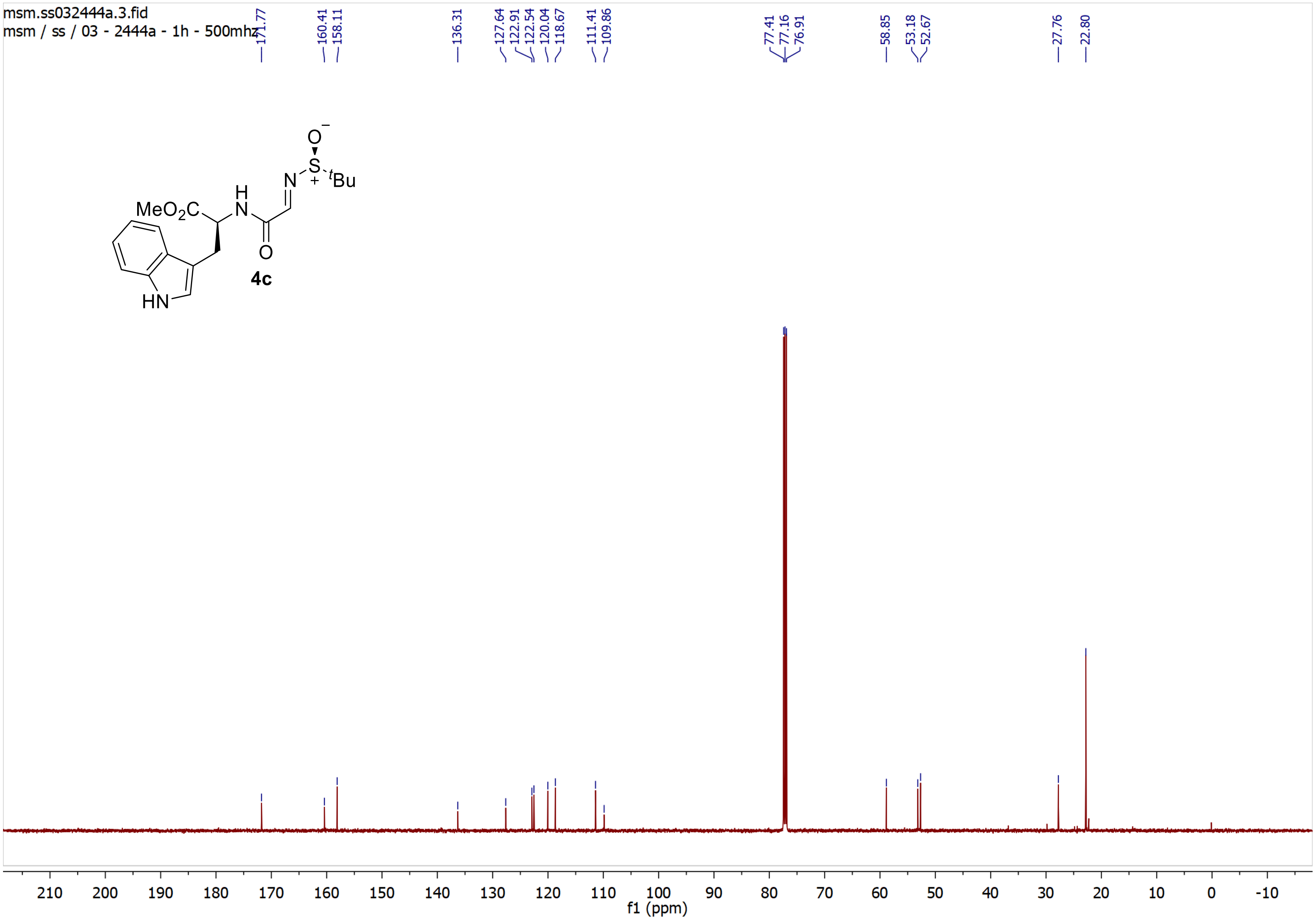
msm.ss032444a.3.fid
msm / ss / 03 - 2444a - 1h - 500mhz
171.77
160.41
158.11
136.31
127.64
122.91
122.54
120.04
118.67
111.41
109.86
77.41
77.16
76.91
58.85
53.18
52.67
27.76
22.80
MeO2C
H
N
O
N
S
+
tBu
O
HN
4c
210 200 190 180 170 160 150 140 130 120 110 100 90 80 70 60 50 40 30 20 10 0 -10
f1 (ppm)

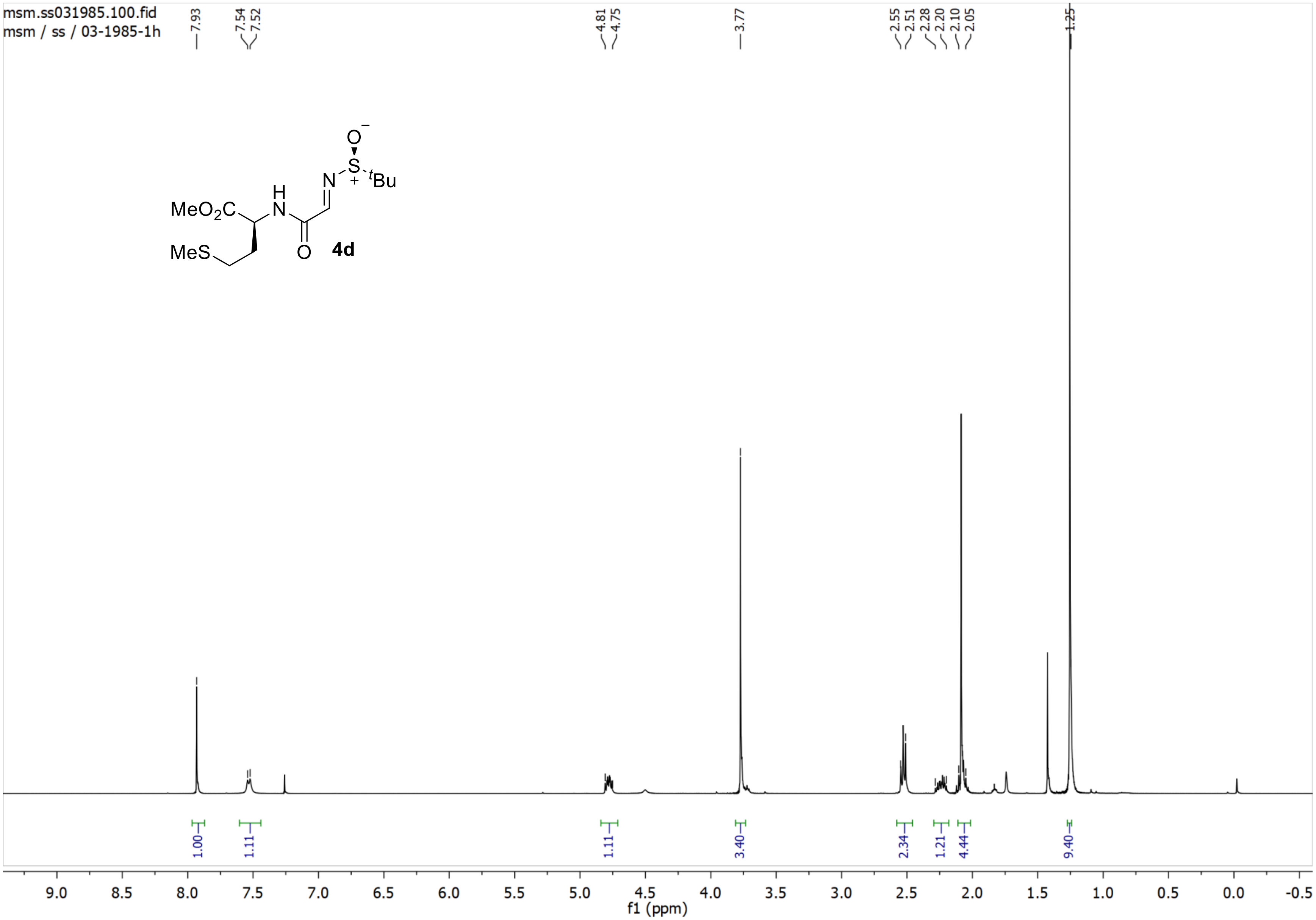

msm.ss031985.100.fid
msm / ss / 03-1985-1h
7.93
7.54
7.52
4.81
4.75
3.77
2.55
2.51
2.28
2.20
2.10
2.05
1.25
MeO2C
MeS
N
H
O
N
S
tBu
4d
1.00
1.11
1.11
3.40
2.34
1.21
4.44
9.40
9.0
8.5
8.0
7.5
7.0
6.5
6.0
5.5
5.0
4.5
4.0
3.5
3.0
2.5
2.0
1.5
1.0
0.5
0.0
-0.5
f1 (ppm)

msmc.ss031985.100.fid
msm / ss-031985 - 13c

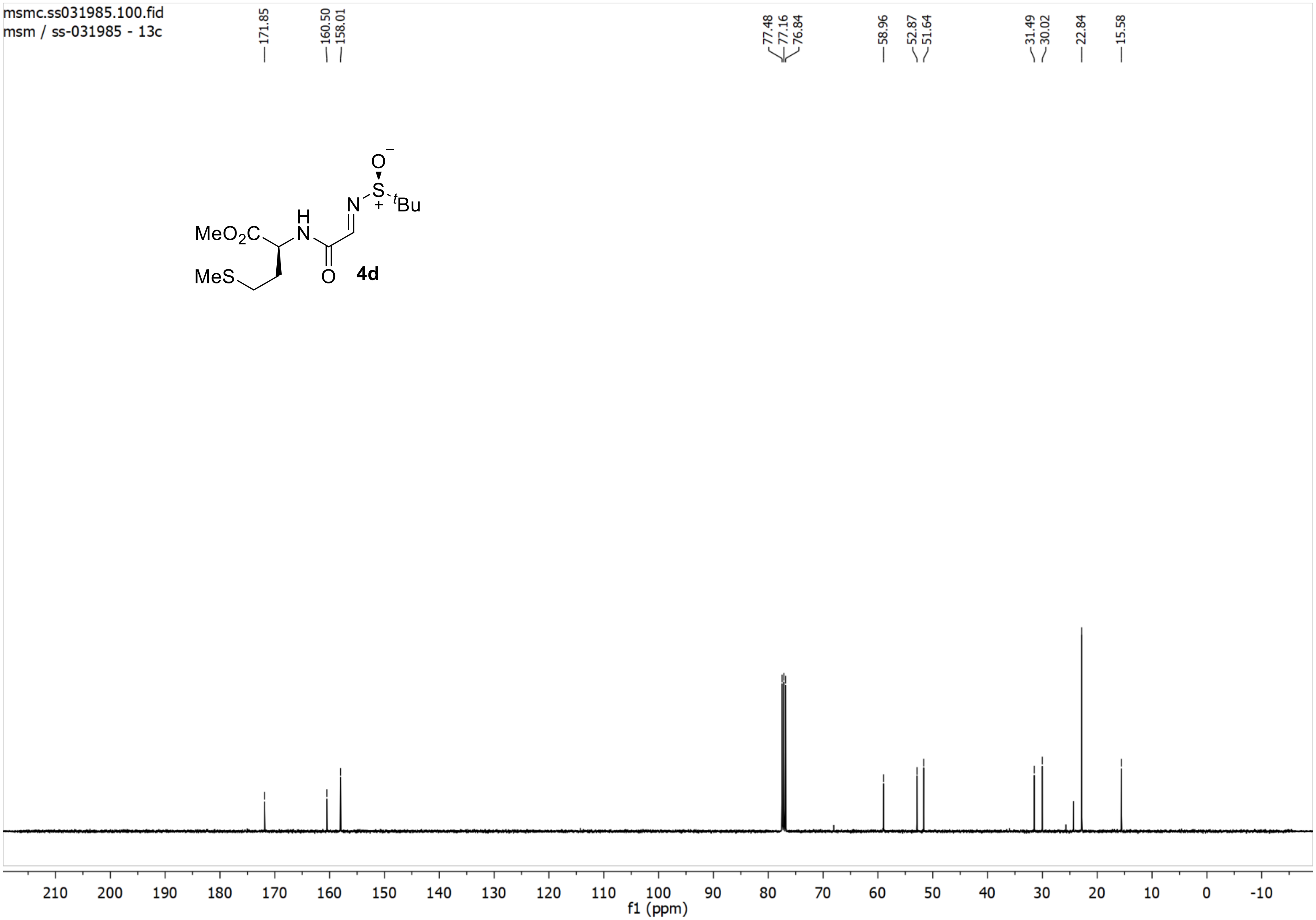

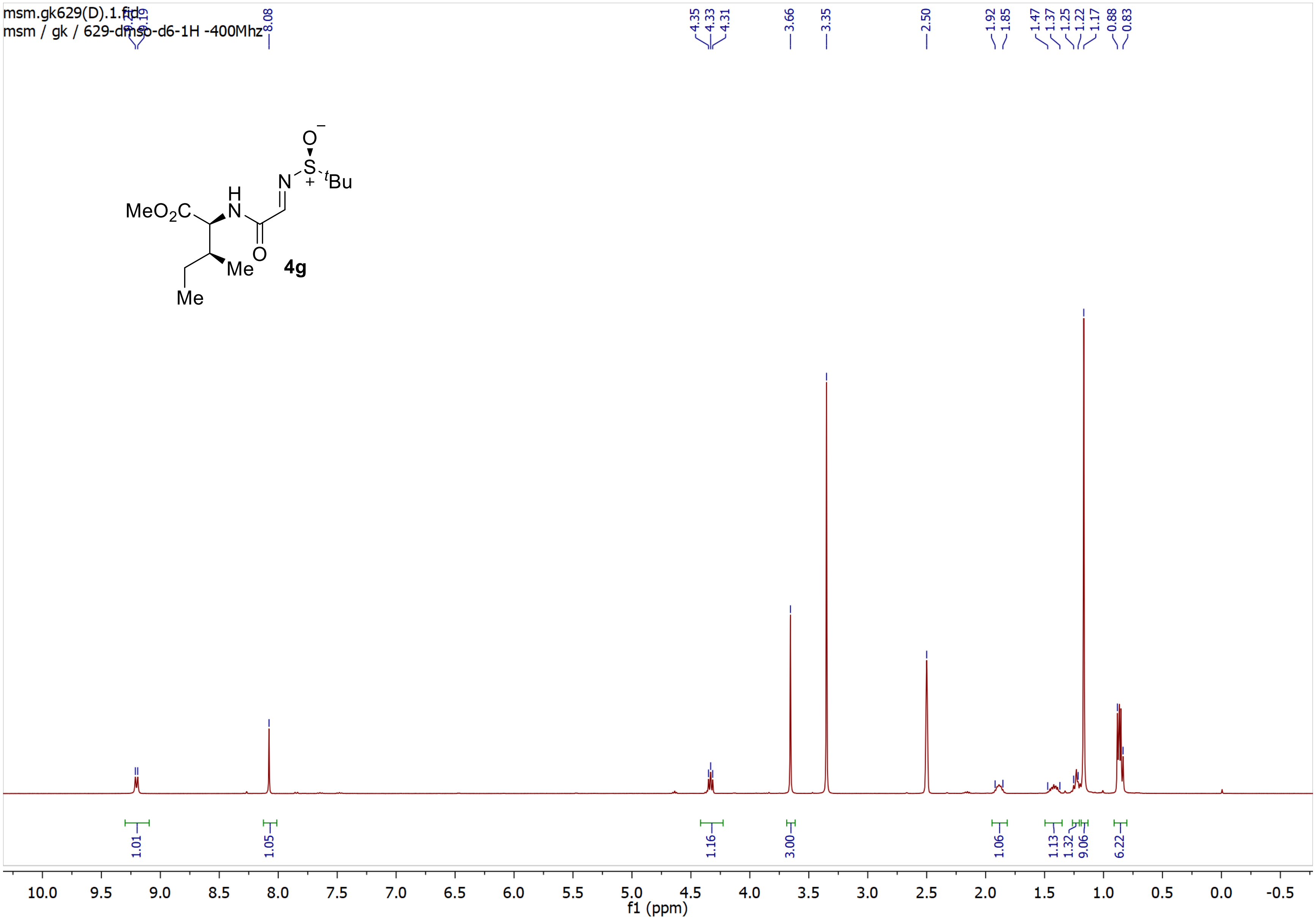

msm.gk629(D).1.fid
msm / gk / 629-dmso-d6-1H -400Mhz
9.21
9.19
8.08
4.35
4.33
4.31
3.66
3.35
2.50
1.92
1.85
1.47
1.37
1.25
1.22
1.17
0.88
0.83
MeO2C
H
N
O
N
S
+
tBu
O
Me
Me
4g
1.01
1.05
1.16
3.00
1.06
1.13
1.32
9.06
6.22
10.0 9.5 9.0 8.5 8.0 7.5 7.0 6.5 6.0 5.5 5.0 4.5 4.0 3.5 3.0 2.5 2.0 1.5 1.0 0.5 0.0 -0.5
f1 (ppm)

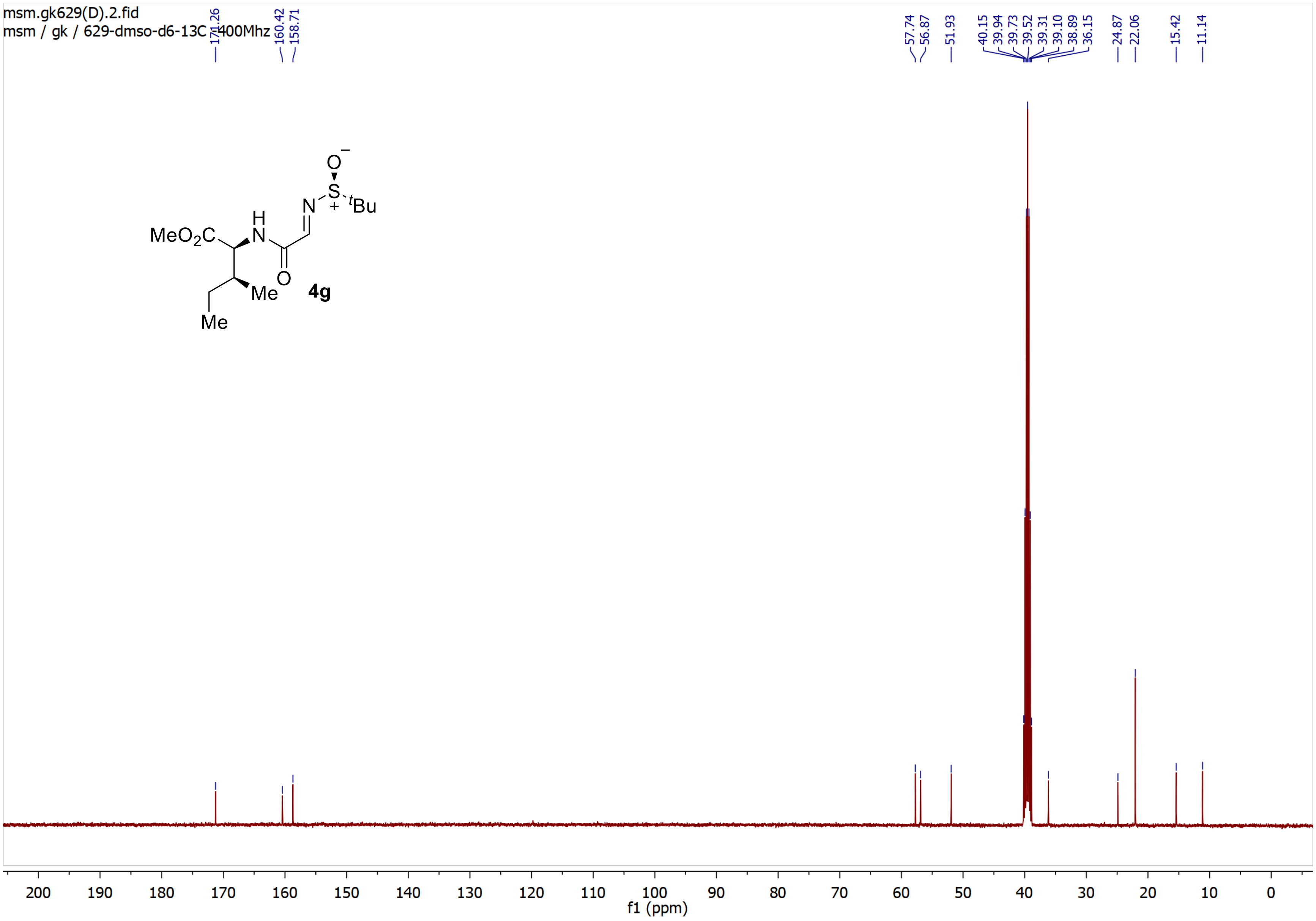

msm.gk629(D).2.fid
msm / gk / 629-dmso-d6-13C 400Mhz
171.26
160.42
158.71
57.74
56.87
51.93
40.15
39.94
39.73
39.52
39.31
39.10
38.89
36.15
24.87
22.06
15.42
11.14
MeO2C
H
N
N
S
tBu
O
O
Me
Me
4g
200 190 180 170 160 150 140 130 120 110 100 90 80 70 60 50 40 30 20 10 0
f1 (ppm)

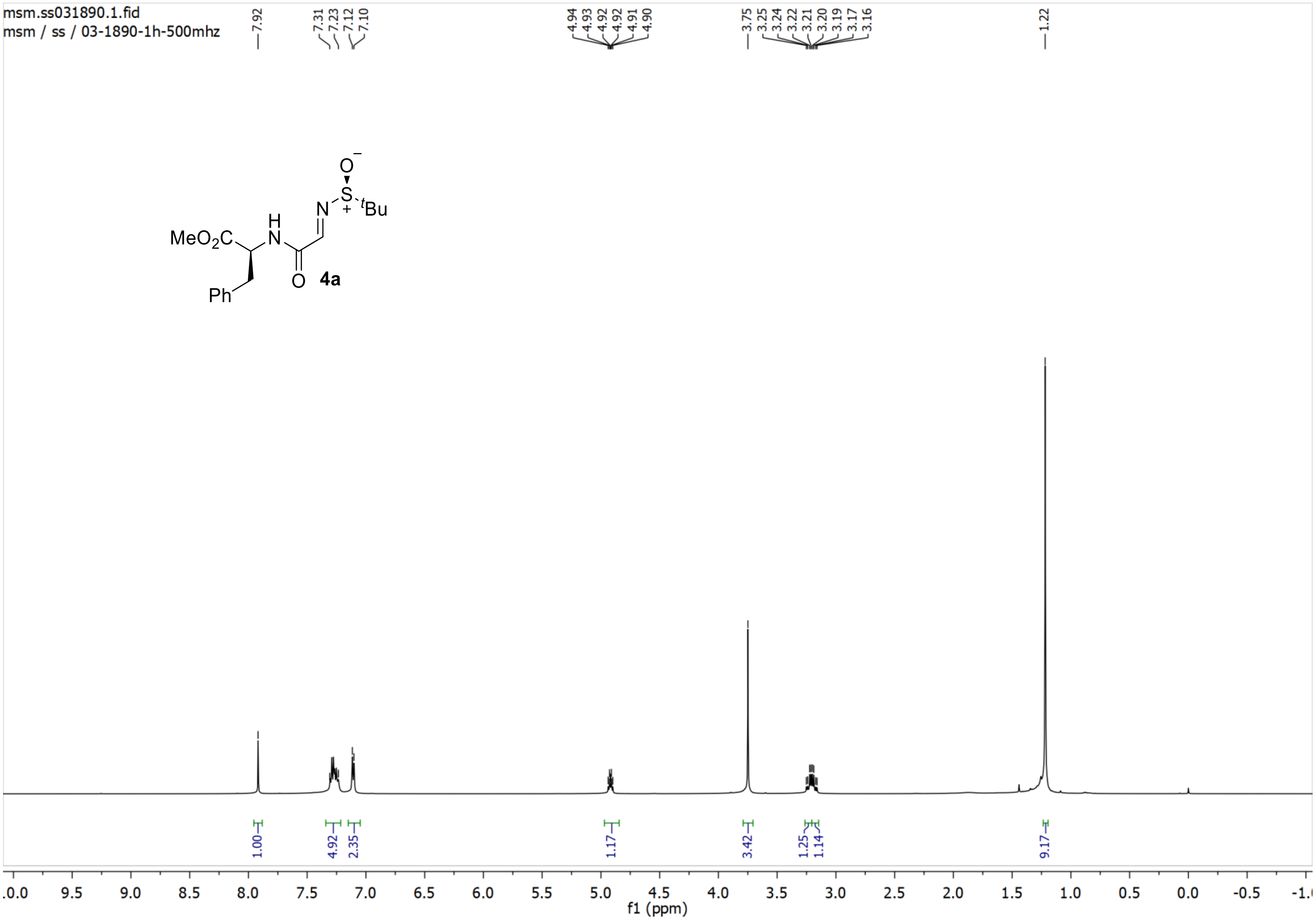

msm.ss031890.1.fid
msm / ss / 03-1890-1h-500mhz
7.92
7.31
7.23
7.12
7.10
4.94
4.93
4.92
4.92
4.91
4.90
3.75
3.25
3.24
3.22
3.21
3.20
3.19
3.17
3.16
1.22
MeO2C
H
N
O
N
S
+
O
−
tBu
Ph
4a
1.00
4.92
2.35
1.17
3.42
1.25
1.14
9.17
f1 (ppm)

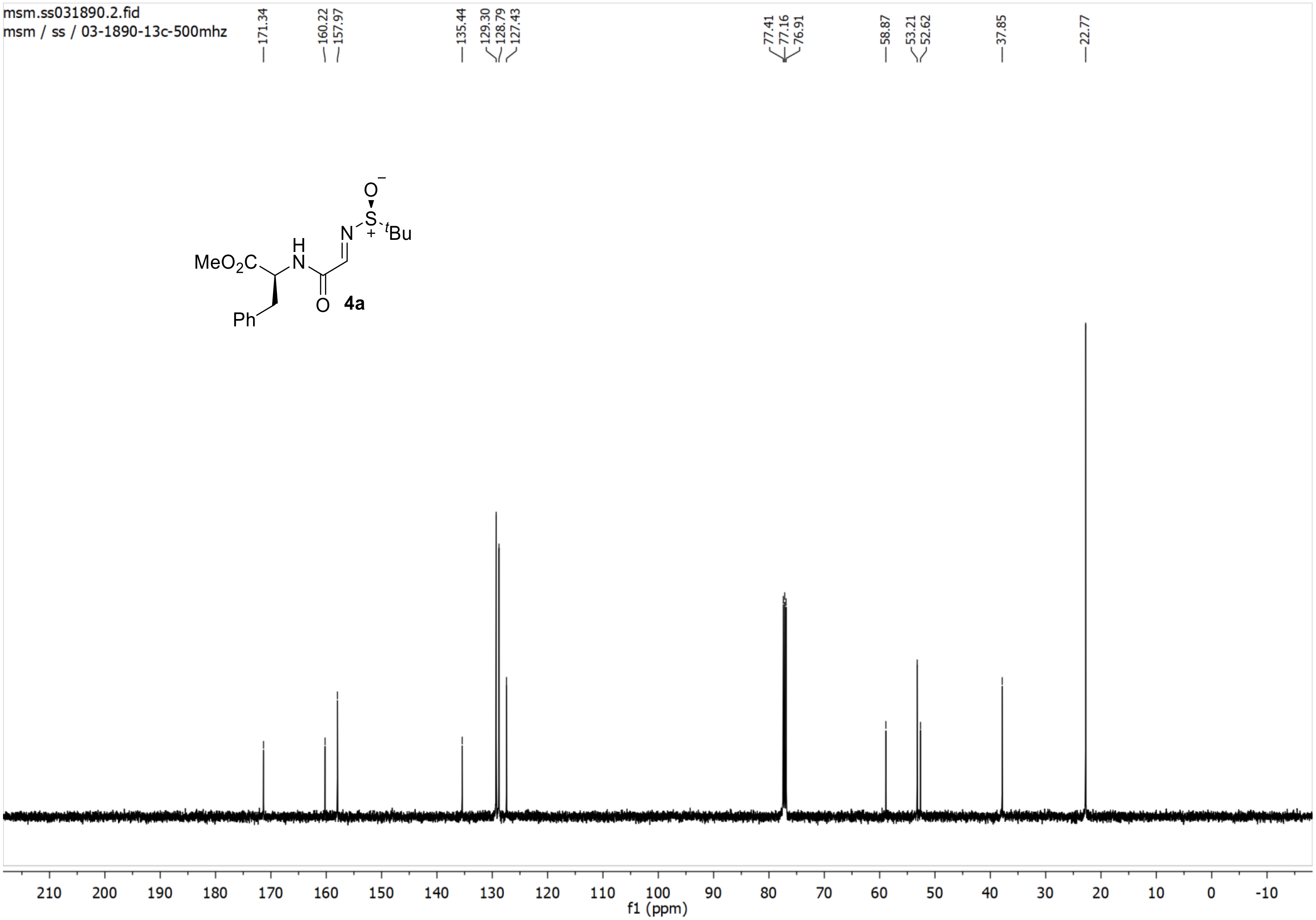
msm.ss031890.2.fid
msm / ss / 03-1890-13c-500mhz
171.34
160.22
157.97
135.44
129.30
128.79
127.43
77.41
77.16
76.91
58.87
53.21
52.62
37.85
22.77
MeO2C
H
N
N
O
S
+
O
tBu
Ph
4a
210 200 190 180 170 160 150 140 130 120 110 100 90 80 70 60 50 40 30 20 10 0 -10
f1 (ppm)

msm.ss03-2428r3.1.fid
msm / ss / 03-2428r3-1H -400Mhz

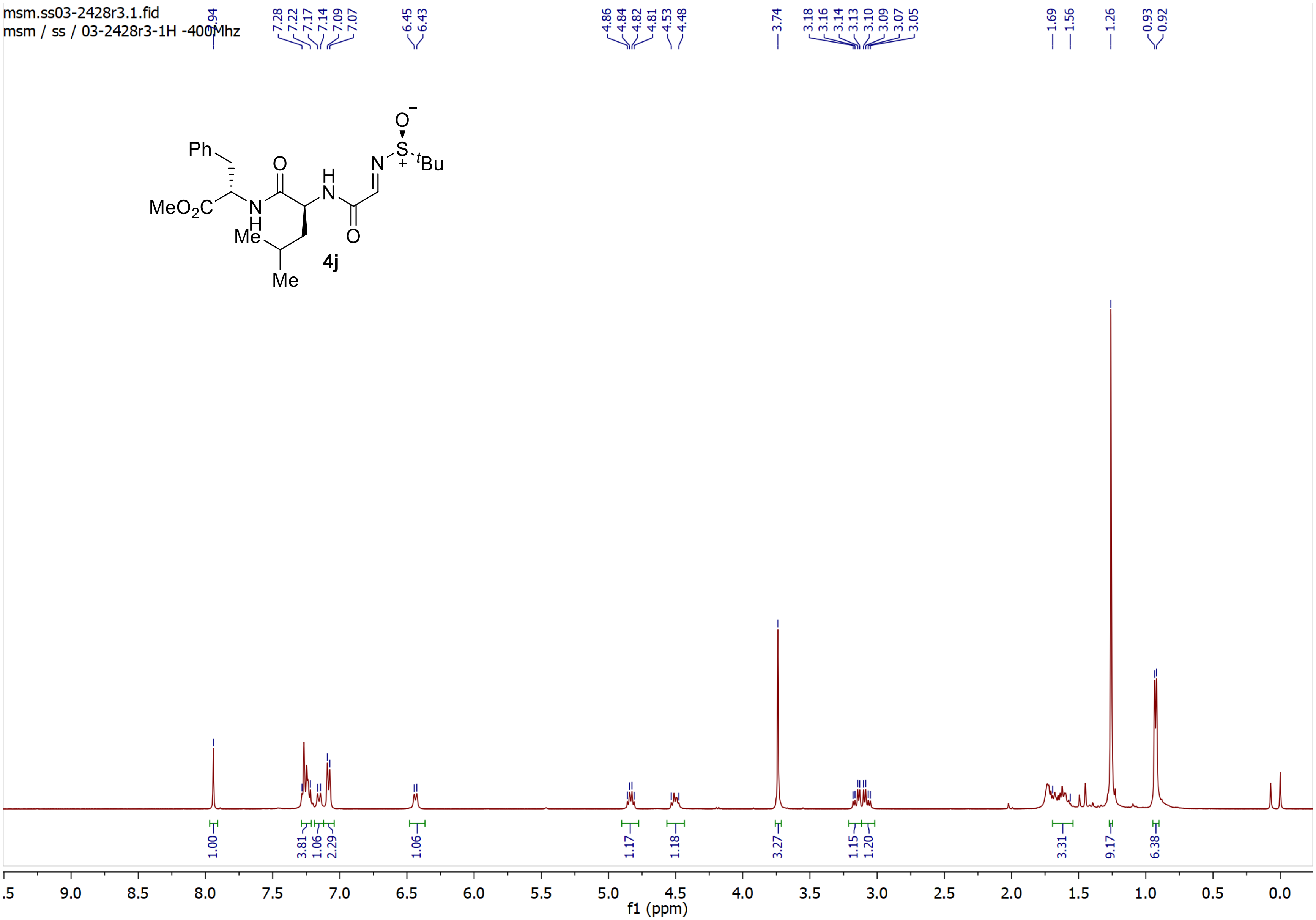

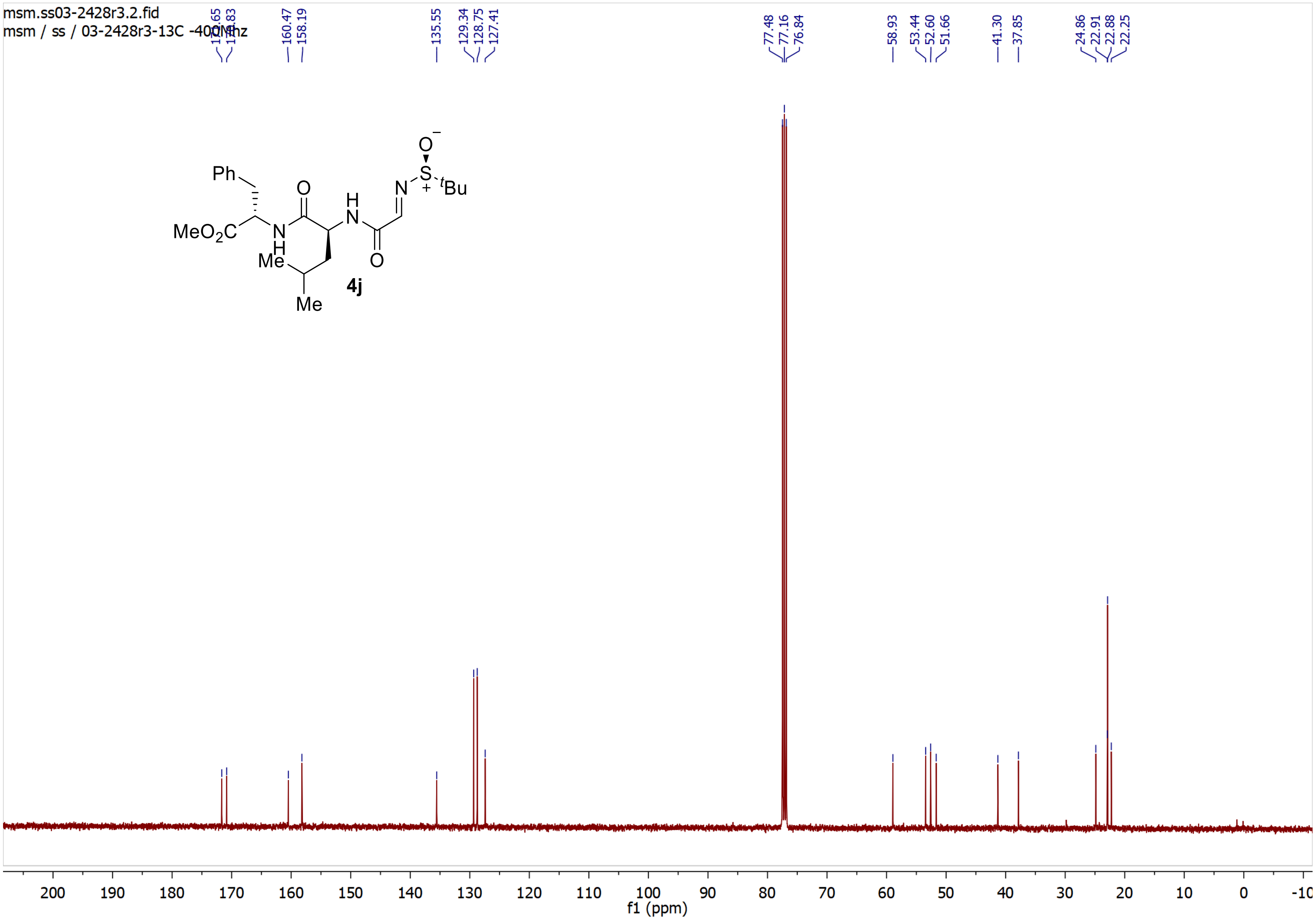
msm.ss03-2428r3.2.fid
msm / ss / 03-2428r3-13C -400Mhz
160.47
158.19
135.55
129.34
128.75
127.41
77.48
77.16
76.84
58.93
53.44
52.60
51.66
41.30
37.85
24.86
22.91
22.88
22.25
Ph
MeO2C
N
H
O
H
N
N
S
O
tBu
O
Me
Me
4j
200 190 180 170 160 150 140 130 120 110 100 90 80 70 60 50 40 30 20 10 0 -10
f1 (ppm)

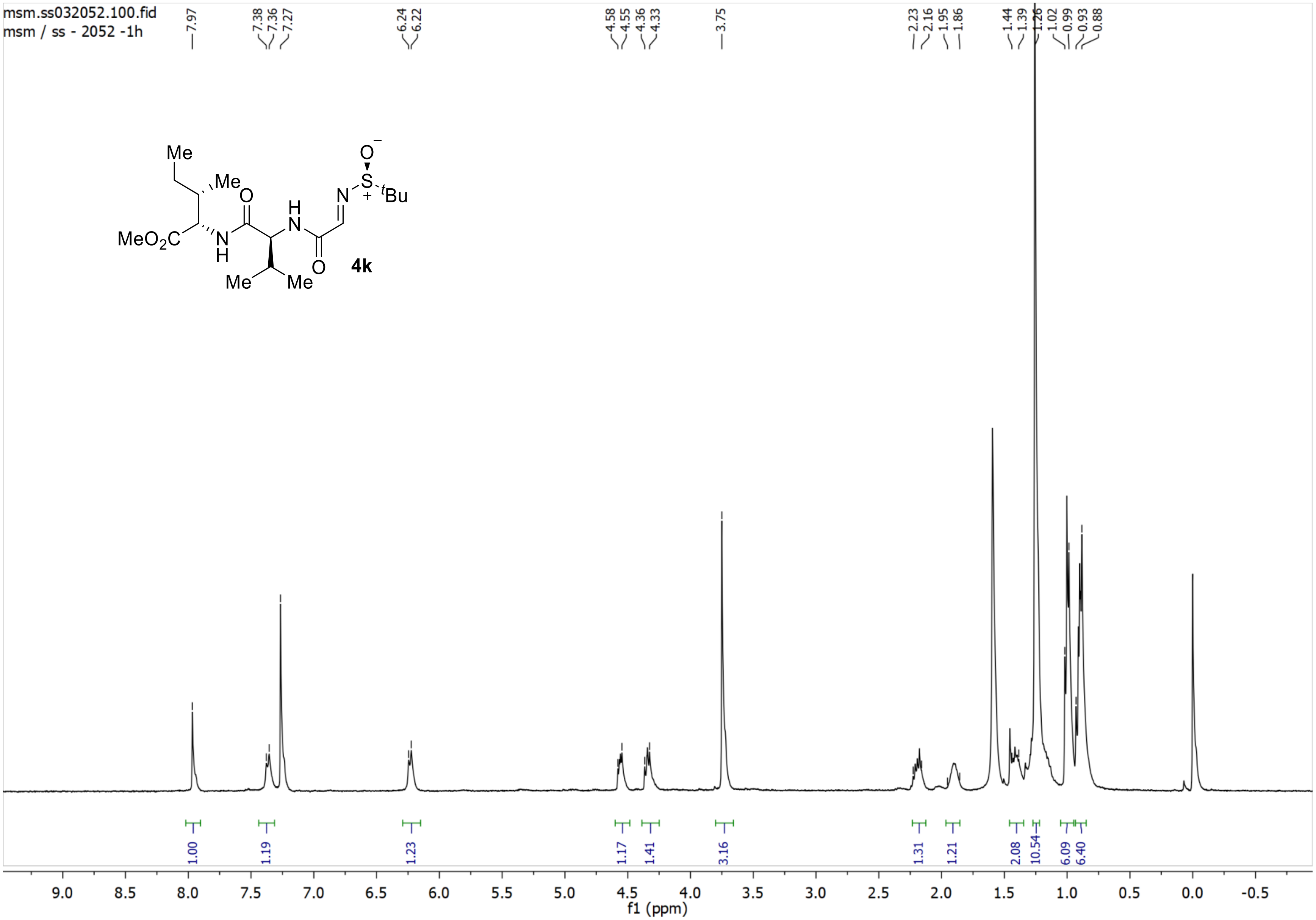
msm.ss032052.100.fid
msm / ss - 2052 -1h
7.97
7.38
7.36
7.27
6.24
6.22
4.58
4.55
4.36
4.33
3.75
2.23
2.16
1.95
1.86
1.44
1.39
1.26
1.02
0.99
0.93
0.88
4k
1.00
1.19
1.23
1.17
1.41
3.16
1.31
1.21
2.08
10.54
6.09
6.40
9.0 8.5 8.0 7.5 7.0 6.5 6.0 5.5 5.0 4.5 4.0 3.5 3.0 2.5 2.0 1.5 1.0 0.5 0.0 -0.5
f1 (ppm)

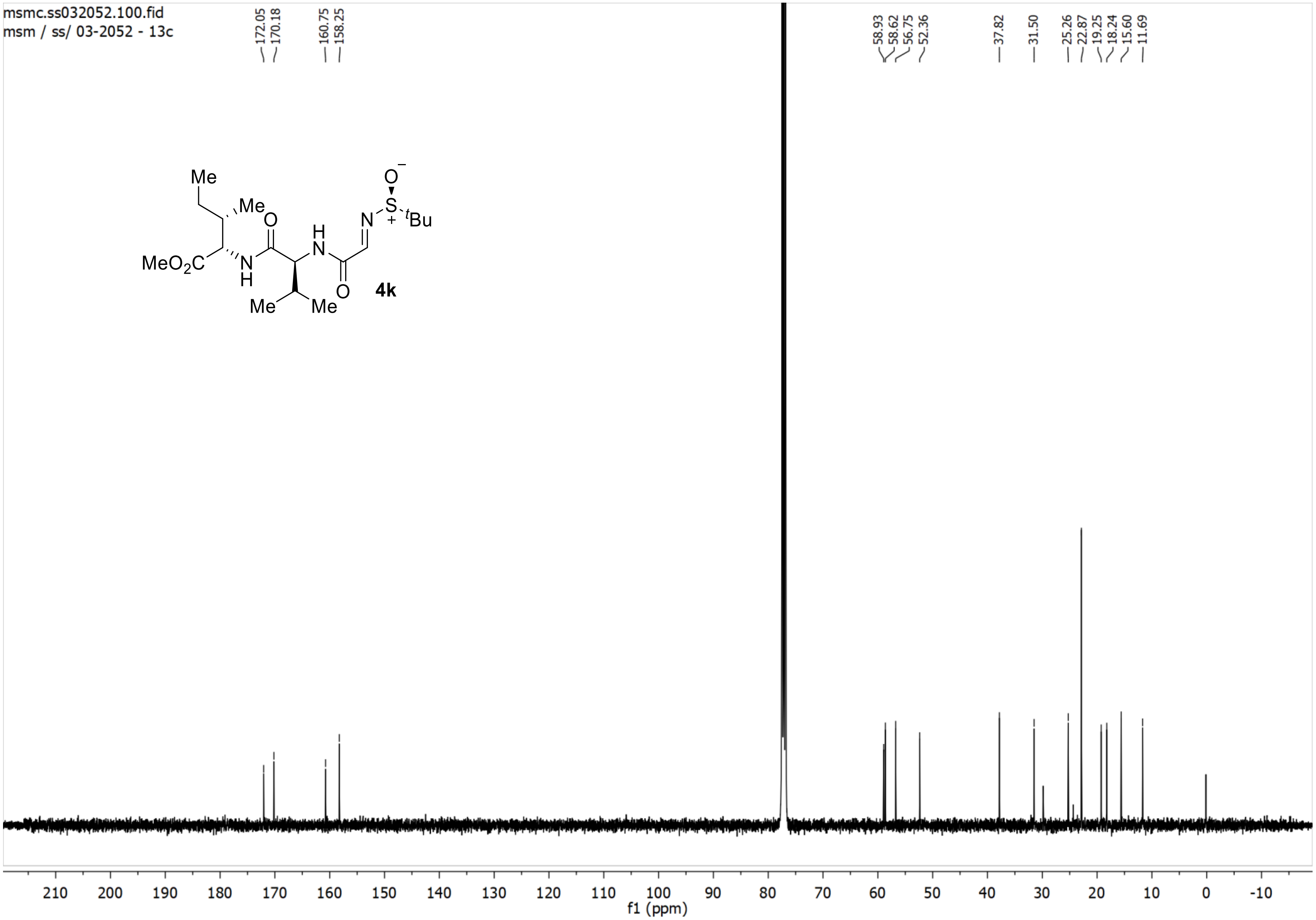
msmc.ss032052.100.fid
msm / ss/ 03-2052 - 13c
172.05
170.18
160.75
158.25
58.93
58.62
56.75
52.36
37.82
31.50
25.26
22.87
19.25
18.24
15.60
11.69
4k
210 200 190 180 170 160 150 140 130 120 110 100 90 80 70 60 50 40 30 20 10 0 -10
f1 (ppm)

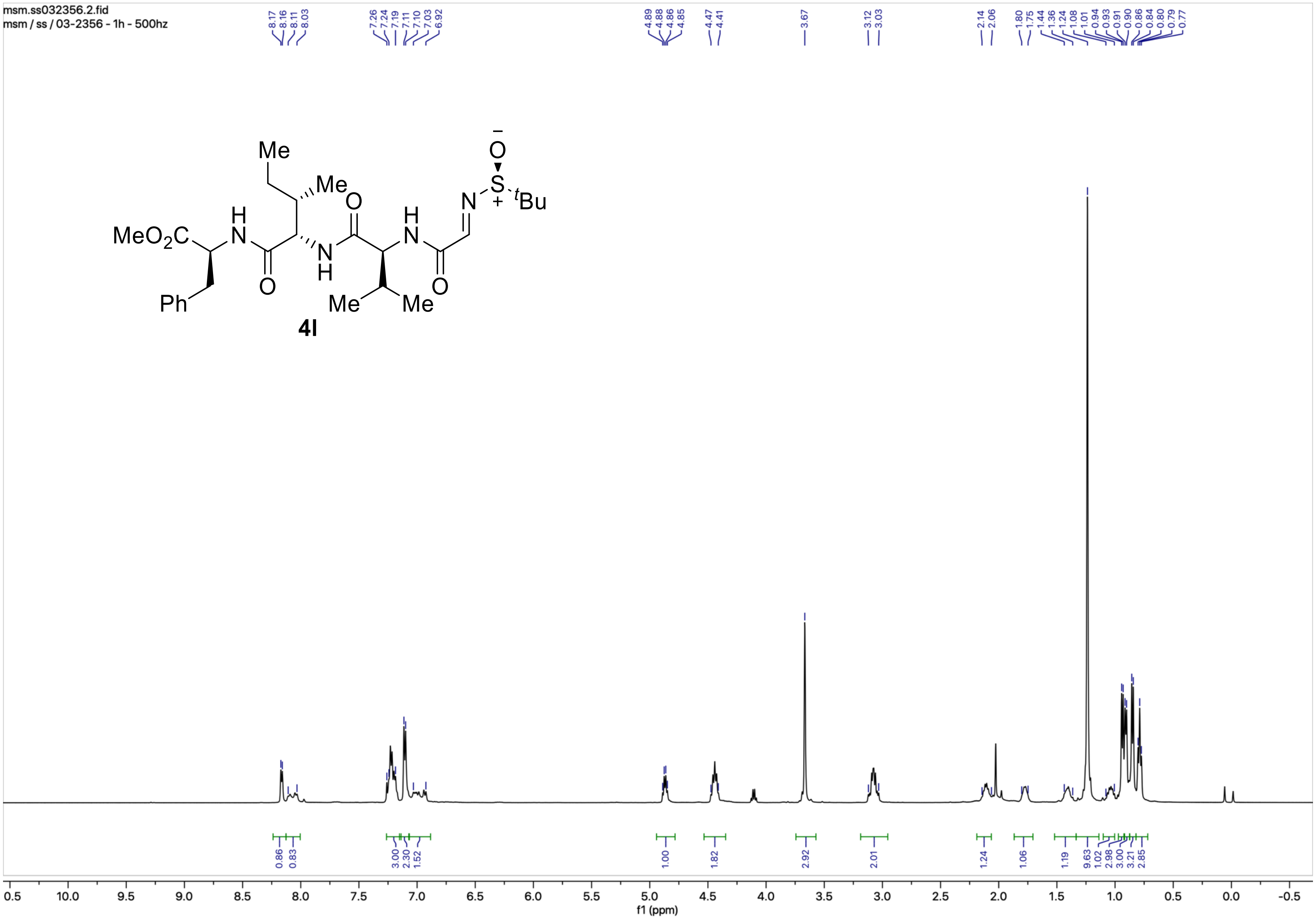
msm.ss032356.2.fid
msm / ss / 03-2356 - 1h - 500hz
8.17 8.16 8.11 8.03
7.26 7.24 7.19 7.11 7.10 7.03 6.92
4.89 4.88 4.86 4.85
4.47 4.41
3.67
3.12 3.03
2.14 2.06
1.80 1.75 1.44 1.36 1.24 1.08 1.01 0.94 0.93 0.91 0.90 0.86 0.84 0.80 0.79 0.77
Me
Me
O
N
S
tBu
MeO2C
H
N
N
H
O
Ph
Me
Me
4l
0.86 0.83
3.00 2.30 1.52
1.00
1.82
2.92
2.01
1.24
1.06
1.19 9.63 1.02 2.98 3.00 3.21 2.85
f1 (ppm)

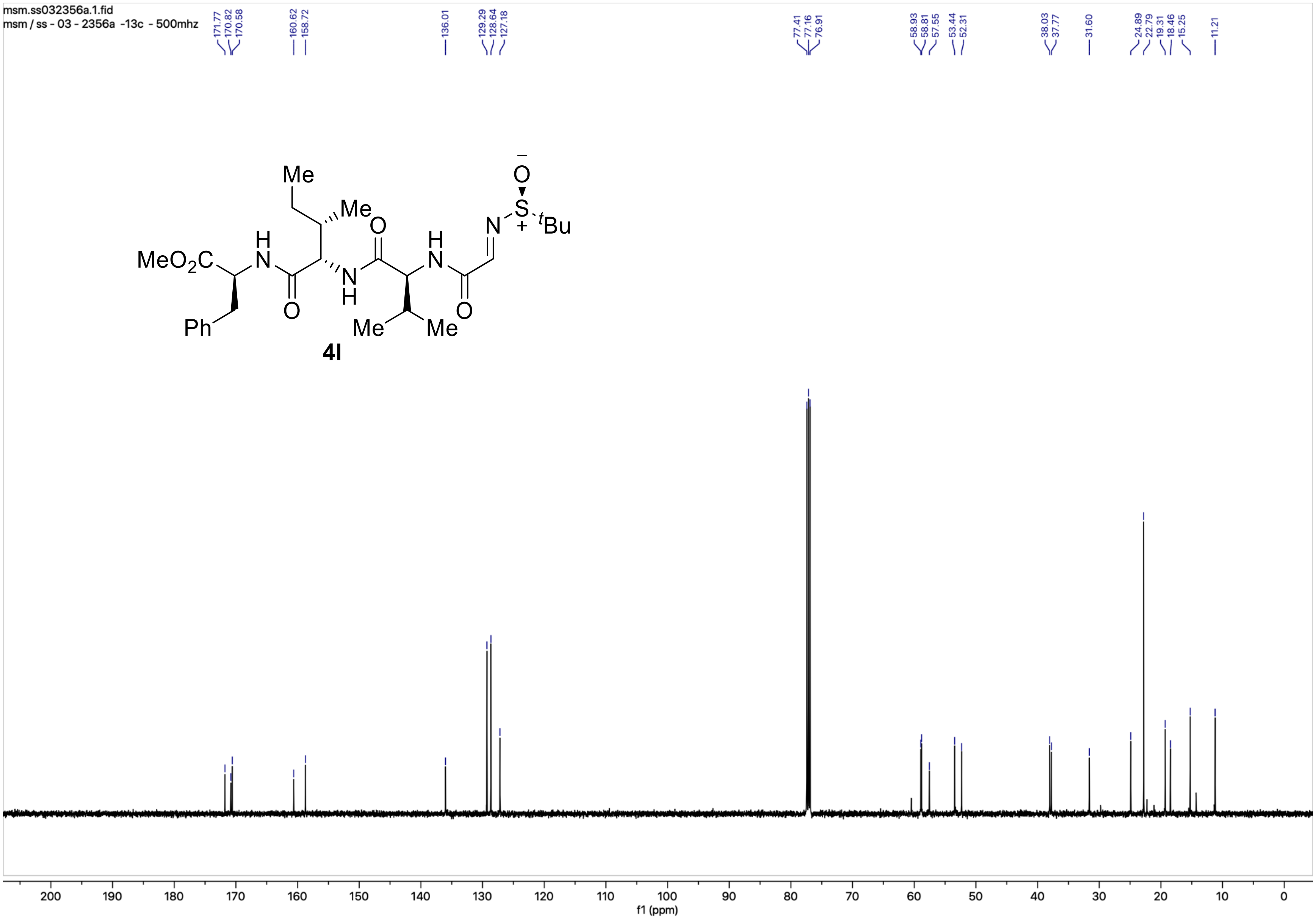
msm.ss032356a.1.fid
msm / ss - 03 - 2356a -13c - 500mhz
171.77
170.82
170.58
160.62
158.72
136.01
129.29
128.64
127.18
77.41
77.16
76.91
58.93
58.81
57.55
53.44
52.31
38.03
37.77
31.60
24.89
22.79
19.31
18.46
15.25
11.21
4l
f1 (ppm)

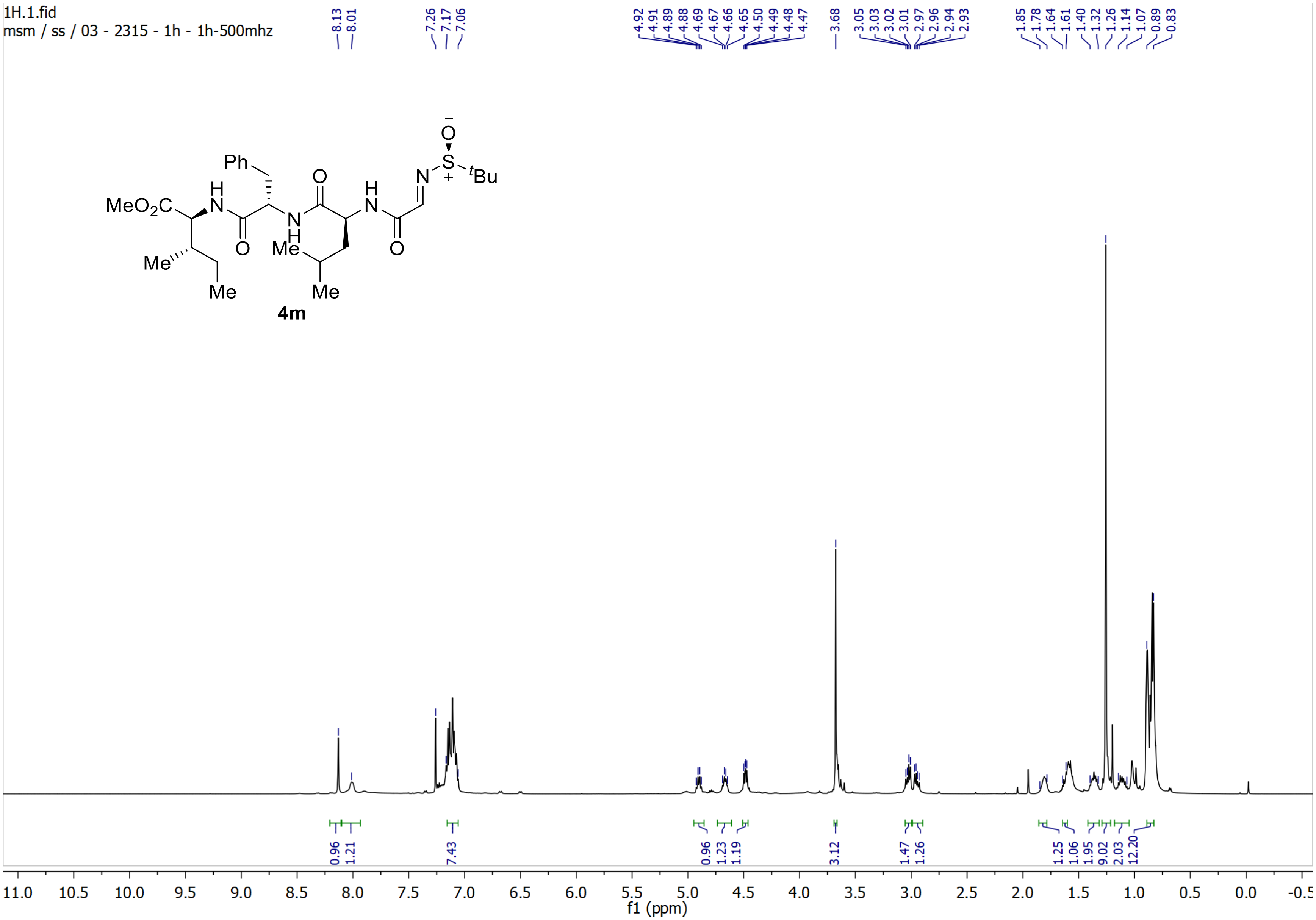
1H.1.fid
msm / ss / 03 - 2315 - 1h - 1h-500mhz
8.13
8.01
7.26
7.17
7.06
4.92
4.91
4.89
4.88
4.69
4.67
4.66
4.65
4.50
4.49
4.48
4.47
3.68
3.05
3.03
3.02
3.01
2.97
2.96
2.94
2.93
1.85
1.78
1.64
1.61
1.40
1.32
1.26
1.14
1.07
0.89
0.83
4m
0.96
1.21
7.43
0.96
1.23
1.19
3.12
1.47
1.26
1.25
1.06
1.95
9.02
2.03
12.20
f1 (ppm)

msm.ss032315.2.fid
msm / ss / 03 - 2315 - 13c -500mhz

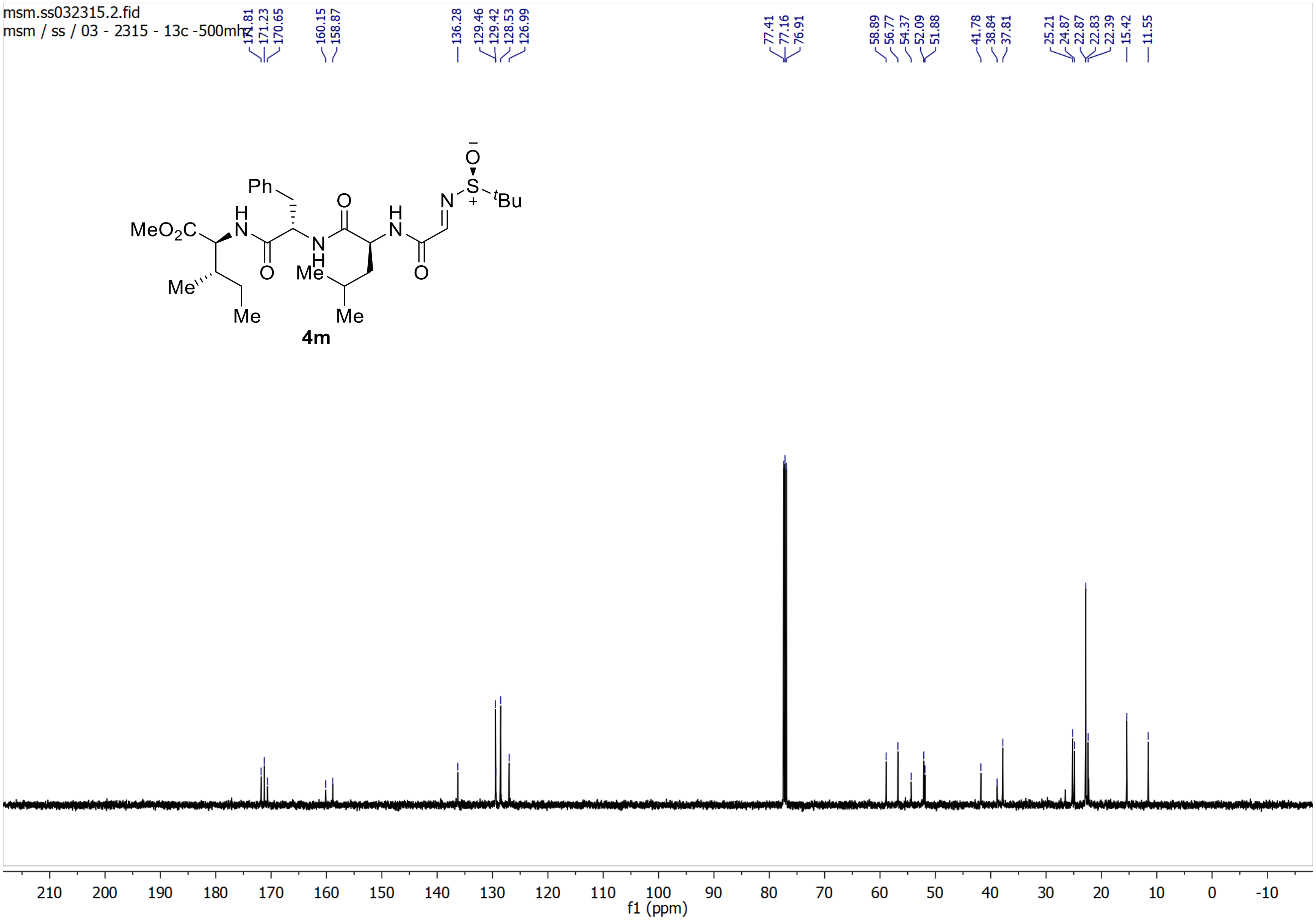

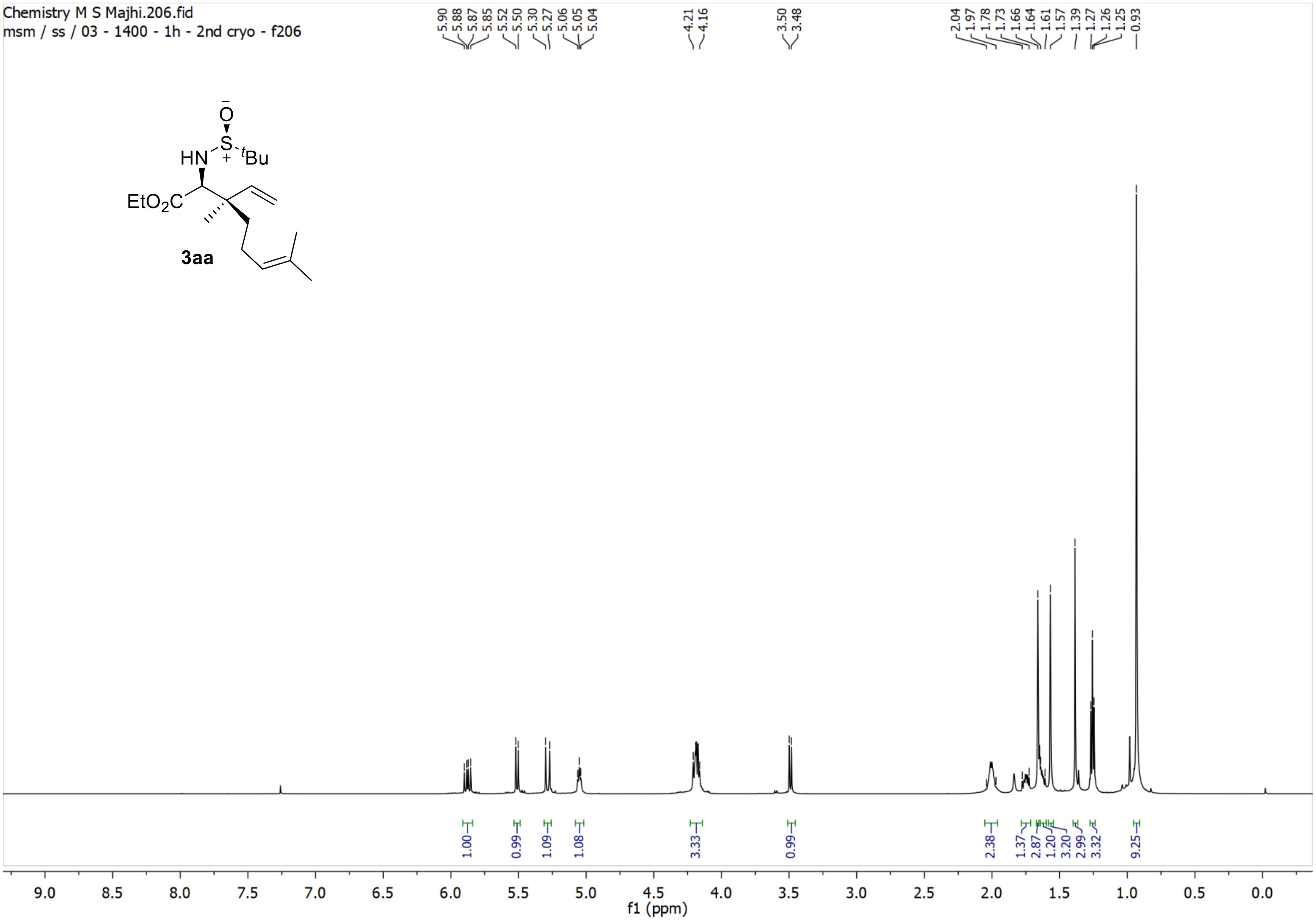
Chemistry M S Majhi.206.fid
msm / ss / 03 - 1400 - 1h - 2nd cryo - f206
3aa
f1 (ppm)

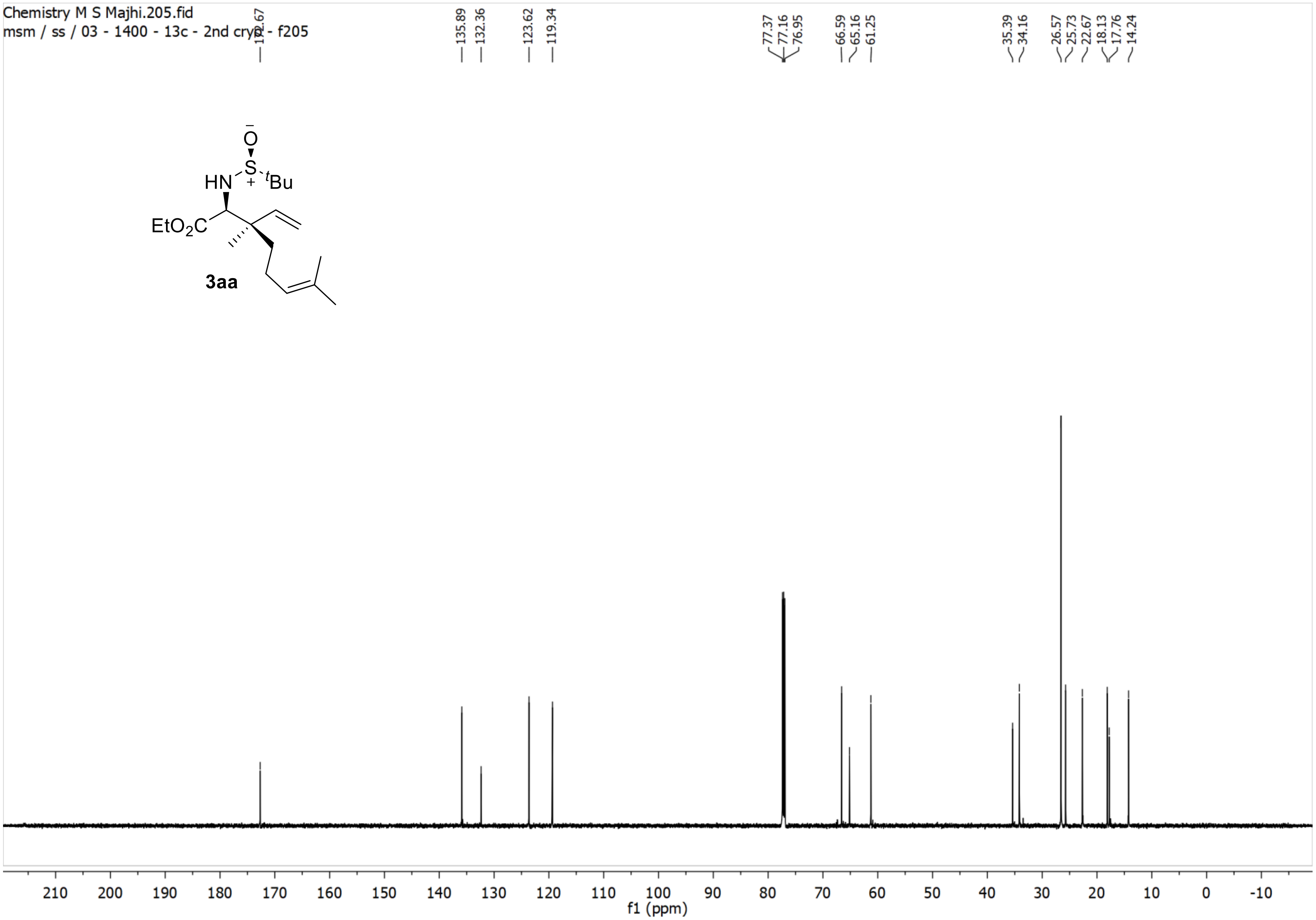
Chemistry M S Majhi.205.fid
msm / ss / 03 - 1400 - 13c - 2nd cryo - f205
172.67
135.89
132.36
123.62
119.34
77.37
77.16
76.95
66.59
65.16
61.25
35.39
34.16
26.57
25.73
22.67
18.13
17.76
14.24
HN
S
tBu
O
EtO2C
3aa
210
200
190
180
170
160
150
140
130
120
110
100
90
80
70
60
50
40
30
20
10
0
-10
f1 (ppm)

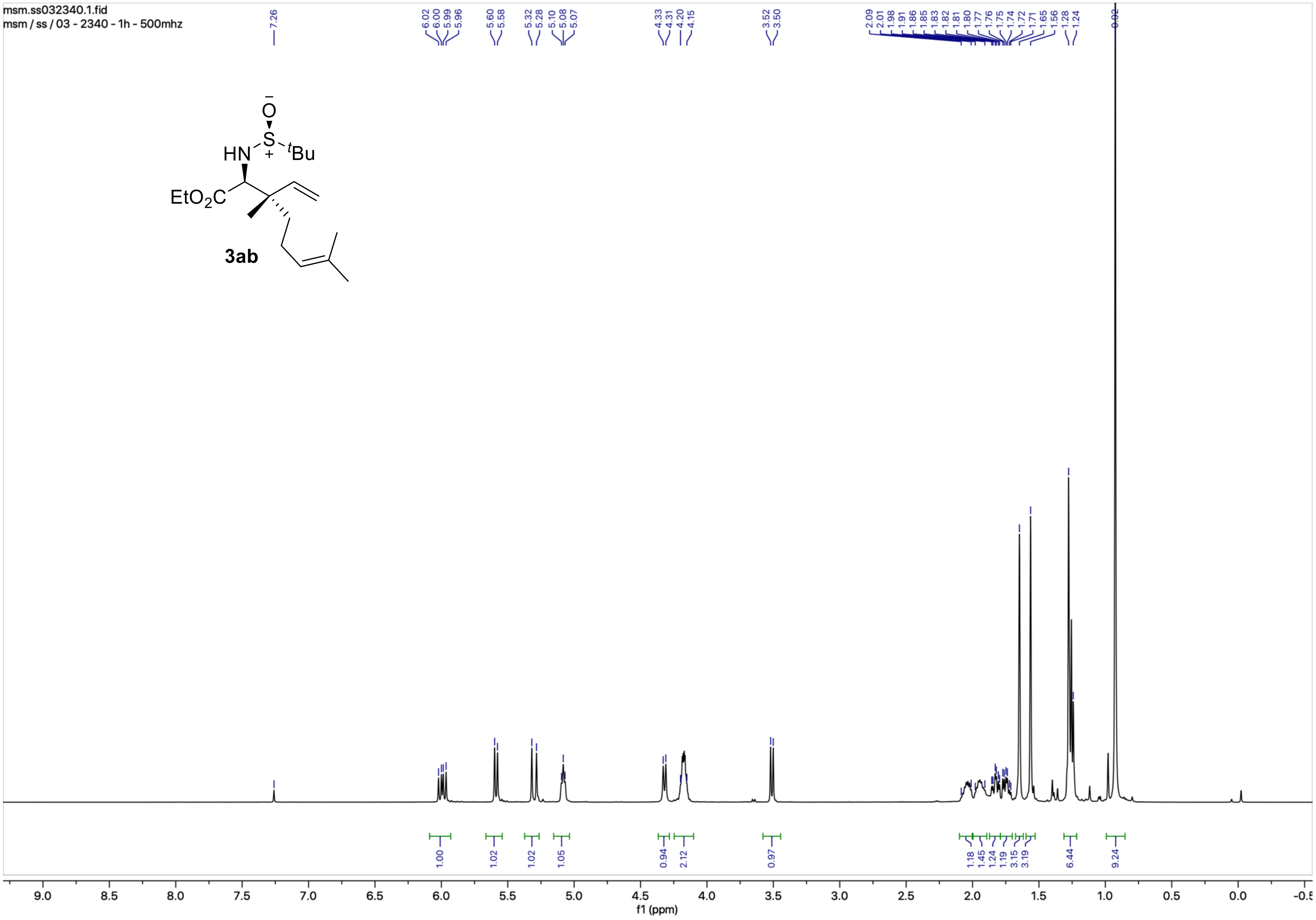
msm.ss032340.1.fid
msm / ss / 03 - 2340 - 1h - 500mhz
3ab
f1 (ppm)

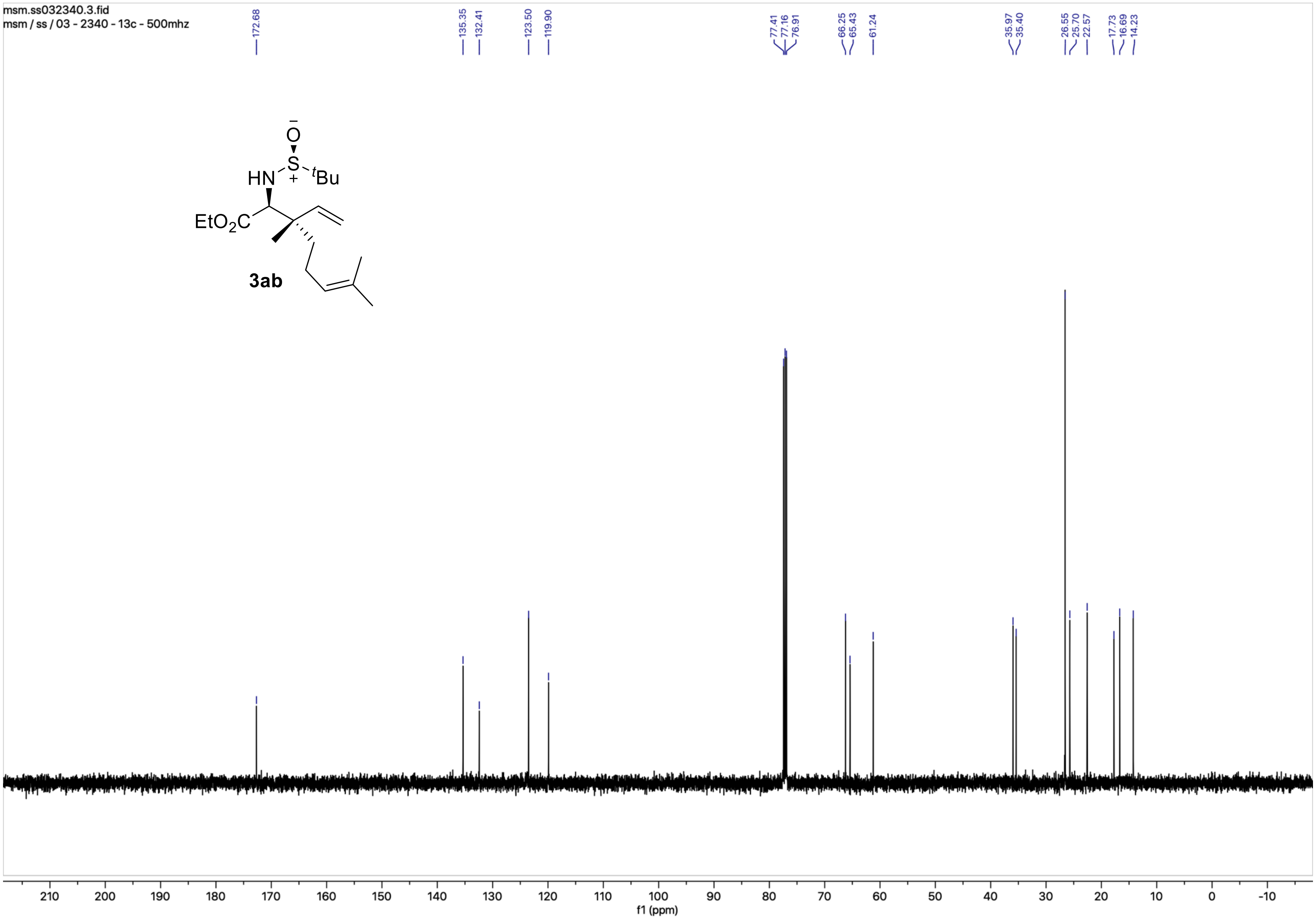
msm.ss032340.3.fid
msm / ss / 03 - 2340 - 13c - 500mhz
172.68
135.35
132.41
123.50
119.90
77.41
77.16
76.91
66.25
65.43
61.24
35.97
35.40
26.55
25.70
22.57
17.73
16.69
14.23
HN
S
tBu
EtO2C
3ab
210
200
190
180
170
160
150
140
130
120
110
100
90
80
70
60
50
40
30
20
10
0
-10
f1 (ppm)

Chemistry M S Majhi.240.fid
msm / ss / 03-1409 - 1h - 2nd cryo - f240

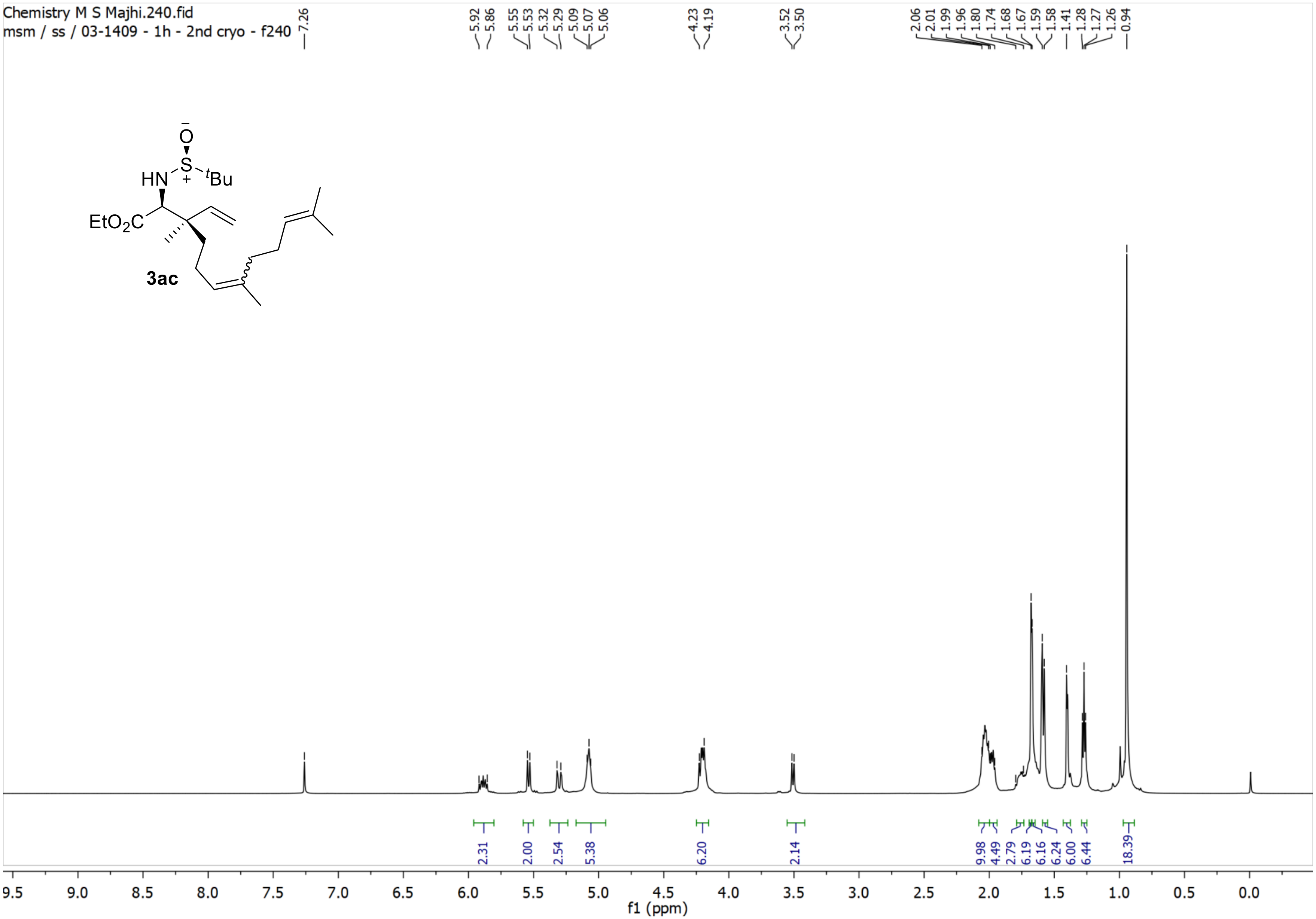

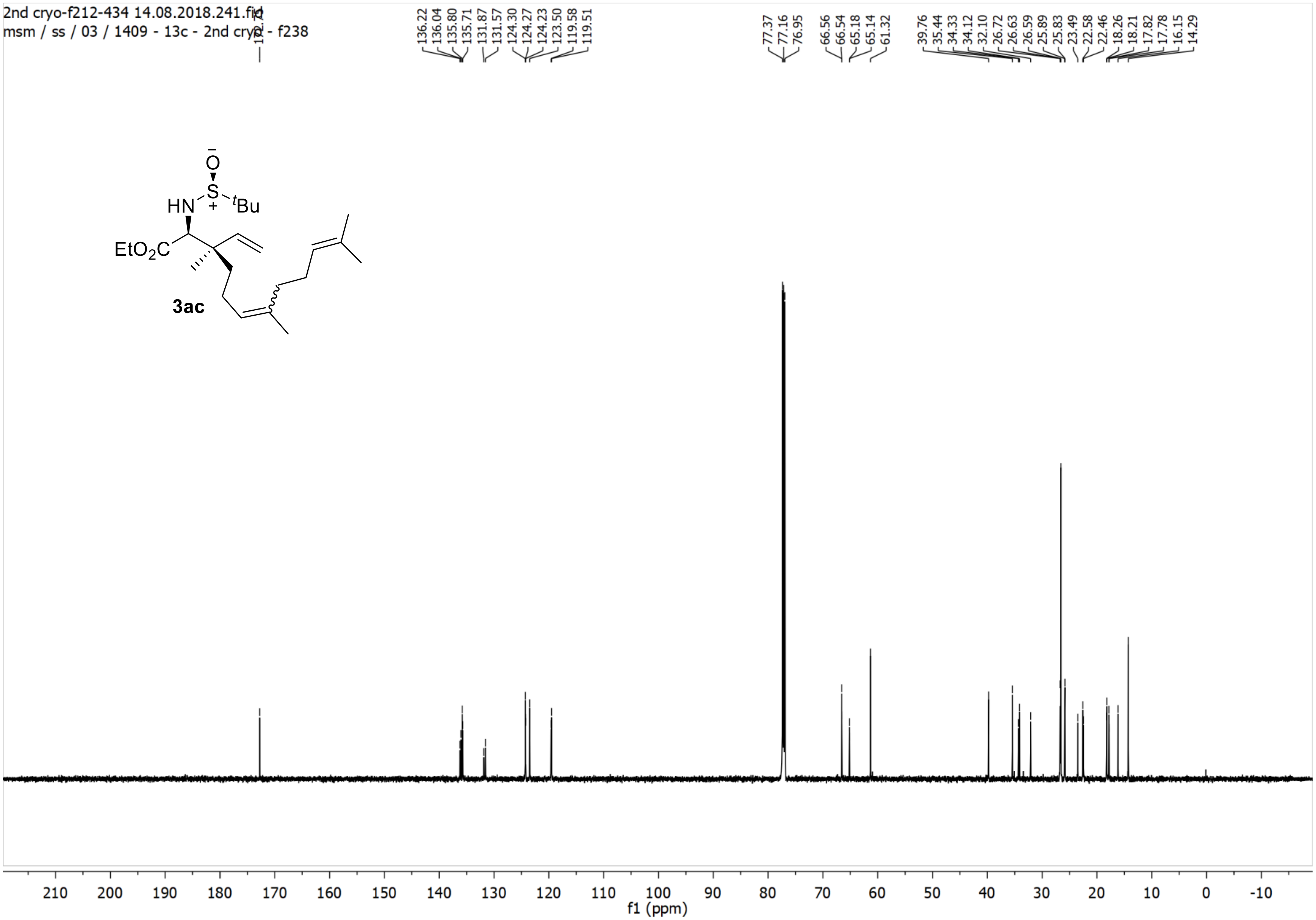

2nd cryo-f212-434 14.08.2018.241.fid
msm / ss / 03 / 1409 - 13c - 2nd cryo - f238
136.22
136.04
135.80
135.71
131.87
131.57
124.30
124.27
124.23
123.50
119.58
119.51
77.37
77.16
76.95
66.56
66.54
65.18
65.14
61.32
39.76
35.44
34.33
34.12
32.10
26.72
26.63
26.59
25.89
25.83
23.49
22.58
22.46
18.26
18.21
17.82
17.78
16.15
14.29
EtO2C
HN
S
tBu
O
3ac
210
200
190
180
170
160
150
140
130
120
110
100
90
80
70
60
50
40
30
20
10
0
-10
f1 (ppm)

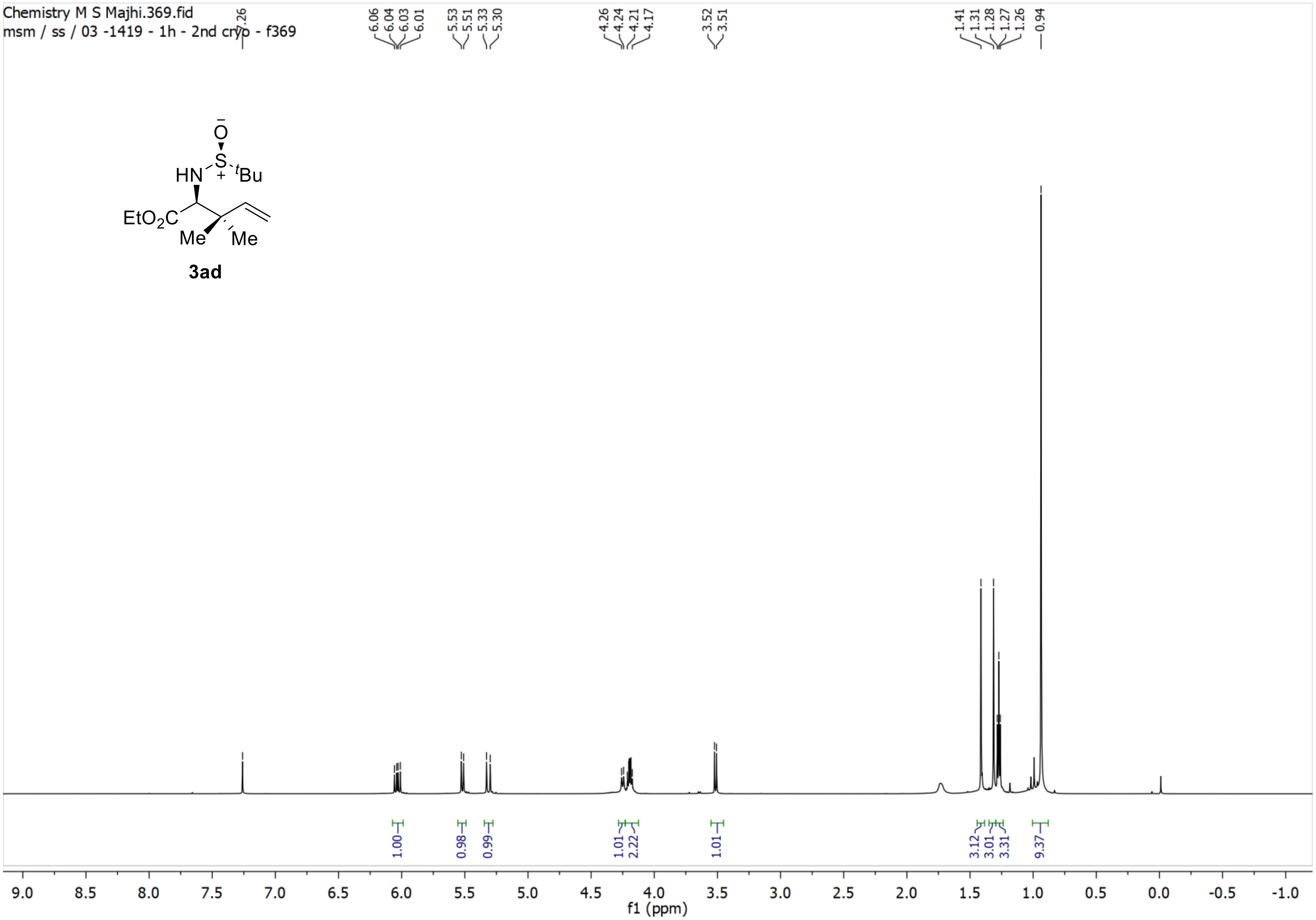
Chemistry M S Majhi.369.fid
msm / ss / 03 -1419 - 1h - 2nd cryo - f369
7.26
6.06
6.04
6.03
6.01
5.53
5.51
5.33
5.30
4.26
4.24
4.21
4.17
3.52
3.51
1.41
1.31
1.28
1.27
1.26
0.94
HN
^{t}Bu
EtO_2C
Me
Me
3ad
1.00
0.98
0.99
1.01
2.22
1.01
3.12
3.01
3.31
9.37
9.0 8.5 8.0 7.5 7.0 6.5 6.0 5.5 5.0 4.5 4.0 3.5 3.0 2.5 2.0 1.5 1.0 0.5 0.0 -0.5 -1.0
f1 (ppm)

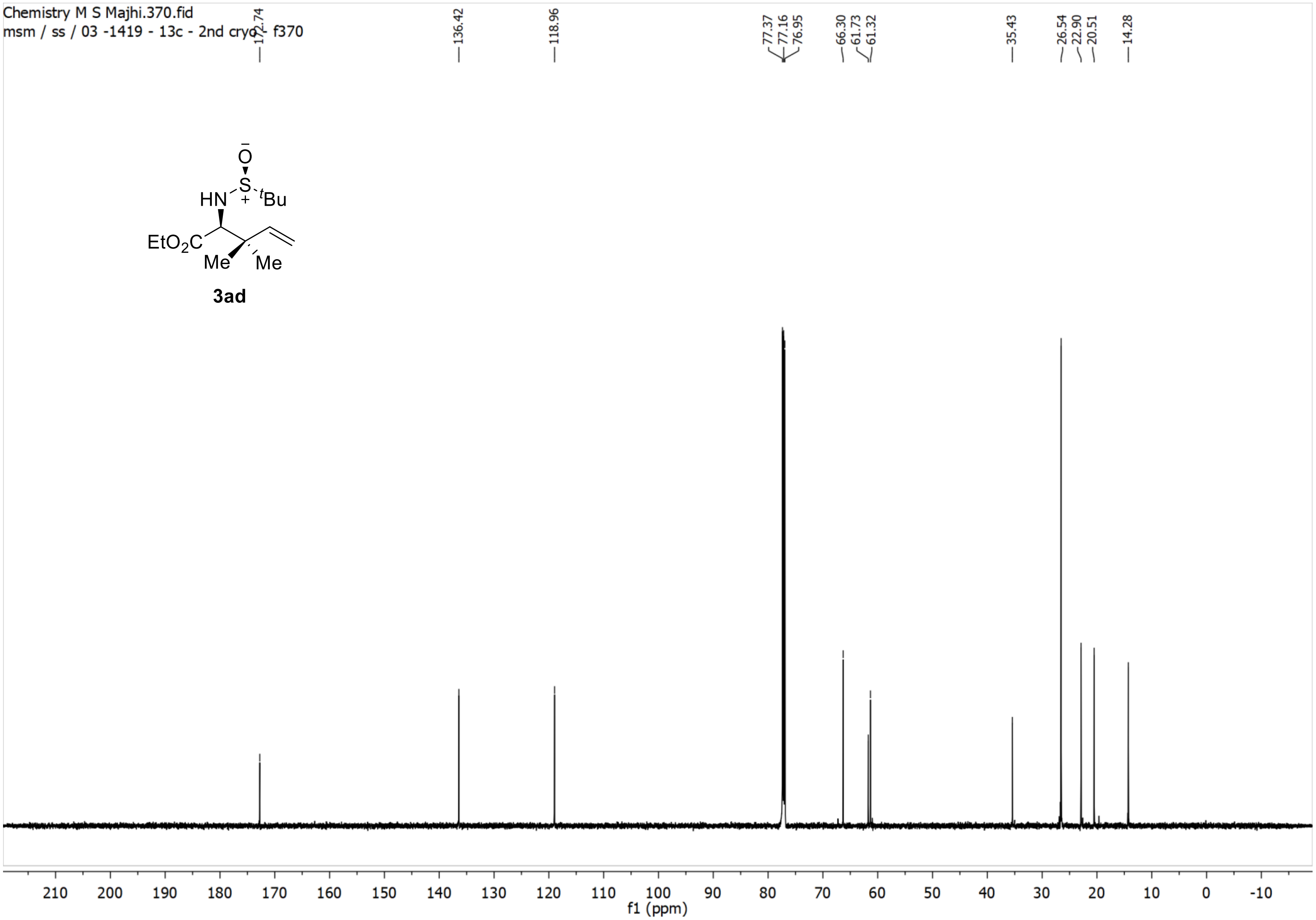
Chemistry M S Majhi.370.fid
msm / ss / 03 -1419 - 13c - 2nd cryo - f370
172.74
136.42
118.96
77.37
77.16
76.95
66.30
61.73
61.32
35.43
26.54
22.90
20.51
14.28
HN
S
tBu
O
EtO2C
Me
Me
3ad
f1 (ppm)

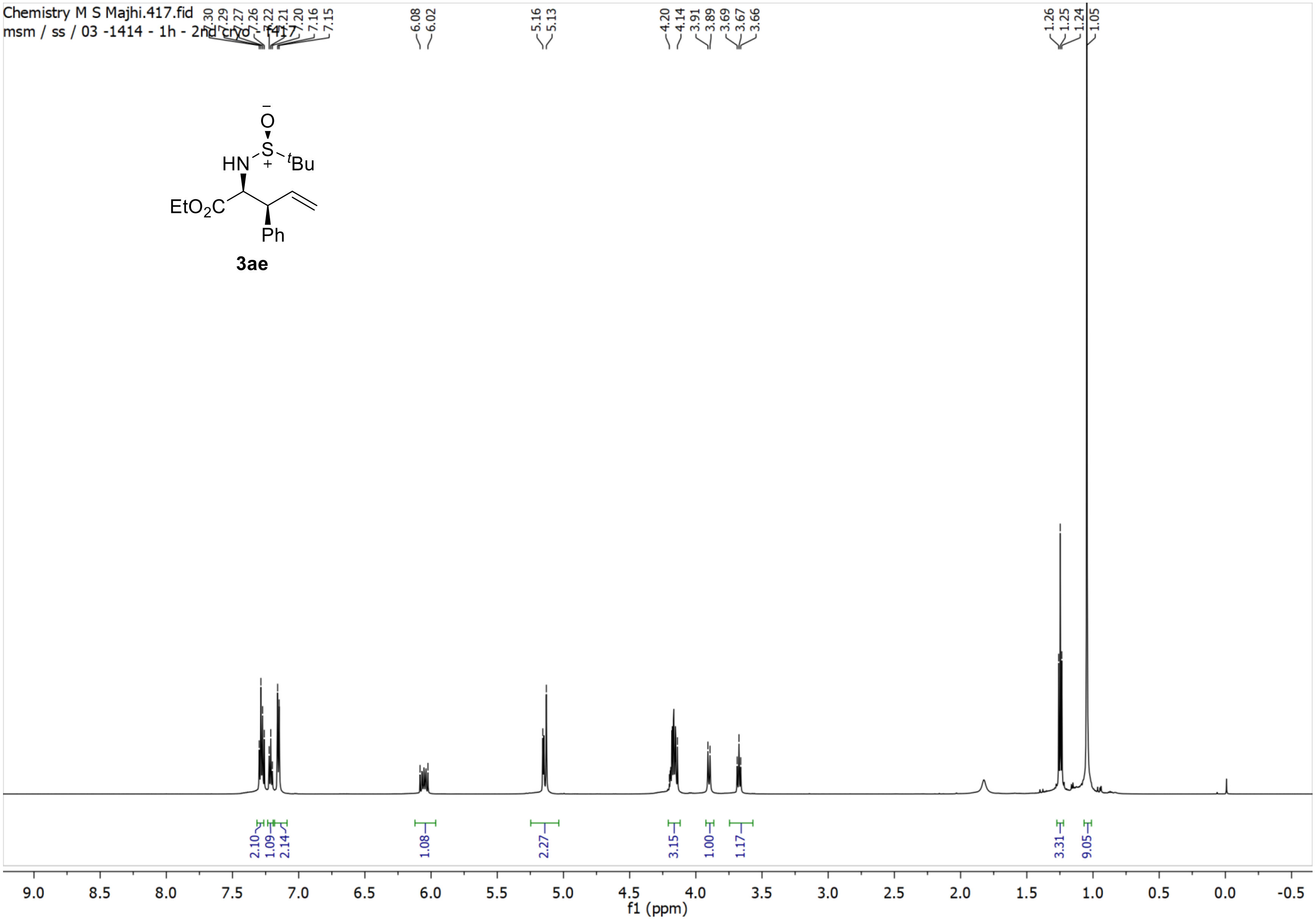
Chemistry M S Majhi.417.fid
msm / ss / 03 -1414 - 1h - 2nd cryo - f417
HN
O
S
tBu
EtO2C
Ph
3ae
f1 (ppm)

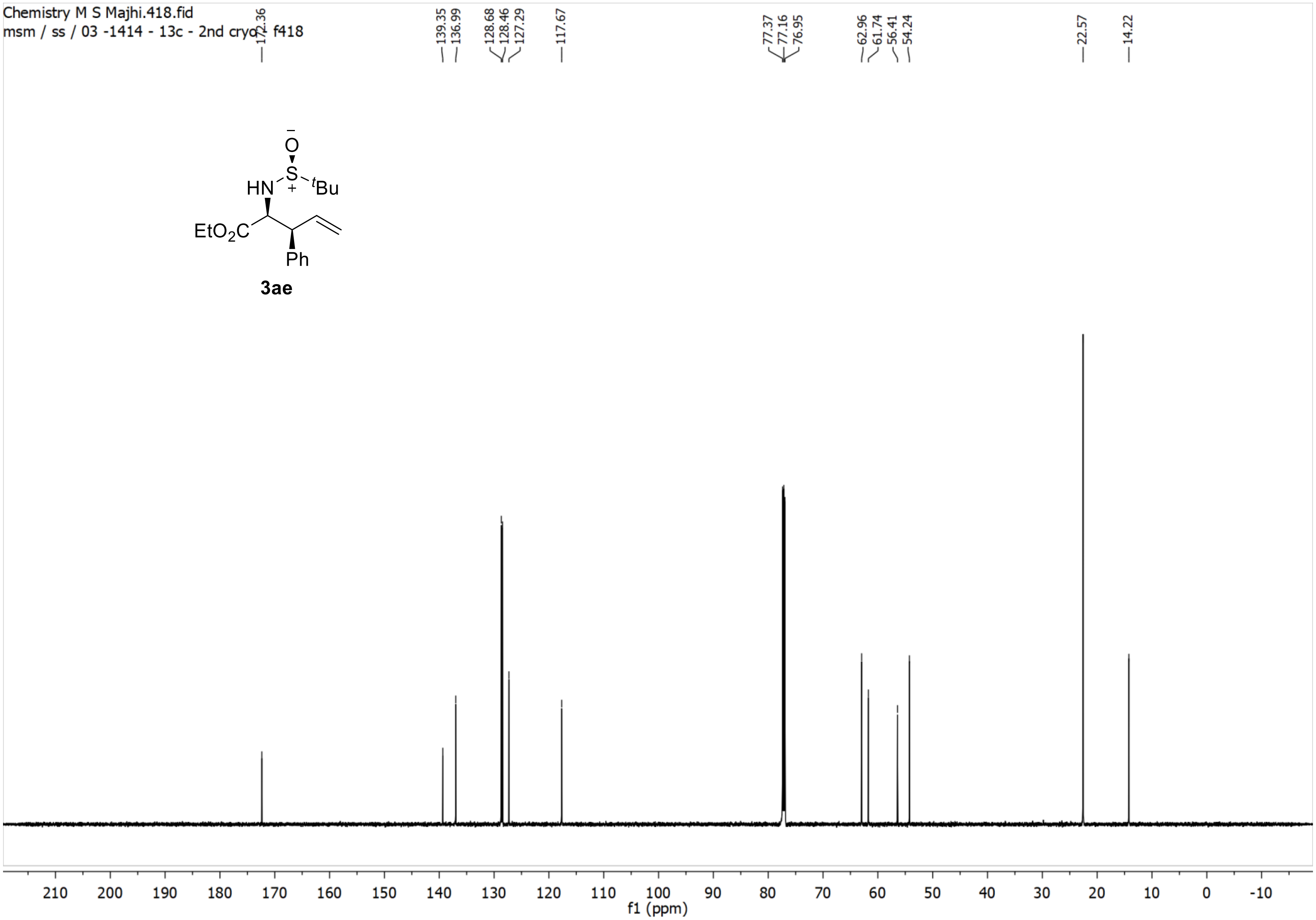
Chemistry M S Majhi.418.fid
msm / ss / 03 -1414 - 13c - 2nd cryo - f418
172.36
139.35
136.99
128.68
128.46
127.29
117.67
77.37
77.16
76.95
62.96
61.74
56.41
54.24
22.57
14.22
O
HN
S
tBu
EtO2C
Ph
210
200
190
180
170
160
150
140
130
120
110
100
90
80
70
60
50
40
30
20
10
0
-10
f1 (ppm)

3ae

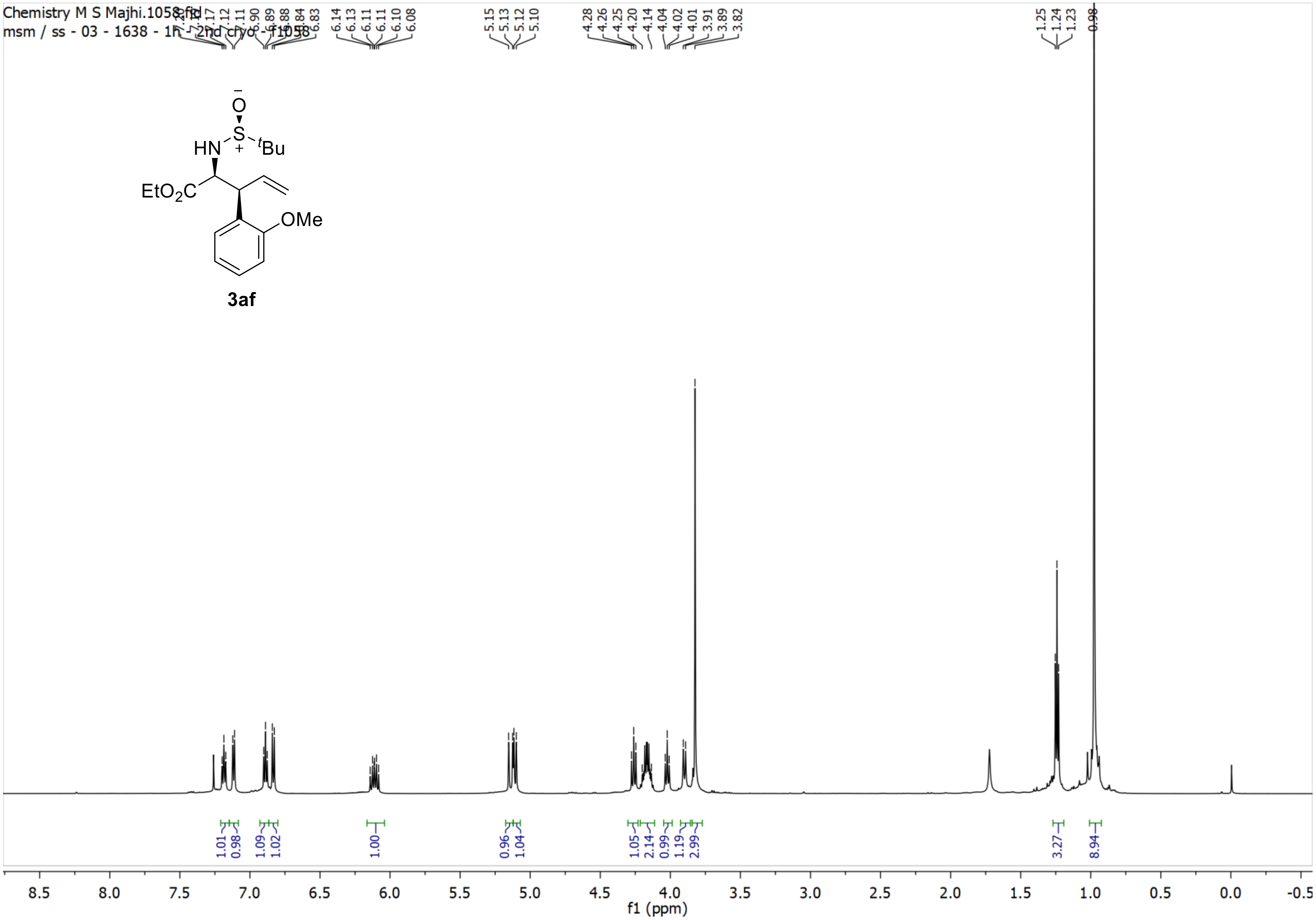

Chemistry M S Majhi.1058.fid
msm / ss - 03 - 1638 - 1h - 2nd cryo - f1058
6.14
6.13
6.11
6.11
6.10
6.08
5.15
5.13
5.12
5.10
4.28
4.26
4.25
4.20
4.14
4.04
4.02
4.01
3.91
3.89
3.82
1.25
1.24
1.23
0.98
HN
S
tBu
O
EtO2C
OMe
3af
1.01
0.98
1.09
1.02
1.00
0.96
1.04
1.05
2.14
0.99
1.19
2.99
3.27
8.94
f1 (ppm)

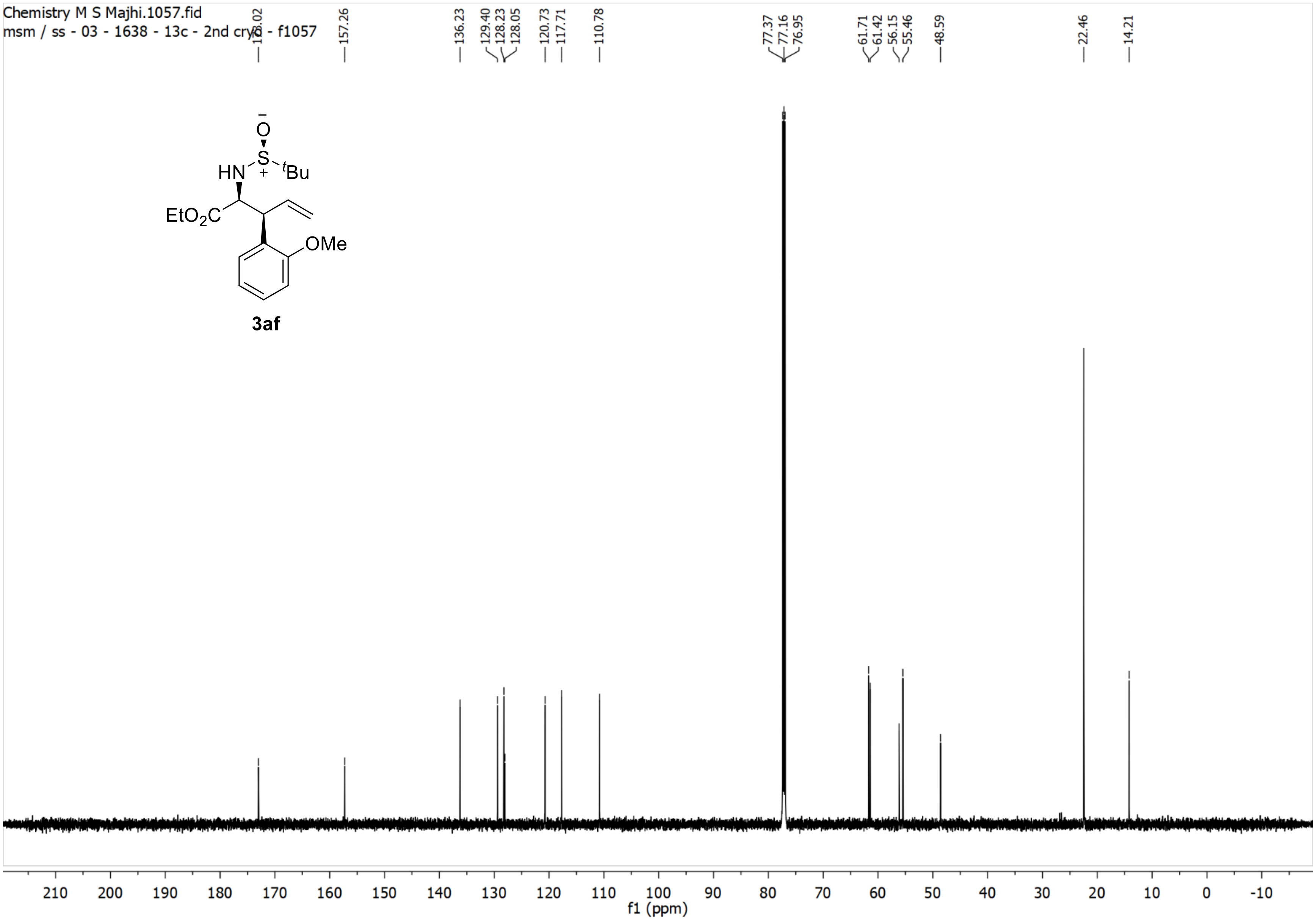
Chemistry M S Majhi.1057.fid
msm / ss - 03 - 1638 - 13c - 2nd cryst - f1057
172.02
157.26
136.23
129.40
128.23
128.05
120.73
117.71
110.78
77.37
77.16
76.95
61.71
61.42
56.15
55.46
48.59
22.46
14.21
HN
S
O
tBu
EtO2C
OMe
3af
f1 (ppm)

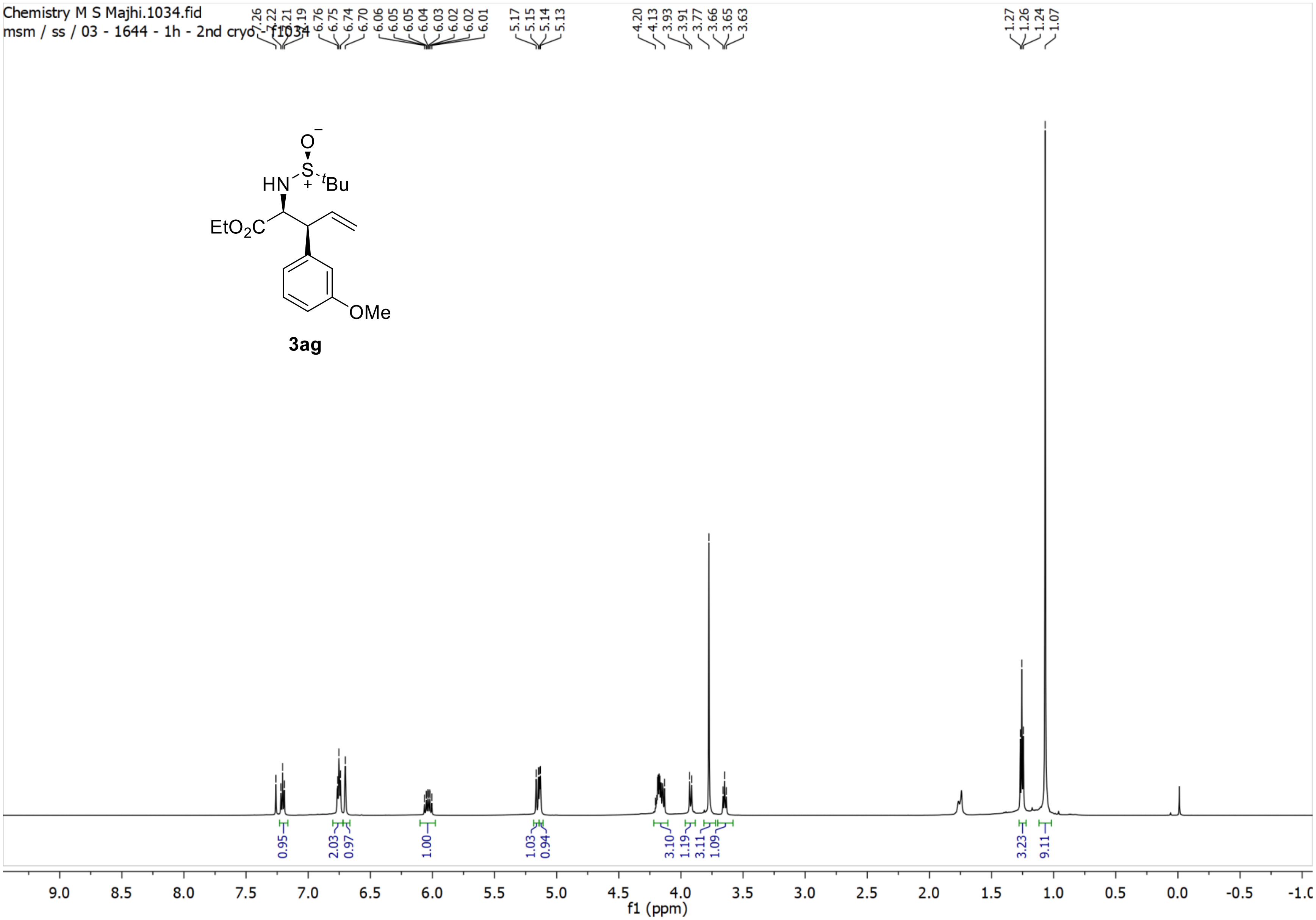

Chemistry M S Majhi.1034.fid
msm / ss / 03 - 1644 - 1h - 2nd cryo - f1034
7.26
7.22
7.21
7.19
6.76
6.75
6.74
6.70
6.06
6.05
6.05
6.04
6.03
6.02
6.02
6.01
5.17
5.15
5.14
5.13
4.20
4.13
3.93
3.91
3.77
3.66
3.65
3.63
1.27
1.26
1.24
1.07
HN
EtO2C
tBu
OMe
3ag
0.95
2.03
0.97
1.00
1.03
0.94
3.10
1.19
3.11
1.09
3.23
9.11
f1 (ppm)

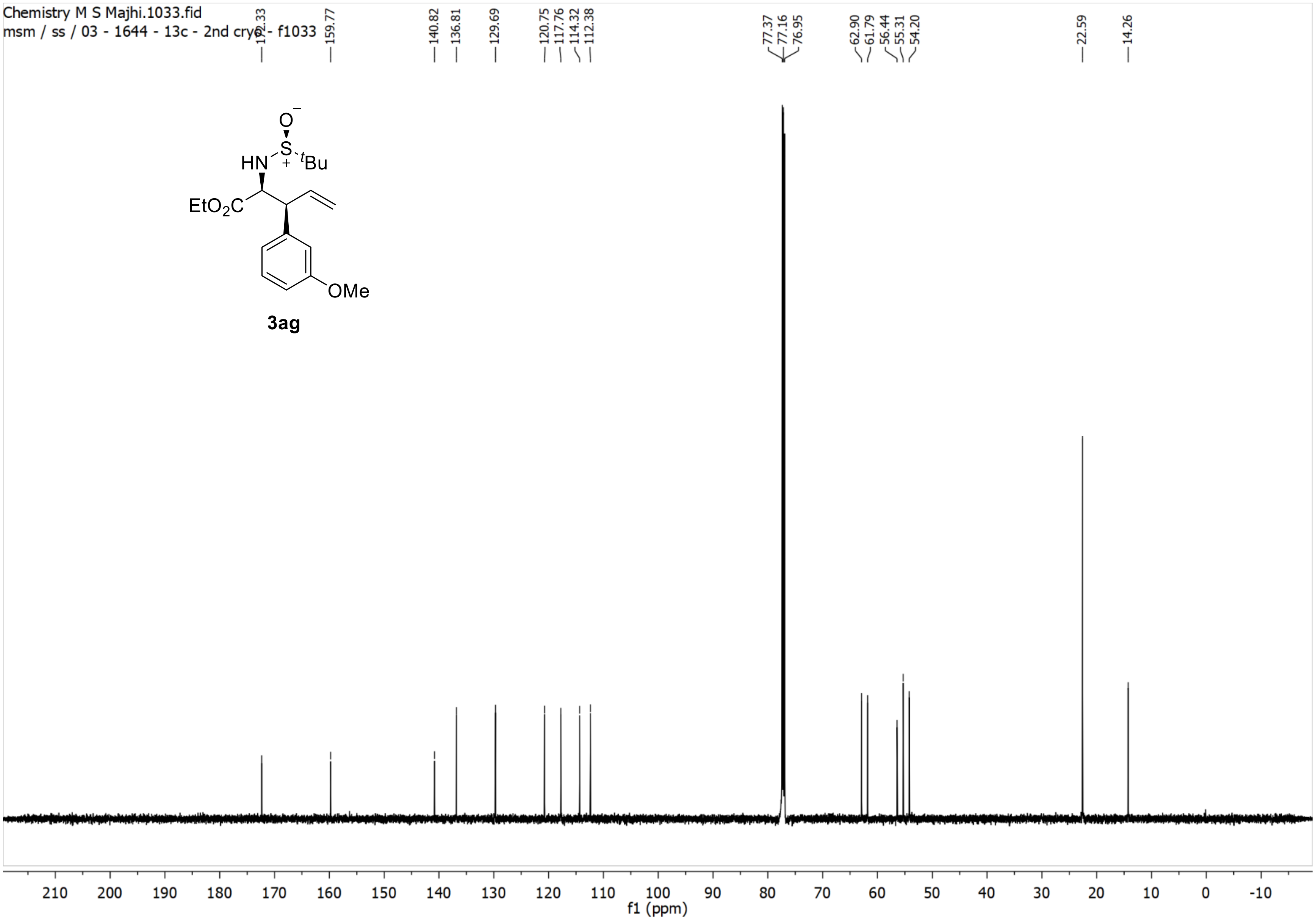

Chemistry M S Majhi.1033.fid
msm / ss / 03 - 1644 - 13c - 2nd cryo - f1033
172.33
159.77
140.82
136.81
129.69
120.75
117.76
114.32
112.38
77.37
77.16
76.95
62.90
61.79
56.44
55.31
54.20
22.59
14.26
O
HN
S
tBu
EtO2C
OMe
3ag
210 200 190 180 170 160 150 140 130 120 110 100 90 80 70 60 50 40 30 20 10 0 -10
f1 (ppm)

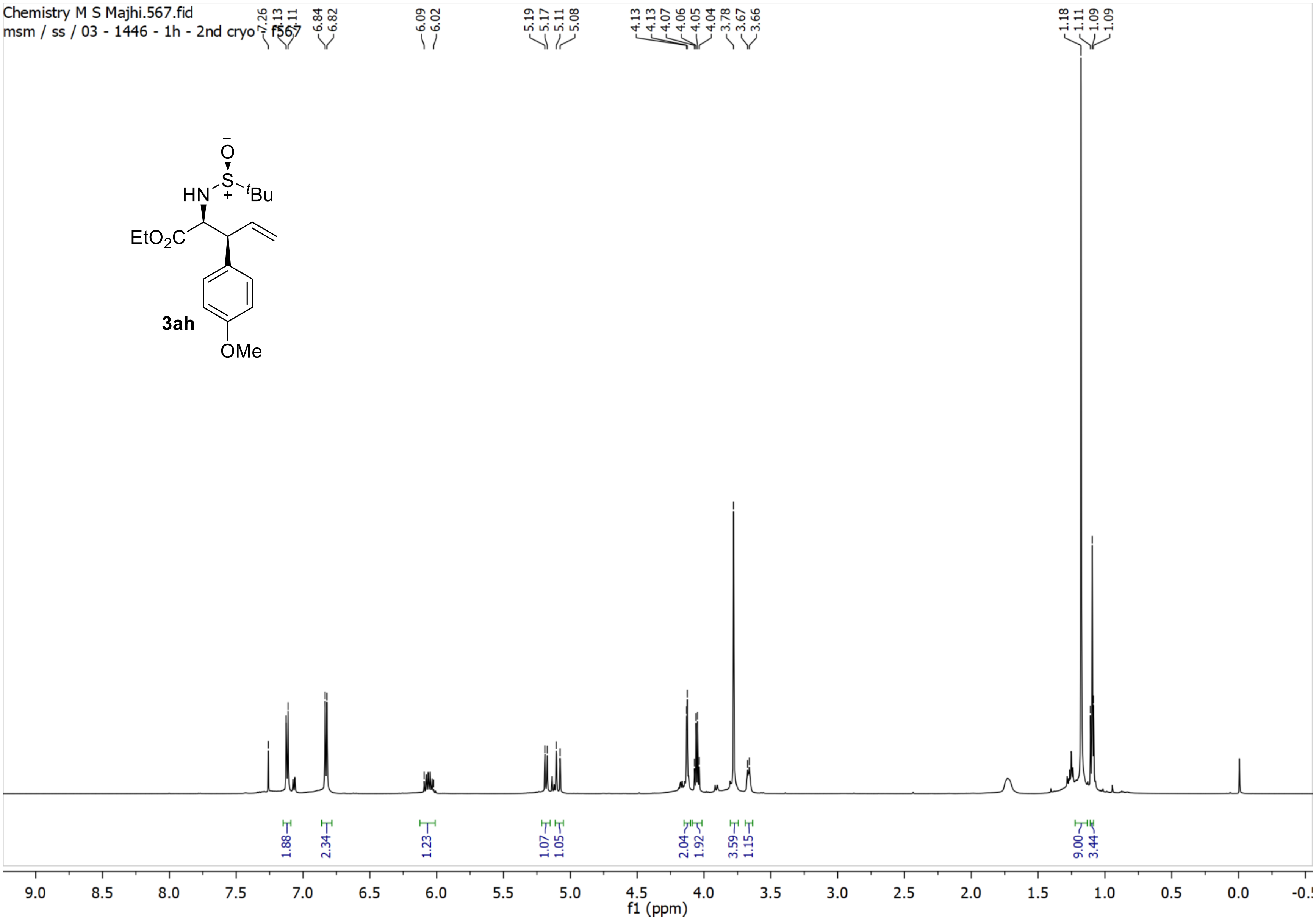

Chemistry M S Majhi.567.fid
msm / ss / 03 - 1446 - 1h - 2nd cryo - f567
7.26
7.13
7.11
6.84
6.82
6.09
6.02
5.19
5.17
5.11
5.08
4.13
4.13
4.07
4.06
4.05
4.04
3.78
3.67
3.66
1.18
1.11
1.09
1.09
HN
O
S
tBu
EtO2C
3ah
OMe
1.88
2.34
1.23
1.07
1.05
2.04
1.92
3.59
1.15
9.00
3.44
f1 (ppm)

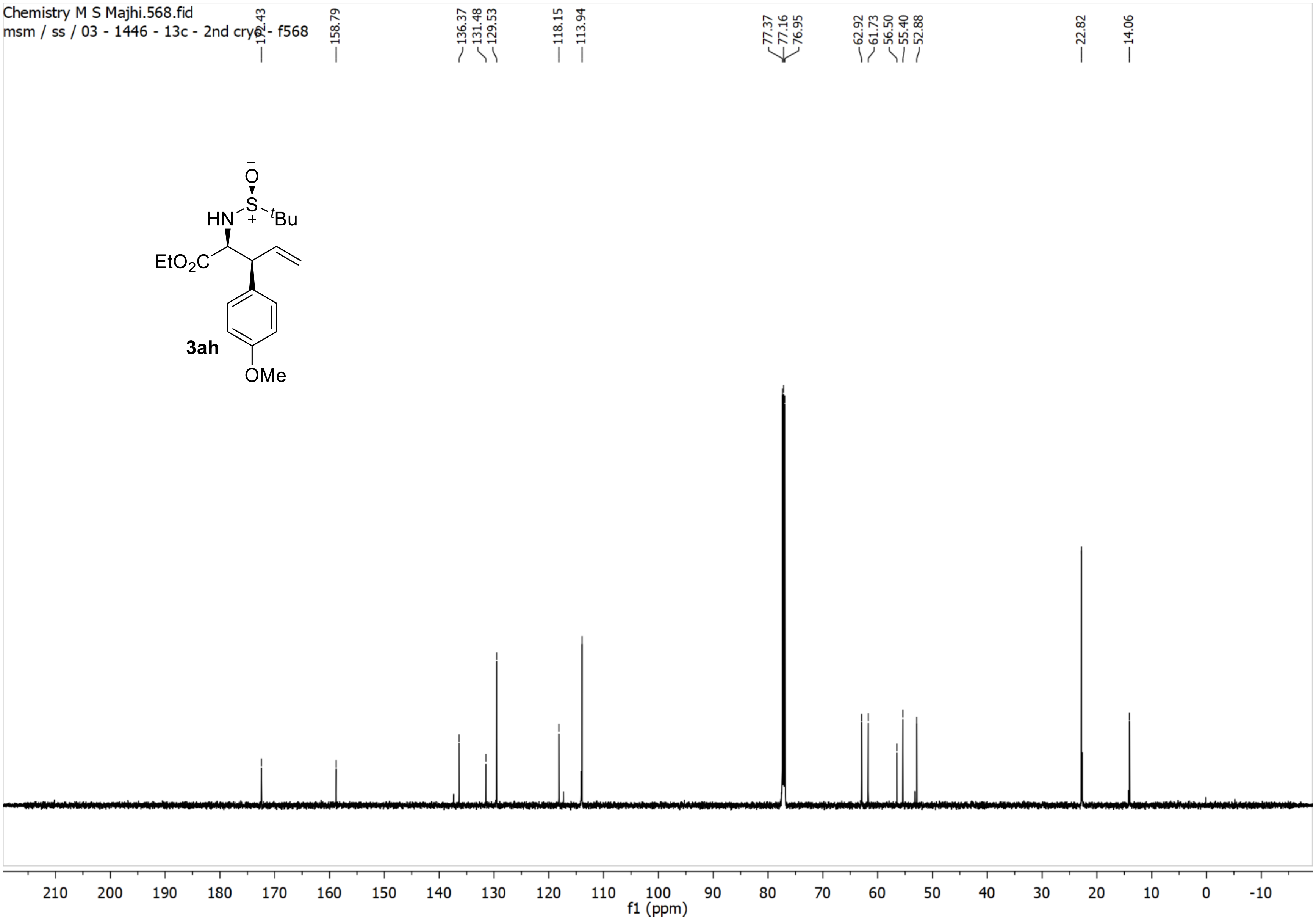

Chemistry M S Majhi.568.fid
msm / ss / 03 - 1446 - 13c - 2nd cryo - f568
172.43
158.79
136.37
131.48
129.53
118.15
113.94
77.37
77.16
76.95
62.92
61.73
56.50
55.40
52.88
22.82
14.06
HN
S
tBu
EtO2C
OMe
3ah
210 200 190 180 170 160 150 140 130 120 110 100 90 80 70 60 50 40 30 20 10 0 -10
f1 (ppm)

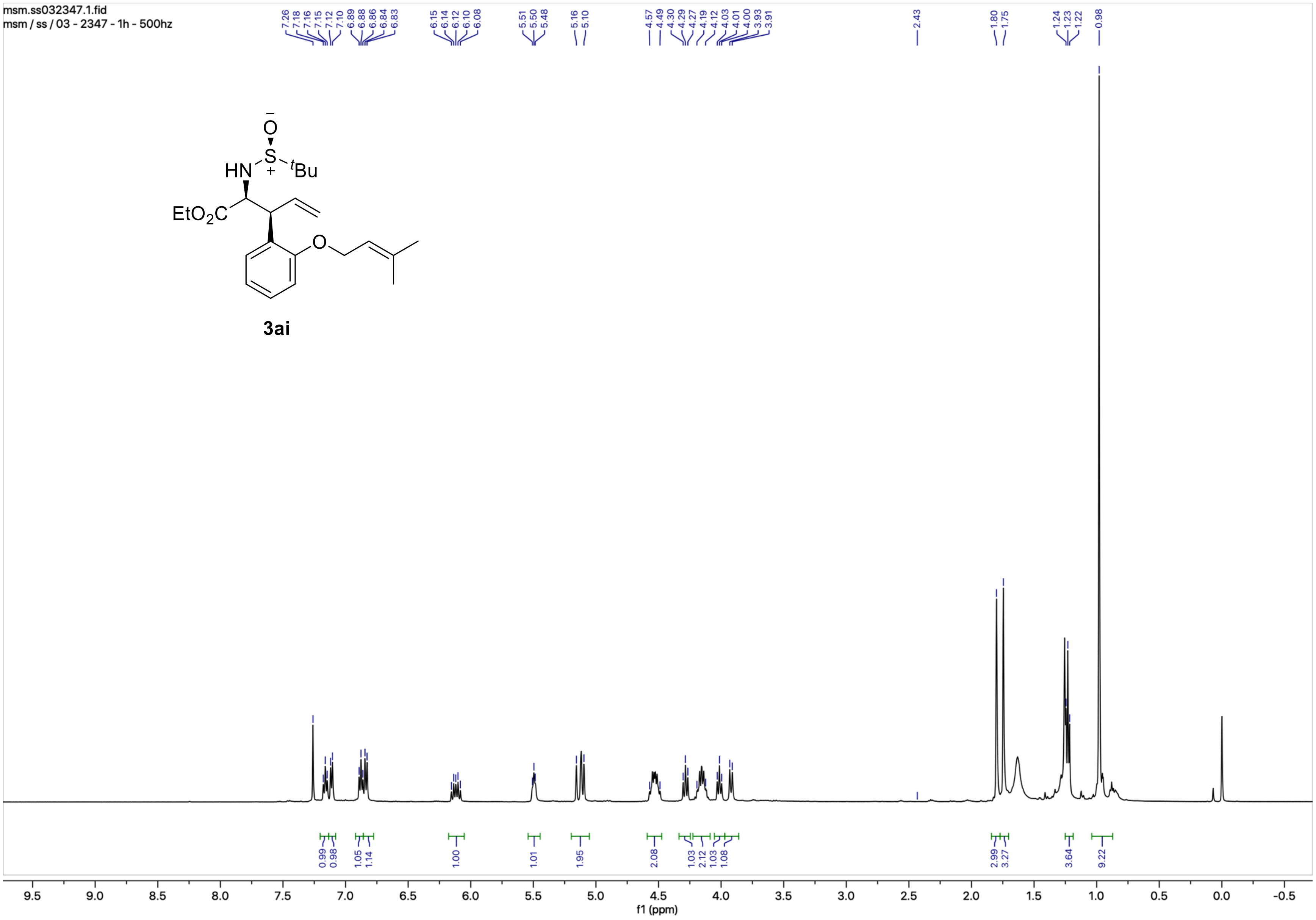
msm.ss032347.1.fid
msm / ss / 03 - 2347 - 1h - 500hz
7.26 7.18 7.16 7.15 7.12 7.10 6.89 6.88 6.86 6.84 6.83
6.15 6.14 6.12 6.10 6.08
5.51 5.50 5.48
5.16 5.10
4.57 4.49 4.30 4.29 4.27 4.19 4.12 4.03 4.01 4.00 3.93 3.91
2.43
1.80 1.75
1.24 1.23 1.22
0.98
0.99 0.98 1.05 1.14 1.00 1.01 1.95 2.08 1.03 2.12 1.03 1.08 2.99 3.27 3.64 9.22
HN
tBu
EtO2C
O
3ai
f1 (ppm)

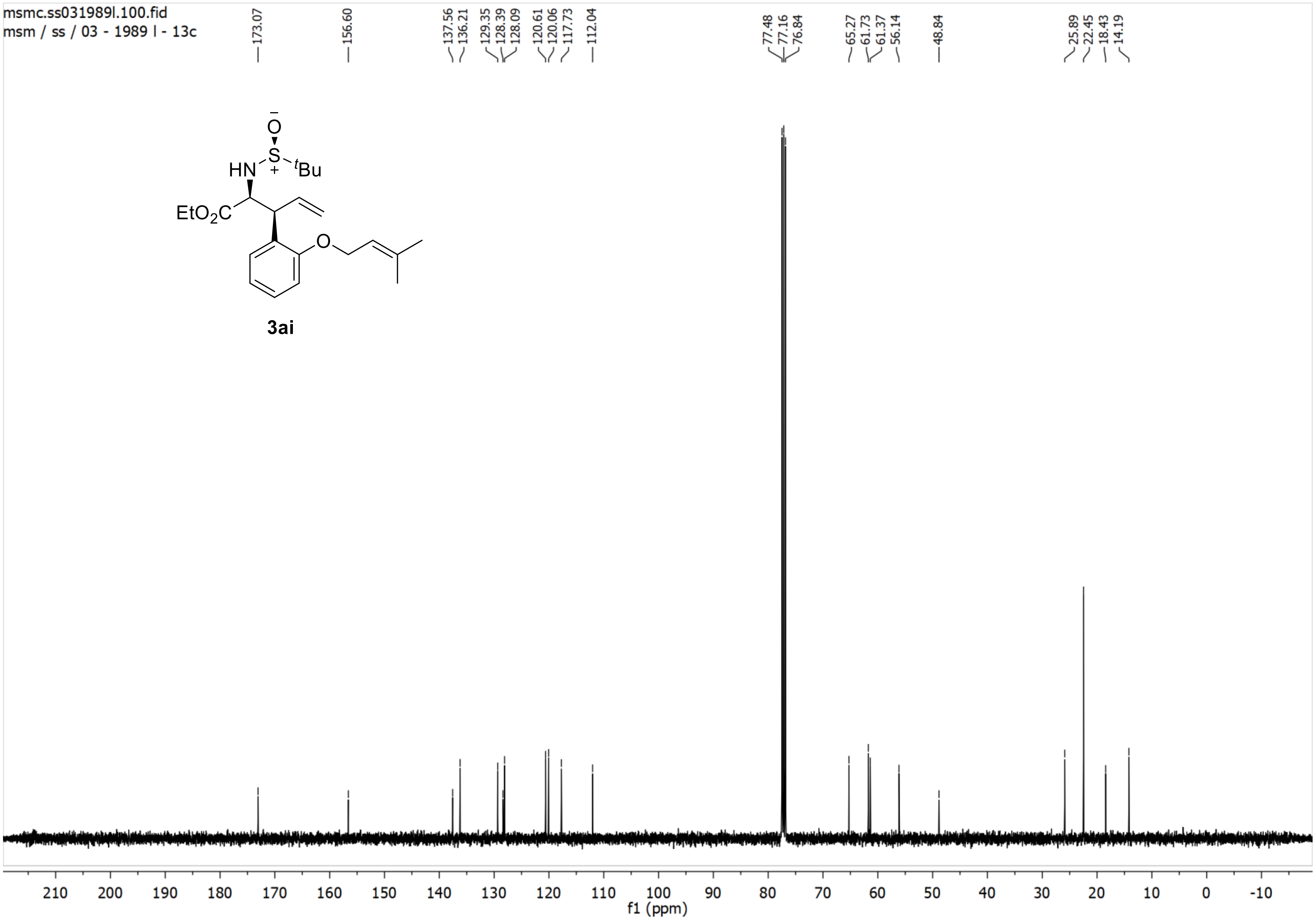
msmc.ss031989l.100.fid
msm / ss / 03 - 1989 l - 13c
173.07
156.60
137.56
136.21
129.35
128.39
128.09
120.61
120.06
117.73
112.04
77.48
77.16
76.84
65.27
61.73
61.37
56.14
48.84
25.89
22.45
18.43
14.19
3ai
210 200 190 180 170 160 150 140 130 120 110 100 90 80 70 60 50 40 30 20 10 0 -10
f1 (ppm)

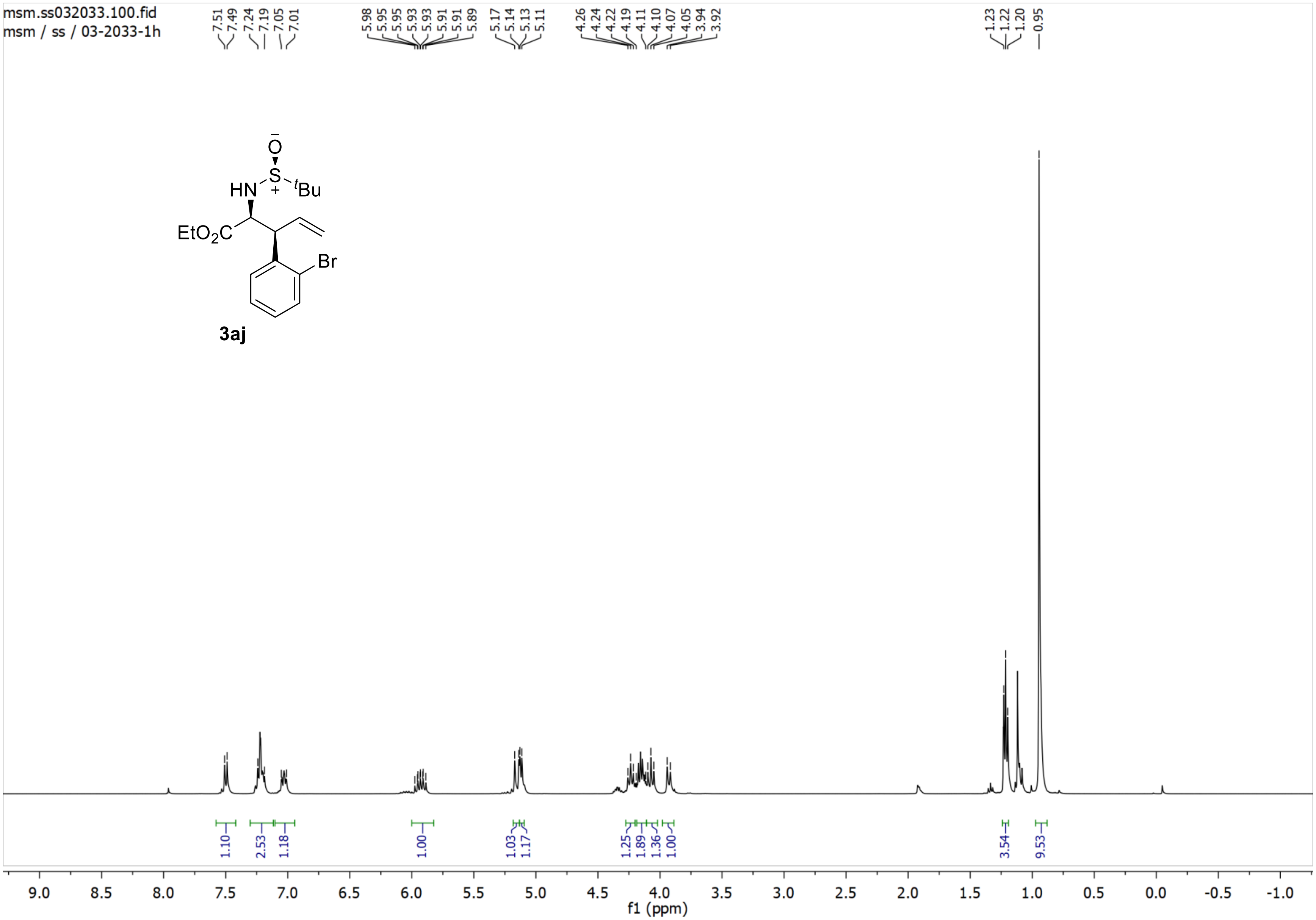
msm.ss032033.100.fid
msm / ss / 03-2033-1h
7.51
7.49
7.24
7.19
7.05
7.01
5.98
5.95
5.95
5.93
5.93
5.91
5.91
5.89
5.17
5.14
5.13
5.11
4.26
4.24
4.22
4.19
4.11
4.10
4.07
4.05
3.94
3.92
1.23
1.22
1.20
0.95
HN
O
S
+
tBu
EtO2C
Br
1.10
2.53
1.18
1.00
1.03
1.17
1.25
1.89
1.36
1.00
3.54
9.53
9.0
8.5
8.0
7.5
7.0
6.5
6.0
5.5
5.0
4.5
4.0
3.5
3.0
2.5
2.0
1.5
1.0
0.5
0.0
-0.5
-1.0
f1 (ppm)

3aj

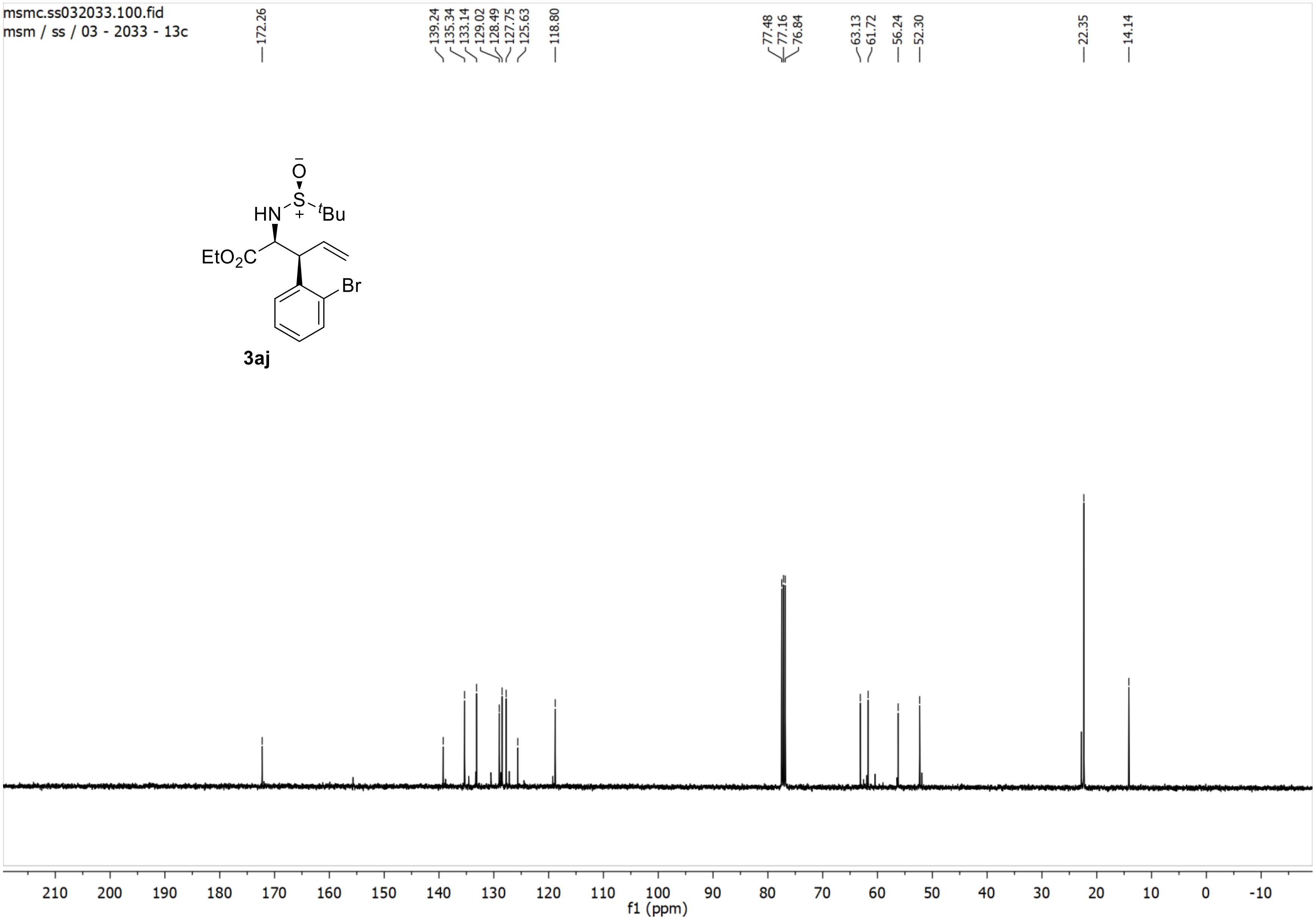
msmc.ss032033.100.fid
msm / ss / 03 - 2033 - 13c
172.26
139.24
135.34
133.14
129.02
128.49
127.75
125.63
118.80
77.48
77.16
76.84
63.13
61.72
56.24
52.30
22.35
14.14
HN
S
O
tBu
EtO2C
Br
3aj
210 200 190 180 170 160 150 140 130 120 110 100 90 80 70 60 50 40 30 20 10 0 -10
f1 (ppm)

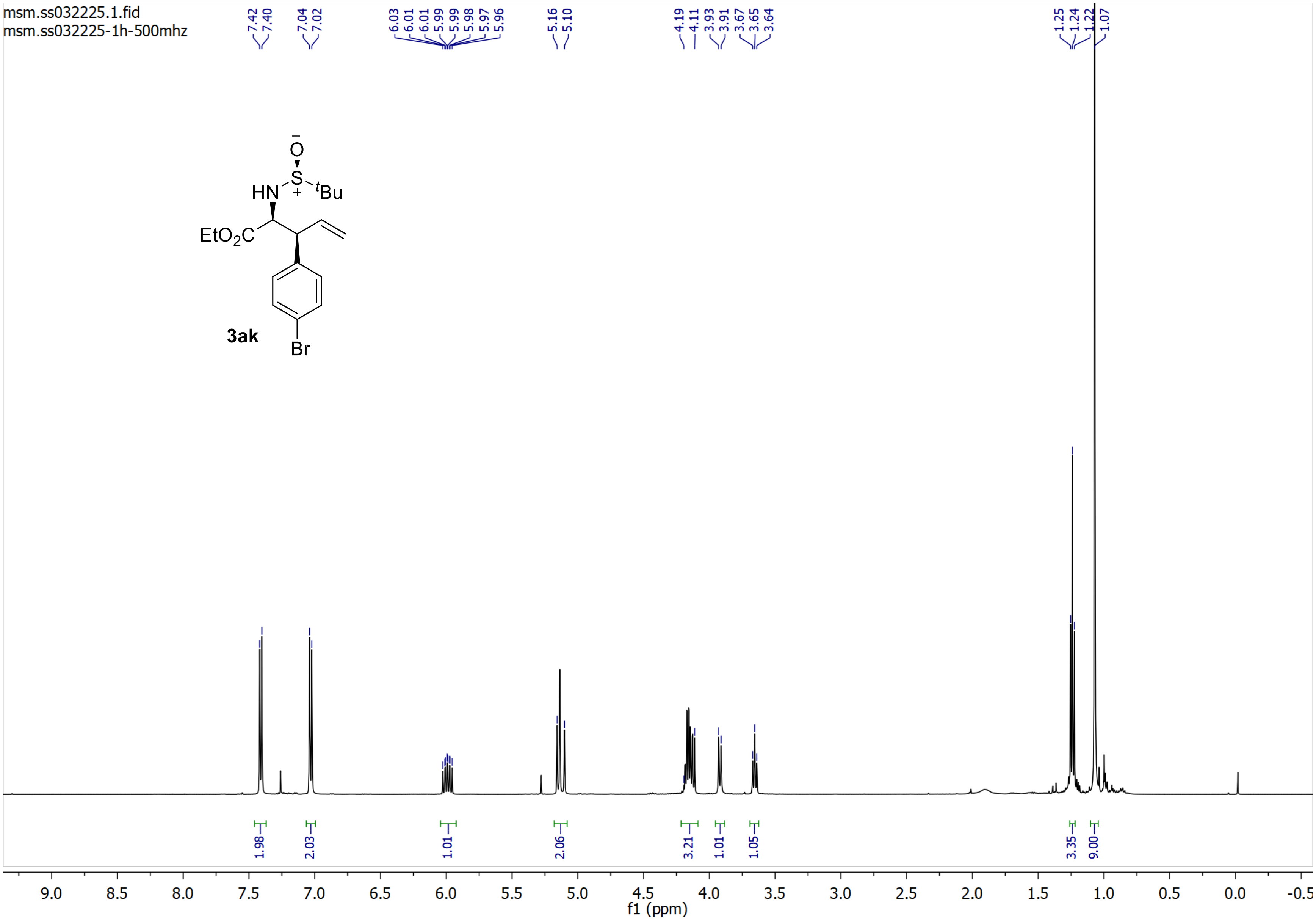

msm.ss032225.1.fid
msm.ss032225-1h-500mhz
7.42
7.40
7.04
7.02
6.03
6.01
6.01
5.99
5.99
5.98
5.97
5.96
5.16
5.10
4.19
4.11
3.93
3.91
3.67
3.65
3.64
1.25
1.24
1.22
1.07
HN
S
+
O
tBu
EtO2C
Br
3ak
1.98
2.03
1.01
2.06
3.21
1.01
1.05
3.35
9.00
9.0
8.5
8.0
7.5
7.0
6.5
6.0
5.5
5.0
4.5
4.0
3.5
3.0
2.5
2.0
1.5
1.0
0.5
0.0
-0.5
f1 (ppm)

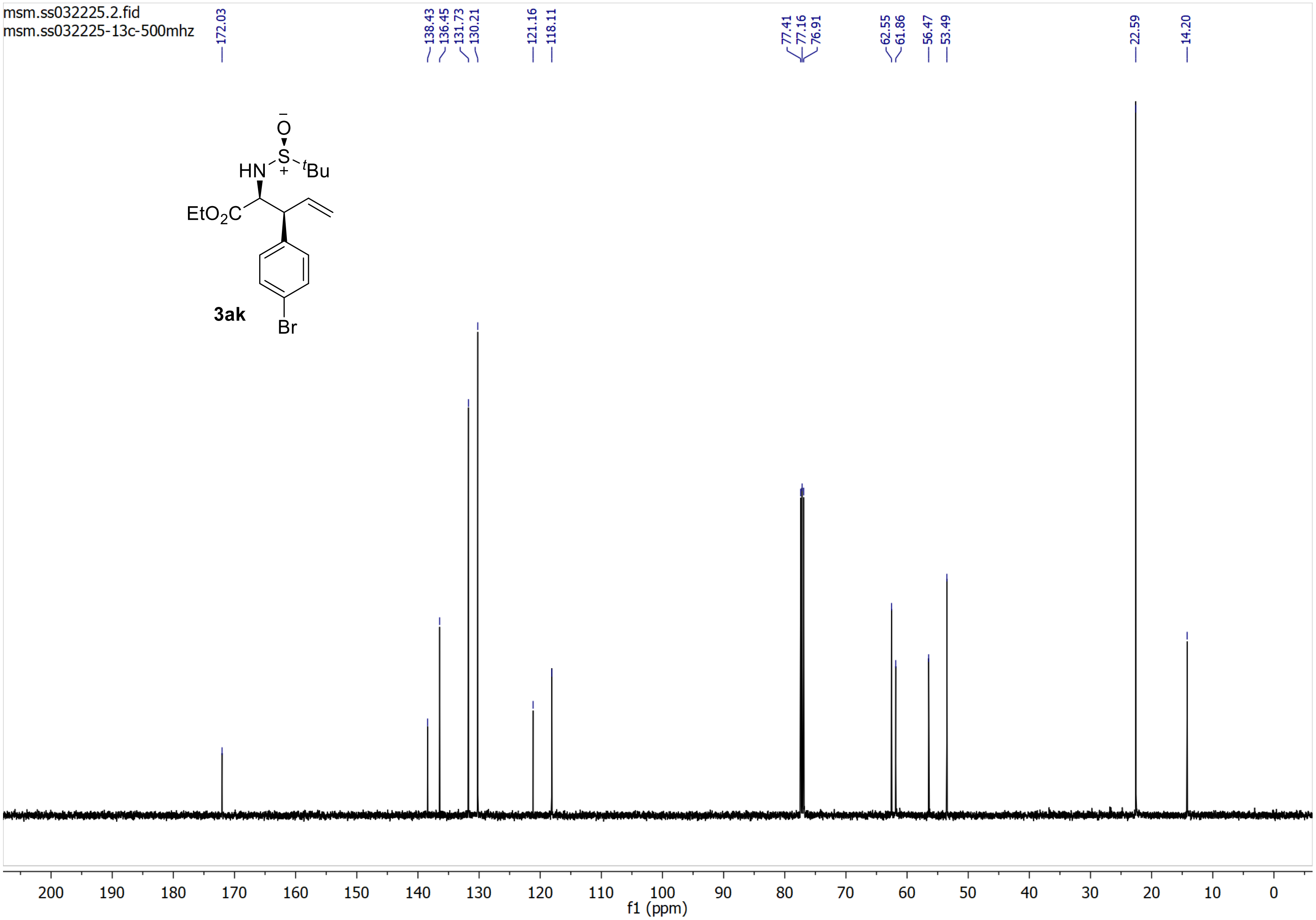
msm.ss032225.2.fid
msm.ss032225-13c-500mhz
172.03
138.43
136.45
131.73
130.21
121.16
118.11
77.41
77.16
76.91
62.55
61.86
56.47
53.49
22.59
14.20
HN
O
S
tBu
EtO2C
Br
3ak
200 190 180 170 160 150 140 130 120 110 100 90 80 70 60 50 40 30 20 10 0
f1 (ppm)

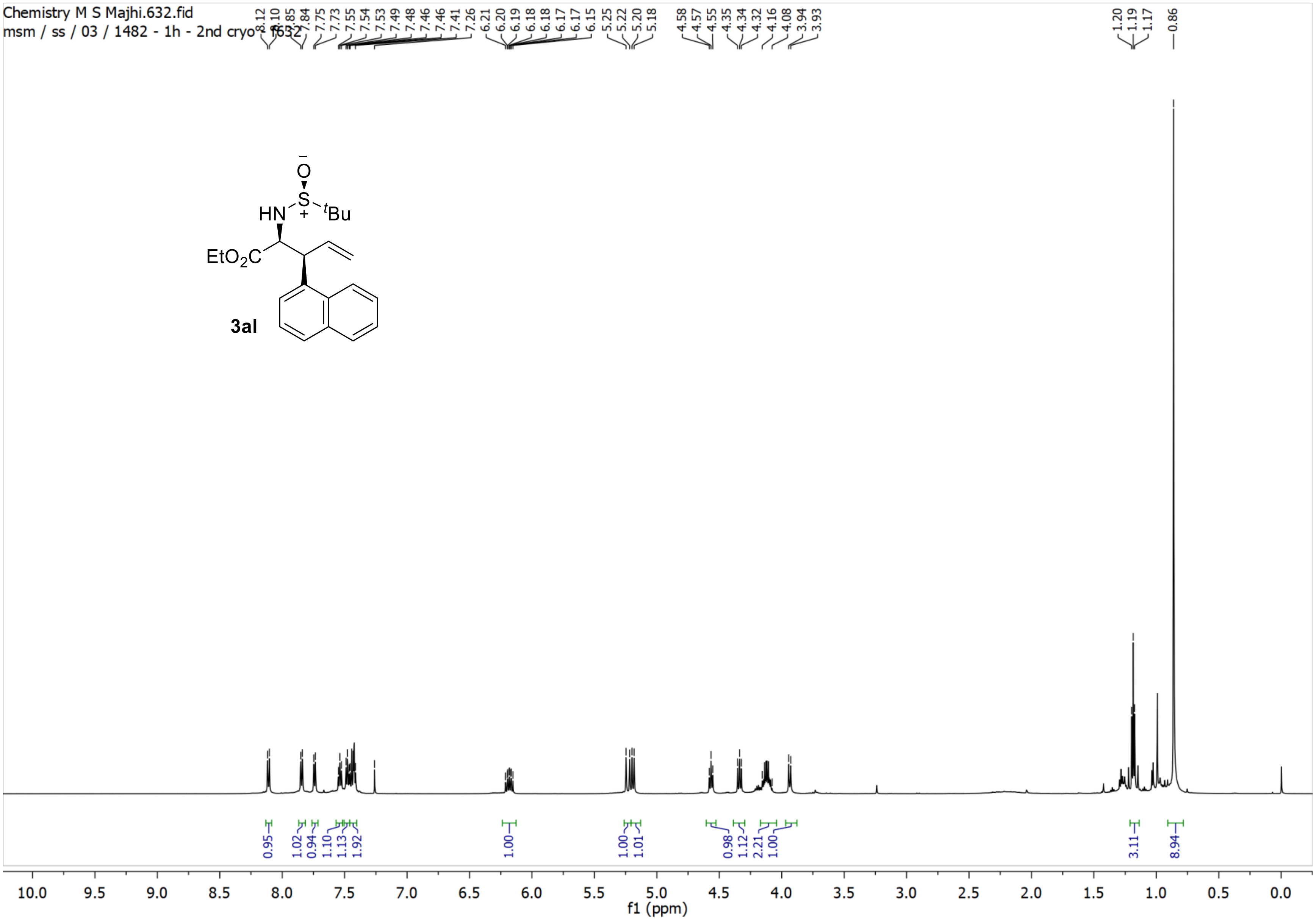
Chemistry M S Majhi.632.fid
msm / ss / 03 / 1482 - 1h - 2nd cryo - f632
3al
f1 (ppm)

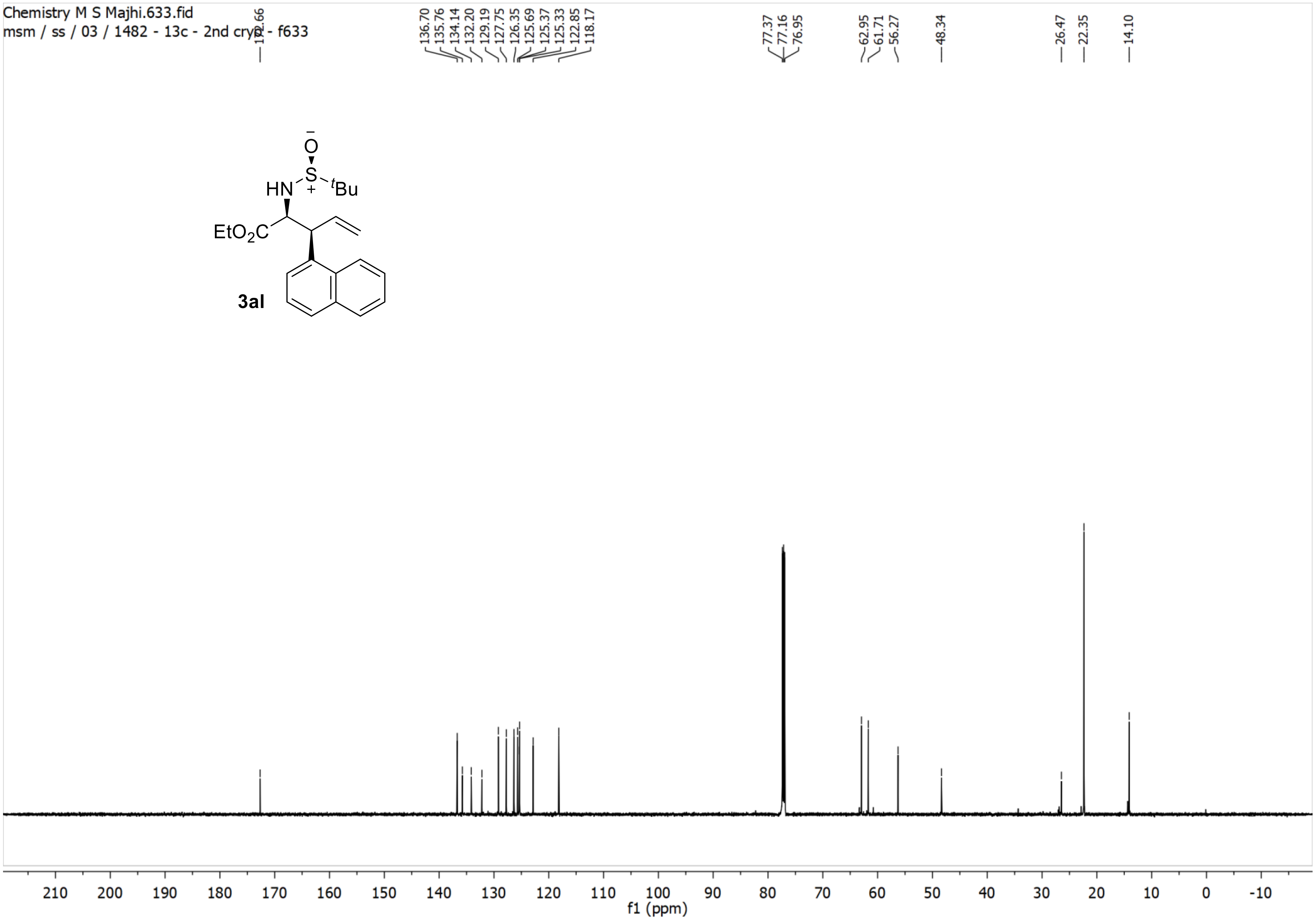
Chemistry M S Majhi.633.fid
msm / ss / 03 / 1482 - 13c - 2nd cryo - f633
172.66
136.70
135.76
134.14
132.20
129.19
127.75
126.35
125.69
125.37
125.33
122.85
118.17
77.37
77.16
76.95
62.95
61.71
56.27
48.34
26.47
22.35
14.10
HN
EtO2C
tBu
3al
210 200 190 180 170 160 150 140 130 120 110 100 90 80 70 60 50 40 30 20 10 0 -10
f1 (ppm)

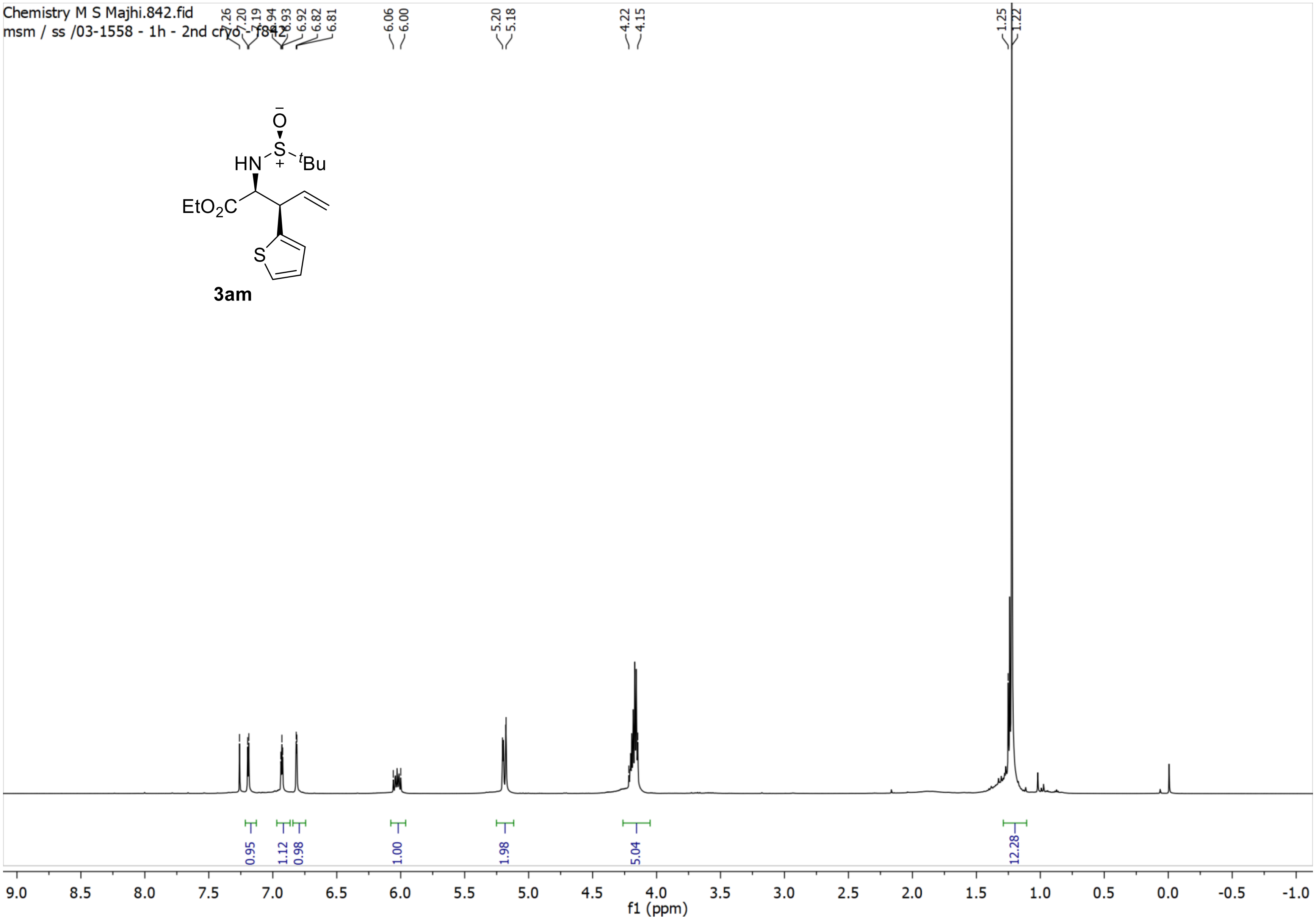

Chemistry M S Majhi.842.fid
msm / ss /03-1558 - 1h - 2nd cryo - f842
7.26
7.20
7.19
6.94
6.93
6.92
6.82
6.81
6.06
6.00
5.20
5.18
4.22
4.15
1.25
1.22
HN
S
O
tBu
EtO2C
S
3am
0.95
1.12
0.98
1.00
1.98
5.04
12.28
9.0
8.5
8.0
7.5
7.0
6.5
6.0
5.5
5.0
4.5
4.0
3.5
3.0
2.5
2.0
1.5
1.0
0.5
0.0
-0.5
-1.0
f1 (ppm)

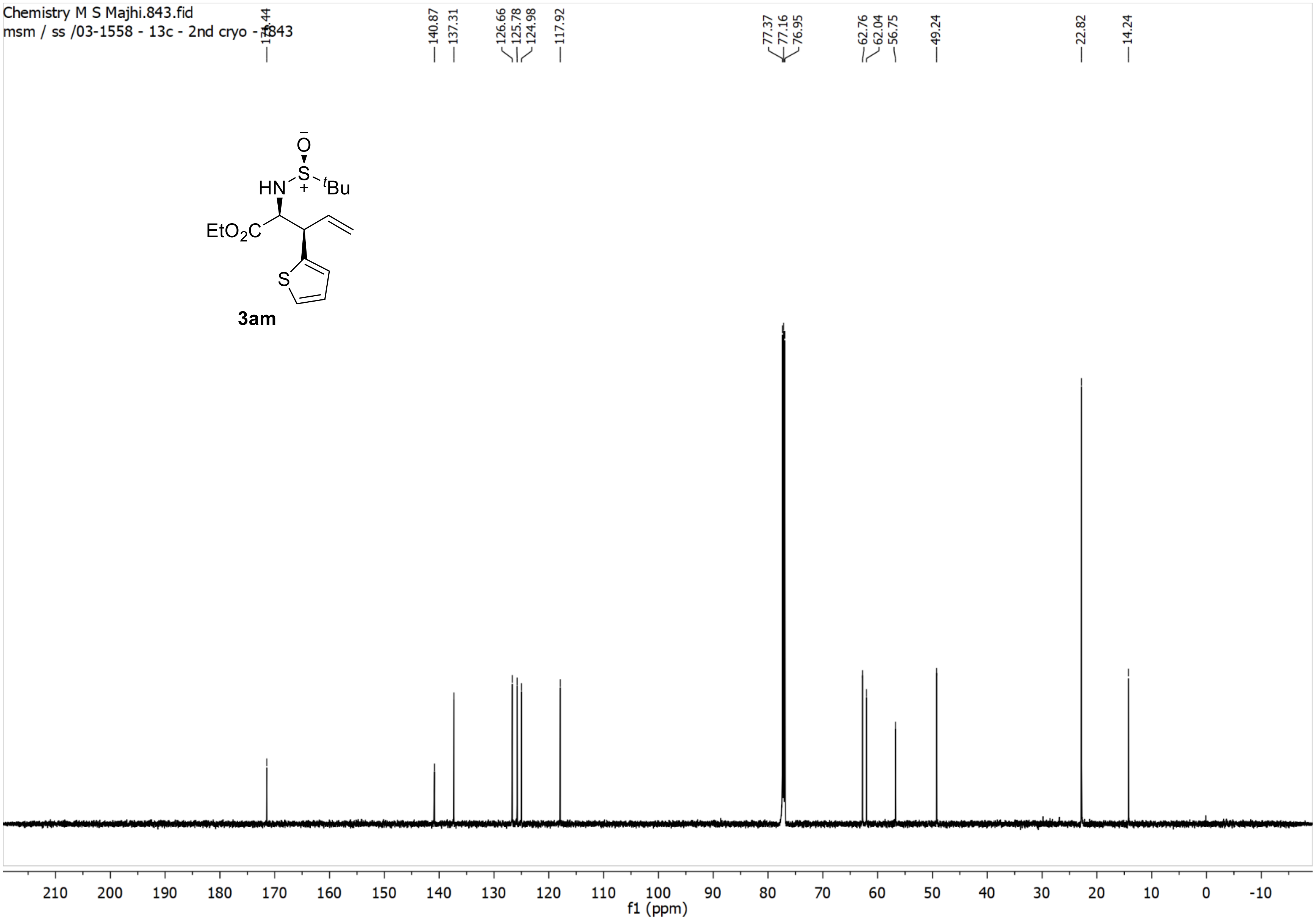

Chemistry M S Majhi.843.fid
msm / ss /03-1558 - 13c - 2nd cryo - f843
171.44
140.87
137.31
126.66
125.78
124.98
117.92
77.37
77.16
76.95
62.76
62.04
56.75
49.24
22.82
14.24
HN
S
O
tBu
EtO2C
S
3am
210 200 190 180 170 160 150 140 130 120 110 100 90 80 70 60 50 40 30 20 10 0 -10
f1 (ppm)

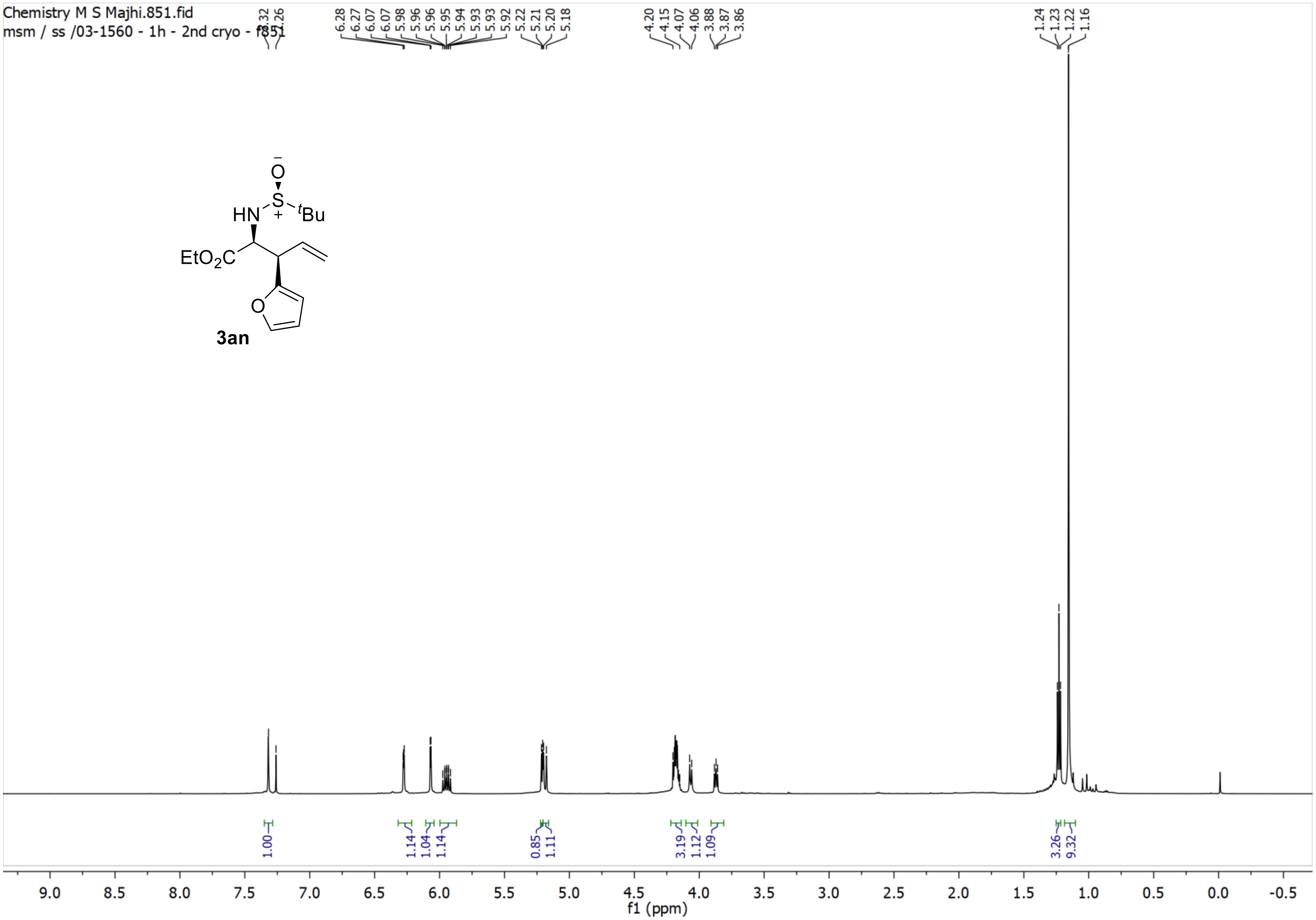
Chemistry M S Majhi.851.fid
msm / ss /03-1560 - 1h - 2nd cryo - f851
3an
f1 (ppm)

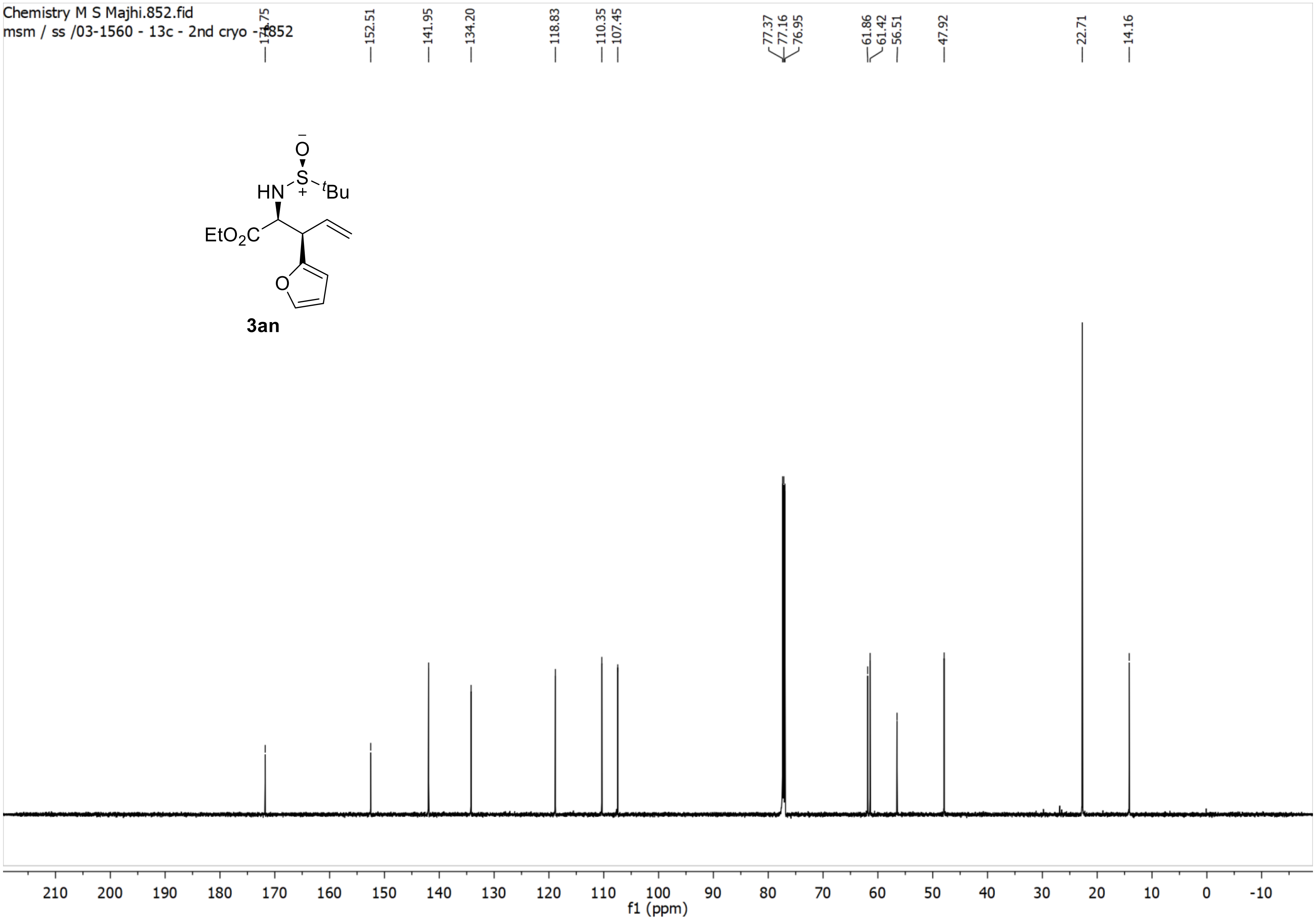

Chemistry M S Majhi.852.fid
msm / ss /03-1560 - 13c - 2nd cryo - 852
171.75
152.51
141.95
134.20
118.83
110.35
107.45
77.37
77.16
76.95
61.86
61.42
56.51
47.92
22.71
14.16
HN
S
O
tBu
EtO2C
O
3an
210 200 190 180 170 160 150 140 130 120 110 100 90 80 70 60 50 40 30 20 10 0 -10
f1 (ppm)

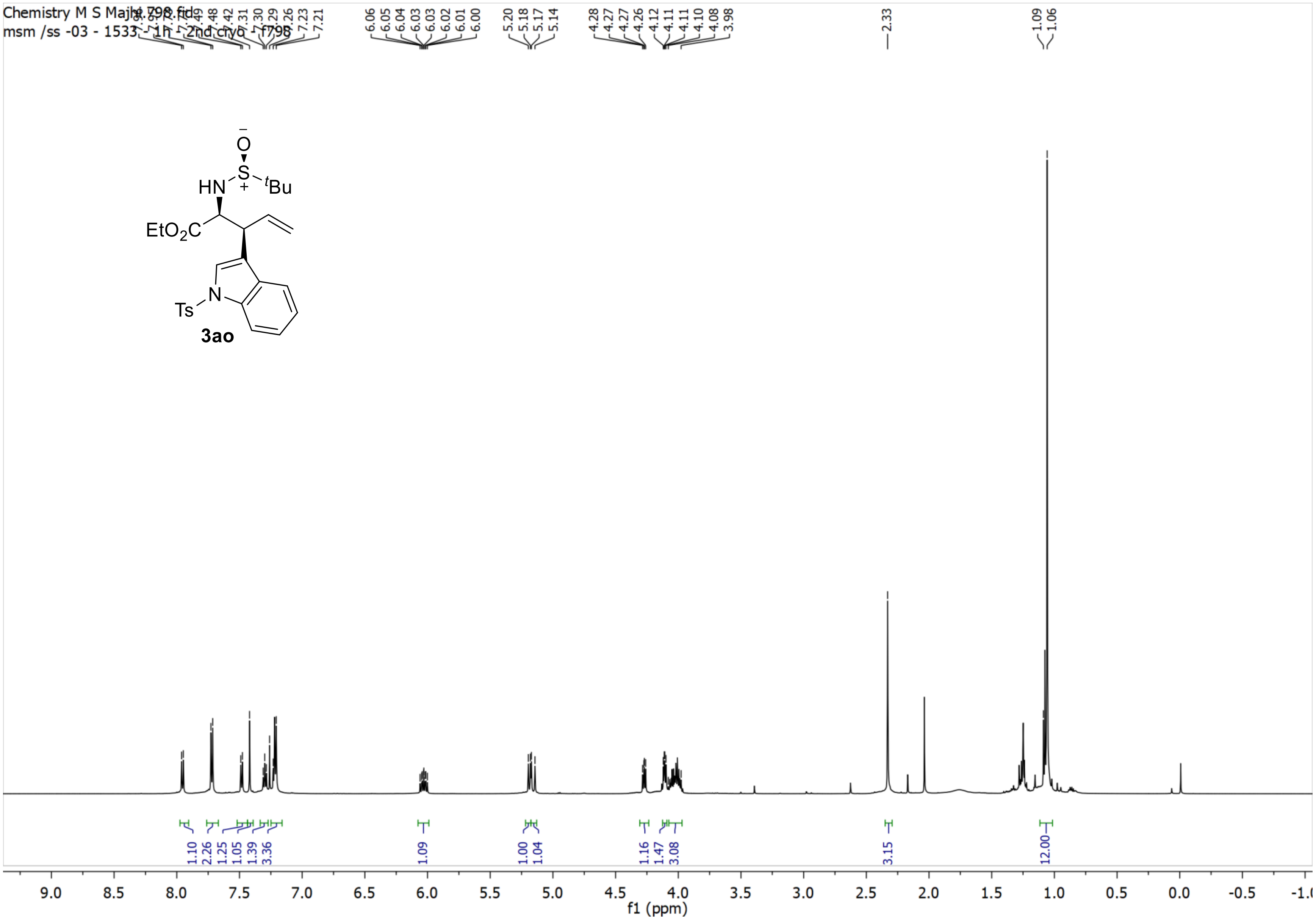
Chemistry M S Majhi 798.fid
msm /ss -03 - 1533 - 1h - 2nd chyo - f798
7.49
7.48
7.42
7.31
7.30
7.29
7.26
7.23
7.21
6.06
6.05
6.04
6.03
6.03
6.02
6.01
6.00
5.20
5.18
5.17
5.14
4.28
4.27
4.27
4.26
4.12
4.11
4.11
4.10
4.08
3.98
2.33
1.09
1.06
HN
S
+
tBu
O
EtO2C
N
Ts
3ao
1.10
2.26
1.25
1.05
1.39
3.36
1.09
1.00
1.04
1.16
1.47
3.08
3.15
12.00
9.0
8.5
8.0
7.5
7.0
6.5
6.0
5.5
5.0
4.5
4.0
3.5
3.0
2.5
2.0
1.5
1.0
0.5
0.0
-0.5
f1 (ppm)

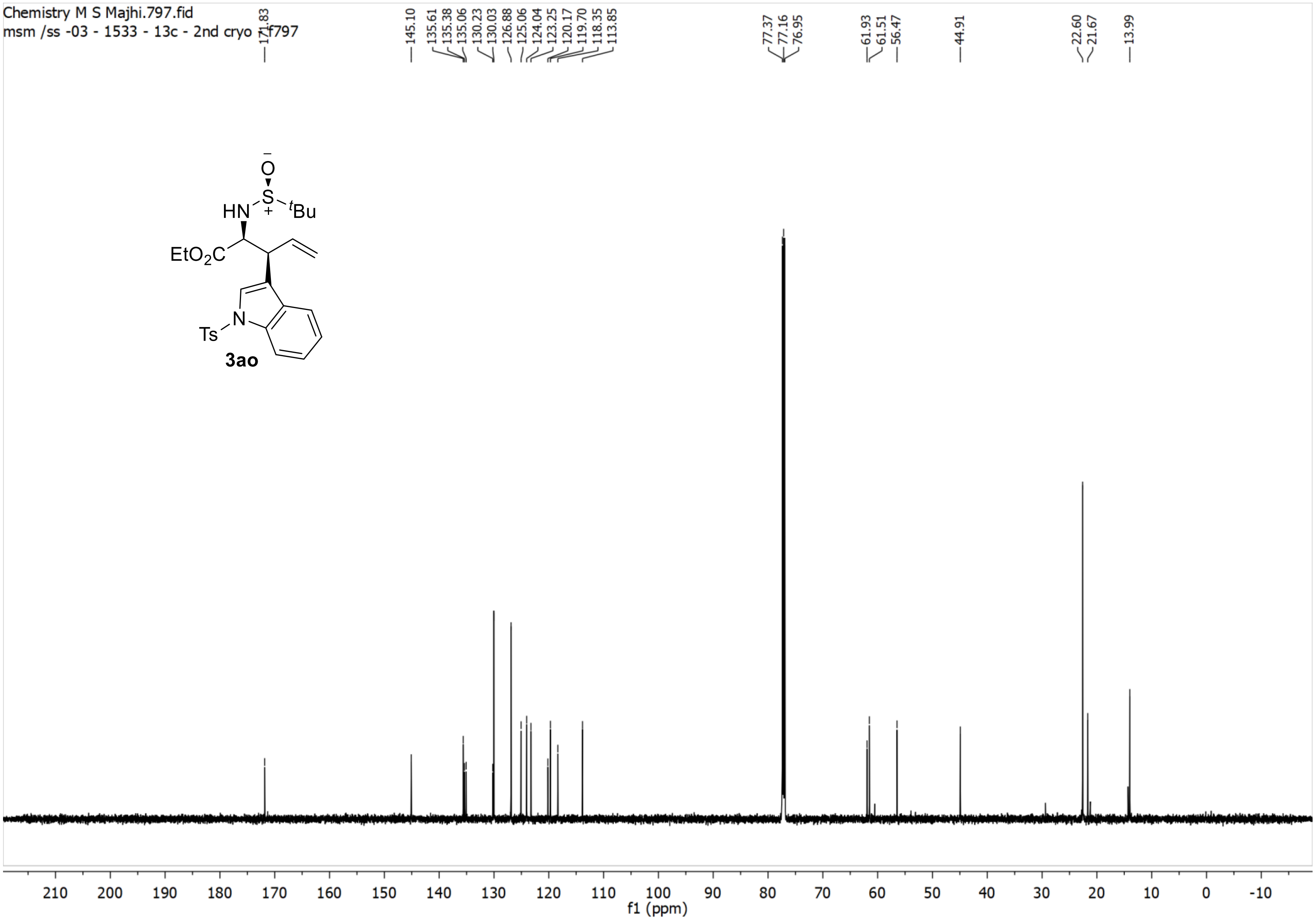
Chemistry M S Majhi.797.fid
msm /ss -03 - 1533 - 13c - 2nd cryo - f797
171.83
145.10
135.61
135.38
135.06
130.23
130.03
126.88
125.06
124.04
123.25
120.17
119.70
118.35
113.85
77.37
77.16
76.95
61.93
61.51
56.47
44.91
22.60
21.67
13.99
HN
S
O
+
−
ᵗBu
EtO₂C
N
Ts
3ao
f1 (ppm)

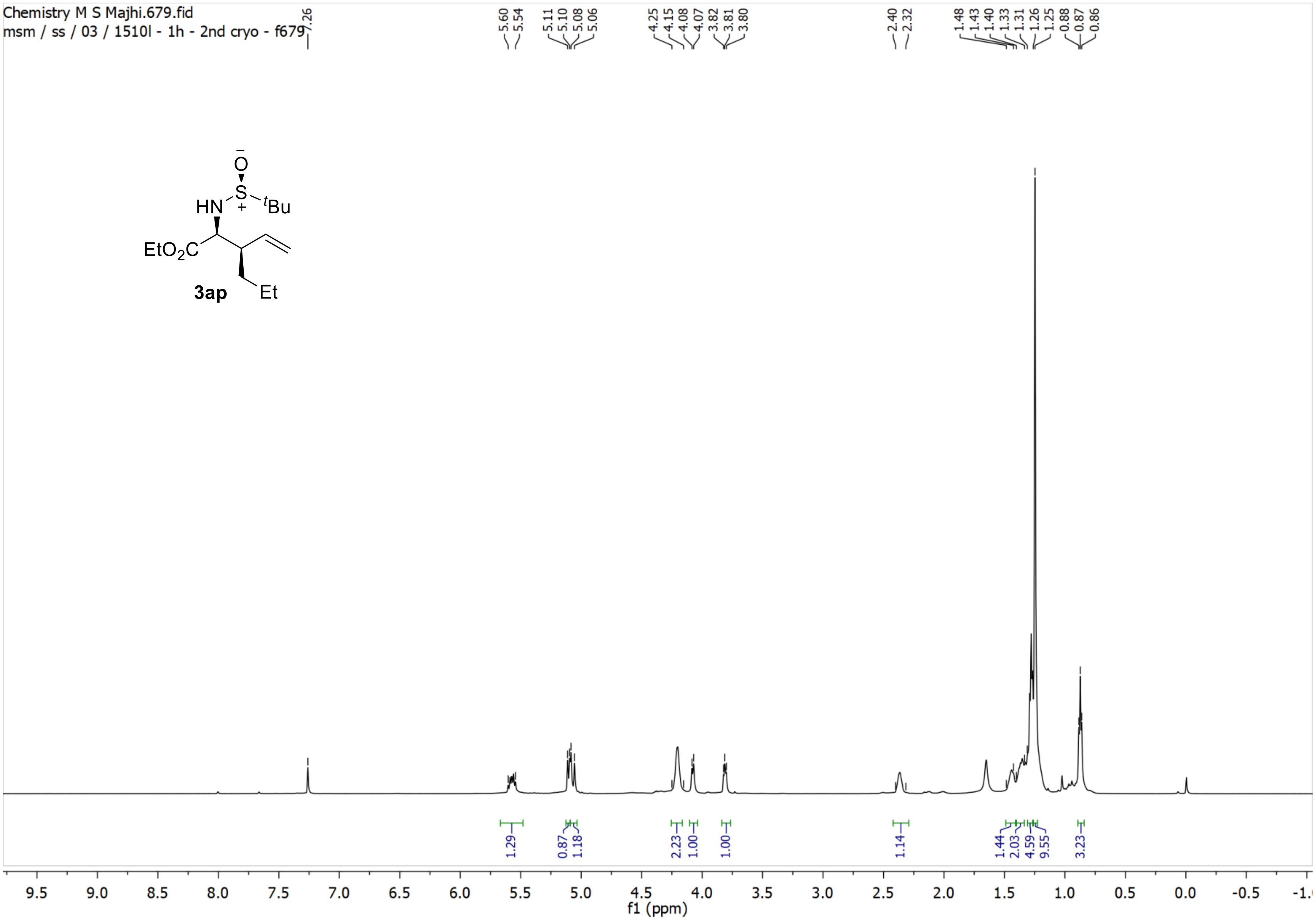
Chemistry M S Majhi.679.fid
msm / ss / 03 / 1510l - 1h - 2nd cryo - f679
3ap
EtO2C
HN
S
O
tBu
Et
f1 (ppm)

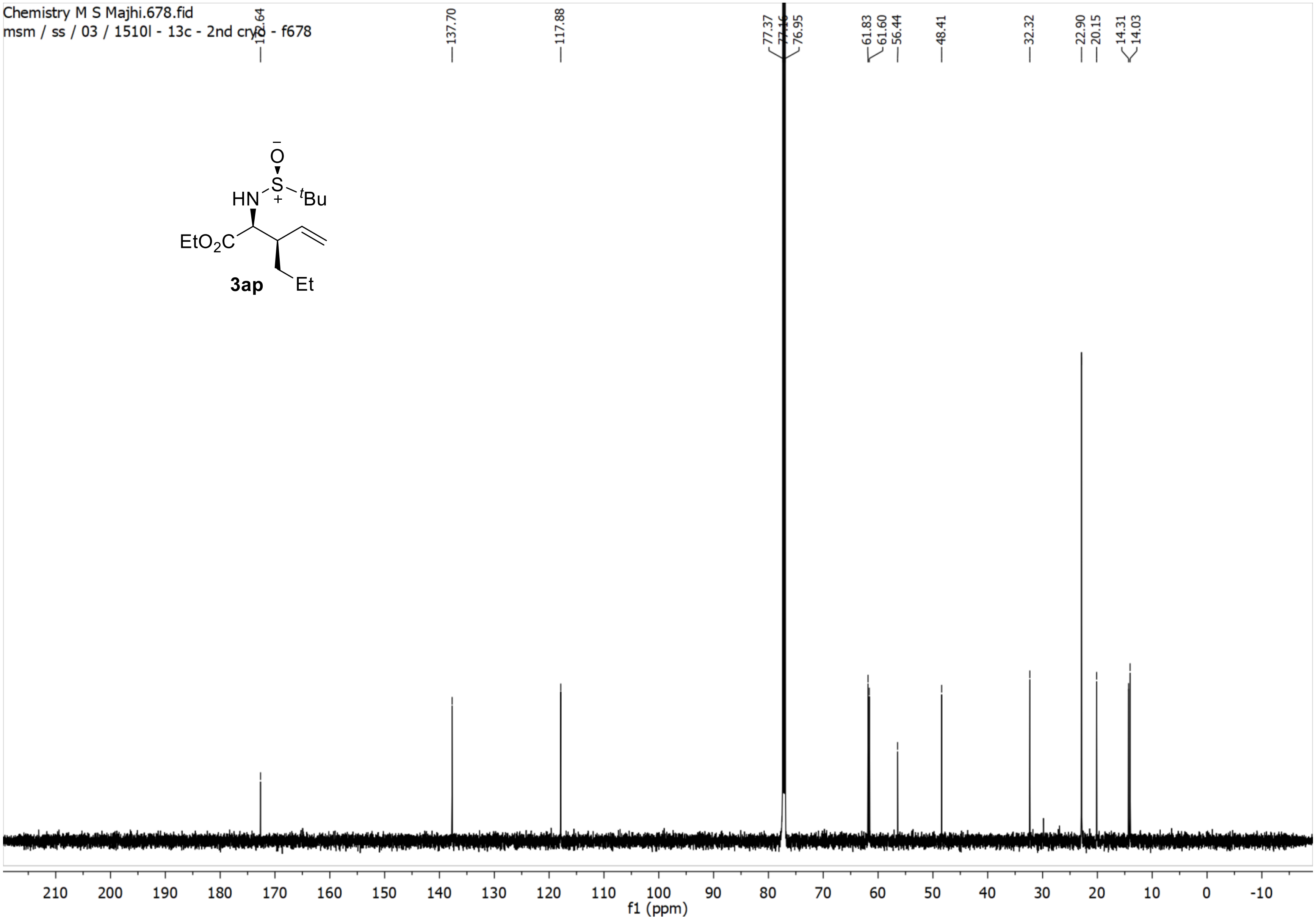

Chemistry M S Majhi.678.fid
msm / ss / 03 / 1510l - 13c - 2nd cryo - f678
172.64
137.70
117.88
77.37
77.16
76.95
61.83
61.60
56.44
48.41
32.32
22.90
20.15
14.31
14.03
HN
S
tBu
O
EtO2C
Et
3ap
210 200 190 180 170 160 150 140 130 120 110 100 90 80 70 60 50 40 30 20 10 0 -10
f1 (ppm)

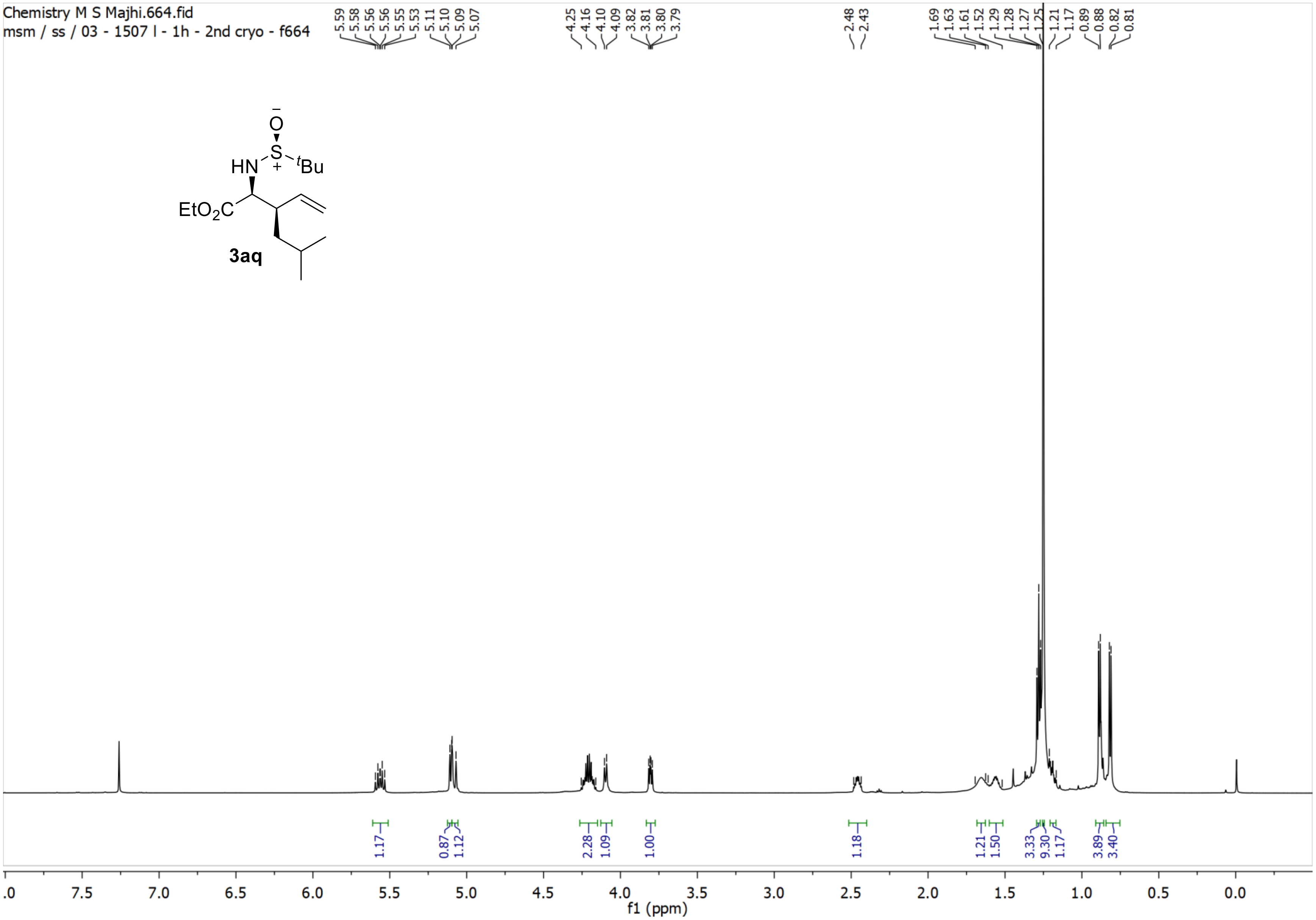
Chemistry M S Majhi.664.fid
msm / ss / 03 - 1507 l - 1h - 2nd cryo - f664
5.59 5.58 5.56 5.56 5.55 5.53 5.11 5.10 5.09 5.07
4.25 4.16 4.10 4.09 3.82 3.81 3.80 3.79
2.48 2.43
1.69 1.63 1.61 1.52 1.29 1.28 1.27 1.25 1.21 1.17 0.89 0.88 0.82 0.81
HN
O
S
tBu
EtO2C
3aq
1.17
0.87 1.12
2.28 1.09
1.00
1.18
1.21 1.50
3.33 9.30 1.17
3.89 3.40
7.5 7.0 6.5 6.0 5.5 5.0 4.5 4.0 3.5 3.0 2.5 2.0 1.5 1.0 0.5 0.0
f1 (ppm)

Chemistry M S Majhi.665.fid
msm / ss / 03 - 1507 l - 13c - 2nd crop - f665

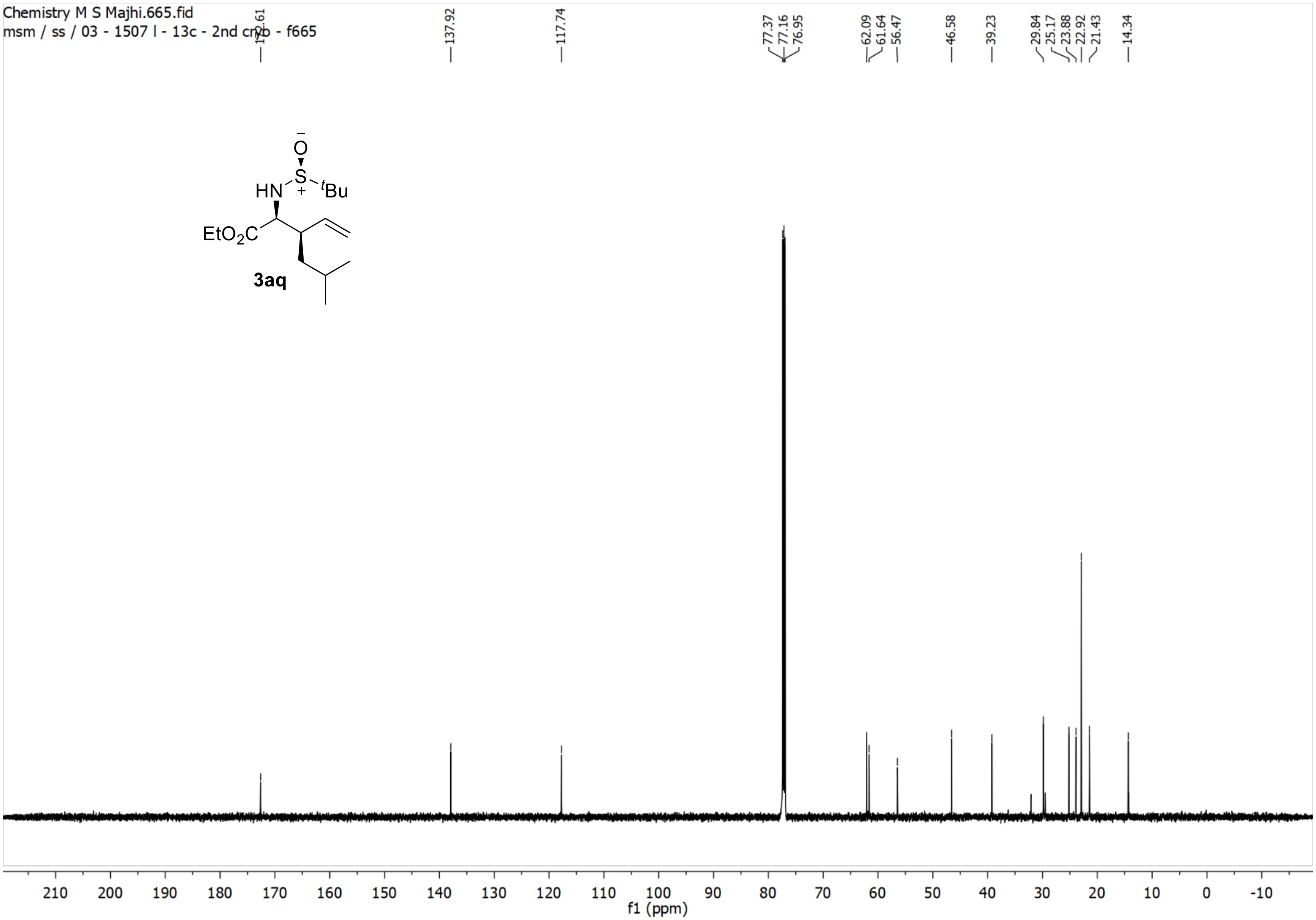

3aq

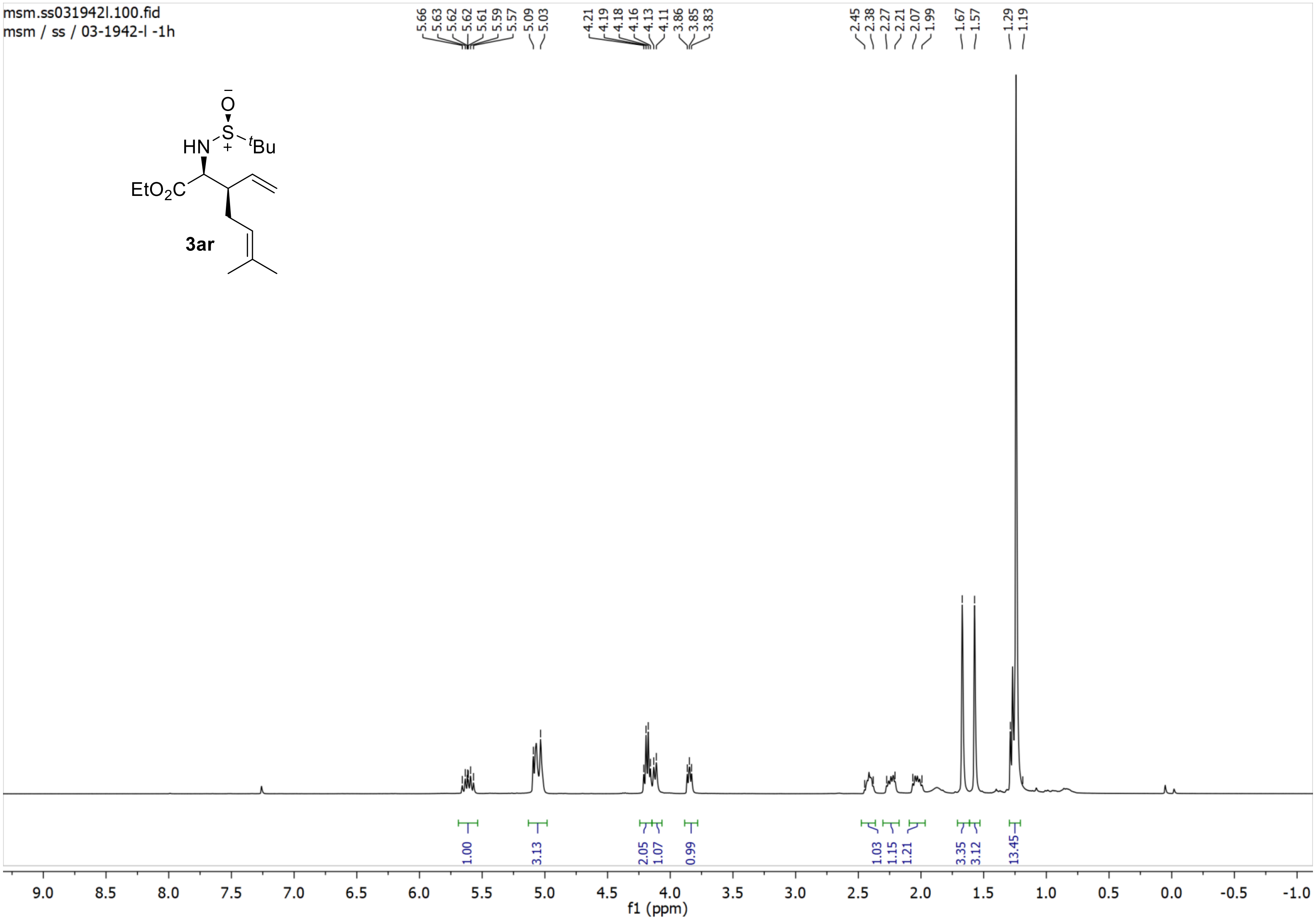
msm.ss031942l.100.fid
msm / ss / 03-1942-l -1h
3ar
f1 (ppm)

msmc.ss031942l.100.fid
msm / ss / 03-1942-l - 13c

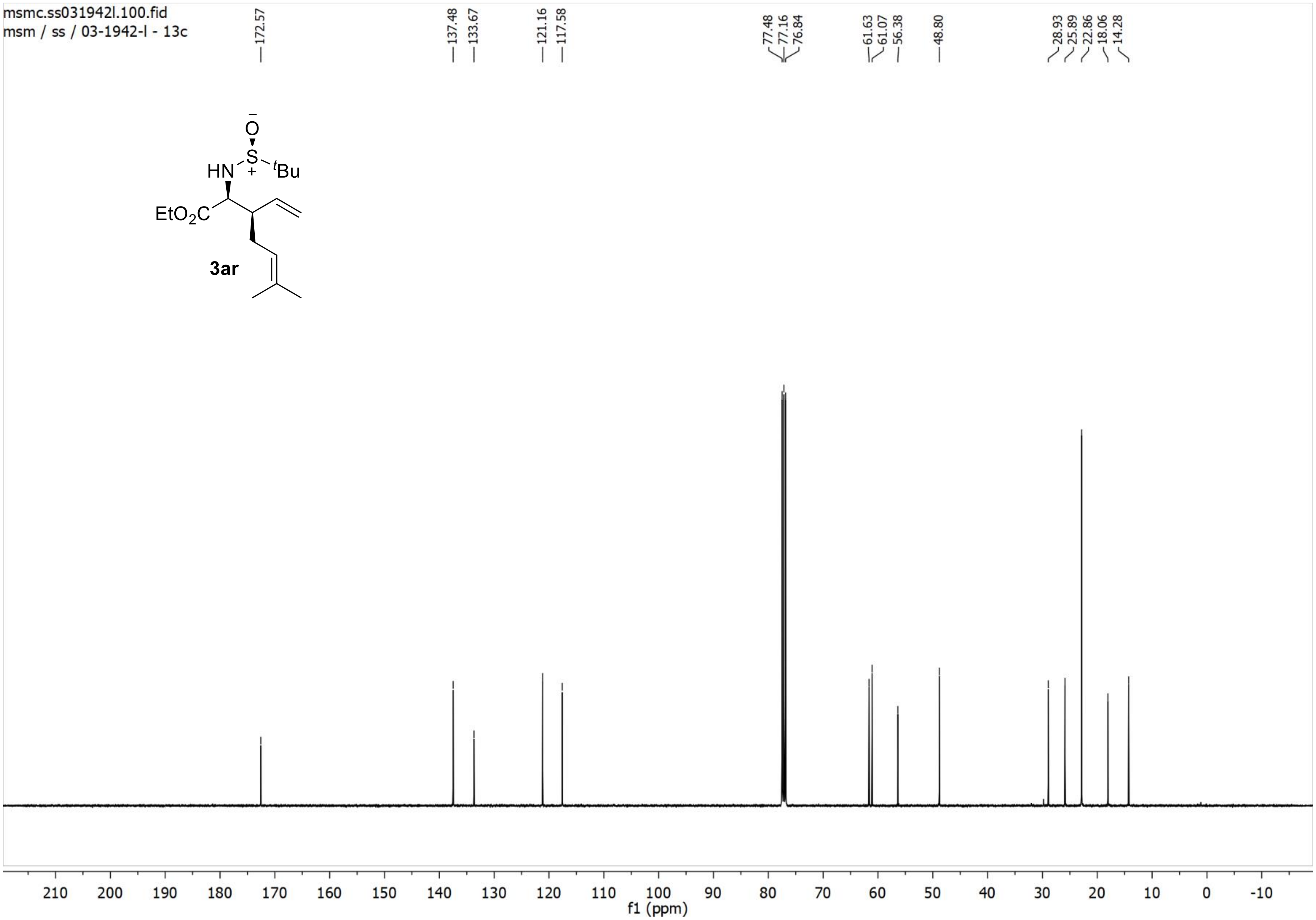

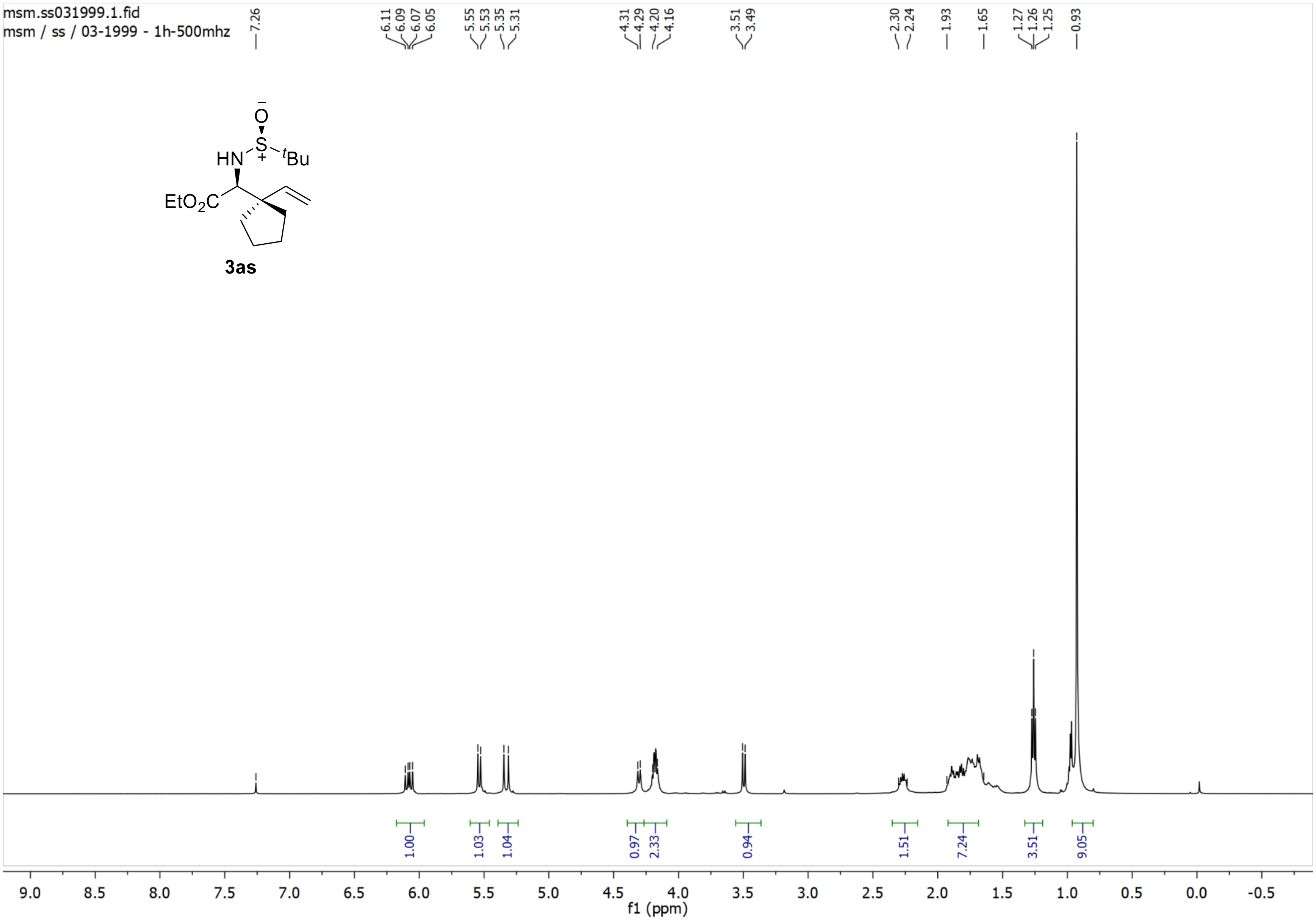

msm.ss031999.1.fid
msm / ss / 03-1999 - 1h-500mhz
7.26
6.11
6.09
6.07
6.05
5.55
5.53
5.35
5.31
4.31
4.29
4.20
4.16
3.51
3.49
2.30
2.24
1.93
1.65
1.27
1.26
1.25
0.93
HN
S
O
tBu
EtO2C
3as
1.00
1.03
1.04
0.97
2.33
0.94
1.51
7.24
3.51
9.05
f1 (ppm)

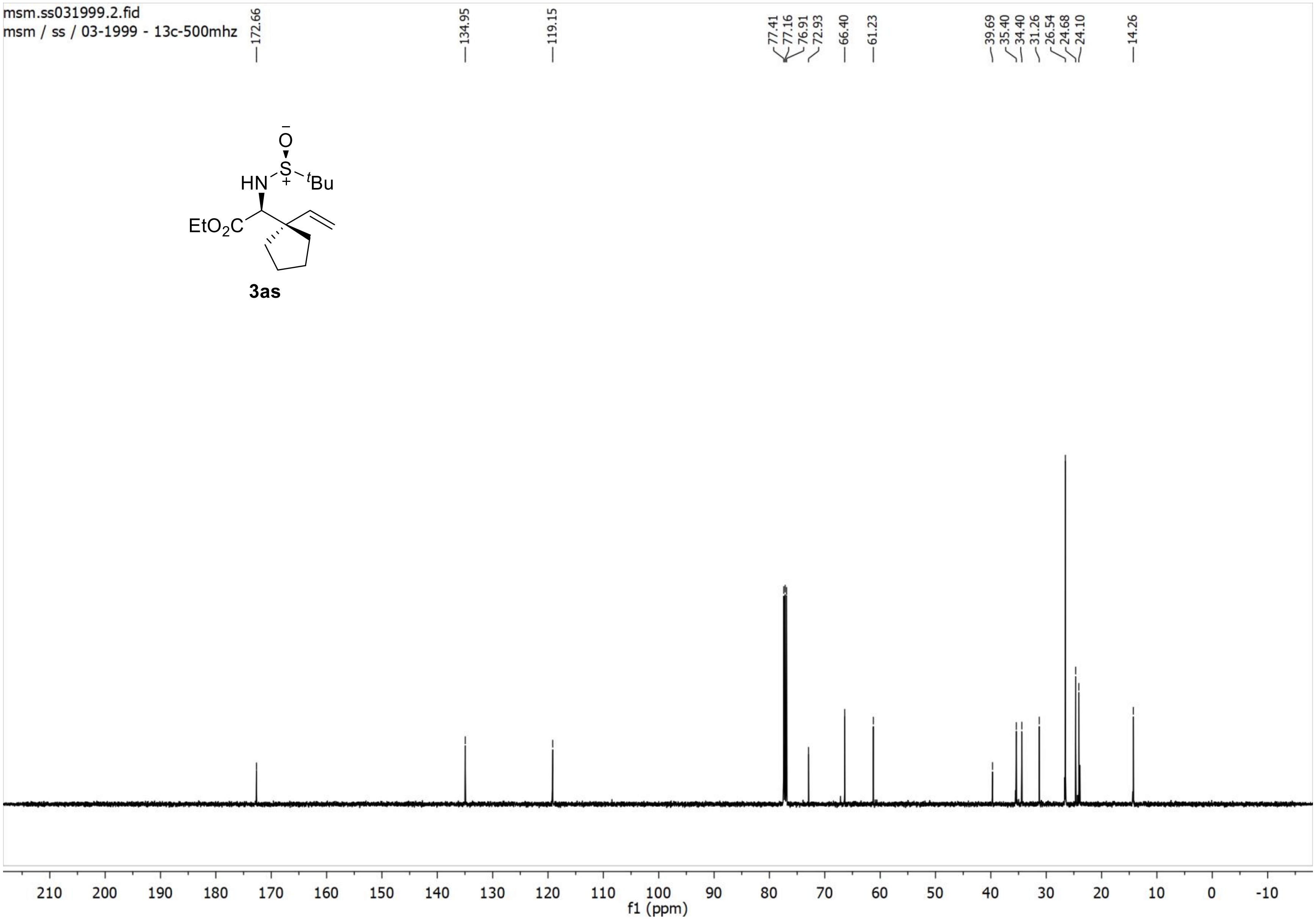

msm.ss031999.2.fid
msm / ss / 03-1999 - 13c-500mhz
172.66
134.95
119.15
77.41
77.16
76.91
72.93
66.40
61.23
39.69
35.40
34.40
31.26
26.54
24.68
24.10
14.26
f1 (ppm)

3as

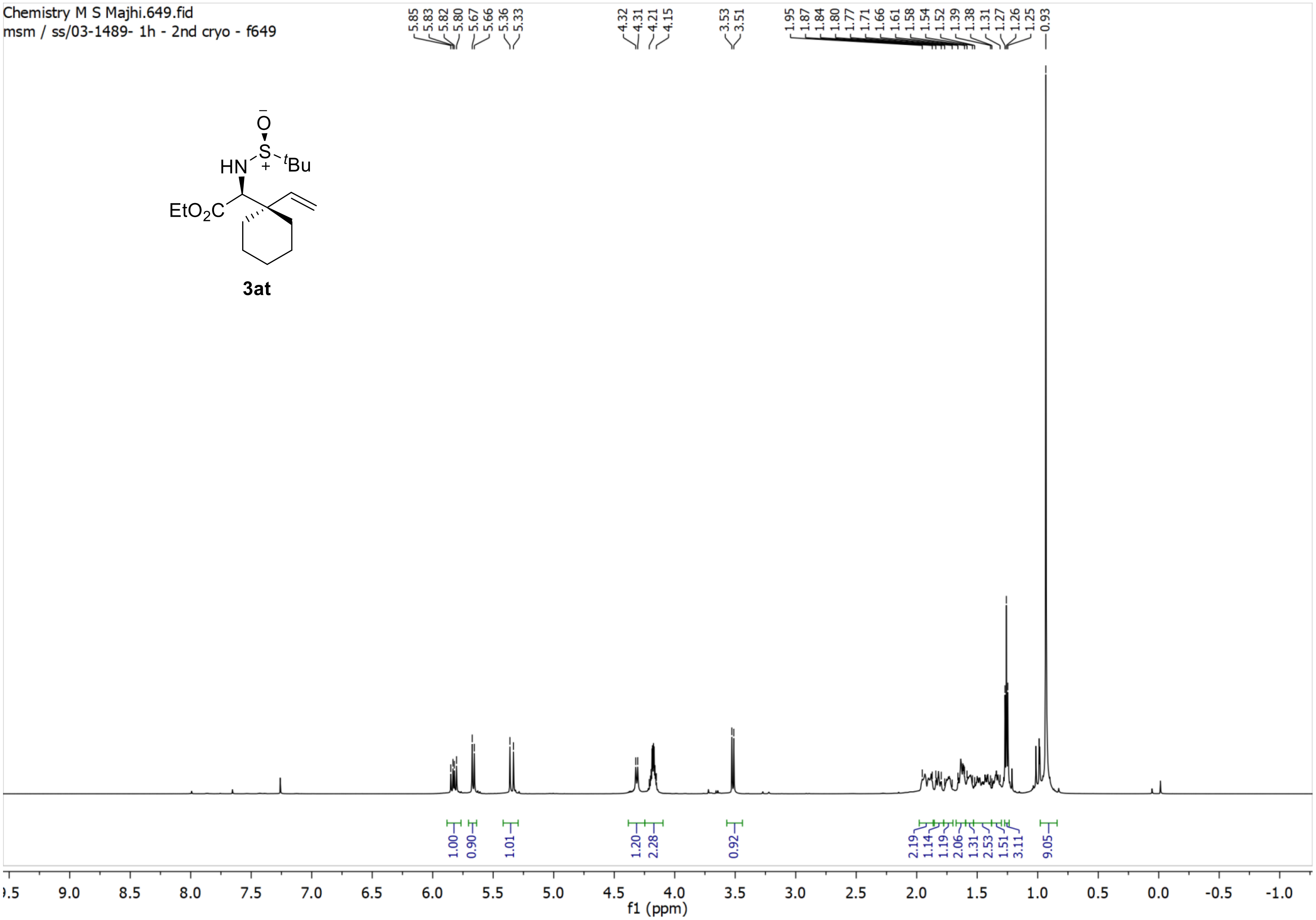

Chemistry M S Majhi.649.fid
msm / ss/03-1489- 1h - 2nd cryo - f649
HN
S
tBu
O
EtO2C
3at
5.85
5.83
5.82
5.80
5.67
5.66
5.36
5.33
4.32
4.31
4.21
4.15
3.53
3.51
1.95
1.87
1.84
1.80
1.77
1.71
1.66
1.61
1.58
1.54
1.52
1.39
1.38
1.31
1.27
1.26
1.25
0.93
1.00
0.90
1.01
1.20
2.28
0.92
2.19
1.14
1.19
2.06
1.31
2.53
1.51
3.11
9.05
f1 (ppm)

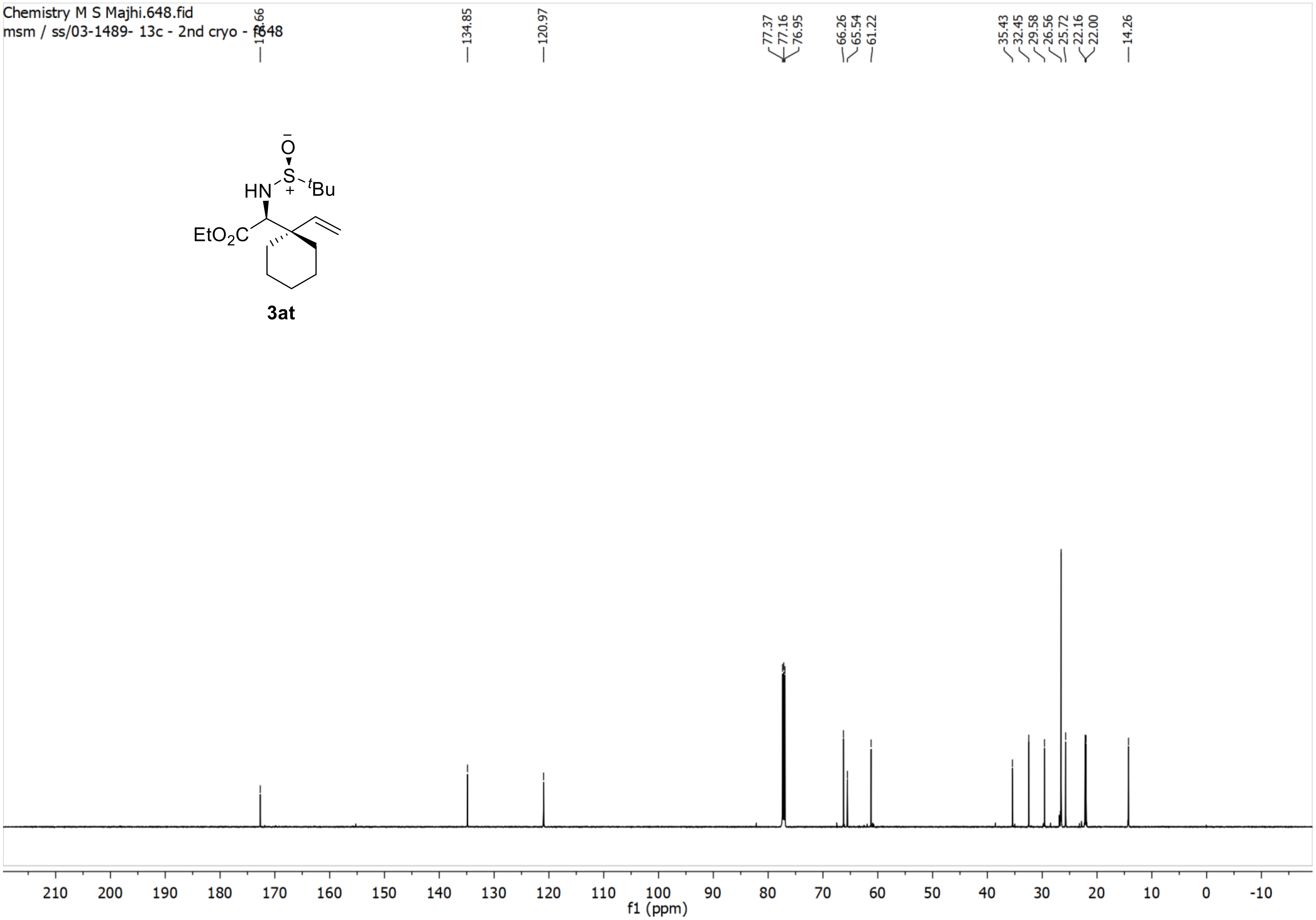

Chemistry M S Majhi.648.fid
msm / ss/03-1489- 13c - 2nd cryo - f648
134.85
120.97
77.37
77.16
76.95
66.26
65.54
61.22
35.43
32.45
29.58
26.56
25.72
22.16
22.00
14.26
HN
S
tBu
O
EtO2C
3at
210 200 190 180 170 160 150 140 130 120 110 100 90 80 70 60 50 40 30 20 10 0 -10
f1 (ppm)

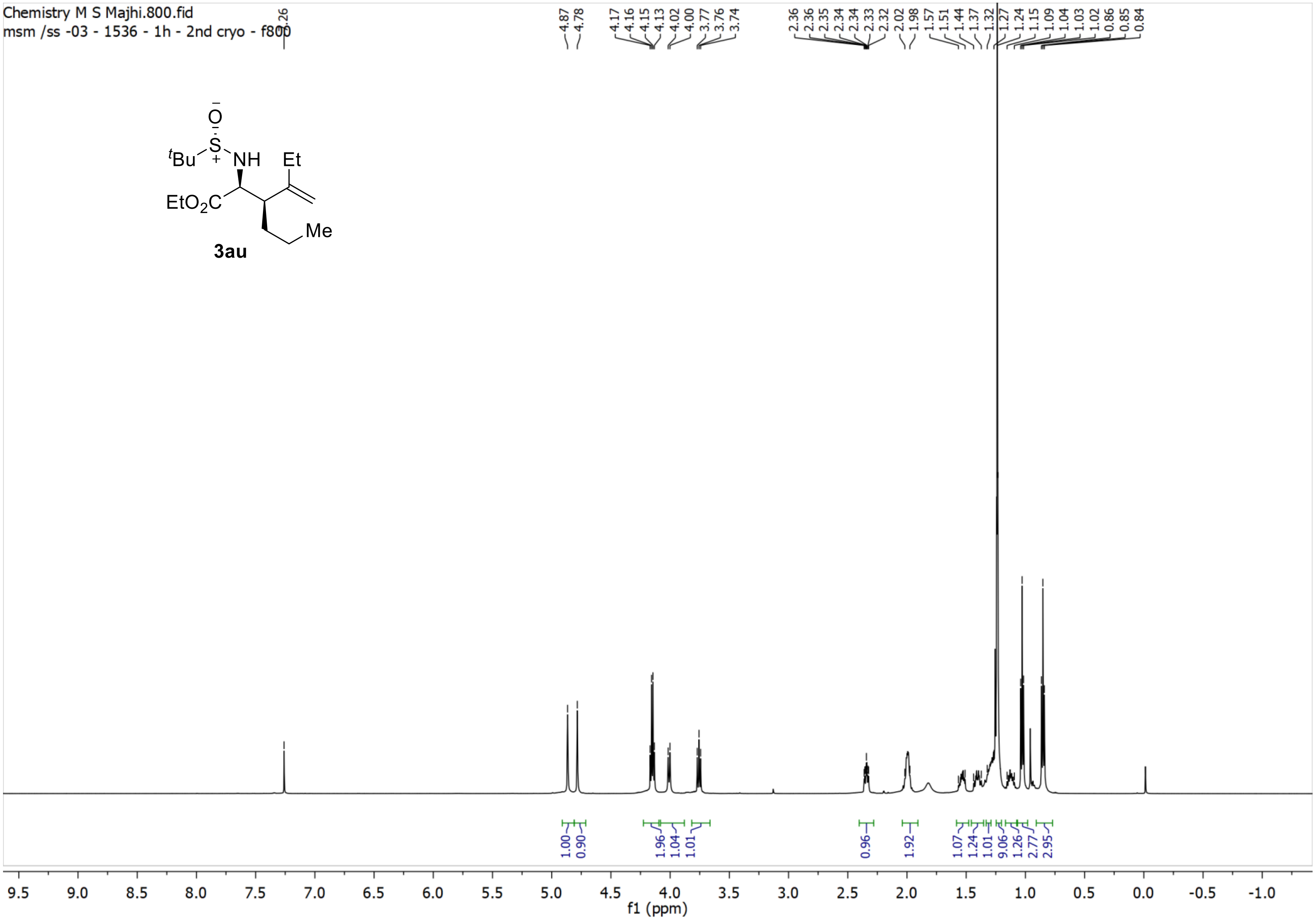
Chemistry M S Majhi.800.fid
msm /ss -03 - 1536 - 1h - 2nd cryo - f800
7.26
4.87
4.78
4.17
4.16
4.15
4.13
4.02
4.00
3.77
3.76
3.74
2.36
2.36
2.35
2.34
2.34
2.33
2.32
2.02
1.98
1.57
1.51
1.44
1.37
1.32
1.27
1.24
1.15
1.09
1.04
1.03
1.02
0.86
0.85
0.84
O
tBu
S
NH
Et
EtO2C
Me
3au
1.00
0.90
1.96
1.04
1.01
0.96
1.92
1.07
1.24
1.01
9.06
1.26
2.77
2.95
9.5 9.0 8.5 8.0 7.5 7.0 6.5 6.0 5.5 5.0 4.5 4.0 3.5 3.0 2.5 2.0 1.5 1.0 0.5 0.0 -0.5 -1.0
f1 (ppm)

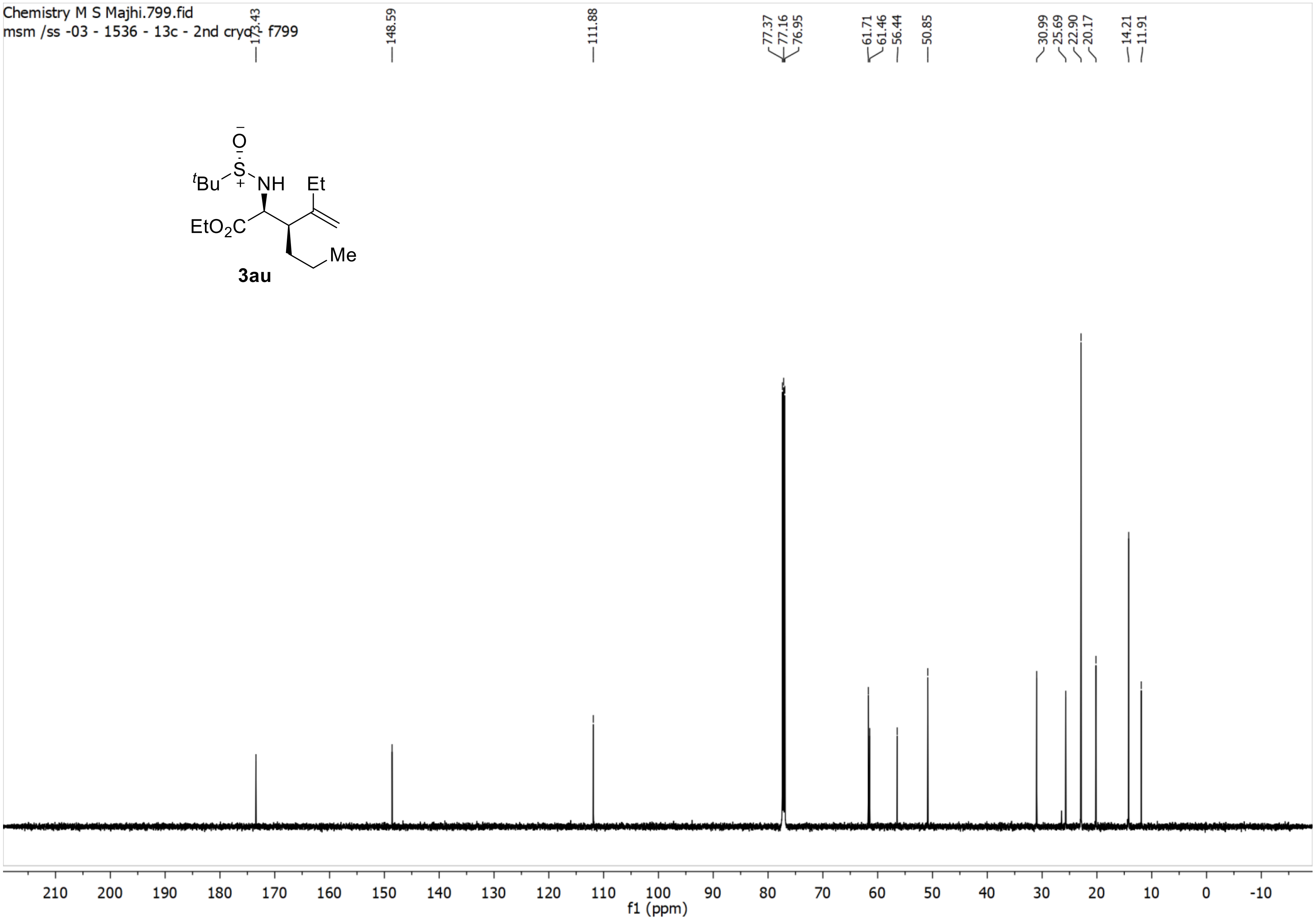
Chemistry M S Majhi.799.fid
msm /ss -03 - 1536 - 13c - 2nd cryo - f799
173.43
148.59
111.88
77.37
77.16
76.95
61.71
61.46
56.44
50.85
30.99
25.69
22.90
20.17
14.21
11.91
O
S
tBu
NH
Et
EtO2C
Me
3au
210
200
190
180
170
160
150
140
130
120
110
100
90
80
70
60
50
40
30
20
10
0
-10
f1 (ppm)

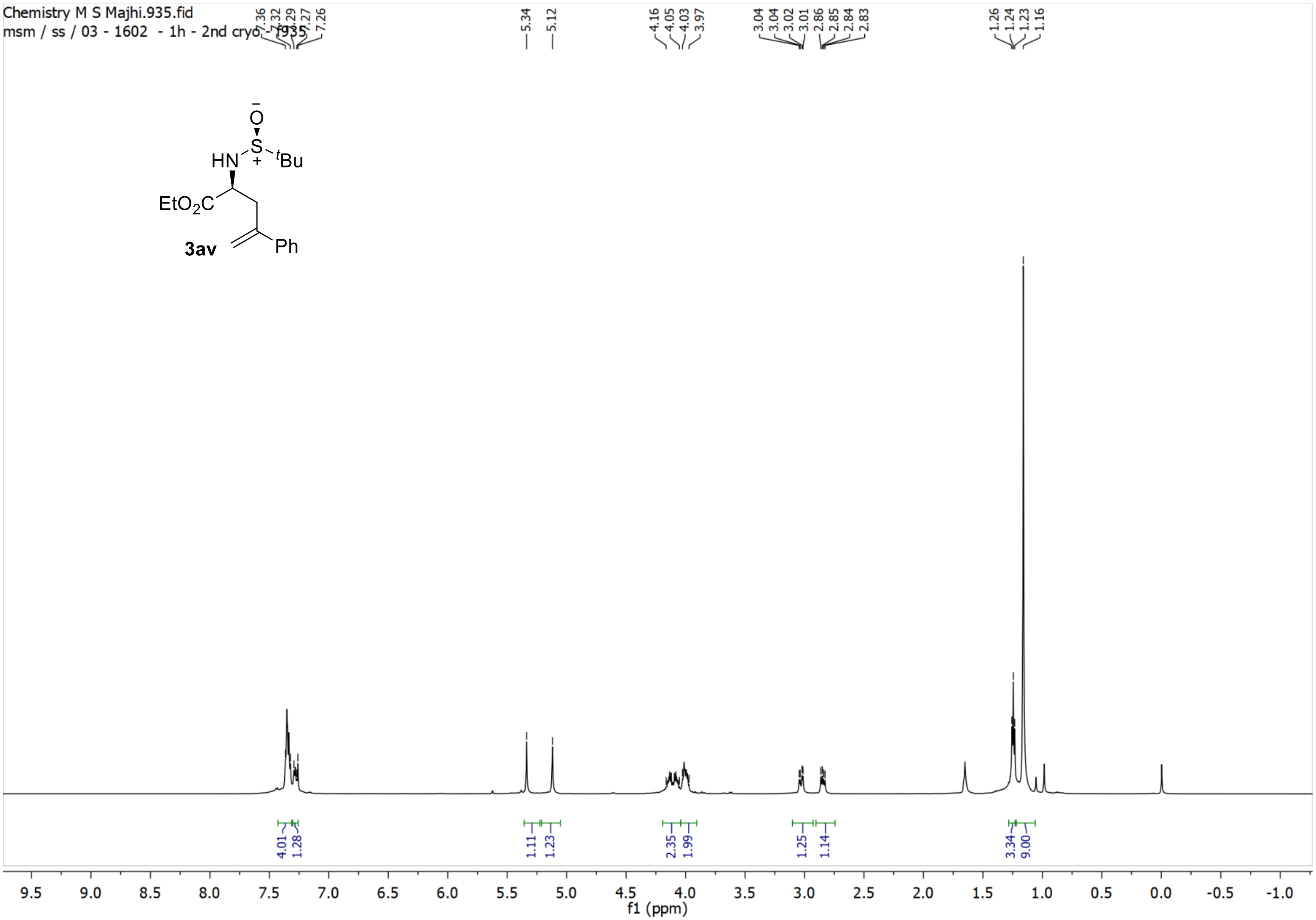

Chemistry M S Majhi.935.fid
msm / ss / 03 - 1602 - 1h - 2nd cryo - f935
7.36
7.32
7.29
7.27
7.26
5.34
5.12
4.16
4.05
4.03
3.97
3.04
3.04
3.02
3.01
2.86
2.85
2.84
2.83
1.26
1.24
1.23
1.16
HN
S
O
tBu
EtO2C
Ph
3av
4.01
1.28
1.11
1.23
2.35
1.99
1.25
1.14
3.34
9.00
9.5
9.0
8.5
8.0
7.5
7.0
6.5
6.0
5.5
5.0
4.5
4.0
3.5
3.0
2.5
2.0
1.5
1.0
0.5
0.0
-0.5
-1.0
f1 (ppm)

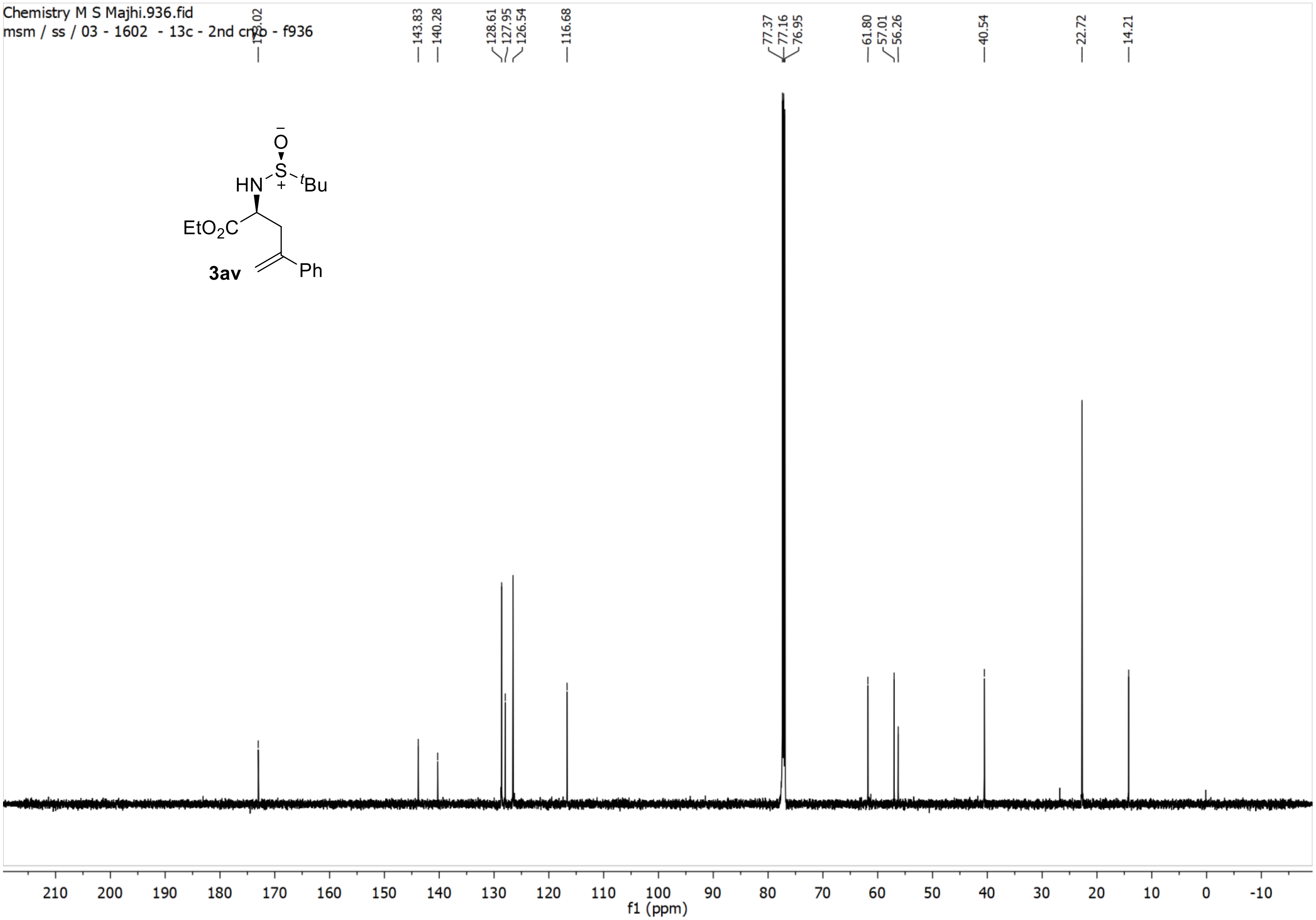
Chemistry M S Majhi.936.fid
msm / ss / 03 - 1602 - 13c - 2nd cryo - f936
173.02
143.83
140.28
128.61
127.95
126.54
116.68
77.37
77.16
76.95
61.80
57.01
56.26
40.54
22.72
14.21
HN
S
O
tBu
EtO2C
Ph
3av
210 200 190 180 170 160 150 140 130 120 110 100 90 80 70 60 50 40 30 20 10 0 -10
f1 (ppm)

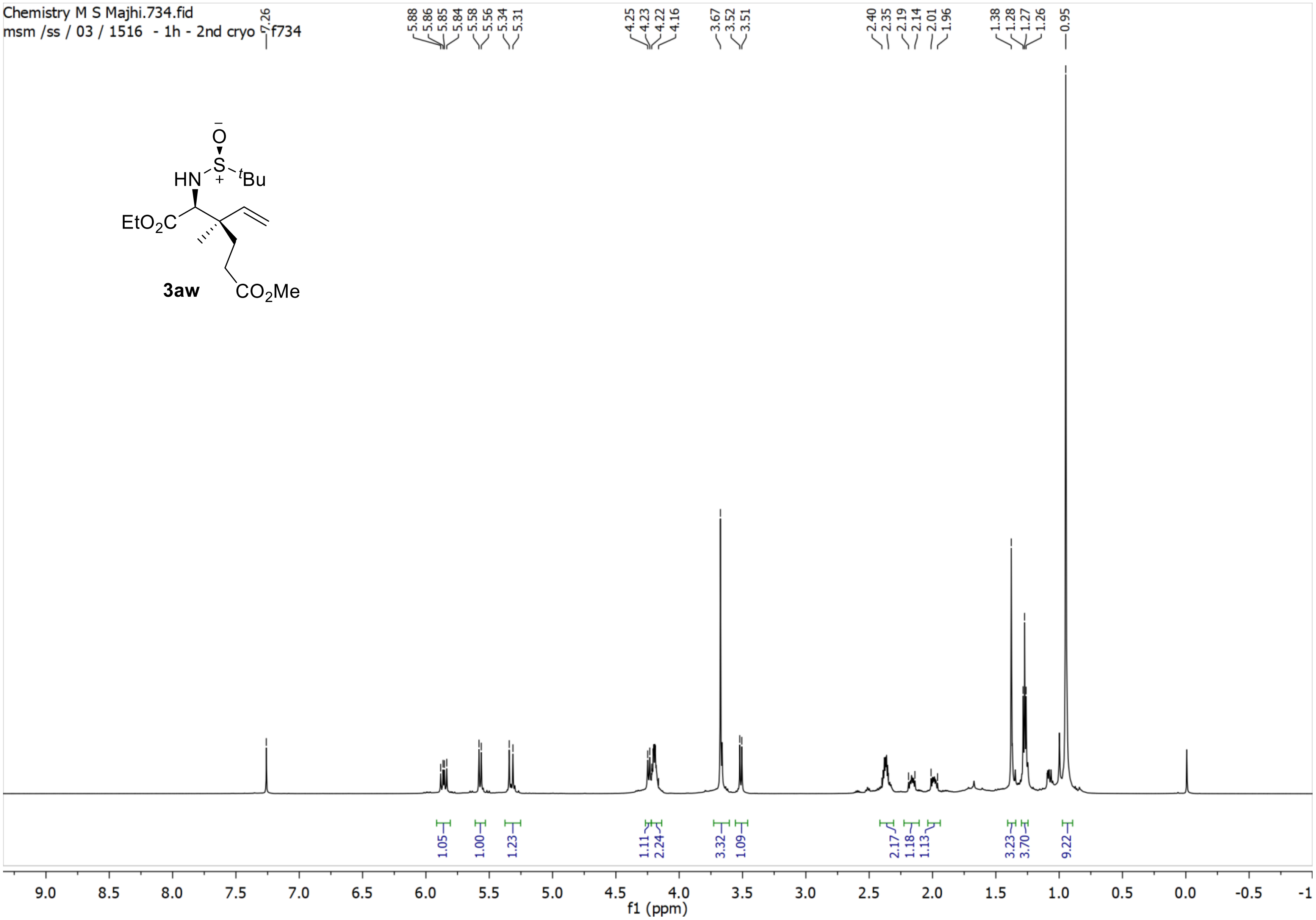

Chemistry M S Majhi.734.fid
msm /ss / 03 / 1516 - 1h - 2nd cryo - f734
7.26
5.88
5.86
5.85
5.84
5.58
5.56
5.34
5.31
4.25
4.23
4.22
4.16
3.67
3.52
3.51
2.40
2.35
2.19
2.14
2.01
1.96
1.38
1.28
1.27
1.26
0.95
3aw
1.05
1.00
1.23
1.11
2.24
3.32
1.09
2.17
1.18
1.13
3.23
3.70
9.22
9.0 8.5 8.0 7.5 7.0 6.5 6.0 5.5 5.0 4.5 4.0 3.5 3.0 2.5 2.0 1.5 1.0 0.5 0.0 -0.5 -1
f1 (ppm)

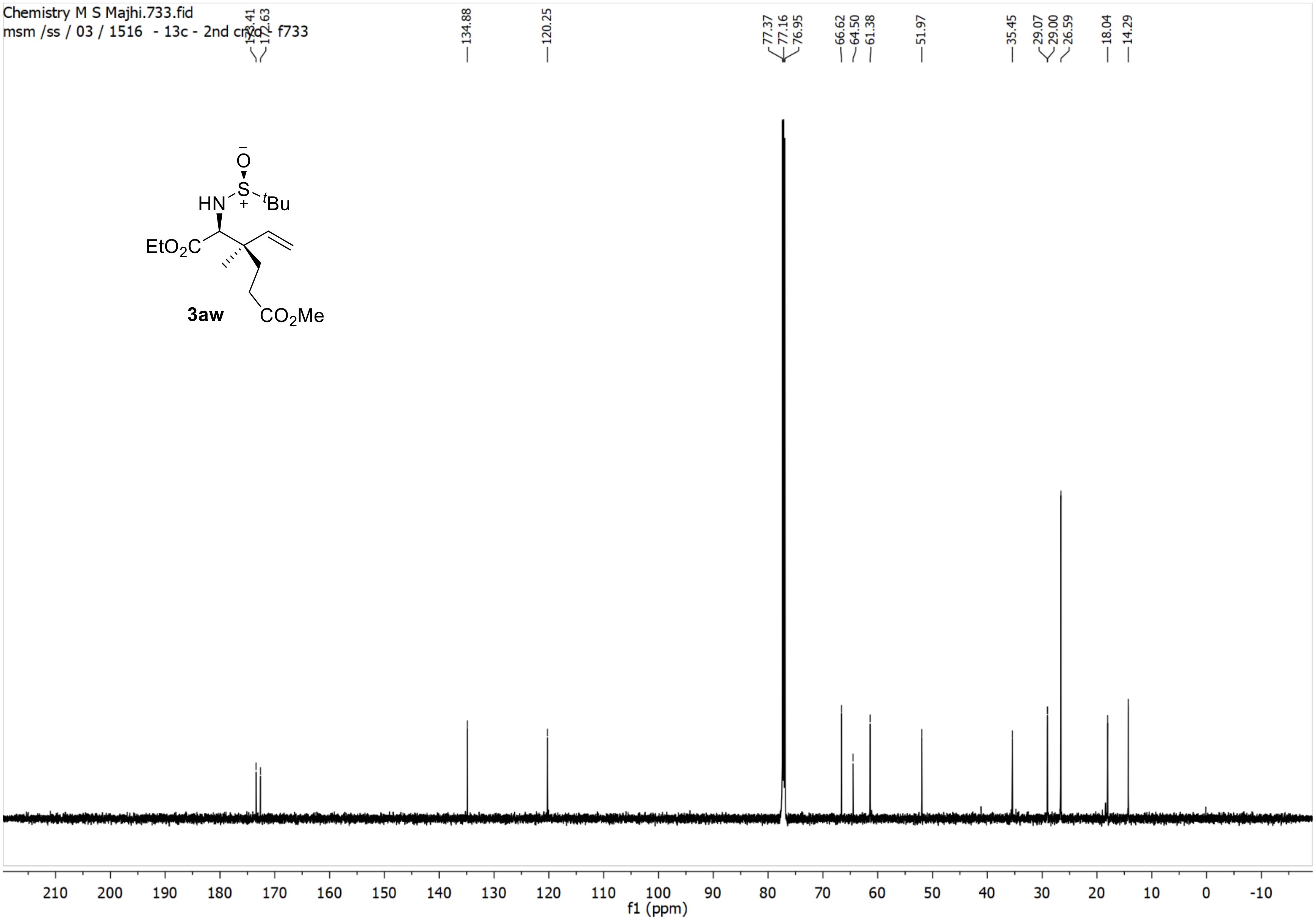
Chemistry M S Majhi.733.fid
msm /ss / 03 / 1516 - 13c - 2nd crydl - f733
173.41
172.63
134.88
120.25
77.37
77.16
76.95
66.62
64.50
61.38
51.97
35.45
29.07
29.00
26.59
18.04
14.29
HN
S
O
tBu
EtO2C
CO2Me
3aw
f1 (ppm)

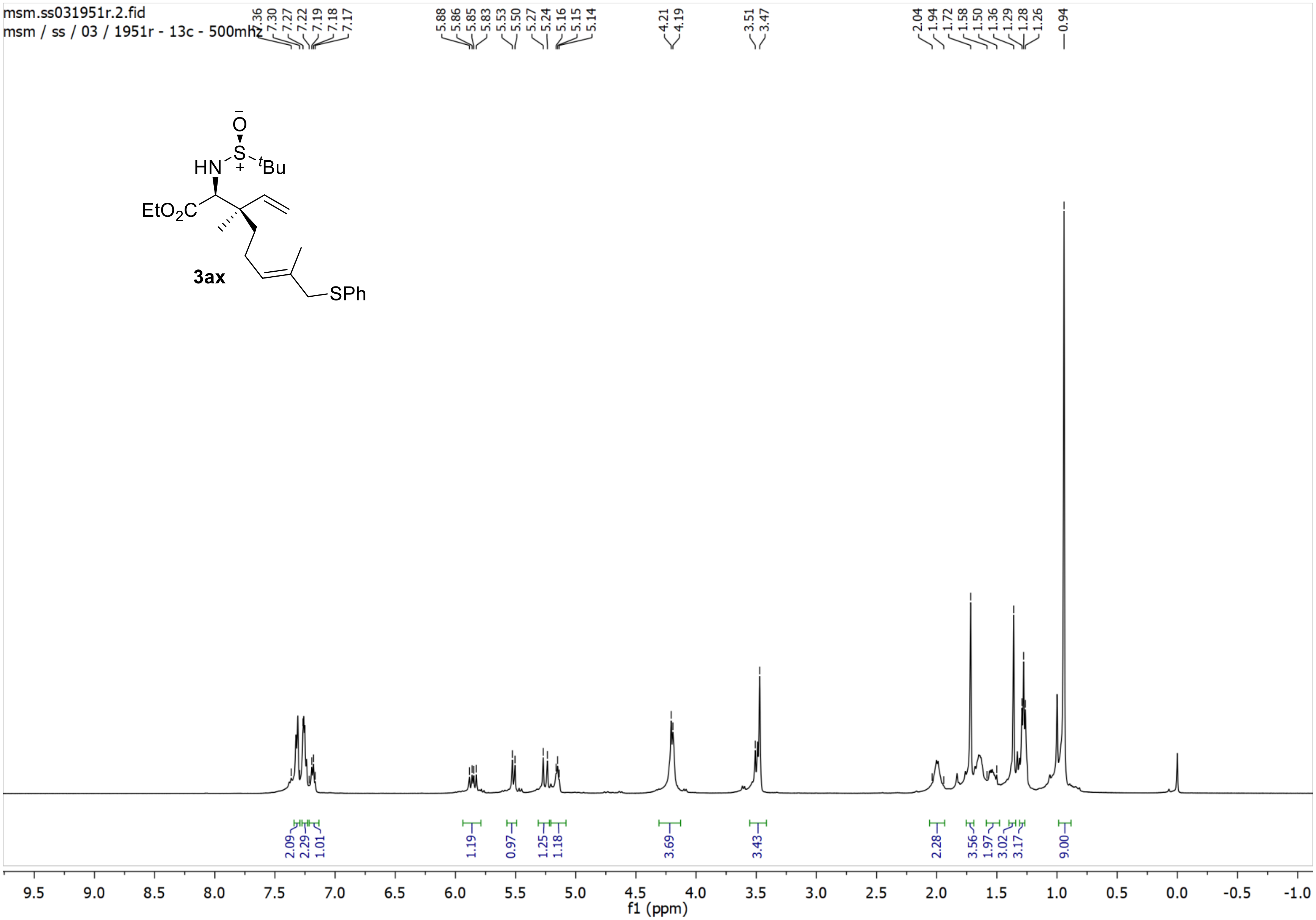

msm.ss031951r.2.fid
msm / ss / 03 / 1951r - 13c - 500mhz
7.36 7.30 7.27 7.22 7.19 7.18 7.17
5.88 5.86 5.85 5.83 5.53 5.50 5.27 5.24 5.16 5.15 5.14
4.21 4.19
3.51 3.47
2.04 1.94 1.72 1.58 1.50 1.36 1.29 1.28 1.26
0.94
HN
S
O
tBu
EtO2C
SPh
3ax
2.09 2.29 1.01
1.19
0.97
1.25 1.18
3.69
3.43
2.28
3.56 1.97 3.02 3.17
9.00
9.5 9.0 8.5 8.0 7.5 7.0 6.5 6.0 5.5 5.0 4.5 4.0 3.5 3.0 2.5 2.0 1.5 1.0 0.5 0.0 -0.5 -1.0
f1 (ppm)

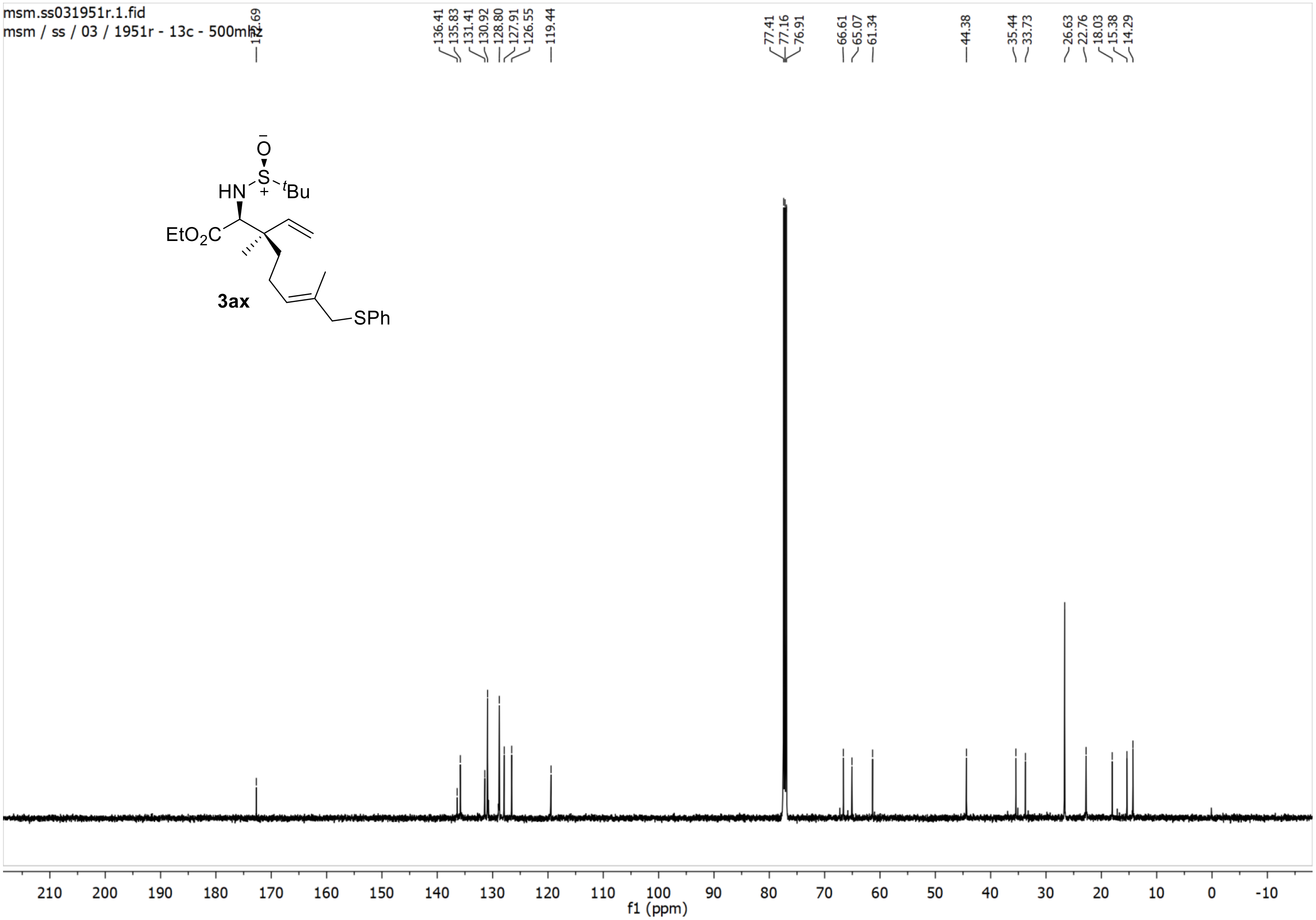
msm.ss031951r.1.fid
msm / ss / 03 / 1951r - 13c - 500mhz
136.41
135.83
131.41
130.92
128.80
127.91
126.55
119.44
77.41
77.16
76.91
66.61
65.07
61.34
44.38
35.44
33.73
26.63
22.76
18.03
15.38
14.29
HN
S
tBu
EtO2C
3ax
SPh
f1 (ppm)

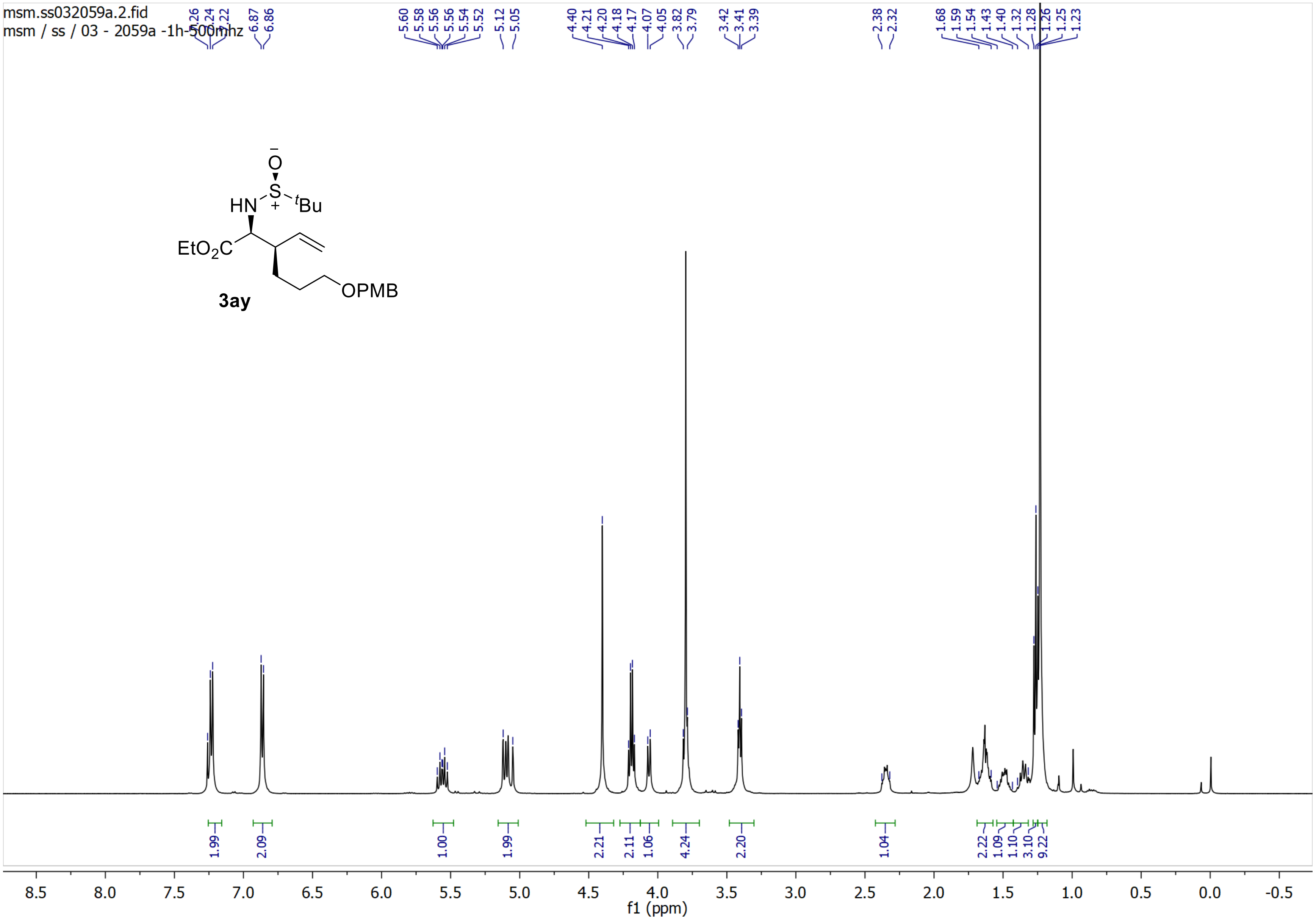

msm.ss032059a.2.fid
msm / ss / 03 - 2059a -1h-500mhz
HN
tBu
EtO2C
OPMB
3ay
f1 (ppm)

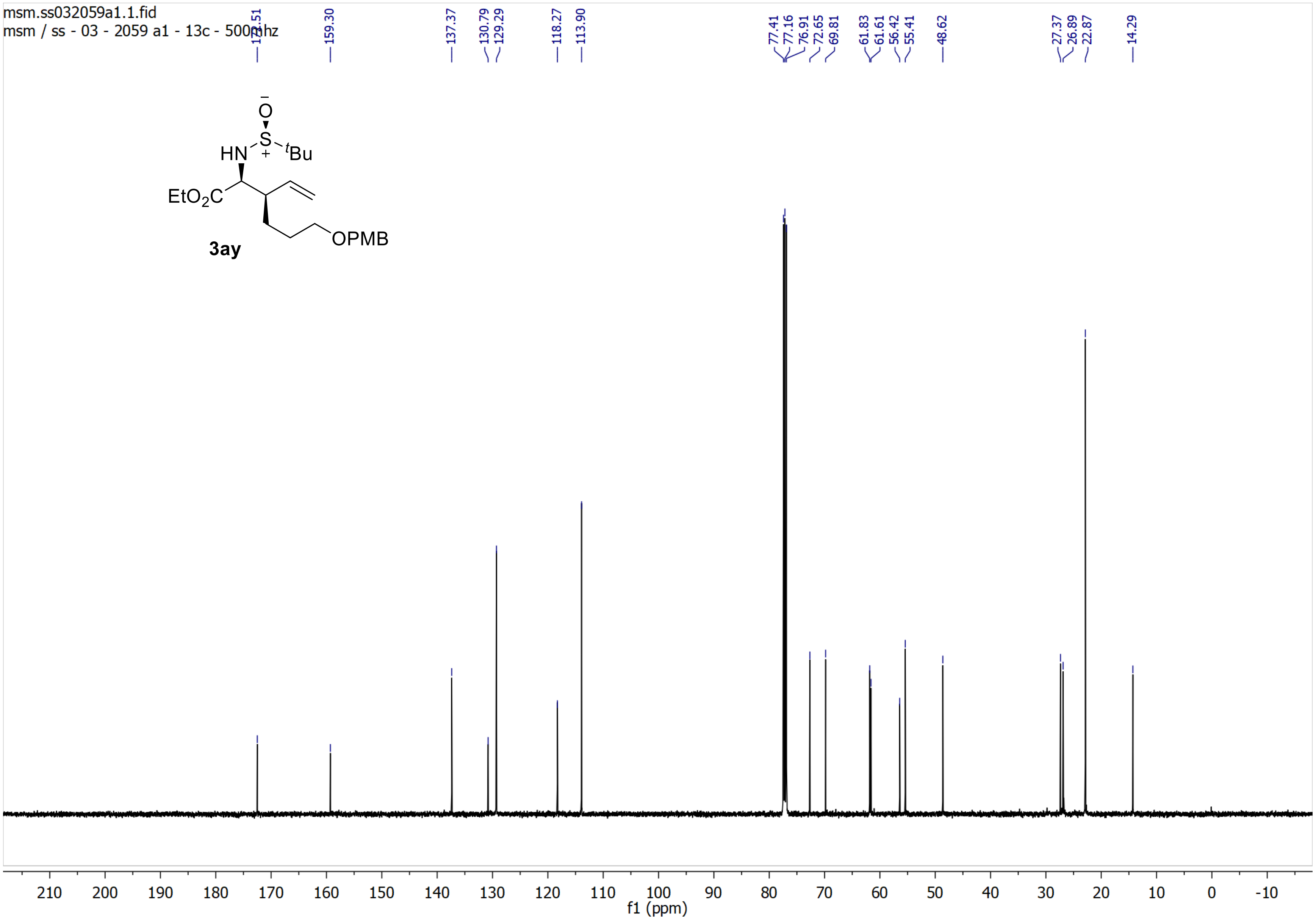

msm.ss032059a1.1.fid
msm / ss - 03 - 2059 a1 - 13c - 500mhz
159.30
137.37
130.79
129.29
118.27
113.90
77.41
77.16
76.91
72.65
69.81
61.83
61.61
56.42
55.41
48.62
27.37
26.89
22.87
14.29
HN
tBu
EtO2C
OPMB
3ay
f1 (ppm)

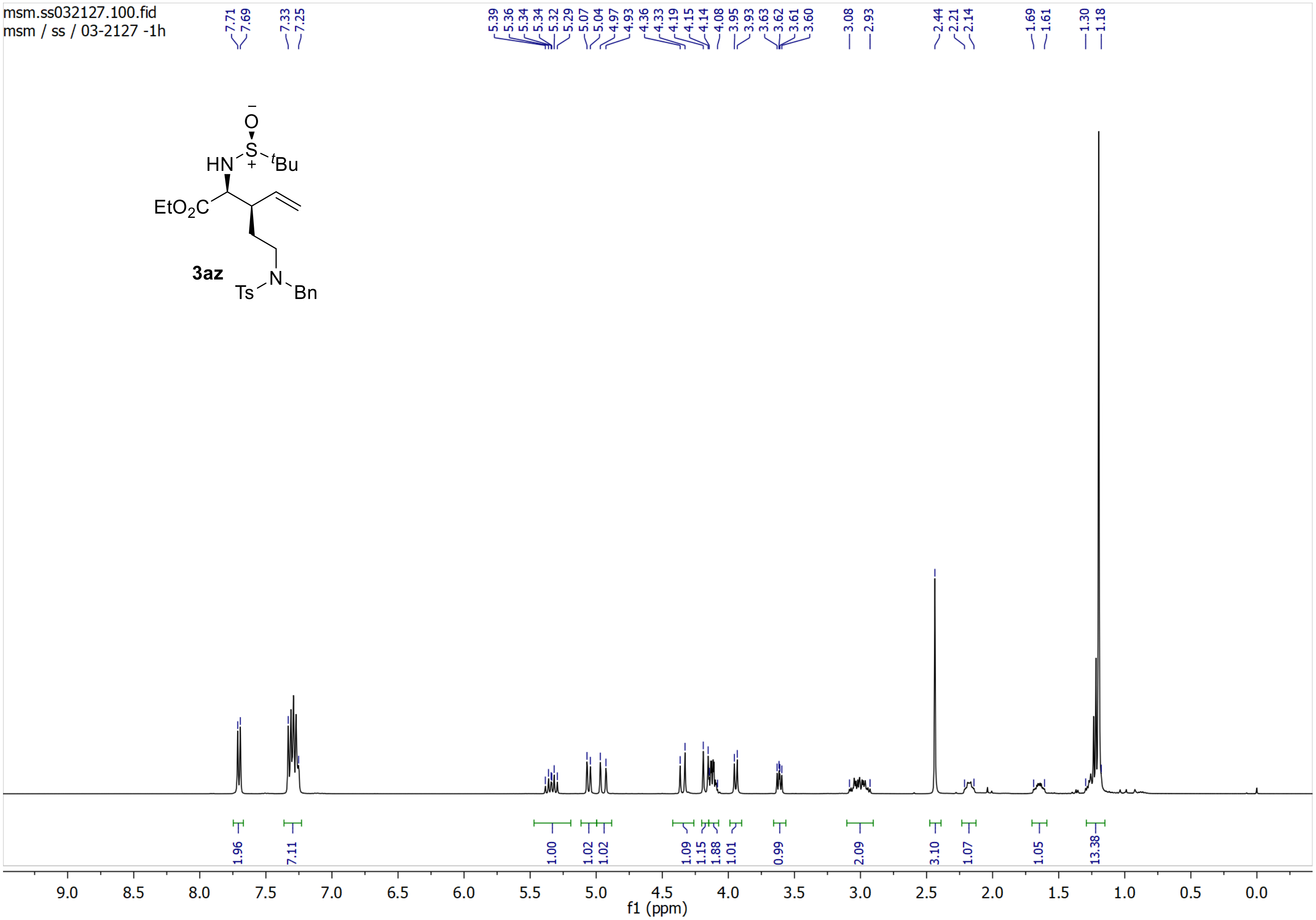
msm.ss032127.100.fid
msm / ss / 03-2127 -1h
7.71
7.69
7.33
7.25
5.39
5.36
5.34
5.34
5.32
5.29
5.07
5.04
4.97
4.93
4.36
4.33
4.19
4.15
4.14
4.08
3.95
3.93
3.63
3.62
3.61
3.60
3.08
2.93
2.44
2.21
2.14
1.69
1.61
1.30
1.18
3az
1.96
7.11
1.00
1.02
1.02
1.09
1.15
1.88
1.01
0.99
2.09
3.10
1.07
1.05
13.38
f1 (ppm)

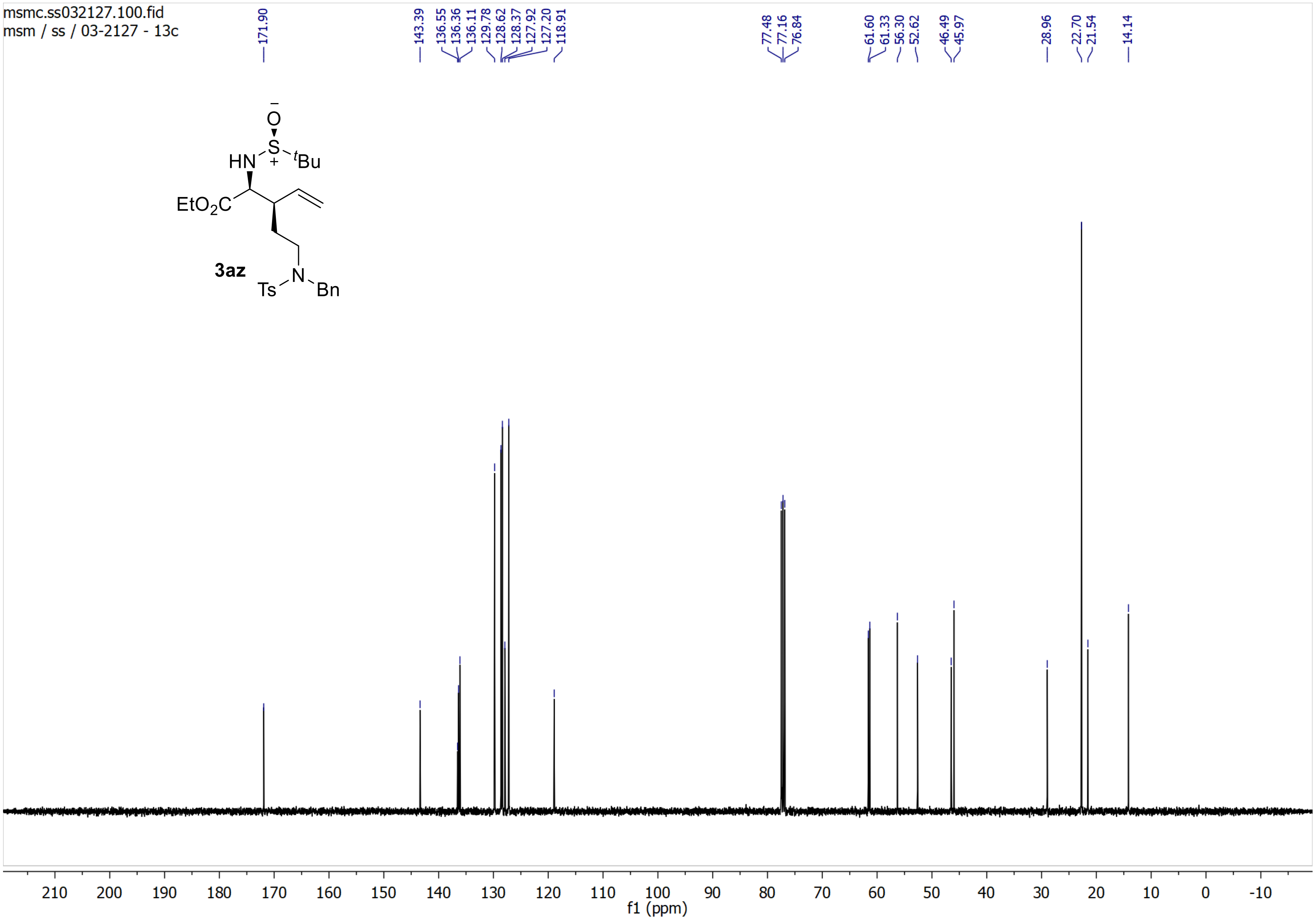
msmc.ss032127.100.fid
msm / ss / 03-2127 - 13c
171.90
143.39
136.55
136.36
136.11
129.78
128.62
128.37
127.92
127.20
118.91
77.48
77.16
76.84
61.60
61.33
56.30
52.62
46.49
45.97
28.96
22.70
21.54
14.14
HN
S
O
tBu
EtO2C
3az
Ts
N
Bn
210 200 190 180 170 160 150 140 130 120 110 100 90 80 70 60 50 40 30 20 10 0 -10
f1 (ppm)

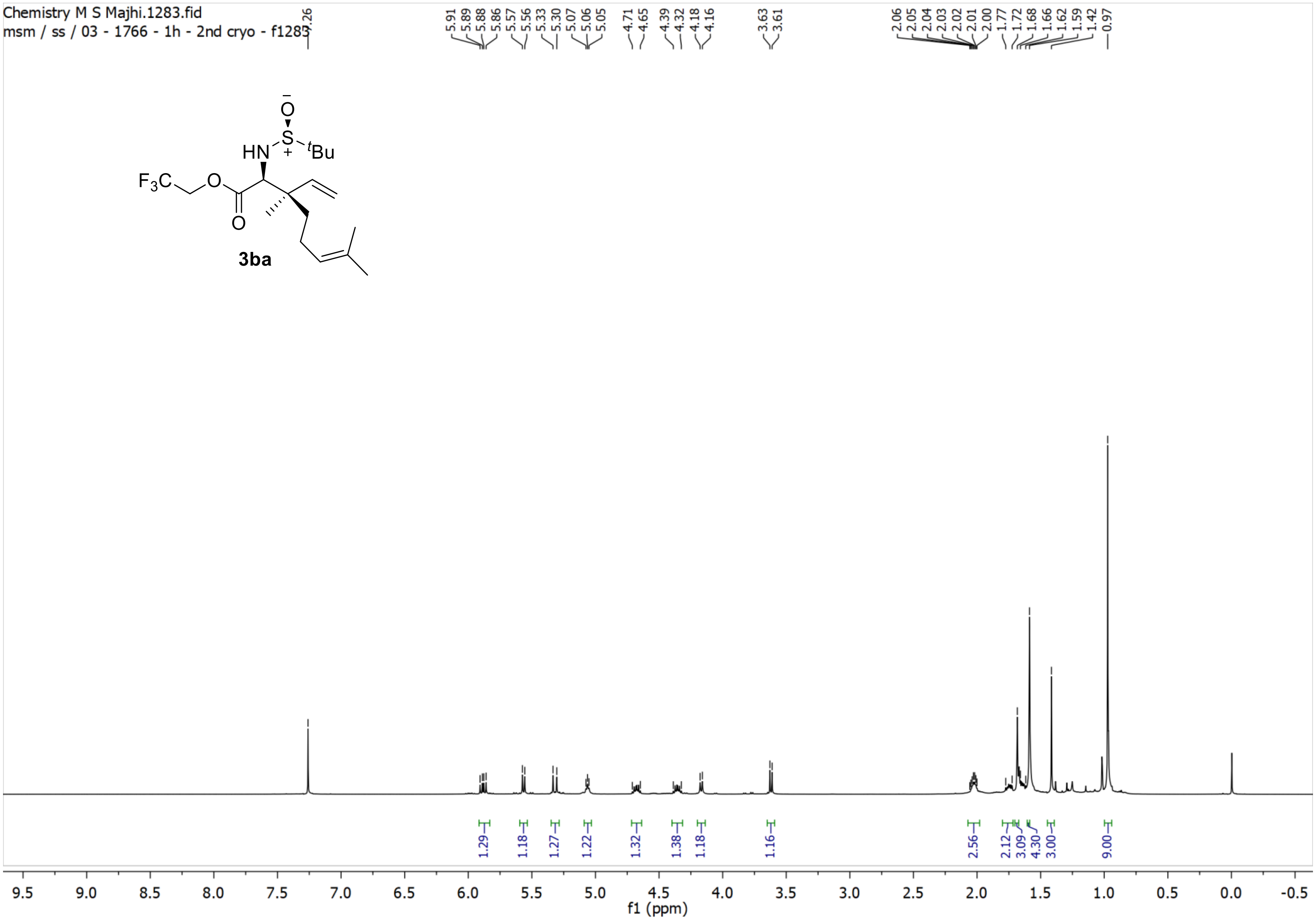

Chemistry M S Majhi.1283.fid
msm / ss / 03 - 1766 - 1h - 2nd cryo - f1283
7.26
5.91 5.89 5.88 5.86 5.57 5.56 5.33 5.30 5.07 5.06 5.05 4.71 4.65 4.39 4.32 4.18 4.16 3.63 3.61
2.06 2.05 2.04 2.03 2.02 2.01 2.00 1.77 1.72 1.68 1.66 1.62 1.59 1.42 0.97
HN
O
S
+
tBu
F3C
O
O
3ba
1.29 1.18 1.27 1.22 1.32 1.38 1.18 1.16 2.56 2.12 3.09 4.30 3.00 9.00
9.5 9.0 8.5 8.0 7.5 7.0 6.5 6.0 5.5 5.0 4.5 4.0 3.5 3.0 2.5 2.0 1.5 1.0 0.5 0.0 -0.5
f1 (ppm)

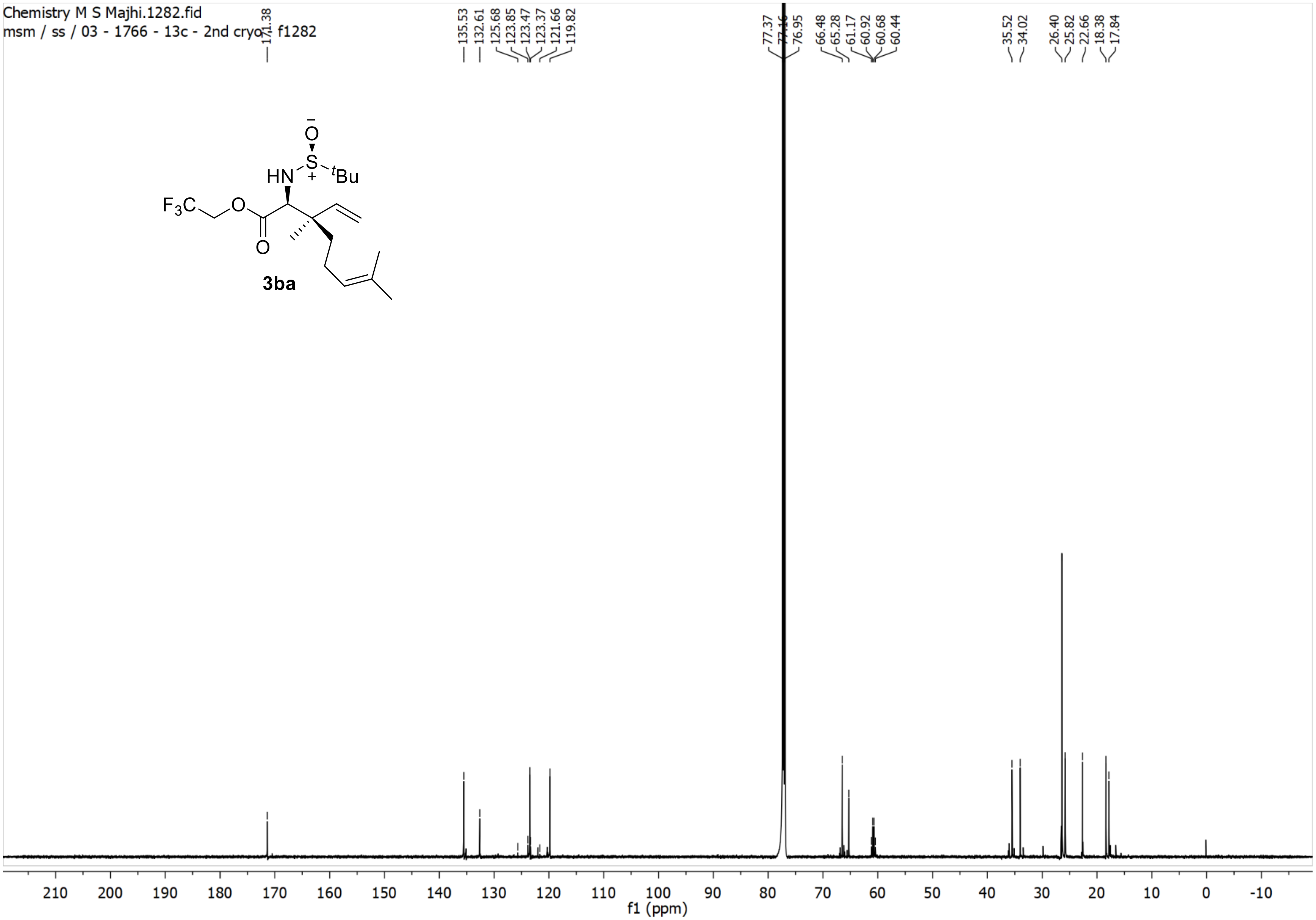

Chemistry M S Majhi.1282.fid
msm / ss / 03 - 1766 - 13c - 2nd cryo - f1282
171.38
135.53
132.61
125.68
123.85
123.47
123.37
121.66
119.82
77.37
77.16
76.95
66.48
65.28
61.17
60.92
60.68
60.44
35.52
34.02
26.40
25.82
22.66
18.38
17.84
HN
S
tBu
O
F3C
3ba
f1 (ppm)

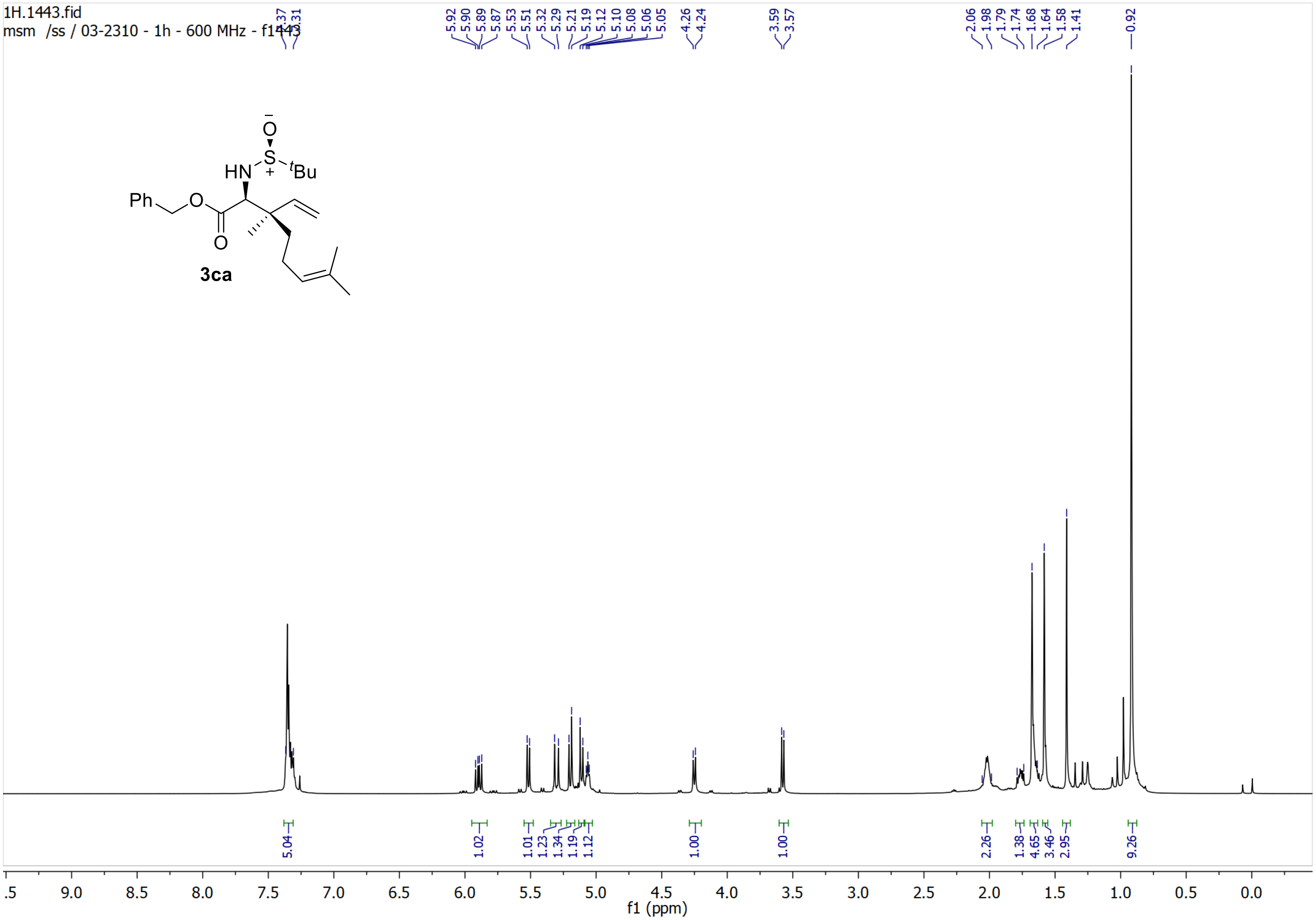

1H.1443.fid
msm /ss / 03-2310 - 1h - 600 MHz - f1443
7.37
7.31
5.92
5.90
5.89
5.87
5.53
5.51
5.32
5.29
5.21
5.19
5.12
5.10
5.08
5.06
5.05
4.26
4.24
3.59
3.57
2.06
1.98
1.79
1.74
1.68
1.64
1.58
1.41
0.92
HN
tBu
Ph
O
S
O
3ca
5.04
1.02
1.01
1.23
1.34
1.19
1.12
1.00
1.00
2.26
1.38
4.65
3.46
2.95
9.26
f1 (ppm)

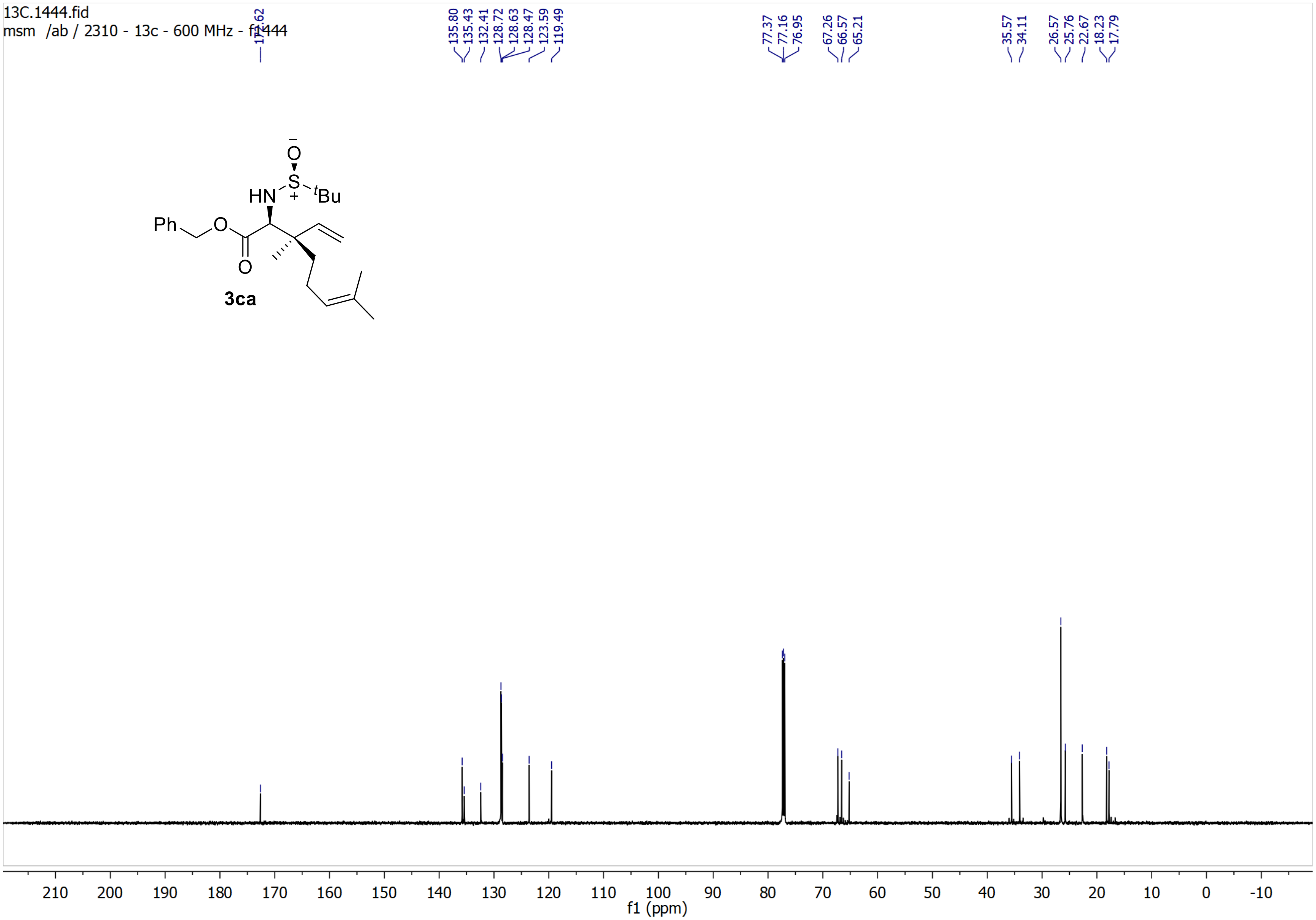

13C.1444.fid
msm /ab / 2310 - 13c - 600 MHz - f1444
172.62
135.80
135.43
132.41
128.72
128.63
128.47
123.59
119.49
77.37
77.16
76.95
67.26
66.57
65.21
35.57
34.11
26.57
25.76
22.67
18.23
17.79
HN
S
O
tBu
Ph
O
O
3ca
210 200 190 180 170 160 150 140 130 120 110 100 90 80 70 60 50 40 30 20 10 0 -10
f1 (ppm)

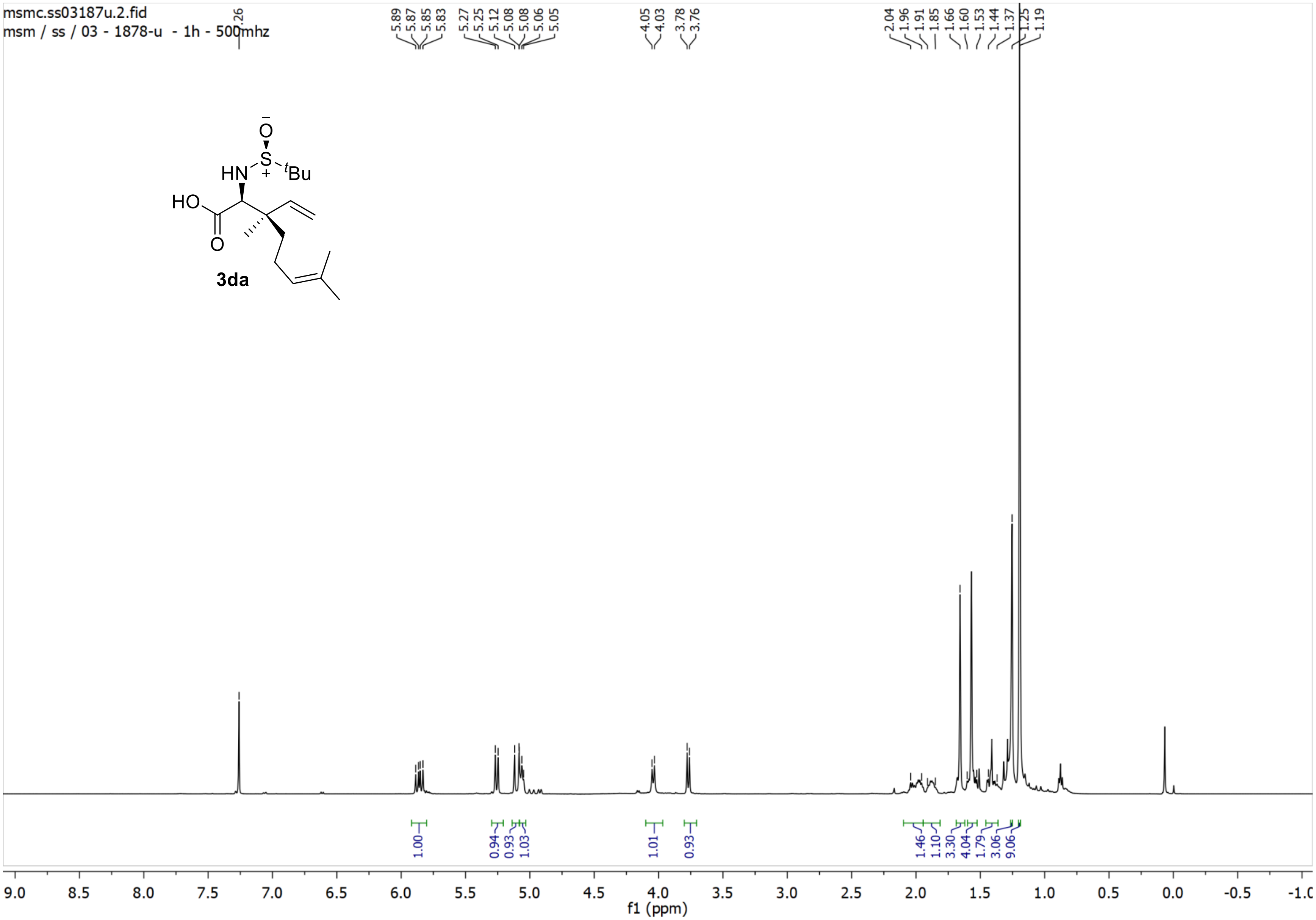
msmc.ss03187u.2.fid
msm / ss / 03 - 1878-u - 1h - 500mhz
7.26
5.89 5.87 5.85 5.83 5.27 5.25 5.12 5.08 5.08 5.06 5.05
4.05 4.03 3.78 3.76
2.04 1.96 1.91 1.85 1.66 1.60 1.53 1.44 1.37 1.25 1.19
HN
HO
O
S
+
tBu
3da
1.00
0.94 0.93 1.03
1.01
0.93
1.46 1.10 3.30 4.04 1.79 3.06 9.06
9.0 8.5 8.0 7.5 7.0 6.5 6.0 5.5 5.0 4.5 4.0 3.5 3.0 2.5 2.0 1.5 1.0 0.5 0.0 -0.5 -1.0
f1 (ppm)

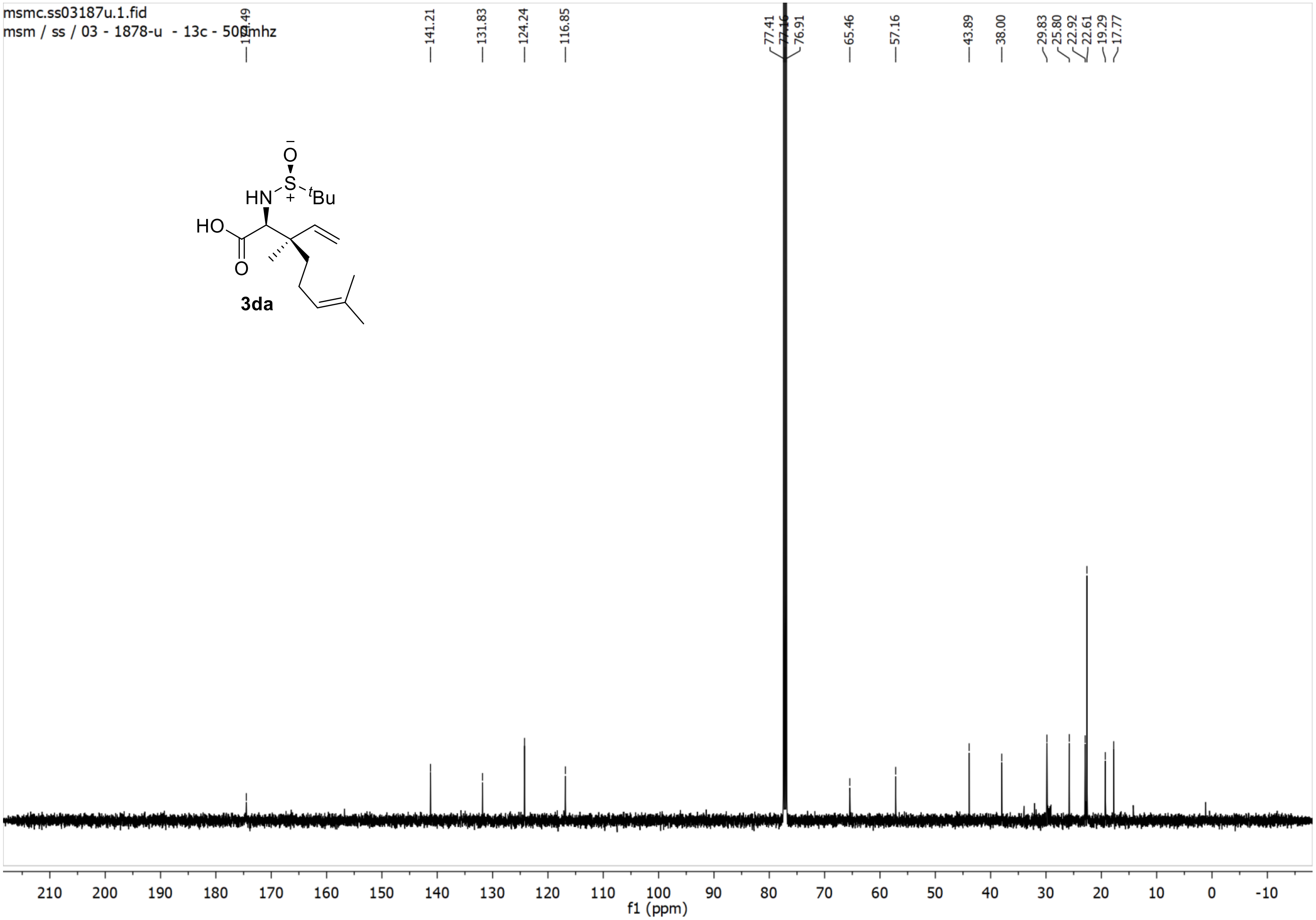
msmc.ss03187u.1.fid
msm / ss / 03 - 1878-u - 13c - 500mhz
141.21
131.83
124.24
116.85
77.41
77.16
76.91
65.46
57.16
43.89
38.00
29.83
25.80
22.92
22.61
19.29
17.77
HN
S
tBu
HO
O
3da
210 200 190 180 170 160 150 140 130 120 110 100 90 80 70 60 50 40 30 20 10 0 -10
f1 (ppm)

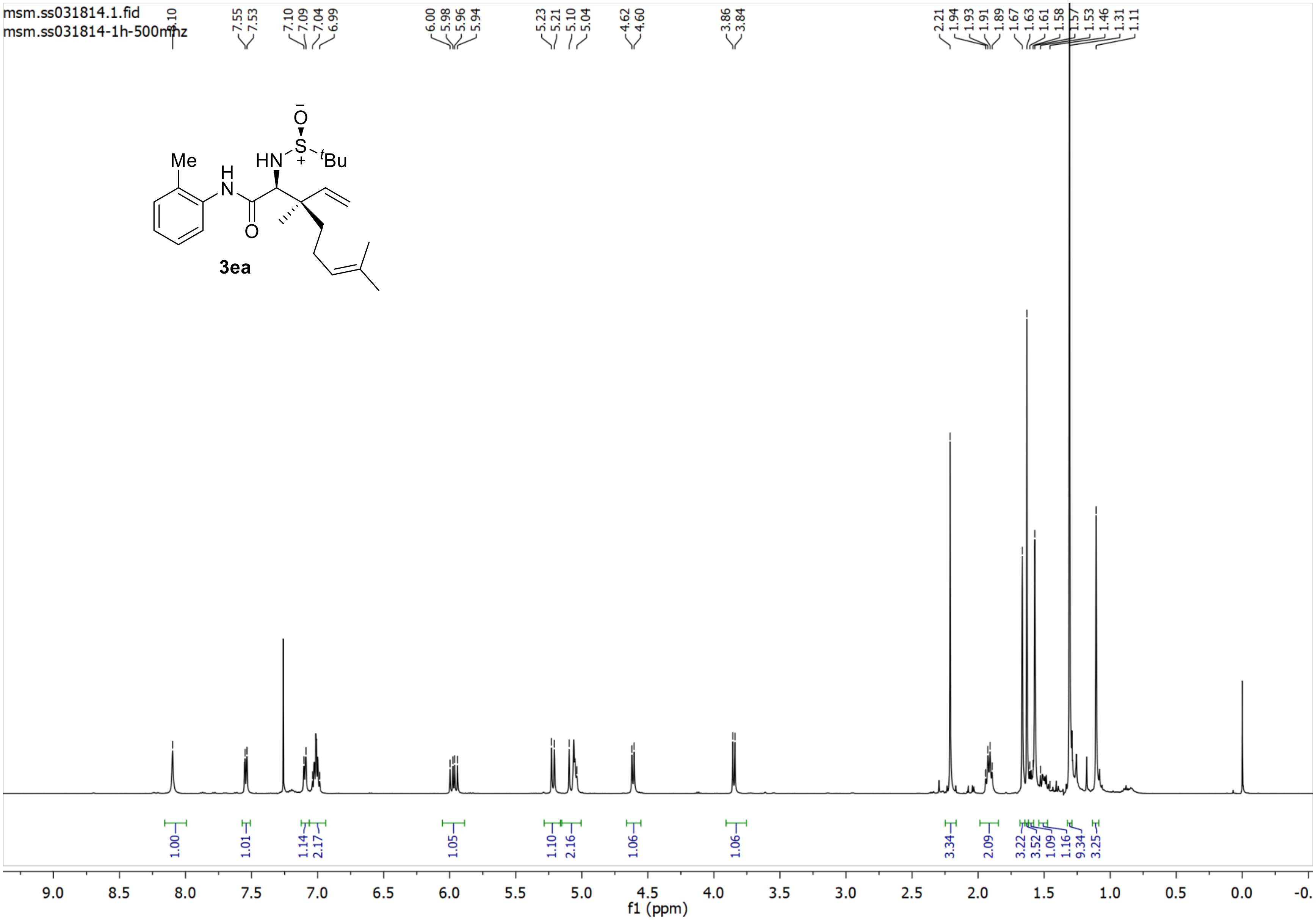
msm.ss031814.1.fid
msm.ss031814-1h-500mhz
8.10
7.55
7.53
7.10
7.09
7.04
6.99
6.00
5.98
5.96
5.94
5.23
5.21
5.10
5.04
4.62
4.60
3.86
3.84
2.21
1.94
1.93
1.91
1.89
1.67
1.63
1.61
1.58
1.57
1.53
1.46
1.31
1.11
Me
H
N
HN
S
O
tBu
O
3ea
1.00
1.01
1.14
2.17
1.05
1.10
2.16
1.06
1.06
3.34
2.09
3.22
3.52
1.09
1.16
9.34
3.25
9.0
8.5
8.0
7.5
7.0
6.5
6.0
5.5
5.0
4.5
4.0
3.5
3.0
2.5
2.0
1.5
1.0
0.5
0.0
-0.
f1 (ppm)

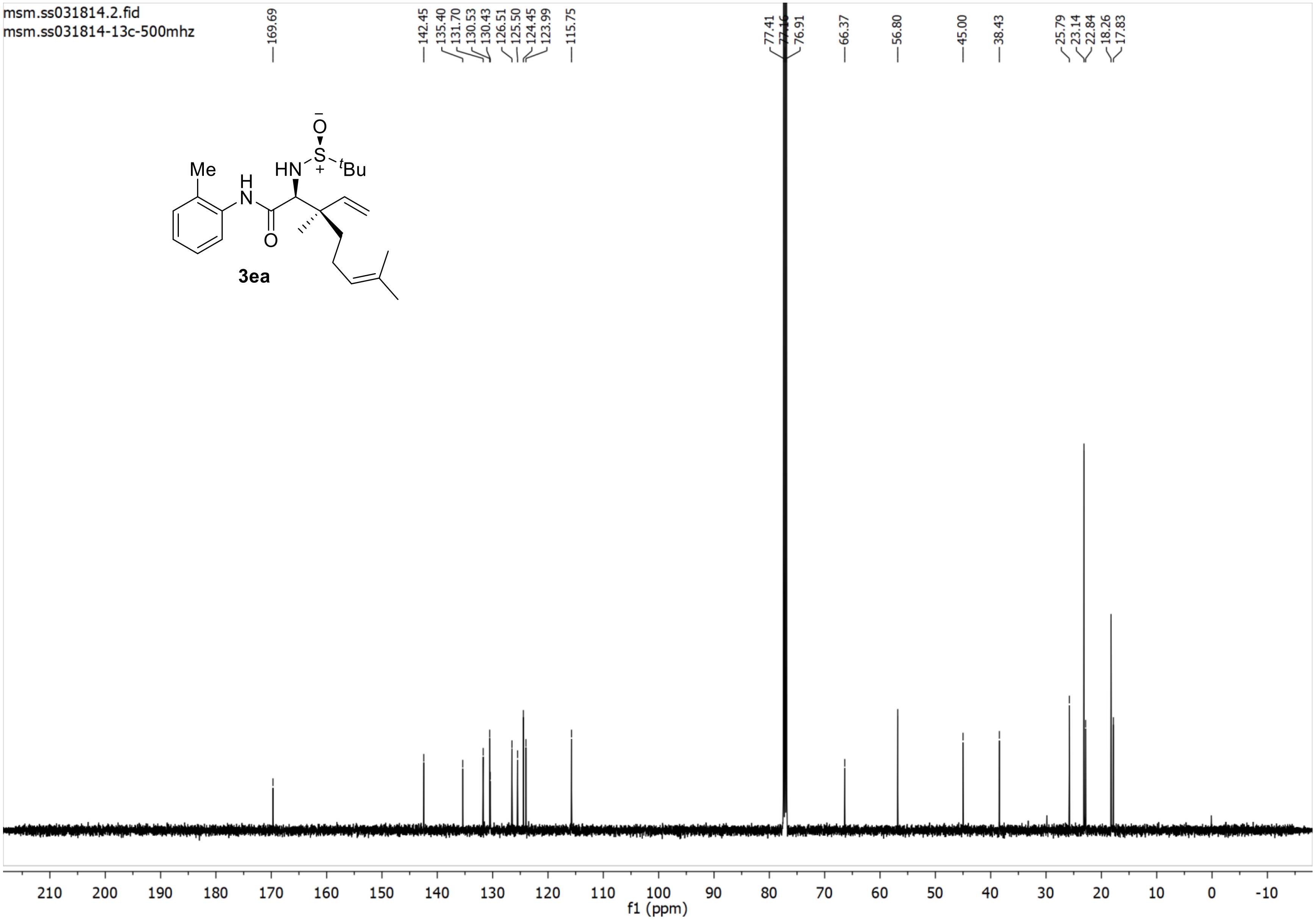
msm.ss031814.2.fid
msm.ss031814-13c-500mhz
169.69
142.45
135.40
131.70
130.53
130.43
126.51
125.50
124.45
123.99
115.75
77.41
76.91
66.37
56.80
45.00
38.43
25.79
23.14
22.84
18.26
17.83
Me
H
N
HN
O
S
tBu
3ea
f1 (ppm)

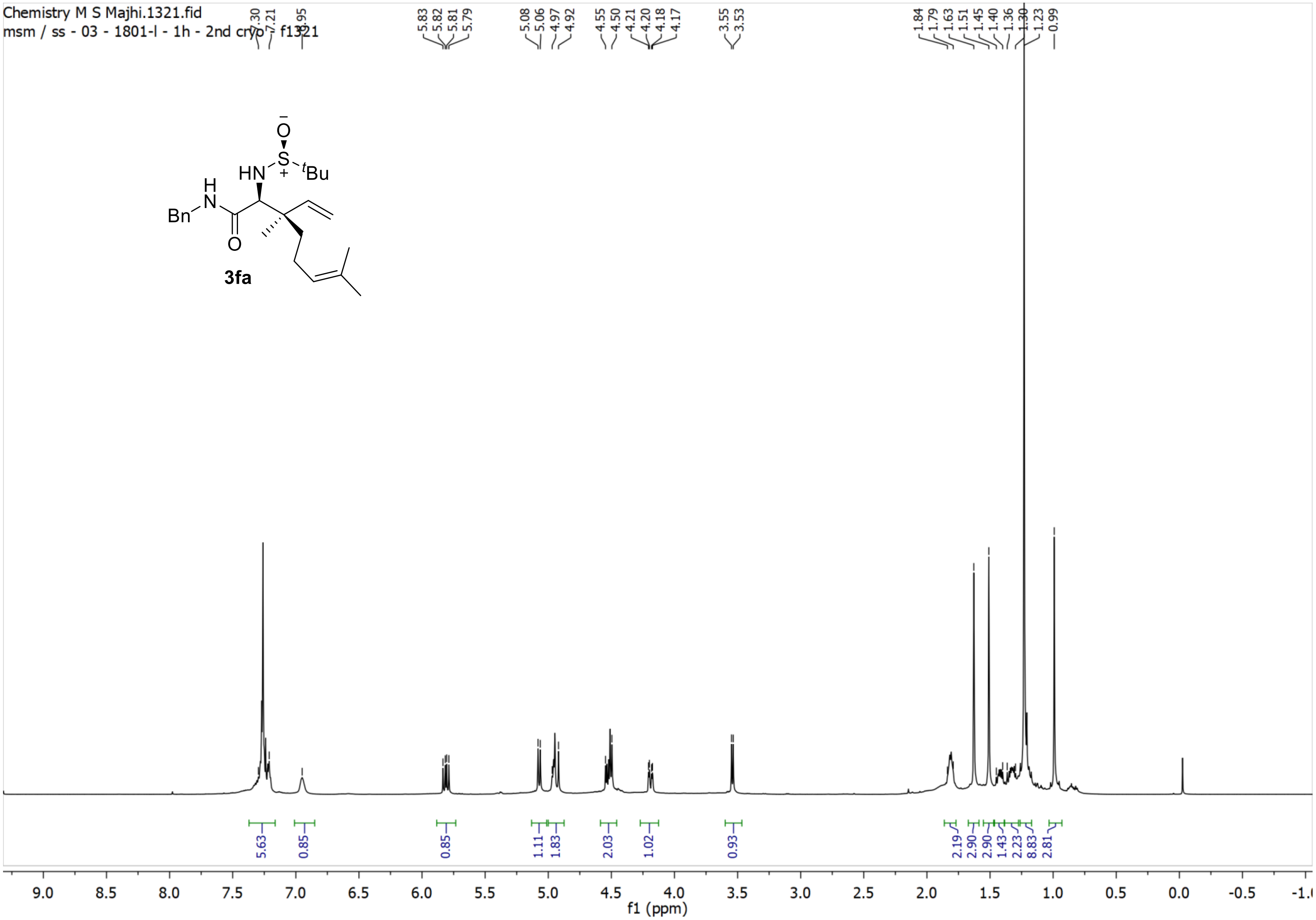
Chemistry M S Majhi.1321.fid
msm / ss - 03 - 1801-l - 1h - 2nd cryo - f1321
3fa
f1 (ppm)

Chemistry M S Majhi.1322.fid
msm / ss - 03 - 1801-l - 13c - 2nd cryo - f1322

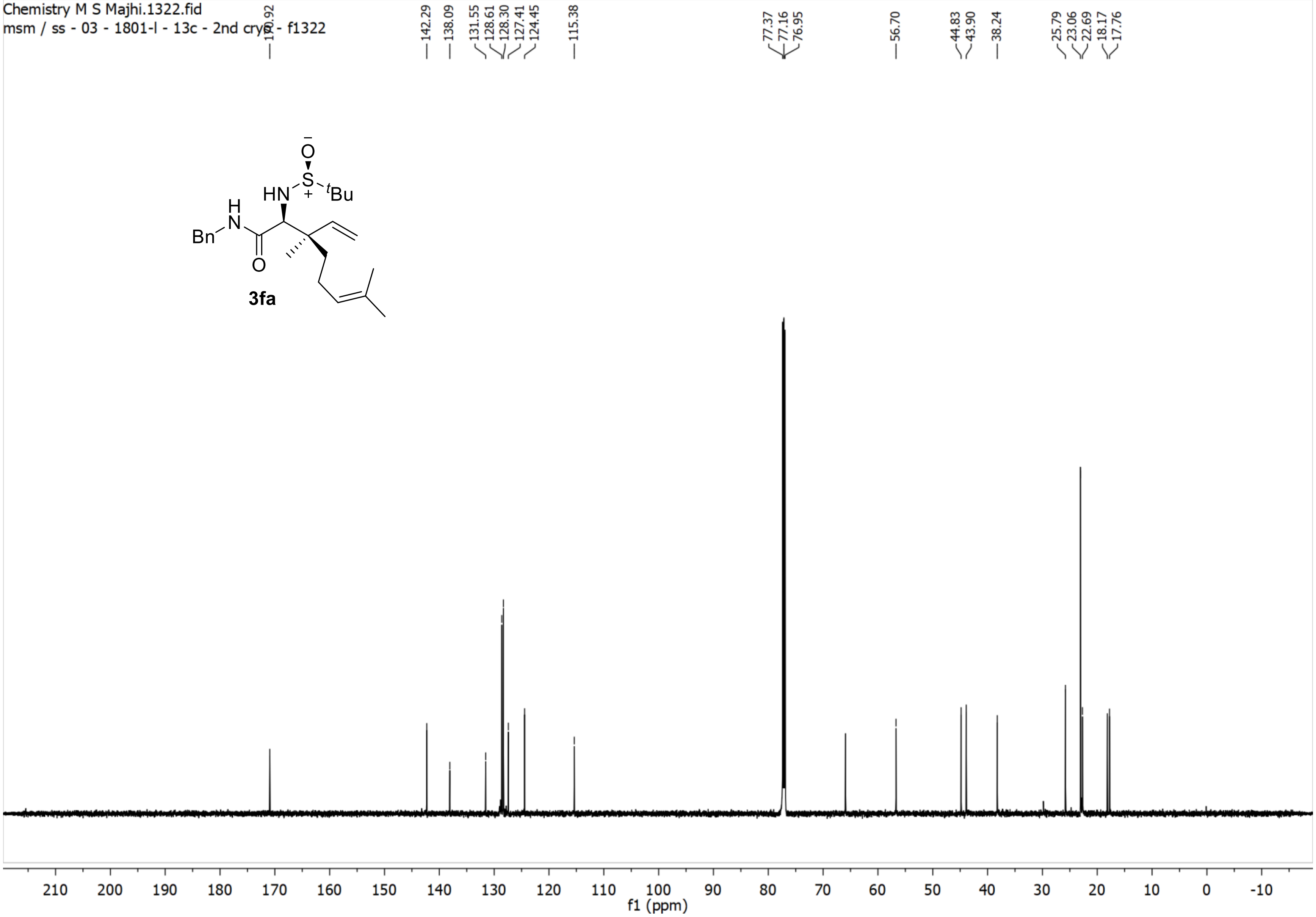

3fa

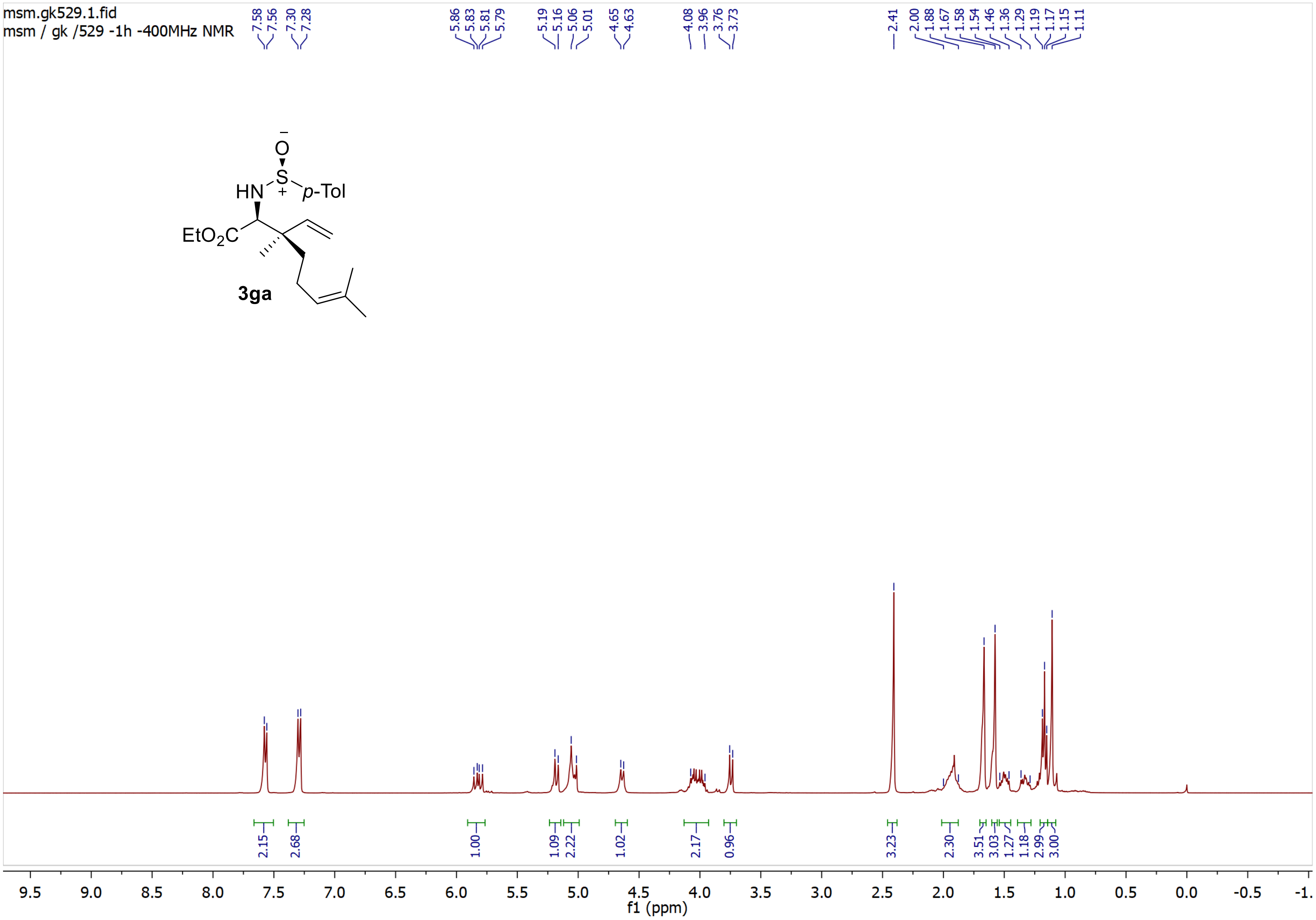

msm.gk529.1.fid
msm / gk /529 -1h -400MHz NMR
7.58
7.56
7.30
7.28
5.86
5.83
5.81
5.79
5.19
5.16
5.06
5.01
4.65
4.63
4.08
3.96
3.76
3.73
2.41
2.00
1.88
1.67
1.58
1.54
1.46
1.36
1.29
1.19
1.17
1.15
1.11
HN
S
+
O
−
p-Tol
EtO2C
3ga
2.15
2.68
1.00
1.09
2.22
1.02
2.17
0.96
3.23
2.30
3.51
3.03
1.27
1.18
2.99
3.00
9.5
9.0
8.5
8.0
7.5
7.0
6.5
6.0
5.5
5.0
4.5
4.0
3.5
3.0
2.5
2.0
1.5
1.0
0.5
0.0
-0.5
f1 (ppm)

msm.gk529.4.fid
msm / gk /529 -13C -400MHz NMR

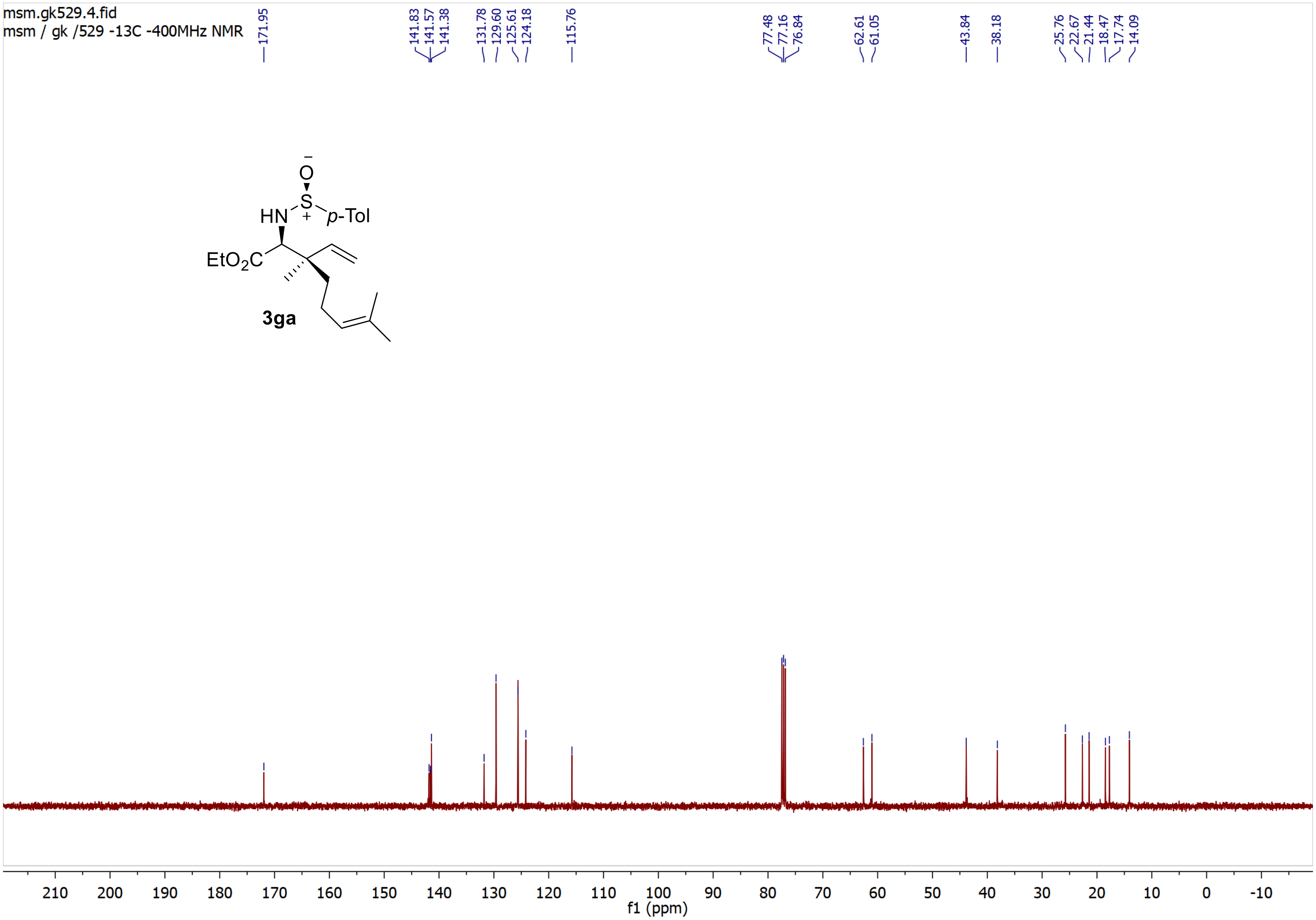

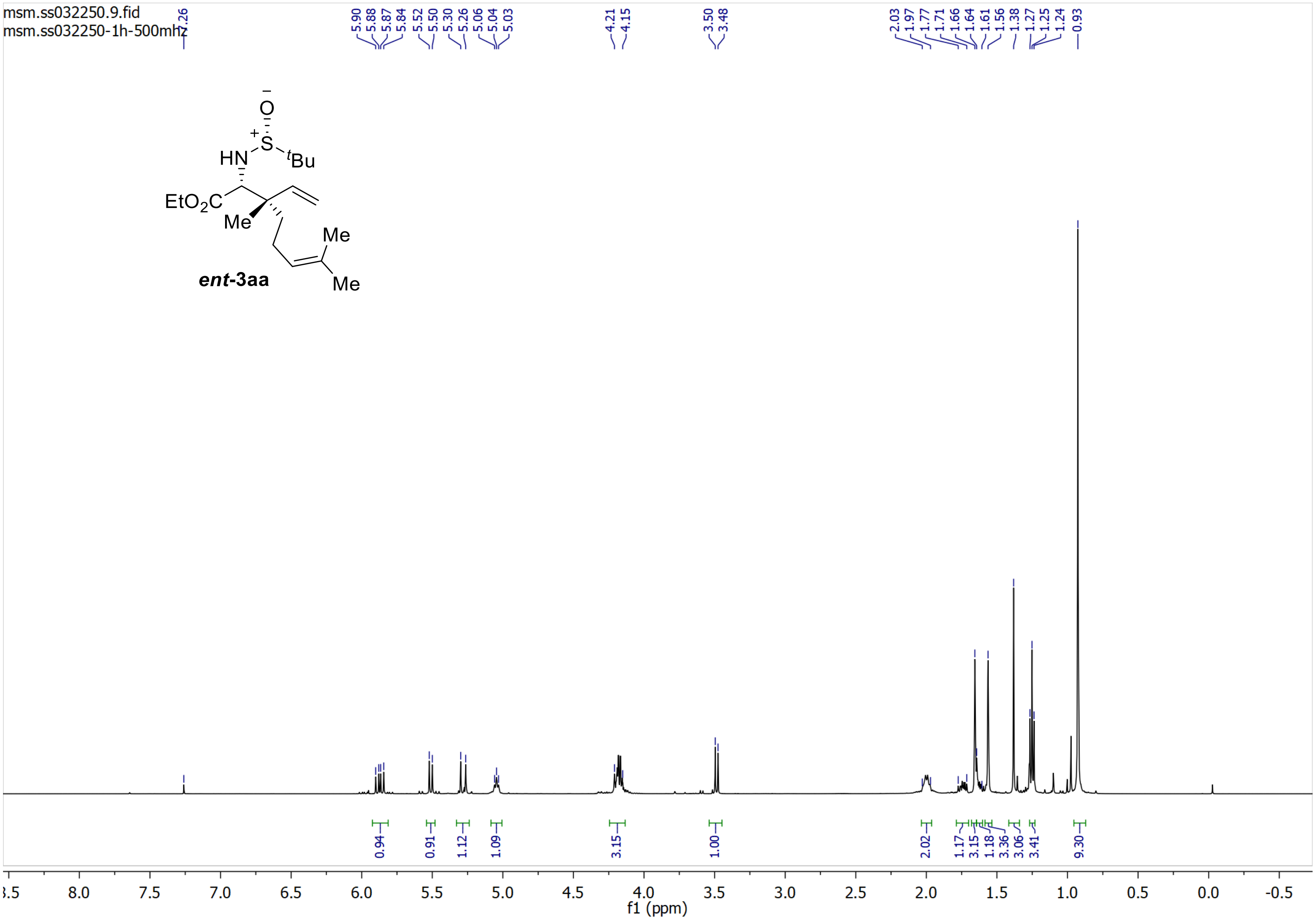
msm.ss032250.9.fid
msm.ss032250-1h-500mhz
7.26
5.90 5.88 5.87 5.84 5.52 5.50 5.30 5.26 5.06 5.04 5.03
4.21 4.15
3.50 3.48
2.03 1.97 1.77 1.71 1.66 1.64 1.61 1.56 1.38 1.27 1.25 1.24 0.93
HN
S
tBu
O
EtO2C
Me
Me
Me
ent-3aa
0.94 0.91 1.12 1.09 3.15 1.00 2.02 1.17 3.15 1.18 3.36 3.06 3.41 9.30
8.0 7.5 7.0 6.5 6.0 5.5 5.0 4.5 4.0 3.5 3.0 2.5 2.0 1.5 1.0 0.5 0.0 -0.5
f1 (ppm)

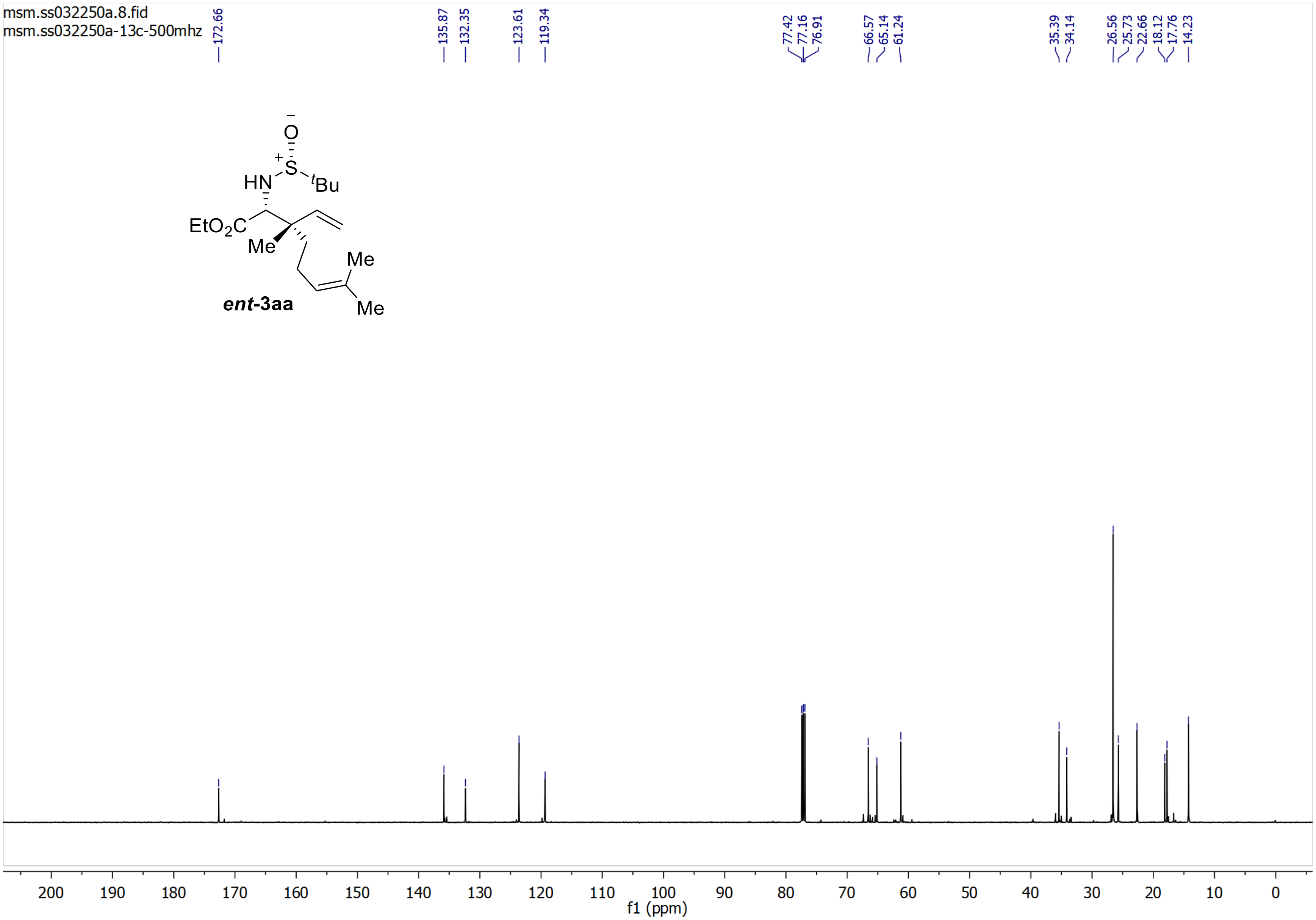

msm.ss032250a.8.fid
msm.ss032250a-13c-500mhz
172.66
135.87
132.35
123.61
119.34
77.42
77.16
76.91
66.57
65.14
61.24
35.39
34.14
26.56
25.73
22.66
18.12
17.76
14.23
HN
S
tBu
O
EtO2C
Me
Me
Me
ent-3aa
f1 (ppm)

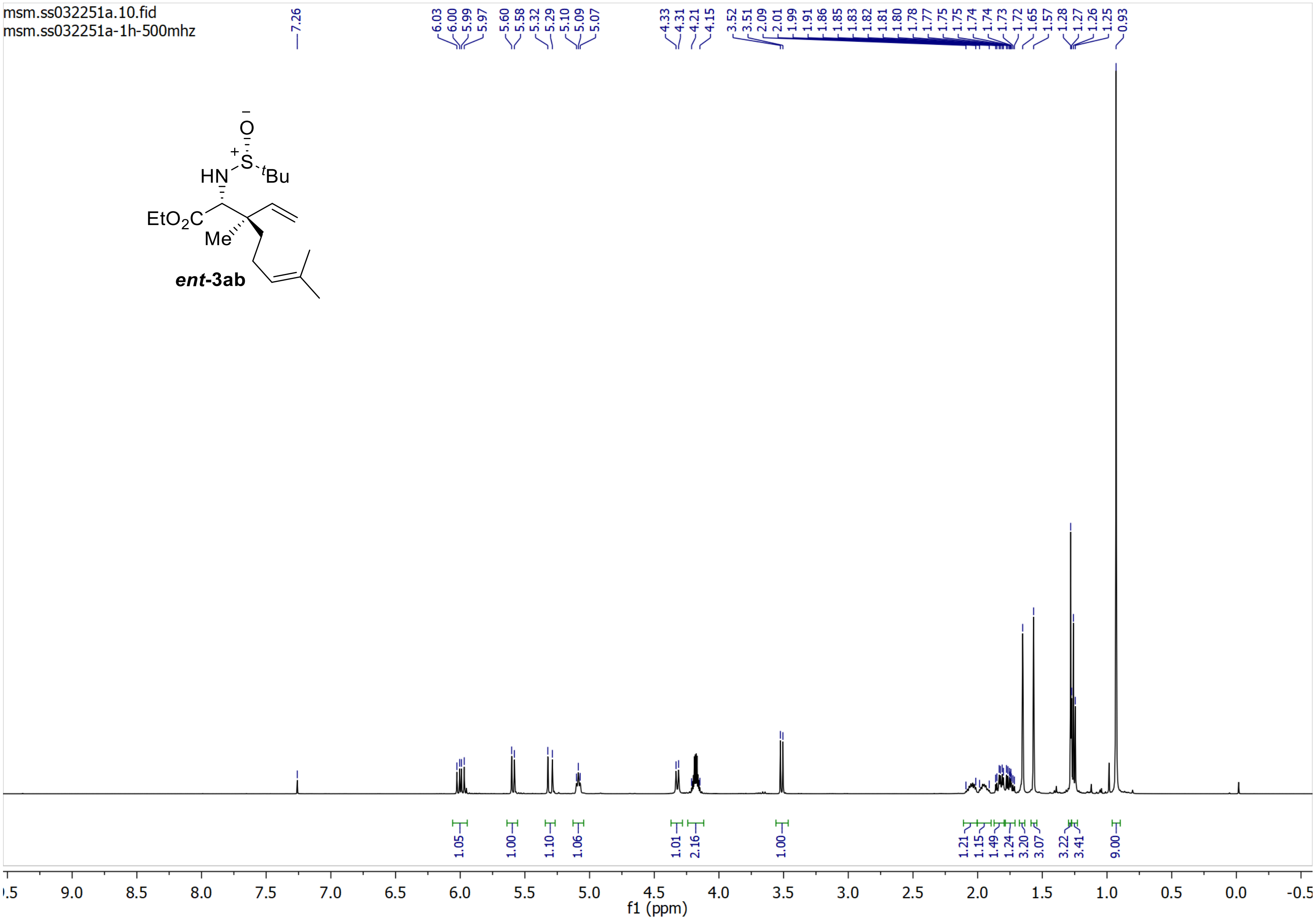

msm.ss032251a.10.fid
msm.ss032251a-1h-500mhz
ent-3ab
f1 (ppm)

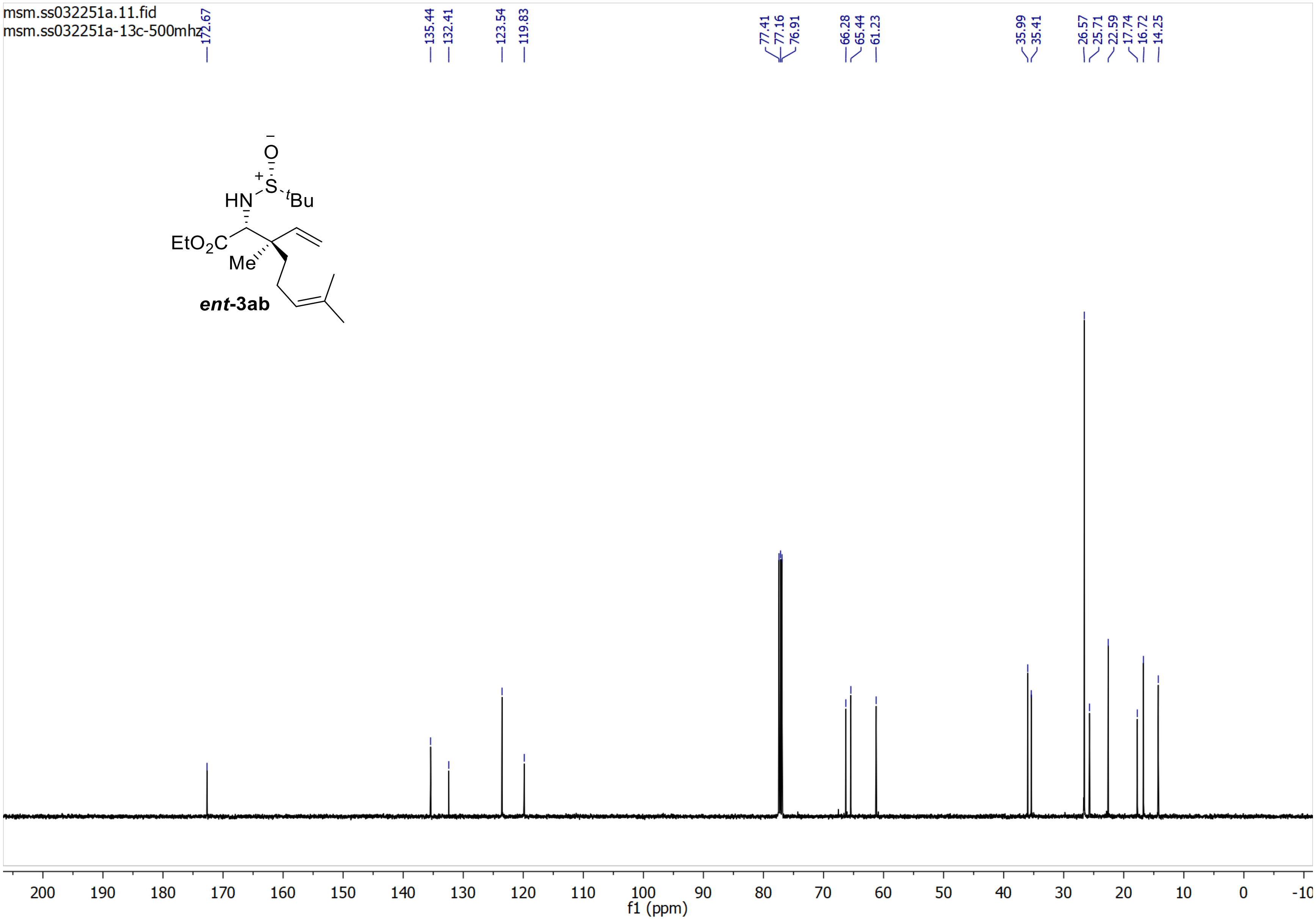
msm.ss032251a.11.fid
msm.ss032251a-13c-500mhz
172.67
135.44
132.41
123.54
119.83
77.41
77.16
76.91
66.28
65.44
61.23
35.99
35.41
26.57
25.71
22.59
17.74
16.72
14.25
HN
S
tBu
O
EtO2C
Me
ent-3ab
200
190
180
170
160
150
140
130
120
110
100
90
80
70
60
50
40
30
20
10
0
-10
f1 (ppm)

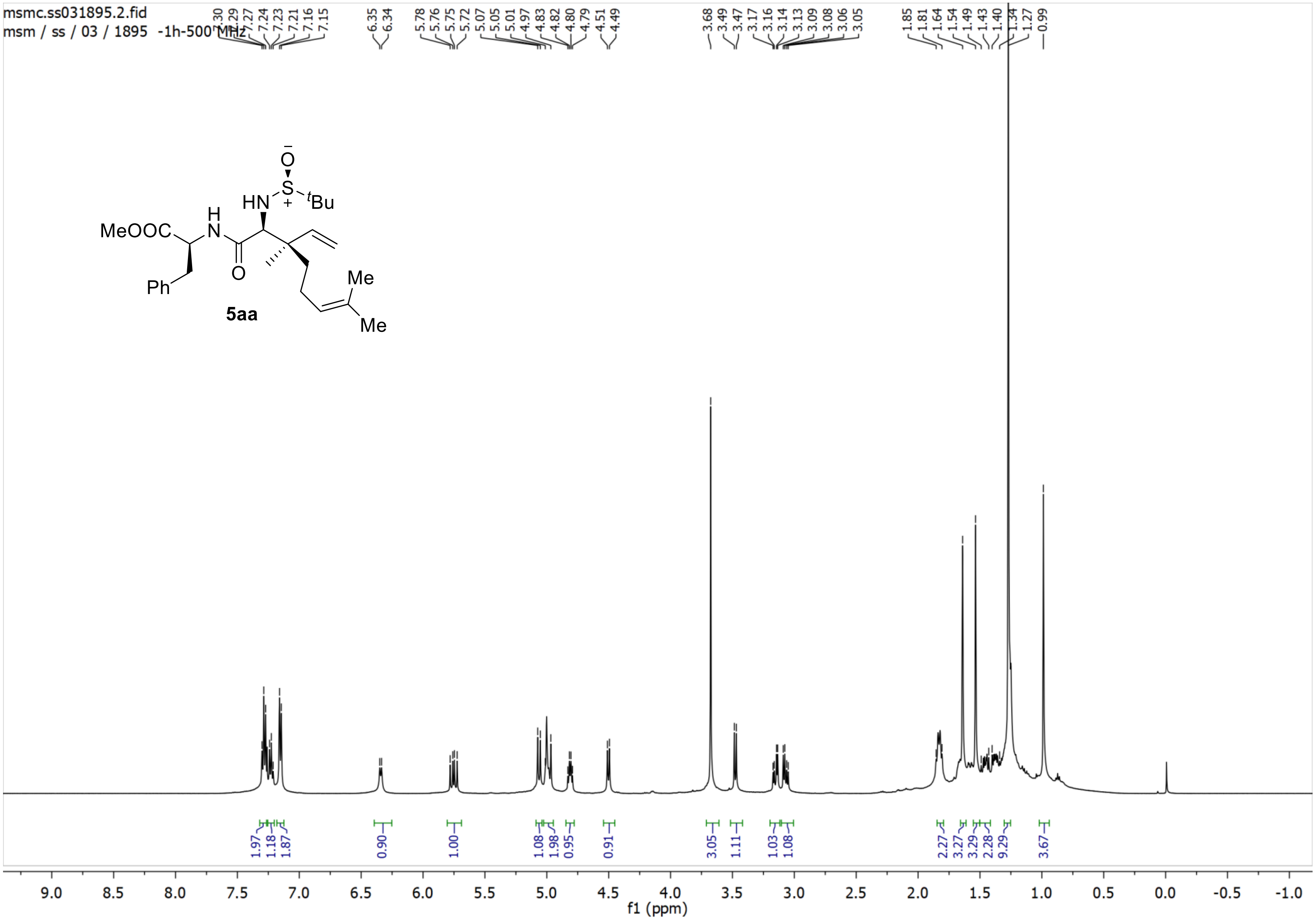
msmc.ss031895.2.fid
msm / ss / 03 / 1895 -1h-500 MHz
5aa
f1 (ppm)

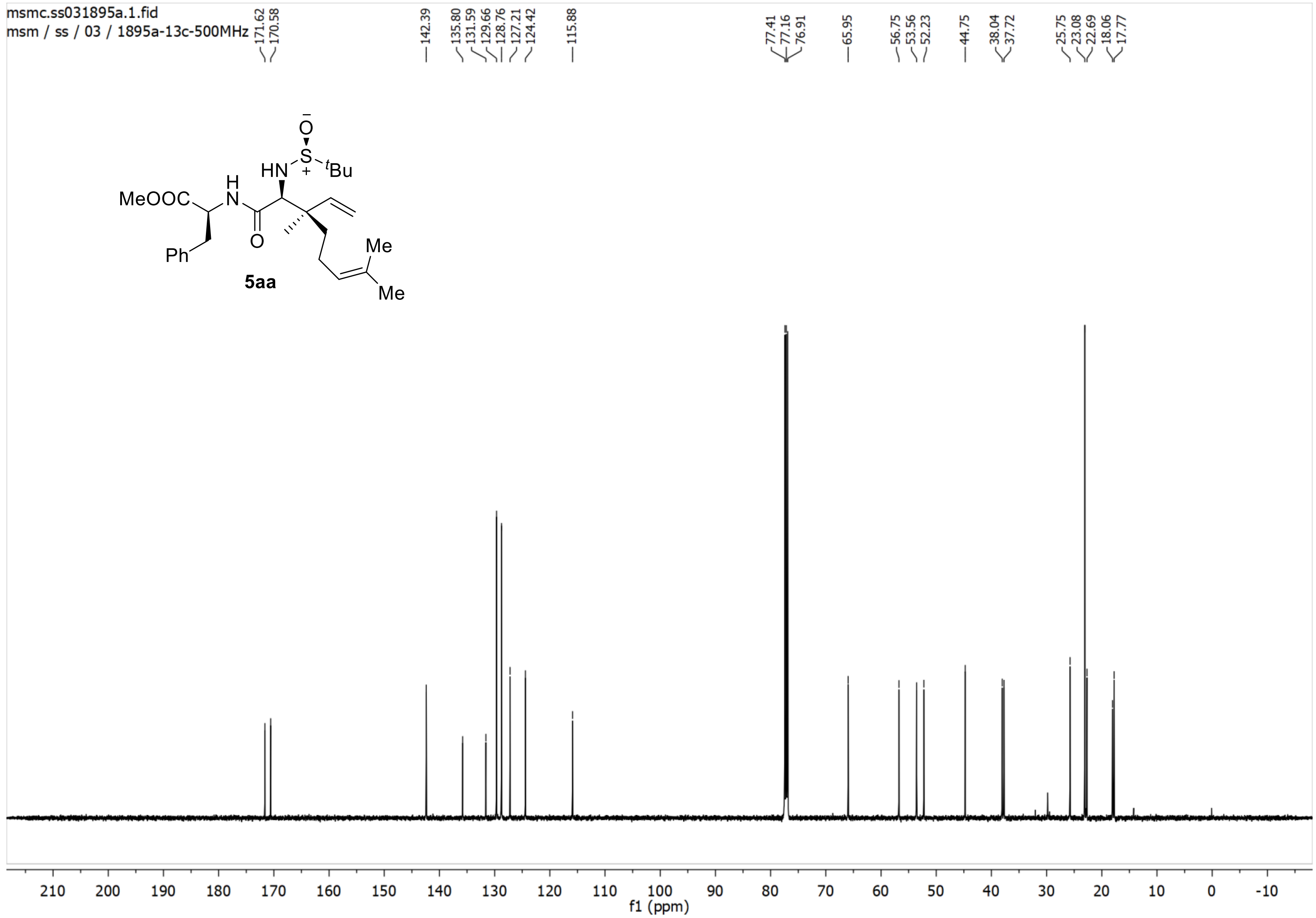

msmc.ss031895a.1.fid
msm / ss / 03 / 1895a-13c-500MHz
171.62
170.58
142.39
135.80
131.59
129.66
128.76
127.21
124.42
115.88
77.41
77.16
76.91
65.95
56.75
53.56
52.23
44.75
38.04
37.72
25.75
23.08
22.69
18.06
17.77
MeOOC
HN
S
O
tBu
Ph
O
Me
Me
5aa
210 200 190 180 170 160 150 140 130 120 110 100 90 80 70 60 50 40 30 20 10 0 -10
f1 (ppm)

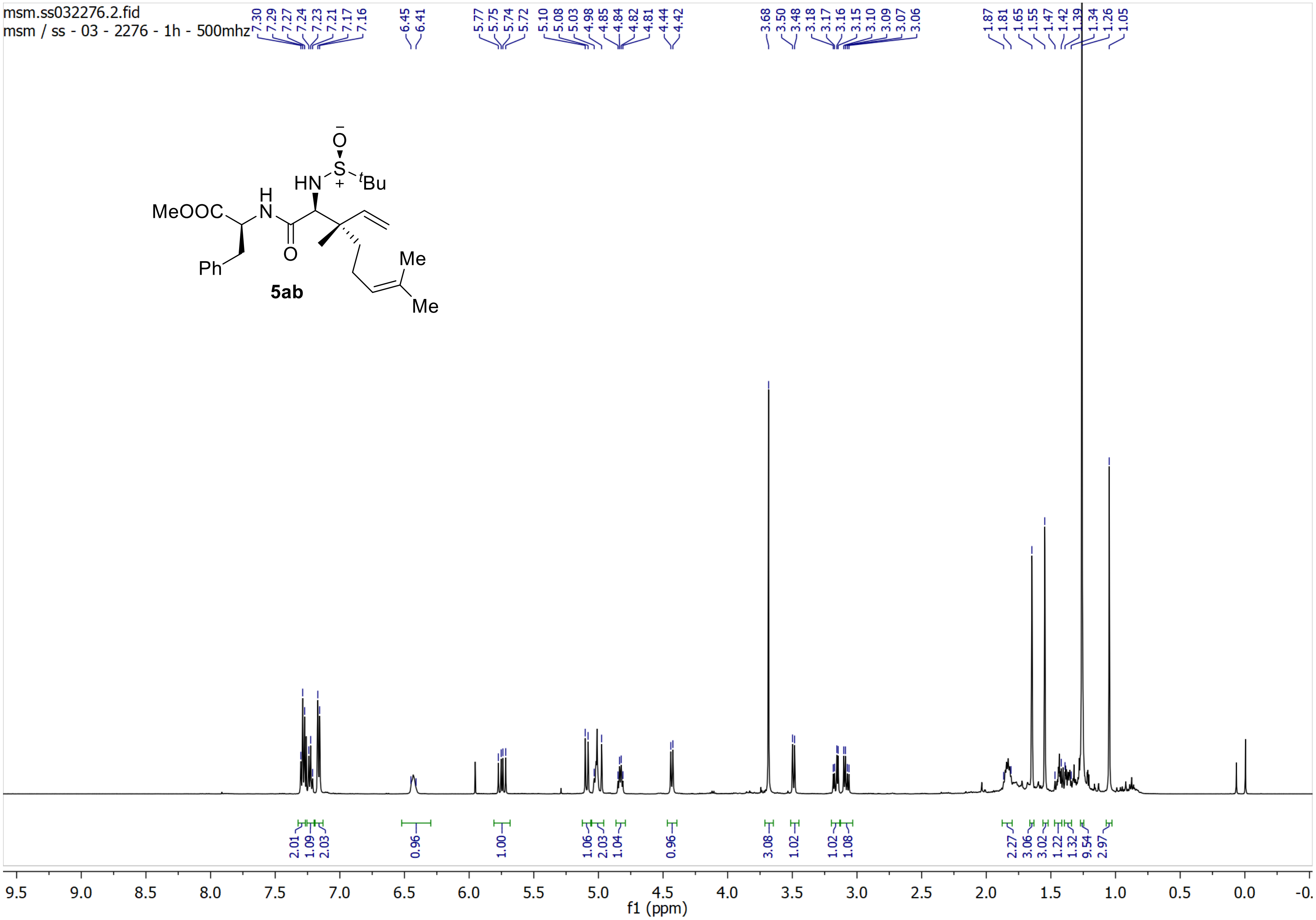
msm.ss032276.2.fid
msm / ss - 03 - 2276 - 1h - 500mhz
7.30 7.29 7.27 7.24 7.23 7.21 7.17 7.16
6.45 6.41
5.77 5.75 5.74 5.72 5.10 5.08 5.03 4.98 4.85 4.84 4.82 4.81 4.44 4.42
3.68 3.50 3.48 3.18 3.17 3.16 3.15 3.10 3.09 3.07 3.06
1.87 1.81 1.65 1.55 1.47 1.42 1.39 1.34 1.26 1.05
MeOOC
HN
O
S
tBu
Ph
Me
Me
5ab
2.01 1.09 2.03 0.96 1.00 1.06 2.03 1.04 0.96 3.08 1.02 1.02 1.08 2.27 3.06 3.02 1.22 1.32 9.54 2.97
9.5 9.0 8.5 8.0 7.5 7.0 6.5 6.0 5.5 5.0 4.5 4.0 3.5 3.0 2.5 2.0 1.5 1.0 0.5 0.0 -0.
f1 (ppm)

msm.ss032276.3.fid
msm / ss - 03 - 2276 - 1h - 500mhz

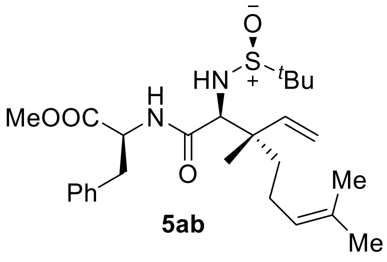

5ab

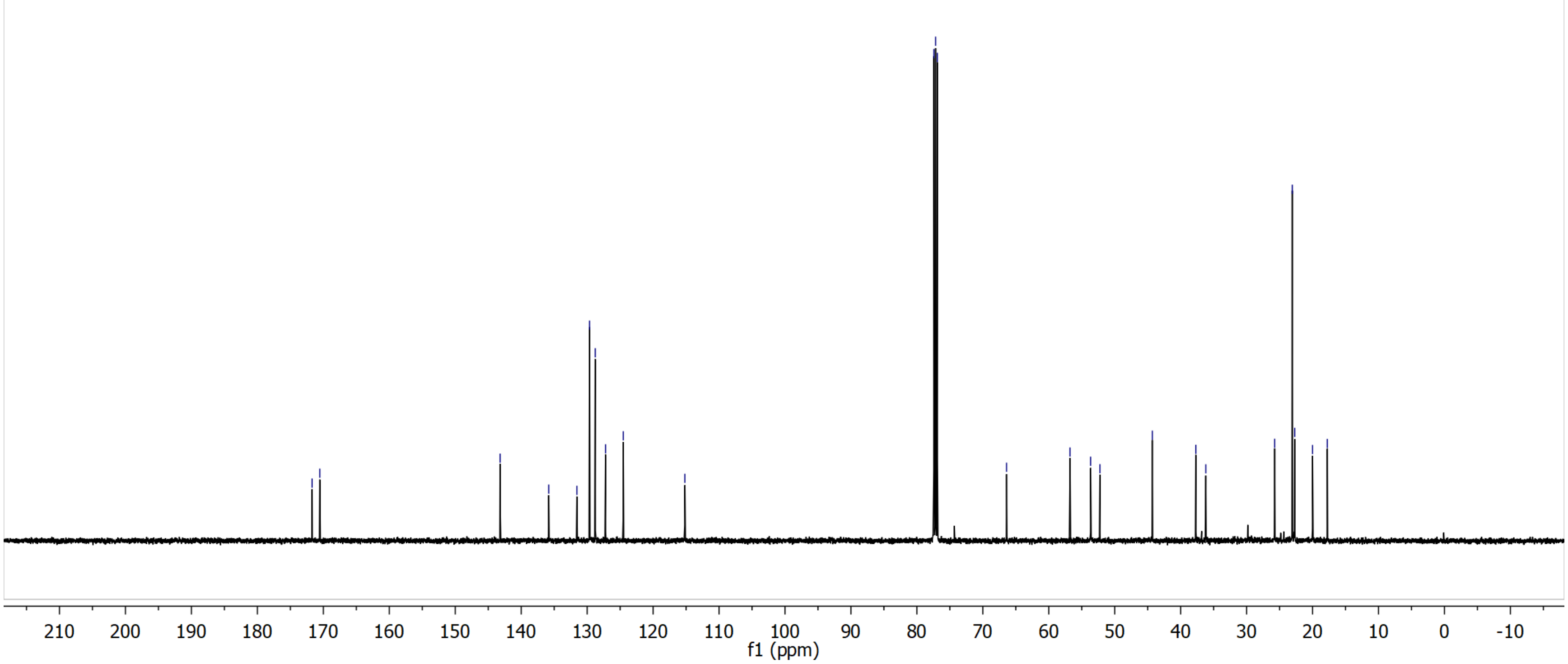

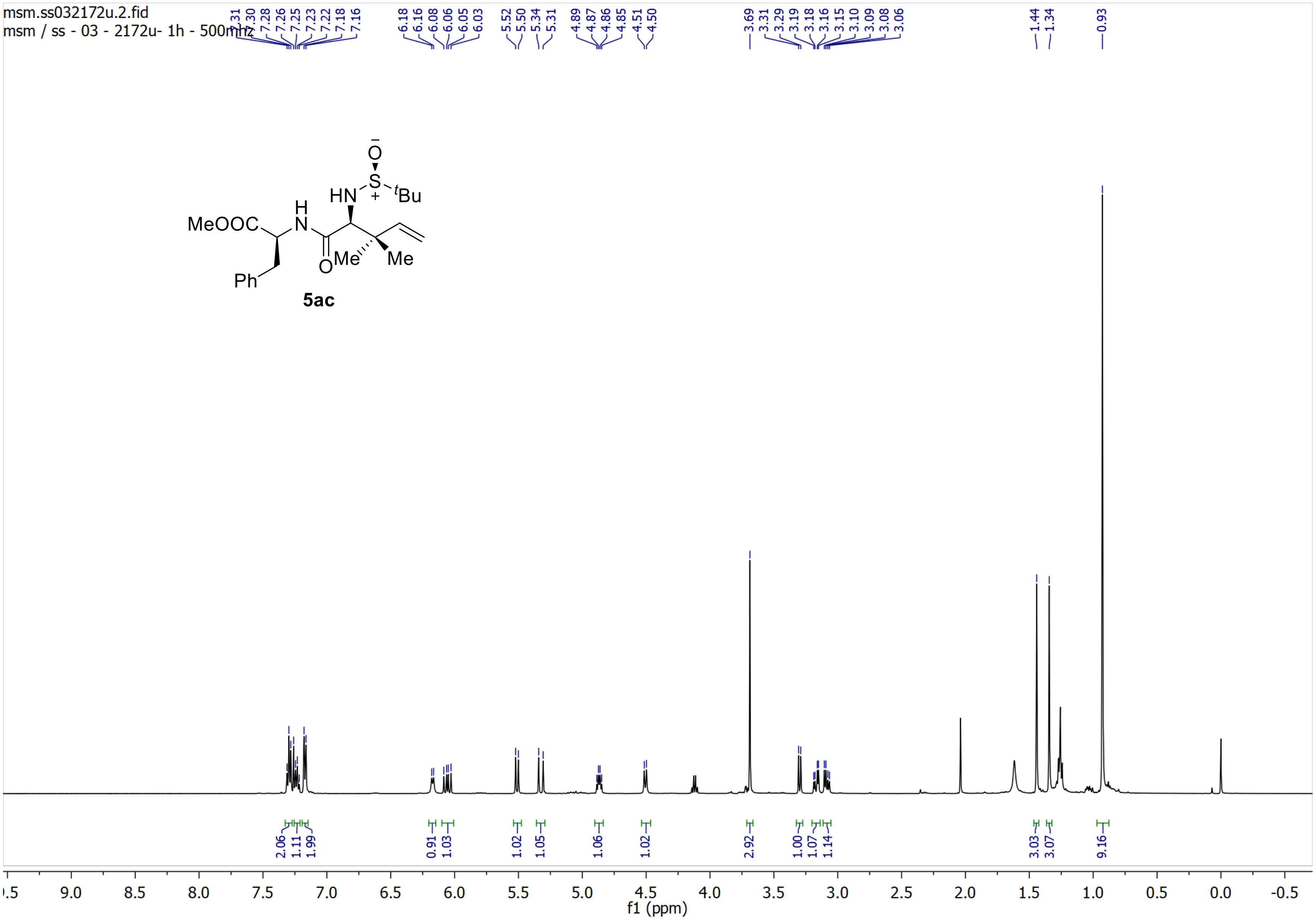

msm.ss032172u.2.fid
msm / ss - 03 - 2172u- 1h - 500mhz
MeOOC
HN
S
tBu
O
Me
Me
Ph
5ac
f1 (ppm)

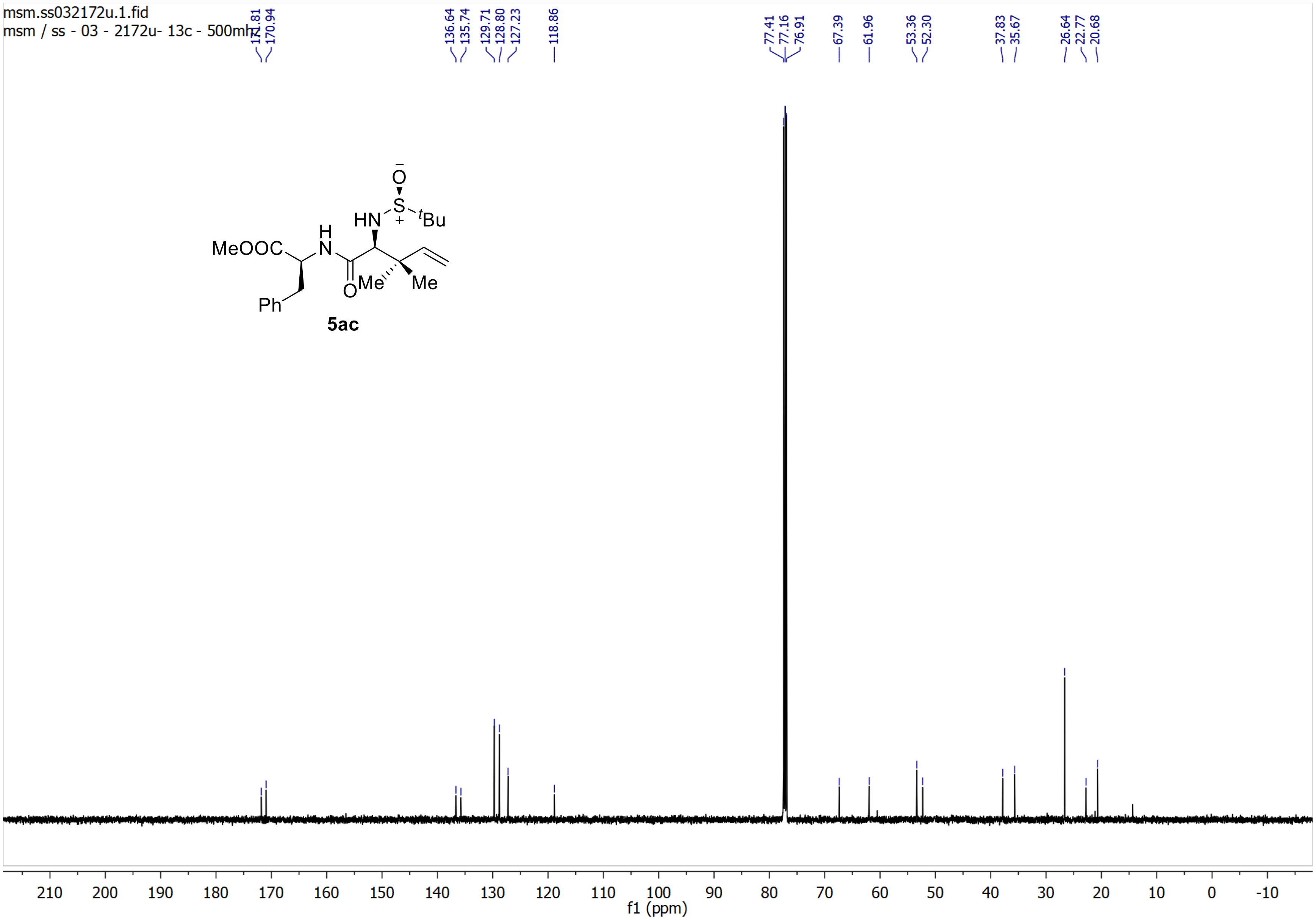
msm.ss032172u.1.fid
msm / ss - 03 - 2172u- 13c - 500mhz
171.81
170.94
136.64
135.74
129.71
128.80
127.23
118.86
77.41
77.16
76.91
67.39
61.96
53.36
52.30
37.83
35.67
26.64
22.77
20.68
MeOOC
Ph
HN
S
tBu
Me
Me
5ac
f1 (ppm)

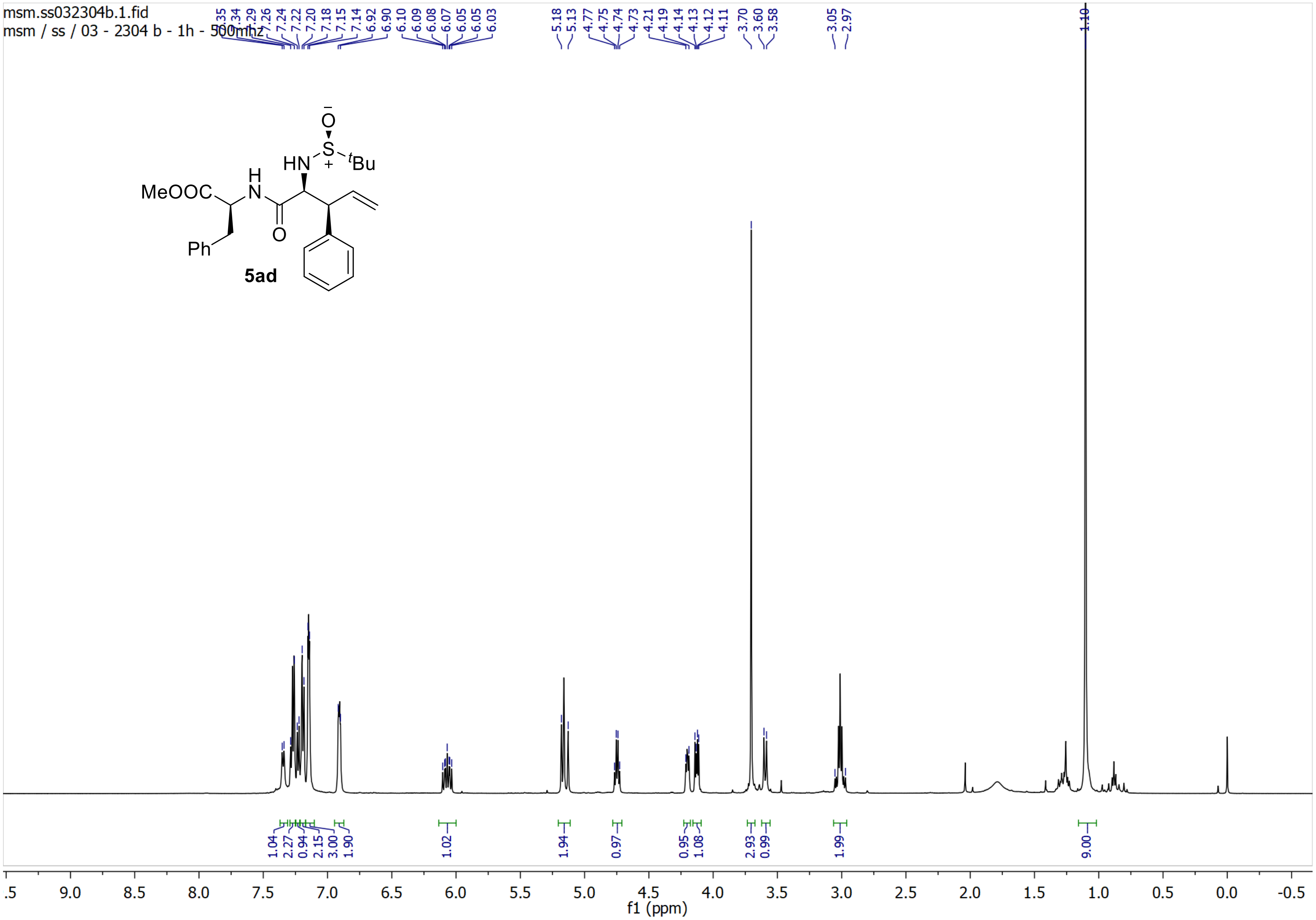

msm.ss032304b.1.fid
msm / ss / 03 - 2304 b - 1h - 500mhz
7.35
7.34
7.29
7.26
7.24
7.22
7.20
7.18
7.15
7.14
6.92
6.90
6.10
6.09
6.08
6.07
6.05
6.05
6.03
5.18
5.13
4.77
4.75
4.74
4.73
4.21
4.19
4.14
4.13
4.12
4.11
3.70
3.60
3.58
3.05
2.97
1.10
MeOOC
H
N
O
HN
S
+
O
tBu
Ph
5ad
1.04
2.27
0.94
2.15
3.00
1.90
1.02
1.94
0.97
0.95
1.08
2.93
0.99
1.99
9.00
.5
9.0
8.5
8.0
7.5
7.0
6.5
6.0
5.5
5.0
4.5
4.0
3.5
3.0
2.5
2.0
1.5
1.0
0.5
0.0
-0.5
f1 (ppm)

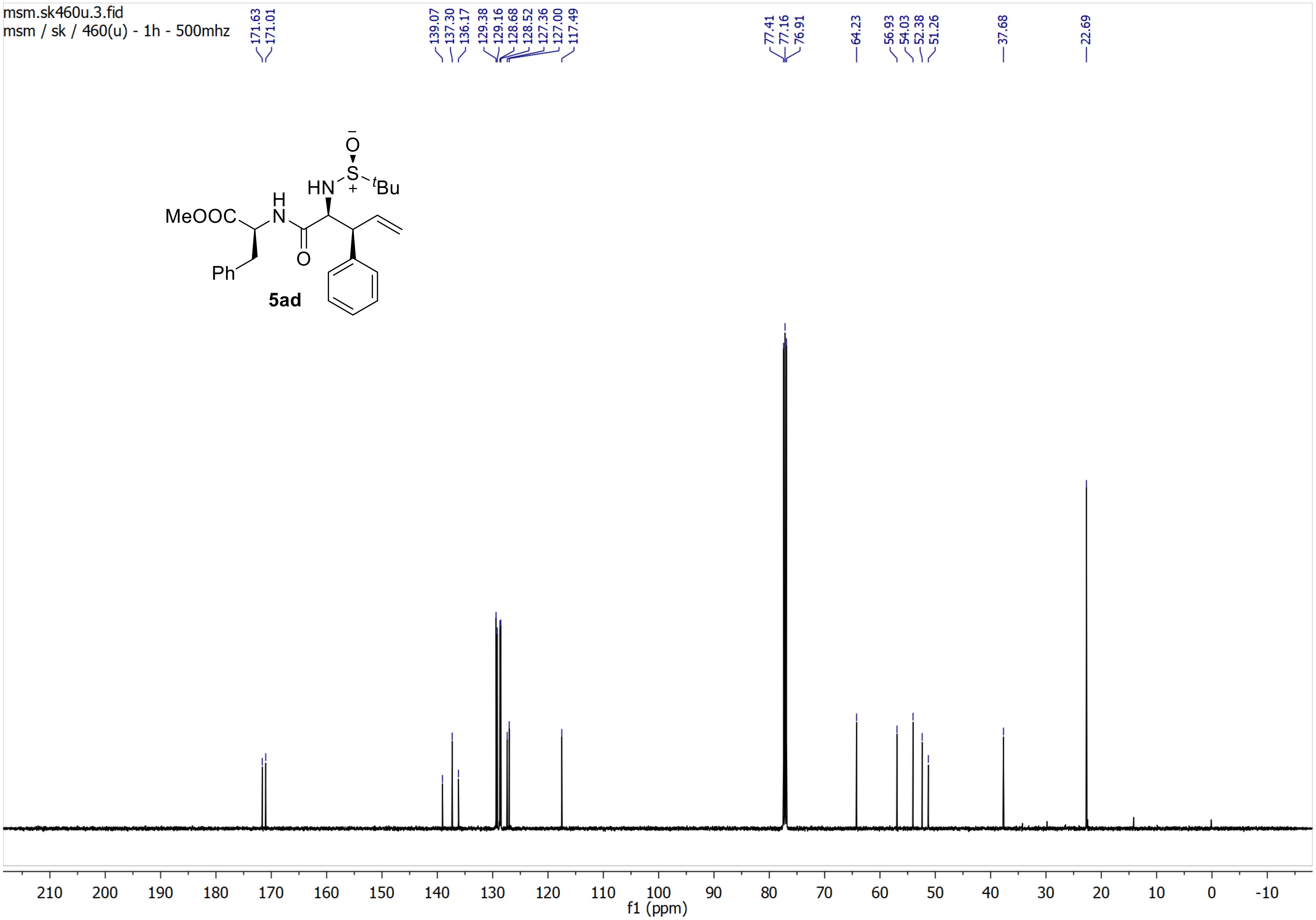

msm.sk460u.3.fid
msm / sk / 460(u) - 1h - 500mhz
171.63
171.01
139.07
137.30
136.17
129.38
129.16
128.68
128.52
127.36
127.00
117.49
77.41
77.16
76.91
64.23
56.93
54.03
52.38
51.26
37.68
22.69
MeOOC
H
N
O
HN
S
O
tBu
Ph
5ad
210 200 190 180 170 160 150 140 130 120 110 100 90 80 70 60 50 40 30 20 10 0 -10
f1 (ppm)

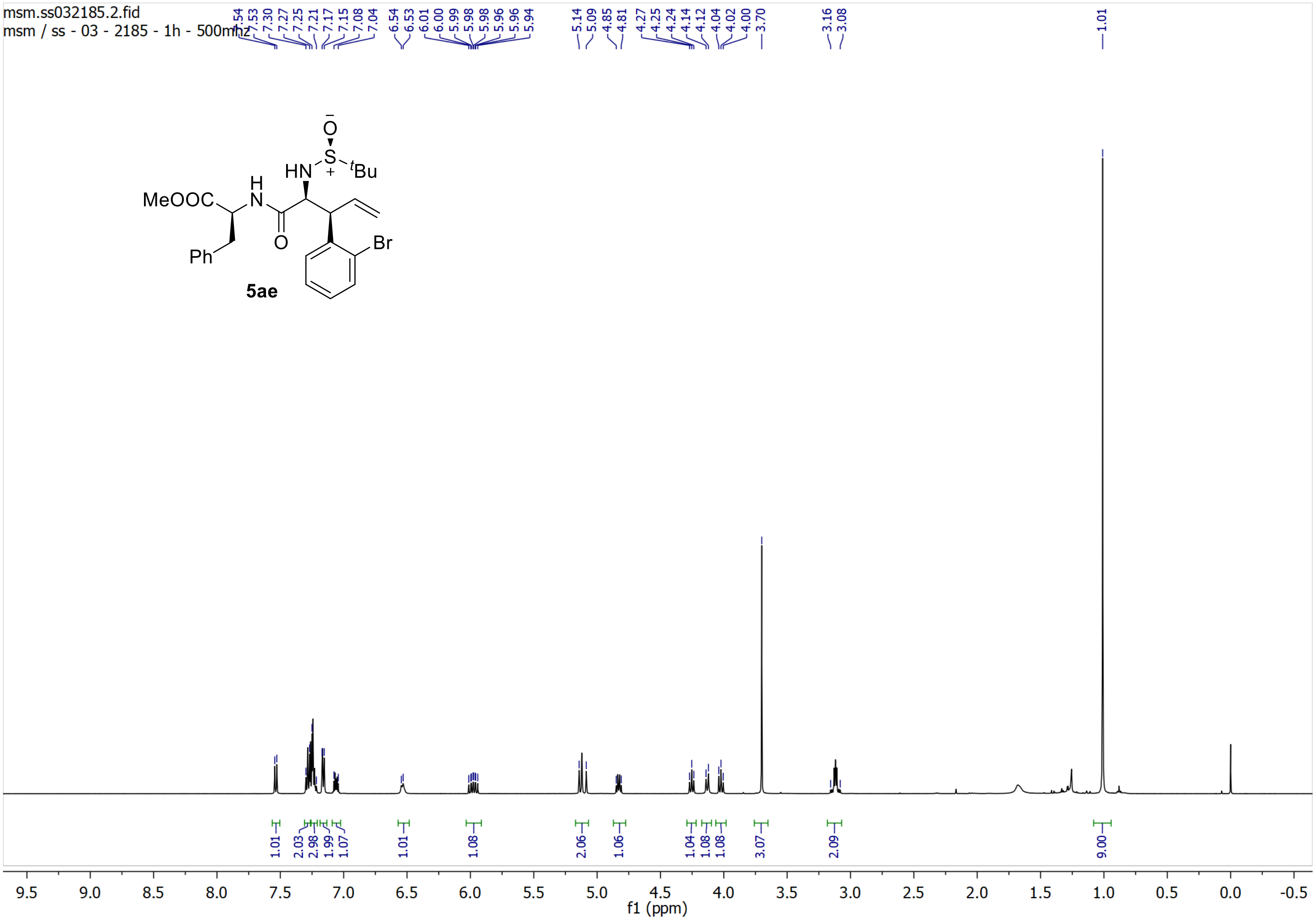
msm.ss032185.2.fid
msm / ss - 03 - 2185 - 1h - 500mhz
MeOOC
HN
tBu
Br
Ph
5ae
f1 (ppm)

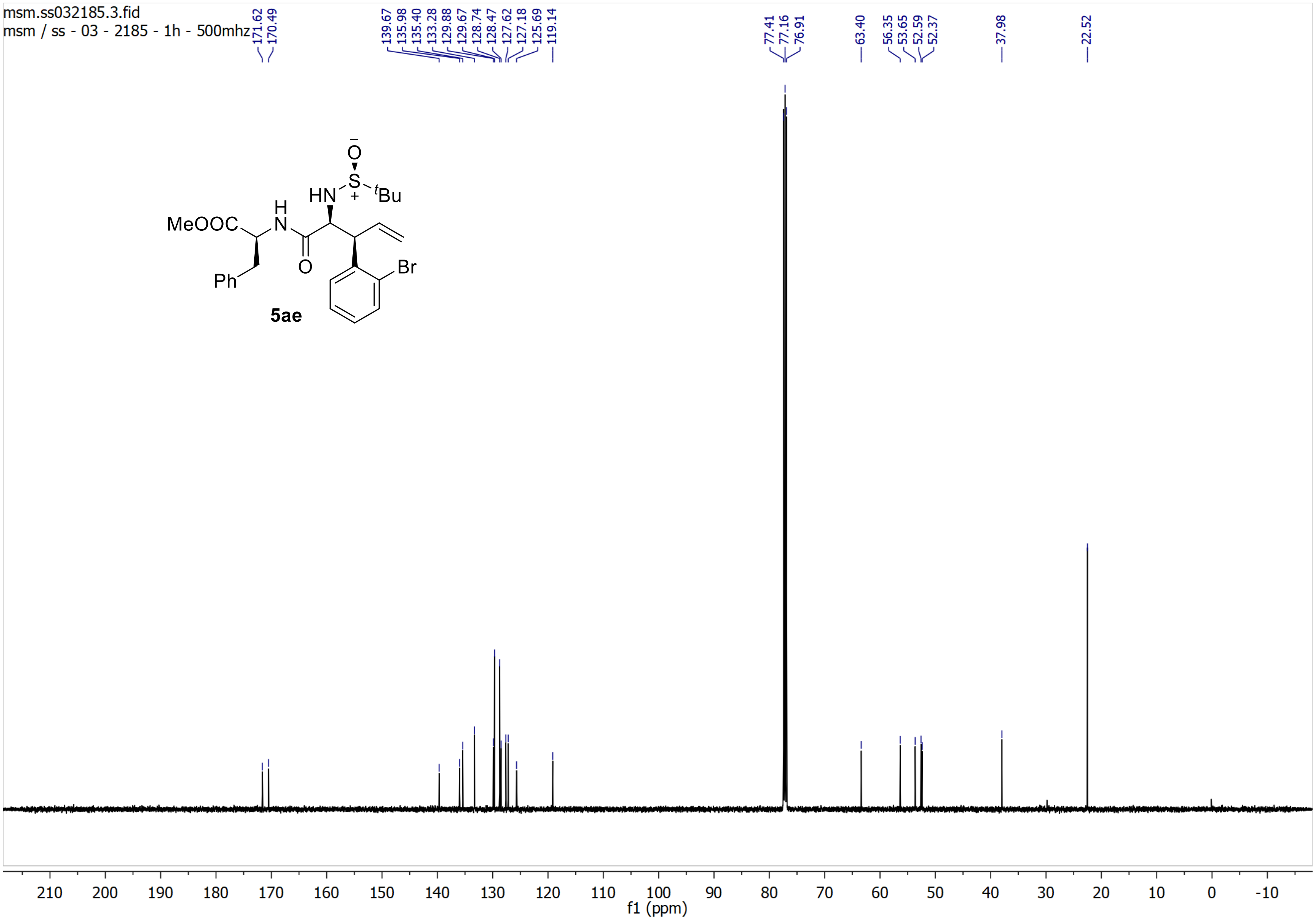

msm.ss032185.3.fid
msm / ss - 03 - 2185 - 1h - 500mhz
171.62
170.49
139.67
135.98
135.40
133.28
129.88
129.67
128.74
128.47
127.62
127.18
125.69
119.14
77.41
77.16
76.91
63.40
56.35
53.65
52.59
52.37
37.98
22.52
MeOOC
H
N
O
HN
S
+
O
tBu
Br
Ph
5ae
210
200
190
180
170
160
150
140
130
120
110
100
90
80
70
60
50
40
30
20
10
0
-10
f1 (ppm)

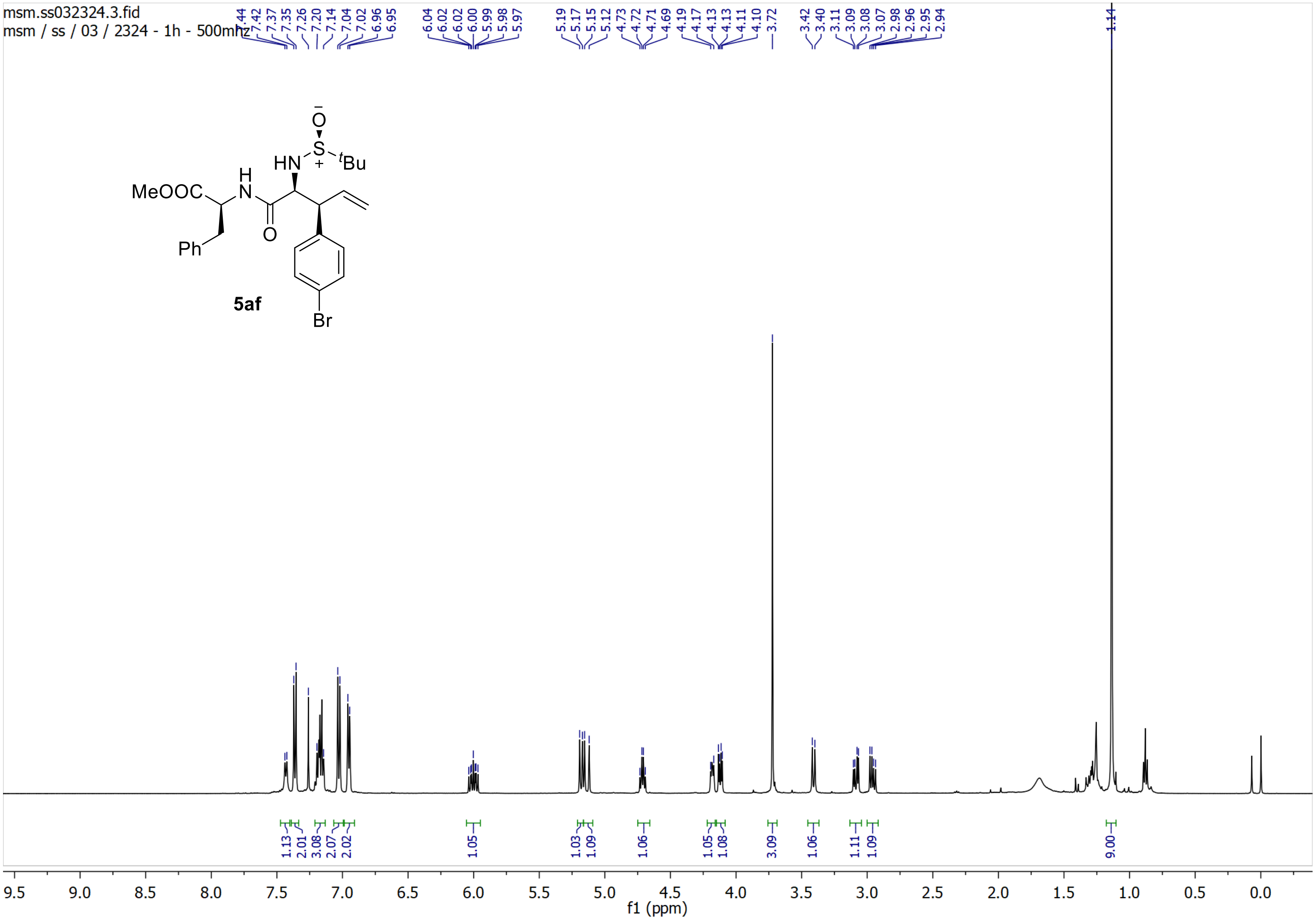
msm.ss032324.3.fid
msm / ss / 03 / 2324 - 1h - 500mhz
5af
f1 (ppm)

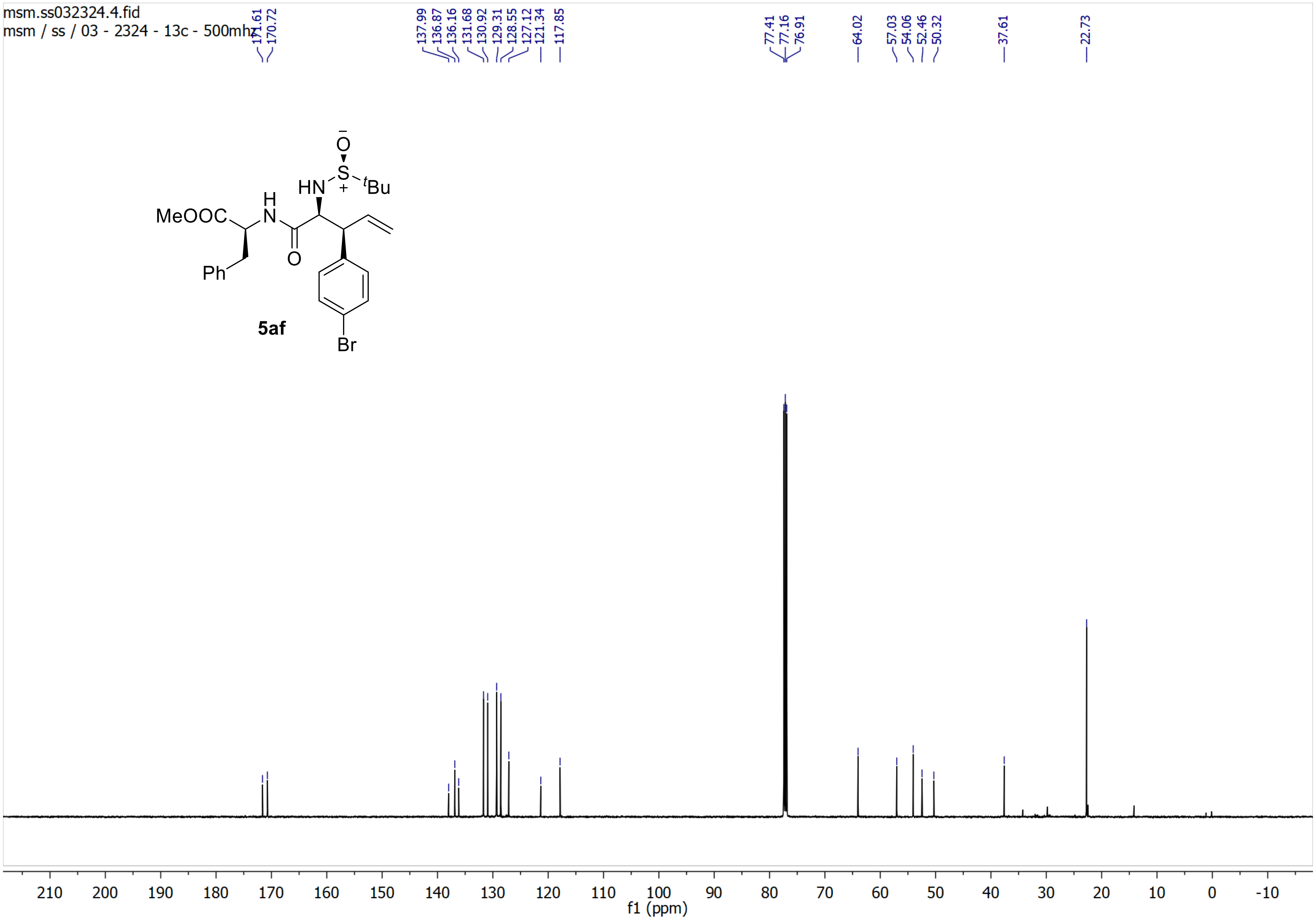
msm.ss032324.4.fid
msm / ss / 03 - 2324 - 13c - 500mhz
171.61
170.72
137.99
136.87
136.16
131.68
130.92
129.31
128.55
127.12
121.34
117.85
77.41
77.16
76.91
64.02
57.03
54.06
52.46
50.32
37.61
22.73
MeOOC
H
N
O
HN
S
+
O
tBu
Ph
Br
5af
210 200 190 180 170 160 150 140 130 120 110 100 90 80 70 60 50 40 30 20 10 0 -10
f1 (ppm)

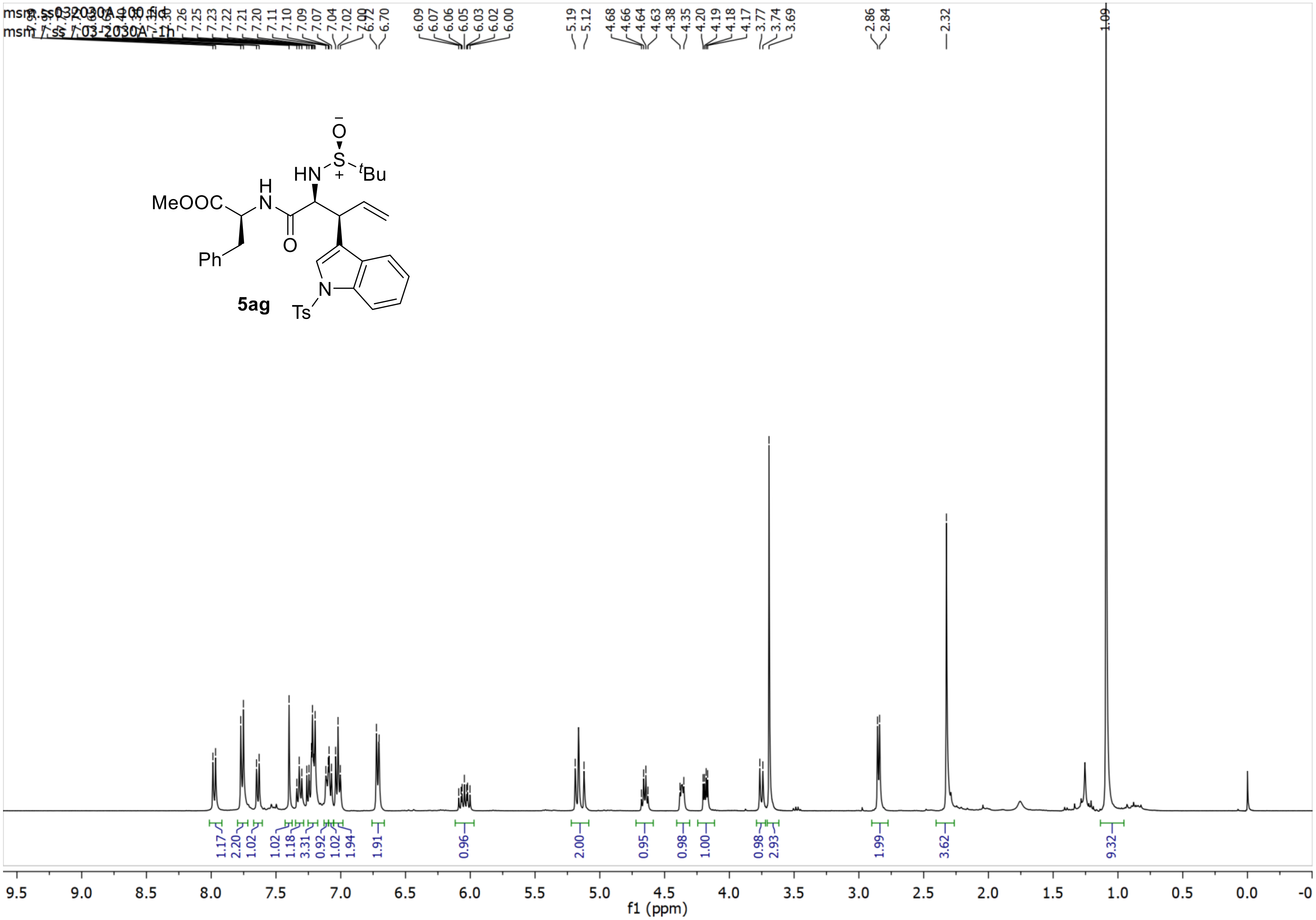

5ag

msmc.ss032030a.100.fid
msm / ss/ 03-2030a - 13c

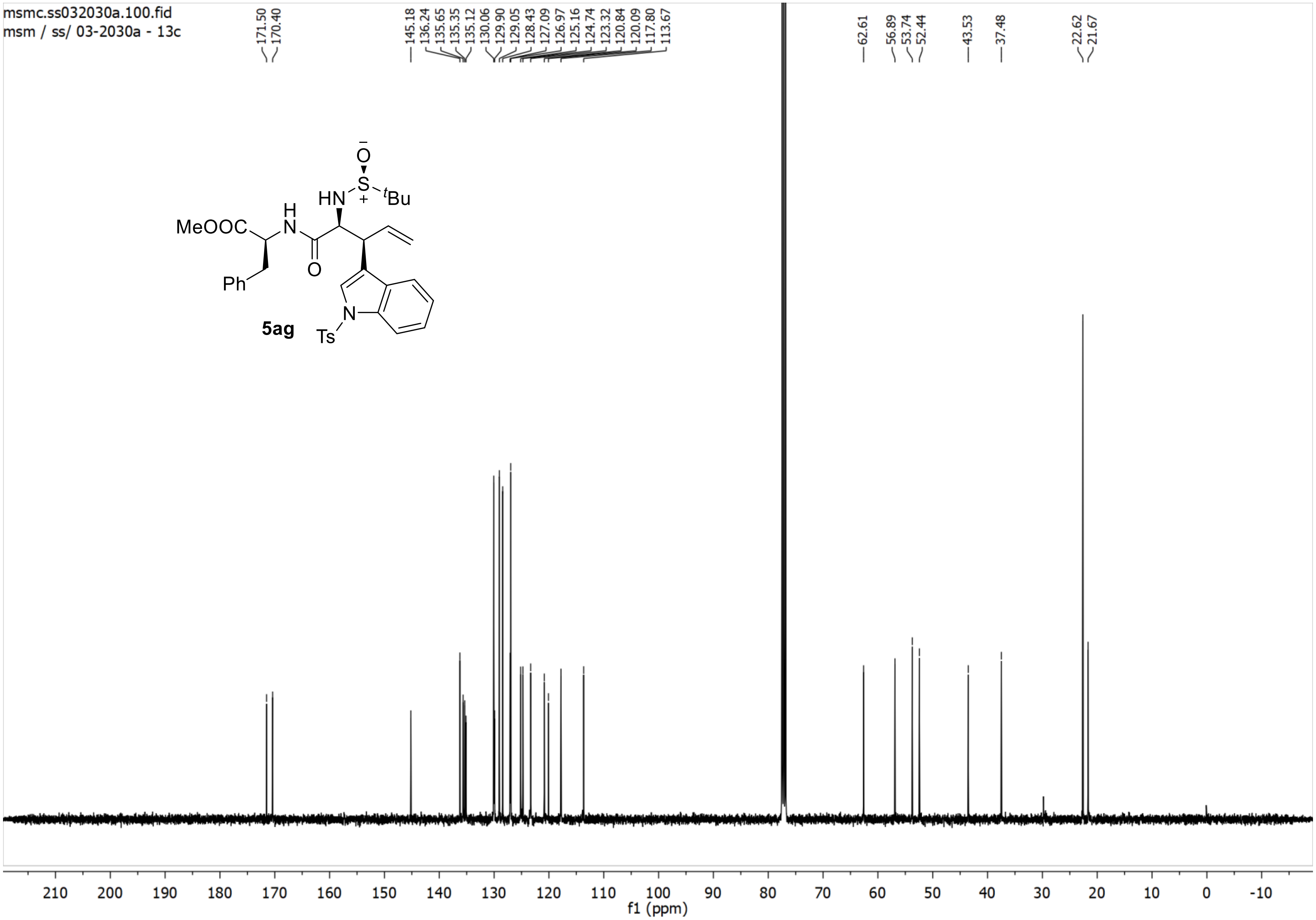

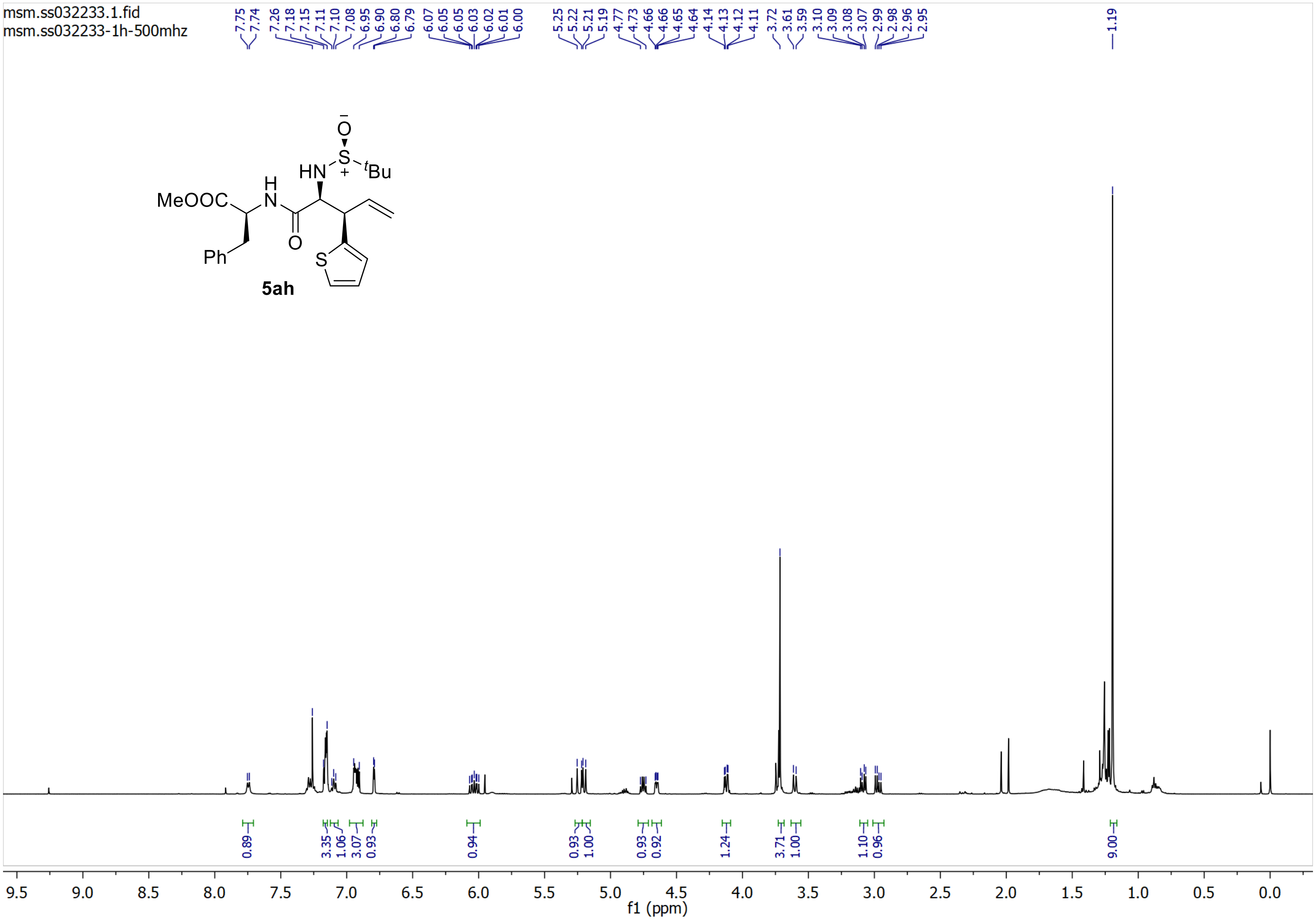

msm.ss032233.1.fid
msm.ss032233-1h-500mhz
7.75 7.74 7.26 7.18 7.15 7.11 7.10 7.08 6.95 6.90 6.80 6.79 6.07 6.05 6.05 6.03 6.02 6.01 6.00 5.25 5.22 5.21 5.19 4.77 4.73 4.66 4.66 4.65 4.64 4.14 4.13 4.12 4.11 3.72 3.61 3.59 3.10 3.09 3.08 3.07 2.99 2.98 2.96 2.95
1.19
MeOOC
H
N
O
HN
S
+
tBu
S
Ph
5ah
0.89 3.35 1.06 3.07 0.93 0.94 0.93 1.00 0.93 0.92 1.24 3.71 1.00 1.10 0.96 9.00
9.5 9.0 8.5 8.0 7.5 7.0 6.5 6.0 5.5 5.0 4.5 4.0 3.5 3.0 2.5 2.0 1.5 1.0 0.5 0.0
f1 (ppm)

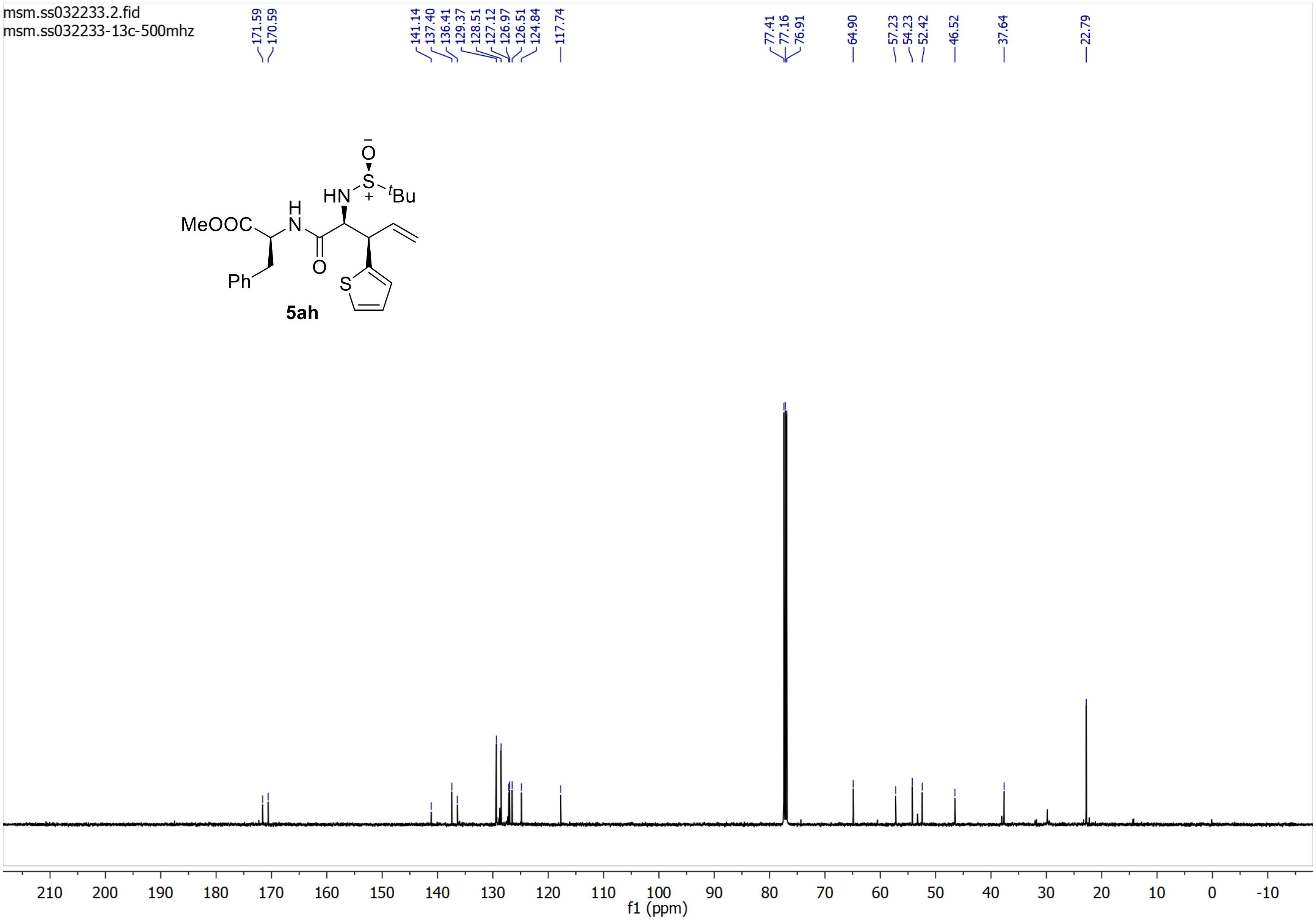

msm.ss032233.2.fid
msm.ss032233-13c-500mhz
171.59
170.59
141.14
137.40
136.41
129.37
128.51
127.12
126.97
126.51
124.84
117.74
77.41
77.16
76.91
64.90
57.23
54.23
52.42
46.52
37.64
22.79
MeOOC
H
N
HN
O
S
+
tBu
O
Ph
S
5ah
210
200
190
180
170
160
150
140
130
120
110
100
90
80
70
60
50
40
30
20
10
0
-10
f1 (ppm)

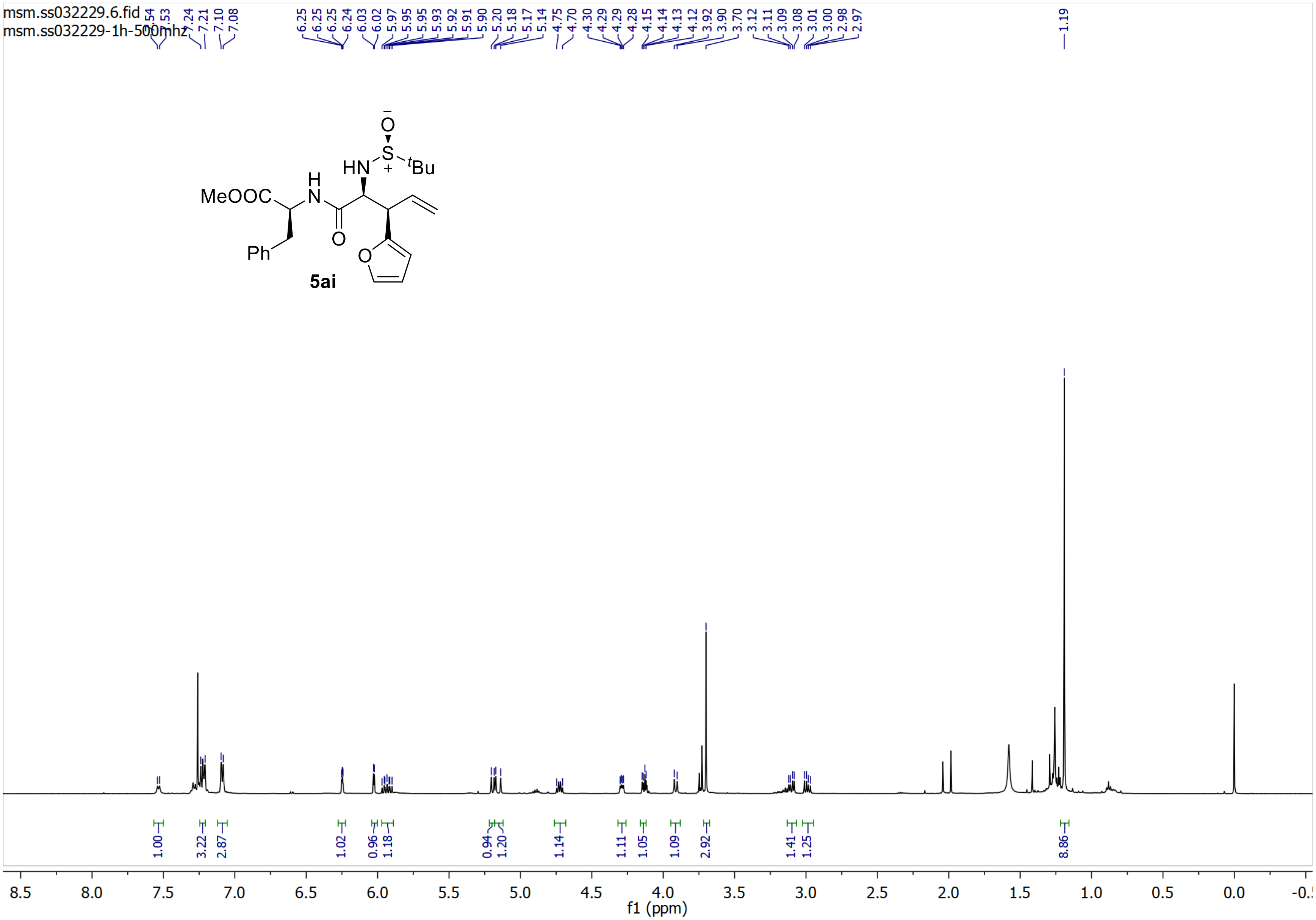
msm.ss032229.6.fid
msm.ss032229-1h-500mhz
7.54 7.53 7.24 7.21 7.10 7.08
6.25 6.25 6.25 6.24 6.03 6.02 5.97 5.95 5.95 5.93 5.92 5.91 5.90 5.20 5.18 5.17 5.14 4.75 4.70 4.30 4.29 4.29 4.28 4.15 4.14 4.13 4.12 3.92 3.90 3.70 3.12 3.11 3.09 3.08 3.01 3.00 2.98 2.97
1.19
MeOOC
H
N
HN
S
O
tBu
O
Ph
O
5ai
1.00 3.22 2.87 1.02 0.96 1.18 0.94 1.20 1.14 1.11 1.05 1.09 2.92 1.41 1.25 8.86
8.5 8.0 7.5 7.0 6.5 6.0 5.5 5.0 4.5 4.0 3.5 3.0 2.5 2.0 1.5 1.0 0.5 0.0
f1 (ppm)

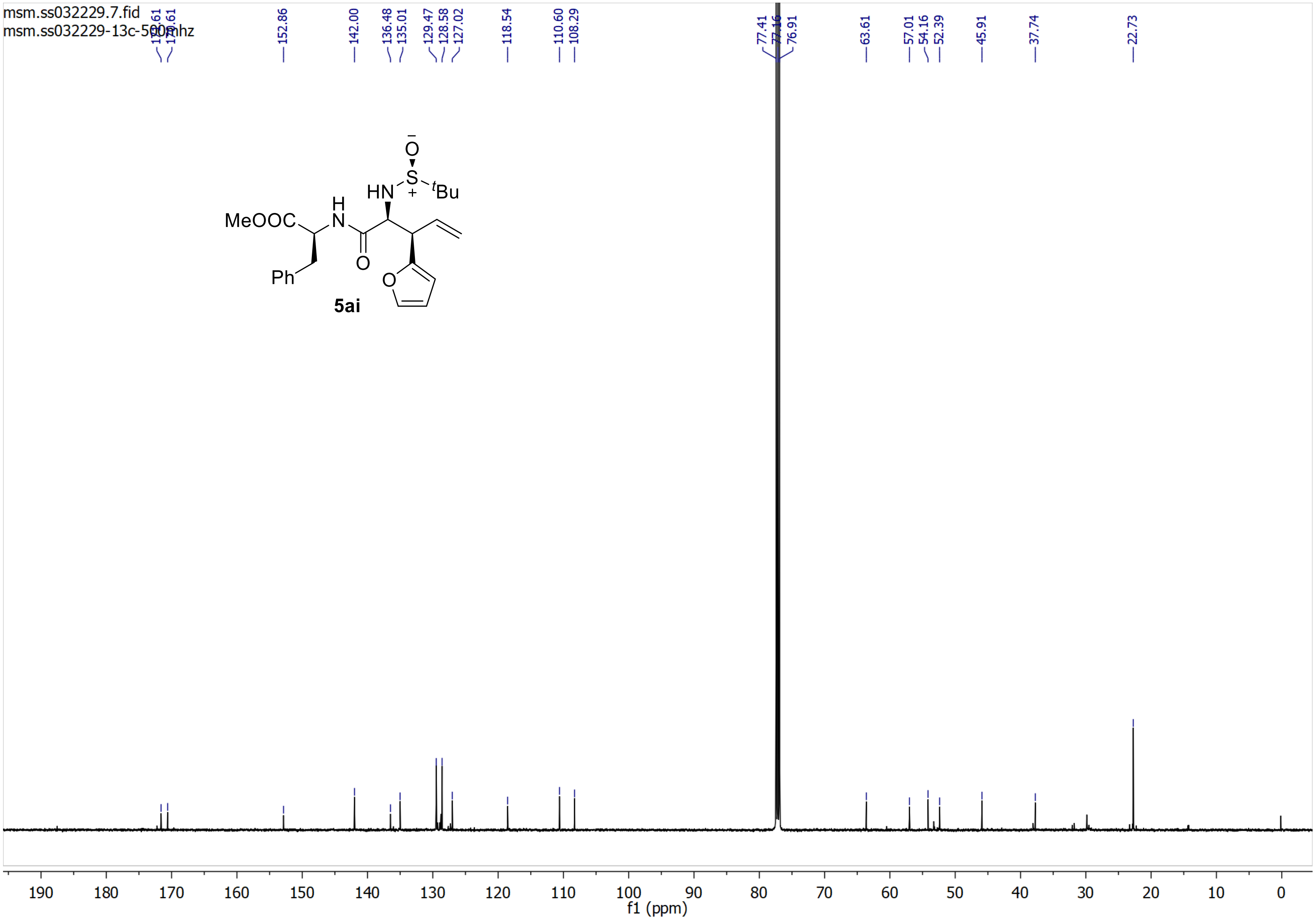
msm.ss032229.7.fid
msm.ss032229-13c-500mhz
152.86
142.00
136.48
135.01
129.47
128.58
127.02
118.54
110.60
108.29
77.41
76.91
63.61
57.01
54.16
52.39
45.91
37.74
22.73
MeOOC
H
N
HN
S
+
O
tBu
O
Ph
O
5ai
190 180 170 160 150 140 130 120 110 100 90 80 70 60 50 40 30 20 10 0
f1 (ppm)

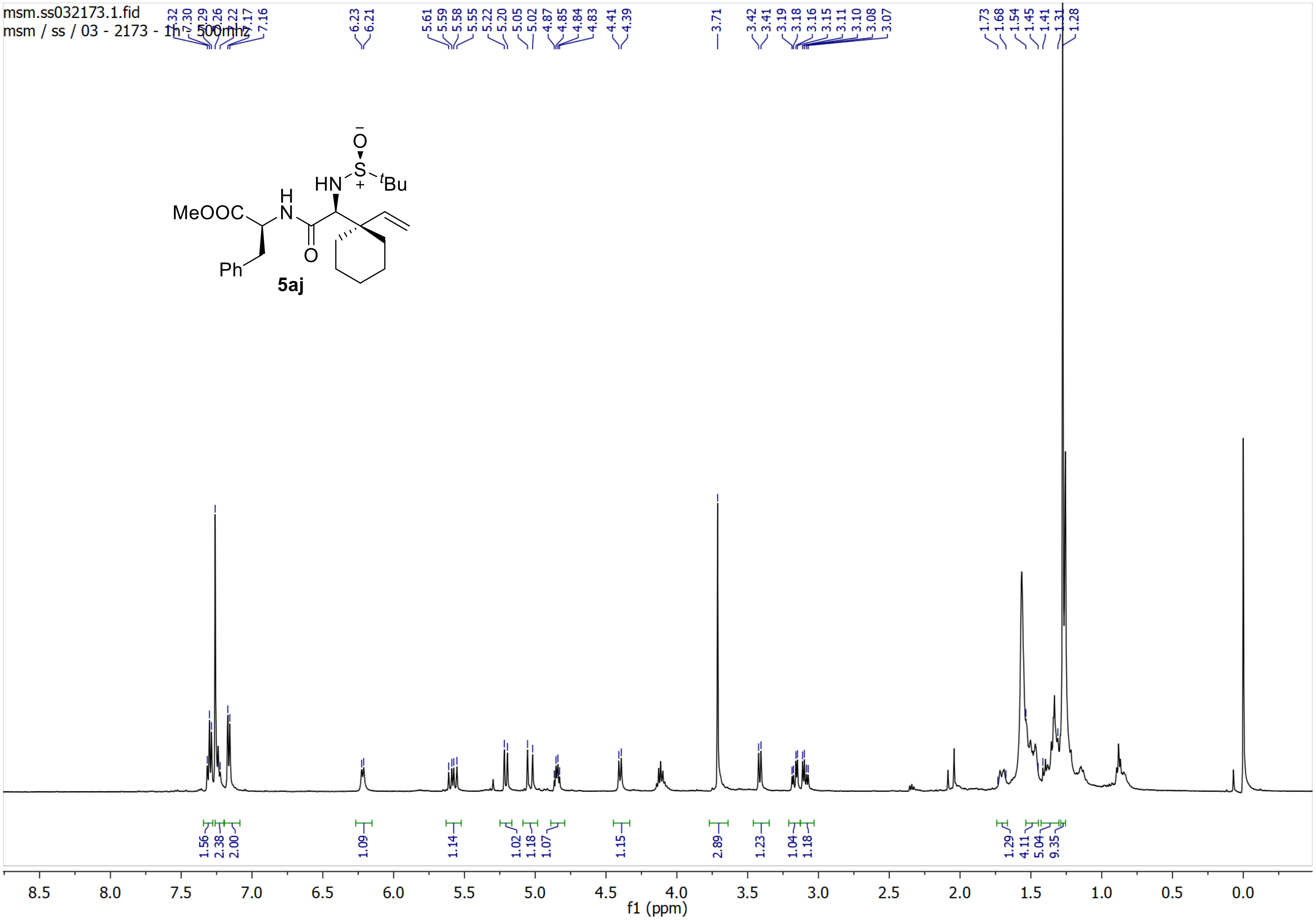

msm.ss032173.1.fid
msm / ss / 03 - 2173 - 1h - 500mhz
MeOOC
H
N
HN
S
O
tBu
O
Ph
5aj
f1 (ppm)

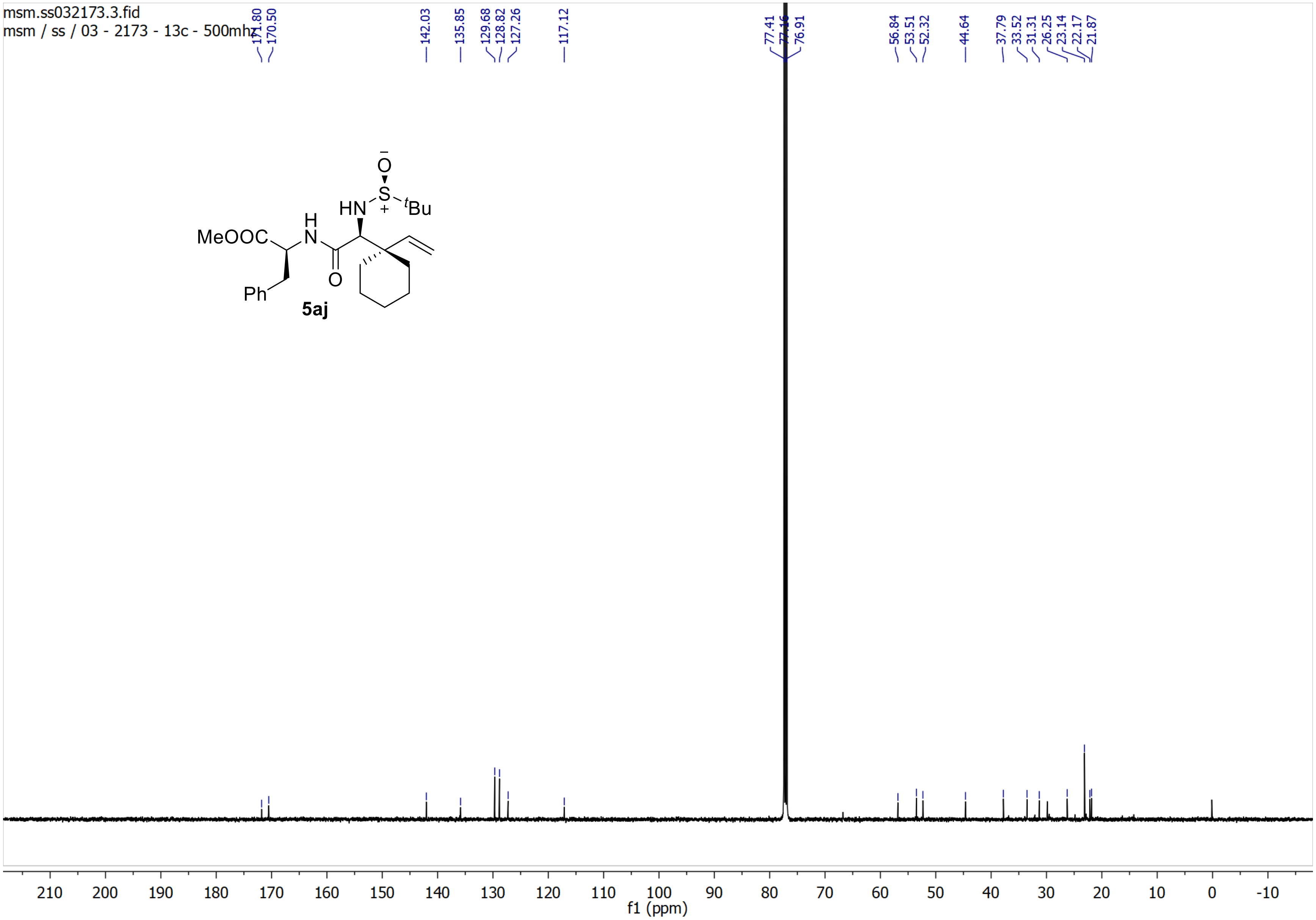
msm.ss032173.3.fid
msm / ss / 03 - 2173 - 13c - 500mhz
171.80
170.50
142.03
135.85
129.68
128.82
127.26
117.12
77.41
77.16
76.91
56.84
53.51
52.32
44.64
37.79
33.52
31.31
26.25
23.14
22.17
21.87
MeOOC
H
N
HN
O
S
tBu
Ph
O
5aj
210 200 190 180 170 160 150 140 130 120 110 100 90 80 70 60 50 40 30 20 10 0 -10
f1 (ppm)

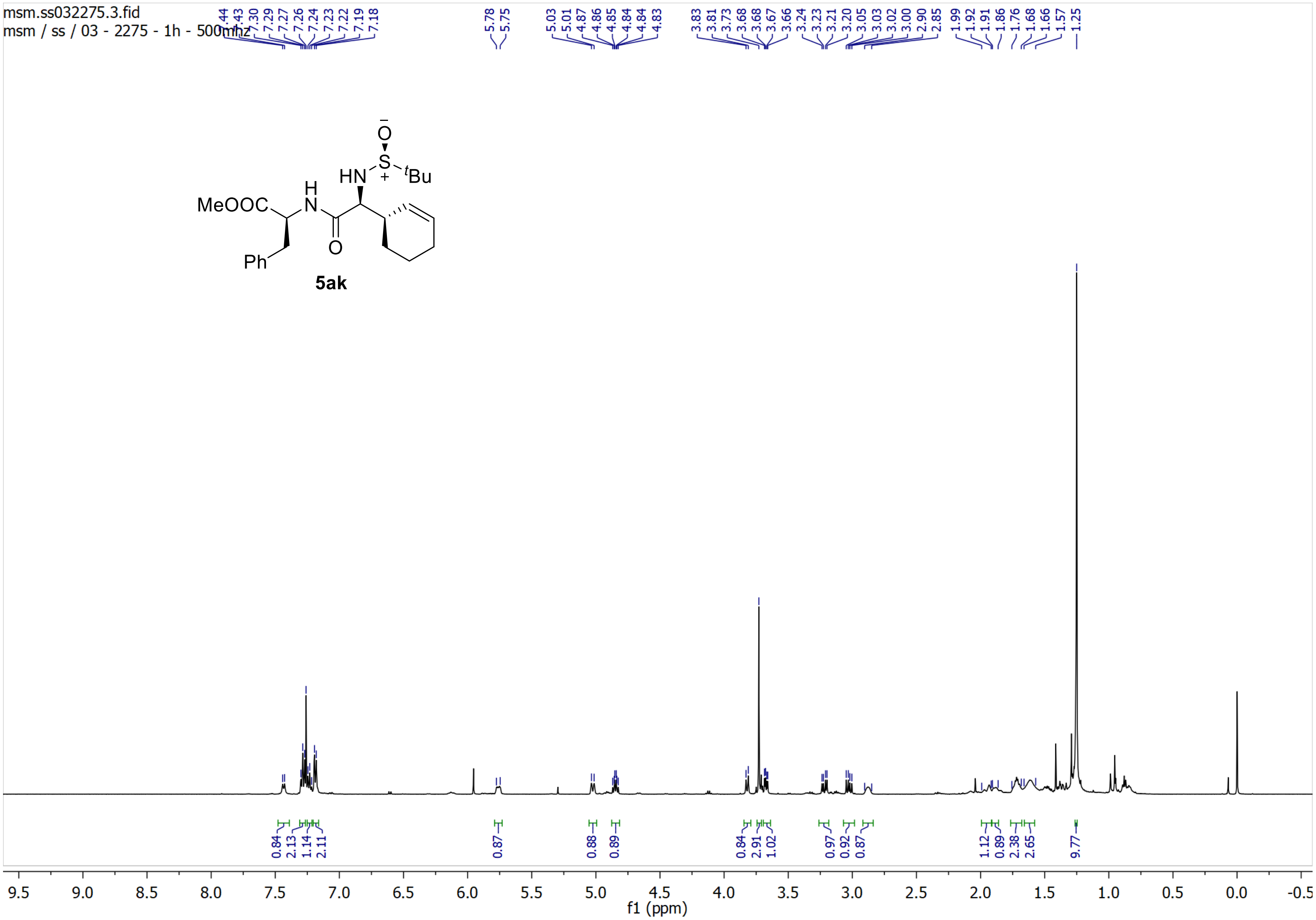
msm.ss032275.3.fid
msm / ss / 03 - 2275 - 1h - 500mhz
7.44 7.43 7.30 7.29 7.27 7.26 7.24 7.23 7.22 7.19 7.18
5.78 5.75
5.03 5.01 4.87 4.86 4.85 4.84 4.84 4.83
3.83 3.81 3.73 3.68 3.68 3.67 3.66 3.24 3.23 3.21 3.20 3.05 3.03 3.02 3.00 2.90 2.85
1.99 1.92 1.91 1.86 1.76 1.68 1.66 1.57 1.25
MeOOC
H
N
O
HN
S
tBu
Ph
5ak
0.84 2.13 1.14 2.11
0.87
0.88 0.89
0.84 2.91 1.02
0.97 0.92 0.87
1.12 0.89 2.38 2.65
9.77
9.5 9.0 8.5 8.0 7.5 7.0 6.5 6.0 5.5 5.0 4.5 4.0 3.5 3.0 2.5 2.0 1.5 1.0 0.5 0.0 -0.5
f1 (ppm)

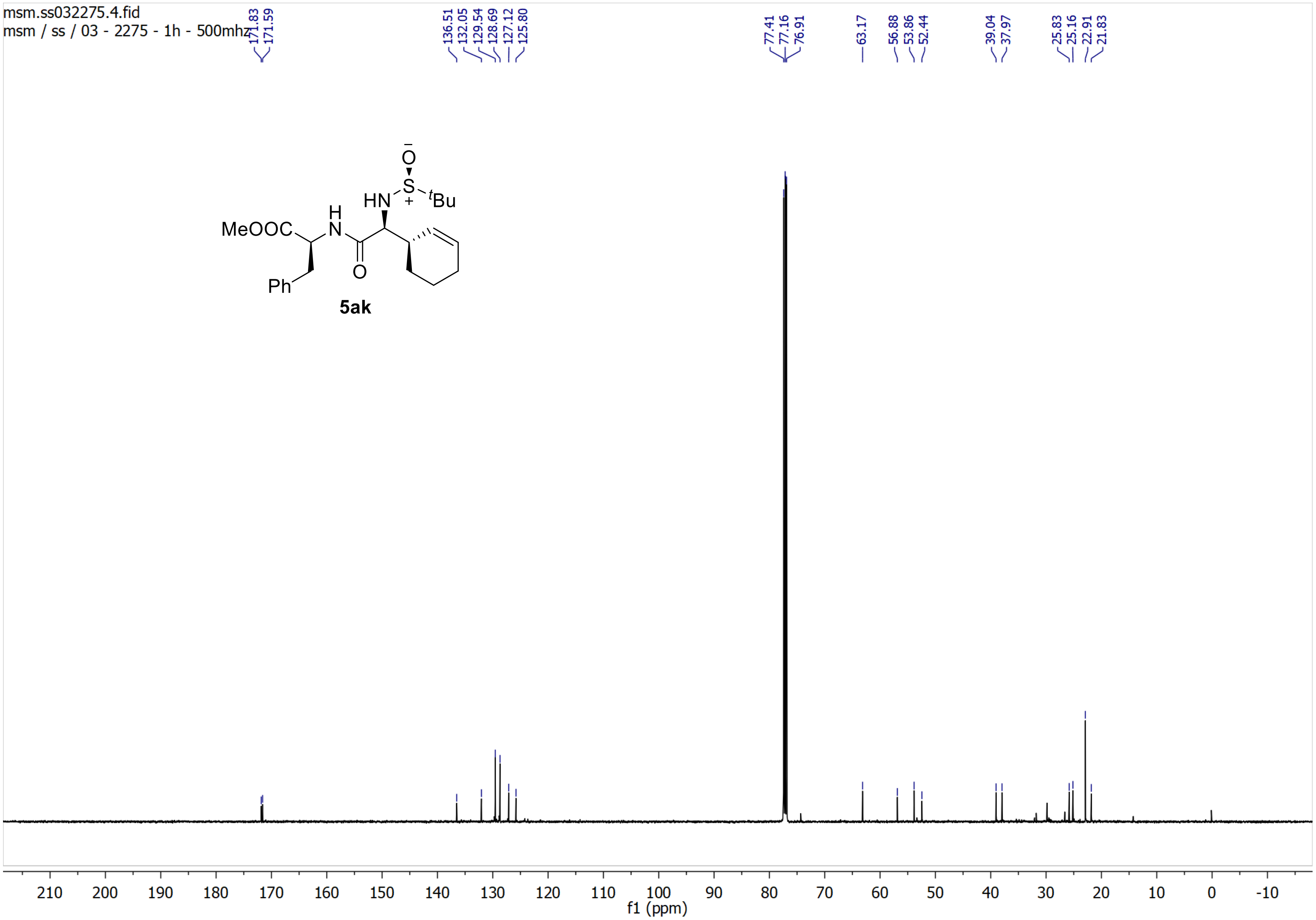

msm.ss032275.4.fid
msm / ss / 03 - 2275 - 1h - 500mhz
171.83
171.59
136.51
132.05
129.54
128.69
127.12
125.80
77.41
77.16
76.91
63.17
56.88
53.86
52.44
39.04
37.97
25.83
25.16
22.91
21.83
MeOOC
H
N
HN
O
S
+
tBu
O
Ph
5ak
210 200 190 180 170 160 150 140 130 120 110 100 90 80 70 60 50 40 30 20 10 0 -10
f1 (ppm)

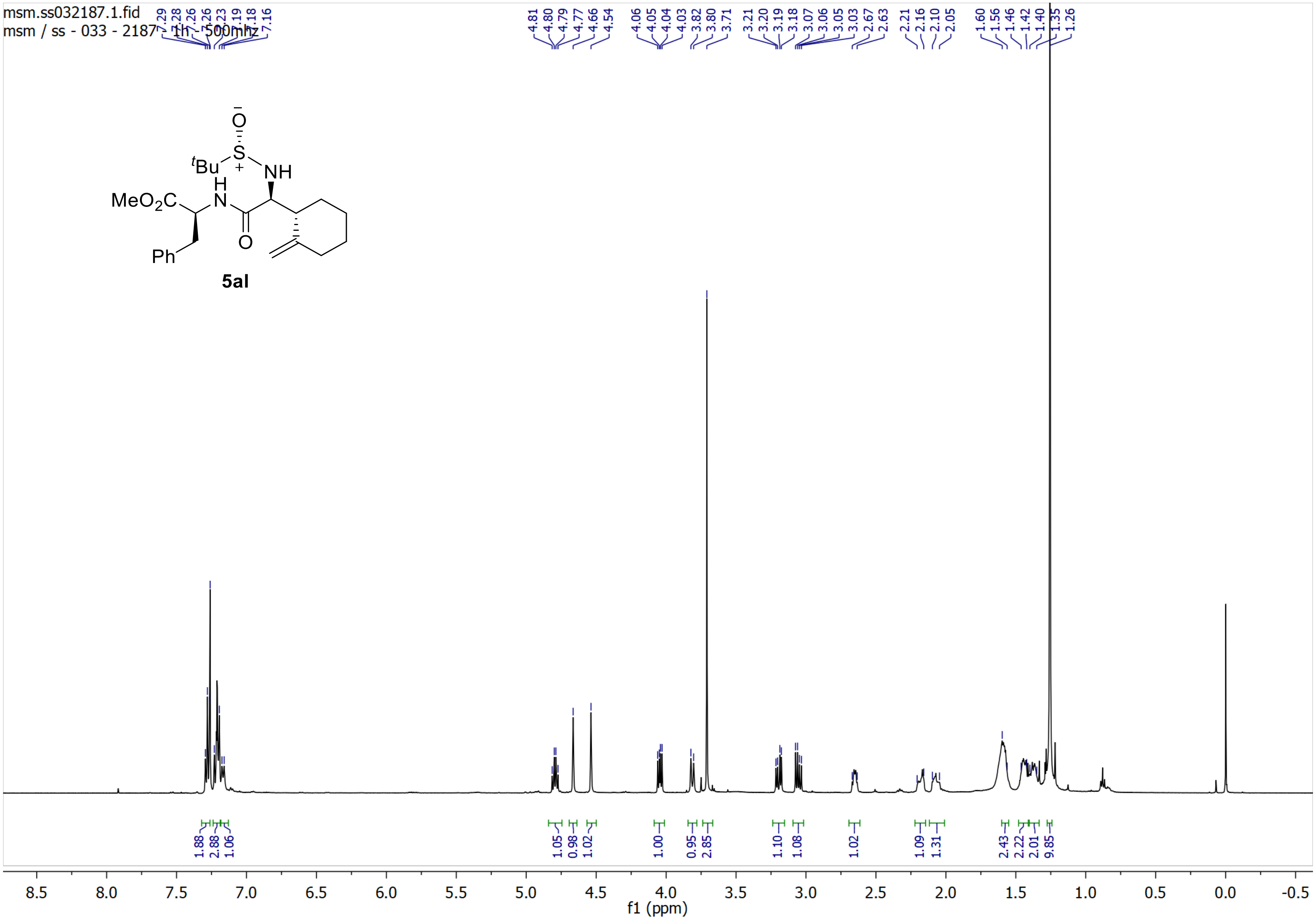
msm.ss032187.1.fid
msm / ss - 033 - 2187 - 1h - 500mhz
5al
f1 (ppm)

msm.ss032187.2.fid
msm / ss - 033 - 2187 - 13c - 500mhz

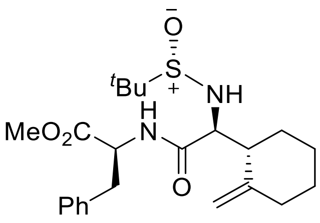

5al

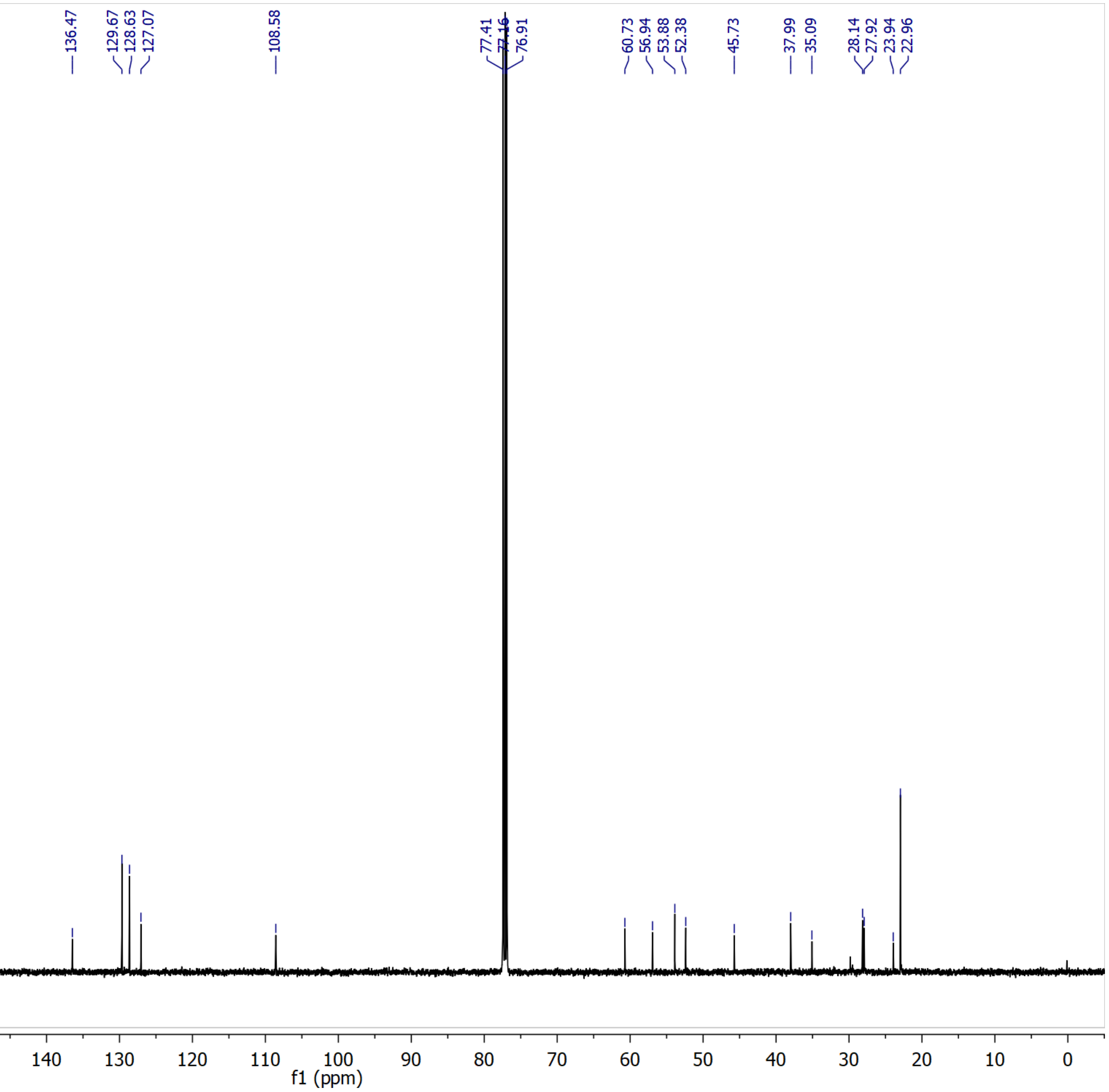

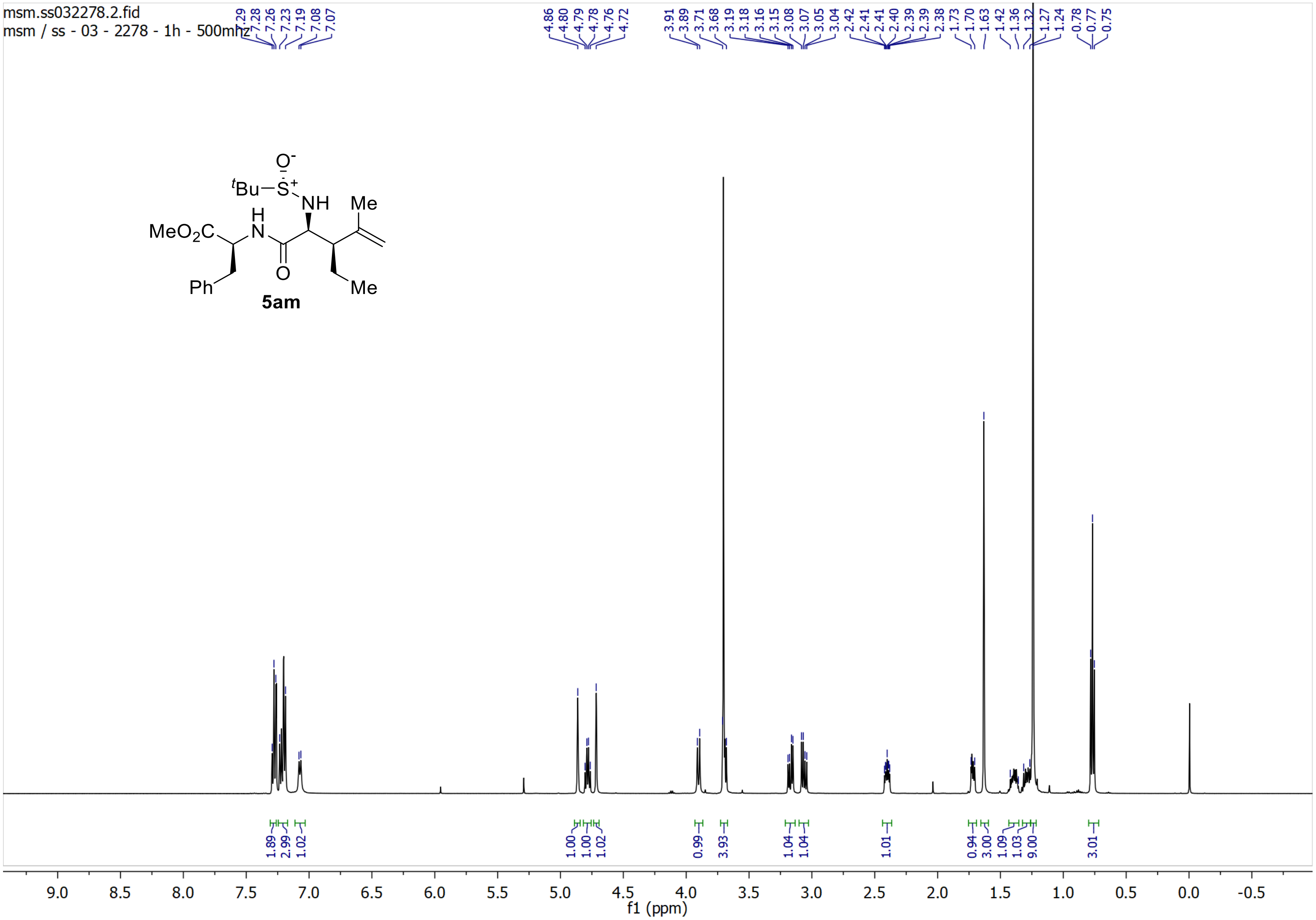
msm.ss032278.2.fid
msm / ss - 03 - 2278 - 1h - 500mhz
5am
f1 (ppm)

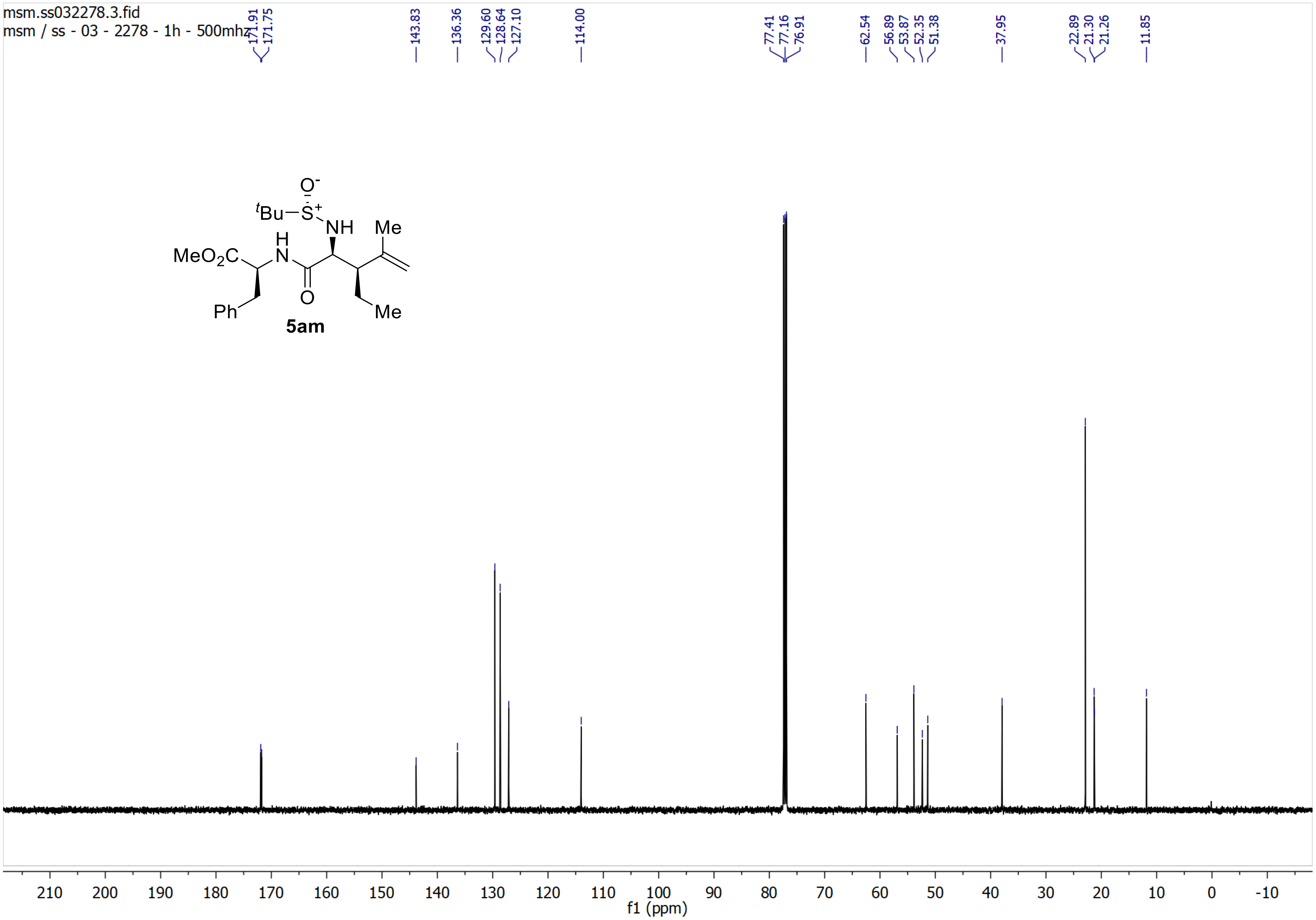

msm.ss032278.3.fid
msm / ss - 03 - 2278 - 1h - 500mhz
171.91
171.75
143.83
136.36
129.60
128.64
127.10
114.00
77.41
77.16
76.91
62.54
56.89
53.87
52.35
51.38
37.95
22.89
21.30
21.26
11.85
5am
210 200 190 180 170 160 150 140 130 120 110 100 90 80 70 60 50 40 30 20 10 0 -10
f1 (ppm)

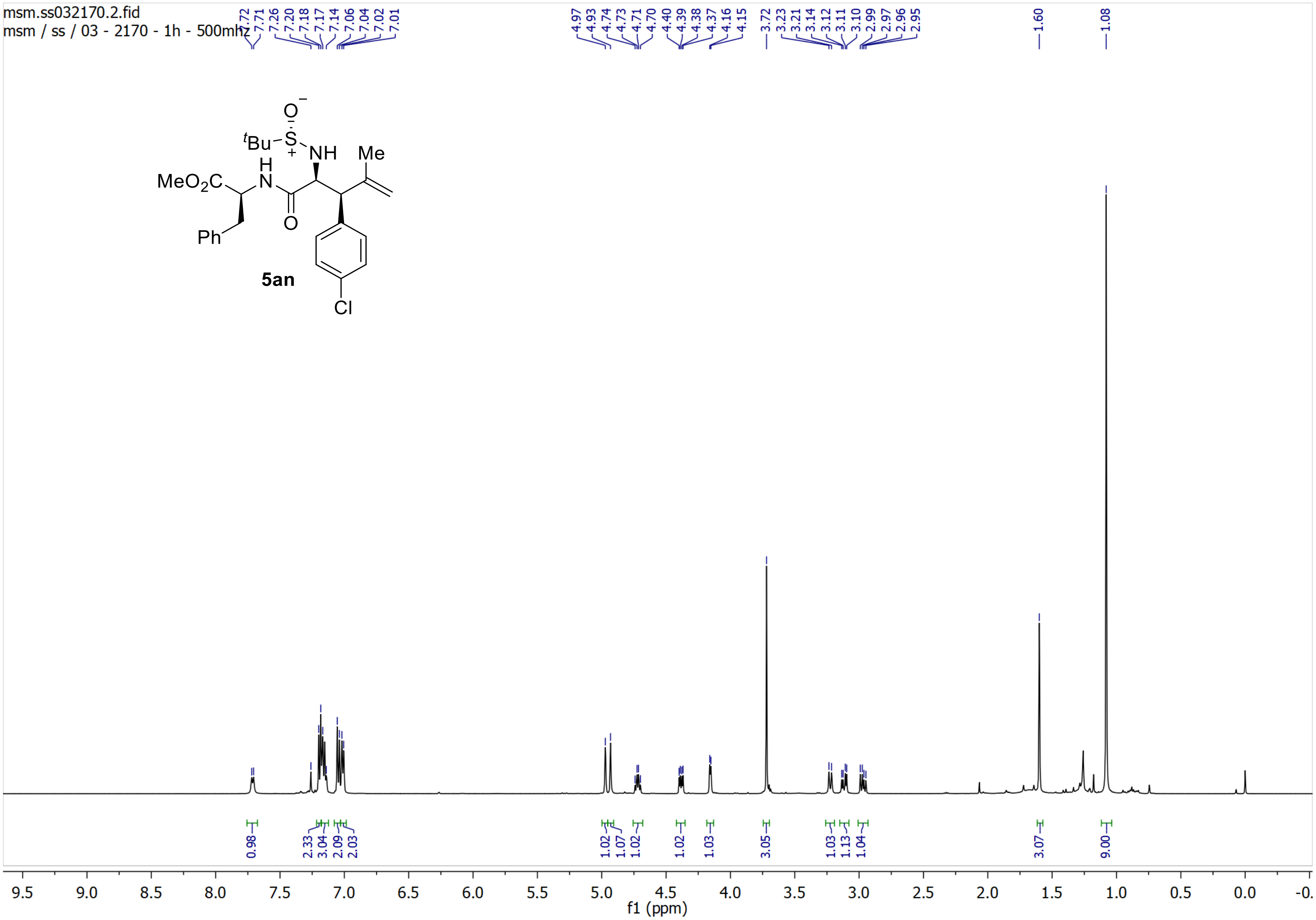
msm.ss032170.2.fid
msm / ss / 03 - 2170 - 1h - 500mhz
7.72 7.71 7.26 7.20 7.18 7.17 7.14 7.06 7.04 7.02 7.01
4.97 4.93 4.74 4.73 4.71 4.70 4.40 4.39 4.38 4.37 4.16 4.15
3.72 3.23 3.21 3.14 3.12 3.11 3.10 2.99 2.97 2.96 2.95
1.60
1.08
tBu
NH
Me
MeO2C
H
N
O
Ph
5an
Cl
0.98 2.33 3.04 2.09 2.03 1.02 1.07 1.02 1.02 1.03 3.05 1.03 1.13 1.04 3.07 9.00
f1 (ppm)

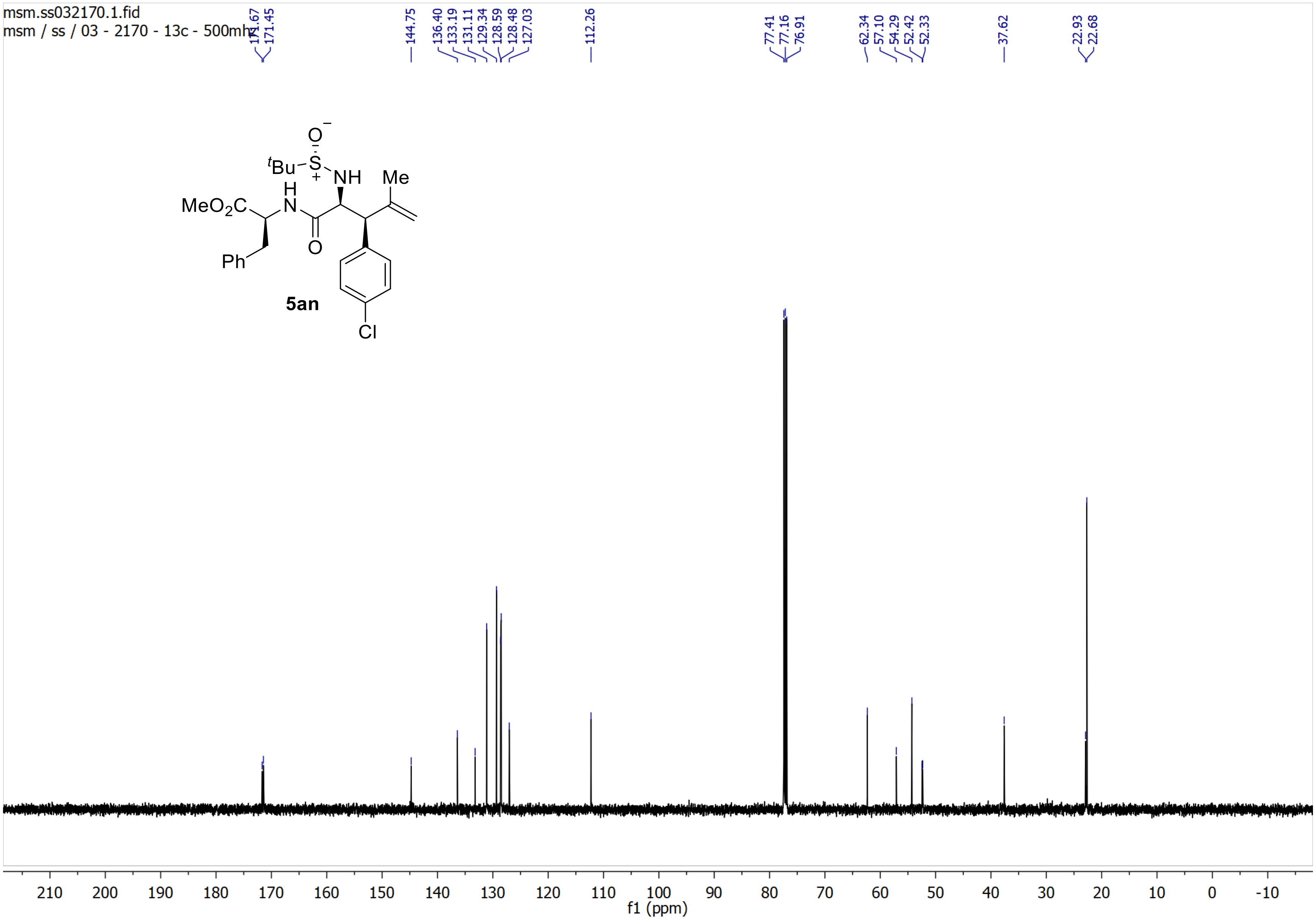
msm.ss032170.1.fid
msm / ss / 03 - 2170 - 13c - 500mhz
171.67
171.45
144.75
136.40
133.19
131.11
129.34
128.59
128.48
127.03
112.26
77.41
77.16
76.91
62.34
57.10
54.29
52.42
52.33
37.62
22.93
22.68
5an
f1 (ppm)

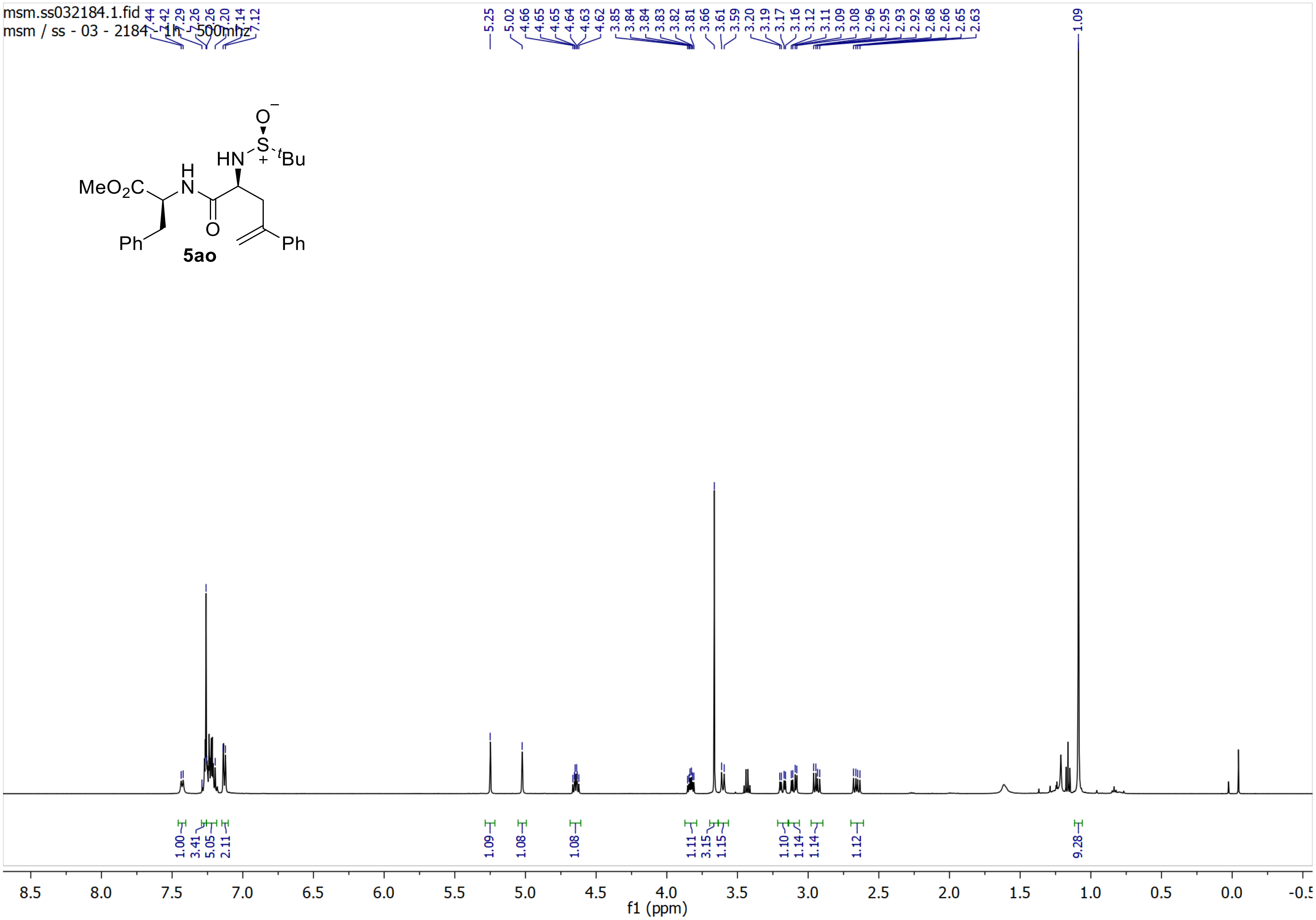

msm.ss032184.1.fid
msm / ss - 03 - 2184 - 1h - 500mhz
7.44
7.42
7.29
7.26
7.26
7.20
7.14
7.12
5.25
5.02
4.66
4.65
4.65
4.64
4.63
4.62
3.85
3.84
3.84
3.83
3.82
3.81
3.66
3.61
3.59
3.20
3.19
3.17
3.16
3.12
3.11
3.09
3.08
2.96
2.95
2.93
2.92
2.68
2.66
2.65
2.63
1.09
MeO2C
H
N
HN
O
S
+
tBu
O
Ph
Ph
5ao
1.00
3.41
5.05
2.11
1.09
1.08
1.08
1.11
3.15
1.15
1.10
1.14
1.14
1.12
9.28
8.5
8.0
7.5
7.0
6.5
6.0
5.5
5.0
4.5
4.0
3.5
3.0
2.5
2.0
1.5
1.0
0.5
0.0
-0.5
f1 (ppm)

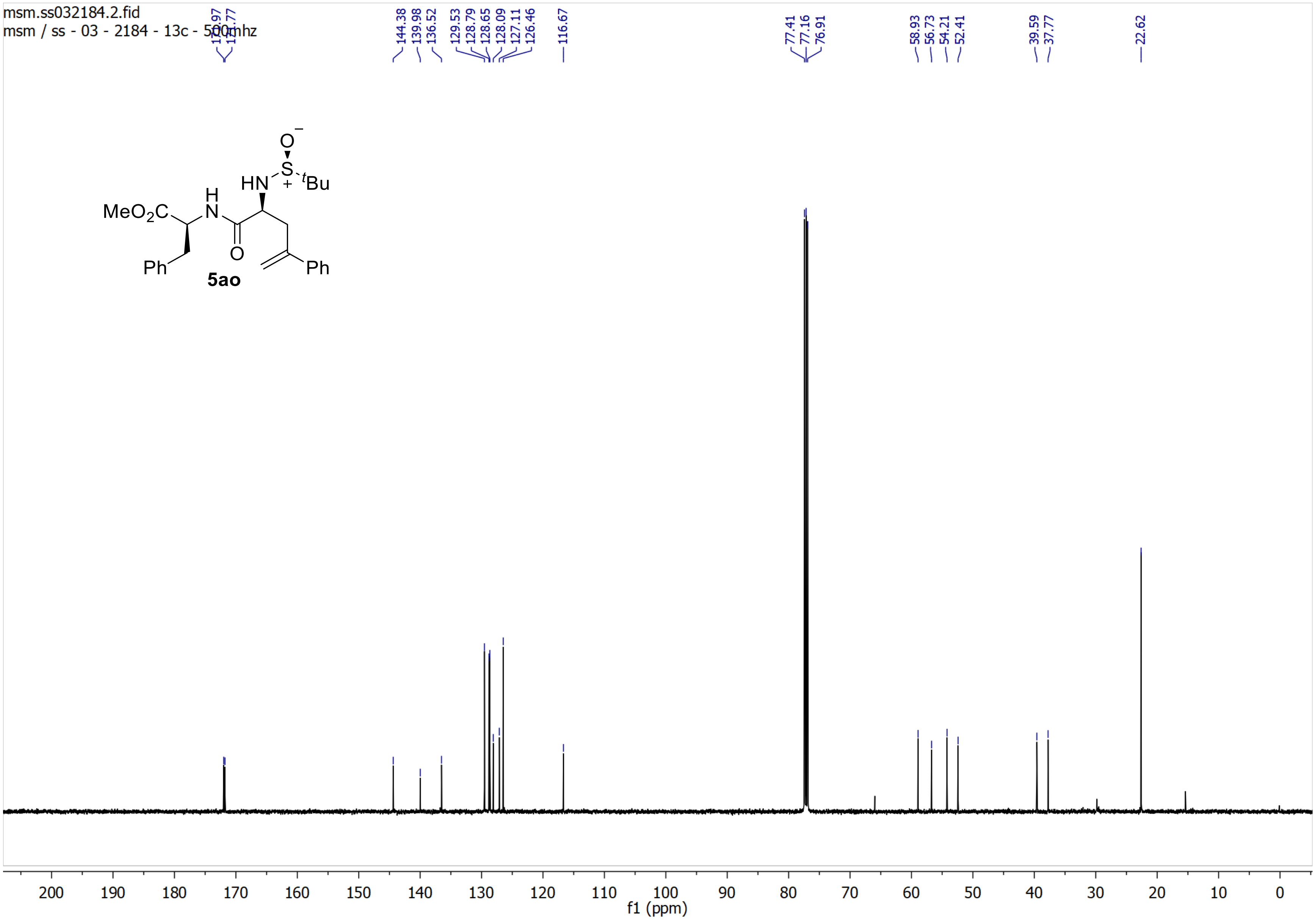
msm.ss032184.2.fid
msm / ss - 03 - 2184 - 13c - 500mhz
144.38
139.98
136.52
129.53
128.79
128.65
128.09
127.11
126.46
116.67
77.41
77.16
76.91
58.93
56.73
54.21
52.41
39.59
37.77
22.62
MeO2C
H
N
HN
O
S
+
tBu
O
Ph
Ph
5ao
200
190
180
170
160
150
140
130
120
110
100
90
80
70
60
50
40
30
20
10
0
f1 (ppm)

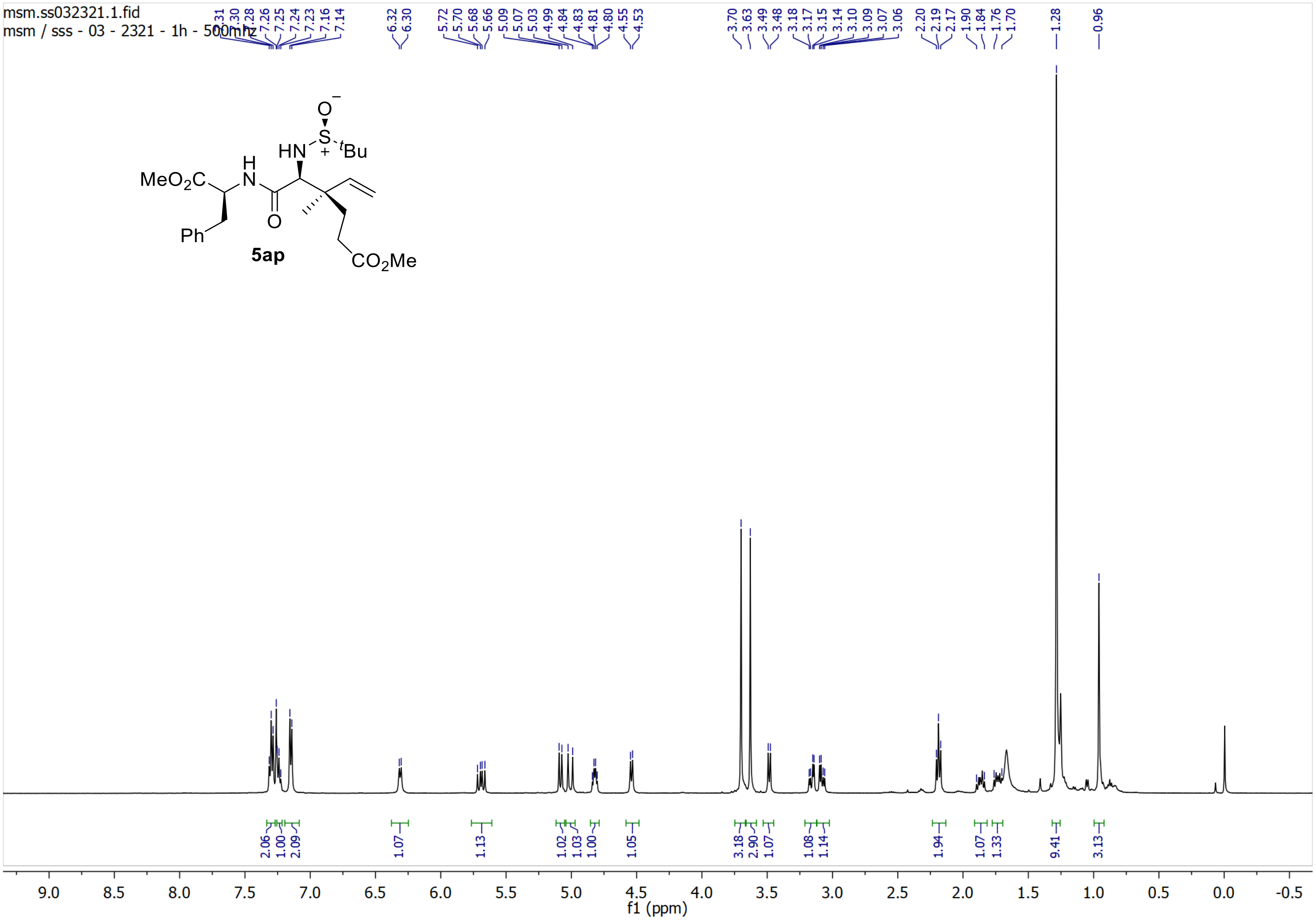
msm.ss032321.1.fid
msm / sss - 03 - 2321 - 1h - 500mhz
7.31 7.30 7.28 7.26 7.25 7.24 7.23 7.16 7.14
6.32 6.30
5.72 5.70 5.68 5.66 5.09 5.07 5.03 4.99 4.84 4.83 4.81 4.80 4.55 4.53
3.70 3.63 3.49 3.48 3.18 3.17 3.15 3.14 3.10 3.09 3.07 3.06
2.20 2.19 2.17 1.90 1.84 1.76 1.70
1.28
0.96
MeO2C
H
N
HN
S
O
tBu
O
Ph
5ap
CO2Me
2.06 1.00 2.09 1.07 1.13 1.02 1.03 1.00 1.05 3.18 2.90 1.07 1.08 1.14 1.94 1.07 1.33 9.41 3.13
9.0 8.5 8.0 7.5 7.0 6.5 6.0 5.5 5.0 4.5 4.0 3.5 3.0 2.5 2.0 1.5 1.0 0.5 0.0 -0.5
f1 (ppm)

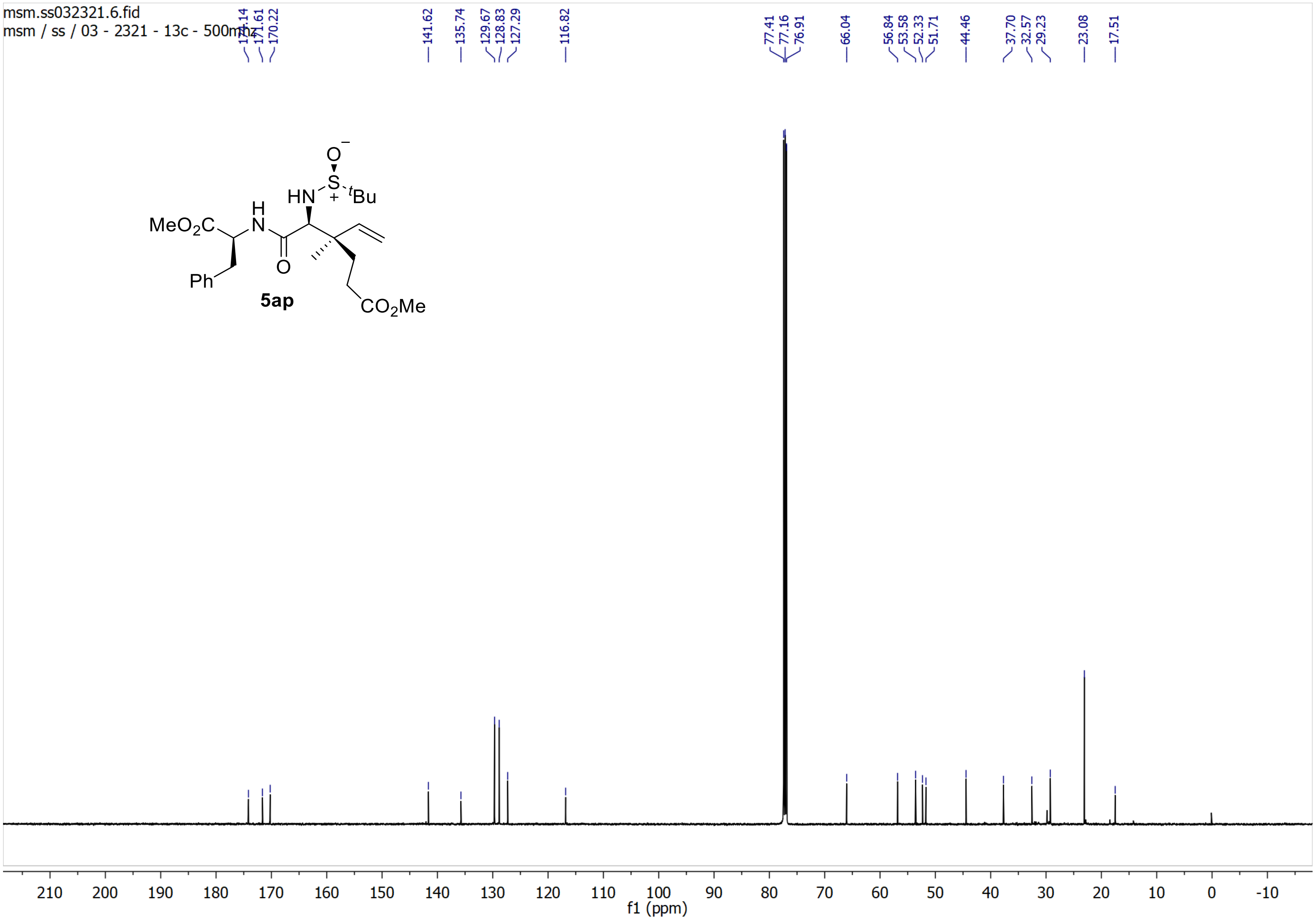

msm.ss032321.6.fid
msm / ss / 03 - 2321 - 13c - 500mhz
174.14
171.61
170.22
141.62
135.74
129.67
128.83
127.29
116.82
77.41
77.16
76.91
66.04
56.84
53.58
52.33
51.71
44.46
37.70
32.57
29.23
23.08
17.51
MeO2C
H
N
O
HN
S
tBu
Ph
CO2Me
5ap
210 200 190 180 170 160 150 140 130 120 110 100 90 80 70 60 50 40 30 20 10 0 -10
f1 (ppm)

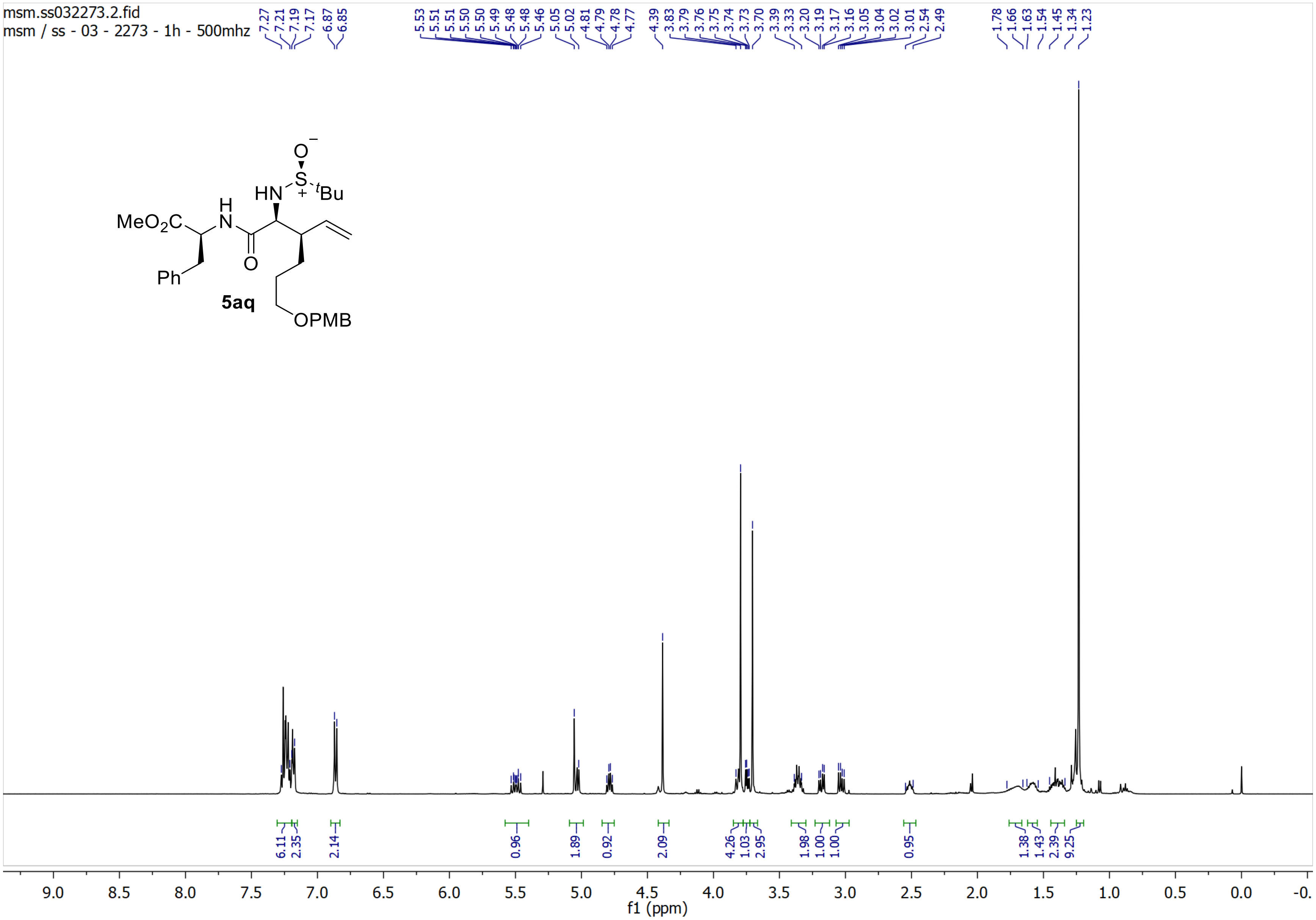
msm.ss032273.2.fid
msm / ss - 03 - 2273 - 1h - 500mhz
7.27
7.21
7.19
7.17
6.87
6.85
5.53
5.51
5.51
5.50
5.50
5.49
5.48
5.48
5.46
5.05
5.02
4.81
4.79
4.78
4.77
4.39
3.83
3.79
3.76
3.75
3.74
3.73
3.70
3.39
3.33
3.20
3.19
3.17
3.16
3.05
3.04
3.02
3.01
2.54
2.49
1.78
1.66
1.63
1.54
1.45
1.34
1.23
MeO2C
H
N
HN
O
S
+
tBu
O
Ph
5aq
OPMB
6.11
2.35
2.14
0.96
1.89
0.92
2.09
4.26
1.03
2.95
1.98
1.00
1.00
0.95
1.38
1.43
2.39
9.25
9.0
8.5
8.0
7.5
7.0
6.5
6.0
5.5
5.0
4.5
4.0
3.5
3.0
2.5
2.0
1.5
1.0
0.5
0.0
-0.
f1 (ppm)

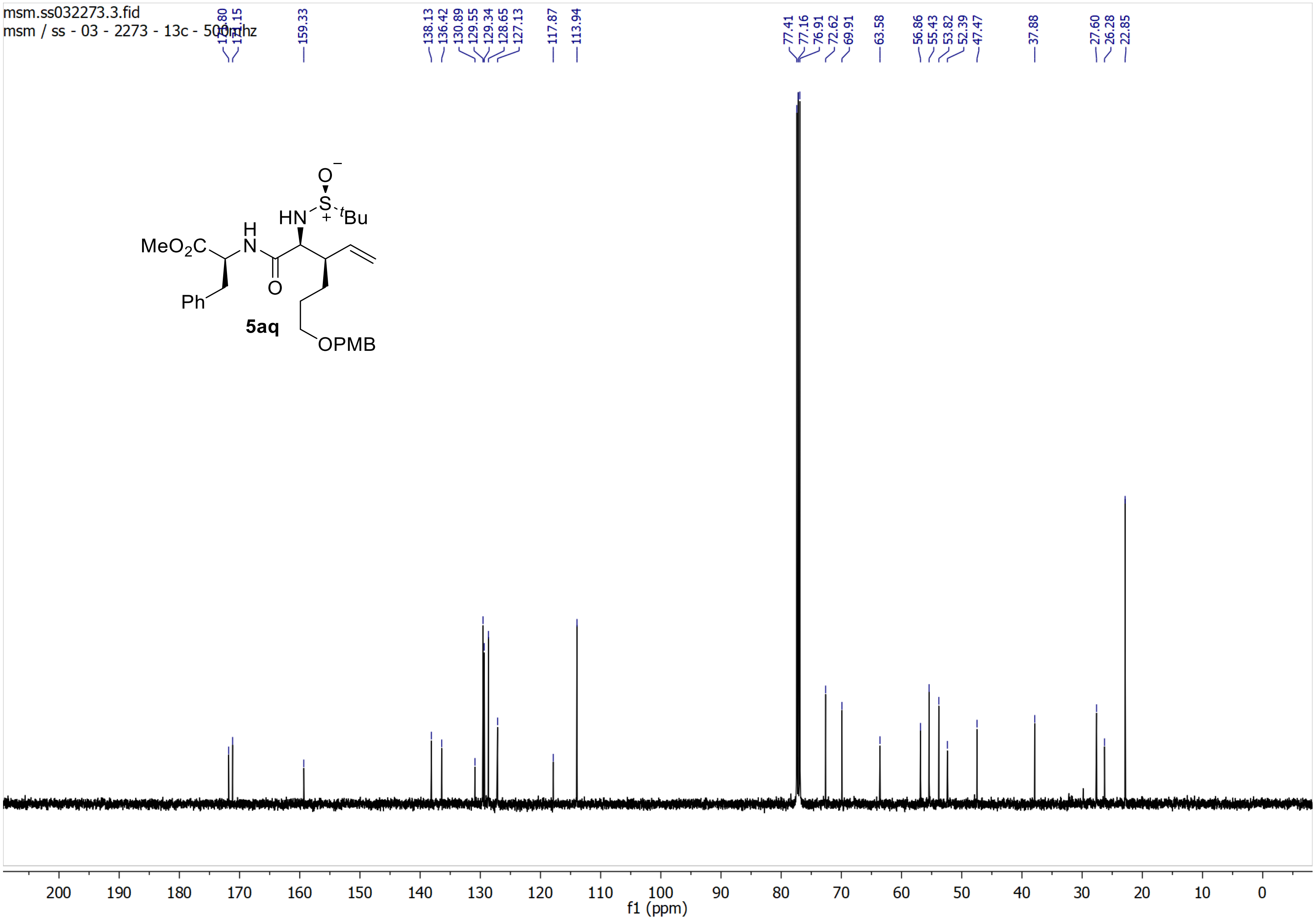
msm.ss032273.3.fid
msm / ss - 03 - 2273 - 13c - 500mhz
159.33
138.13
136.42
130.89
129.55
129.34
128.65
127.13
117.87
113.94
77.41
77.16
76.91
72.62
69.91
63.58
56.86
55.43
53.82
52.39
47.47
37.88
27.60
26.28
22.85
MeO2C
H
N
HN
S
+
O
−
tBu
O
Ph
5aq
OPMB
200
190
180
170
160
150
140
130
120
110
100
90
80
70
60
50
40
30
20
10
0
f1 (ppm)

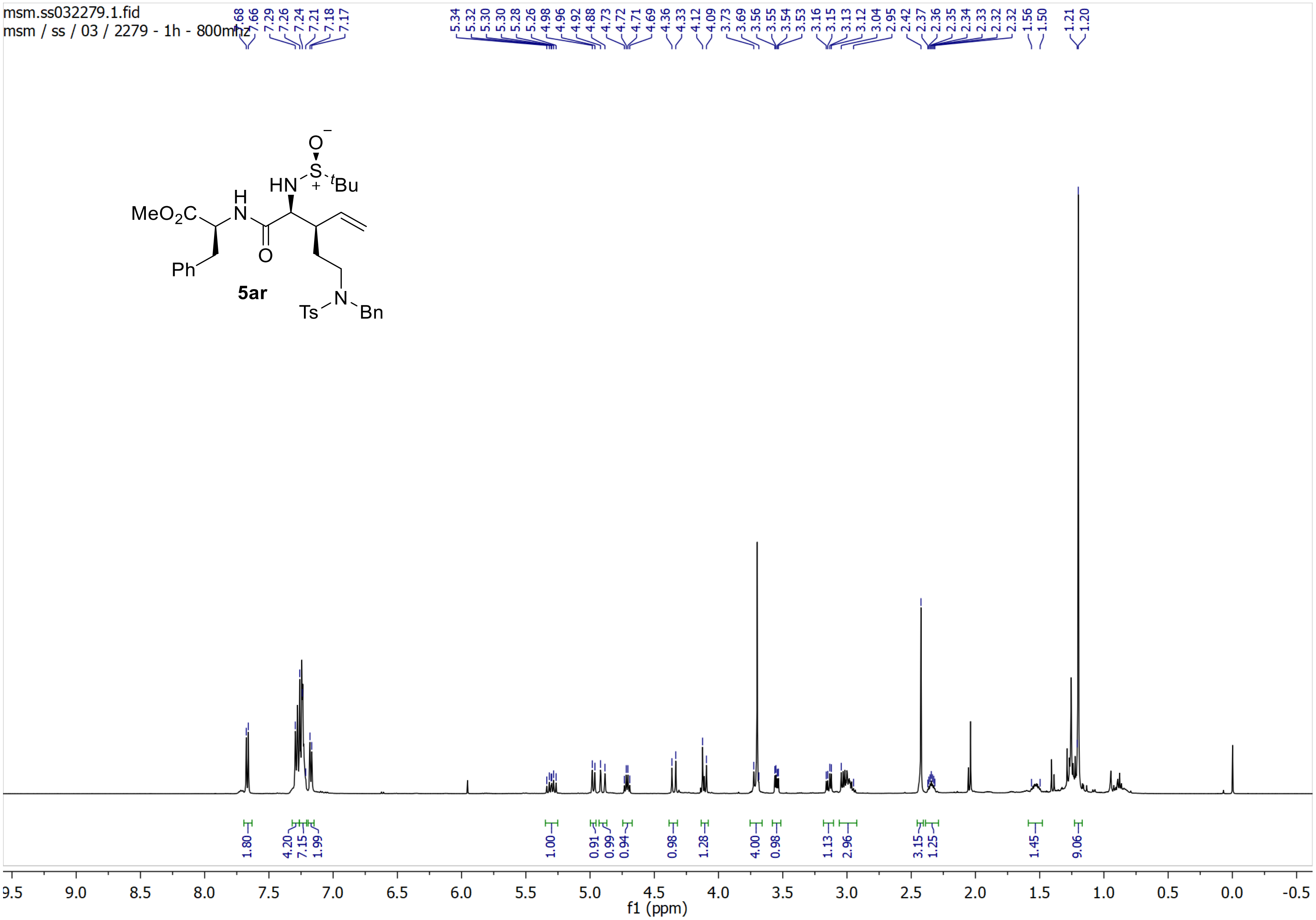
msm.ss032279.1.fid
msm / ss / 03 / 2279 - 1h - 800mhz
5ar
f1 (ppm)

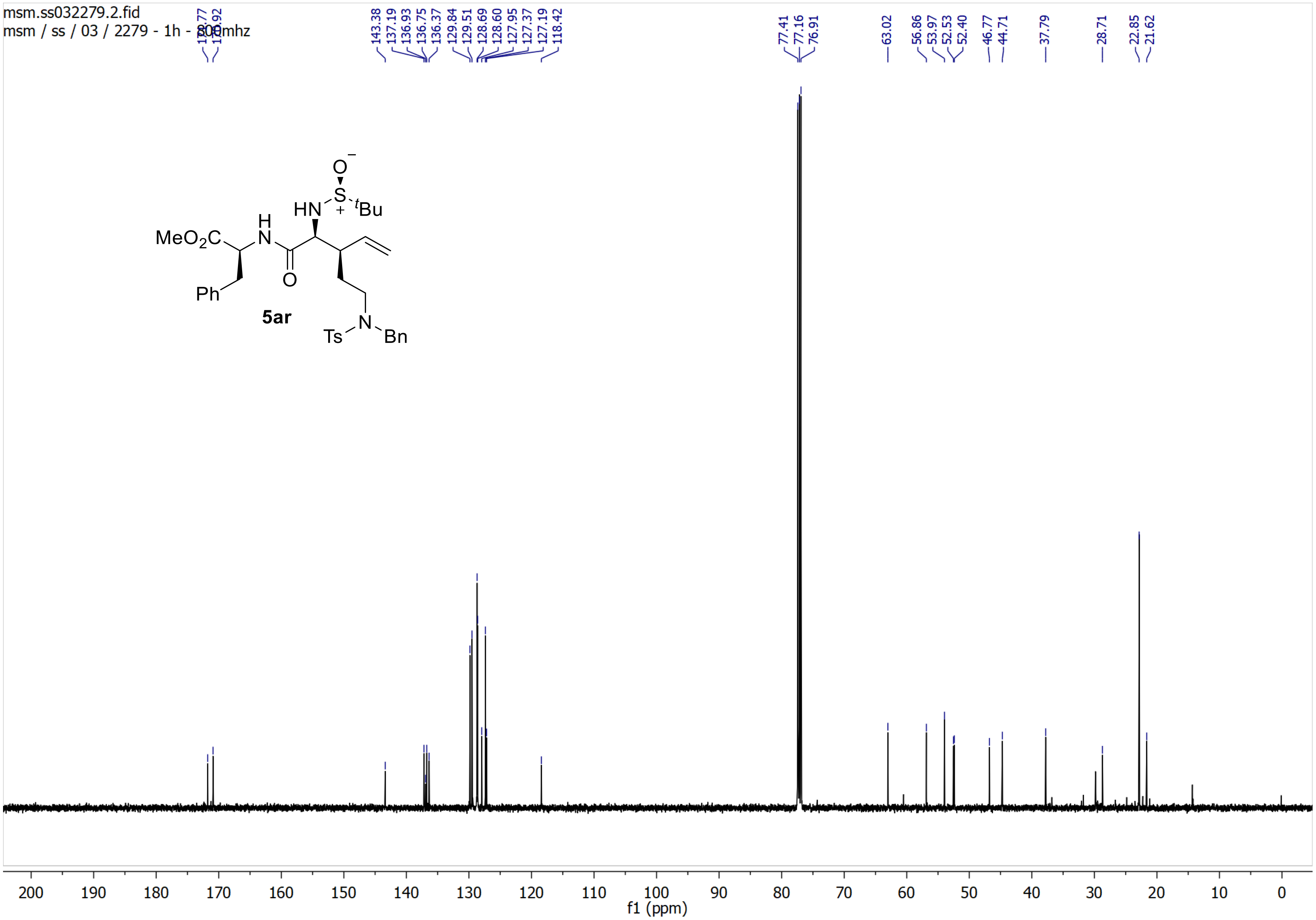
msm.ss032279.2.fid
msm / ss / 03 / 2279 - 1h - 800mhz
143.38
137.19
136.93
136.75
136.37
129.84
129.51
128.69
128.60
127.95
127.37
127.19
118.42
77.41
77.16
76.91
63.02
56.86
53.97
52.53
52.40
46.77
44.71
37.79
28.71
22.85
21.62
MeO2C
H
N
HN
O
S
+
tBu
O
Ph
Ts
N
Bn
5ar
200 190 180 170 160 150 140 130 120 110 100 90 80 70 60 50 40 30 20 10 0
f1 (ppm)

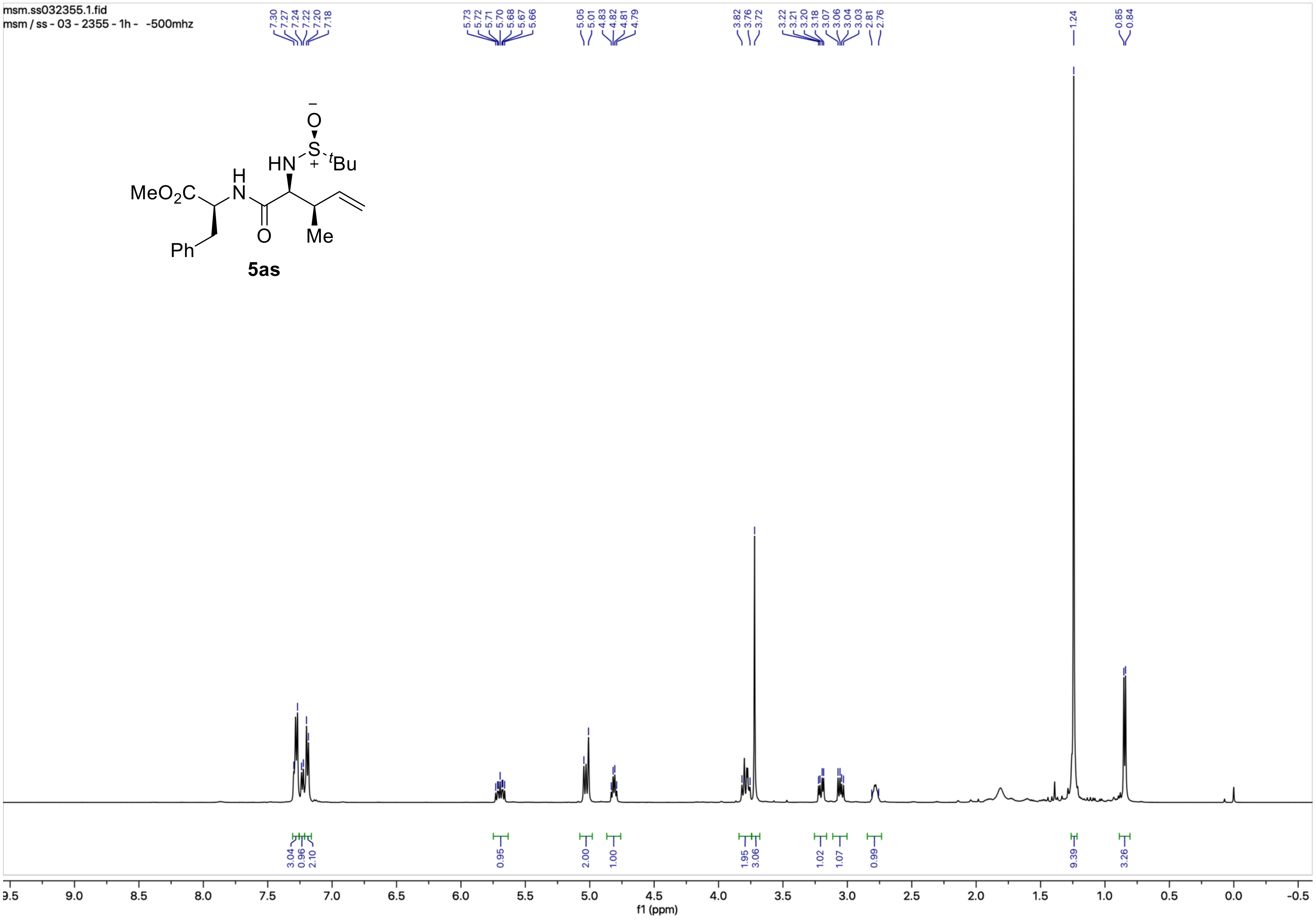

msm.ss032355.1.fid
msm / ss - 03 - 2355 - 1h - -500mhz
5as
f1 (ppm)

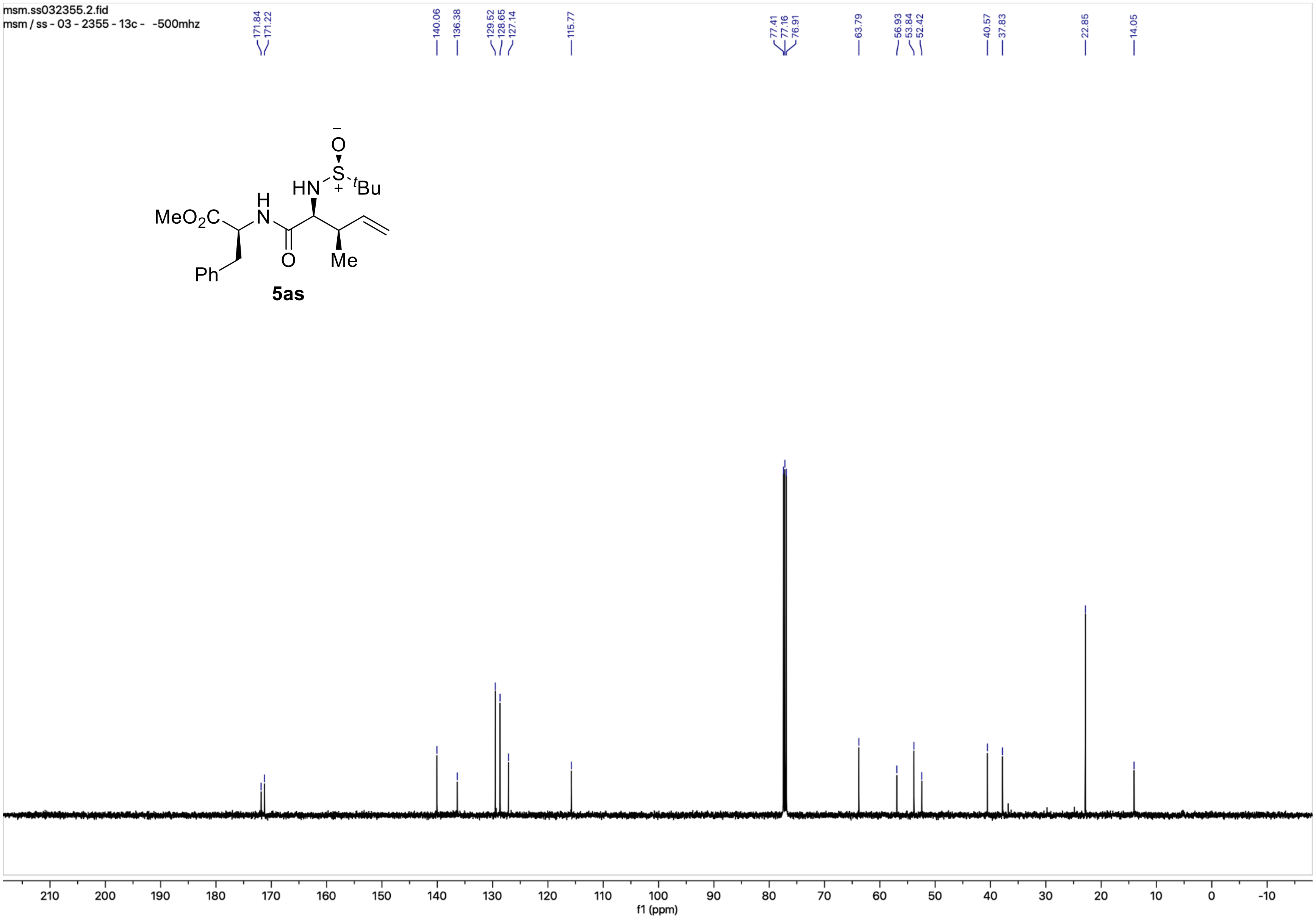
msm.ss032355.2.fid
msm / ss - 03 - 2355 - 13c - -500mhz
171.84
171.22
140.06
136.38
129.52
128.65
127.14
115.77
77.41
77.16
76.91
63.79
56.93
53.84
52.42
40.57
37.83
22.85
14.05
MeO2C
H
N
HN
O
S
+
tBu
O
Me
Ph
210
200
190
180
170
160
150
140
130
120
110
100
90
80
70
60
50
40
30
20
10
0
-10
f1 (ppm)

5as

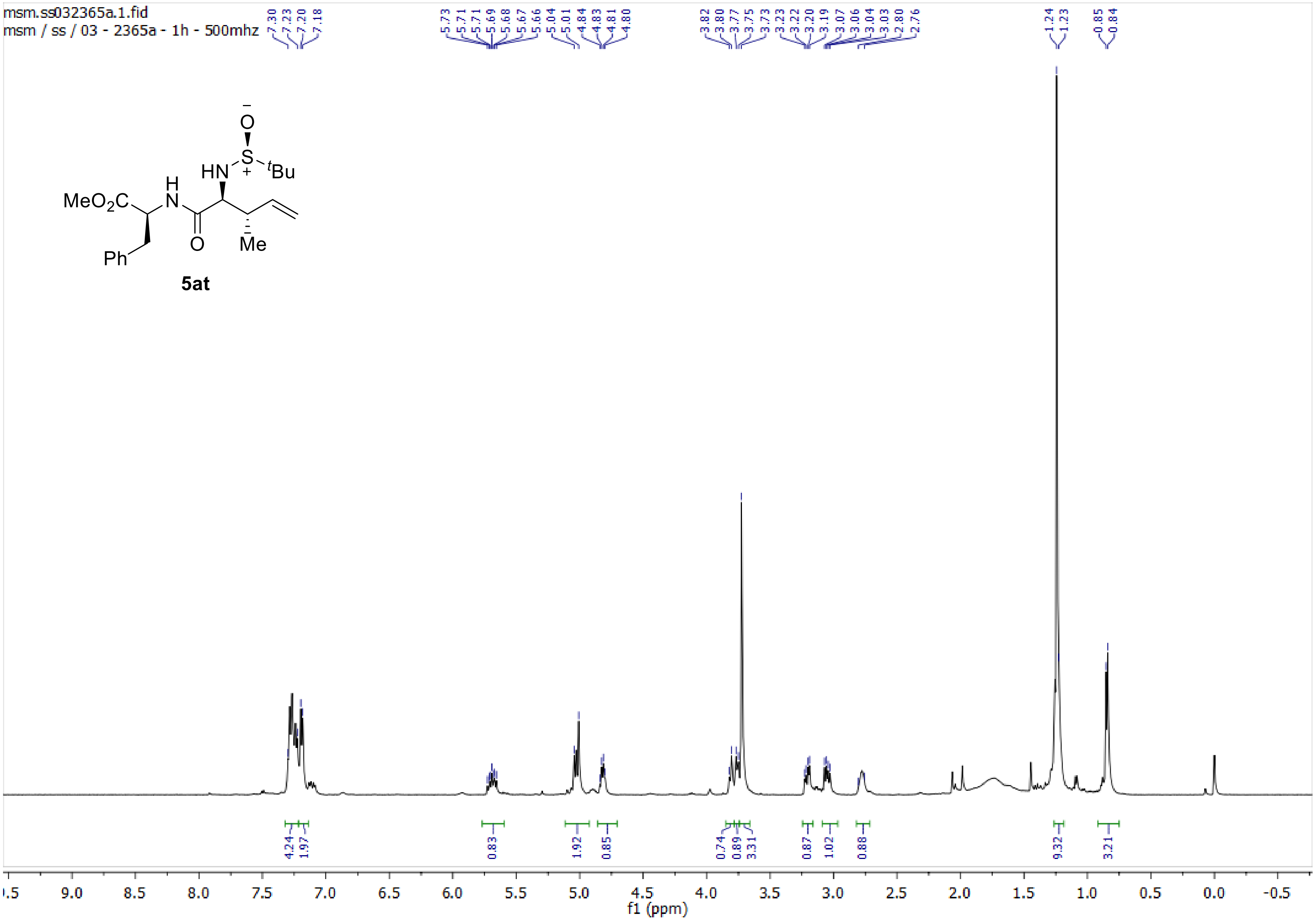
msm.ss032365a.1.fid
msm / ss / 03 - 2365a - 1h - 500mhz
7.30
7.23
7.20
7.18
5.73
5.71
5.71
5.69
5.68
5.67
5.66
5.04
5.01
4.84
4.83
4.81
4.80
3.82
3.80
3.77
3.75
3.73
3.23
3.22
3.20
3.19
3.07
3.06
3.04
3.03
2.80
2.76
1.24
1.23
0.85
0.84
MeO2C
H
N
HN
O
S
+
tBu
Me
Ph
5at
4.24
1.97
0.83
1.92
0.85
0.74
0.89
3.31
0.87
1.02
0.88
9.32
3.21
9.0
8.5
8.0
7.5
7.0
6.5
6.0
5.5
5.0
4.5
4.0
3.5
3.0
2.5
2.0
1.5
1.0
0.5
0.0
-0.5
f1 (ppm)

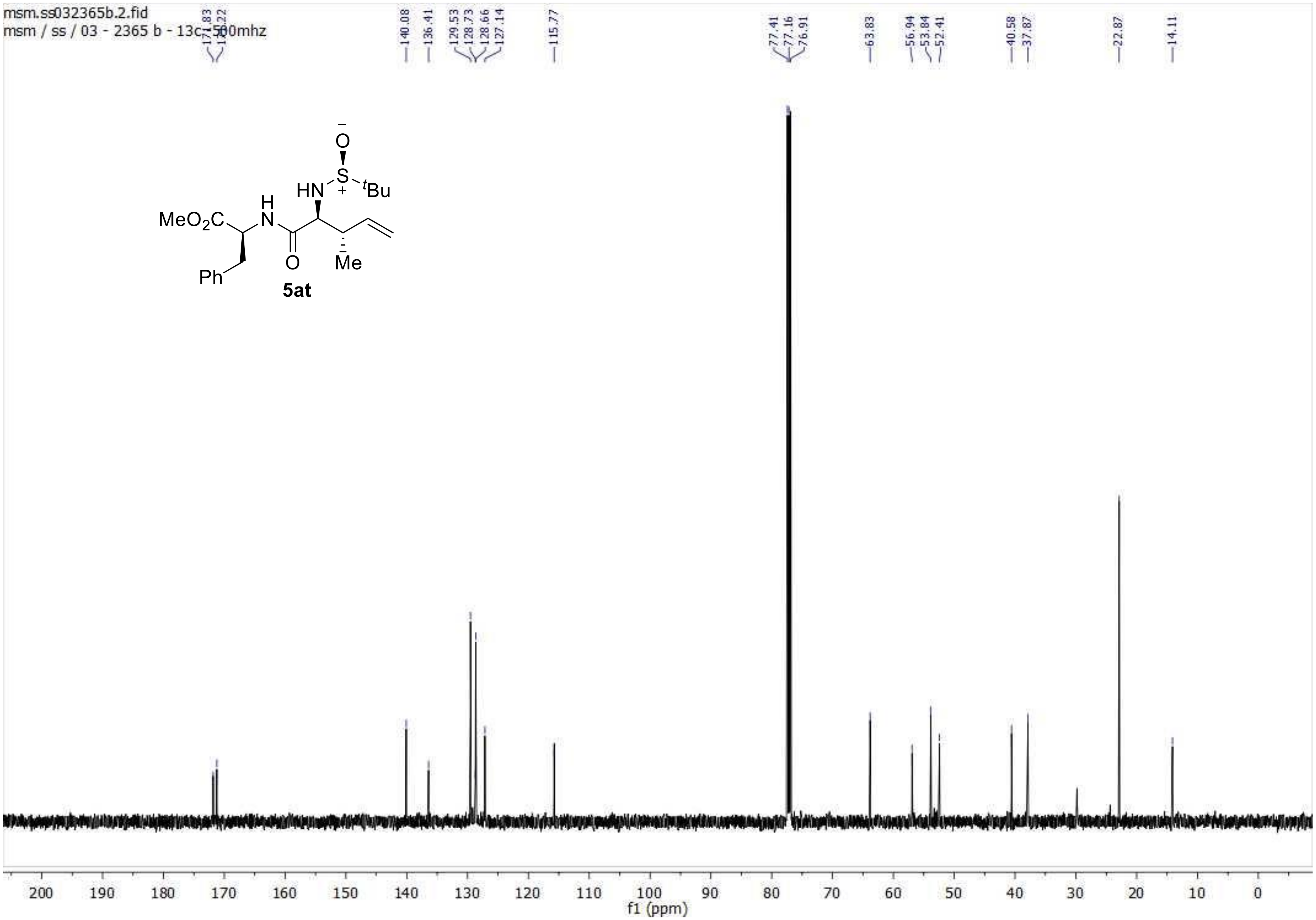
msm.ss032365b.2.fid
msm / ss / 03 - 2365 b - 13c-500mhz
171.83
140.08
136.41
129.53
128.73
128.66
127.14
115.77
77.41
77.16
76.91
63.83
56.94
53.84
52.41
40.58
37.87
22.87
14.11
MeO2C
H
N
HN
O
S
+
tBu
O
Me
Ph
5at
200 190 180 170 160 150 140 130 120 110 100 90 80 70 60 50 40 30 20 10 0
f1 (ppm)

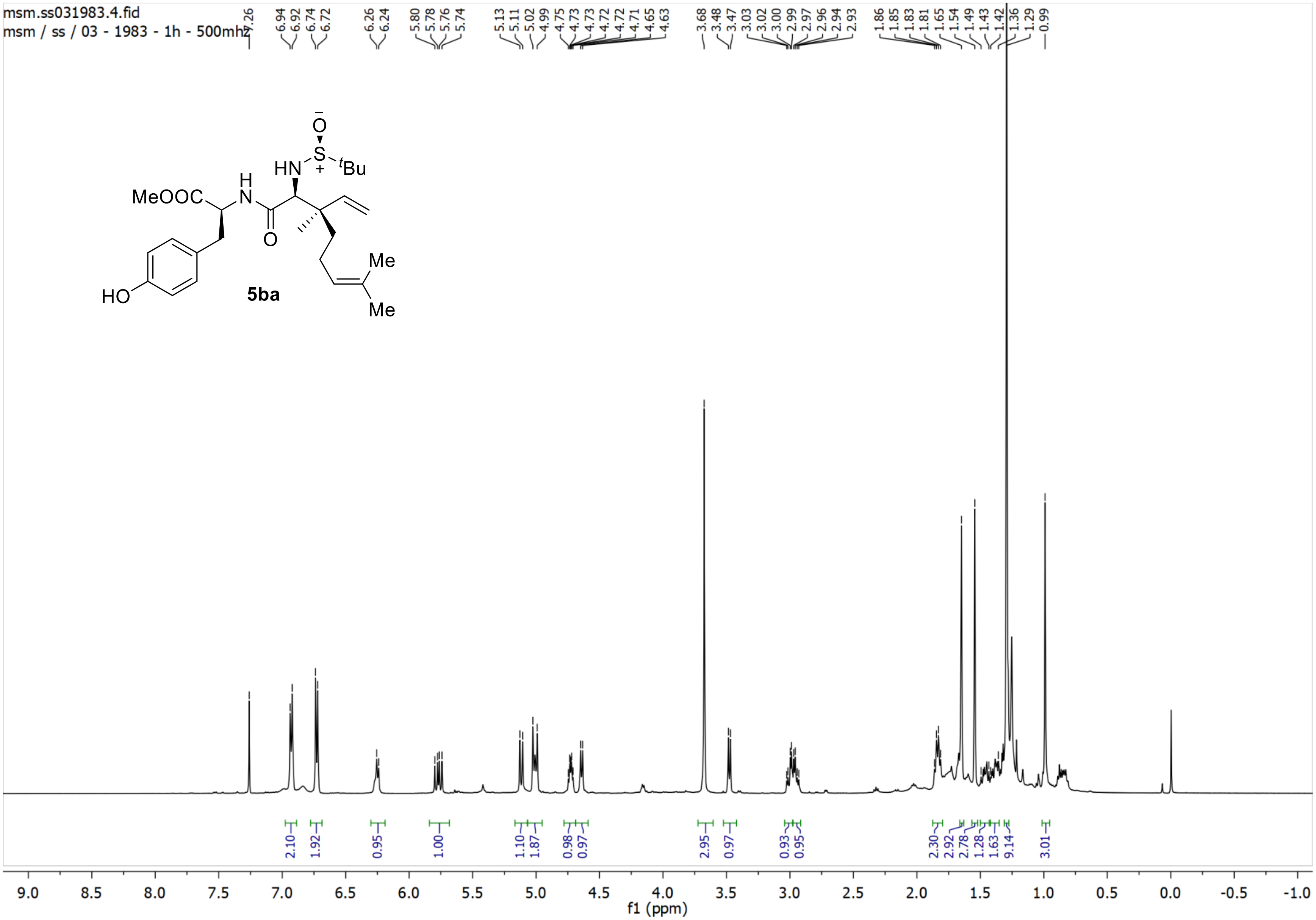

msm.ss031983.4.fid
msm / ss / 03 - 1983 - 1h - 500mhz
7.26
6.94
6.92
6.74
6.72
6.26
6.24
5.80
5.78
5.76
5.74
5.13
5.11
5.02
4.99
4.75
4.73
4.73
4.72
4.72
4.71
4.65
4.63
3.68
3.48
3.47
3.03
3.02
3.00
2.99
2.97
2.96
2.94
2.93
1.86
1.85
1.83
1.81
1.65
1.54
1.49
1.43
1.42
1.36
1.29
0.99
MeOOC
HN
O
S
tBu
H
N
O
Me
Me
HO
5ba
2.10
1.92
0.95
1.00
1.10
1.87
0.98
0.97
2.95
0.97
0.93
0.95
2.30
2.92
2.78
1.28
1.63
9.14
3.01
9.0
8.5
8.0
7.5
7.0
6.5
6.0
5.5
5.0
4.5
4.0
3.5
3.0
2.5
2.0
1.5
1.0
0.5
0.0
-0.5
-1.0
f1 (ppm)

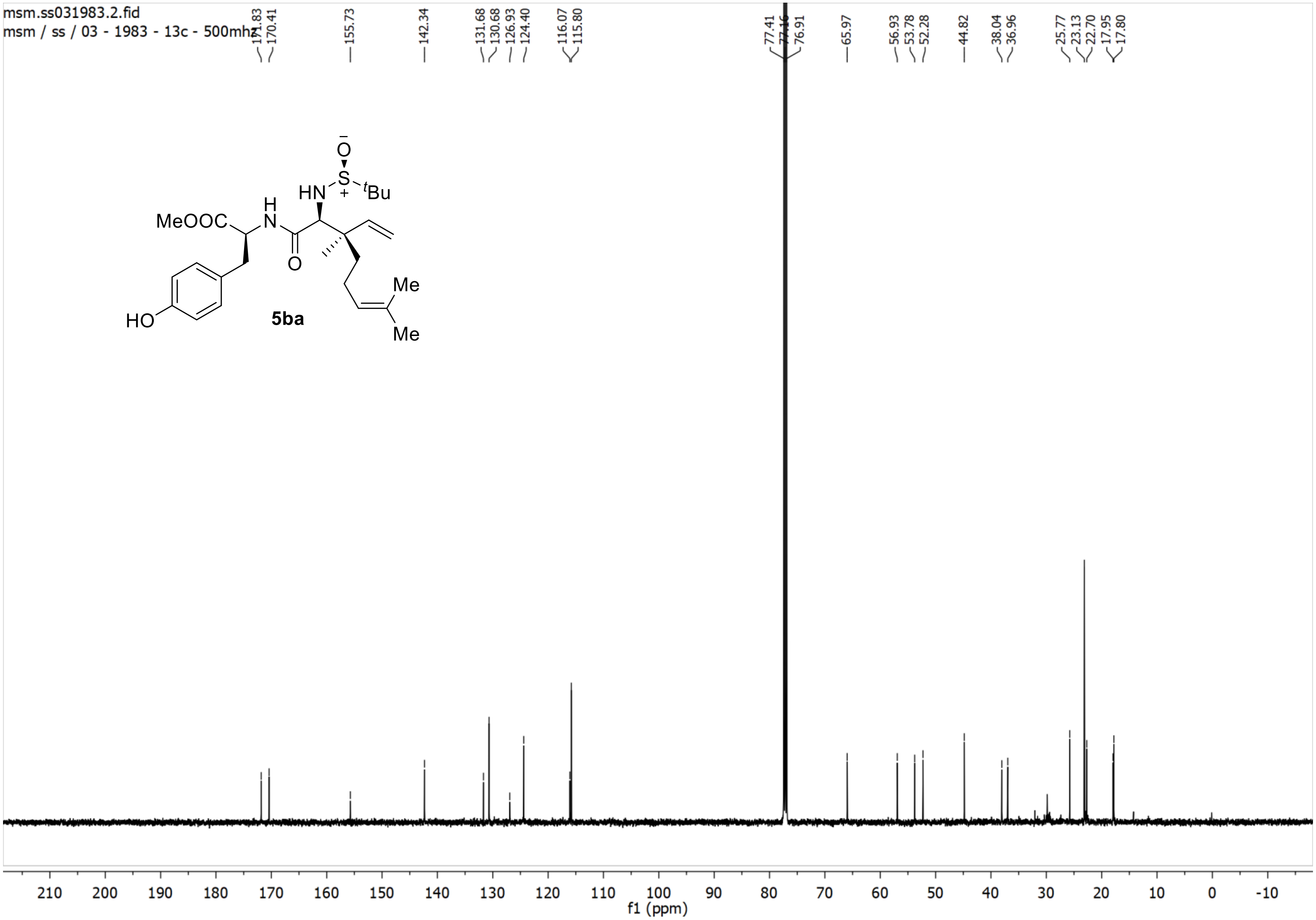

msm.ss031983.2.fid
msm / ss / 03 - 1983 - 13c - 500mhz
171.83
170.41
155.73
142.34
131.68
130.68
126.93
124.40
116.07
115.80
77.41
77.16
76.91
65.97
56.93
53.78
52.28
44.82
38.04
36.96
25.77
23.13
22.70
17.95
17.80
MeOOC
H
N
HN
O
S
+
tBu
O
Me
Me
HO
5ba
210 200 190 180 170 160 150 140 130 120 110 100 90 80 70 60 50 40 30 20 10 0 -10
f1 (ppm)

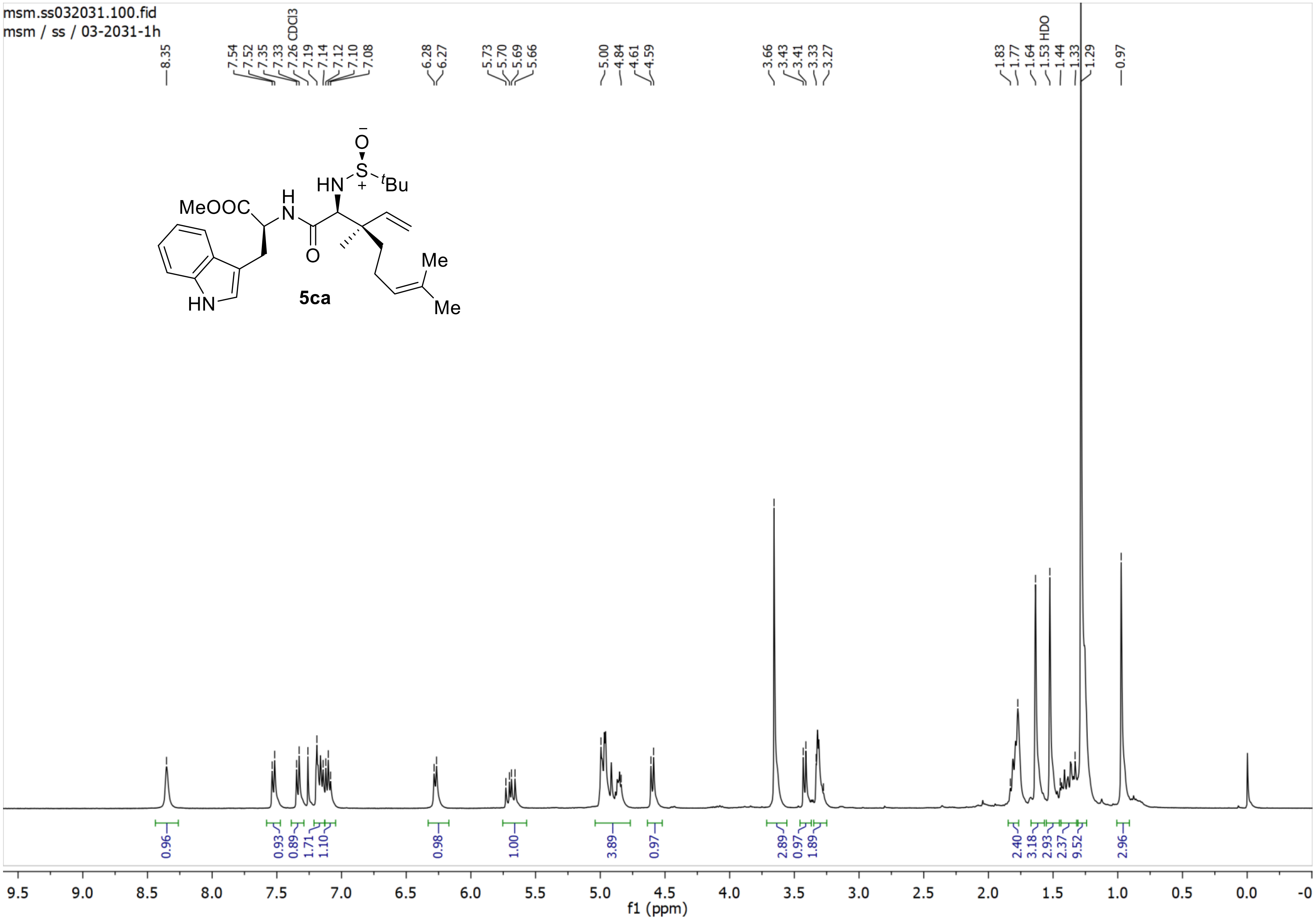
msm.ss032031.100.fid
msm / ss / 03-2031-1h
8.35
7.54
7.52
7.35
7.33
7.26 CDCl3
7.19
7.14
7.12
7.10
7.08
6.28
6.27
5.73
5.70
5.69
5.66
5.00
4.84
4.61
4.59
3.66
3.43
3.41
3.33
3.27
1.83
1.77
1.64
1.53 HDO
1.44
1.33
1.29
0.97
MeOOC
H
N
HN
S
O
tBu
O
Me
Me
HN
5ca
0.96
0.93
0.89
1.71
1.10
0.98
1.00
3.89
0.97
2.89
0.97
1.89
2.40
3.18
2.93
2.37
9.52
2.96
f1 (ppm)

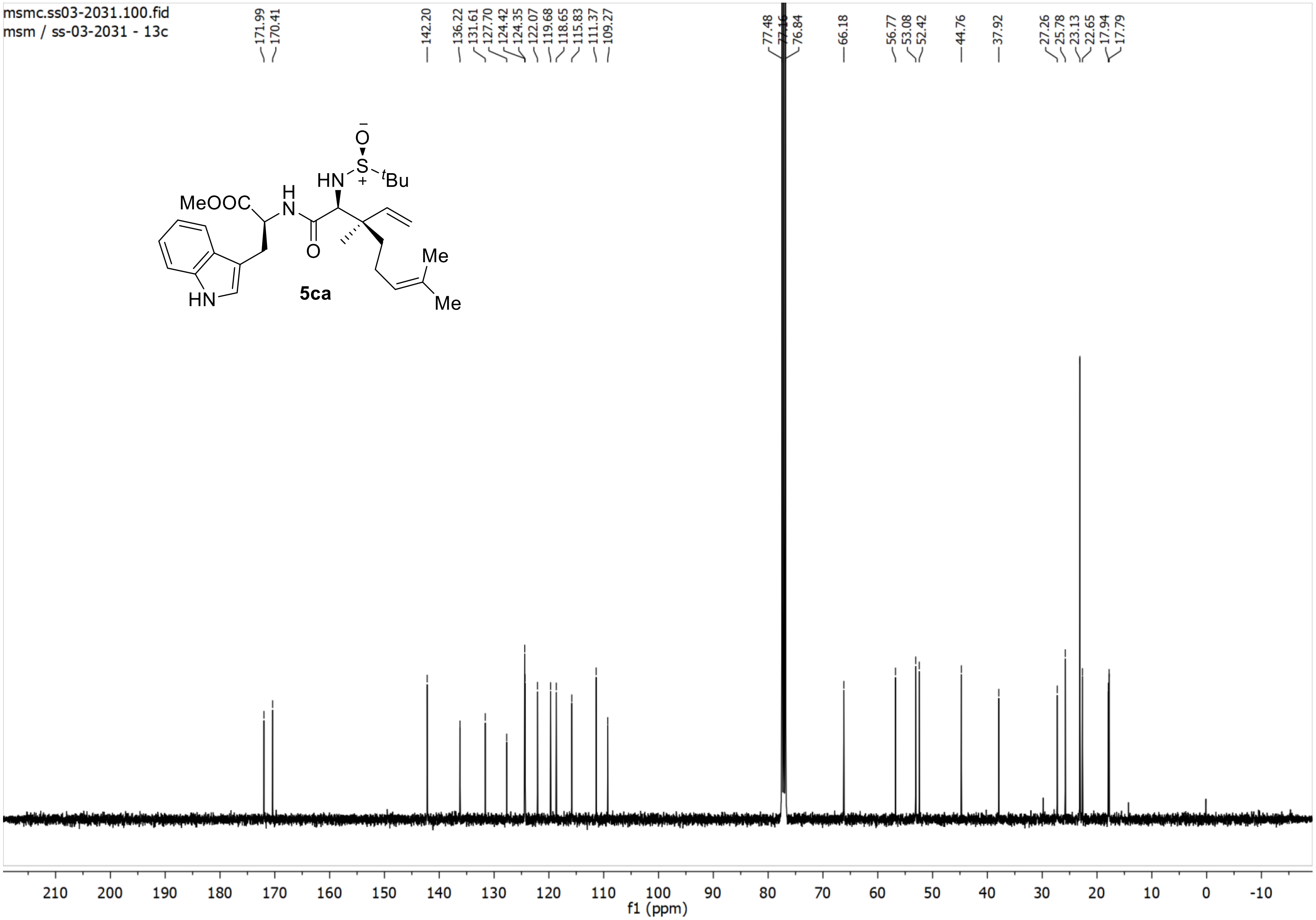
msmc.ss03-2031.100.fid
msm / ss-03-2031 - 13c
171.99
170.41
142.20
136.22
131.61
127.70
124.42
124.35
122.07
119.68
118.65
115.83
111.37
109.27
77.48
77.16
76.84
66.18
56.77
53.08
52.42
44.76
37.92
27.26
25.78
23.13
22.65
17.94
17.79
MeOOC
H
N
HN
O
S
tBu
Me
Me
HN
5ca
210 200 190 180 170 160 150 140 130 120 110 100 90 80 70 60 50 40 30 20 10 0 -10
f1 (ppm)

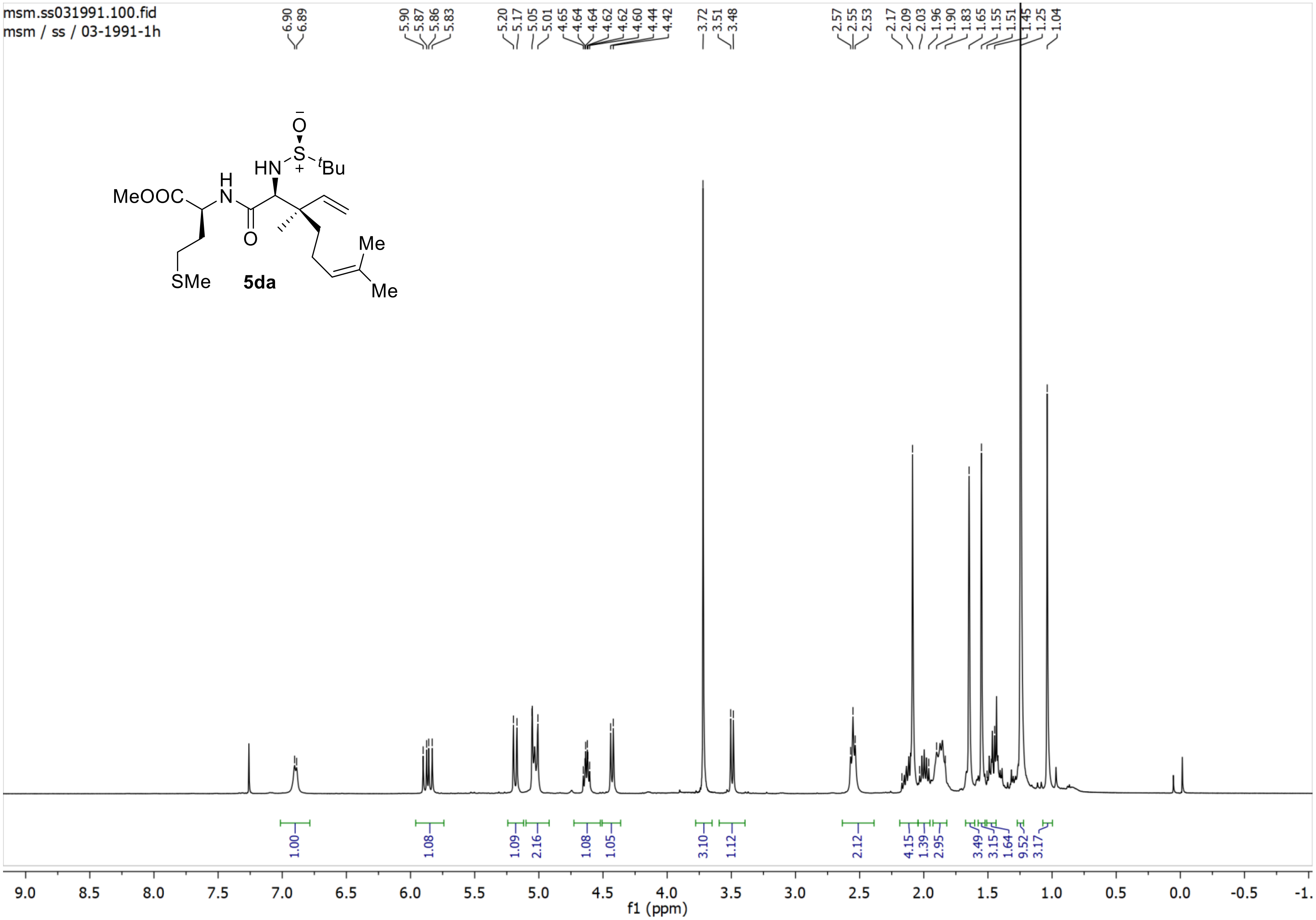

msm.ss031991.100.fid
msm / ss / 03-1991-1h
6.90
6.89
5.90
5.87
5.86
5.83
5.20
5.17
5.05
5.01
4.65
4.64
4.64
4.62
4.62
4.60
4.44
4.42
3.72
3.51
3.48
2.57
2.55
2.53
2.17
2.09
2.03
1.96
1.90
1.83
1.65
1.55
1.51
1.45
1.25
1.04
MeOOC
H
N
O
HN
S
tBu
Me
Me
SMe
5da
1.00
1.08
1.09
2.16
1.08
1.05
3.10
1.12
2.12
4.15
1.39
2.95
3.49
3.15
1.64
9.52
3.17
f1 (ppm)

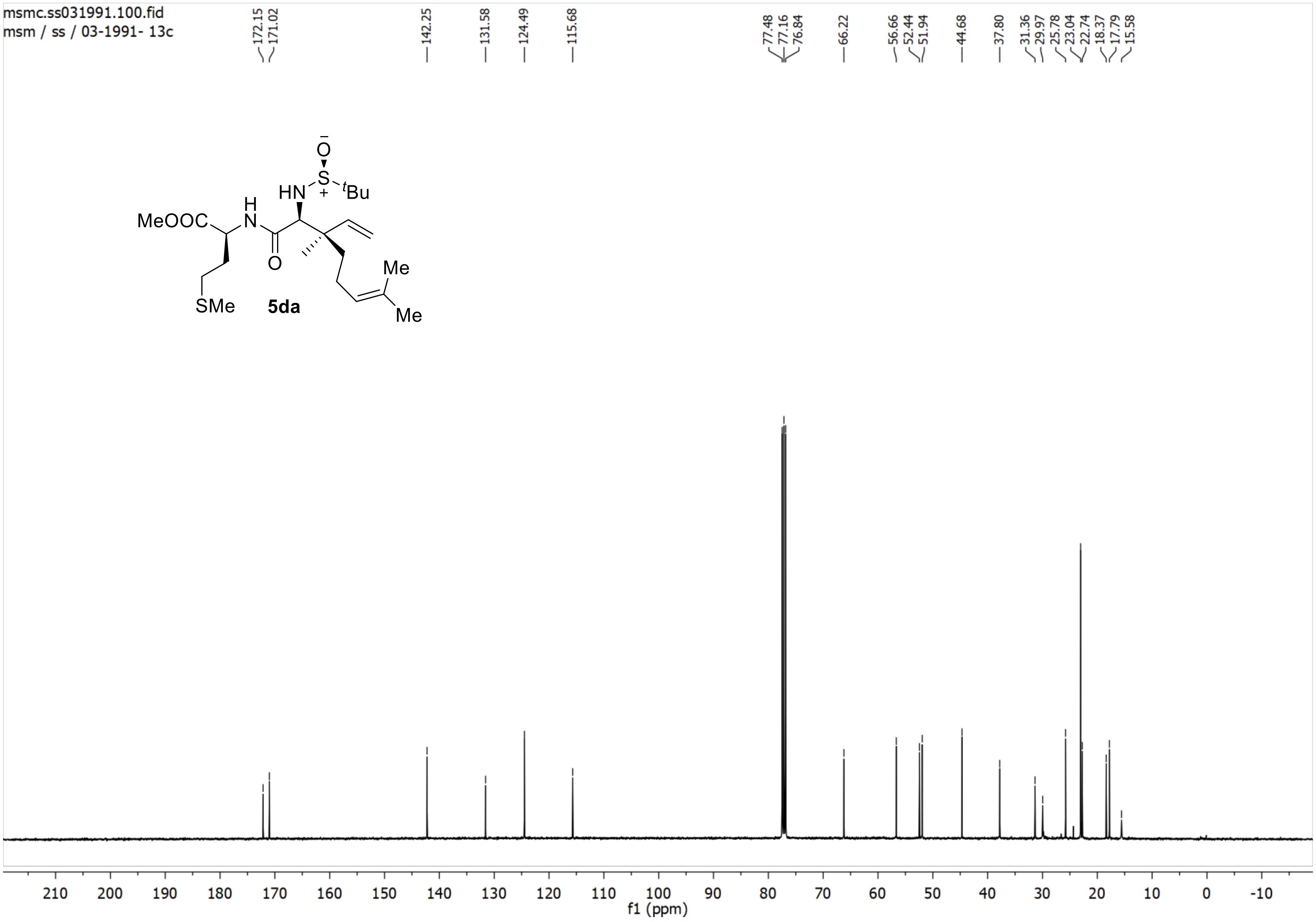
msmc.ss031991.100.fid
msm / ss / 03-1991- 13c
172.15
171.02
142.25
131.58
124.49
115.68
77.48
77.16
76.84
66.22
56.66
52.44
51.94
44.68
37.80
31.36
29.97
25.78
23.04
22.74
18.37
17.79
15.58
MeOOC
H
N
HN
O
S
+
tBu
O
SMe
5da
Me
Me
210
200
190
180
170
160
150
140
130
120
110
100
90
80
70
60
50
40
30
20
10
0
-10
f1 (ppm)

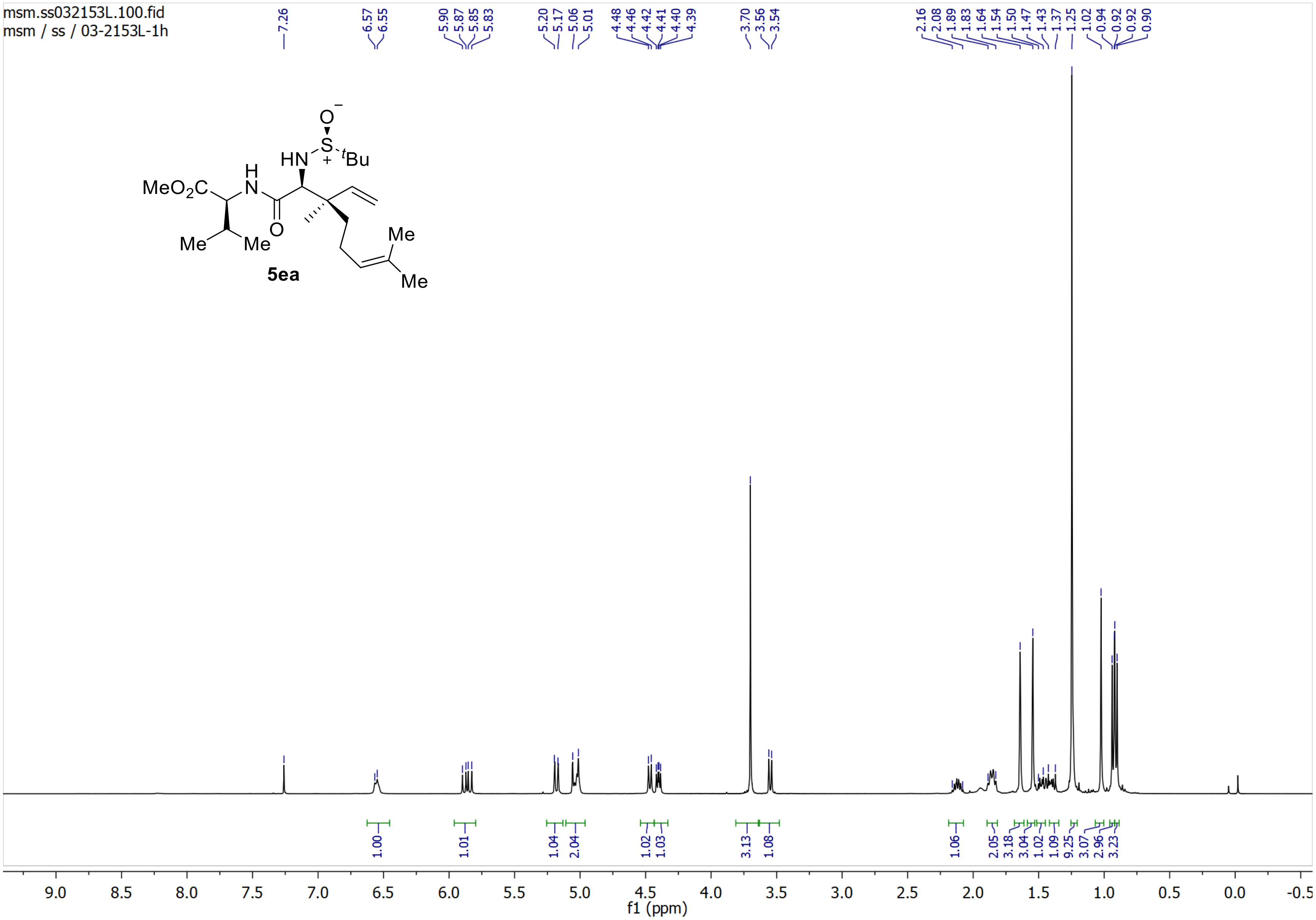
msm.ss032153L.100.fid
msm / ss / 03-2153L-1h
7.26
6.57
6.55
5.90
5.87
5.85
5.83
5.20
5.17
5.06
5.01
4.48
4.46
4.42
4.41
4.40
4.39
3.70
3.56
3.54
2.16
2.08
1.89
1.83
1.64
1.54
1.50
1.47
1.43
1.37
1.25
1.02
0.94
0.92
0.92
0.90
5ea
1.00
1.01
1.04
2.04
1.02
1.03
3.13
1.08
1.06
2.05
3.18
3.04
1.02
1.09
9.25
3.07
2.96
3.23
f1 (ppm)

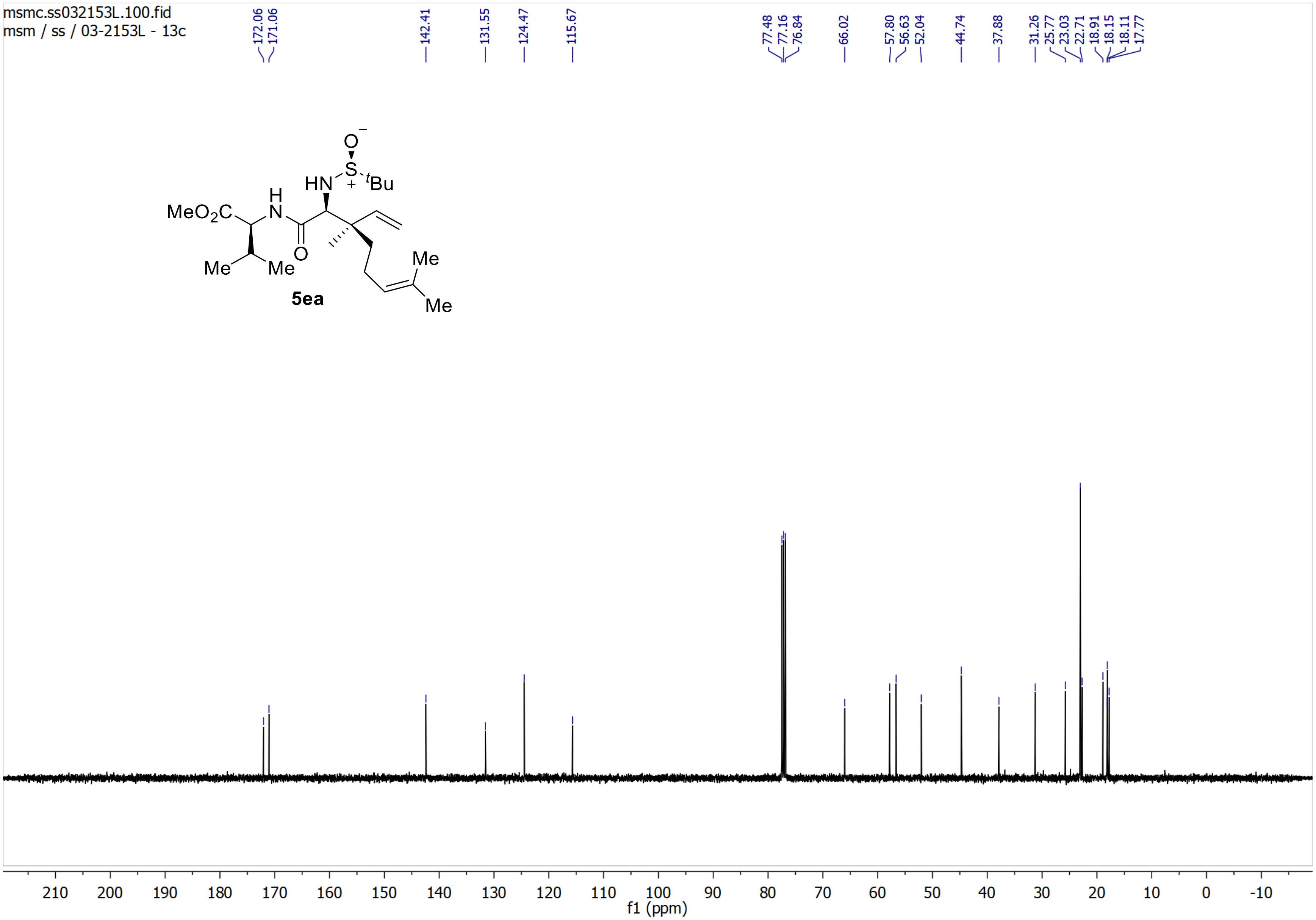
msmc.ss032153L.100.fid
msm / ss / 03-2153L - 13c
172.06
171.06
142.41
131.55
124.47
115.67
77.48
77.16
76.84
66.02
57.80
56.63
52.04
44.74
37.88
31.26
25.77
23.03
22.71
18.91
18.15
18.11
17.77
MeO2C
H
N
HN
S
+
O
tBu
O
Me
Me
Me
Me
5ea
210 200 190 180 170 160 150 140 130 120 110 100 90 80 70 60 50 40 30 20 10 0 -10
f1 (ppm)

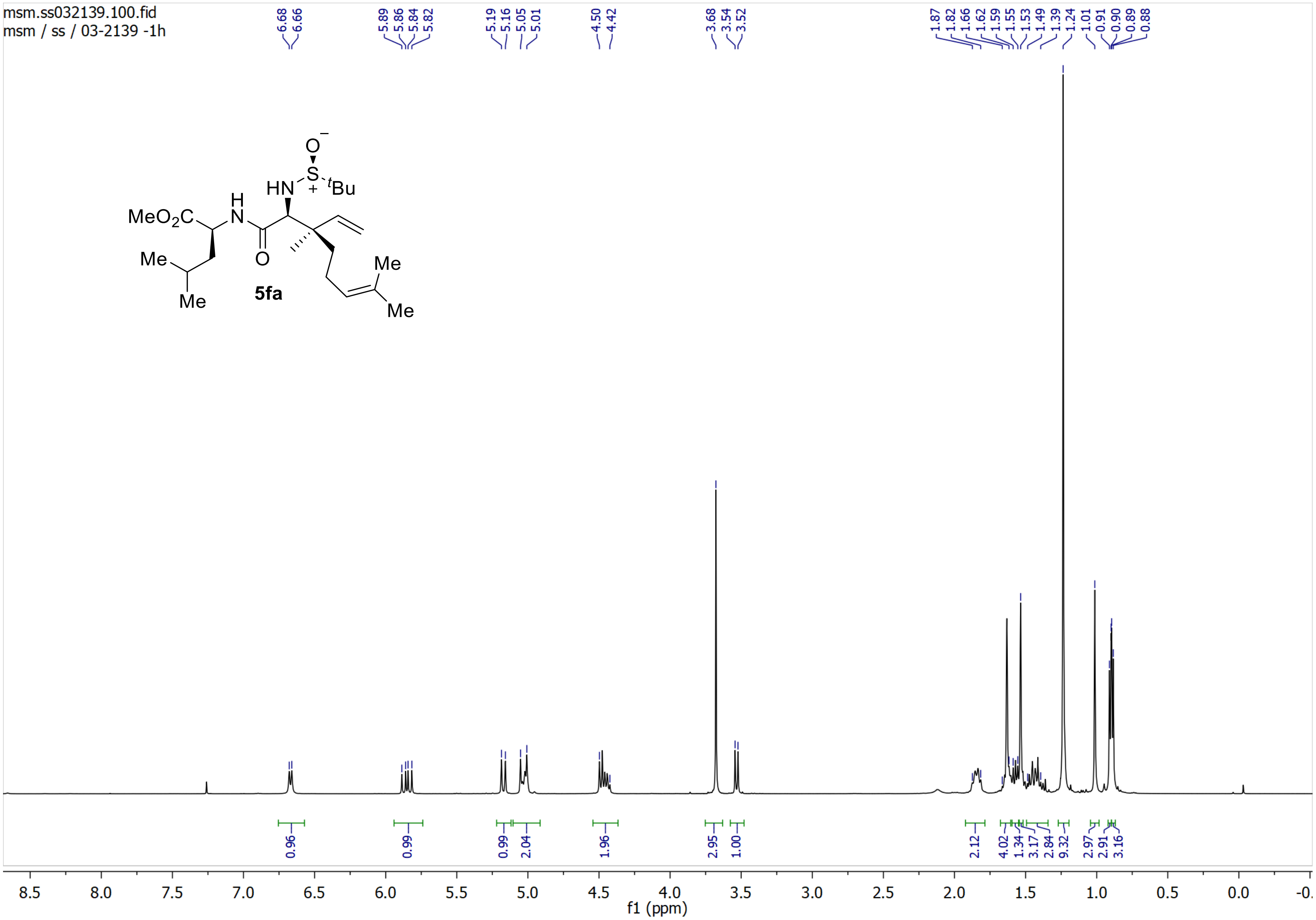

msm.ss032139.100.fid
msm / ss / 03-2139 -1h
6.68
6.66
5.89
5.86
5.84
5.82
5.19
5.16
5.05
5.01
4.50
4.42
3.68
3.54
3.52
1.87
1.82
1.66
1.62
1.59
1.55
1.53
1.49
1.39
1.24
1.01
0.91
0.90
0.89
0.88
5fa
0.96
0.99
0.99
2.04
1.96
2.95
1.00
2.12
4.02
1.34
3.17
2.84
9.32
2.97
2.91
3.16
f1 (ppm)

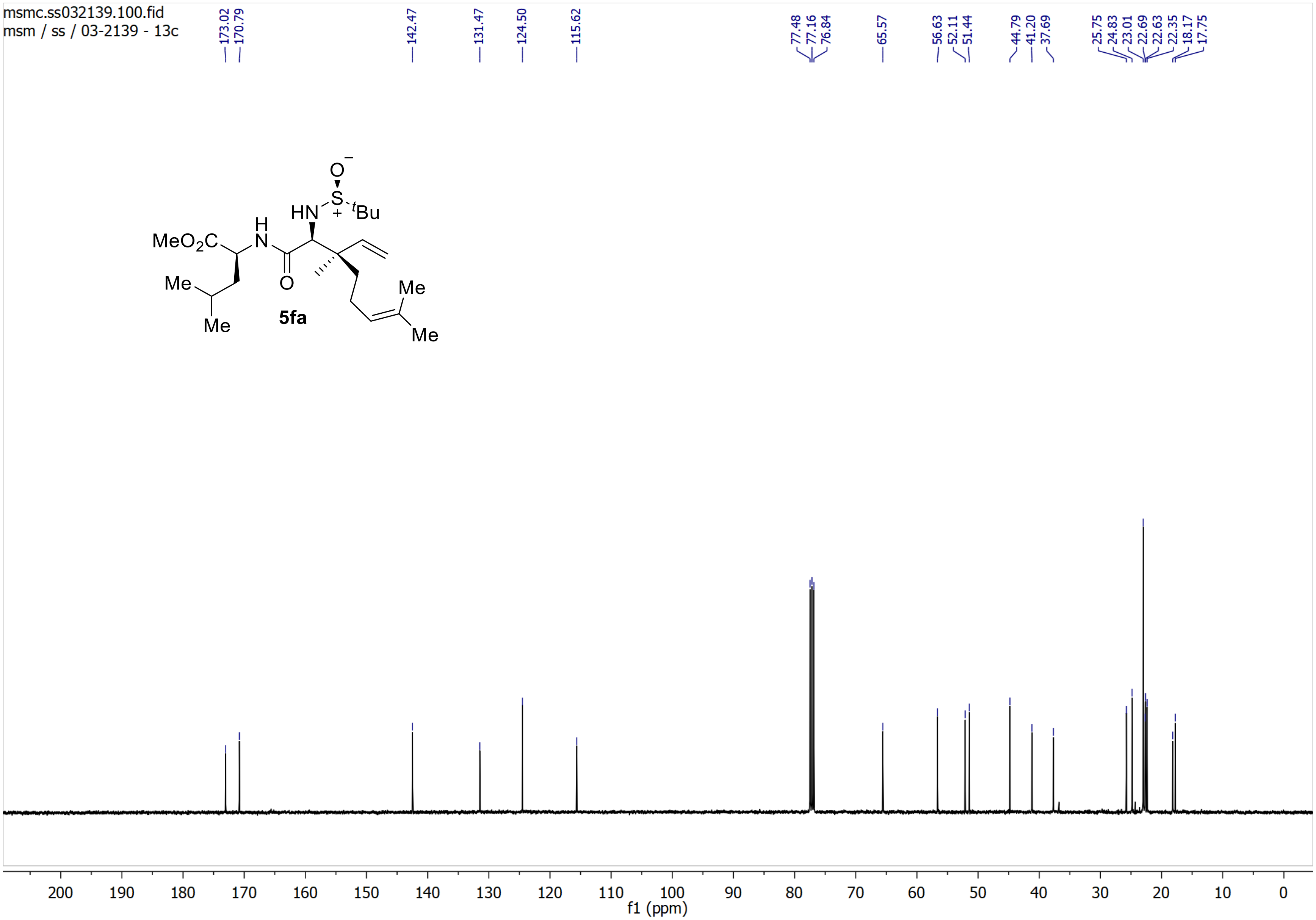
msmc.ss032139.100.fid
msm / ss / 03-2139 - 13c
173.02
170.79
142.47
131.47
124.50
115.62
77.48
77.16
76.84
65.57
56.63
52.11
51.44
44.79
41.20
37.69
25.75
24.83
23.01
22.69
22.63
22.35
18.17
17.75
5fa
f1 (ppm)

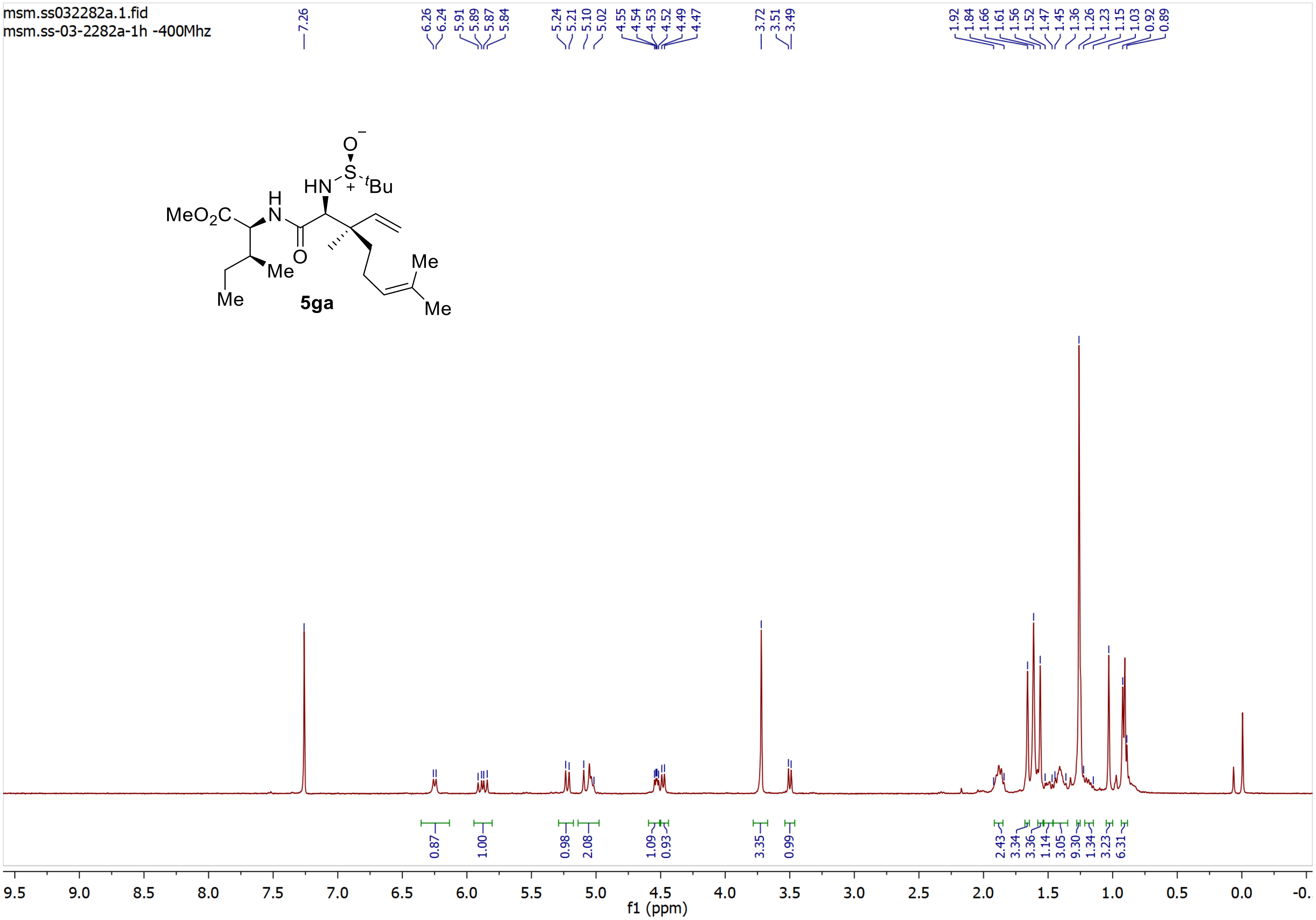
msm.ss032282a.1.fid
msm.ss-03-2282a-1h -400Mhz
7.26
6.26
6.24
5.91
5.89
5.87
5.84
5.24
5.21
5.10
5.02
4.55
4.54
4.53
4.52
4.49
4.47
3.72
3.51
3.49
1.92
1.84
1.66
1.61
1.56
1.52
1.47
1.45
1.36
1.26
1.23
1.15
1.03
0.92
0.89
MeO2C
HN
tBu
Me
5ga
0.87
1.00
0.98
2.08
1.09
0.93
3.35
0.99
2.43
3.34
3.36
1.14
3.05
9.30
1.34
3.23
6.31
f1 (ppm)

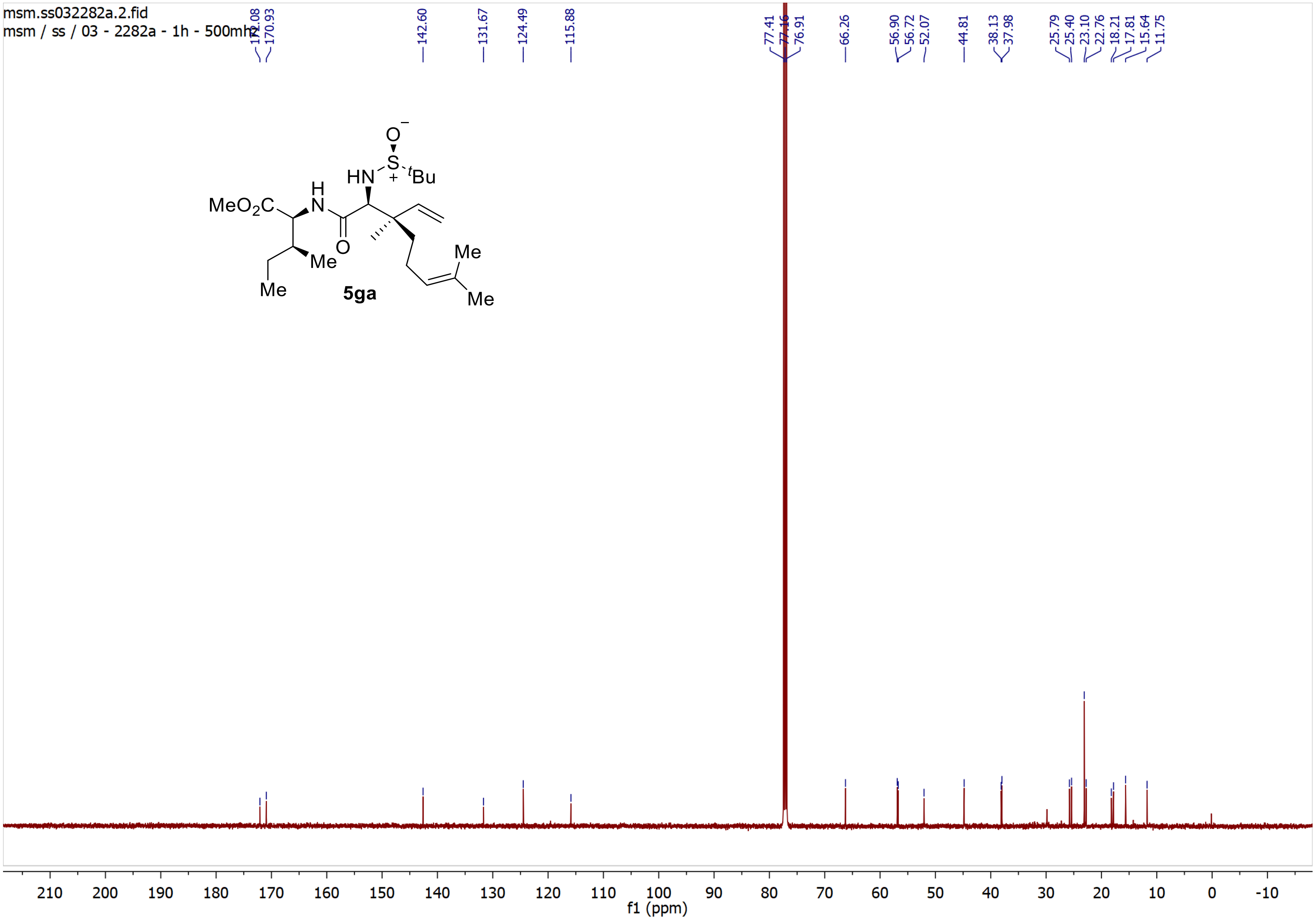
msm.ss032282a.2.fid
msm / ss / 03 - 2282a - 1h - 500mhz
172.08
170.93
142.60
131.67
124.49
115.88
77.41
77.16
76.91
66.26
56.90
56.72
52.07
44.81
38.13
37.98
25.79
25.40
23.10
22.76
18.21
17.81
15.64
11.75
MeO2C
H
N
HN
O
S
tBu
Me
Me
Me
Me
O
5ga
f1 (ppm)

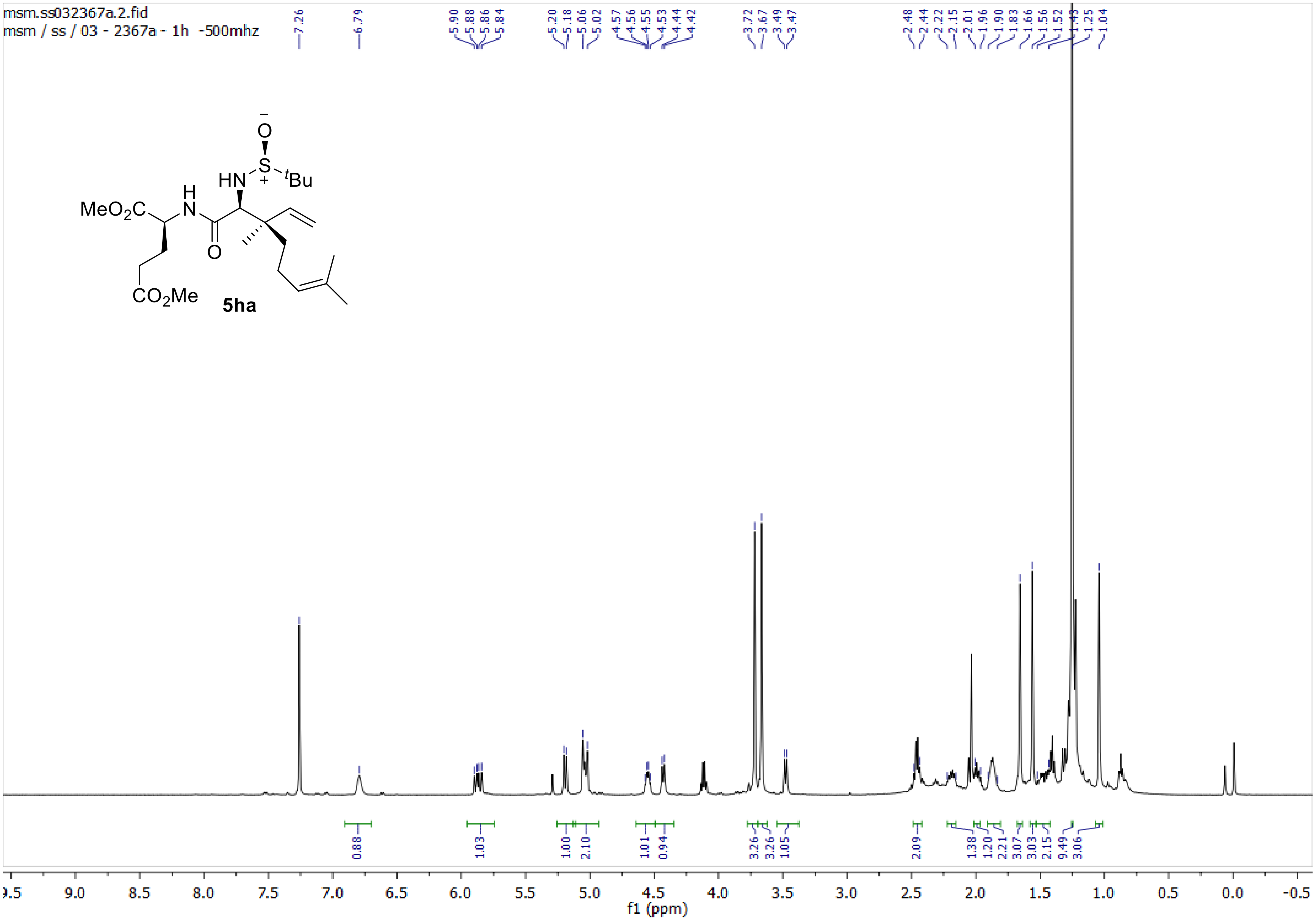
msm.ss032367a.2.fid
msm / ss / 03 - 2367a - 1h -500mhz
7.26
6.79
5.90 5.88 5.86 5.84
5.20 5.18 5.06 5.02
4.57 4.56 4.55 4.53 4.44 4.42
3.72 3.67 3.49 3.47
2.48 2.44 2.22 2.15 2.01 1.96 1.90 1.83 1.66 1.56 1.52 1.25 1.04
MeO2C
HN
O
S
tBu
CO2Me
5ha
0.88
1.03
1.00 2.10
1.01 0.94
3.26 3.26 1.05
2.09
1.38 1.20 2.21 3.07 3.03 2.15 9.49 3.06
f1 (ppm)

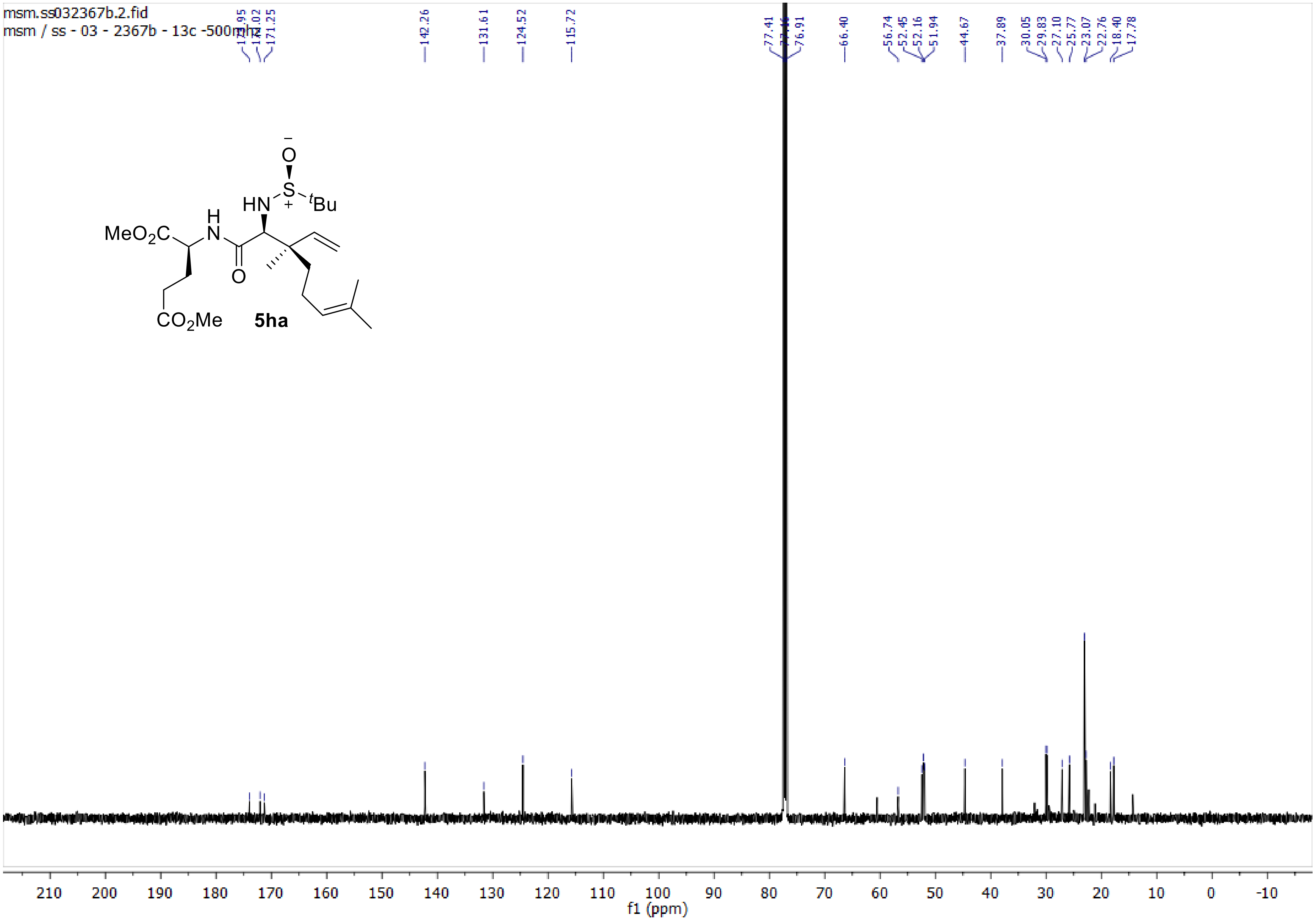
msm.ss032367b.2.fid
msm / ss - 03 - 2367b - 13c -500mhz
142.26
131.61
124.52
115.72
77.41
76.91
66.40
56.74
52.45
52.16
51.94
44.67
37.89
30.05
29.83
27.10
25.77
23.07
22.76
18.40
17.78
MeO2C
CO2Me
HN
tBu
5ha
f1 (ppm)

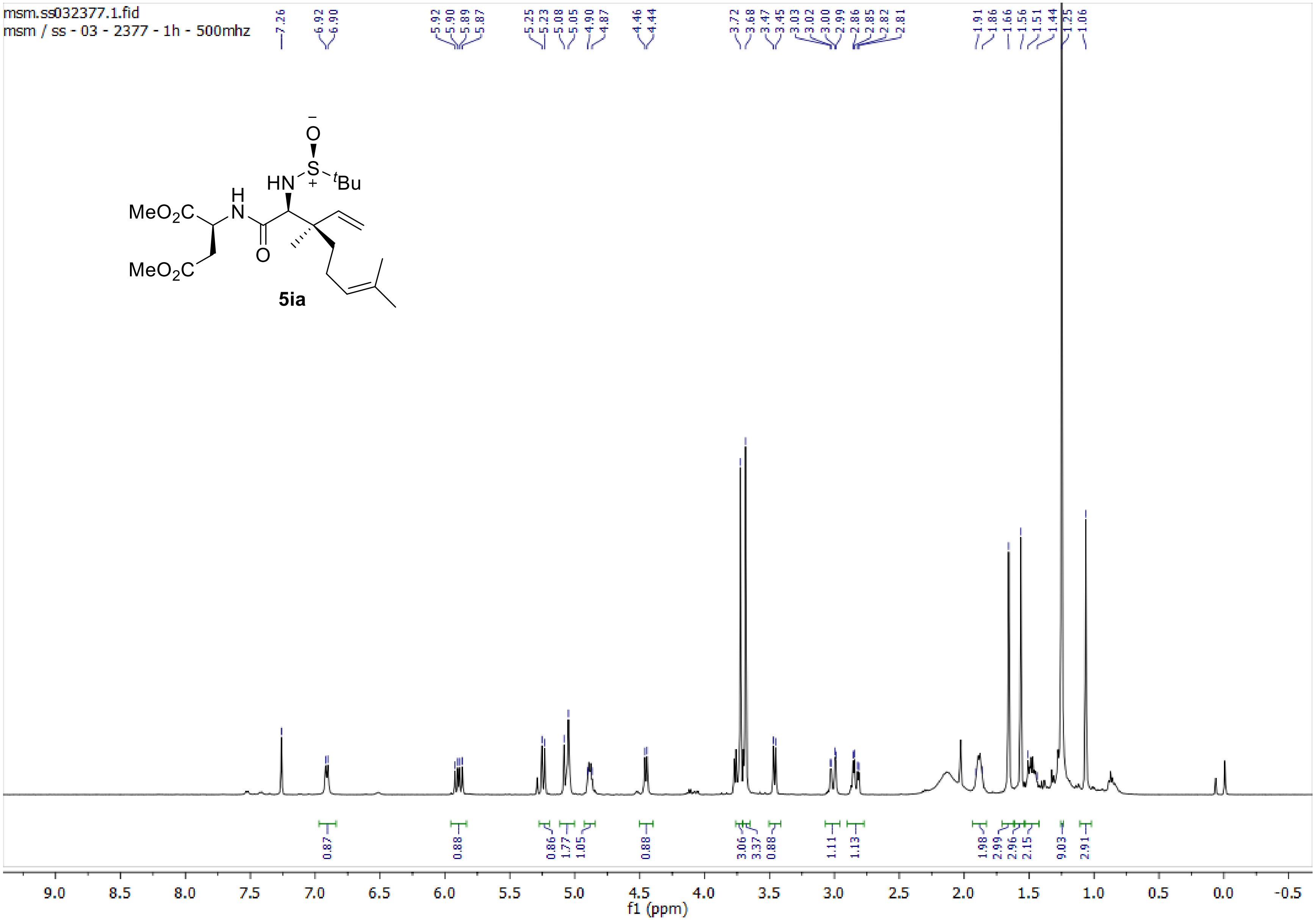
msm.ss032377.1.fid
msm / ss - 03 - 2377 - 1h - 500mhz
7.26
6.92
6.90
5.92
5.90
5.89
5.87
5.25
5.23
5.08
5.05
4.90
4.87
4.46
4.44
3.72
3.68
3.47
3.45
3.03
3.02
3.00
2.99
2.86
2.85
2.82
2.81
1.91
1.86
1.66
1.56
1.51
1.44
1.25
1.06
MeO2C
MeO2C
H
N
HN
S
tBu
O
5ia
0.87
0.88
0.86
1.77
1.05
0.88
3.06
3.37
0.88
1.11
1.13
1.98
2.99
2.96
2.15
9.03
2.91
f1 (ppm)

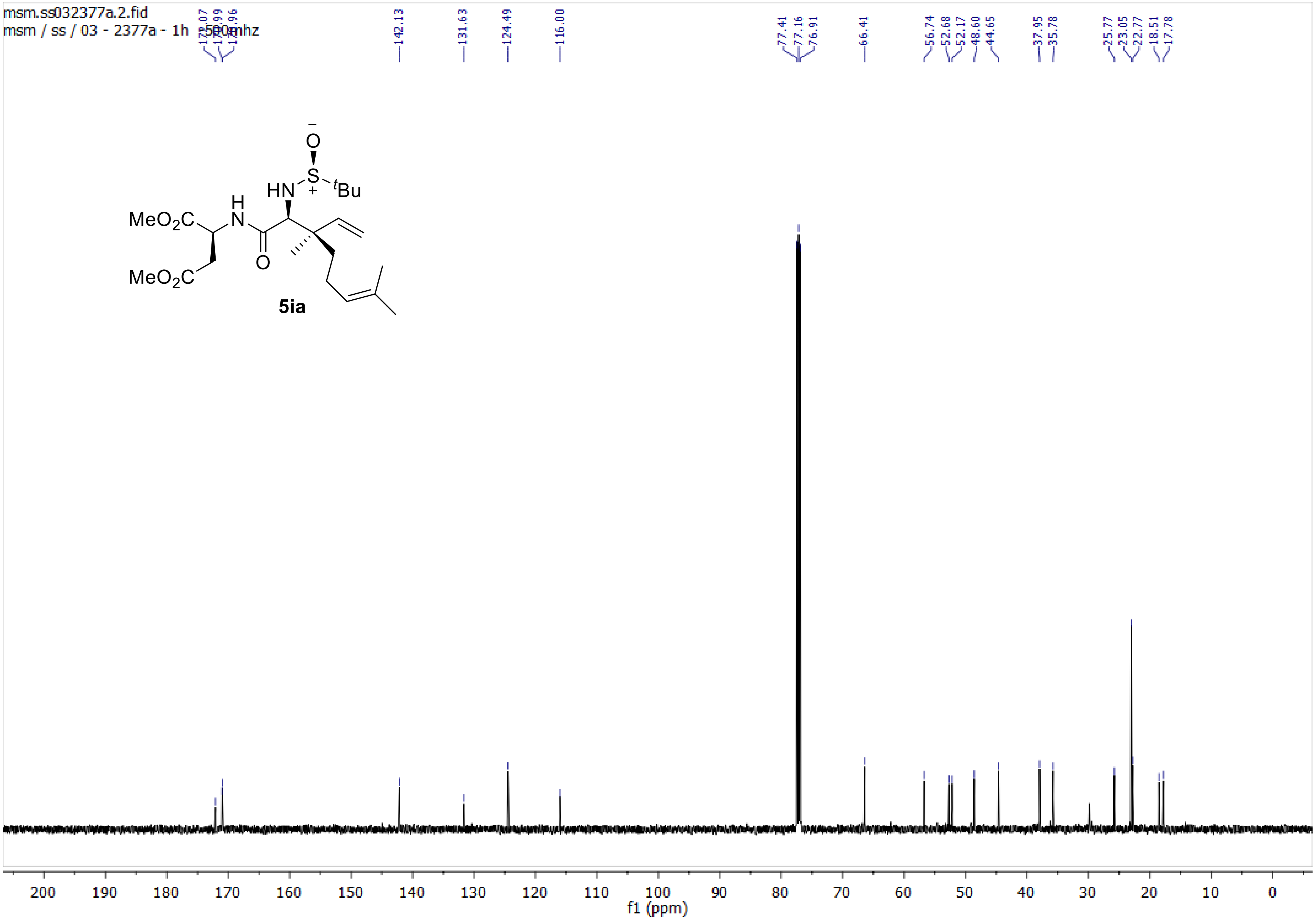

msm.ss032377a.2.fid
msm / ss / 03 - 2377a - 1h
142.13
131.63
124.49
116.00
77.41
77.16
76.91
66.41
56.74
52.68
52.17
48.60
44.65
37.95
35.78
25.77
23.05
22.77
18.51
17.78
5ia
f1 (ppm)

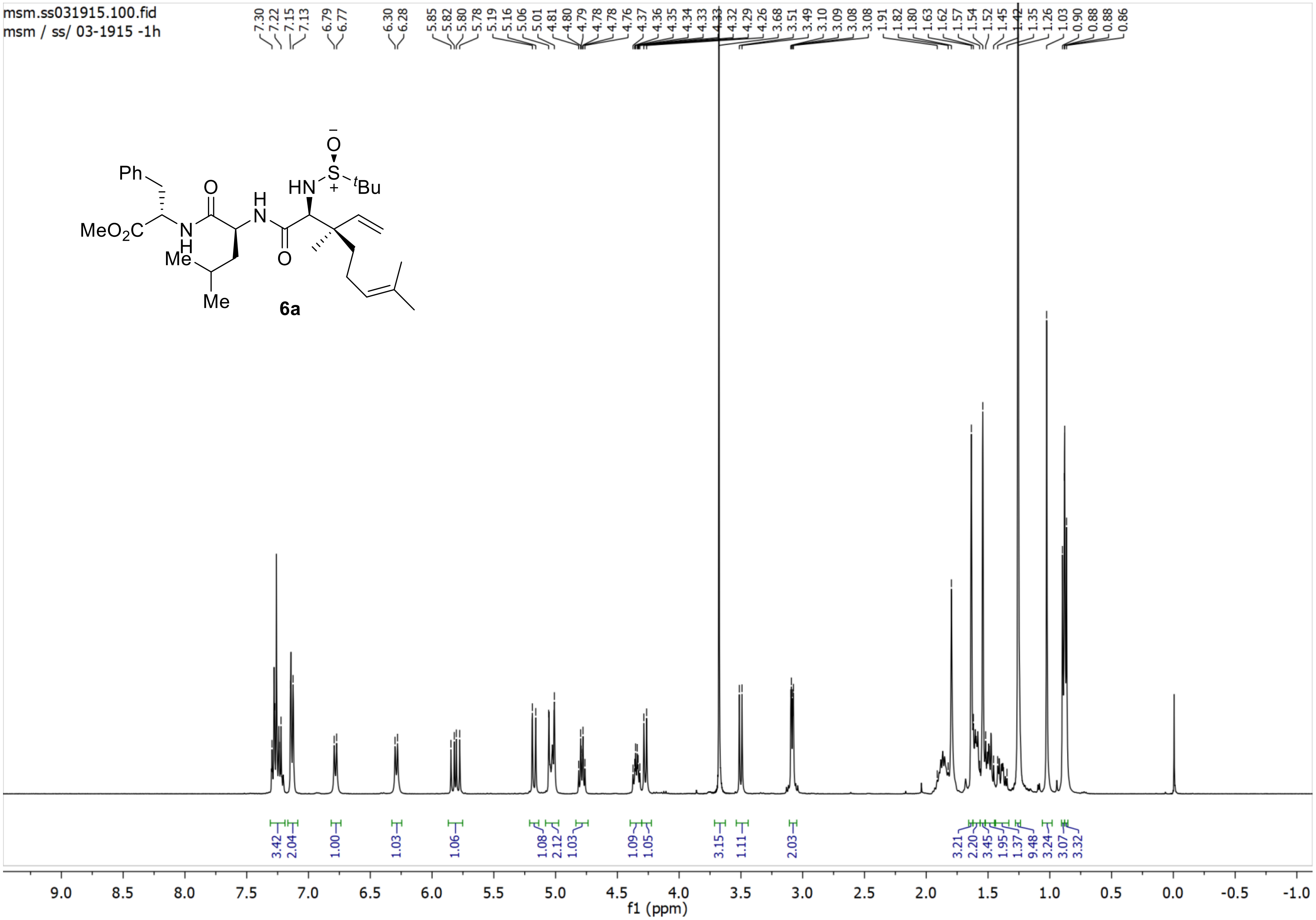
msm.ss031915.100.fid
msm / ss/ 03-1915 -1h
6a
f1 (ppm)

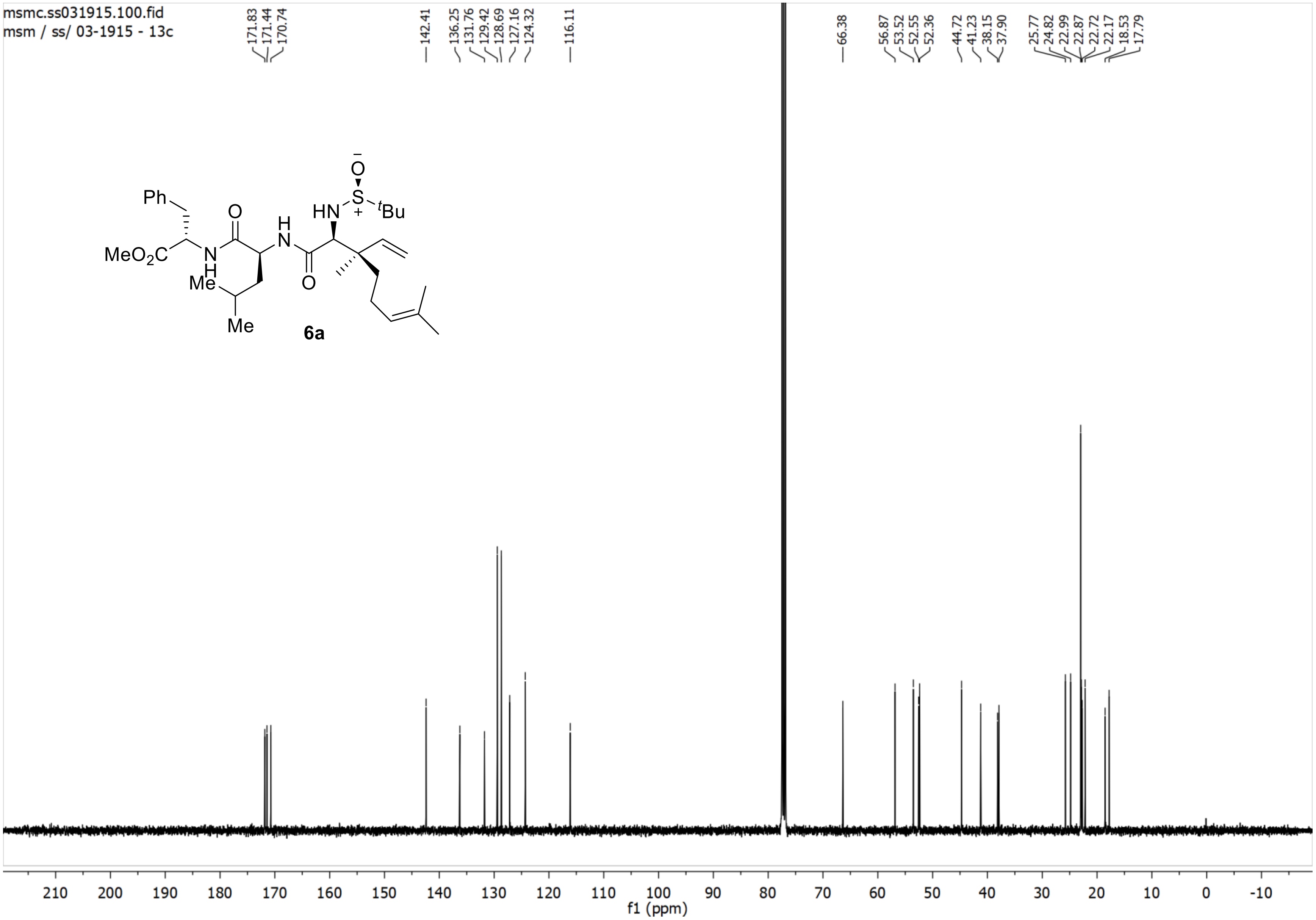
msmc.ss031915.100.fid
msm / ss/ 03-1915 - 13c
171.83
171.44
170.74
142.41
136.25
131.76
129.42
128.69
127.16
124.32
116.11
66.38
56.87
53.52
52.55
52.36
44.72
41.23
38.15
37.90
25.77
24.82
22.99
22.87
22.72
22.17
18.53
17.79
Ph
MeO2C
N
H
O
N
H
O
HN
S
O
tBu
Me
Me
6a
210
200
190
180
170
160
150
140
130
120
110
100
90
80
70
60
50
40
30
20
10
0
-10
f1 (ppm)

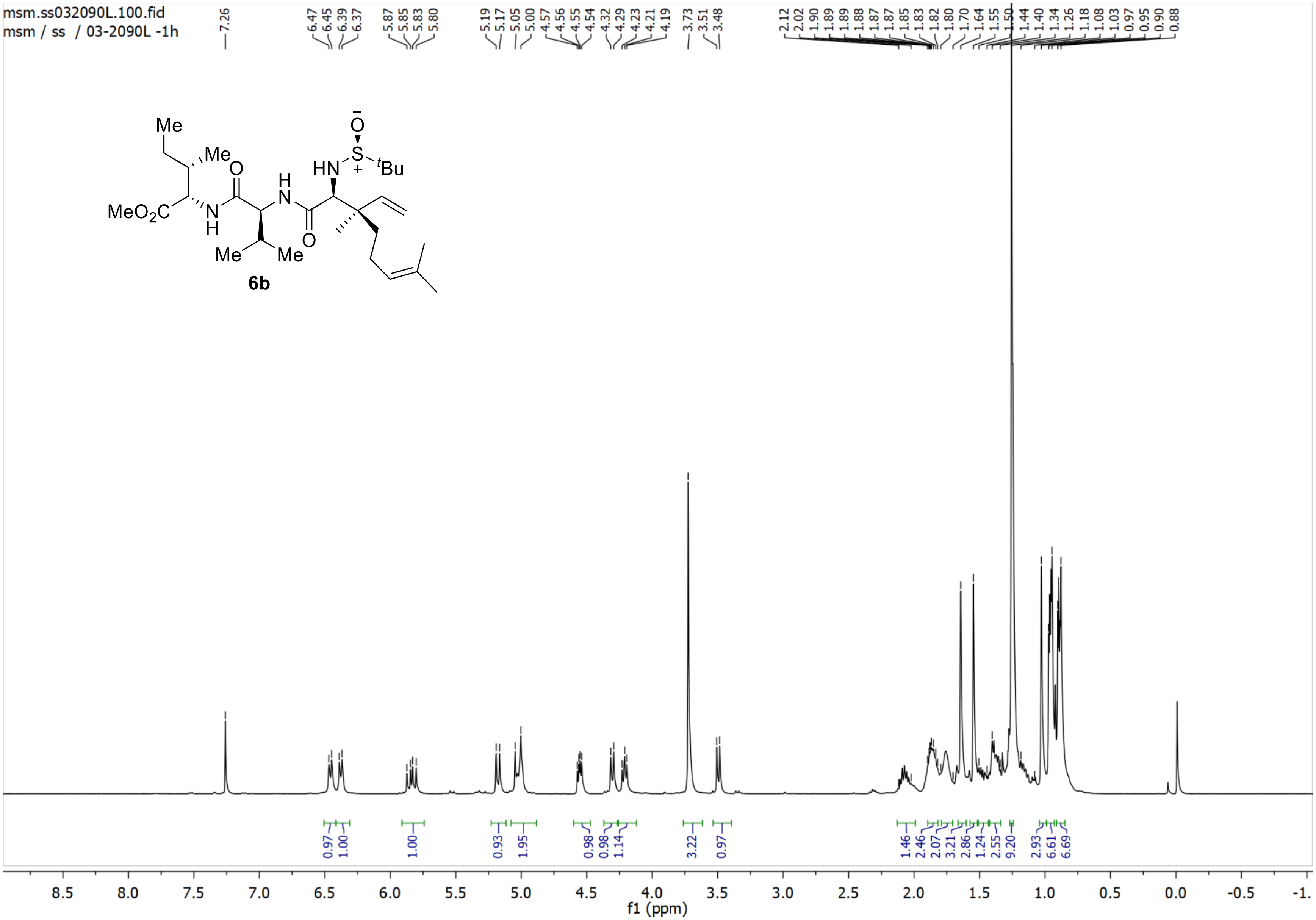
msm.ss032090L.100.fid
msm / ss / 03-2090L -1h
7.26
6.47 6.45 6.39 6.37
5.87 5.85 5.83 5.80
5.19 5.17 5.05 5.00 4.57 4.56 4.55 4.54 4.32 4.29 4.23 4.21 4.19
3.73 3.51 3.48
2.12 2.02 1.90 1.89 1.89 1.88 1.87 1.87 1.85 1.83 1.82 1.80 1.70 1.64 1.55 1.50 1.44 1.40 1.34 1.26 1.18 1.08 1.03 0.97 0.95 0.90 0.88
0.97 1.00 1.00 0.93 1.95 0.98 0.98 1.14 3.22 0.97 1.46 2.46 2.07 3.21 2.86 1.24 2.55 9.20 2.93 6.61 6.69
6b
f1 (ppm)

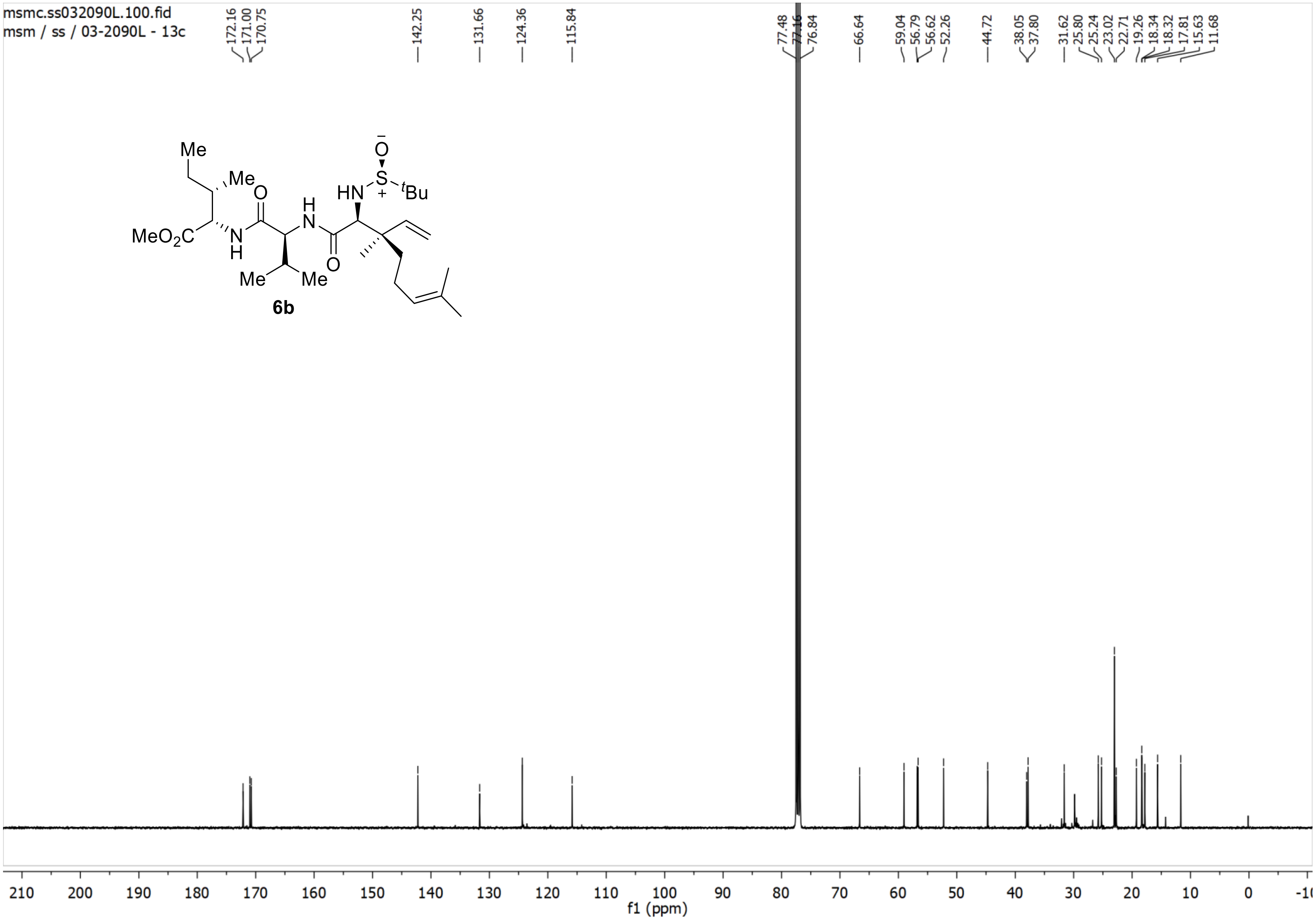

msmc.ss032090L.100.fid
msm / ss / 03-2090L - 13c
172.16
171.00
170.75
142.25
131.66
124.36
115.84
77.48
77.16
76.84
66.64
59.04
56.79
56.62
52.26
44.72
38.05
37.80
31.62
25.80
25.24
23.02
22.71
19.26
18.34
18.32
17.81
15.63
11.68
6b
f1 (ppm)

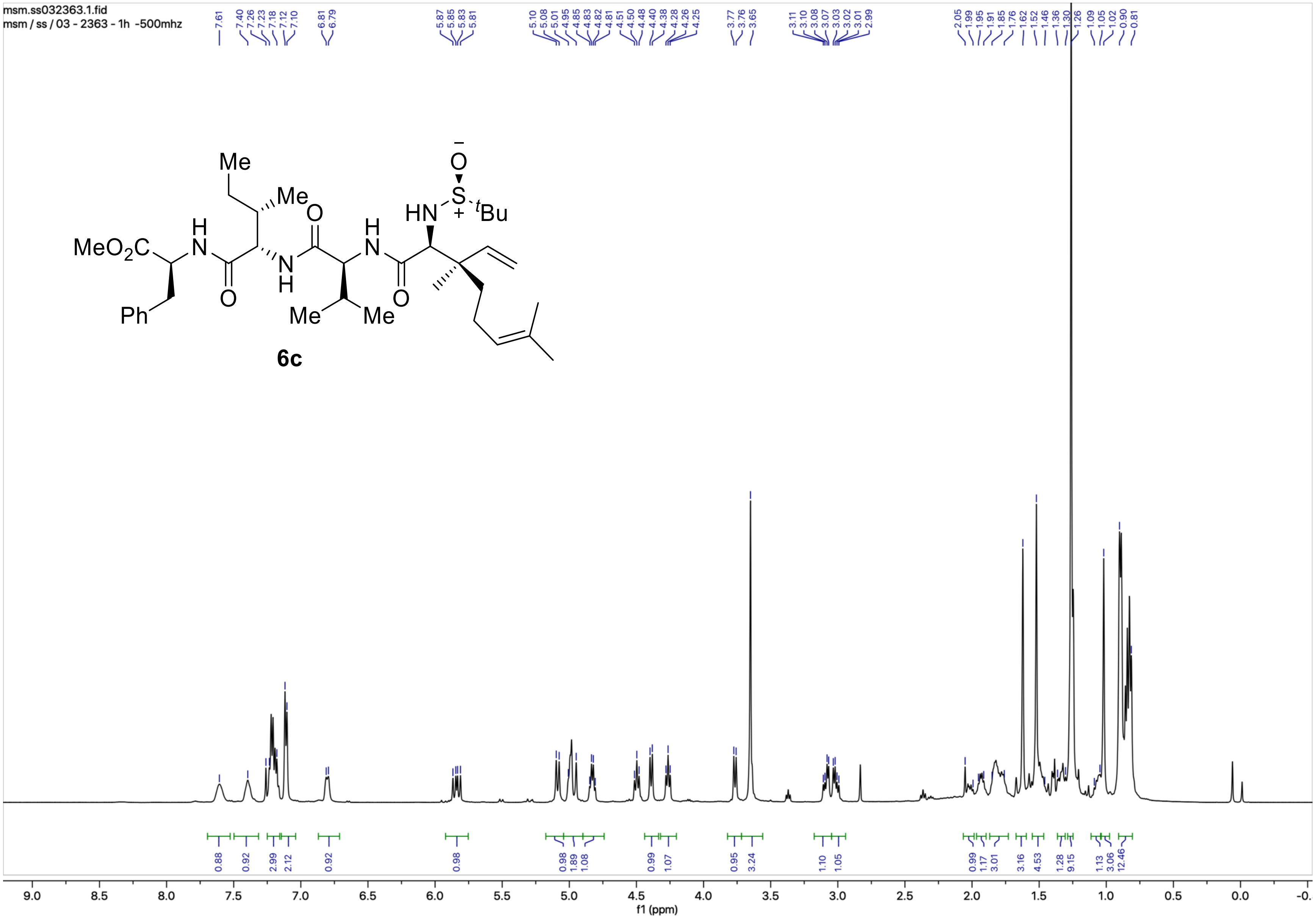
msm.ss032363.1.fid
msm / ss / 03 - 2363 - 1h -500mhz
6c
f1 (ppm)

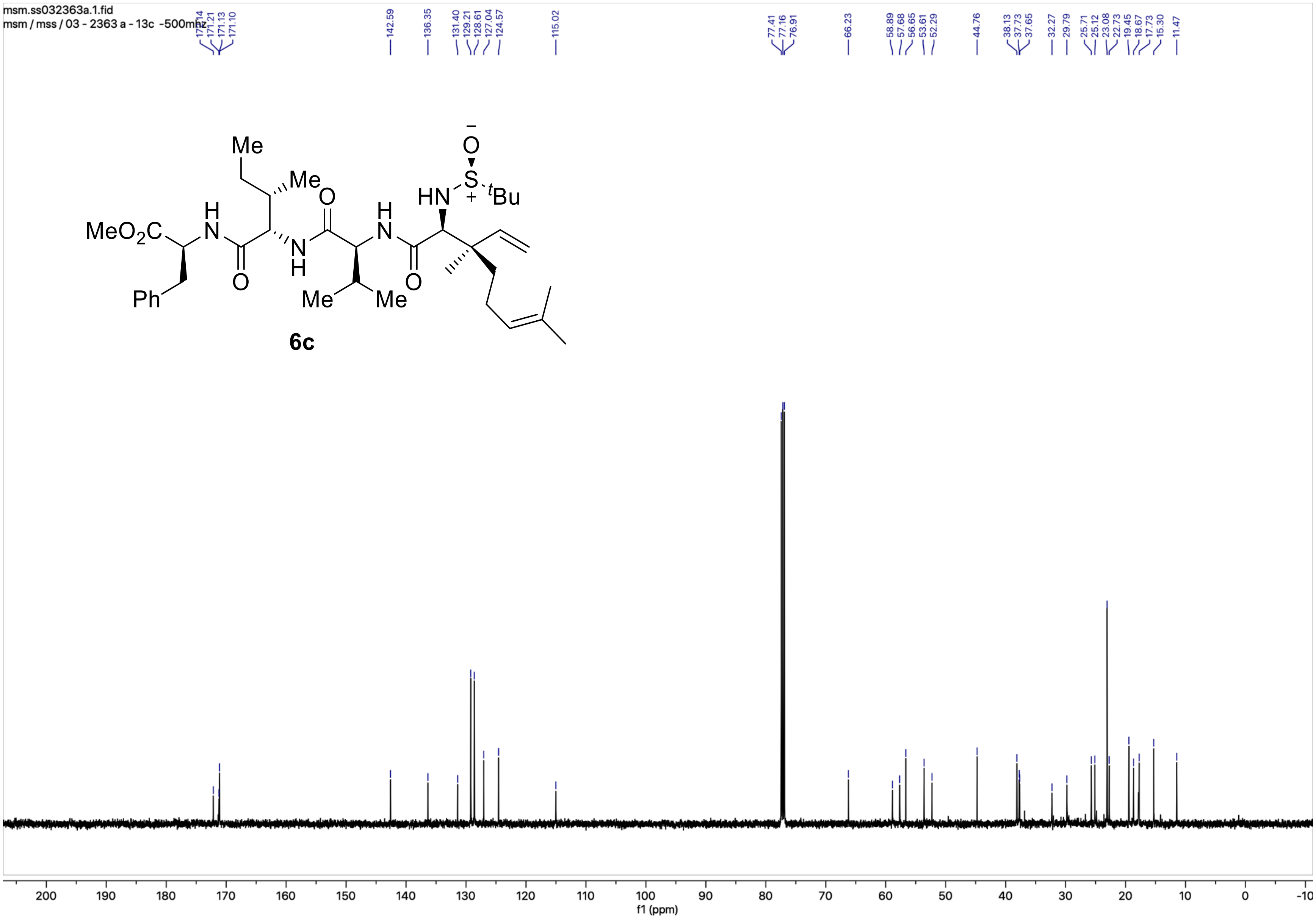
msm.ss032363a.1.fid
msm / mss / 03 - 2363 a - 13c -500mhz
172.14
171.21
171.13
171.10
142.59
136.35
131.40
129.21
128.61
127.04
124.57
115.02
77.41
77.16
76.91
66.23
58.89
57.68
56.65
53.61
52.29
44.76
38.13
37.73
37.65
32.27
29.79
25.71
25.12
23.08
22.73
19.45
18.67
17.73
15.30
11.47
6c
f1 (ppm)

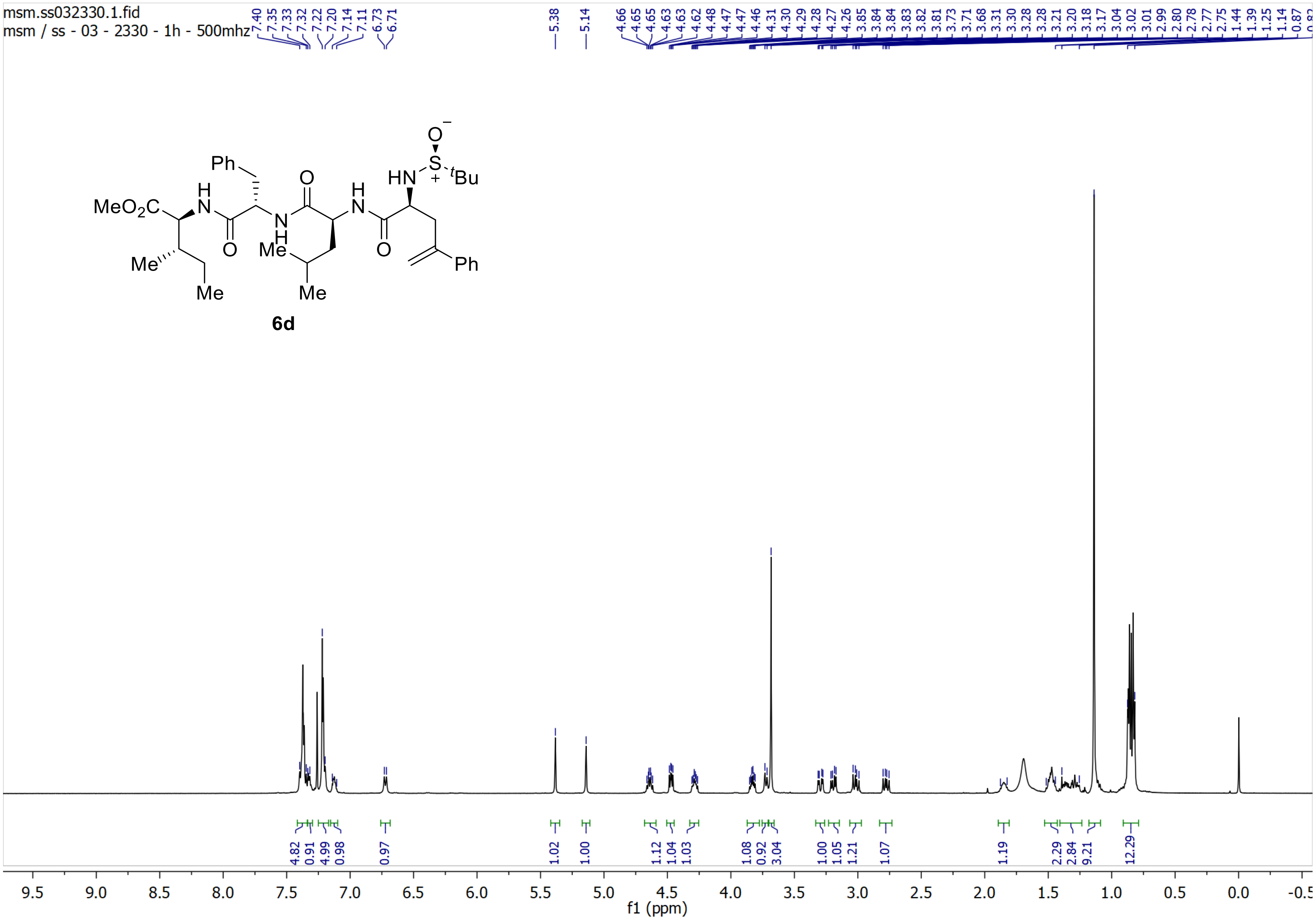
msm.ss032330.1.fid
msm / ss - 03 - 2330 - 1h - 500mhz
6d
f1 (ppm)

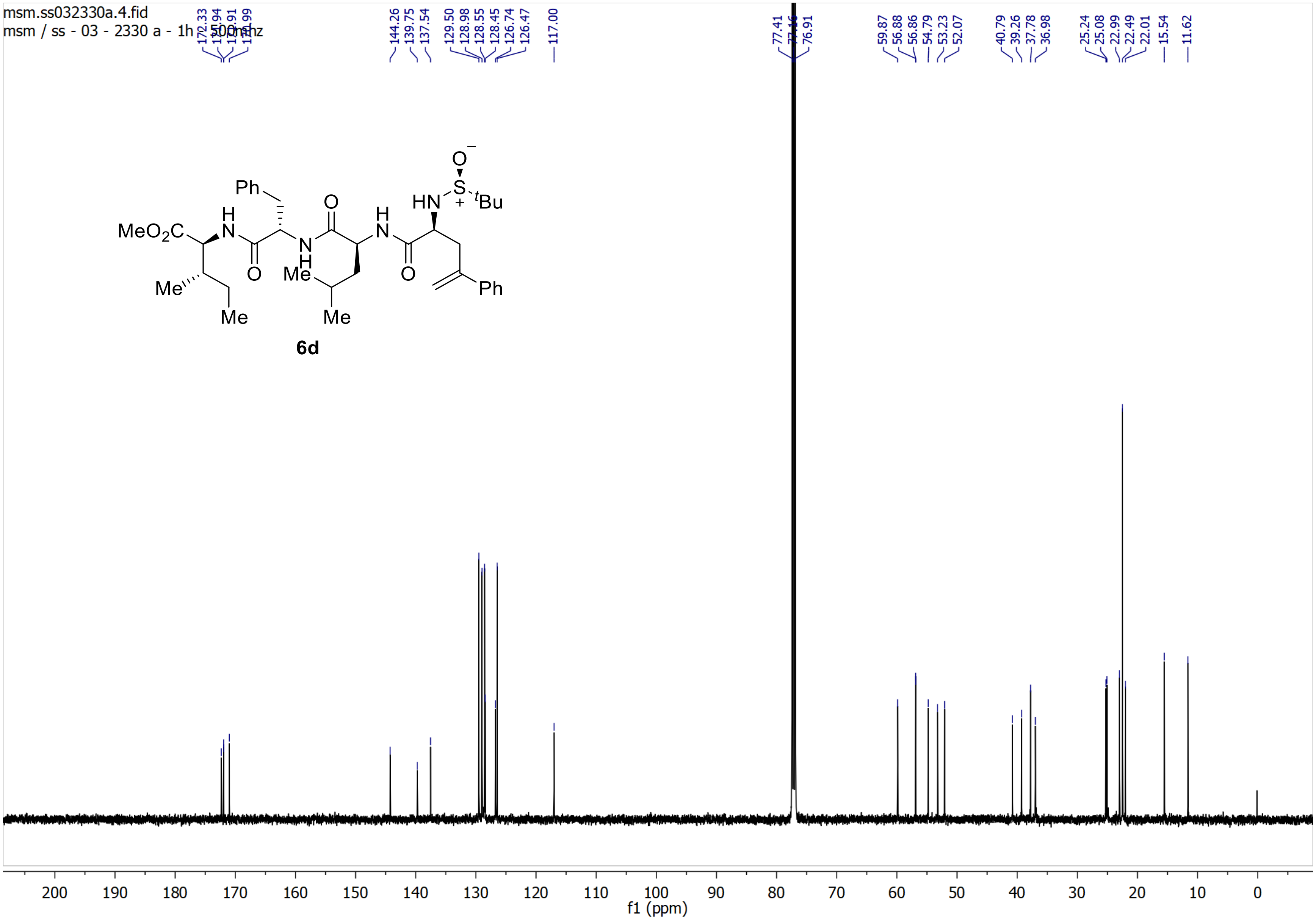
msm.ss032330a.4.fid
msm / ss - 03 - 2330 a - 1h - 500mhz
172.33
171.94
170.91
170.99
144.26
139.75
137.54
129.50
128.98
128.55
128.45
126.74
126.47
117.00
77.41
77.16
76.91
59.87
56.88
56.86
54.79
53.23
52.07
40.79
39.26
37.78
36.98
25.24
25.08
22.99
22.49
22.01
15.54
11.62
f1 (ppm)

6d

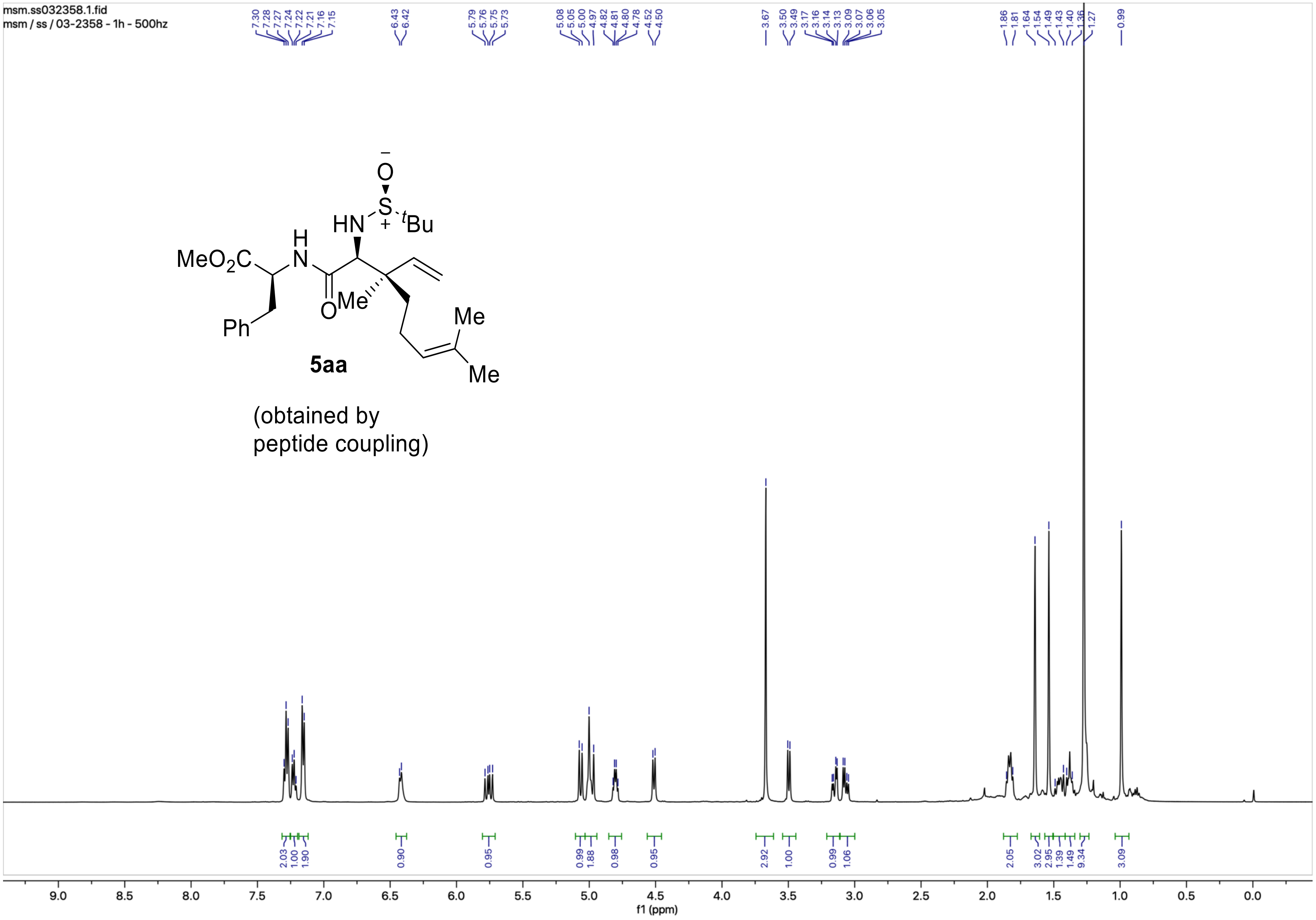
msm.ss032358.1.fid
msm / ss / 03-2358 - 1h - 500hz
7.30
7.28
7.27
7.24
7.22
7.21
7.16
7.15
6.43
6.42
5.79
5.76
5.75
5.73
5.08
5.05
5.00
4.97
4.82
4.81
4.80
4.78
4.52
4.50
3.67
3.50
3.49
3.17
3.16
3.14
3.13
3.09
3.07
3.06
3.05
1.86
1.81
1.64
1.54
1.49
1.43
1.40
1.36
1.27
0.99
MeO2C
H
N
HN
O
S
tBu
Me
Me
Me
O
Ph
5aa
(obtained by
peptide coupling)
2.03
1.00
1.90
0.90
0.95
0.99
1.88
0.98
0.95
2.92
1.00
0.99
1.06
2.05
3.02
2.95
1.39
1.49
9.34
3.09
9.0
8.5
8.0
7.5
7.0
6.5
6.0
5.5
5.0
4.5
4.0
3.5
3.0
2.5
2.0
1.5
1.0
0.5
0.0
f1 (ppm)

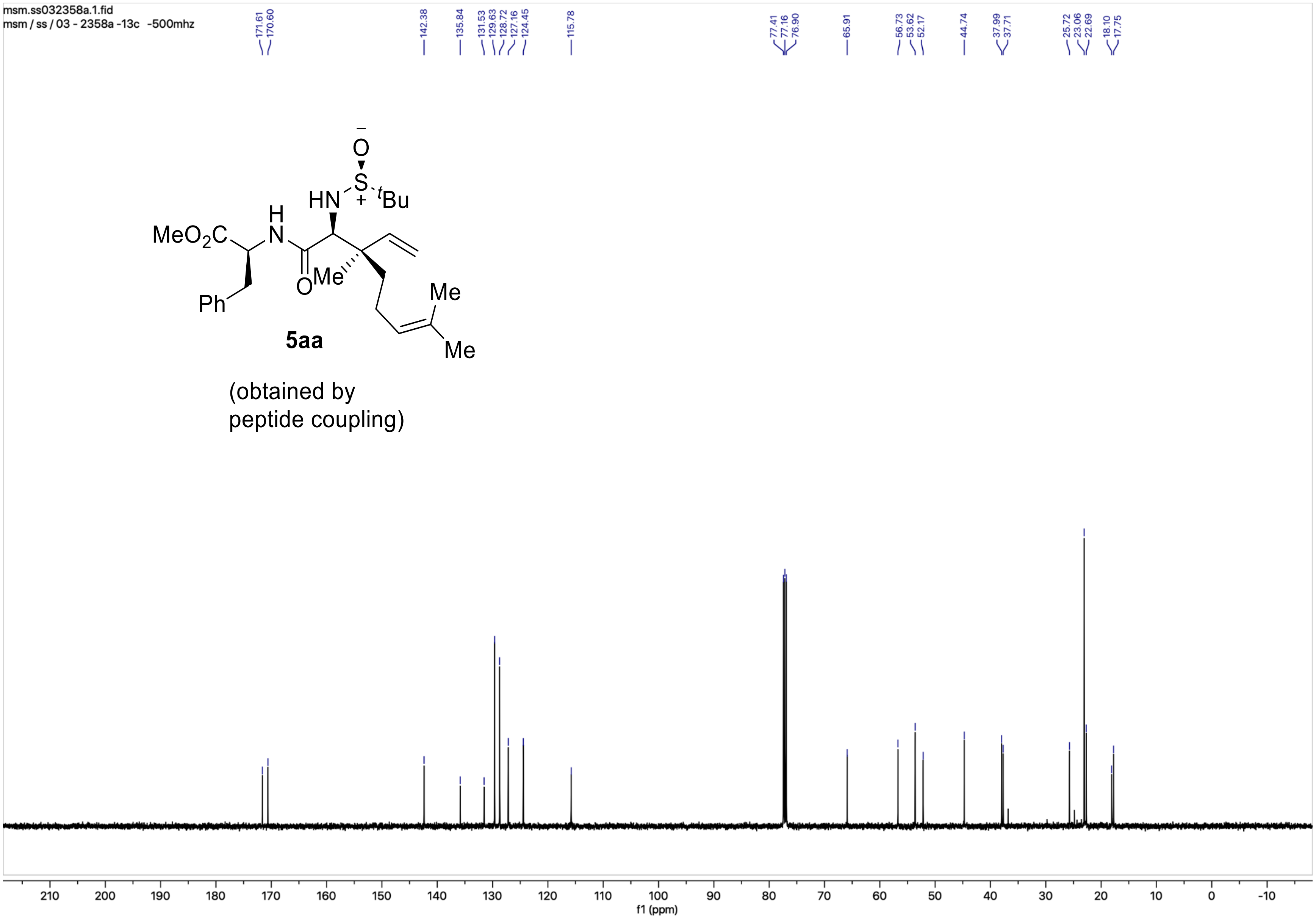
msm.ss032358a.1.fid
msm / ss / 03 - 2358a -13c -500mhz
171.61
170.60
142.38
135.84
131.53
129.63
128.72
127.16
124.45
115.78
77.41
77.16
76.90
65.91
56.73
53.62
52.17
44.74
37.99
37.71
25.72
23.06
22.69
18.10
17.75
5aa
(obtained by peptide coupling)
210 200 190 180 170 160 150 140 130 120 110 100 90 80 70 60 50 40 30 20 10 0 -10
f1 (ppm)

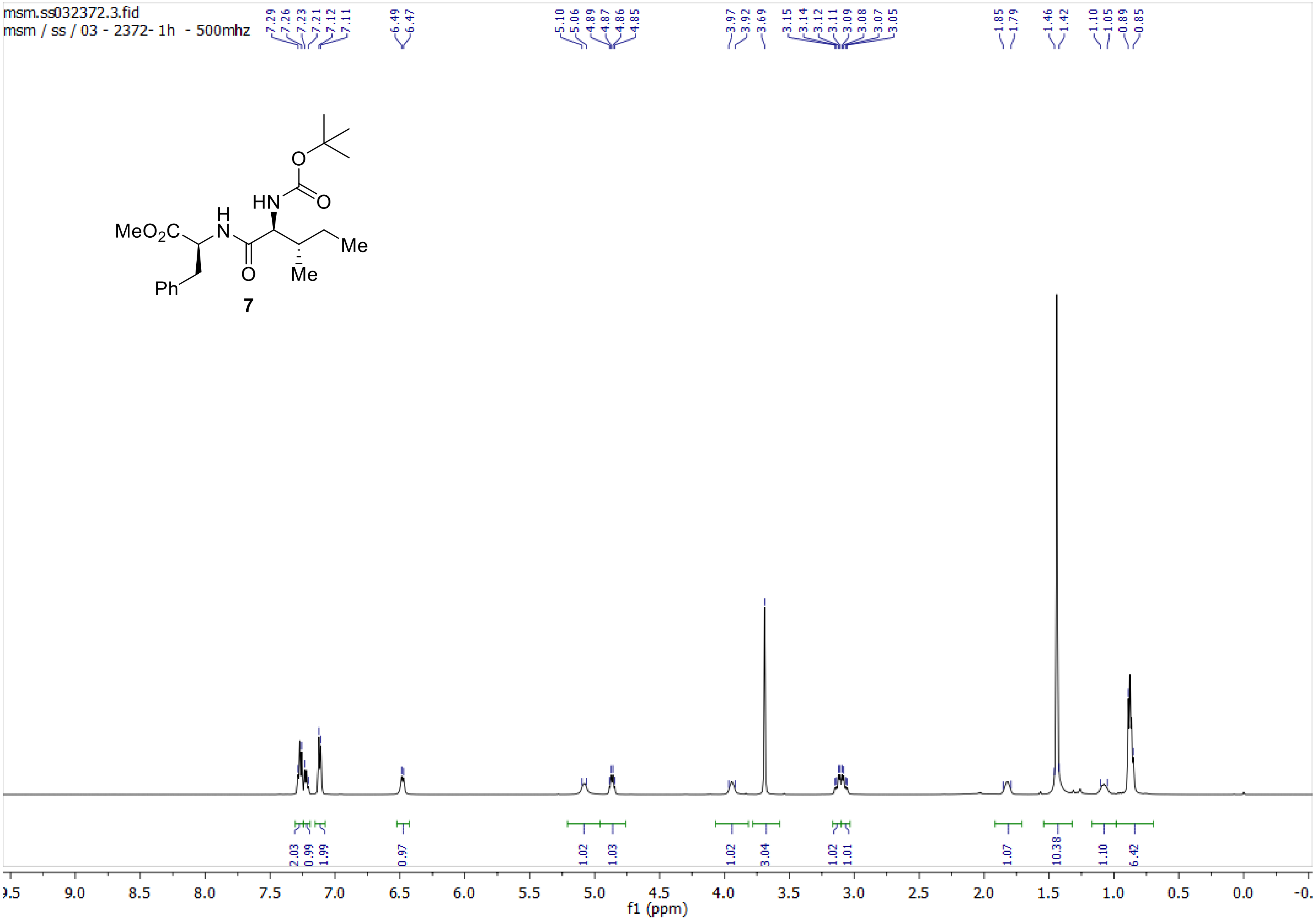
msm.ss032372.3.fid
msm / ss / 03 - 2372- 1h - 500mhz
7.29 7.26 7.23 7.21 7.12 7.11
6.49 6.47
5.10 5.06 4.89 4.87 4.86 4.85
3.97 3.92 3.69
3.15 3.14 3.12 3.11 3.09 3.08 3.07 3.05
1.85 1.79
1.46 1.42
1.10 1.05 0.89 0.85
MeO2C
H
N
HN
O
O
O
Me
Me
Ph
7
2.03 0.99 1.99 0.97 1.02 1.03 1.02 3.04 1.02 1.01 1.07 10.38 1.10 6.42
f1 (ppm)

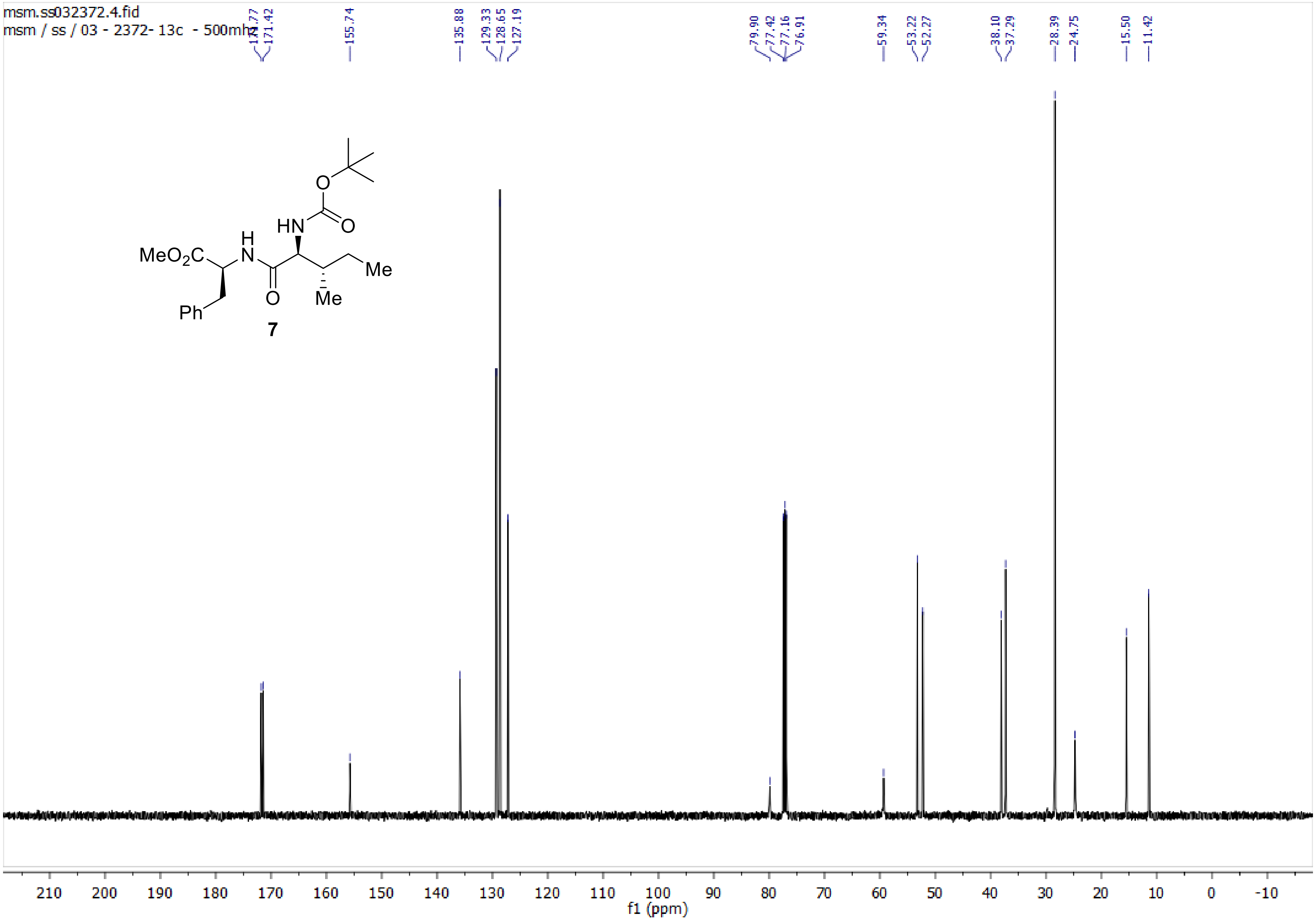

msm.ss032372.4.fid
msm / ss / 03 - 2372- 13c - 500mhz
171.42
155.74
135.88
129.33
128.65
127.19
79.90
77.42
77.16
76.91
59.34
53.22
52.27
38.10
37.29
28.39
24.75
15.50
11.42
MeO2C
H
N
HN
O
O
O
Me
Me
Ph
7
210
200
190
180
170
160
150
140
130
120
110
100
90
80
70
60
50
40
30
20
10
0
-10
f1 (ppm)

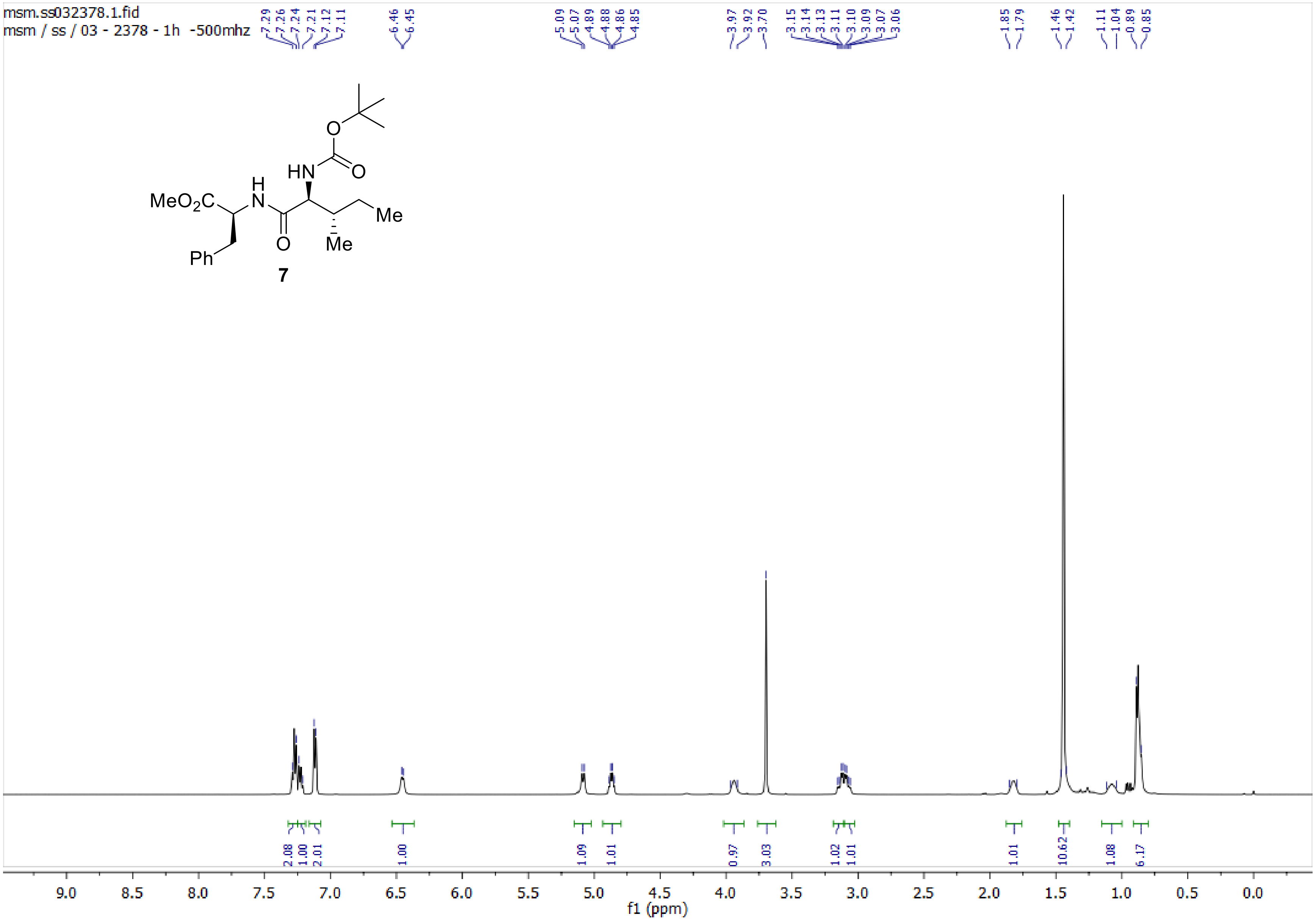
msm.ss032378.1.fid
msm / ss / 03 - 2378 - 1h -500mhz
7.29 7.26 7.24 7.21 7.12 7.11
6.46 6.45
5.09 5.07 4.89 4.88 4.86 4.85
3.97 3.92 3.70
3.15 3.14 3.13 3.11 3.10 3.09 3.07 3.06
1.85 1.79
1.46 1.42
1.11 1.04 0.89 0.85
MeO2C
H
N
HN
O
O
O
Me
Me
Ph
2.08 1.00 2.01 1.00 1.09 1.01 0.97 3.03 1.02 1.01 1.01 10.62 1.08 6.17
9.0 8.5 8.0 7.5 7.0 6.5 6.0 5.5 5.0 4.5 4.0 3.5 3.0 2.5 2.0 1.5 1.0 0.5 0.0
f1 (ppm)

7

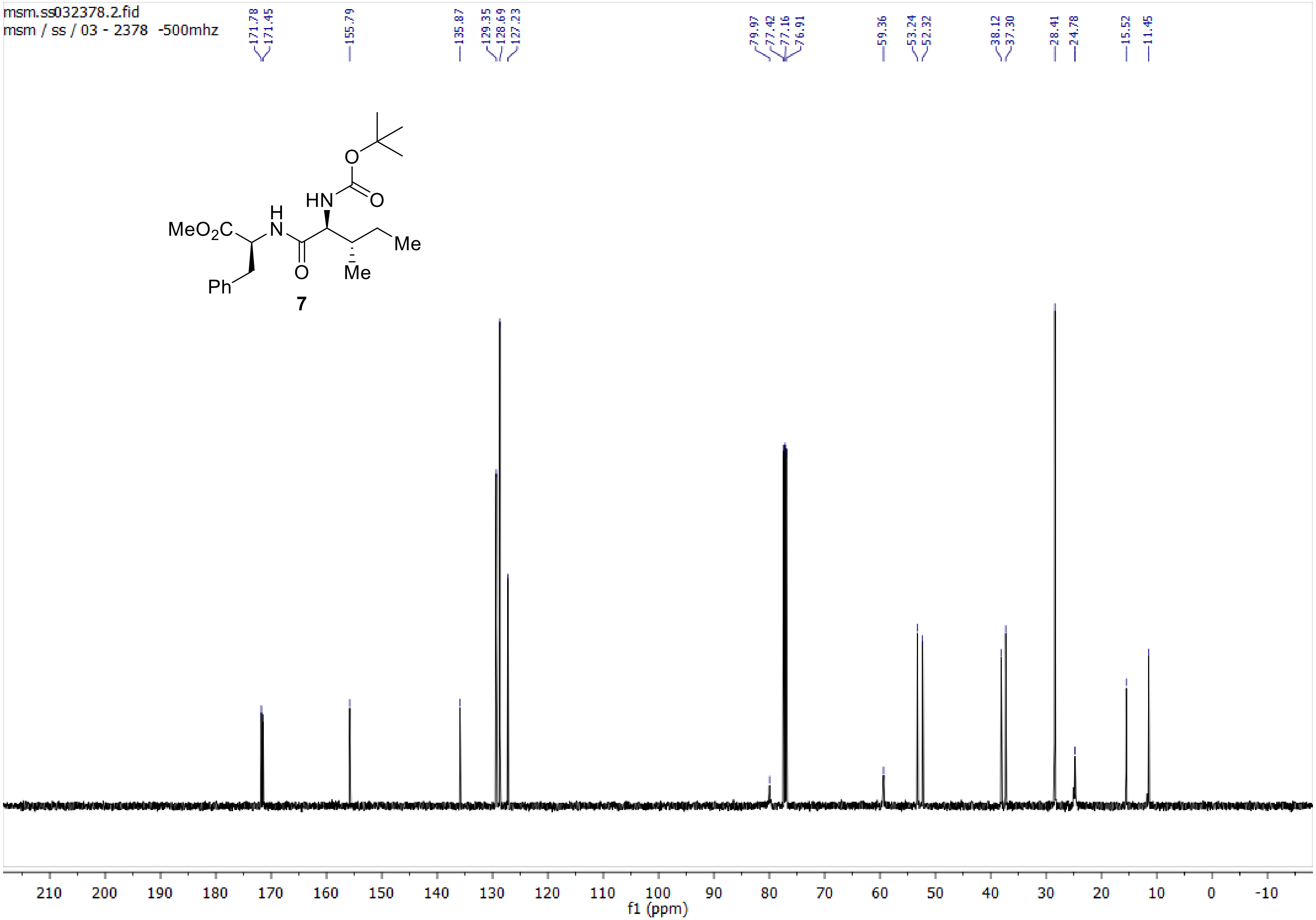
msm.ss032378.2.fid
msm / ss / 03 - 2378 -500mhz
171.78
171.45
155.79
135.87
129.35
128.69
127.23
79.97
77.42
77.16
76.91
59.36
53.24
52.32
38.12
37.30
28.41
24.78
15.52
11.45
MeO2C
H
N
HN
O
O
O
Me
Me
Ph
7
210
200
190
180
170
160
150
140
130
120
110
100
90
80
70
60
50
40
30
20
10
0
-10
f1 (ppm)

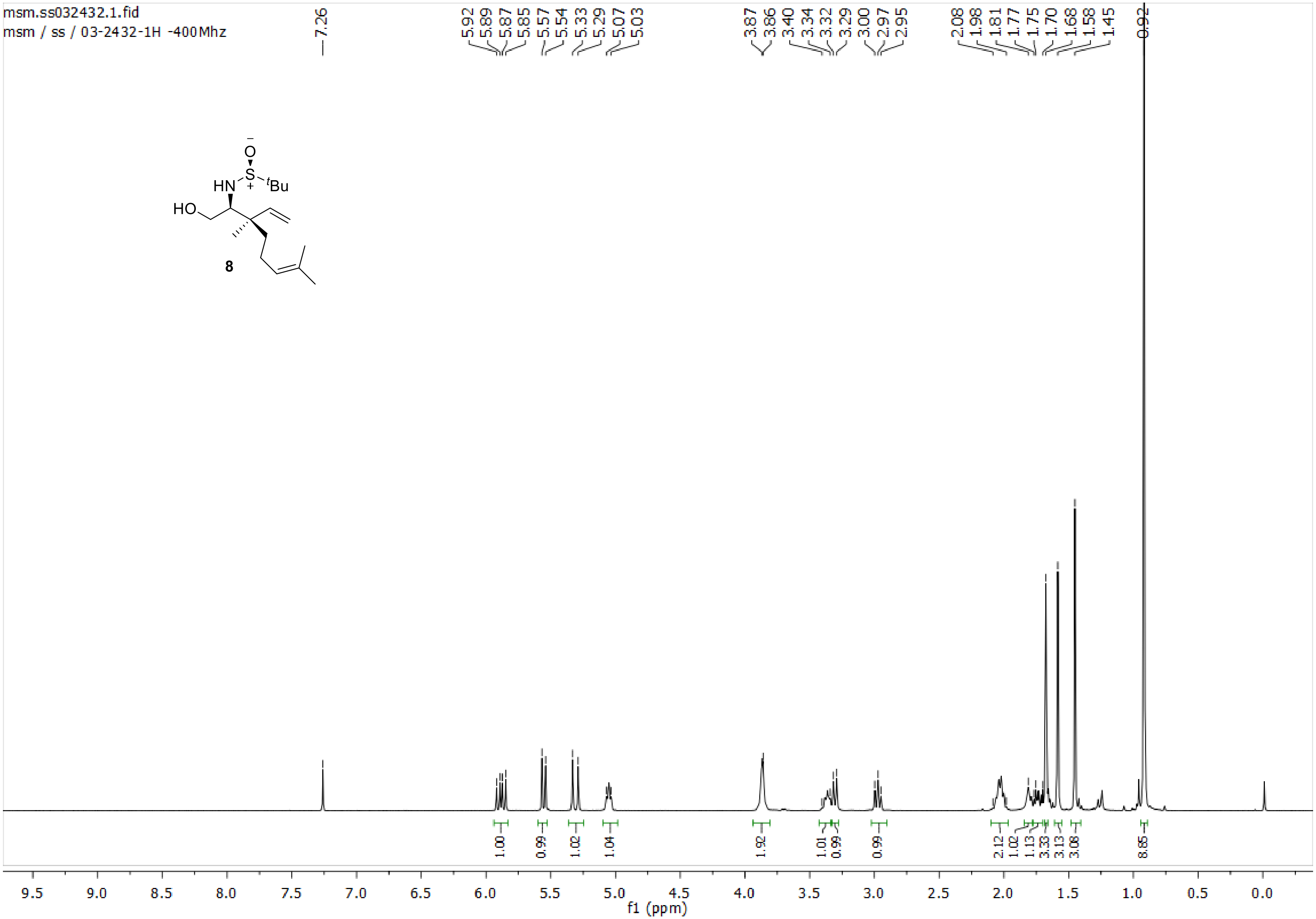
msm.ss032432.1.fid
msm / ss / 03-2432-1H -400Mhz
7.26
5.92 5.89 5.87 5.85 5.57 5.54 5.33 5.29 5.07 5.03
3.87 3.86 3.40 3.34 3.32 3.29 3.00 2.97 2.95
2.08 1.98 1.81 1.77 1.75 1.70 1.68 1.58 1.45
0.92
HO
HN
S
O
tBu
8
1.00 0.99 1.02 1.04 1.92 1.01 0.99 0.99 2.12 1.02 1.13 3.33 3.13 3.08 8.85
9.5 9.0 8.5 8.0 7.5 7.0 6.5 6.0 5.5 5.0 4.5 4.0 3.5 3.0 2.5 2.0 1.5 1.0 0.5 0.0
f1 (ppm)

msm.ss032432.2.fid
msm / ss / 03-2432-13C -400Mhz

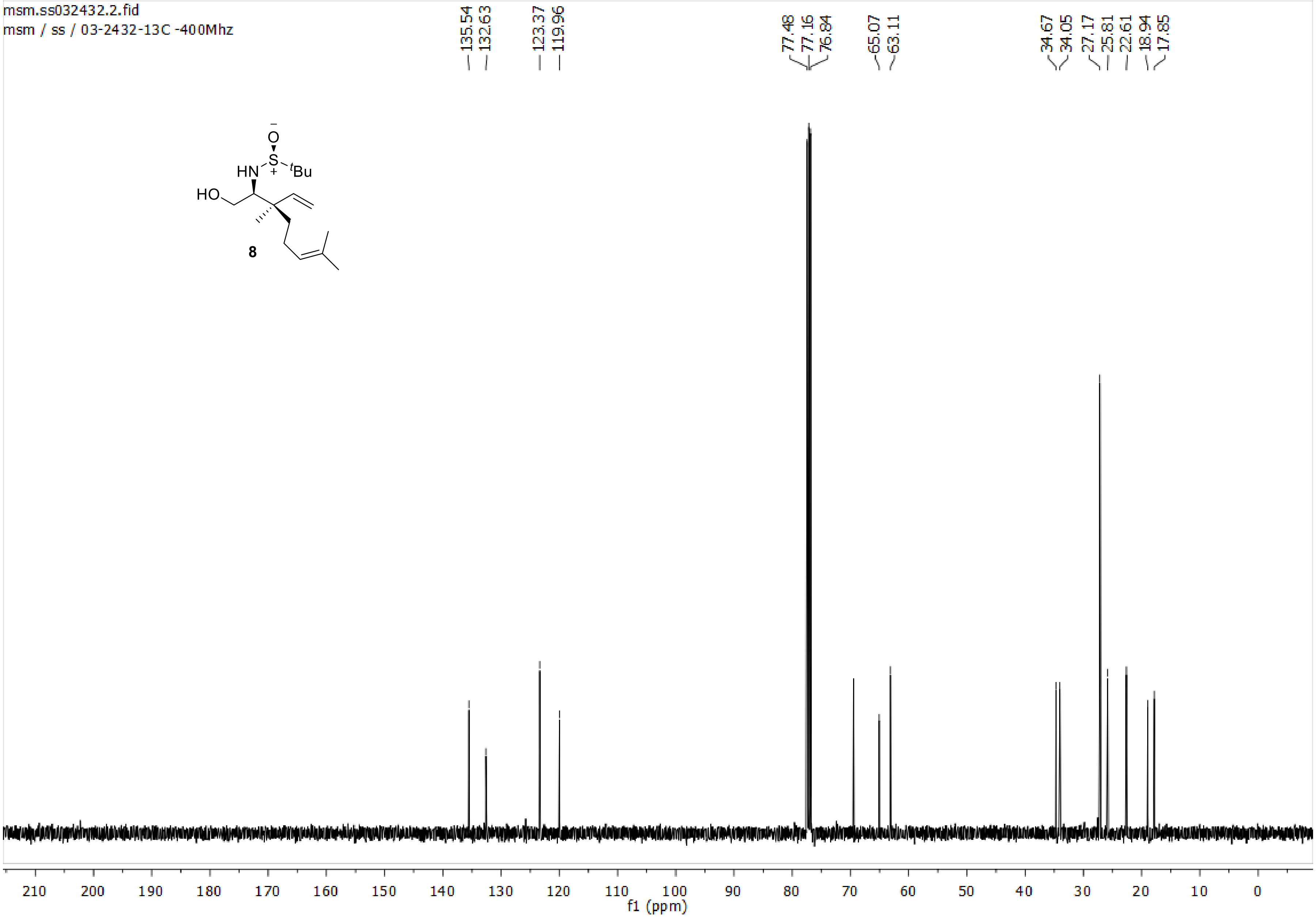

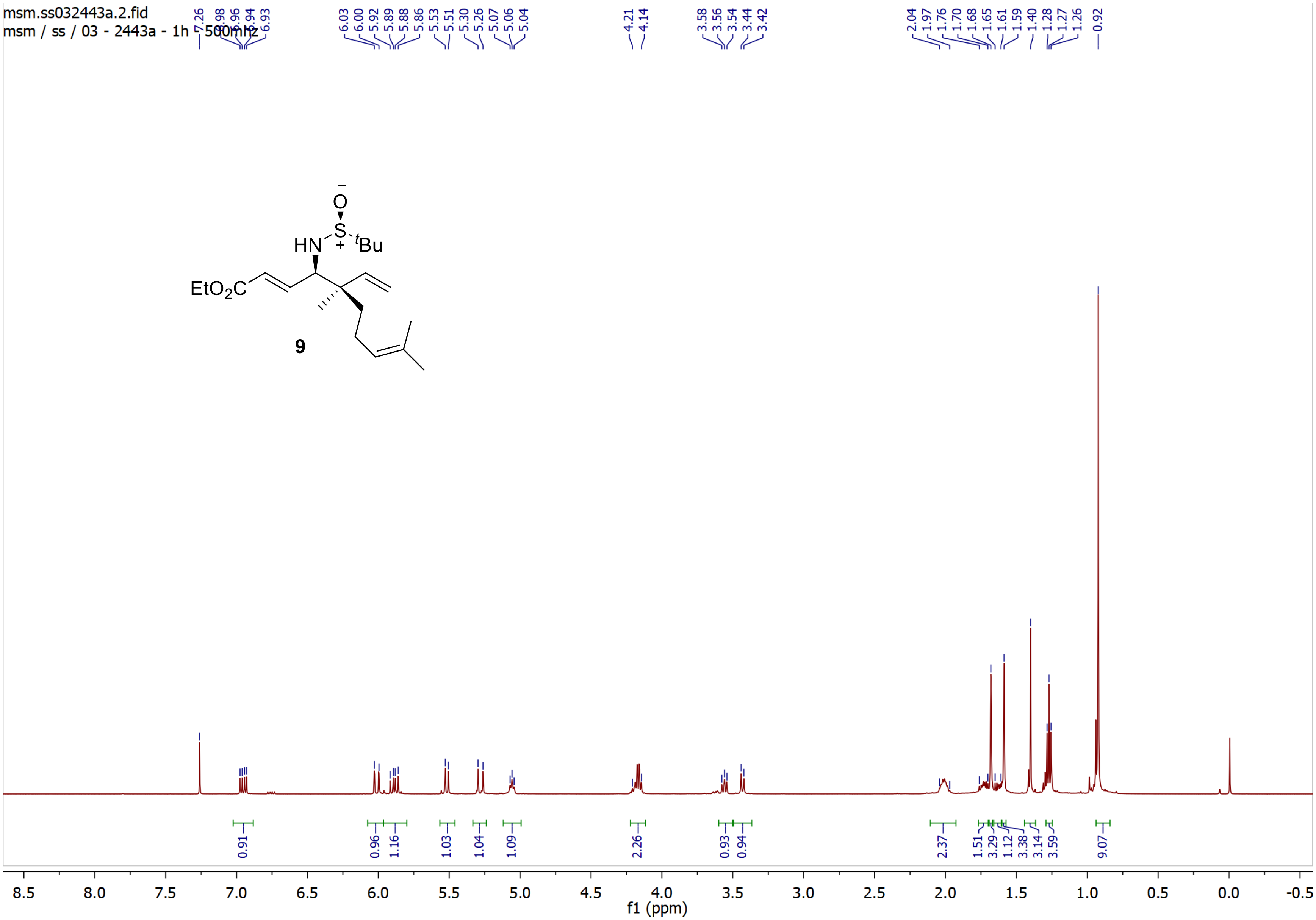
msm.ss032443a.2.fid
msm / ss / 03 - 2443a - 1h - 500mhz
7.26 6.98 6.96 6.94 6.93
6.03 6.00 5.92 5.89 5.88 5.86 5.53 5.51 5.30 5.26 5.07 5.06 5.04
4.21 4.14
3.58 3.56 3.54 3.44 3.42
2.04 1.97 1.76 1.70 1.68 1.65 1.61 1.59 1.40 1.28 1.27 1.26
0.92
HN
S
O
tBu
EtO2C
9
0.91 0.96 1.16 1.03 1.04 1.09 2.26 0.93 0.94 2.37 1.51 3.29 1.12 3.38 3.14 3.59 9.07
8.5 8.0 7.5 7.0 6.5 6.0 5.5 5.0 4.5 4.0 3.5 3.0 2.5 2.0 1.5 1.0 0.5 0.0 -0.5
f1 (ppm)

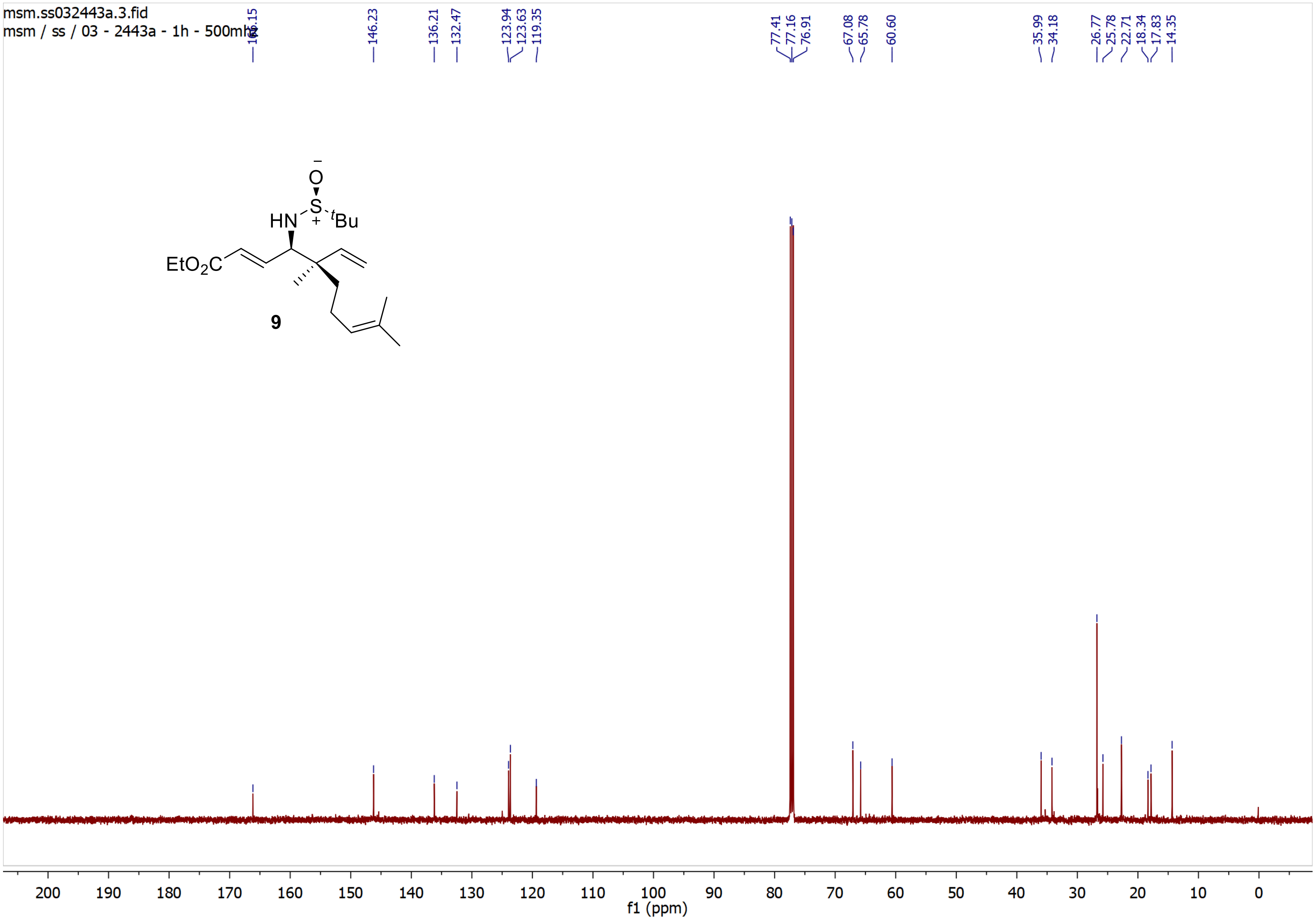

msm.ss032443a.3.fid
msm / ss / 03 - 2443a - 1h - 500mhz
146.23
136.21
132.47
123.94
123.63
119.35
77.41
77.16
76.91
67.08
65.78
60.60
35.99
34.18
26.77
25.78
22.71
18.34
17.83
14.35
O
S
HN
+
tBu
EtO_2C
9
200 190 180 170 160 150 140 130 120 110 100 90 80 70 60 50 40 30 20 10 0
f1 (ppm)

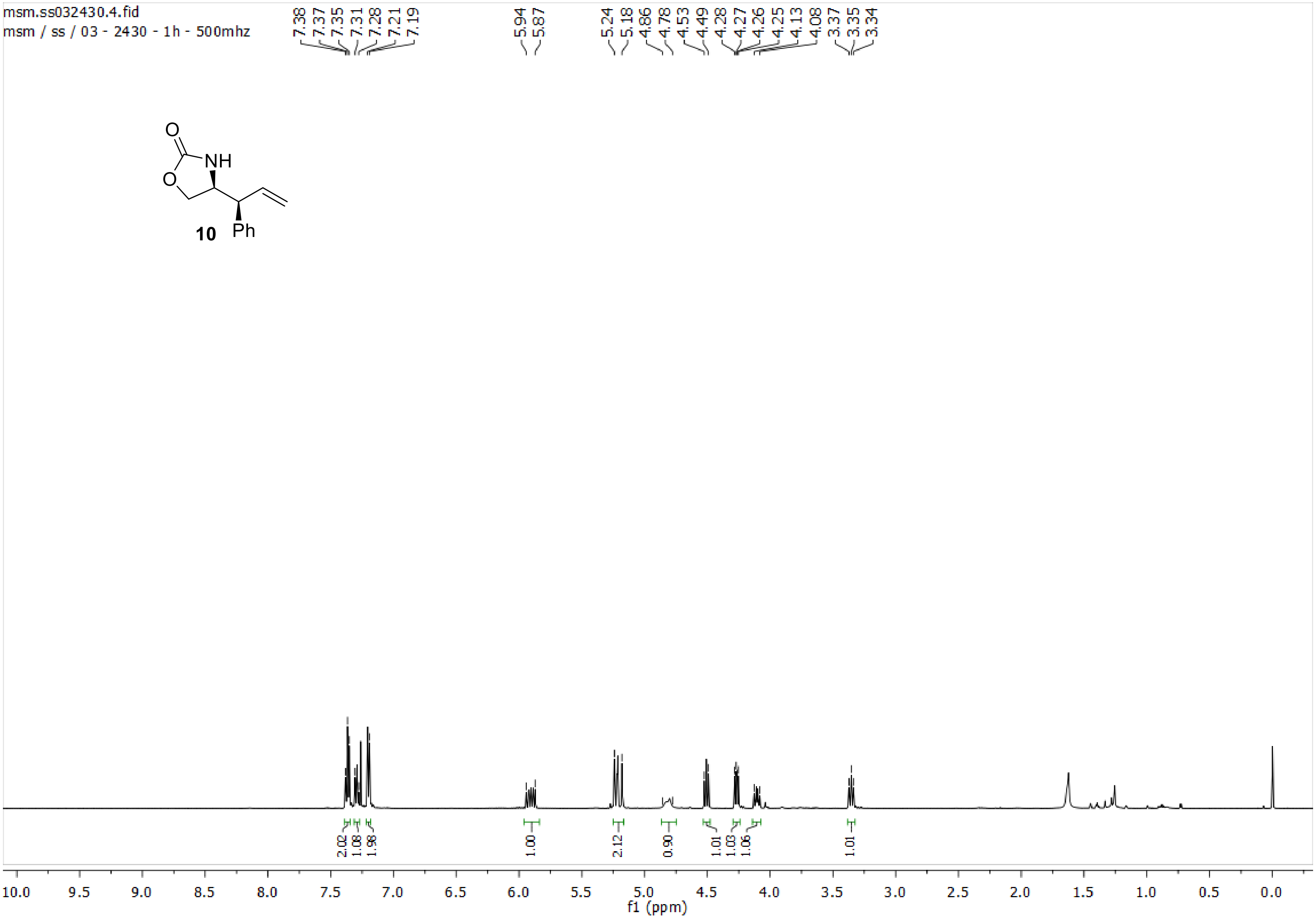
msm.ss032430.4.fid
msm / ss / 03 - 2430 - 1h - 500mhz
7.38
7.37
7.35
7.31
7.28
7.21
7.19
5.94
5.87
5.24
5.18
4.86
4.78
4.53
4.49
4.28
4.27
4.26
4.25
4.13
4.08
3.37
3.35
3.34
O
NH
O
10
Ph
2.02
1.08
1.98
1.00
2.12
0.90
1.01
1.03
1.06
1.01
10.0
9.5
9.0
8.5
8.0
7.5
7.0
6.5
6.0
5.5
5.0
4.5
4.0
3.5
3.0
2.5
2.0
1.5
1.0
0.5
0.0
f1 (ppm)

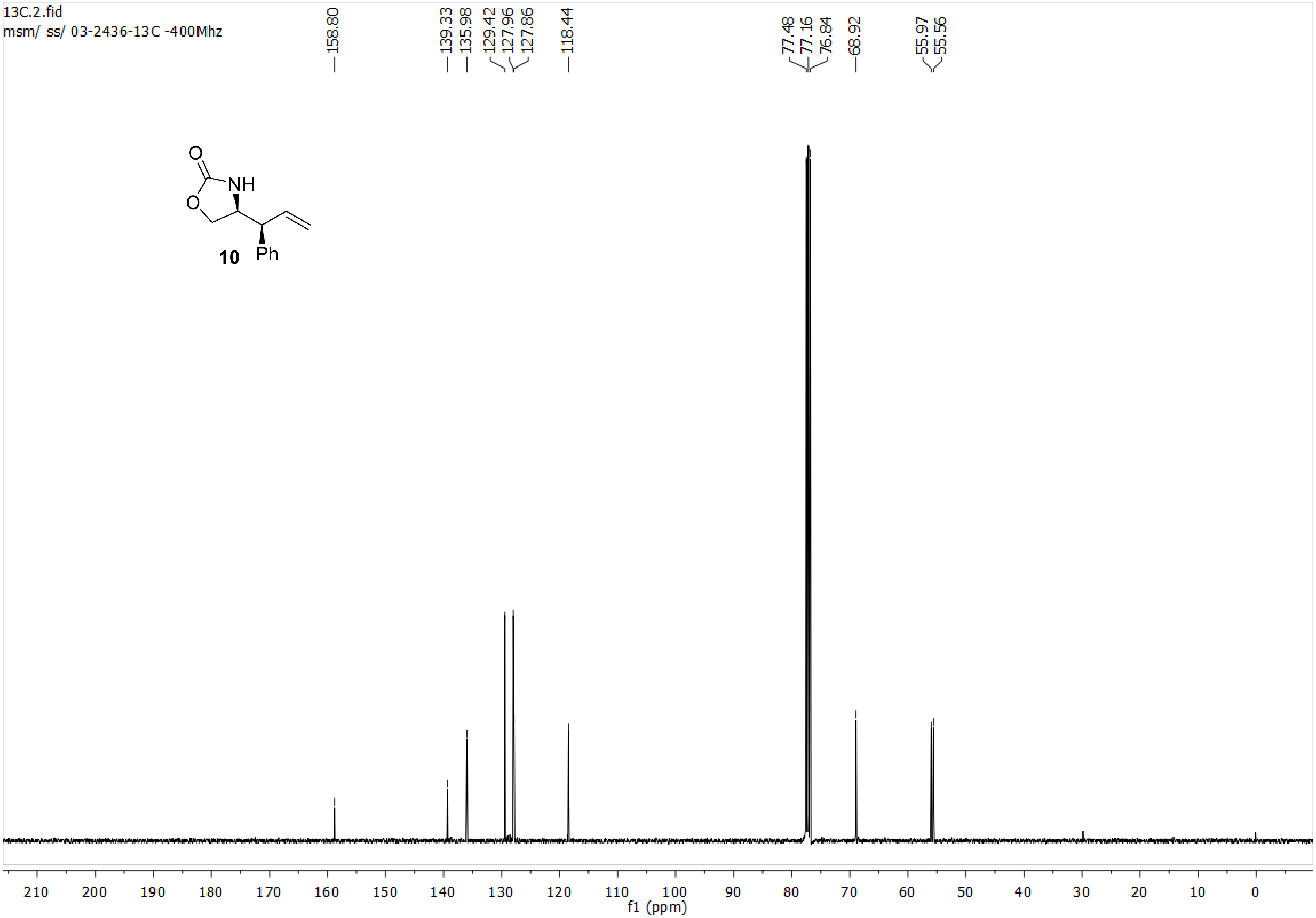
13C.2.fid
msm/ ss/ 03-2436-13C -400Mhz
158.80
139.33
135.98
129.42
127.96
127.86
118.44
77.48
77.16
76.84
68.92
55.97
55.56
O
NH
O
10
Ph
210 200 190 180 170 160 150 140 130 120 110 100 90 80 70 60 50 40 30 20 10 0
f1 (ppm)

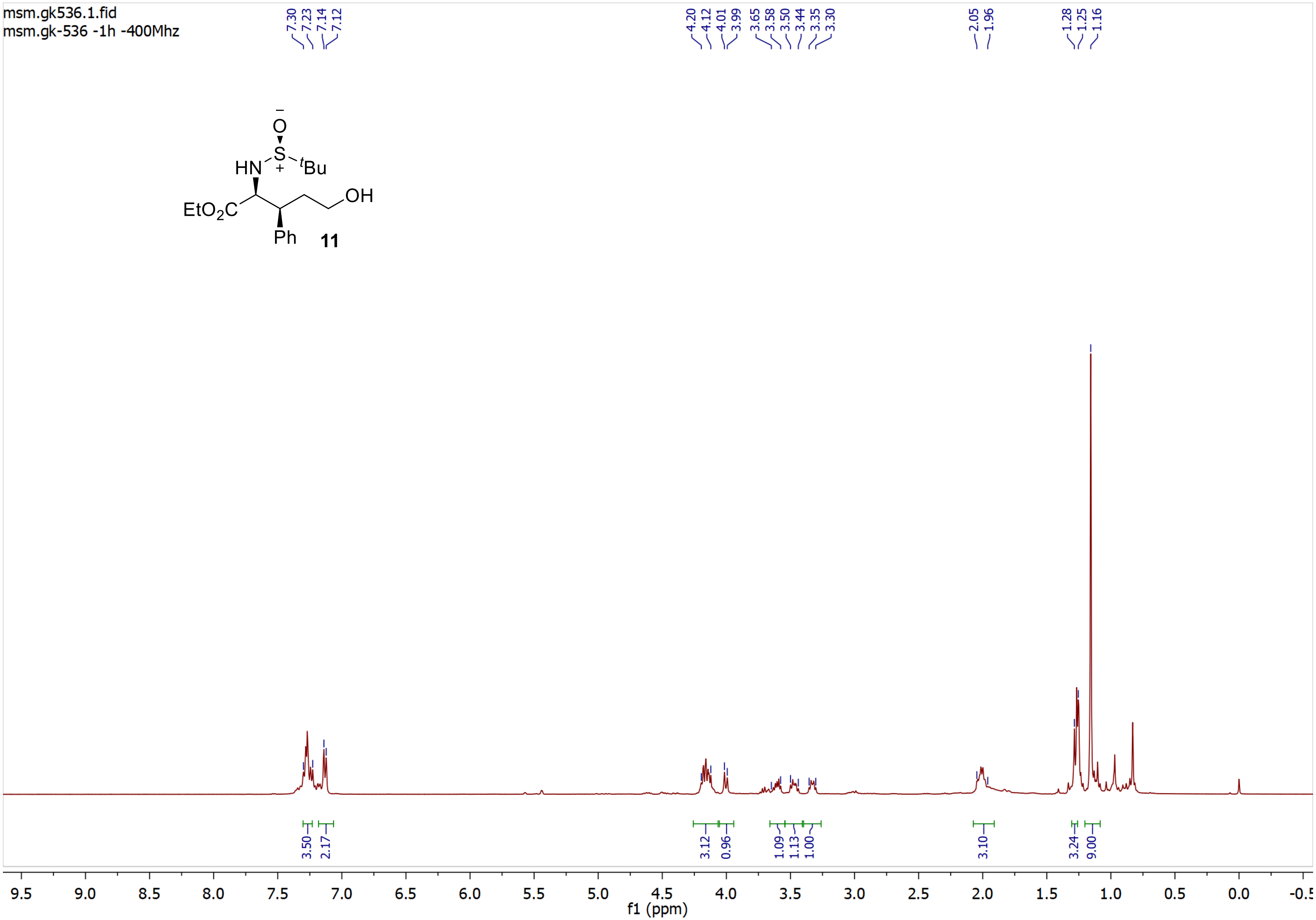
msm.gk536.1.fid
msm.gk-536 -1h -400Mhz
7.30
7.23
7.14
7.12
4.20
4.12
4.01
3.99
3.65
3.58
3.50
3.44
3.35
3.30
2.05
1.96
1.28
1.25
1.16
HN
S
O
tBu
EtO2C
OH
Ph
11
3.50
2.17
3.12
0.96
1.09
1.13
1.00
3.10
3.24
9.00
9.5
9.0
8.5
8.0
7.5
7.0
6.5
6.0
5.5
5.0
4.5
4.0
3.5
3.0
2.5
2.0
1.5
1.0
0.5
0.0
-0.5
f1 (ppm)

msm.gk536.2.fid
msm.gk536 - 13c - 500mhz

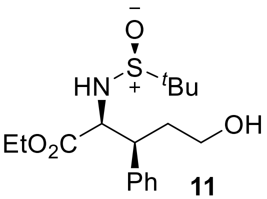

11

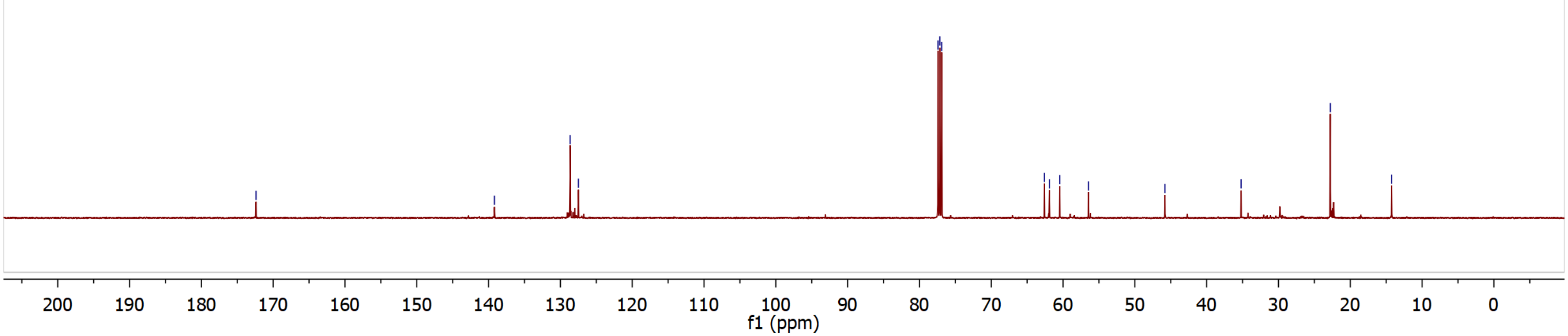

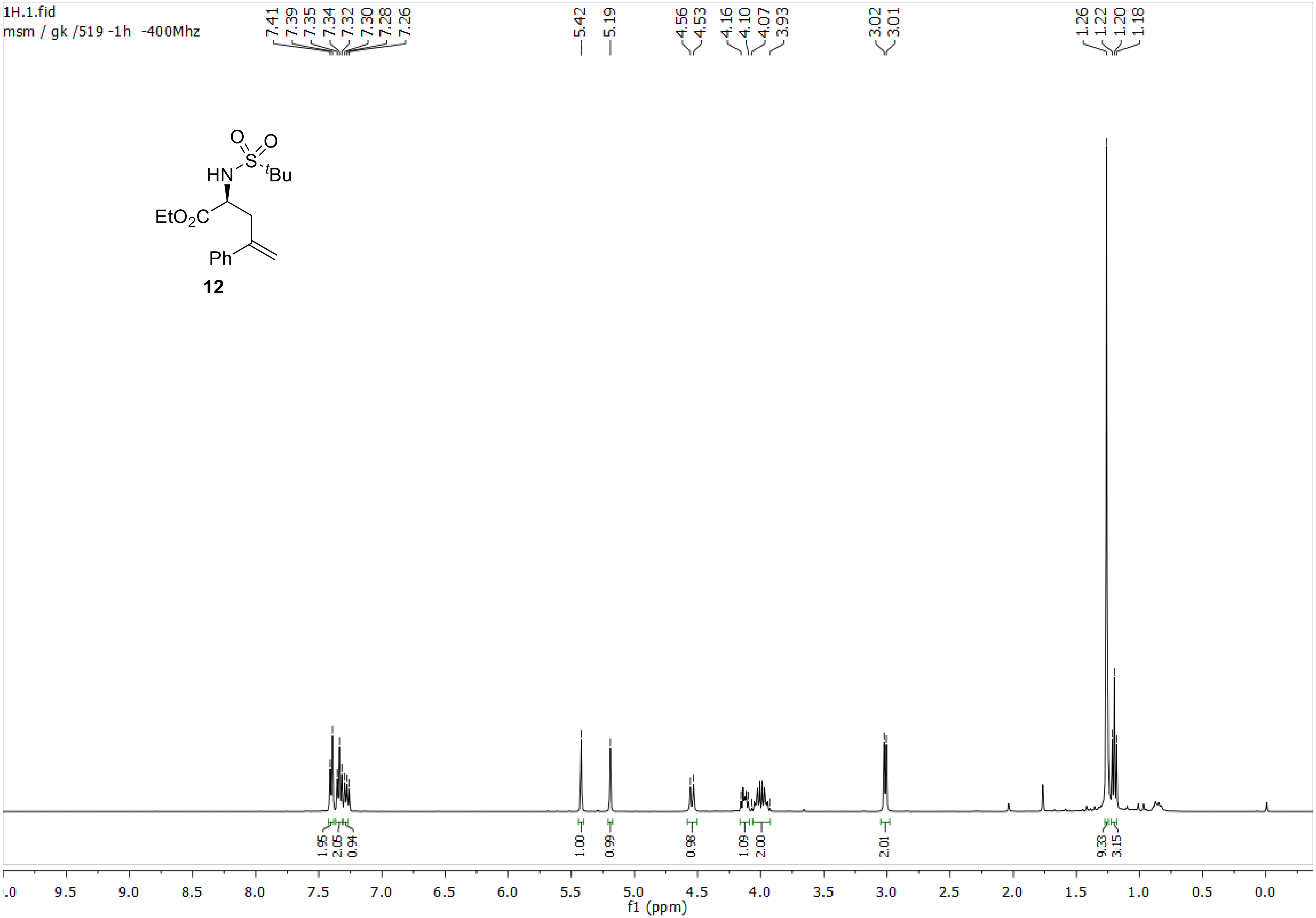

1H.1.fid
msm / gk /519 -1h -400Mhz
7.41 7.39 7.35 7.34 7.32 7.30 7.28 7.26
5.42
5.19
4.56 4.53
4.16 4.10 4.07 3.93
3.02 3.01
1.26 1.22 1.20 1.18
HN
S
O
O
tBu
EtO2C
Ph
12
1.95 2.05 0.94
1.00
0.99
0.98
1.09 2.00
2.01
9.33 3.15
f1 (ppm)

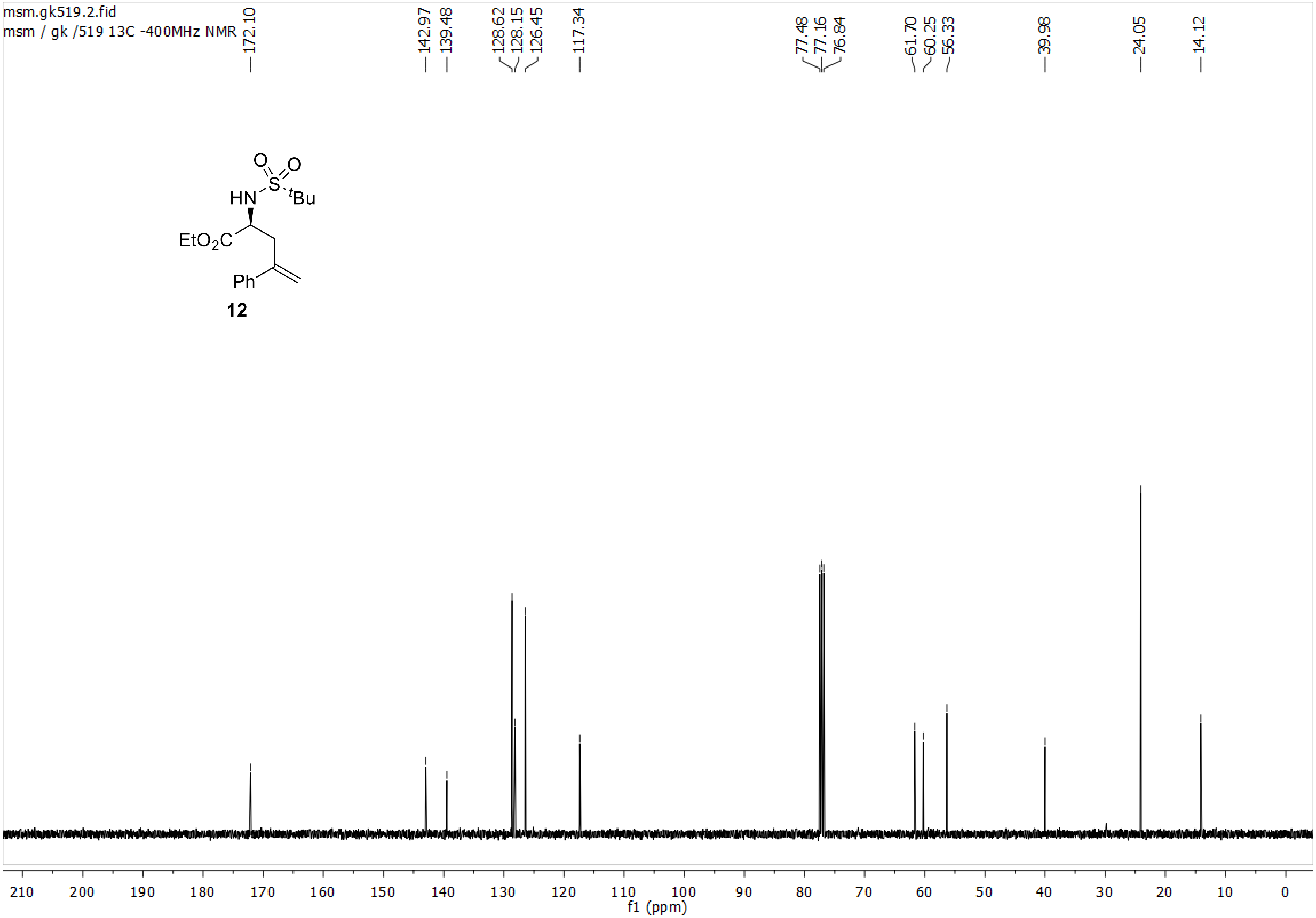
msm.gk519.2.fid
msm / gk /519 13C -400MHz NMR
172.10
142.97
139.48
128.62
128.15
126.45
117.34
77.48
77.16
76.84
61.70
60.25
56.33
39.98
24.05
14.12
HN
S
O
O
tBu
EtO2C
Ph
12
f1 (ppm)

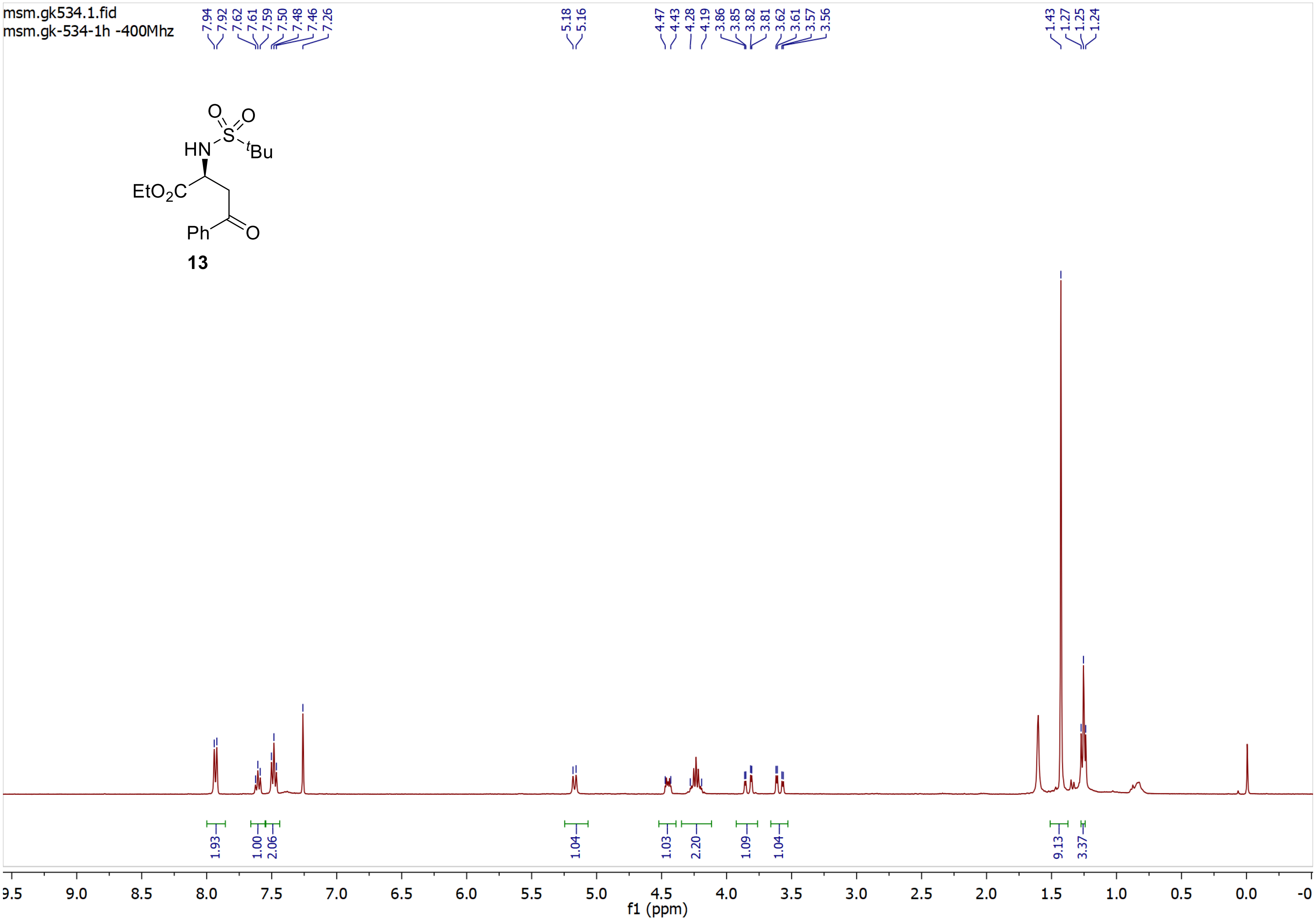
msm.gk534.1.fid
msm.gk-534-1h -400Mhz
7.94
7.92
7.62
7.61
7.59
7.50
7.48
7.46
7.26
5.18
5.16
4.47
4.43
4.28
4.19
3.86
3.85
3.82
3.81
3.62
3.61
3.57
3.56
1.43
1.27
1.25
1.24
HN
S
O
O
tBu
EtO2C
Ph
O
13
1.93
1.00
2.06
1.04
1.03
2.20
1.09
1.04
9.13
3.37
f1 (ppm)

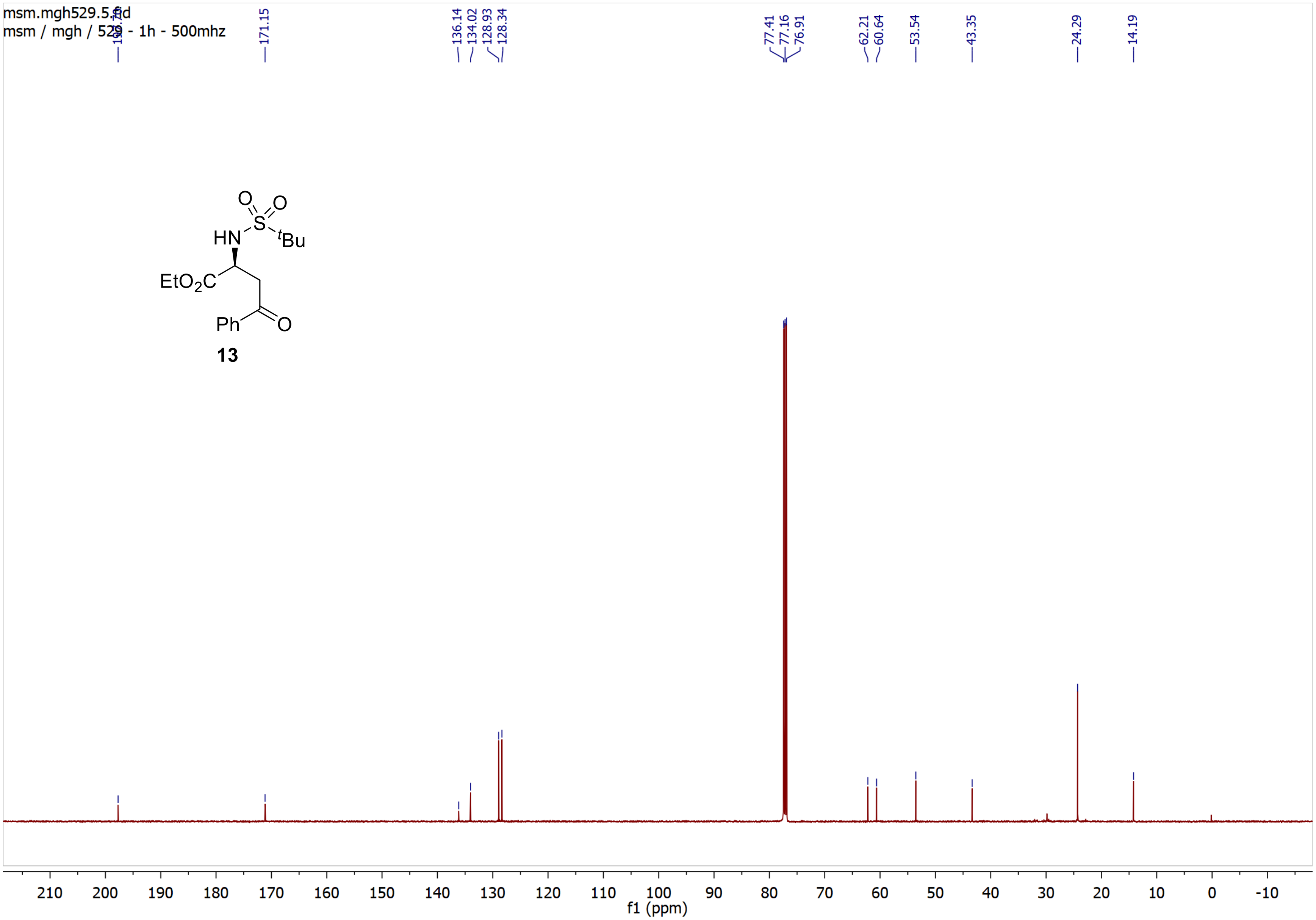
msm.mgh529.5.fid
msm / mgh / 529 - 1h - 500mhz
171.15
136.14
134.02
128.93
128.34
77.41
77.16
76.91
62.21
60.64
53.54
43.35
24.29
14.19
HN
EtO2C
tBu
Ph
13
210 200 190 180 170 160 150 140 130 120 110 100 90 80 70 60 50 40 30 20 10 0 -10
f1 (ppm)
